# Prokaryotic Diversity in the Rhizosphere of Organic, Intensive, and Transitional Coffee Farms in Brazil

**DOI:** 10.1371/journal.pone.0106355

**Published:** 2015-06-17

**Authors:** Adam Collins Caldwell, Lívia Carneiro Fidéles Silva, Cynthia Canêdo da Silva, Cleber Costa Ouverney

**Affiliations:** 1 Department of Biological Sciences, San Jose State University, San Jose, California, 95192–0100, United States of America; 2 Department of Microbiology, Federal University of Viçosa, Viçosa, MG, 36570, Brazil; Graz University of Technology (TU Graz), AUSTRIA

## Abstract

Despite a continuous rise in consumption of coffee over the past 60 years and recent studies showing positive benefits linked to human health, intensive coffee farming practices have been associated with environmental damage, risks to human health, and reductions in biodiversity. In contrast, organic farming has become an increasingly popular alternative, with both environmental and health benefits. This study aimed to characterize and determine the differences in the prokaryotic soil microbiology of three Brazilian coffee farms: one practicing intensive farming, one practicing organic farming, and one undergoing a transition from intensive to organic practices. Soil samples were collected from 20 coffee plant rhizospheres (soil directly influenced by the plant root exudates) and 10 control sites (soil 5 m away from the coffee plantation) at each of the three farms for a total of 90 samples. Profiling of 16S rRNA gene V4 regions revealed high levels of prokaryotic diversity in all three farms, with thousands of species level operational taxonomic units identified in each farm. Additionally, a statistically significant difference was found between each farm’s coffee rhizosphere microbiome, as well as between coffee rhizosphere soils and control soils. Two groups of prokaryotes associated with the nitrogen cycle, the archaeal genus *Candidatus* Nitrososphaera and the bacterial order Rhizobiales were found to be abundant and statistically different in composition between the three farms and in inverse relationship to each other. Many of the nitrogen-fixing genera known to enhance plant growth were found in low numbers (e.g. *Rhizobium*, *Agrobacter*, *Acetobacter*, *Rhodospirillum*, *Azospirillum*), but the families in which they belong had some of the highest relative abundance in the dataset, suggesting many new groups may exist in these samples that can be further studied as potential plant growth-promoting bacteria to improve coffee production while diminishing negative environmental impacts.

## Introduction

Coffee consumption has increased nearly 20 times between 1952 and 2011 [1]. To supply the demand many countries, including Brazil, have adopted intensive agricultural practices, with heavy use of chemical fertilizers and an array of chemical treatments to combat pests (insecticides, pesticides, fungicides) and competing plants (herbicides), all of which have a negative impact on the environment [2–4]. Although coffee consumption has been linked to numerous physiological benefits [5–8], several studies have associated pesticides or herbicides in coffee agriculture to health problems [9, 10], including various types of cancer [11]. More recently, organic farming, which removes the use of chemical treatments [12], has provided a healthier alternative that benefits consumers, farmers, and the environment.

Brazil has been a leading country in coffee production for the past 150 years, growing about one third of the world’s coffee [13]. Over 50% of that productivity happens in the southeastern state of Minas Gerais, where the coffee variety Arabica (*Coffea arabica*) is the most common [14]. Despite being a major economic activity, little is known about the bacterial community composition associated with soil of coffee plantations and how that community differs between intensive and organic agricultural practices. The soil microbiome is quite diverse with an estimated 6,400–38,000 taxa per gram of soil [15]. Biological species diversity has long been thought to confer benefits in sustainability, with increased diversity providing resistance to stress, disturbance, and changing soil conditions [16]. Traditionally, studies of bacterial diversity were limited to culture based studies, but since ≥99% of bacteria are estimated to be uncultivable [17], culture independent methods to study diversity such as use of the 16S rRNA marker gene have come into widespread use [18, 19].

Bacteria that enhance plant growth, referred to as plant growth-promoting bacteria (PGPB), have been characterized primarily based on culture methods [20], including the PGPB associated with the coffee rhizosphere. PGPB in the coffee rhizosphere can increase crop production by acting as plant growth promoters or supplying plants with nutrients, as demonstrated with isolates such as the [21, 22]phosphate-solubilizing bacteria [23] and nitrogen-fixing bacteria [8]. *Acetobacter diazotrophicus* was one of the first nitrogen-fixing bacteria to be associated with coffee plants, being found in plant tissues as well as in the rhizosphere [8]. Additionally, *Achromobacter*, *Stenotrophomonas*, and *Leifsonia* isolates were associated with enhanced Robusta coffee (*Coffea canephora*) [24], which represents 30% of Brazil’s national coffee production, only second to Arabica with the remaining 70%.

A small study showed the rhizosphere of Arabica coffee to be dominated by the genera *Bacillus*, *Pseudomonas*, *Micrococcus*, *Serratia*, and *Flavobacterium*, whereas Robusta coffee was dominated by *Bacillus*, *Pseudomonas*, and *Flavobacterium*, both listed in decreasing order of abundance [25]. An in-depth investigation applying high throughput next-generation sequencing to provide a deeper characterization of the coffee-associated rhizobacteria community is lacking.

Farming has a substantial impact on the environment. Soil degradation and erosion, pressure on limited water supplies, and reliance on fossil fuel based fertilizers and pesticides are all issues identified with current agricultural processes. Climate change is also greatly affected by agriculture, with 12–14% of greenhouse gas emissions estimated to be due to agriculture, primarily due to production and use of conventional nitrogen fertilizers [26]. Organic farming has been suggested as one method to mitigate some of these environmental concerns, though the environmental and socioeconomic impacts of organic versus conventional farming are complex [27]. While biodiversity and soil organic content increase under organic farming in general, the effects on nutrient leaching and greenhouse gas emissions are complicated by increased land use due to reduced yields [28]. In coffee production, similar results have been found. Organic cultivation methods were associated with reduced greenhouse gas emissions per kg of coffee, but due to lower yields, organic farming required more land usage to generate comparable production to conventionally farmed coffee, with the associated environmental implications [29]. However, a large-scale study in Costa Rica, covering thousands of farms, showed benefits to organic certification of coffee farms due to reduction in chemical inputs (pesticides, herbicides, and inorganic fertilizers) and adoption of environmentally friendly management practices [30].

The goal of this project was to differentiate the diversity and composition of microbial communities associated with the rhizosphere of coffee plants between organic and pesticide-treated coffee farms using next generation sequencing technology. This study expects to provide a first investigation of such differences in coffee soils and identify potential biological markers for future microbial soil manipulations.

## Materials and Methods

### Sample Sites

Our study was conducted in private land. No specific permissions were required from an authority. Soil samples were collected from three coffee farms in the region of Zona da Mata, located in the southeastern part of the State of Minas Gerais, Brazil, during the harvesting season. All three farms are located within a 5.5 km radius, near the mountain range of the second highest peak in Brazil, “Pico da Bandeira,” (Portuguese for *Flag Peak*; elevation 2,892m). The region has been explored for coffee farms since the early 1830s due to its propitious hilly topography, mild temperatures (18.8°C annual average temperature; annual average range 12.4°C—25.9°C), and precipitation patterns (1,340 mm annual average precipitation; annual range 1,000–1,680 mm). The soil type is known as latosols, yellowish-red in color from iron and aluminum oxide deposits.

Most coffee in Brazil is produced in this region and each of the three farms from which samples were collected for this study grew the same coffee variety, the *Coffea arabica* L., (cultivar Red Catuai, IAC-44) a breed developed by the Campinas Agronomy Institute (IAC in Portuguese) for higher yield in Brazilian soil and climate conditions [31, 32] and higher resistance to fungal diseases [33, 34]. Even though each farm has belonged to the same respective family for at least 3 generations, each showed a distinct method of farming practice. The intensive farm (INT) practices heavy use of pesticides, herbicides, fertilizers, and other treatments of the soil on an annual cycle. The fertilizer NPK 20-05-20 (by Heringer Fertilizantes, Manhuaçu, MG, Brazil) is applied three times per year (October, December, and February). NPK 20-05-20 stands for 20% nitrogen, 5% phosphate, and 20% potassium. The primary pesticide used at the INT farm is IMPACT (by Cheminova, Lemvig, Denmark), with active ingredient flutriafol to combat fungal diseases such as the common coffee leaf rust and coffee berry disease [33, 35]. The pH of the soil in the intensive farm is controlled semi-annually by the addition of lime once the pH falls below 5.5. The transitional (TRN) farm on the other hand switched to organic farming 4 years prior to our sample collection and within that period has been using natural compost as the fertilizer with no addition of pesticides or herbicides. In addition other crops such as manioc (*Manihot esculenta*, aka cassava) or corn are planted among the coffee trees to enhance soil health quality, possibly by enriching soil with nitrogen-fixing bacteria. Four years prior to organic practices, TRN soils were treated with pesticides, herbicide and chemical fertilizers. Neighboring farmers still apply pesticides, which could potentially be carried in air to the TRN farm. The organic farm (ORG) practiced no use of chemical pesticides, herbicides or industrialized fertilizers for over 18 years. It is believed this land was a forest before turning into a farm and was never under the direct influence of chemical pesticides or fertilizers. Natural compost has been the primary fertilizer at ORG and, similar to the TRN farm, other crops were planted within the coffee crops such as manioc.

General characteristics of each farm were collected at site and included the crop density, measured by the distance between each coffee tree in a row and the distance between each row; the age of each farm; the number of coffee trees planted; and the coffee yield, measured as the number of 60 kg coffee bags produced per number of coffee trees harvested. In addition the coordinates for each site was taken using GPS on a mobile device (Table 1). The yield of coffee production for the year we collected the samples varied among the three farms as follows: the intensive farm yield ranked highest with 1.4 x 10^−2^ bags/tree, followed by the organic farm with 1.2 x 10^−2^ bags/tree, while the transitional farm had the lowest yield with 1.0 x 10^−2^ bags/tree.

**Table 1:** Farm characteristics.

Site	Planting density (m)^a^	Latitude (South)	Longitude (West)	Age (years)	Coffee trees	Coffee production (60 kg bags)
**Intensive (INT)**	0.5 x 3.5	-20.619836	-42.137103	10	32,000	450
**Transitional (TRN)**	1.0 x 2.0	-20.644278	-42.196235	4	3,000	30
**Organic (ORG)**	2.0 x 4.0	-20.593598	-42.153856	18	5,000	60

Collected from interviews.

^a^ Distance between each coffee tree in a row versus distance between rows (in meter).

### Sample Collection and Soil Chemical Analysis

A total of 90 samples were collected for genomic DNA extraction, 30 samples from each of the three farms (INT, TRN, ORG), where 20 samples were from rhizosphere soil, next to the coffee tree trunk, and 10 control soil samples were collected 5 meters away from the coffee plantation. All samples were collected 2 cm below the surface in sterile Whirl-Pak Bags (Nasco, Fort Atkinson, WI). Samples were kept and transported in ice from each farm and stored at -20°C until DNA extractions. The TRN site had no bare soil next to the plantation and instead grass was growing in the control soil, hence samples were collected from the grass rhizosphere.

Separate soil chemical analyses were done between the rhizosphere and the control soil areas at each farm, one analysis per area, for a total of four analyses among all three farms (S1 Table). Sample collection and preparation for chemical analyses followed the standard protocol used by the local farmers as instructed by the laboratory carrying out the analyses (Manhuaçu Laboratório de Análise de Solos, Manhuaçu, MG). Basically, 20 single samples of approximately equal weight and equally distributed within the study area, were collected in Whirl-Pak Bags, then combined in a clean container, and homogenized. Finally, 250 g of the homogenate from each of the four study areas were submitted for chemical analysis. Results for the soil chemical analyses are shown in S1 Table. In general, the rhizosphere of each farm had a higher pH than its control soil counterpart. A soil pH between 5.5 and 6.0 is considered adequate for coffee plantation. The intensive farm had the highest pH (6.22) among all soils tested, perhaps due to the application of lime to the soil to control pH. The organic farm rhizosphere had the second highest pH (6.10). Similar to pH, nutrients such as phosphorous (P), calcium (Ca^2+^), and magnesium (Mg^2+^) were higher in the rhizosphere samples as compared to the respective control soil. There was no clear pattern where one farm had consistent higher concentration of available nutrients over the other.

### Genomic DNA Extraction and Purity

Genomic DNA extraction was done within 24 h of the last collection using the MO-BIO PowerSoil DNA Extraction Kit (cat 12888–100). Extraction followed manufacturer recommendations on 0.25 g of soil from each sample. DNA quantification (ng/μl) and purity were determined using a NanoDrop-1000. All 90 soil samples were submitted for 16S rRNA gene amplification and sequencing.

### PCR Amplification and Sequencing

Genomic DNA samples were sent to Argonne National Laboratory, where they underwent an amplicon sequencing protocol as previously described [19, 36]. Forward primer 515F (5-GTGCCAGCMGCCGCGGTAA-3) and reverse primer 806R (5-GGACTACHVGGGTWTCTAAT-3) were used to PCR amplify 253bp (not including primers) covering hyper variable region V4 of the 16S rRNA gene. Amplicons were sequenced with Illumina’s MiSeq, using 2x250bp reads (paired end sequencing).

### Sequence Analysis

Sequencing reads were demultiplexed, then poor-quality regions were filtered/trimmed with Trimmomatic (version 0.32) and discarded from further analysis. Raw reads were deposited in Zenodo [37]. Sequences were processed in mothur (version 1.32.1), following a custom protocol based on the Schloss lab MiSeq SOP [38]. In summary, we built contigs from paired end sequences, aligned contigs to a SILVA-based reference alignment [39], taxonomically classified contigs using the GreenGenes database [40], removed chimeras (using the UCHIME algorithm), and clustered the sequences into operational taxonomic units (OTUs) at 97% sequence similarity. The taxonomic classification was imported and displayed in an interactive hierarchical phylogenetic plot for each of the samples using Krona [41].

Various statistical tests were performed using mothur after the sequences were clustered into OTUs. Richness and diversity were calculated using the Chao algorithm and Shannon index, respectively. In order to ascertain the statistical differences between samples, first a pairwise comparison of samples was generated using the Yue and Clayton theta calculator, as implemented in mothur. Both 2-dimensional Principle Coordinates Analysis (PCoA) and 2-dimensional Non-metric multidimensional scaling (NMDS) were performed on the distance matrix. Additionally, Analysis of Molecular Variance (AMOVA), which calculates differences between and among genetic populations, were performed comparing samples based on site (INT, TRN, or ORG), sample type (coffee rhizosphere or control), and the various combinations of site and sample type. Finally, LEfSe [42] was used to measure statistical differences of OTUs in the coffee rhizosphere between each of the sites.

## Results

### DNA sequences

Of the 90 genomic DNA samples sequenced, 81 samples (90%) contained sufficient sequence data for further analysis, with read pairs median of 58,339, ranging from 21,959 to 92,883. All 20 samples for each of the rhizosphere from the intensive (INT-Coffee), transitional (TRN-Coffee), and organic (ORG-Coffee) farms contained sufficient sequence data. Among the soil control samples, out of the 10 samples collected from each site, sufficient sequence data were retrieved from nine of the intensive (INT-Control), 10 from the transitional (TRN-Control), and 2 from the organic (ORG-Control) farm. Most samples with low sequence count were from ORG-Control (eight out 10), which also had low DNA yield, perhaps due to low microbial load in those soils. After quality filtering, the eight ORG-Control samples with low sequence counts were discarded and the 81 remaining samples had a median size of 47,715 read pairs (range 18,320–72,205).

### Taxonomy

At the broad taxonomic level, we identified sequences from 48 bacterial phyla and 3 archaeal phyla (S2 Table) among all samples. In addition, 1.3% (34822) of the Bacteria sequences and 0.9% (47) of the Archaea sequences were determined unclassified. Bacteria dominated the community with 98% of the sequences compared to 2% Archaea. On average, Proteobacteria was the most prevalent phylum at each sample site, with Acidobacteria and Actinobacteria being either the second or third most prevalent, except for the TRN-Control site, where Acidobacteria was the most prevalent, followed by Proteobacteria and Verrucomicrobia (Table 2). The TRN-Control site had grass covering the soil, whereas the other control sites (INT-Control and ORG-Control) were bare soil (no vegetation coverage). Within Archaea, phylum Thaumarchaeota [43, 44] represented 89.7% of the sequences retrieved, with the remaining sequences clustered within phylum Euryarchaeota (8.3%), phylum Parvarchaeota (1.9%), or unclassified Archaea (~0.1%). Recently, Thaumarchaeota was reclassified from an order to a phylum [45] in the Archaea domain. Greengenes has not yet updated its classification and Thaumarchaeota remains an order in that database. Hence, we classified all our sequences using the Ribosomal Database Project (RDP) classifier, version September 2014 [46]. The same number of sequences previously classified in the order Thaumarchaeota in Greengenes was classified as phylum Thaumarchaeota by RDP. To agree to a more updated classification we will refer to our order Thaumarchaeota sequences as phylum Thaumarchaeota. Euryarchaeota is a poorly described group in soil [47] and Parvarchaeota is a recently proposed environmental group with no pure isolates, having been found in low abundance in acidic environments [48]. Taxonomic groups can be visualized and be individually interrogated for relative abundance and number of sequences identified in Krona plots (S1 Fig).

**Table 2:** Most prevalent phyla.

Sample Site	1^st^	2^nd^	3^rd^
**INT-Coffee**	Proteobacteria (32.7%)	Acidobacteria (16.5%)	Actinobacteria (12.5%)
**INT-Control**	Proteobacteria (37.4%)	Actinobacteria (18.7%)	Acidobacteria (10.7%)
**TRN-Coffee**	Proteobacteria (29.7%)	Acidobacteria (20.5%)	Actinobacteria (10.7%)
**TRN-Control**	Acidobacteria (29.7%)	Proteobacteria (18.7%)	Verrucomicrobia (15.7%)
**ORG-Coffee**	Proteobacteria (32.8%)	Actinobacteria (20.1%)	Acidobacteria (17.0%)
**ORG-Control**	Proteobacteria (35.7%)	Acidobacteria (15.6%)	Actinobacteria (9.7%)

Taxonomic classification raking the 1^st^, 2^nd^, and 3^rd^ most prevalent phylum in each of the six sample sites. Data were classified using mothur’s classify.seqs command and GreenGenes as the reference database (May 2013). In parentheses is the relative abundance of each phylum as a percentage of the total number of sequences within each site.

### OTUs, Richness, and Diversity

For comparison, we normalized sequences in each sample to 3,000 random sequences for a total of 243,000 sequences (81 samples x 3,000 sequences per sample), where 45,545 (18.7%) were identified at least once (referred here as unique sequences). These unique sequences were clustered into OTUs, resulting in 12,650 OTUs at the 97% similarity level, 7,273 OTUs at the 95% and 2,505 OTUs at the 90% similarity level. For clarity all other analyses and statistics in this study used OTUs clustered at the 97% similarity level.

The highest number of OTUs at 97% sequence similarity was found in the rhizosphere samples of each farm as compared to the control soil (Table 3). And among the rhizospheres, the transitional farm had the highest number of OTUs (6,133) followed by the intensive farm (5,661) and lastly the organic farm (4,911). Number of OTUs per sample site was, as expected, directly correlated to the number of samples analyzed. Twice as many OTUs were identified in the rhizosphere (twice as many samples) as in the control soil samples for INT and TRN with the lowest number of OTUs identified in the ORG-Control site, where only two out of 10 samples had high quality sequences as detailed above.

**Table 3:** Summary data of OTUs, richness, and diversity.

Sample	Total OTUs	Per Sample OTUs (Min-Max [Median])	Chaos Richness [95% CI^a^]	Shannon Diversity [95% CI]
INT-Coffee	5661	769–1068 [923]	9673 [9270–10122]	6.90 [6.88–6.91]
INT-Control	2779	114–1019 [880]	4515 [4266–4804]	6.27 [6.25–6.29]
TRN-Coffee	6133	719–1089 [947.5]	10929 [10461–11448]	6.94 [6.93–6.95]
TRN-Control	2987	612–819 [705.5]	5263 [4964–5608]	5.73 [5.70–5.75]
ORG-Coffee	4911	119–1050 [823]	7778 [7463–8133]	6.64 [6.63–6.65]
ORG-Control	1011	98–955 [526.5]	1901 [1715–2138]	5.55 [5.50–5.59]

Values shown for each of the six sites (INT, TRN, or ORG) and each sample type (coffee or control) combinations, calculated using mothur. OTUs were clustered at 97% sequence similarity.

^a^ CI = confidence interval

**Fig 1:**
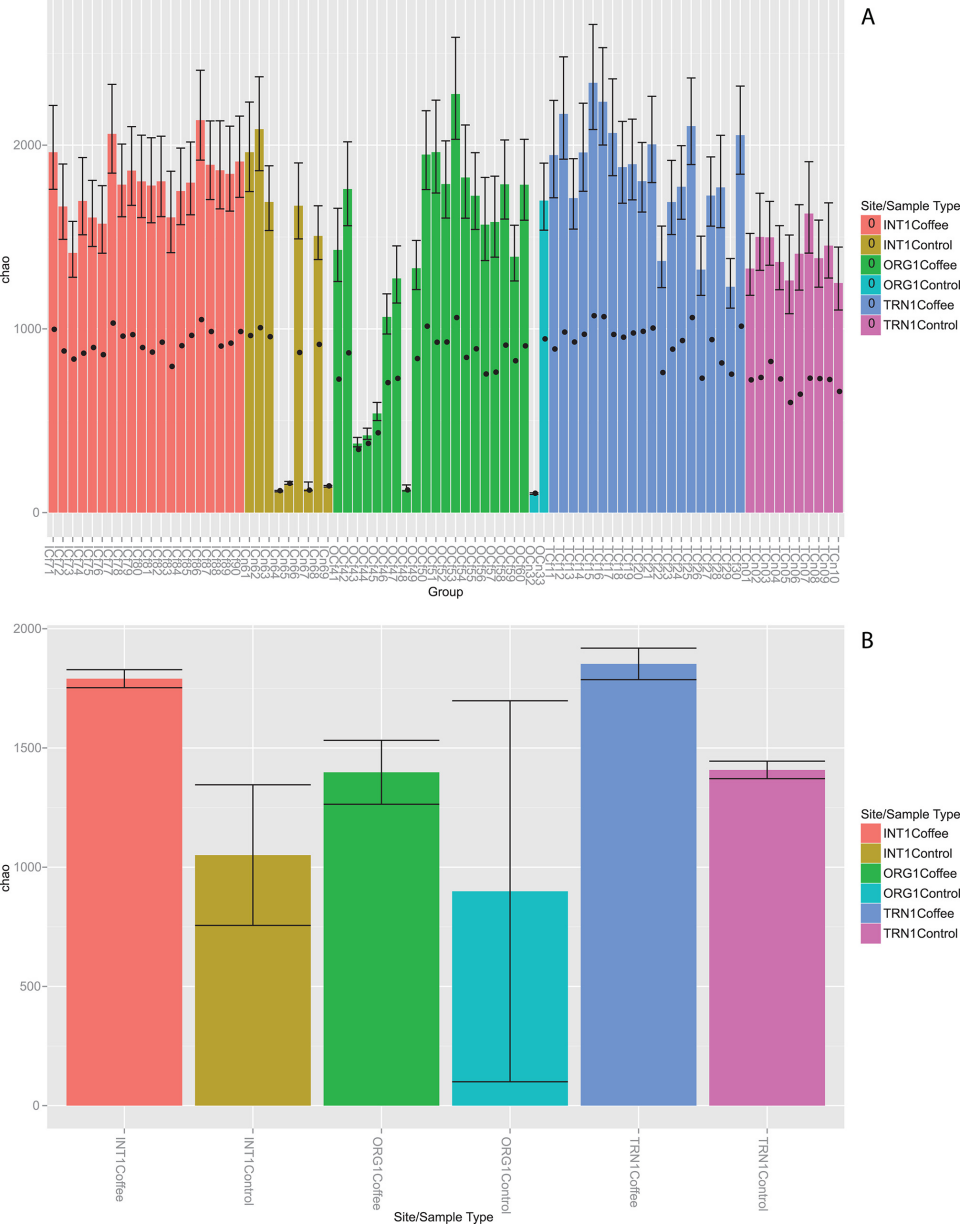
Chao richness estimates of species-level OTUs. **(A)** Richness level calculated for each of 81 samples, color-coded and grouped by site (INT, TRN, or ORG) and sample type (coffee rhizosphere or soil control) as detailed in the methods. Richness estimates (± 95% confidence intervals) of 97% similarity OTUs calculated in mothur and plotted in R. Black circle indicates number of observed OTUs. **(B)** Mean Chao richness estimates (± standard error) from individual samples shown in Fig 1A within each sample site and sample type calculated in R.

Chao algorithm used to estimate richness (number of unique taxa) and Shannon index to estimate diversity, suggest the coffee rhizosphere samples (INT-Coffee, TRN-Coffee, and ORG-Coffee) had higher richness and diversity compared to their respective control samples (Table 3). Among the rhizosphere samples, the transitional farm (TRN-Coffee) had the highest richness, followed by INT-Coffee, then ORG-Coffee, with no overlapping 95% confidence intervals. Richness estimates of individual samples (Fig 1A) had high variability in the INT-Control, ORG-Coffee, and ORG-Control sites compared to the low variability observed in the INT-Coffee, TRN-Coffee, and TRN-Control sites. It is unclear what regulates richness in these habitats. Even though Chao richness values of some control samples were within the confidence intervals (CI) of the respective rhizosphere sample site (Fig 1A), the mean Chao richness value in each of the three rhizosphere sites was higher as compared to the respective control site (Fig 1B). Interestingly, the TRN-Control samples, which were collected in soil covered with grass, showed higher richness level as compared to the other two controls (INT and ORG), suggesting a unique and diverse microbiome is associated with the grass (Table 3).

### Statistics

AMOVA statistical analysis (based on OTUs) showed that each site (INT, TRN, ORG) was statistically different from the other (p <0.0001), when rhizosphere and control samples were combined per site (INT-ORG df = 50, INT-TRN df = 58, ORG-TRN df = 51). Similarly, combined coffee rhizosphere samples were statistically significantly different from combined control samples (p<0.0001, df = 80). Comparisons between the rhizosphere versus control samples within each respective sample site were also significantly different (p < 0.05) for the intensive (INT-Coffee x INT-Control) and transitional (TRN-Coffee x TRN-Control) samples, but not for the organic farm (ORG-Coffee x ORG-Control), most likely due to the low sample size (n = 2) of the control (ORG-Control), suggesting further sampling is needed for appropriate comparisons. All comparisons between coffee rhizospheres were highly significant: INT-Coffee x ORG-Coffee p<0.0001, df = 39; INT-Coffee x TRN-Coffee p = 0.0002, df = 39; and ORG-Coffee x TRN-Coffee p<0.0001, df = 39.

**Fig 2:**
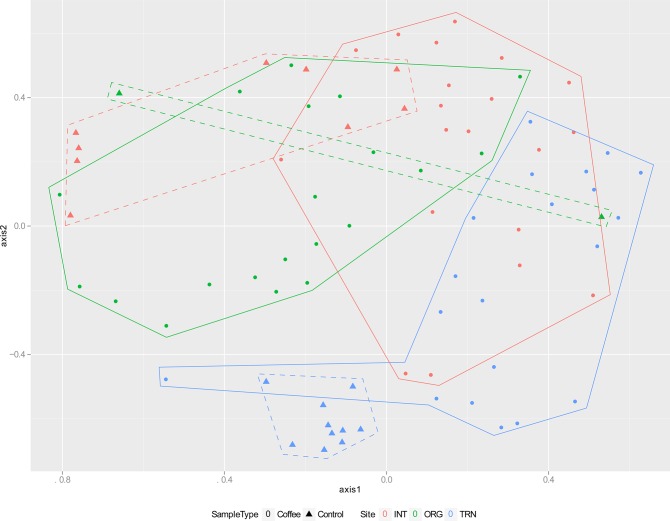
NMDS of species-level OTUs, grouped by site and sample type. NMDS plot, analyzing differences in 97% similarity OTU taxonomy between samples. NMDS axes calculated in mothur, results plotted in R. Number of random configurations tested = 1000, with 10000 iterations of each configuration. R^2^ = 0.711. Lines indicate clustering of samples in each site-sample type combination. Sites were INT, TRN, and ORG whereas sample types included either coffee rhizosphere or soil control.

Comparable results were obtained from both 2-dimensional PCoA (data not shown) (R^2^ = 0.710) and 2-dimensional NMDS (R^2^ = 0.711, lowest stress = 0.289) (Fig 2). Those results largely agreed with the AMOVA results, where although overlap exists between sites, each site distribution takes a slightly different shape. On the sample level, it is interesting to note that individual samples from one site are in some cases found closer to samples from a different site than they are to the nearest sample from the same site. The relatively high inter-sample distances indicate coffee rhizobacteria are likely influenced by more than just the type of cultivation. The OTUs in the controls formed different groupings than their respective rhizosphere samples, despite control samples being collected near the coffee plants. While the control samples for the intensive farm and the transitional farm slightly overlap their respective coffee rhizosphere samples, the organic farm’s controls do not. The samples taken from the transitional control grass rhizosphere (TRN-Control) also show a much tighter grouping than those of the coffee rhizosphere, underscoring the noted heterogeneity of the coffee rhizosphere samples.

**Fig 3:**
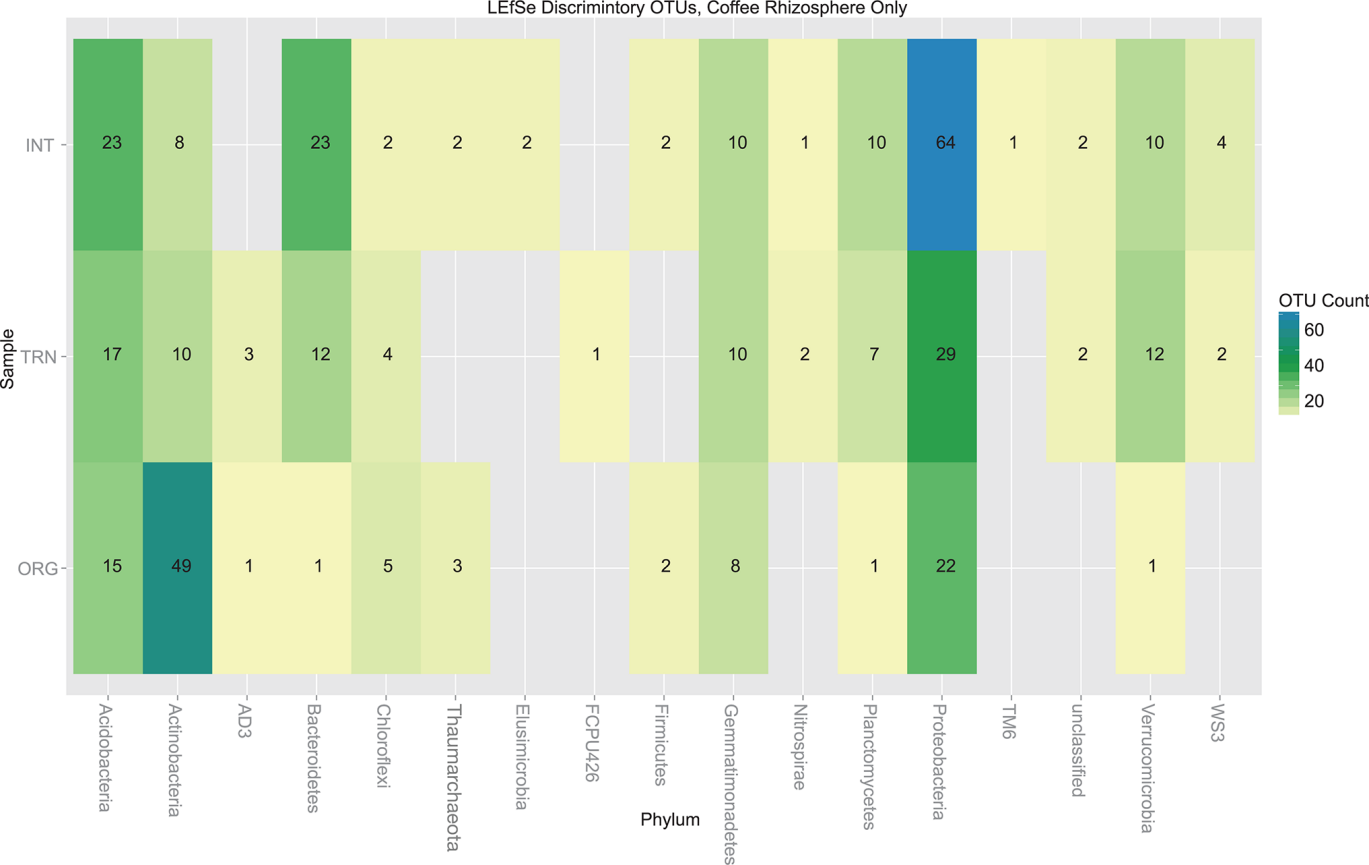
LEfSe discriminatory OTUs among coffee rhizosphere samples. Heatmap of LEfSe results. The number of discriminatory OTUs in each phylum is shown in corresponding box for each of the three sample sites (INT, TRN, and ORG). Statistic calculated in mothur and results plotted in R.

In order to determine which OTUs were statistically significantly different in abundance between sites, we used mothur’s implementation of the LEfSe algorithm. We focused only on the coffee rhizosphere samples for this analysis. While the bulk of the 12,650 OTUs were not found to vary significantly among farms, 385 OTUs (3%) were identified to be discriminatory among the three sites (Fig 3), all of which had a linear discriminant analysis (LDA) score >2, the threshold for statistical significance. The phyla with the most discriminatory OTUs mirrored the most abundant phyla for the overall project, where Proteobacteria had 115 OTUs, Actinobacteria 67 OTUs, and Acidobacteria 55 OTUs. Together, these three phyla accounted for over 60% of the 385 OTUs identified with significant differences in abundance between samples. A similar trend can be seen in several phyla. In Bacteroidetes, Proteobacteria, and Acidobacteria the most discriminatory OTUs were in the intensive farming site, followed by the transitional, followed by the organic. The opposite trend was observed for Actinobacteria, where nearly 5 times more abundant OTUs were found in the organic farm as compared to either transitional or intensive farms.

Two distinct groups of prokaryotes involved in the nitrogen cycle, *Candidatus* Nitrososphaera (an ammonia oxidizing Archaea [AOA] in the phylum Thaumarchaeota) and Rhizobiales bacteria, were identified among the discriminatory OTUs. Two discriminatory *Ca*. Nitrososphaera OTUs were found in the intensive farm and 3 in the organic farm. *Ca*. Nitrososphaera accounted for 1.7% of overall sequences and 88% of the observed Archaea. The highest relative abundance of *Ca*. Nitrososphaera sequences was found in the INT farm, with 2.18% of the total sequences, followed by the TRN farm (1.74%) and the ORG farm (1.52%). Order Rhizobiales of the Proteobacteria, which accounted for 6.7% of overall sequences, also had many discriminatory OTUs (6 in INT, 6 in ORG, and 5 in TRN). The trend in abundances of Rhizobiales was the inverse of *Ca*. Nitrososphaera abundances: the highest amounts were seen in the ORG farm (8.83%) followed by TRN (5.77%) and INT (5.62%). While not all Rhizobiales are considered ammonia-oxidizing bacteria (AOB), many are involved in nitrogen fixation when associated with leguminous plants, and some members are also plant or animal pathogens [49]. Three families within the order Rhizobiales were found in larger proportions than most other identified bacteria in the rhizosphere samples of each farm included Bradyrhizobiaceae (0.68% INT; 1.89% ORG; and 0.84%TRN), Rhizobiaceae (0.41% INT; 0.30% ORG; and 0.14%TRN), and Rhodobiaceae (0.12% INT; 0.03% ORG; and 0.84%TRN) (S2 Fig). Numerous other groups known to support plant growth via the nitrogen cycle were present, but at a minor relative abundance and at no significant difference among the three farms

Three common Bacteria genera involved in nitrogen fixation are *Acetobacter* (family Acetobacteraceae), *Azospirillum* (family Rhodospirillaceae), and *Azotobacter* (family Pseudomonadaceae). The family Acetabacteraceaea was proportionally larger in the organic farm rhizosphere (S2 Fig), but only 3 *Acetobacter* sequences were detected, all in the intensive farm rhizosphere samples. *Azospirillum* had 1 sequence in INT-Coffee, 4 in TRN-Coffee and 0 in ORG-Coffee samples. Even though 19,971 sequences were detected in the Pseudomonadaceae family, none were classified in the genus *Azotobacter*. Instead, genus *Pseudomonas* represented 82% (16,370 sequences) of the Pseudomonadaceae family. *Pseudomonas* sp. are known to act as biocontrol-PGPB by inhibiting plant soil-borne pathogens [50], but except for the transient farm, the *Pseudomonas* sp. sequences were mostly in the control soil of each respective farm (0.5% of INT-Coffee and 2.35% of the INT-Control; 0.43% ORG-Coffee and 5.77% of ORG-Control; and 0.11% TRN-Coffee and 0.02% TRN-Control).

Among the three PGPB associated with Robusta coffee, *Achromobacter*, *Stenotrophomonas*, and *Leifsonia*, only members of the latter were not detected in our samples. The relative abundance of *Achromobacter* sp. was 0.03% for all sequences, but 60% of the sequences (538) was found in the organic farm rhizosphere. Only a total of 682 sequences of *Stenotrophomonas* sp. (0.02% of all sequences) were detected, representing 0.03% of the INT-Coffee, 0.02% of the ORG-Coffee, and 0.01% of the TRN-Coffee total sequences.

## Discussion

Even though the three farms included in this study were located in close proximity (within a 5.5 km radius), they had the same type of soil classification (latosols) and grew the same species of coffee plants (*Coffea arabica* L., variety Red Catuai) the microbiomes identified among them differed drastically. Some of the major distinct characteristics among these farms were the farming practices and the dispersal of the coffee plants (distance among the trees), which have a direct effect on the soil chemistry and the microbes associated with the rhizosphere. As with other soil microbiomes, the rhizospheric soil associated with coffee plants contained a highly diverse group of prokaryotes. Thousands of species-level OTUs were identified in each farm when aggregating results from their respective 20 individual 0.25g soil samples. Despite similarities in coarse-grained taxonomy, with Proteobacteria, Acidobacteria, and Actinobacteria making up over half of the bacterial species identified in each of the sites, each farm had a distinct phylogenetic signature. Statistically significant results were found both when comparing the three farms against each other as a whole, as well as when comparing individual OTU abundances between the farms. Additionally, the soil prokaryotes associated with the coffee plant rhizosphere were more diverse and were compositionally different from their respective control soil samples. Interestingly, when comparing differences in diversity between coffee rhizosphere samples and their respective controls, the smallest increase in diversity was observed between the intensive farm’s two sample types (INT-Coffee x INT-Control), perhaps due to the transitional site having grass growing on its control (enhancing diversity), and the organic site’s control having a small sample size. Nonetheless, the control samples allowed us to remove some of the “noise” in the complex rhizosphere microbiome, making it possible to establish more discrete portions of the community associated with the coffee rhizosphere. While the data suggest coffee plants growing in different farms have different rhizospheric populations, it is left to further studies whether these changes are due directly to the farming practices or other confounding factors such as soil chemistry.

To our knowledge this is the first investigation of the microbial diversity in coffee rhizosphere using deep sequencing. Compared to other studies using next-generation sequencing targeting the bacterial community in soil of the eastern Brazil’s savanna-like *cerrado* biome and in Brazilian soil of sugarcane crops [51–53] the predominant phyla were also Proteobacteria, Acidobacteria, and Actinobacteria, indicating these groups are not only common but may play an important role in diverse Brazilian crop soils.

It has been known that PGPB in the rhizosphere can affect plants directly by promoting plant growth or indirectly by either inhibiting plant pathogens or increasing plant resistance to pathogens (Biocontrol-PGPB) [50, 54, 55]. Agriculture has greatly benefitted from this knowledge and PGPB inoculants have been used extensively to increase farm productivity [56]. Classic examples of PGPB that benefit plants by fixing nitrogen in the genera *Rhizobium*, *Agrobacterium*, *Acetobacter* and *Azospirillum* were detected in fairly low relative abundance in our samples, even though the families in which these microbes belong to had some of the highest relative abundances (# of sequences in the family divided by all sequences in each sampling site) compared to all other families in our database. This suggests new prominent beneficial microbes for coffee plants can be discovered within those families. For instance, the genus *Acetobacter*, which includes the species *A*. *diazotrophicus*, a model PGPB organism associated with coffee and sugarcane crops in Brazil [57], was only detected in the intensive coffee rhizosphere in our samples (3 sequences total); whereas we classified 8,309 sequences in the family Acetobacteraceae, half of which were in the organic coffee rhizosphere.

By contrast, two large groups of ammonia oxidizing prokaryotes were discovered in this study, *Ca*. Nitrososphaera (1.7% of total sequences) and Rhizobiales (6.7%), serving as potential new avenues of further exploration. The trend of increasing abundances of *Ca*. Nitrososphaera from ORG < TRN < INT is worthy of future study, since the difference is statistically significant, and all known members of the genus are associated with the nitrogen cycle. While the trend is reversed in Rhizobiales, a targeted study with a longer 16S rRNA gene amplicon would be needed to elucidate which genera are present in order to hypothesize whether the organisms present play a role in nitrogen fixation or plant pathogenicity. Coffee plants have high nitrogen requirements for proper berry development [58] and some plants have been shown to establish infections from multiple nitrogen-fixing bacteria genera [59]. Since the nitrogen cycle and nitrogen availability play a significant role in overall production levels in coffee and other crops, it would be prudent to continue studying these groups and how they may relate to nitrogen availability and yield of coffee.

The goal of this project was to differentiate the microbial communities associated with the rhizosphere of coffee plants between organic and pesticide-treated coffee farms using next generation sequencing. Our initial hypothesis, that microbial diversity is reduced in pesticide-treated farms as compared to organic farms, was not supported. In fact, the organic farm showed the lowest levels of observed OTUs, richness, and diversity. There are many unexplored differences between the farms, but it is possible the combination of soil conditions and coffee plant density in the organic farm were inappropriate to sustain a more diverse microbial community or the fertilization and other chemical amendments applied in the intensive farm had a diversity promoting effect. Soil chemical composition alone cannot explain the lower OTU numbers observed in the organic farm, since except for sodium and phosphorous, levels of nutrients and organic matter in the organic farm rhizosphere were either similar or higher than those in the other two farms.

Reports show that yields in organic coffee farming are lower than in intensive farming [60] by as much as 20% over a three year average [27]. One long-term goal application of our study is to investigate potential microbes with a positive effect in coffee production. Manipulation of the microbial composition in soil to enrich for such microbes and stimulate plant growth, as shown in other types of farming, could be applied in coffee production to provide an alternative to chemical treatment and potentially reduce the use of industrial fertilizer, herbicides and pesticides [50] or in bioremediation to remove contaminants from previous farming [20] in the transition to a more organic practice. For example, part of the increase in soybean production in Brazil is due to soil inoculation with PGPB strains [61]. We propose that *Ca*. Nitrososphaera and Rhizobiales are two contenders for such future manipulation studies in coffee farms. A recent study reported a higher abundance of ammonia-oxidizing Archaea (AOA) *Ca*. Nitrososphaera in agriculture soils for wheat, corn, and sugarcane crops as compared to non-agricultural controls [62]. In agreement to this study, Zhalnina et al. (2013) reported *Ca*. Nitrososphaera to be positively correlated to agricultural management and inversely correlated to the plant diazotrophic symbiont, *Bradyzhizobium*.

The higher cost of growing organic coffee compared to conventional farming has led many farmers to look for intensification of production to remain competitive, especially during times of low prices in the coffee market [27]. Continuation of this study to a larger sample size can provide insight into increasing yields without the cost of environmental damage.

## Supporting Information

S1 Table Soil Chemical Analysis. One chemical analysis was performed per sampling site area for a total of four analyses. Soil sample collection followed the guidelines provided by the laboratory conducting the analysis. Measurements of Zn, Fe, Mn, Cu B and S were not performed for the organic farm soils.
(XLSX)

S2 Table Phylum level taxonomy summary. Taxonomic classifications done in mothur, using GreenGenes database (May 2013).
(XLSX)

S1 Fig Interactive phylogeny. Online phylogenetic display of 16S rRNA sequences for each of the 81 samples from the Intensive farm rhizosphere (ICf 71–90) and control (ICn 61–69), the Transient farm rhizosphere (TCf 11–30) and control (TCn 1–10), and the Organic farm rhizosphere (OCf 41–60) and control (OCn 32–33). A radial phylogeny rooted at the center is automatically displayed once a sample is selected from the list of samples on the left. The percentage values represent the relative abundance of the sequences clustering in that phylogenetic group. The hierarchy of each section can be further interrogated by either double clicking a section or by selecting a section and clicking the right or left arrows on the top left corner of the page (right arrow moves toward the root of the chart). Specific terms can be searched and quantified. Figures were generated using Krona [35], based on mothur’s taxonomic classifications.
(HTML)

S2 Fig Quantification of common families of nitrogen fixation bacteria. Relative abundance of nitrogen-fixing archaeal and bacterial family-level groups detected in the rhizosphere of each of the three farms (INT, ORG, and TRN) or all sites together (% of the Total). The family-level relative abundance was calculated as the percentage of sequences of each family found in the total number of sequences retrieved from each rhizosphere. The % of the Total was the number of family-level sequences divided by the total number of sequences from all sites combined. Even though a low number of sequences were retrieved from the genus level of the nitrogen-fixing bacteria, the relative abundance of the respective families were some of the highest among all families in the study.
Raw 16S rRNA sequence data have been deposited at Zenodo: http://dx.doi.org/10.5281/zenodo.11120
The full mothur protocol and additional data analysis scripts can be downloaded from Zenodo: http://dx.doi.org/10.5281/zenodo.11126
(PNG)

## References

[1] UNCTAD. Market: United Nations Conference on Trade and Development; 2012 [updated 4/23/2012; cited 2014 02/14]. Available from: http://www.unctad.info/en/Infocomm/Beverages/Coffee-French-version-only/Market/.

[2] Geiger F, Bengtsson J, Berendse F, Weisser WW, Emmerson M, Morales MB, et al Persistent negative effects of pesticides on biodiversity and biological control potential on European farmland. Basic Appl Ecol. 2010;11(2):97–105. 10.1016/J.Baae.2009.12.001 .

[3] Lewis SE, Brodie JE, Bainbridge ZT, Rohde KW, Davis AM, Masters BL, et al Herbicides: A new threat to the Great Barrier Reef. Environ Pollut. 2009;157(8–9):2470–84. 10.1016/J.Envpol.2009.03.006 . 19349104

[4] Mann RM, Hyne RV, Choung CB, Wilson SP. Amphibians and agricultural chemicals: Review of the risks in a complex environment. Environ Pollut. 2009;157(11):2903–27. 10.1016/J.Envpol.2009.05.015 . 19500891

[5] Butt MS, Sultan MT. Coffee and its Consumption: Benefits and Risks. Crit Rev Food Sci. 2011;51(4):363–73. 10.1080/10408390903586412 . 21432699

[6] Carvalho DD, Brigagão MRPL, dos Santos MH, de Paula FBA, Giusti-Paiva A, Azevedo L. Organic and Conventional Coffea arabica L.: A Comparative Study of the Chemical Composition and Physiological, Biochemical and Toxicological Effects in Wistar Rats. Plant Food Hum Nutr. 2011;66(2):114–21. 10.1007/S11130-011-0221-9 . 21523414

[7] Higdon JV, Frei B. Coffee and health: A review of recent human research. Crit Rev Food Sci. 2006;46(2):101–23. 10.1080/10408390500400009 . 16507475

[8] Jimenez-Salgado T, Fuentes-Ramirez LE, Tapia-Hernandez A, Mascarua-Esparza MA, Martinez-Romero E, Caballero-Mellado J. Coffea arabica L., a new host plant for Acetobacter diazotrophicus, and isolation of other nitrogen-fixing acetobacteria. Appl Environ Microb. 1997;63(9):3676–83. . 10.1128/aem.63.9.3676-3683.1997 PMC168673 9293018

[9] Hoppin JA, Valcin M, Henneberger PK, Kullman GJ, Umbach DM, London SJ, et al Pesticide use and chronic bronchitis among farmers in the agricultural health study. Am J Ind Med. 2007;50(12):969–79. 10.1002/Ajim.20523 . 17975796 PMC2806052

[10] Tual S, Clin B, Levêque-Morlais N, Raherison C, Baldi I, Lebailly P. Agricultural exposures and chronic bronchitis: findings from the AGRICAN (AGRIculture and CANcer) cohort. Ann Epidemiol. 2013;23(9):539–45. 10.1016/J.Annepidem.2013.06.005 . 23886973

[11] Abou El Azm AR, Yousef M, Mansour N, Awad A, El Dardiry S, Abdel Aziz I. New Insights on Non-B non-C Hepatocellular Carcinoma in Mid Delta Region, Egypt. Journal of gastrointestinal cancer. 2014. Epub 2014/02/04. 10.1007/s12029-013-9573-8 . 24488435

[12] dos Santos JS, dos Santos MLP, Conti MM. Comparative Study of Metal Contents in Brazilian Coffees Cultivated by Conventional and Organic Agriculture Applying Principal Component Analysis. J Brazil Chem Soc. 2010;21(8):1468–76. 10.1590/S0103-50532010000800009 .

[13] ICO. EXPORTING COUNTRIES: TOTAL PRODUCTION: International Coffee Organization; 2014 [updated January 2014; cited 2014 02/14]. Available: http://www.ico.org/prices/po.htm.

[14] Barros S. Brazil—Coffee Semi-annual In: Agriculture USDo, editor. Sao Paulo: USDA Foreign Agricultural Service; 2013.

[15] Curtis TP, Sloan WT, Scannell JW. Estimating prokaryotic diversity and its limits. P Natl Acad Sci USA. 2002;99(16):10494–9. 10.1073/Pnas.142680199 . PMC124953 12097644

[16] Yachi S, Loreau M. Biodiversity and ecosystem productivity in a fluctuating environment: The insurance hypothesis. P Natl Acad Sci USA. 1999;96(4):1463–8. 10.1073/Pnas.96.4.1463 . PMC15485 9990046

[17] Rappe MS, Giovannoni SJ. The uncultured microbial majority. Annu Rev Microbiol. 2003;57:369–94. 10.1146/Annurev.Micro.57.030502.090759 . 14527284

[18] Konstantinidis KT, Rossello-Mora R. Classifying the uncultivated microbial majority: A place for metagenomic data in the Candidatus proposal. Systematic and applied microbiology. 2015. Epub 2015/02/15. 10.1016/j.syapm.2015.01.001 . 25681255

[19] Caporaso JG, Lauber CL, Walters WA, Berg-Lyons D, Huntley J, Fierer N, et al Ultra-high-throughput microbial community analysis on the Illumina HiSeq and MiSeq platforms. Isme J. 2012;6(8):1621–4. 10.1038/Ismej.2012.8 . 22402401 PMC3400413

[20] de-Bashan LE, Hernandez JP, Bashan Y. The potential contribution of plant growth-promoting bacteria to reduce environmental degradation—A comprehensive evaluation. Applied Soil Ecology. 2012;61:171–89. 10.1016/J.Apsoil.2011.09.003 .

[21] Bhattacharya S, Bagyaraj DJ. Effectiveness of arbuscular mycorrhizal fungal isolates on arabica coffee (Coffea arabica L.). Biol Agric Hortic. 2002;20(2):125–31. 10.1080/01448765.2002.9754956 .

[22] Siqueira JO, Saggin-Júnior OJ, Flores-Aylas WW, Guimarães PTG. Arbuscular mycorrhizal inoculation and superphosphate application influence plant development and yield of coffee in Brazil. Mycorrhiza. 1998;7(6):293–300. 10.1007/S005720050195 .

[23] Muleta D, Assefa F, Börjesson E, Granhall U. Phosphate-solubilising rhizobacteria associated with Coffea arabica L. in natural coffee forests of southwestern Ethiopia. Journal of the Saudi Society of Agricultural Sciences. 2013;12(1):73–84. 10.1016/j.jssas.2012.07.002

[24] Wedhastri S, Yudianti NF, Widada J, Baon JB. Ability of Non Symbiotic Nitrogen-Fixing Bacteria Isolated from Coffee Plant Rhizosphere and Their Effects on Robusta Coffee Seedlings. Journal of Agricultural Science and Technology. 2012;A(2):660–6.

[25] Velmourougane K, Kumari D. P., Muralidhara H. R., & Prakasan C. B. Microbiology of coffee rhizosphere. Indian Coffee. 2006;70(5):10–3.

[26] Foresight. The Future of Food and Farming. The Government Office for Science, 2011 2011. Report No.

[27] Lyngbæk AE, Muschler RG, Sinclair FL. Productivity and profitability of multistrata organic versus conventional coffee farms in Costa Rica. Agroforest Syst. 2001;53(2):205–13. 10.1023/A:1013332722014 .

[28] Mondelaers K, Aertsens J, Van Huylenbroeck G. A meta-analysis of the differences in environmental impacts between organic and conventional farming. Brit Food J. 2009;111(10):1098–119. 10.1108/00070700910992925 .

[29] Noponen MRA, Edwards-Jones G, Haggar JP, Soto G, Attarzadeh N, Healey JR. Greenhouse gas emissions in coffee grown with differing input levels under conventional and organic management. Agr Ecosyst Environ. 2012;151:6–15. 10.1016/J.Agee.2012.01.019 .

[30] Blackman A, Naranjo MA. Does eco-certification have environmental benefits? Organic coffee in Costa Rica. Ecol Econ. 2012;83:58–66. 10.1016/j.ecolecon.2012.08.001

[31] Nogueira AM, Carvalho SPd, Batholo GF, Mendes ANG. Vegetative vigor and yield evaluations of coffee vultivars, "Catuai Vermelho" and "Amarelo" (*Coffea arabica* L.) planted isolated and different combinations. Ciência e Agrotecnologia. 2005;29(1):27–33.

[32] Smrke S, Kroslakova I, Gloess AN, Yeretzian C. Differentiation of degrees of ripeness of Catuai and Tipica green coffee by chromatographical and statistical techniques. Food chemistry. 2015;174:637–42. Epub 2014/12/23. 10.1016/j.foodchem.2014.11.060 . 25529730

[33] Silva MdC, Várzea V, Guerra-Guimarães L, Azinheira HG, Fernandez D, Petitot A-S, et al Coffee resistance to the main diseases: leaf rust and coffee berry disease. Brazilian Journal of Plant Physiology. 2006;18:119–47.

[34] Rodrigues WN, Tomaz MA, Apostólico MA, Colodetti TV, Martins LD, Christo LF, et al Severity of Leaf Rust and Brown Eyespot in Genotypes of Coffea arabica L. Cultivated with High Plant Density. American Journal of Plant Sciences. 2014;5:3702–9. 10.4236/ajps.2014.525386

[35] Vandermeer J, Jackson D, Perfecto I. Qualitative Dynamics of the Coffee Rust Epidemic: Educating Intuition with Theoretical Ecology. Bioscience. 2014;64(3):210–8. 10.1093/Biosci/Bit034 .

[36] Caporaso JG, Lauber CL, Walters WA, Berg-Lyons D, Lozupone CA, Turnbaugh PJ, et al Global patterns of 16S rRNA diversity at a depth of millions of sequences per sample. P Natl Acad Sci USA. 2011;108:4516–22. 10.1073/Pnas.1000080107 . PMC3063599 20534432

[37] Caldwell AC, Silva LCF, Silva CCd, Ouverney CC. 16S rRNA Sequence Data, Brazilian Coffee Soils. Zenodo. 2014.

[38] Kozich JJ, Westcott SL, Baxter NT, Highlander SK, Schloss PD. Development of a Dual-Index Sequencing Strategy and Curation Pipeline for Analyzing Amplicon Sequence Data on the MiSeq Illumina Sequencing Platform. Appl Environ Microb. 2013;79(17):5112–20. 10.1128/Aem.01043-13 . PMC3753973 23793624

[39] Quast C, Pruesse E, Yilmaz P, Gerken J, Schweer T, Yarza P, et al The SILVA ribosomal RNA gene database project: improved data processing and web-based tools. Nucleic Acids Res. 2013;41(D1):D590–D6. 10.1093/Nar/Gks1219 . 23193283 PMC3531112

[40] Second Genome I, Colorado Uo, Queensland Uo. Greengenes Database. In: Consortium TGD, editor. May 2013 ed2013.

[41] Ondov BD, Bergman NH, Phillippy AM. Interactive metagenomic visualization in a Web browser. Bmc Bioinformatics. 2011;12. doi: Artn 385 10.1186/1471-2105-12-385 . PMC3190407 21961884

[42] Segata N, Izard J, Waldron L, Gevers D, Miropolsky L, Garrett WS, et al Metagenomic biomarker discovery and explanation. Genome Biol. 2011;12(6). doi: Artn R60 10.1186/Gb-2011-12-6-R60 . PMC3218848 21702898

[43] Barns SM, Delwiche CF, Palmer JD, Pace NR. Perspectives on archaeal diversity, thermophily and monophyly from environmental rRNA sequences. P Natl Acad Sci USA. 1996;93(17):9188–93. 10.1073/Pnas.93.17.9188 . PMC38617 8799176

[44] Fuhrman JA, Mccallum K, Davis AA. Novel Major Archaebacterial Group from Marine Plankton. Nature. 1992;356(6365):148–9. . 1545865 10.1038/356148a0

[45] Brochier-Armanet C, Boussau B, Gribaldo S, Forterre P. Mesophilic Crenarchaeota: proposal for a third archaeal phylum, the Thaumarchaeota. Nature reviews Microbiology. 2008;6(3):245–52. Epub 2008/02/16. 10.1038/nrmicro1852 . 18274537

[46] Wang Q, Garrity GM, Tiedje JM, Cole JR. Naive Bayesian classifier for rapid assignment of rRNA sequences into the new bacterial taxonomy. Appl Environ Microbiol. 2007;73(16):5261–7. Epub 2007/06/26. 10.1128/AEM.00062-07 17586664 PMC1950982

[47] Hu HW, Zhang LM, Yuan CL, He JZ. Contrasting Euryarchaeota communities between upland and paddy soils exhibited similar pH-impacted biogeographic patterns. Soil Biol Biochem. 2013;64:18–27. 10.1016/J.Soilbio.2013.04.003 .

[48] Baker BJ, Comolli LR, Dick GJ, Hauser LJ, Hyatt D, Dill BD, et al Enigmatic, ultrasmall, uncultivated Archaea. P Natl Acad Sci USA. 2010;107(19):8806–11. 10.1073/Pnas.0914470107 . PMC2889320 20421484

[49] Carvalho FM, Souza RC, Barcellos FG, Hungria M, Vasconcelos ATR. Genomic and evolutionary comparisons of diazotrophic and pathogenic bacteria of the order Rhizobiales. Bmc Microbiol. 2010;10 10.1186/1471-2180-10-37 . PMC2907836 20144182

[50] Bashan Y. Inoculants of plant growth-promoting bacteria for use in agriculture. Biotechnol Adv. 1998;16(4):729–70. 10.1016/S0734-9750(98)00003-2 .

[51] Dini-Andreote F, Andreote FD, Costa R, Taketani RG, van Elsas JD, Araújo WL. Bacterial soil community in a Brazilian sugarcane field. Plant Soil. 2010;336(1–2):337–49. 10.1007/S11104-010-0486-Z .

[52] Quirino BF, Pappas GJ, Tagliaferro AC, Collevatti RG, Neto EL, da Silva MRSS, et al Molecular phylogenetic diversity of bacteria associated with soil of the savanna-like Cerrado vegetation. Microbiol Res. 2009;164(1):59–70. 10.1016/J.Micres.2006.12.001 . 17324564

[53] Rampelotto PH, Ferreira AD, Barboza ADM, Roesch LFW. Changes in Diversity, Abundance, and Structure of Soil Bacterial Communities in Brazilian Savanna Under Different Land Use Systems. Microb Ecol. 2013;66(3):593–607. 10.1007/S00248-013-0235-Y . 23624541

[54] van Loon LC. Plant responses to plant growth-promoting rhizobacteria. Eur J Plant Pathol. 2007;119(3):243–54. 10.1007/S10658-007-9165-1 .

[55] Yang J, Kloepper JW, Ryu CM. Rhizosphere bacteria help plants tolerate abiotic stress. Trends Plant Sci. 2009;14(1):1–4. 10.1016/J.Tplants.2008.10.004 . 19056309

[56] Rodriguez H, Fraga R, Gonzalez T, Bashan Y. Genetics of phosphate solubilization and its potential applications for improving plant growth-promoting bacteria. Plant Soil. 2006;287(1–2):15–21. 10.1007/S11104-006-9056-9 .

[57] Muthukumarasamy R, Revathi G, Loganathan P. Effect of inorganic N on the population, in vitro colonization and morphology of Acetobacter diazotrophicus (syn. Gluconacetobacter diazotrophicus). Plant Soil. 2002;243(1):91–102. 10.1023/A:1019963928947 .

[58] de Lima Filho OF, Malavolta E. Studies on mineral nutrition of the coffee plant (Coffea arabica L. cv. Catuai Vermelho). LXIV. Remobilization and re-utilization of nitrogen and potassium by normal and deficient plants. Brazilian journal of biology = Revista brasleira de biologia. 2003;63(3):481–90. Epub 2004/02/05. . 14758707 10.1590/s1519-69842003000300014

[59] Op den Camp RHM, Polone E, Fedorova E, Roelofsen W, Squartini A, Op den Camp HJM, et al Nonlegume Parasponia andersonii Deploys a Broad Rhizobium Host Range Strategy Resulting in Largely Variable Symbiotic Effectiveness. Mol Plant Microbe In. 2012;25(7):954–63. 10.1094/Mpmi-11-11-0304 . 22668002

[60] Valkila J. Fair Trade organic coffee production in Nicaragua—Sustainable development or a poverty trap? Ecol Econ. 2009;68(12):3018–25. 10.1016/J.Ecolecon.2009.07.002 .

[61] Hungria M, Vargas MAT. Environmental factors affecting N-2 fixation in grain legumes in the tropics, with an emphasis on Brazil. Field Crop Res. 2000;65(2–3):151–64. 10.1016/S0378-4290(99)00084-2 .

[62] Zhalnina K, de Quadros PD, Gano KA, Davis-Richardson A, Fagen JR, Brown CT, et al Ca. Nitrososphaera and Bradyrhizobium are inversely correlated and related to agricultural practices in long-term field experiments. Frontiers in microbiology. 2013;4:104 Epub 2013/05/04. 10.3389/fmicb.2013.00104 23641242 PMC3640186

