# Supplementary material for: Prokaryotic Diversity in the Rhizosphere of Organic, Intensive, and Transitional Coffee Farms in Brazil
Source: PLoS One. 2015 Jun 17;10(6):e0106355. doi: 10.1371/journal.pone.0106355 (PMC4471275; doi:10.1371/journal.pone.0106355)
Supplement: S1 Fig — Online phylogenetic display of 16S rRNA sequences for each of the 81 samples from the Intensive farm rhizosphere (ICf 71–90) and control (ICn 61–69), the Transient farm rhizosphere (TCf 11–30) and control (TCn 1–10), and the Organic farm rhizosphere (OCf 41–60) and control (OCn 32–33). A radial phylogeny rooted at the center is automatically displayed once a sample is selected from the list of samples on the left. The percentage values represent the relative abundance of the sequences clustering in that phylogenetic group. The hierarchy of each section can be further interrogated by either double clicking a section or by selecting a section and clicking the right or left arrows on the top left corner of the page (right arrow moves toward the root of the chart). Specific terms can be searched and quantified. Figures were generated using Krona [35], based on mothur’s taxonomic classifications. (HTML) [file pone.0106355.s003.html]

Javascript must be enabled to view this page.

xml version='1.0' encoding='ASCII'?


count

ICf71
ICf72
ICf73
ICf74
ICf75
ICf76
ICf77
ICf78
ICf79
ICf80
ICf81
ICf82
ICf83
ICf84
ICf85
ICf86
ICf87
ICf88
ICf89
ICf90
ICn61
ICn62
ICn63
ICn64
ICn65
ICn66
ICn67
ICn68
ICn69
OCf41
OCf42
OCf43
OCf44
OCf45
OCf46
OCf47
OCf48
OCf49
OCf50
OCf51
OCf52
OCf53
OCf54
OCf55
OCf56
OCf57
OCf58
OCf59
OCf60
OCn32
OCn33
TCf11
TCf12
TCf13
TCf14
TCf15
TCf16
TCf17
TCf18
TCf19
TCf20
TCf21
TCf22
TCf23
TCf24
TCf25
TCf26
TCf27
TCf28
TCf29
TCf30
TCn01
TCn02
TCn03
TCn04
TCn05
TCn06
TCn07
TCn08
TCn09
TCn10


35516
38137
39904
36746
35831
40282
36927
32793
34782
28776
34793
32212
35677
34942
31272
31091
40561
44623
37209
36859
35055
35192
34265
19017
17621
31438
18156
29232
41774
33053
32978
25876
27123
29966
26225
35803
17498
23984
30479
35099
30107
34362
30465
33399
35861
35681
33655
29644
39602
16591
35704
31851
38512
34705
42184
39377
35338
37312
43485
38746
52372
42763
31892
30405
36027
40010
32562
40644
39803
30937
33829
40022
48210
37552
22773
37666
28209
28571
38929
21010
22282


653
1507
1087
486
411
826
477
499
405
512
1241
765
807
1193
1391
850
782
1394
1188
995
1869
966
1177
0
20
995
34
487
1
713
602
213
96
797
366
142
1
377
709
237
306
561
788
555
1142
1783
784
231
785
59
979
575
374
1682
1276
923
559
819
1421
898
1873
1221
102
656
651
333
435
1018
356
263
558
368
252
154
71
288
160
221
108
81
86


540
1380
711
416
355
682
346
367
352
499
1235
754
789
926
1377
811
739
1325
1171
944
1819
948
1167
0
20
991
34
382
1
699
515
213
96
797
349
136
1
204
411
200
260
542
778
548
1130
1764
642
191
697
59
864
561
287
1398
1247
854
524
722
1282
810
1572
1093
92
487
594
286
271
945
144
241
277
353
244
149
65
281
154
196
100
81
71


1
0
0
3
0
4
1
0
0
0
0
0
0
0
0
0
0
0
0
0
0
0
0
0
0
0
0
0
0
0
0
0
0
0
0
0
0
0
0
0
0
1
0
5
1
2
0
0
0
0
1
25
37
0
0
0
5
2
1
1
0
0
0
0
17
2
22
0
28
14
6
3
20
3
7
13
1
16
26
2
3


1
0
0
3
0
4
1
0
0
0
0
0
0
0
0
0
0
0
0
0
0
0
0
0
0
0
0
0
0
0
0
0
0
0
0
0
0
0
0
0
0
1
0
5
1
2
0
0
0
0
1
22
36
0
0
0
5
2
1
1
0
0
0
0
17
2
22
0
28
14
6
3
20
3
7
13
1
16
26
2
3


1
0
0
3
0
4
1
0
0
0
0
0
0
0
0
0
0
0
0
0
0
0
0
0
0
0
0
0
0
0
0
0
0
0
0
0
0
0
0
0
0
1
0
5
1
2
0
0
0
0
1
22
36
0
0
0
5
2
1
1
0
0
0
0
17
2
22
0
28
14
6
3
20
3
7
13
1
16
26
2
3


1
0
0
3
0
4
1
0
0
0
0
0
0
0
0
0
0
0
0
0
0
0
0
0
0
0
0
0
0
0
0
0
0
0
0
0
0
0
0
0
0
1
0
5
1
2
0
0
0
0
1
22
36
0
0
0
5
2
1
1
0
0
0
0
17
2
22
0
28
14
6
3
20
3
7
13
1
16
26
2
3


1
0
0
3
0
4
1
0
0
0
0
0
0
0
0
0
0
0
0
0
0
0
0
0
0
0
0
0
0
0
0
0
0
0
0
0
0
0
0
0
0
1
0
5
1
2
0
0
0
0
1
22
36
0
0
0
5
2
1
1
0
0
0
0
17
2
22
0
28
14
6
3
20
3
7
13
1
16
26
2
3


0
0
0
0
0
0
0
0
0
0
0
0
0
0
0
0
0
0
0
0
0
0
0
0
0
0
0
0
0
0
0
0
0
0
0
0
0
0
0
0
0
0
0
0
0
0
0
0
0
0
0
3
1
0
0
0
0
0
0
0
0
0
0
0
0
0
0
0
0
0
0
0
0
0
0
0
0
0
0
0
0


0
0
0
0
0
0
0
0
0
0
0
0
0
0
0
0
0
0
0
0
0
0
0
0
0
0
0
0
0
0
0
0
0
0
0
0
0
0
0
0
0
0
0
0
0
0
0
0
0
0
0
3
1
0
0
0
0
0
0
0
0
0
0
0
0
0
0
0
0
0
0
0
0
0
0
0
0
0
0
0
0


0
0
0
0
0
0
0
0
0
0
0
0
0
0
0
0
0
0
0
0
0
0
0
0
0
0
0
0
0
0
0
0
0
0
0
0
0
0
0
0
0
0
0
0
0
0
0
0
0
0
0
3
1
0
0
0
0
0
0
0
0
0
0
0
0
0
0
0
0
0
0
0
0
0
0
0
0
0
0
0
0


0
0
0
0
0
0
0
0
0
0
0
0
0
0
0
0
0
0
0
0
0
0
0
0
0
0
0
0
0
0
0
0
0
0
0
0
0
0
0
0
0
0
0
0
0
0
0
0
0
0
0
3
1
0
0
0
0
0
0
0
0
0
0
0
0
0
0
0
0
0
0
0
0
0
0
0
0
0
0
0
0


539
1380
711
413
355
678
345
367
352
499
1235
754
789
926
1377
811
739
1325
1171
944
1819
948
1167
0
20
991
34
382
1
699
515
213
96
797
349
136
1
204
411
200
260
541
778
543
1129
1762
642
191
697
59
863
536
250
1398
1247
854
519
720
1281
809
1572
1093
92
487
577
284
249
945
116
227
271
350
223
146
58
268
153
180
74
79
68


0
0
0
16
0
11
0
0
1
0
0
1
0
1
0
0
1
0
0
0
0
0
0
0
0
0
0
0
0
0
0
0
0
0
0
0
0
0
0
0
0
1
0
0
1
0
0
0
0
0
0
0
14
0
0
0
0
0
0
0
0
0
25
0
227
4
116
0
5
2
0
0
0
0
1
0
0
0
0
0
0


0
0
0
16
0
11
0
0
1
0
0
1
0
1
0
0
0
0
0
0
0
0
0
0
0
0
0
0
0
0
0
0
0
0
0
0
0
0
0
0
0
1
0
0
1
0
0
0
0
0
0
0
12
0
0
0
0
0
0
0
0
0
25
0
227
4
102
0
5
2
0
0
0
0
1
0
0
0
0
0
0


0
0
0
16
0
11
0
0
1
0
0
1
0
1
0
0
0
0
0
0
0
0
0
0
0
0
0
0
0
0
0
0
0
0
0
0
0
0
0
0
0
1
0
0
1
0
0
0
0
0
0
0
12
0
0
0
0
0
0
0
0
0
25
0
227
4
102
0
5
2
0
0
0
0
1
0
0
0
0
0
0


0
0
0
16
0
11
0
0
1
0
0
1
0
1
0
0
0
0
0
0
0
0
0
0
0
0
0
0
0
0
0
0
0
0
0
0
0
0
0
0
0
1
0
0
1
0
0
0
0
0
0
0
12
0
0
0
0
0
0
0
0
0
25
0
227
4
102
0
5
2
0
0
0
0
1
0
0
0
0
0
0


0
0
0
0
0
0
0
0
0
0
0
0
0
0
0
0
1
0
0
0
0
0
0
0
0
0
0
0
0
0
0
0
0
0
0
0
0
0
0
0
0
0
0
0
0
0
0
0
0
0
0
0
2
0
0
0
0
0
0
0
0
0
0
0
0
0
14
0
0
0
0
0
0
0
0
0
0
0
0
0
0


0
0
0
0
0
0
0
0
0
0
0
0
0
0
0
0
1
0
0
0
0
0
0
0
0
0
0
0
0
0
0
0
0
0
0
0
0
0
0
0
0
0
0
0
0
0
0
0
0
0
0
0
2
0
0
0
0
0
0
0
0
0
0
0
0
0
14
0
0
0
0
0
0
0
0
0
0
0
0
0
0


0
0
0
0
0
0
0
0
0
0
0
0
0
0
0
0
1
0
0
0
0
0
0
0
0
0
0
0
0
0
0
0
0
0
0
0
0
0
0
0
0
0
0
0
0
0
0
0
0
0
0
0
2
0
0
0
0
0
0
0
0
0
0
0
0
0
14
0
0
0
0
0
0
0
0
0
0
0
0
0
0


539
1380
711
397
355
667
345
367
351
499
1235
753
789
925
1377
811
738
1325
1171
944
1819
948
1167
0
20
991
34
382
1
699
515
213
96
797
349
136
1
204
411
200
260
540
778
543
1128
1762
642
191
697
59
863
536
236
1398
1247
854
519
720
1281
809
1572
1093
67
487
350
280
133
945
111
225
271
350
223
146
57
268
153
180
74
79
68


539
1380
711
397
355
667
345
367
351
499
1235
753
789
925
1377
811
738
1325
1171
944
1819
948
1167
0
20
991
34
382
1
699
515
213
96
797
349
136
1
204
411
200
260
540
778
543
1128
1762
642
191
697
59
863
536
236
1398
1247
854
519
720
1281
809
1572
1093
67
487
350
280
133
945
111
225
271
350
223
146
57
268
153
180
74
79
68


539
1379
711
397
354
667
344
367
351
499
1234
753
789
925
1374
811
734
1320
1168
944
1815
947
1165
0
20
988
34
381
1
699
515
213
96
797
349
136
1
204
410
199
259
540
778
543
1127
1761
641
191
697
59
863
536
236
1397
1247
854
519
719
1281
807
1572
1093
67
484
350
280
133
945
111
225
271
350
223
146
57
268
153
180
74
79
65


66
114
46
143
46
134
30
75
19
66
51
65
33
40
184
84
89
407
144
96
419
208
250
0
0
187
0
112
0
187
128
181
0
501
76
42
0
8
59
49
37
111
117
100
240
236
155
34
122
0
47
201
74
114
18
59
84
101
45
81
138
62
26
38
124
58
30
91
18
75
38
315
154
104
34
223
97
116
64
48
40


33
318
162
46
132
78
111
32
90
93
800
349
514
456
476
269
340
436
593
258
600
213
367
0
0
436
0
82
0
69
43
29
67
53
3
43
0
28
65
75
48
90
56
52
50
42
181
17
114
0
74
187
48
160
61
244
95
165
132
106
138
91
23
61
32
44
2
95
8
24
58
15
37
15
8
18
17
19
7
17
7


374
715
370
107
154
410
158
215
212
319
301
259
179
269
525
302
222
277
339
480
509
413
404
0
20
280
21
108
1
387
303
3
0
223
239
28
0
159
231
43
125
265
533
301
655
1346
148
90
350
59
717
80
29
1003
1155
460
285
332
1063
501
1177
889
5
332
107
148
35
695
64
27
155
0
1
1
0
0
0
0
0
0
1


47
161
117
85
17
13
36
40
21
8
34
48
39
114
76
38
31
63
42
51
212
41
75
0
0
66
13
60
0
44
34
0
0
14
30
12
0
6
36
20
42
53
48
82
159
109
110
45
87
0
14
9
16
70
5
30
25
74
10
91
77
29
13
35
82
14
54
36
13
89
9
14
9
25
3
25
34
37
0
14
14


19
71
16
16
5
32
9
5
9
13
48
32
24
46
113
118
52
137
50
59
75
72
69
0
0
19
0
19
0
12
7
0
29
6
1
11
1
3
19
12
7
21
24
8
23
28
47
5
24
0
11
59
69
50
8
61
30
47
31
28
42
22
0
18
5
16
12
28
8
10
11
6
22
1
12
2
5
8
3
0
3


0
1
0
0
1
0
1
0
0
0
1
0
0
0
3
0
4
5
3
0
4
1
2
0
0
3
0
1
0
0
0
0
0
0
0
0
0
0
1
1
1
0
0
0
1
1
1
0
0
0
0
0
0
1
0
0
0
1
0
2
0
0
0
3
0
0
0
0
0
0
0
0
0
0
0
0
0
0
0
0
3


0
1
0
0
1
0
1
0
0
0
1
0
0
0
3
0
4
5
3
0
4
1
2
0
0
3
0
1
0
0
0
0
0
0
0
0
0
0
1
1
1
0
0
0
1
1
1
0
0
0
0
0
0
1
0
0
0
1
0
2
0
0
0
3
0
0
0
0
0
0
0
0
0
0
0
0
0
0
0
0
3


0
0
0
0
0
0
0
0
0
0
0
0
0
0
0
0
0
0
0
0
0
0
0
0
0
0
0
0
0
0
0
0
0
0
0
0
0
0
0
0
0
0
0
0
0
0
0
0
0
0
0
0
0
0
0
0
0
0
0
0
0
0
0
0
0
0
0
0
0
0
0
0
1
0
0
0
0
0
0
0
0


0
0
0
0
0
0
0
0
0
0
0
0
0
0
0
0
0
0
0
0
0
0
0
0
0
0
0
0
0
0
0
0
0
0
0
0
0
0
0
0
0
0
0
0
0
0
0
0
0
0
0
0
0
0
0
0
0
0
0
0
0
0
0
0
0
0
0
0
0
0
0
0
1
0
0
0
0
0
0
0
0


0
0
0
0
0
0
0
0
0
0
0
0
0
0
0
0
0
0
0
0
0
0
0
0
0
0
0
0
0
0
0
0
0
0
0
0
0
0
0
0
0
0
0
0
0
0
0
0
0
0
0
0
0
0
0
0
0
0
0
0
0
0
0
0
0
0
0
0
0
0
0
0
1
0
0
0
0
0
0
0
0


0
0
0
0
0
0
0
0
0
0
0
0
0
0
0
0
0
0
0
0
0
0
0
0
0
0
0
0
0
0
0
0
0
0
0
0
0
0
0
0
0
0
0
0
0
0
0
0
0
0
0
0
0
0
0
0
0
0
0
0
0
0
0
0
0
0
0
0
0
0
0
0
1
0
0
0
0
0
0
0
0


0
0
0
0
0
0
0
0
0
0
0
0
0
0
0
0
0
0
0
0
0
0
0
0
0
0
0
0
0
0
0
0
0
0
0
0
0
0
0
0
0
0
0
0
0
0
0
0
0
0
0
0
0
0
0
0
0
0
0
0
0
0
0
0
0
0
0
0
0
0
0
0
1
0
0
0
0
0
0
0
0


81
126
334
54
47
135
100
67
48
9
5
9
16
38
5
23
37
62
9
48
33
14
5
0
0
4
0
105
0
9
85
0
0
0
14
6
0
156
265
37
36
16
10
7
4
14
129
40
83
0
70
14
75
274
16
49
28
91
135
83
290
84
4
153
14
14
138
46
194
9
277
3
2
0
0
7
1
7
1
0
11


0
0
0
0
0
0
0
0
0
0
0
0
0
0
0
0
0
0
0
0
0
0
0
0
0
0
0
0
0
0
0
0
0
0
0
0
0
0
0
0
0
0
0
0
0
0
0
0
0
0
0
0
0
0
0
0
0
0
0
0
0
0
0
1
0
0
0
0
4
0
0
0
0
0
0
0
0
0
0
0
0


0
0
0
0
0
0
0
0
0
0
0
0
0
0
0
0
0
0
0
0
0
0
0
0
0
0
0
0
0
0
0
0
0
0
0
0
0
0
0
0
0
0
0
0
0
0
0
0
0
0
0
0
0
0
0
0
0
0
0
0
0
0
0
0
0
0
0
0
4
0
0
0
0
0
0
0
0
0
0
0
0


0
0
0
0
0
0
0
0
0
0
0
0
0
0
0
0
0
0
0
0
0
0
0
0
0
0
0
0
0
0
0
0
0
0
0
0
0
0
0
0
0
0
0
0
0
0
0
0
0
0
0
0
0
0
0
0
0
0
0
0
0
0
0
0
0
0
0
0
4
0
0
0
0
0
0
0
0
0
0
0
0


0
0
0
0
0
0
0
0
0
0
0
0
0
0
0
0
0
0
0
0
0
0
0
0
0
0
0
0
0
0
0
0
0
0
0
0
0
0
0
0
0
0
0
0
0
0
0
0
0
0
0
0
0
0
0
0
0
0
0
0
0
0
0
0
0
0
0
0
4
0
0
0
0
0
0
0
0
0
0
0
0


0
0
0
0
0
0
0
0
0
0
0
0
0
0
0
0
0
0
0
0
0
0
0
0
0
0
0
0
0
0
0
0
0
0
0
0
0
0
0
0
0
0
0
0
0
0
0
0
0
0
0
0
0
0
0
0
0
0
0
0
0
0
0
0
0
0
0
0
4
0
0
0
0
0
0
0
0
0
0
0
0


0
0
0
0
0
0
0
0
0
0
0
0
0
0
0
0
0
0
0
0
0
0
0
0
0
0
0
0
0
0
0
0
0
0
0
0
0
0
0
0
0
0
0
0
0
0
0
0
0
0
0
0
0
0
0
0
0
0
0
0
0
0
0
1
0
0
0
0
0
0
0
0
0
0
0
0
0
0
0
0
0


0
0
0
0
0
0
0
0
0
0
0
0
0
0
0
0
0
0
0
0
0
0
0
0
0
0
0
0
0
0
0
0
0
0
0
0
0
0
0
0
0
0
0
0
0
0
0
0
0
0
0
0
0
0
0
0
0
0
0
0
0
0
0
1
0
0
0
0
0
0
0
0
0
0
0
0
0
0
0
0
0


0
0
0
0
0
0
0
0
0
0
0
0
0
0
0
0
0
0
0
0
0
0
0
0
0
0
0
0
0
0
0
0
0
0
0
0
0
0
0
0
0
0
0
0
0
0
0
0
0
0
0
0
0
0
0
0
0
0
0
0
0
0
0
1
0
0
0
0
0
0
0
0
0
0
0
0
0
0
0
0
0


0
0
0
0
0
0
0
0
0
0
0
0
0
0
0
0
0
0
0
0
0
0
0
0
0
0
0
0
0
0
0
0
0
0
0
0
0
0
0
0
0
0
0
0
0
0
0
0
0
0
0
0
0
0
0
0
0
0
0
0
0
0
0
1
0
0
0
0
0
0
0
0
0
0
0
0
0
0
0
0
0


0
0
0
0
0
0
0
0
0
0
2
0
0
1
0
0
0
1
0
0
4
0
0
0
0
0
0
0
0
0
0
0
0
0
0
0
0
0
0
0
0
0
0
0
0
0
0
0
1
0
0
0
0
0
0
0
0
1
2
0
1
0
0
0
0
0
0
0
0
0
0
1
1
0
0
0
0
0
0
0
0


0
0
0
0
0
0
0
0
0
0
2
0
0
1
0
0
0
1
0
0
4
0
0
0
0
0
0
0
0
0
0
0
0
0
0
0
0
0
0
0
0
0
0
0
0
0
0
0
1
0
0
0
0
0
0
0
0
1
2
0
1
0
0
0
0
0
0
0
0
0
0
1
1
0
0
0
0
0
0
0
0


0
0
0
0
0
0
0
0
0
0
2
0
0
1
0
0
0
1
0
0
4
0
0
0
0
0
0
0
0
0
0
0
0
0
0
0
0
0
0
0
0
0
0
0
0
0
0
0
1
0
0
0
0
0
0
0
0
1
2
0
1
0
0
0
0
0
0
0
0
0
0
1
1
0
0
0
0
0
0
0
0


0
0
0
0
0
0
0
0
0
0
0
0
0
0
0
0
0
1
0
0
1
0
0
0
0
0
0
0
0
0
0
0
0
0
0
0
0
0
0
0
0
0
0
0
0
0
0
0
0
0
0
0
0
0
0
0
0
0
0
0
0
0
0
0
0
0
0
0
0
0
0
0
0
0
0
0
0
0
0
0
0


0
0
0
0
0
0
0
0
0
0
0
0
0
0
0
0
0
1
0
0
1
0
0
0
0
0
0
0
0
0
0
0
0
0
0
0
0
0
0
0
0
0
0
0
0
0
0
0
0
0
0
0
0
0
0
0
0
0
0
0
0
0
0
0
0
0
0
0
0
0
0
0
0
0
0
0
0
0
0
0
0


0
0
0
0
0
0
0
0
0
0
2
0
0
1
0
0
0
0
0
0
3
0
0
0
0
0
0
0
0
0
0
0
0
0
0
0
0
0
0
0
0
0
0
0
0
0
0
0
1
0
0
0
0
0
0
0
0
1
2
0
1
0
0
0
0
0
0
0
0
0
0
1
1
0
0
0
0
0
0
0
0


0
0
0
0
0
0
0
0
0
0
2
0
0
1
0
0
0
0
0
0
3
0
0
0
0
0
0
0
0
0
0
0
0
0
0
0
0
0
0
0
0
0
0
0
0
0
0
0
1
0
0
0
0
0
0
0
0
0
2
0
0
0
0
0
0
0
0
0
0
0
0
1
1
0
0
0
0
0
0
0
0


0
0
0
0
0
0
0
0
0
0
0
0
0
0
0
0
0
0
0
0
0
0
0
0
0
0
0
0
0
0
0
0
0
0
0
0
0
0
0
0
0
0
0
0
0
0
0
0
0
0
0
0
0
0
0
0
0
1
0
0
1
0
0
0
0
0
0
0
0
0
0
0
0
0
0
0
0
0
0
0
0


0
0
0
0
0
1
0
0
0
0
0
0
0
0
0
1
0
0
0
1
13
0
0
0
0
0
0
0
0
0
0
0
0
0
0
0
0
0
0
0
0
0
0
0
0
0
0
0
0
0
0
0
0
0
0
0
0
0
0
0
0
0
0
0
0
0
0
0
0
0
0
0
0
0
0
0
0
0
0
0
0


0
0
0
0
0
0
0
0
0
0
0
0
0
0
0
0
0
0
0
1
3
0
0
0
0
0
0
0
0
0
0
0
0
0
0
0
0
0
0
0
0
0
0
0
0
0
0
0
0
0
0
0
0
0
0
0
0
0
0
0
0
0
0
0
0
0
0
0
0
0
0
0
0
0
0
0
0
0
0
0
0


0
0
0
0
0
0
0
0
0
0
0
0
0
0
0
0
0
0
0
1
3
0
0
0
0
0
0
0
0
0
0
0
0
0
0
0
0
0
0
0
0
0
0
0
0
0
0
0
0
0
0
0
0
0
0
0
0
0
0
0
0
0
0
0
0
0
0
0
0
0
0
0
0
0
0
0
0
0
0
0
0


0
0
0
0
0
0
0
0
0
0
0
0
0
0
0
0
0
0
0
1
3
0
0
0
0
0
0
0
0
0
0
0
0
0
0
0
0
0
0
0
0
0
0
0
0
0
0
0
0
0
0
0
0
0
0
0
0
0
0
0
0
0
0
0
0
0
0
0
0
0
0
0
0
0
0
0
0
0
0
0
0


0
0
0
0
0
0
0
0
0
0
0
0
0
0
0
0
0
0
0
1
3
0
0
0
0
0
0
0
0
0
0
0
0
0
0
0
0
0
0
0
0
0
0
0
0
0
0
0
0
0
0
0
0
0
0
0
0
0
0
0
0
0
0
0
0
0
0
0
0
0
0
0
0
0
0
0
0
0
0
0
0


0
0
0
0
0
1
0
0
0
0
0
0
0
0
0
1
0
0
0
0
10
0
0
0
0
0
0
0
0
0
0
0
0
0
0
0
0
0
0
0
0
0
0
0
0
0
0
0
0
0
0
0
0
0
0
0
0
0
0
0
0
0
0
0
0
0
0
0
0
0
0
0
0
0
0
0
0
0
0
0
0


0
0
0
0
0
1
0
0
0
0
0
0
0
0
0
1
0
0
0
0
10
0
0
0
0
0
0
0
0
0
0
0
0
0
0
0
0
0
0
0
0
0
0
0
0
0
0
0
0
0
0
0
0
0
0
0
0
0
0
0
0
0
0
0
0
0
0
0
0
0
0
0
0
0
0
0
0
0
0
0
0


0
0
0
0
0
1
0
0
0
0
0
0
0
0
0
1
0
0
0
0
10
0
0
0
0
0
0
0
0
0
0
0
0
0
0
0
0
0
0
0
0
0
0
0
0
0
0
0
0
0
0
0
0
0
0
0
0
0
0
0
0
0
0
0
0
0
0
0
0
0
0
0
0
0
0
0
0
0
0
0
0


0
0
0
0
0
1
0
0
0
0
0
0
0
0
0
1
0
0
0
0
6
0
0
0
0
0
0
0
0
0
0
0
0
0
0
0
0
0
0
0
0
0
0
0
0
0
0
0
0
0
0
0
0
0
0
0
0
0
0
0
0
0
0
0
0
0
0
0
0
0
0
0
0
0
0
0
0
0
0
0
0


0
0
0
0
0
0
0
0
0
0
0
0
0
0
0
0
0
0
0
0
4
0
0
0
0
0
0
0
0
0
0
0
0
0
0
0
0
0
0
0
0
0
0
0
0
0
0
0
0
0
0
0
0
0
0
0
0
0
0
0
0
0
0
0
0
0
0
0
0
0
0
0
0
0
0
0
0
0
0
0
0


73
112
225
54
40
93
76
60
46
9
3
9
15
24
2
12
21
41
9
30
14
12
2
0
0
4
0
64
0
9
77
0
0
0
10
6
0
138
194
31
31
12
9
6
2
8
63
30
75
0
70
11
71
244
12
46
24
86
133
74
279
83
4
141
14
12
127
42
166
9
277
2
1
0
0
7
1
7
1
0
11


14
38
132
21
10
58
22
30
27
2
1
0
6
13
0
1
4
20
2
4
4
6
0
0
0
4
0
18
0
0
4
0
0
0
0
1
0
103
16
3
5
8
4
2
1
1
29
6
6
0
40
8
21
171
4
27
6
51
112
27
235
40
0
78
4
8
80
37
113
9
276
2
0
0
0
1
0
6
0
0
0


13
14
102
13
10
47
9
6
2
1
0
0
0
2
0
1
0
0
1
4
0
1
0
0
0
1
0
3
0
0
0
0
0
0
0
0
0
8
4
0
0
0
1
2
1
0
3
6
0
0
9
4
10
38
3
8
0
23
4
7
69
7
0
26
2
0
35
1
26
1
5
1
0
0
0
1
0
0
0
0
0


13
14
102
13
10
47
9
6
2
1
0
0
0
2
0
1
0
0
1
4
0
1
0
0
0
1
0
3
0
0
0
0
0
0
0
0
0
8
4
0
0
0
1
2
1
0
3
6
0
0
9
4
10
38
3
8
0
23
4
7
69
7
0
26
2
0
35
1
26
1
5
1
0
0
0
1
0
0
0
0
0


13
14
102
13
10
47
9
6
2
1
0
0
0
2
0
1
0
0
1
4
0
1
0
0
0
1
0
3
0
0
0
0
0
0
0
0
0
8
4
0
0
0
1
2
1
0
3
6
0
0
9
4
10
38
3
8
0
23
4
7
69
7
0
26
2
0
35
1
26
1
5
1
0
0
0
1
0
0
0
0
0


0
0
0
0
0
6
3
12
0
0
0
0
0
0
0
0
0
0
1
0
3
0
0
0
0
0
0
0
0
0
0
0
0
0
0
0
0
0
0
0
0
0
0
0
0
0
0
0
0
0
0
0
10
0
0
0
1
0
7
0
0
0
0
0
0
0
12
0
26
5
222
1
0
0
0
0
0
6
0
0
0


0
0
0
0
0
6
3
12
0
0
0
0
0
0
0
0
0
0
1
0
2
0
0
0
0
0
0
0
0
0
0
0
0
0
0
0
0
0
0
0
0
0
0
0
0
0
0
0
0
0
0
0
10
0
0
0
0
0
0
0
0
0
0
0
0
0
12
0
26
5
222
1
0
0
0
0
0
0
0
0
0


0
0
0
0
0
6
3
12
0
0
0
0
0
0
0
0
0
0
1
0
2
0
0
0
0
0
0
0
0
0
0
0
0
0
0
0
0
0
0
0
0
0
0
0
0
0
0
0
0
0
0
0
10
0
0
0
0
0
0
0
0
0
0
0
0
0
12
0
26
5
222
1
0
0
0
0
0
0
0
0
0


0
0
0
0
0
0
0
0
0
0
0
0
0
0
0
0
0
0
0
0
1
0
0
0
0
0
0
0
0
0
0
0
0
0
0
0
0
0
0
0
0
0
0
0
0
0
0
0
0
0
0
0
0
0
0
0
0
0
0
0
0
0
0
0
0
0
0
0
0
0
0
0
0
0
0
0
0
0
0
0
0


0
0
0
0
0
0
0
0
0
0
0
0
0
0
0
0
0
0
0
0
1
0
0
0
0
0
0
0
0
0
0
0
0
0
0
0
0
0
0
0
0
0
0
0
0
0
0
0
0
0
0
0
0
0
0
0
0
0
0
0
0
0
0
0
0
0
0
0
0
0
0
0
0
0
0
0
0
0
0
0
0


0
0
0
0
0
0
0
0
0
0
0
0
0
0
0
0
0
0
0
0
0
0
0
0
0
0
0
0
0
0
0
0
0
0
0
0
0
0
0
0
0
0
0
0
0
0
0
0
0
0
0
0
0
0
0
0
1
0
7
0
0
0
0
0
0
0
0
0
0
0
0
0
0
0
0
0
0
6
0
0
0


0
0
0
0
0
0
0
0
0
0
0
0
0
0
0
0
0
0
0
0
0
0
0
0
0
0
0
0
0
0
0
0
0
0
0
0
0
0
0
0
0
0
0
0
0
0
0
0
0
0
0
0
0
0
0
0
1
0
7
0
0
0
0
0
0
0
0
0
0
0
0
0
0
0
0
0
0
6
0
0
0


1
24
30
8
0
5
10
12
25
1
1
0
6
11
0
0
4
20
0
0
1
5
0
0
0
3
0
15
0
0
4
0
0
0
0
1
0
95
12
3
5
8
3
0
0
1
26
0
6
0
31
4
1
133
1
19
5
28
101
20
166
33
0
52
2
8
33
36
61
3
49
0
0
0
0
0
0
0
0
0
0


1
24
30
8
0
5
10
12
25
1
1
0
6
11
0
0
4
20
0
0
1
5
0
0
0
3
0
15
0
0
4
0
0
0
0
1
0
95
12
3
5
8
3
0
0
1
26
0
6
0
31
4
1
133
1
19
5
28
101
20
166
33
0
52
2
8
33
36
61
3
49
0
0
0
0
0
0
0
0
0
0


1
24
30
8
0
5
10
12
25
1
1
0
6
11
0
0
4
20
0
0
1
5
0
0
0
3
0
15
0
0
4
0
0
0
0
1
0
95
12
3
5
8
3
0
0
1
26
0
6
0
31
4
1
133
1
19
5
28
101
20
166
33
0
52
2
8
33
36
61
3
49
0
0
0
0
0
0
0
0
0
0


59
74
93
33
30
35
54
30
19
7
2
9
9
11
2
11
17
21
7
26
10
6
2
0
0
0
0
46
0
9
73
0
0
0
10
5
0
35
178
28
26
4
5
4
1
7
34
24
69
0
30
3
50
73
8
19
18
35
21
47
44
43
4
63
10
4
47
5
53
0
1
0
1
0
0
6
1
1
1
0
11


59
74
93
33
30
35
54
30
19
7
2
9
9
11
2
11
17
21
7
26
10
6
2
0
0
0
0
46
0
9
73
0
0
0
10
5
0
35
178
28
26
4
5
4
1
7
34
24
69
0
30
3
50
73
8
19
18
35
21
47
44
43
4
63
10
4
47
5
53
0
1
0
1
0
0
6
1
1
1
0
11


59
74
93
33
30
35
54
30
19
7
2
9
9
11
2
11
17
21
7
26
10
6
2
0
0
0
0
46
0
9
73
0
0
0
10
5
0
35
178
28
26
4
5
4
1
7
34
24
69
0
30
3
50
73
8
19
18
35
21
47
44
43
4
63
10
4
47
5
53
0
1
0
1
0
0
6
1
1
1
0
11


59
74
93
33
30
35
54
30
19
7
2
9
9
11
2
11
17
21
7
26
10
6
2
0
0
0
0
46
0
9
73
0
0
0
10
5
0
35
178
28
26
4
5
4
1
7
34
24
69
0
30
3
50
73
8
19
18
35
21
47
44
43
4
63
10
4
47
5
53
0
1
0
1
0
0
6
1
1
1
0
11


8
14
109
0
7
41
24
7
2
0
0
0
1
13
3
10
16
20
0
17
2
2
3
0
0
0
0
41
0
0
8
0
0
0
4
0
0
18
71
6
5
4
1
1
2
6
66
10
7
0
0
3
4
30
4
3
4
4
0
9
10
1
0
11
0
2
11
4
24
0
0
0
0
0
0
0
0
0
0
0
0


8
14
109
0
7
41
24
7
2
0
0
0
1
13
3
10
16
20
0
17
2
2
3
0
0
0
0
41
0
0
8
0
0
0
4
0
0
18
71
6
5
4
1
1
2
6
66
10
7
0
0
3
4
30
4
3
4
4
0
9
10
1
0
11
0
2
11
4
24
0
0
0
0
0
0
0
0
0
0
0
0


8
14
109
0
7
41
24
7
2
0
0
0
1
13
3
10
16
20
0
17
2
2
3
0
0
0
0
41
0
0
8
0
0
0
4
0
0
18
71
6
5
4
1
1
2
6
66
10
7
0
0
3
4
30
4
3
4
4
0
9
10
1
0
11
0
2
11
4
24
0
0
0
0
0
0
0
0
0
0
0
0


8
14
109
0
7
41
24
7
2
0
0
0
1
13
3
10
16
20
0
17
2
2
3
0
0
0
0
41
0
0
8
0
0
0
4
0
0
18
71
6
5
4
1
1
2
6
66
10
7
0
0
3
4
30
4
3
4
4
0
9
10
1
0
11
0
2
11
4
24
0
0
0
0
0
0
0
0
0
0
0
0


8
14
109
0
7
41
24
7
2
0
0
0
1
13
3
10
16
20
0
17
2
2
3
0
0
0
0
41
0
0
8
0
0
0
4
0
0
18
71
6
5
4
1
1
2
6
66
10
7
0
0
3
4
30
4
3
4
4
0
9
10
1
0
11
0
2
11
4
24
0
0
0
0
0
0
0
0
0
0
0
0


29
1
42
15
8
4
30
63
5
4
1
2
2
227
9
16
4
7
7
3
17
4
5
0
0
0
0
0
0
5
2
0
0
0
3
0
0
15
33
0
10
3
0
0
6
5
12
0
5
0
38
0
11
8
12
18
7
6
3
5
10
40
6
13
41
32
25
27
13
13
3
12
6
4
6
0
5
18
7
0
4


29
1
42
15
8
4
30
63
5
4
1
2
2
227
9
16
4
7
7
3
17
4
5
0
0
0
0
0
0
5
2
0
0
0
3
0
0
15
33
0
10
3
0
0
6
5
12
0
5
0
38
0
11
8
12
18
7
6
3
5
10
40
6
13
41
32
25
27
13
13
3
12
6
4
6
0
5
18
7
0
4


5
0
40
4
3
0
11
5
1
1
0
0
2
4
3
9
0
2
5
2
2
2
0
0
0
0
0
0
0
0
0
0
0
0
3
0
0
2
12
0
3
0
0
0
4
1
2
0
0
0
16
0
2
0
0
4
1
1
1
3
0
11
1
1
15
3
1
2
1
0
0
0
0
0
0
0
0
0
0
0
0


5
0
40
4
3
0
11
5
1
1
0
0
2
4
3
9
0
2
5
2
2
2
0
0
0
0
0
0
0
0
0
0
0
0
3
0
0
2
12
0
3
0
0
0
4
1
2
0
0
0
16
0
2
0
0
4
1
1
1
3
0
11
1
1
15
3
1
2
1
0
0
0
0
0
0
0
0
0
0
0
0


5
0
40
4
3
0
11
5
1
1
0
0
2
4
3
9
0
2
5
2
2
2
0
0
0
0
0
0
0
0
0
0
0
0
3
0
0
2
12
0
3
0
0
0
4
1
2
0
0
0
16
0
2
0
0
4
1
1
1
3
0
11
1
1
15
3
1
2
1
0
0
0
0
0
0
0
0
0
0
0
0


5
0
40
4
3
0
11
5
1
1
0
0
2
4
3
9
0
2
5
2
2
2
0
0
0
0
0
0
0
0
0
0
0
0
3
0
0
2
12
0
3
0
0
0
4
1
2
0
0
0
16
0
2
0
0
4
1
1
1
3
0
11
1
1
15
3
1
2
1
0
0
0
0
0
0
0
0
0
0
0
0


24
1
2
8
5
4
19
58
4
3
1
2
0
223
6
7
4
5
2
1
15
2
5
0
0
0
0
0
0
5
2
0
0
0
0
0
0
13
21
0
7
3
0
0
2
4
10
0
5
0
22
0
9
7
12
12
6
5
2
2
10
29
5
12
26
27
24
25
12
13
1
12
6
4
6
0
5
18
7
0
4


24
1
2
8
5
4
19
58
4
3
1
2
0
223
6
7
4
5
2
1
15
2
5
0
0
0
0
0
0
5
2
0
0
0
0
0
0
13
21
0
7
3
0
0
2
4
10
0
5
0
22
0
9
7
12
12
6
5
2
2
10
29
5
12
26
27
24
25
12
13
1
12
6
4
6
0
5
18
7
0
4


24
1
2
8
5
4
19
58
4
3
1
2
0
223
6
7
4
5
2
1
15
2
5
0
0
0
0
0
0
5
2
0
0
0
0
0
0
13
21
0
7
3
0
0
2
4
10
0
5
0
22
0
9
7
12
12
6
5
2
2
10
29
5
12
26
27
24
25
12
13
1
12
6
4
6
0
5
18
7
0
4


24
1
2
8
5
4
19
58
4
3
1
2
0
223
6
7
4
5
2
1
15
2
5
0
0
0
0
0
0
5
2
0
0
0
0
0
0
13
21
0
7
3
0
0
2
4
10
0
5
0
22
0
9
7
12
12
6
5
2
2
10
29
5
12
26
27
24
25
12
13
1
12
6
4
6
0
5
18
7
0
4


0
0
0
3
0
0
0
0
0
0
0
0
0
0
0
0
0
0
0
0
0
0
0
0
0
0
0
0
0
0
0
0
0
0
0
0
0
0
0
0
0
0
0
0
0
0
0
0
0
0
0
0
0
1
0
2
0
0
0
0
0
0
0
0
0
2
0
0
0
0
2
0
0
0
0
0
0
0
0
0
0


0
0
0
3
0
0
0
0
0
0
0
0
0
0
0
0
0
0
0
0
0
0
0
0
0
0
0
0
0
0
0
0
0
0
0
0
0
0
0
0
0
0
0
0
0
0
0
0
0
0
0
0
0
1
0
2
0
0
0
0
0
0
0
0
0
2
0
0
0
0
2
0
0
0
0
0
0
0
0
0
0


0
0
0
3
0
0
0
0
0
0
0
0
0
0
0
0
0
0
0
0
0
0
0
0
0
0
0
0
0
0
0
0
0
0
0
0
0
0
0
0
0
0
0
0
0
0
0
0
0
0
0
0
0
1
0
2
0
0
0
0
0
0
0
0
0
2
0
0
0
0
2
0
0
0
0
0
0
0
0
0
0


0
0
0
3
0
0
0
0
0
0
0
0
0
0
0
0
0
0
0
0
0
0
0
0
0
0
0
0
0
0
0
0
0
0
0
0
0
0
0
0
0
0
0
0
0
0
0
0
0
0
0
0
0
1
0
2
0
0
0
0
0
0
0
0
0
2
0
0
0
0
2
0
0
0
0
0
0
0
0
0
0


3
0
0
1
1
5
1
2
0
0
0
0
0
2
0
0
2
0
1
0
0
0
0
0
0
0
0
0
0
0
0
0
0
0
0
0
0
2
0
0
0
0
0
0
2
0
1
0
0
0
7
0
1
2
1
2
0
0
1
0
1
4
0
3
2
1
1
0
5
0
1
0
0
1
0
0
0
0
0
0
0


3
0
0
1
1
5
1
2
0
0
0
0
0
2
0
0
2
0
1
0
0
0
0
0
0
0
0
0
0
0
0
0
0
0
0
0
0
2
0
0
0
0
0
0
2
0
1
0
0
0
7
0
1
2
1
2
0
0
1
0
1
4
0
3
2
1
1
0
5
0
1
0
0
1
0
0
0
0
0
0
0


3
0
0
1
1
5
1
2
0
0
0
0
0
2
0
0
2
0
1
0
0
0
0
0
0
0
0
0
0
0
0
0
0
0
0
0
0
2
0
0
0
0
0
0
2
0
1
0
0
0
7
0
1
2
1
2
0
0
1
0
1
4
0
3
2
1
1
0
5
0
1
0
0
1
0
0
0
0
0
0
0


3
0
0
1
1
5
1
2
0
0
0
0
0
2
0
0
2
0
1
0
0
0
0
0
0
0
0
0
0
0
0
0
0
0
0
0
0
2
0
0
0
0
0
0
2
0
1
0
0
0
7
0
1
2
1
2
0
0
1
0
1
4
0
3
2
1
1
0
5
0
1
0
0
1
0
0
0
0
0
0
0


3
0
0
1
1
5
1
2
0
0
0
0
0
2
0
0
2
0
1
0
0
0
0
0
0
0
0
0
0
0
0
0
0
0
0
0
0
2
0
0
0
0
0
0
2
0
1
0
0
0
7
0
1
2
1
2
0
0
1
0
1
4
0
3
2
1
1
0
5
0
1
0
0
1
0
0
0
0
0
0
0


3
0
0
1
1
5
1
2
0
0
0
0
0
2
0
0
2
0
1
0
0
0
0
0
0
0
0
0
0
0
0
0
0
0
0
0
0
2
0
0
0
0
0
0
2
0
1
0
0
0
7
0
1
2
1
2
0
0
1
0
1
4
0
3
2
1
1
0
5
0
1
0
0
1
0
0
0
0
0
0
0


34858
36625
38812
36258
35416
39447
36442
32288
34373
28261
33543
31439
34866
33739
29875
30235
39763
43226
36006
35858
33183
34223
33084
19017
17601
30436
18122
28745
41773
32337
32371
25660
27026
29166
25859
35655
17497
23606
29770
34860
29799
33798
29677
32843
34714
33896
32868
29413
38809
16532
34717
31273
38135
33018
40904
38453
34773
36490
42060
37847
50488
41532
31783
29747
35371
39670
32125
39621
39443
30671
33250
39651
47941
37389
22700
37373
28045
28344
38817
20927
22194


0
0
0
1
0
0
0
0
0
0
0
0
0
0
1
0
0
0
0
0
5
0
0
0
0
0
0
0
0
0
0
1
0
0
0
0
0
0
0
0
0
0
0
0
0
0
1
0
0
0
0
0
0
0
0
0
0
0
0
0
0
0
0
0
0
1
0
0
0
0
0
0
0
0
0
0
0
0
0
0
0


0
0
0
1
0
0
0
0
0
0
0
0
0
0
1
0
0
0
0
0
5
0
0
0
0
0
0
0
0
0
0
1
0
0
0
0
0
0
0
0
0
0
0
0
0
0
1
0
0
0
0
0
0
0
0
0
0
0
0
0
0
0
0
0
0
1
0
0
0
0
0
0
0
0
0
0
0
0
0
0
0


0
0
0
1
0
0
0
0
0
0
0
0
0
0
1
0
0
0
0
0
5
0
0
0
0
0
0
0
0
0
0
1
0
0
0
0
0
0
0
0
0
0
0
0
0
0
1
0
0
0
0
0
0
0
0
0
0
0
0
0
0
0
0
0
0
1
0
0
0
0
0
0
0
0
0
0
0
0
0
0
0


0
0
0
1
0
0
0
0
0
0
0
0
0
0
1
0
0
0
0
0
5
0
0
0
0
0
0
0
0
0
0
1
0
0
0
0
0
0
0
0
0
0
0
0
0
0
1
0
0
0
0
0
0
0
0
0
0
0
0
0
0
0
0
0
0
1
0
0
0
0
0
0
0
0
0
0
0
0
0
0
0


0
0
0
1
0
0
0
0
0
0
0
0
0
0
1
0
0
0
0
0
5
0
0
0
0
0
0
0
0
0
0
1
0
0
0
0
0
0
0
0
0
0
0
0
0
0
1
0
0
0
0
0
0
0
0
0
0
0
0
0
0
0
0
0
0
1
0
0
0
0
0
0
0
0
0
0
0
0
0
0
0


0
0
0
1
0
0
0
0
0
0
0
0
0
0
1
0
0
0
0
0
5
0
0
0
0
0
0
0
0
0
0
1
0
0
0
0
0
0
0
0
0
0
0
0
0
0
1
0
0
0
0
0
0
0
0
0
0
0
0
0
0
0
0
0
0
1
0
0
0
0
0
0
0
0
0
0
0
0
0
0
0


134
0
15
537
104
623
37
75
4
18
2
2
0
0
4
32
38
1
38
87
178
219
14
50
0
18
0
2
0
56
151
332
1
798
385
38
93
0
23
42
64
121
228
229
212
127
6
15
33
0
17
799
1315
41
25
71
306
96
54
20
49
26
84
11
268
139
1156
9
855
1240
115
270
379
169
540
404
353
433
299
36
158


127
0
15
526
98
545
36
65
4
16
2
1
0
0
3
30
38
1
37
82
178
217
14
50
0
18
0
2
0
54
144
271
1
783
385
36
93
0
22
41
63
120
227
228
211
124
5
15
32
0
16
478
892
36
23
68
274
89
47
18
45
24
80
10
262
136
969
9
773
1199
110
209
245
132
238
324
206
212
218
30
117


127
0
15
526
98
545
36
65
4
16
2
1
0
0
3
30
38
1
37
82
178
217
14
50
0
18
0
2
0
54
144
271
1
783
385
36
93
0
22
41
63
120
227
228
211
124
5
15
32
0
16
478
892
36
23
68
274
89
47
18
45
24
80
10
262
136
969
9
773
1199
110
209
245
132
238
324
206
212
218
30
117


127
0
15
526
98
545
36
65
4
16
2
1
0
0
3
30
38
1
37
82
178
217
14
50
0
18
0
2
0
54
144
271
1
783
385
36
93
0
22
41
63
120
227
228
211
124
5
15
32
0
16
478
892
36
23
68
274
89
47
18
45
24
80
10
262
136
969
9
773
1199
110
209
245
132
238
324
206
212
218
30
117


127
0
15
526
98
545
36
65
4
16
2
1
0
0
3
30
38
1
37
82
178
217
14
50
0
18
0
2
0
54
144
271
1
783
385
36
93
0
22
41
63
120
227
228
211
124
5
15
32
0
16
478
892
36
23
68
274
89
47
18
45
24
80
10
262
136
969
9
773
1199
110
209
245
132
238
324
206
212
218
30
117


127
0
15
526
98
545
36
65
4
16
2
1
0
0
3
30
38
1
37
82
178
217
14
50
0
18
0
2
0
54
144
271
1
783
385
36
93
0
22
41
63
120
227
228
211
124
5
15
32
0
16
478
892
36
23
68
274
89
47
18
45
24
80
10
262
136
969
9
773
1199
110
209
245
132
238
324
206
212
218
30
117


5
0
0
9
2
65
0
4
0
2
0
0
0
0
0
1
0
0
0
5
0
0
0
0
0
0
0
0
0
2
7
61
0
14
0
2
0
0
0
0
0
0
0
1
0
3
0
0
1
0
1
315
413
5
2
2
26
6
4
2
3
1
2
1
3
1
175
0
67
37
1
58
132
37
299
72
144
216
75
6
39


5
0
0
9
2
65
0
4
0
2
0
0
0
0
0
1
0
0
0
5
0
0
0
0
0
0
0
0
0
2
7
61
0
14
0
2
0
0
0
0
0
0
0
1
0
3
0
0
1
0
1
315
413
5
2
2
26
6
4
2
3
1
2
1
3
1
175
0
67
37
1
58
132
37
299
72
144
216
75
6
39


5
0
0
9
2
65
0
4
0
2
0
0
0
0
0
1
0
0
0
5
0
0
0
0
0
0
0
0
0
2
7
61
0
14
0
2
0
0
0
0
0
0
0
1
0
3
0
0
1
0
1
315
413
5
2
2
26
6
4
2
3
1
2
1
3
1
175
0
67
37
1
58
132
37
299
72
144
216
75
6
39


5
0
0
9
2
65
0
4
0
2
0
0
0
0
0
1
0
0
0
5
0
0
0
0
0
0
0
0
0
2
7
61
0
14
0
2
0
0
0
0
0
0
0
1
0
3
0
0
1
0
1
315
413
5
2
2
26
6
4
2
3
1
2
1
3
1
175
0
67
37
1
58
132
37
299
72
144
216
75
6
39


5
0
0
9
2
65
0
4
0
2
0
0
0
0
0
1
0
0
0
5
0
0
0
0
0
0
0
0
0
2
7
61
0
14
0
2
0
0
0
0
0
0
0
1
0
3
0
0
1
0
1
315
413
5
2
2
26
6
4
2
3
1
2
1
3
1
175
0
67
37
1
58
132
37
299
72
144
216
75
6
39


2
0
0
2
4
13
1
6
0
0
0
1
0
0
1
1
0
0
1
0
0
2
0
0
0
0
0
0
0
0
0
0
0
1
0
0
0
0
1
1
1
1
1
0
1
0
1
0
0
0
0
6
10
0
0
1
6
1
3
0
1
1
2
0
3
2
12
0
15
4
4
3
2
0
3
8
3
5
6
0
2


2
0
0
2
4
13
1
6
0
0
0
1
0
0
1
1
0
0
1
0
0
2
0
0
0
0
0
0
0
0
0
0
0
1
0
0
0
0
1
1
1
1
1
0
1
0
1
0
0
0
0
6
10
0
0
1
6
1
3
0
1
1
2
0
3
2
12
0
15
4
4
3
2
0
3
8
3
5
6
0
2


2
0
0
2
4
13
1
6
0
0
0
1
0
0
1
1
0
0
1
0
0
2
0
0
0
0
0
0
0
0
0
0
0
1
0
0
0
0
1
1
1
1
1
0
1
0
1
0
0
0
0
6
10
0
0
1
6
1
3
0
1
1
2
0
3
2
12
0
15
4
4
3
2
0
3
8
3
5
6
0
2


2
0
0
2
4
13
1
6
0
0
0
1
0
0
1
1
0
0
1
0
0
2
0
0
0
0
0
0
0
0
0
0
0
1
0
0
0
0
1
1
1
1
1
0
1
0
1
0
0
0
0
6
10
0
0
1
6
1
3
0
1
1
2
0
3
2
12
0
15
4
4
3
2
0
3
8
3
5
6
0
2


2
0
0
2
4
13
1
6
0
0
0
1
0
0
1
1
0
0
1
0
0
2
0
0
0
0
0
0
0
0
0
0
0
1
0
0
0
0
1
1
1
1
1
0
1
0
1
0
0
0
0
6
10
0
0
1
6
1
3
0
1
1
2
0
3
2
12
0
15
4
4
3
2
0
3
8
3
5
6
0
2


6620
7125
7428
8002
4025
7718
5840
6971
6440
3885
4355
4595
5429
6783
4747
3952
6843
8203
3843
5552
4889
3974
5362
340
1211
3908
511
4143
3811
8282
5258
2194
1355
2538
6654
7880
790
6216
7042
3368
3970
6098
6462
6908
6739
6272
4138
5550
7751
1564
6604
8582
9627
6142
6581
7824
7228
7562
8233
7072
8818
7505
5134
6165
8718
8120
10035
6637
9162
9082
6092
11023
14015
9222
7352
12786
8118
7850
13233
5959
7196


59
3
6
3
10
0
130
78
47
19
7
8
0
23
3
5
9
6
9
13
31
9
3
0
0
2
0
2
0
8
3
0
0
0
79
1
0
0
12
3
6
45
53
8
2
3
6
1
4
0
139
0
2
66
289
31
11
10
26
17
43
156
2
135
10
28
22
224
31
6
19
0
0
1
0
0
0
0
0
0
0


59
3
6
3
10
0
130
78
47
19
7
8
0
23
3
5
9
6
9
13
31
9
3
0
0
2
0
2
0
8
3
0
0
0
79
1
0
0
12
3
6
45
53
8
2
3
6
1
4
0
139
0
2
66
289
31
11
10
26
17
43
156
2
135
10
28
22
224
31
6
19
0
0
1
0
0
0
0
0
0
0


59
3
6
3
10
0
130
78
47
19
7
8
0
23
3
5
9
6
9
13
31
9
3
0
0
2
0
2
0
8
3
0
0
0
79
1
0
0
12
3
6
45
53
8
2
3
6
1
4
0
139
0
2
66
289
31
11
10
26
17
43
156
2
135
10
28
22
224
31
6
19
0
0
1
0
0
0
0
0
0
0


59
3
6
3
10
0
130
78
47
19
7
8
0
23
3
5
9
6
9
13
31
9
3
0
0
2
0
2
0
8
3
0
0
0
79
1
0
0
12
3
6
45
53
8
2
3
6
1
4
0
139
0
2
66
289
31
11
10
26
17
43
156
2
135
10
28
22
224
31
6
19
0
0
1
0
0
0
0
0
0
0


59
3
6
3
10
0
130
78
47
19
7
8
0
23
3
5
9
6
9
13
31
9
3
0
0
2
0
2
0
8
3
0
0
0
79
1
0
0
12
3
6
45
53
8
2
3
6
1
4
0
139
0
2
66
289
31
11
10
26
17
43
156
2
135
10
28
22
224
31
6
19
0
0
1
0
0
0
0
0
0
0


0
0
0
0
0
0
13
5
0
0
0
4
0
1
0
0
1
0
0
4
0
0
0
0
0
0
0
5
0
0
0
0
0
0
0
0
0
0
0
1
1
3
1
0
0
0
2
0
0
0
0
1
2
4
1
0
1
1
0
5
0
0
1
1
0
2
0
0
0
0
0
0
0
0
0
0
0
0
0
0
0


0
0
0
0
0
0
13
5
0
0
0
4
0
1
0
0
1
0
0
4
0
0
0
0
0
0
0
5
0
0
0
0
0
0
0
0
0
0
0
1
1
3
1
0
0
0
2
0
0
0
0
1
2
4
1
0
1
1
0
5
0
0
1
1
0
2
0
0
0
0
0
0
0
0
0
0
0
0
0
0
0


0
0
0
0
0
0
13
5
0
0
0
4
0
1
0
0
1
0
0
4
0
0
0
0
0
0
0
5
0
0
0
0
0
0
0
0
0
0
0
1
1
3
1
0
0
0
2
0
0
0
0
1
2
4
1
0
1
1
0
5
0
0
1
1
0
2
0
0
0
0
0
0
0
0
0
0
0
0
0
0
0


0
0
0
0
0
0
13
5
0
0
0
4
0
1
0
0
1
0
0
4
0
0
0
0
0
0
0
5
0
0
0
0
0
0
0
0
0
0
0
1
1
3
1
0
0
0
2
0
0
0
0
1
2
4
1
0
1
1
0
5
0
0
1
1
0
2
0
0
0
0
0
0
0
0
0
0
0
0
0
0
0


0
0
0
0
0
0
13
5
0
0
0
4
0
1
0
0
1
0
0
4
0
0
0
0
0
0
0
5
0
0
0
0
0
0
0
0
0
0
0
1
1
3
1
0
0
0
2
0
0
0
0
1
2
4
1
0
1
1
0
5
0
0
1
1
0
2
0
0
0
0
0
0
0
0
0
0
0
0
0
0
0


482
366
145
197
179
112
436
506
455
444
329
352
399
522
314
164
282
195
197
251
139
149
246
1
23
70
0
106
0
315
271
35
11
167
493
61
0
166
149
58
93
426
628
371
427
721
71
91
424
0
654
173
212
231
340
381
206
266
481
283
636
767
53
378
482
490
318
410
530
187
409
364
302
255
87
374
112
70
338
208
155


482
366
145
197
179
112
436
506
455
444
329
352
399
522
314
164
282
195
197
251
139
149
246
1
23
70
0
106
0
315
271
35
11
167
493
61
0
166
149
58
93
426
628
371
427
721
71
91
424
0
654
173
212
231
340
381
206
266
481
283
636
767
53
378
482
490
318
410
530
187
409
364
302
255
87
374
112
70
338
208
155


482
366
145
197
179
112
436
506
455
444
329
352
399
522
314
164
282
195
197
251
139
149
246
1
23
70
0
106
0
315
271
35
11
167
493
61
0
166
149
58
93
426
628
371
427
721
71
91
424
0
654
173
212
231
340
381
206
266
481
283
636
767
53
378
482
490
318
410
530
187
409
364
302
255
87
374
112
70
338
208
155


482
366
145
197
179
112
436
506
455
444
329
352
399
522
314
164
282
195
197
251
139
149
246
1
23
70
0
106
0
315
271
35
11
167
493
61
0
166
149
58
93
426
628
371
427
721
71
91
424
0
654
173
212
231
340
381
206
266
481
283
636
767
53
378
482
490
318
410
530
187
409
364
302
255
87
374
112
70
338
208
155


482
366
145
197
179
112
436
506
455
444
329
352
399
522
314
164
282
195
197
251
139
149
246
1
23
70
0
106
0
315
271
35
11
167
493
61
0
166
149
58
93
426
628
371
427
721
71
91
424
0
654
173
212
231
340
381
206
266
481
283
636
767
53
378
482
490
318
410
530
187
409
364
302
255
87
374
112
70
338
208
155


2234
2866
4015
701
1631
1174
2462
1734
2129
1269
1928
1720
2854
1724
1814
1719
2868
2480
1858
2093
1649
1962
2720
178
536
1833
434
1411
2129
2207
1065
69
204
273
1681
534
43
2197
1751
1064
1883
2594
2634
1953
1222
1207
1399
1978
1907
425
1736
660
1171
2434
2760
1872
1782
1564
3040
3061
3078
2054
302
2036
1306
2575
1023
2031
1343
1007
1396
666
522
902
145
535
144
230
701
525
468


4
3
0
2
5
1
3
1
3
1
2
5
2
2
1
6
2
1
3
4
3
2
4
0
0
0
0
0
0
2
1
0
0
0
0
0
0
0
0
0
1
1
4
2
1
1
1
1
2
0
7
0
0
1
0
2
2
0
1
0
0
2
0
2
1
1
1
3
0
0
2
0
1
0
0
0
0
0
0
0
0


4
3
0
2
5
1
3
1
3
1
2
5
2
2
1
6
2
1
3
4
3
2
4
0
0
0
0
0
0
2
1
0
0
0
0
0
0
0
0
0
1
1
4
2
1
1
1
1
2
0
7
0
0
1
0
2
2
0
1
0
0
2
0
2
1
1
1
3
0
0
2
0
1
0
0
0
0
0
0
0
0


4
3
0
2
5
1
3
1
3
1
2
5
2
2
1
6
2
1
3
4
3
2
4
0
0
0
0
0
0
2
1
0
0
0
0
0
0
0
0
0
1
1
4
2
1
1
1
1
2
0
7
0
0
1
0
2
2
0
1
0
0
2
0
2
1
1
1
3
0
0
2
0
1
0
0
0
0
0
0
0
0


4
3
0
2
5
1
3
1
3
1
2
5
2
2
1
6
2
1
3
4
3
2
4
0
0
0
0
0
0
2
1
0
0
0
0
0
0
0
0
0
1
1
4
2
1
1
1
1
2
0
7
0
0
1
0
2
2
0
1
0
0
2
0
2
1
1
1
3
0
0
2
0
1
0
0
0
0
0
0
0
0


0
0
0
0
0
0
1
0
0
0
0
0
0
0
0
0
0
0
0
0
0
1
0
0
0
0
0
0
0
0
0
0
0
0
0
0
0
0
0
0
0
0
0
0
0
0
0
0
0
0
0
0
0
0
0
0
0
0
0
0
0
0
0
2
0
0
0
0
0
0
0
0
0
0
0
0
0
0
0
0
0


0
0
0
0
0
0
1
0
0
0
0
0
0
0
0
0
0
0
0
0
0
1
0
0
0
0
0
0
0
0
0
0
0
0
0
0
0
0
0
0
0
0
0
0
0
0
0
0
0
0
0
0
0
0
0
0
0
0
0
0
0
0
0
2
0
0
0
0
0
0
0
0
0
0
0
0
0
0
0
0
0


0
0
0
0
0
0
1
0
0
0
0
0
0
0
0
0
0
0
0
0
0
1
0
0
0
0
0
0
0
0
0
0
0
0
0
0
0
0
0
0
0
0
0
0
0
0
0
0
0
0
0
0
0
0
0
0
0
0
0
0
0
0
0
2
0
0
0
0
0
0
0
0
0
0
0
0
0
0
0
0
0


0
0
0
0
0
0
1
0
0
0
0
0
0
0
0
0
0
0
0
0
0
1
0
0
0
0
0
0
0
0
0
0
0
0
0
0
0
0
0
0
0
0
0
0
0
0
0
0
0
0
0
0
0
0
0
0
0
0
0
0
0
0
0
2
0
0
0
0
0
0
0
0
0
0
0
0
0
0
0
0
0


33
31
35
11
25
15
91
19
48
30
42
29
47
34
20
36
73
28
69
29
28
43
28
98
0
9
9
21
0
5
6
0
35
0
22
1
0
29
26
12
23
31
17
8
5
3
15
31
34
0
51
9
17
86
96
79
71
31
77
130
127
66
4
97
42
89
31
96
55
52
43
2
1
8
0
0
0
0
9
4
3


33
31
35
11
25
15
91
19
48
30
42
29
47
34
20
36
73
28
69
29
28
43
28
98
0
9
9
21
0
5
6
0
35
0
22
1
0
29
26
12
23
31
17
8
5
3
15
31
34
0
51
9
17
86
96
79
71
31
77
130
127
66
4
97
42
89
31
96
55
52
43
2
1
8
0
0
0
0
9
4
3


33
31
35
11
25
15
91
19
48
30
42
29
47
34
20
36
73
28
69
29
28
43
28
98
0
9
9
21
0
5
6
0
35
0
22
1
0
29
26
12
23
31
17
8
5
3
15
31
34
0
51
9
17
86
96
79
71
31
77
130
127
66
4
97
42
89
31
96
55
52
43
2
1
8
0
0
0
0
9
4
3


33
31
35
11
25
15
91
19
48
30
42
29
47
34
20
36
73
28
69
29
28
43
28
98
0
9
9
21
0
5
6
0
35
0
22
1
0
29
26
12
23
31
17
8
5
3
15
31
34
0
51
9
17
86
96
79
71
31
77
130
127
66
4
97
42
89
31
96
55
52
43
2
1
8
0
0
0
0
9
4
3


2127
2747
3896
678
1545
1131
2288
1671
2020
1204
1814
1629
2738
1642
1747
1623
2718
2343
1733
2003
1579
1848
2594
80
535
1798
425
1379
2128
2171
1037
69
169
273
1653
521
43
2154
1690
1018
1795
2498
2566
1927
1202
1188
1344
1926
1815
424
1641
640
1134
2295
2612
1755
1646
1487
2901
2813
2887
1944
287
1892
1251
2444
963
1889
1276
953
1318
664
516
889
145
532
143
223
691
518
464


1871
2132
3012
580
1122
920
1732
1341
1646
973
1299
1242
1671
1258
1336
1244
1991
1878
1264
1610
1368
1498
2173
80
86
1513
298
1081
939
2028
872
69
130
272
1555
494
43
1759
1394
875
1547
2156
2395
1789
1127
1082
1105
1660
1564
351
1303
510
978
1818
2053
1479
1338
1300
2456
2248
2430
1548
259
1433
1066
2029
898
1581
1118
856
1125
639
501
835
141
518
139
222
682
461
450


1871
2132
3012
580
1122
920
1732
1341
1646
973
1299
1242
1671
1258
1336
1244
1991
1878
1264
1610
1368
1498
2173
80
86
1513
298
1081
939
2028
872
69
130
272
1555
494
43
1759
1394
875
1547
2156
2395
1789
1127
1082
1105
1660
1564
351
1303
510
978
1818
2053
1479
1338
1300
2456
2248
2430
1548
259
1433
1066
2029
898
1581
1118
856
1125
639
501
835
141
518
139
222
682
461
450


1871
2132
3012
580
1122
920
1732
1341
1646
973
1299
1242
1671
1258
1336
1244
1991
1878
1264
1610
1368
1498
2173
80
86
1513
298
1081
939
2028
872
69
130
272
1555
494
43
1759
1394
875
1547
2156
2395
1789
1127
1082
1105
1660
1564
351
1303
510
978
1818
2053
1479
1338
1300
2456
2248
2430
1548
259
1433
1066
2029
898
1581
1118
856
1125
639
501
835
141
518
139
222
682
461
450


165
239
333
55
252
69
298
69
168
141
323
220
744
221
221
200
349
219
252
171
35
136
186
0
0
27
0
56
0
17
42
0
0
1
8
14
0
36
104
29
67
117
44
37
14
21
44
16
63
73
165
25
77
193
276
145
157
99
246
305
256
195
19
282
149
251
44
177
58
69
127
24
12
42
1
13
2
0
8
37
12


165
239
333
55
252
69
298
69
168
141
323
220
744
221
221
200
349
219
252
171
35
136
186
0
0
27
0
56
0
17
42
0
0
1
8
14
0
36
104
29
67
117
44
37
14
21
44
16
63
73
165
25
77
193
276
145
157
99
246
305
256
195
19
282
149
251
44
177
58
69
127
24
12
42
1
13
2
0
8
37
12


165
239
333
55
252
69
298
69
168
141
323
220
744
221
221
200
349
219
252
171
35
136
186
0
0
27
0
56
0
17
42
0
0
1
8
14
0
36
104
29
67
117
44
37
14
21
44
16
63
73
165
25
77
193
276
145
157
99
246
305
256
195
19
282
149
251
44
177
58
69
127
24
12
42
1
13
2
0
8
37
12


73
357
537
42
163
133
236
253
187
68
179
155
303
156
179
169
355
222
206
200
165
184
224
0
448
256
127
240
1188
121
116
0
39
0
89
13
0
355
186
108
166
198
110
92
56
82
181
243
172
0
147
101
64
262
260
117
105
77
166
231
183
171
9
160
24
122
7
120
9
20
47
0
0
10
1
1
1
1
0
12
0


73
357
537
42
163
133
236
253
187
68
179
155
303
156
179
169
355
222
206
200
165
184
224
0
448
256
127
240
1188
121
116
0
39
0
89
13
0
355
186
108
166
198
110
92
56
82
181
243
172
0
147
101
64
262
260
117
105
77
166
231
183
171
9
160
24
122
7
120
9
20
47
0
0
10
1
1
1
1
0
12
0


73
357
537
42
163
133
236
253
187
68
179
155
303
156
179
169
355
222
206
200
165
184
224
0
448
256
127
240
1188
121
116
0
39
0
89
13
0
355
186
108
166
198
110
92
56
82
181
243
172
0
147
101
64
262
260
117
105
77
166
231
183
171
9
160
24
122
7
120
9
20
47
0
0
10
1
1
1
1
0
12
0


18
19
14
1
8
9
22
8
19
22
13
12
20
7
11
10
23
24
11
22
11
30
11
0
1
2
0
2
1
5
7
0
0
0
1
0
0
4
6
6
15
27
17
9
5
3
14
7
16
0
26
4
15
22
23
14
46
11
33
29
18
30
0
17
12
42
14
11
91
8
19
1
3
2
2
0
1
0
1
8
2


18
19
14
1
8
9
22
8
19
22
13
12
20
7
11
10
23
24
11
22
11
30
11
0
1
2
0
2
1
5
7
0
0
0
1
0
0
4
6
6
15
27
17
9
5
3
14
7
16
0
26
4
15
22
23
14
46
11
33
29
18
30
0
17
12
42
14
11
91
8
19
1
3
2
2
0
1
0
1
8
2


18
19
14
1
8
9
22
8
19
22
13
12
20
7
11
10
23
24
11
22
11
30
11
0
1
2
0
2
1
5
7
0
0
0
1
0
0
4
6
6
15
27
17
9
5
3
14
7
16
0
26
4
15
22
23
14
46
11
33
29
18
30
0
17
12
42
14
11
91
8
19
1
3
2
2
0
1
0
1
8
2


70
85
84
10
56
27
79
43
58
34
70
57
67
46
46
54
75
108
53
57
39
68
94
0
1
26
0
11
1
29
21
0
0
0
6
12
0
14
35
34
64
64
47
16
14
15
39
20
56
1
37
11
20
52
52
36
63
46
61
118
64
42
11
43
12
41
28
43
12
2
33
0
4
5
0
3
1
7
1
3
1


70
85
84
10
56
27
79
43
58
34
70
57
67
46
46
54
75
108
53
57
39
68
94
0
1
26
0
11
1
29
21
0
0
0
6
12
0
14
35
34
64
64
47
16
14
15
39
20
56
1
37
11
20
52
52
36
63
46
61
118
64
42
11
43
12
41
28
43
12
2
33
0
4
5
0
3
1
7
1
3
1


70
85
84
10
56
27
79
43
58
34
70
57
67
46
46
54
75
108
53
57
39
68
94
0
1
26
0
11
1
29
21
0
0
0
6
12
0
14
35
34
64
64
47
16
14
15
39
20
56
1
37
11
20
52
52
36
63
46
61
118
64
42
11
43
12
41
28
43
12
2
33
0
4
5
0
3
1
7
1
3
1


70
85
84
10
56
27
79
43
58
34
70
57
67
46
46
54
75
108
53
57
39
68
94
0
1
26
0
11
1
29
21
0
0
0
6
12
0
14
35
34
64
64
47
16
14
15
39
20
56
1
37
11
20
52
52
36
63
46
61
118
64
42
11
43
12
41
28
43
12
2
33
0
4
5
0
3
1
7
1
3
1


795
55
27
3854
523
2631
193
515
187
195
37
168
11
90
46
74
40
58
10
125
42
44
30
0
0
53
70
62
122
637
825
1313
312
1120
410
2376
101
67
183
259
71
178
318
749
1197
1435
62
89
505
0
804
3556
3053
122
65
687
954
1577
364
20
500
1032
2849
25
3957
1338
3042
99
1836
2469
1382
6788
9032
5197
5777
7964
6181
5679
8201
3553
5029


795
55
27
3854
523
2631
193
515
187
195
37
168
11
90
46
74
40
58
10
125
42
44
30
0
0
53
70
62
122
637
825
1313
312
1120
410
2376
101
67
183
259
71
178
318
749
1197
1435
62
89
505
0
804
3556
3053
122
65
687
954
1577
364
20
500
1032
2849
25
3957
1338
3042
99
1836
2469
1382
6788
9032
5197
5777
7964
6181
5679
8201
3553
5029


0
0
0
0
0
8
0
0
0
0
0
0
0
0
0
0
0
0
0
0
0
0
0
0
0
0
0
0
0
0
0
0
0
0
0
0
0
0
0
0
0
0
0
0
0
2
0
0
0
0
0
0
6
0
0
0
0
0
0
0
0
0
5
0
0
0
3
0
4
0
0
0
0
0
0
0
0
0
0
0
0


0
0
0
0
0
8
0
0
0
0
0
0
0
0
0
0
0
0
0
0
0
0
0
0
0
0
0
0
0
0
0
0
0
0
0
0
0
0
0
0
0
0
0
0
0
2
0
0
0
0
0
0
6
0
0
0
0
0
0
0
0
0
5
0
0
0
3
0
4
0
0
0
0
0
0
0
0
0
0
0
0


0
0
0
0
0
8
0
0
0
0
0
0
0
0
0
0
0
0
0
0
0
0
0
0
0
0
0
0
0
0
0
0
0
0
0
0
0
0
0
0
0
0
0
0
0
2
0
0
0
0
0
0
6
0
0
0
0
0
0
0
0
0
5
0
0
0
3
0
4
0
0
0
0
0
0
0
0
0
0
0
0


17
0
0
260
19
270
3
4
24
18
5
75
1
15
5
10
21
1
4
18
1
1
2
0
0
5
0
1
0
11
32
661
312
376
2
784
1
2
5
47
19
12
6
57
134
19
1
6
40
0
7
254
86
3
7
42
43
38
14
2
14
2
292
0
112
25
54
0
34
128
81
730
1144
890
386
912
351
397
1041
493
356


2
0
0
145
5
145
1
2
14
7
0
45
1
8
3
7
11
0
0
7
0
0
2
0
0
0
0
0
0
0
18
442
299
261
0
485
0
1
3
37
7
3
1
23
70
12
1
1
21
0
3
85
48
1
1
7
16
19
4
1
4
1
264
0
71
15
17
0
11
6
48
395
651
581
236
389
231
219
598
256
213


2
0
0
145
5
145
1
2
14
7
0
45
1
8
3
7
11
0
0
7
0
0
2
0
0
0
0
0
0
0
18
442
299
261
0
485
0
1
3
37
7
3
1
23
70
12
1
1
21
0
3
85
48
1
1
7
16
19
4
1
4
1
264
0
71
15
17
0
11
6
48
395
651
581
236
389
231
219
598
256
213


0
0
0
0
0
0
0
0
0
0
0
1
0
0
0
0
0
0
0
0
0
0
0
0
0
0
0
0
0
0
0
0
0
0
0
0
0
0
0
0
0
0
0
0
0
0
0
0
0
0
0
0
0
0
0
0
0
0
0
0
0
0
0
0
0
0
1
0
0
0
0
0
0
0
0
1
0
0
0
0
0


0
0
0
0
0
0
0
0
0
0
0
1
0
0
0
0
0
0
0
0
0
0
0
0
0
0
0
0
0
0
0
0
0
0
0
0
0
0
0
0
0
0
0
0
0
0
0
0
0
0
0
0
0
0
0
0
0
0
0
0
0
0
0
0
0
0
1
0
0
0
0
0
0
0
0
1
0
0
0
0
0


15
0
0
81
13
78
2
2
8
8
4
9
0
4
1
3
5
0
4
9
0
0
0
0
0
0
0
1
0
11
12
216
0
97
0
226
0
1
2
5
1
8
5
29
56
7
0
0
14
0
4
163
32
2
4
32
24
16
7
0
10
1
8
0
35
10
29
0
16
115
25
289
385
243
92
483
72
139
388
182
98


0
0
0
2
7
25
0
0
0
0
0
1
0
1
0
1
0
0
0
1
0
0
0
0
0
0
0
1
0
1
0
1
0
1
0
3
0
1
0
2
1
1
0
6
4
0
0
0
0
0
1
86
8
0
0
4
16
2
2
0
5
0
1
0
26
0
15
0
13
113
20
246
314
175
70
393
50
90
284
149
72


0
0
0
1
0
3
1
0
1
0
0
0
0
2
0
0
0
0
0
0
0
0
0
0
0
0
0
0
0
1
1
8
0
0
0
0
0
0
0
0
0
0
0
2
3
0
0
0
0
0
1
0
1
0
0
3
0
3
1
0
0
1
0
0
0
1
11
0
3
0
2
5
6
14
2
5
3
4
3
3
1


15
0
0
78
6
50
1
2
7
8
4
8
0
1
1
2
5
0
4
8
0
0
0
0
0
0
0
0
0
9
11
207
0
96
0
223
0
0
2
3
0
7
5
21
49
7
0
0
14
0
2
77
23
2
4
25
8
11
4
0
5
0
7
0
9
9
3
0
0
2
3
38
65
54
20
85
19
45
101
30
25


0
0
0
3
1
2
0
0
0
0
0
0
0
0
0
0
0
0
0
0
0
1
0
0
0
5
0
0
0
0
0
0
0
0
2
1
0
0
0
1
11
0
0
2
0
0
0
5
1
0
0
0
2
0
2
2
1
0
2
0
0
0
0
0
0
0
5
0
4
5
4
5
13
1
6
7
0
7
14
1
2


0
0
0
3
1
2
0
0
0
0
0
0
0
0
0
0
0
0
0
0
0
1
0
0
0
5
0
0
0
0
0
0
0
0
2
1
0
0
0
1
11
0
0
2
0
0
0
5
1
0
0
0
2
0
2
2
1
0
2
0
0
0
0
0
0
0
5
0
4
5
4
5
13
1
6
7
0
7
14
1
2


0
0
0
31
0
45
0
0
2
3
1
20
0
3
1
0
5
1
0
2
1
0
0
0
0
0
0
0
0
0
2
3
13
18
0
72
1
0
0
4
0
1
0
3
8
0
0
0
4
0
0
6
4
0
0
1
2
3
1
1
0
0
20
0
6
0
2
0
3
2
4
41
95
65
52
32
48
32
41
54
43


0
0
0
31
0
45
0
0
2
3
1
20
0
3
1
0
5
1
0
2
1
0
0
0
0
0
0
0
0
0
2
3
13
18
0
72
1
0
0
4
0
1
0
3
8
0
0
0
4
0
0
6
4
0
0
1
2
3
1
1
0
0
20
0
6
0
2
0
3
2
4
41
95
65
52
32
48
32
41
54
43


777
54
27
3582
502
2170
190
510
162
176
32
93
10
75
39
63
18
55
6
106
40
43
28
0
0
48
70
61
122
624
792
651
0
744
408
1574
100
65
178
212
49
165
312
689
1061
1407
61
83
465
0
797
3283
2924
119
58
645
902
1518
350
18
485
1030
2465
25
3822
1311
2980
99
1798
2341
1292
6043
7849
4290
5301
7034
5738
5221
7130
3047
4642


528
17
10
1929
337
1670
118
341
91
122
11
51
1
42
27
39
8
18
1
61
21
26
17
0
0
33
0
16
121
501
568
545
0
632
324
520
100
17
112
83
32
118
245
490
638
982
22
19
241
0
66
2694
2542
77
27
309
645
1279
250
12
370
83
2278
12
2886
1091
2716
54
1715
2086
971
5088
6936
3718
4851
6068
4869
4696
6044
2615
3947


528
17
10
1929
337
1670
118
341
91
122
11
51
1
42
27
39
8
18
1
61
21
26
17
0
0
33
0
16
121
501
568
545
0
632
324
520
100
17
112
83
32
118
245
490
638
982
22
19
241
0
66
2694
2542
77
27
309
645
1279
250
12
370
83
2278
12
2886
1091
2716
54
1715
2086
971
5088
6936
3718
4851
6068
4869
4696
6044
2615
3947


202
18
6
1335
109
340
38
81
32
39
10
37
4
20
7
10
7
35
4
18
15
12
6
0
0
15
0
31
0
85
207
103
0
106
48
1015
0
38
60
123
14
40
37
156
351
287
36
49
172
0
726
485
291
37
24
312
221
200
88
4
87
935
147
9
831
181
216
27
64
236
274
828
655
474
279
849
473
286
895
348
422


173
15
6
1260
71
312
17
49
31
28
7
23
3
18
4
5
7
35
3
12
14
12
6
0
0
15
0
26
0
59
188
102
0
89
46
943
0
38
58
116
11
35
37
147
334
250
36
49
120
0
707
457
262
34
24
303
207
179
84
4
80
907
126
9
755
136
205
25
36
224
246
552
450
307
216
686
276
199
703
311
333


1
0
0
0
1
0
0
0
0
0
0
0
0
0
0
0
0
0
0
0
0
0
0
0
0
0
0
0
0
1
2
0
0
0
0
0
0
0
0
0
0
0
0
0
0
0
0
0
0
0
0
0
1
0
0
0
0
3
0
0
0
0
1
0
0
0
0
0
0
0
3
2
12
16
3
0
5
7
10
1
2


28
3
0
75
37
28
21
32
1
11
3
14
1
2
3
5
0
0
1
6
1
0
0
0
0
0
0
5
0
25
17
1
0
17
2
72
0
0
2
7
3
5
0
9
17
37
0
0
52
0
19
28
28
3
0
9
14
18
4
0
7
28
20
0
76
45
11
2
28
12
25
274
193
151
60
163
192
80
182
36
87


47
19
11
318
56
160
34
88
39
15
11
5
5
13
5
14
3
2
1
27
4
5
5
0
0
0
70
14
1
38
17
3
0
6
36
39
0
10
6
6
3
7
30
43
72
138
3
15
52
0
5
104
91
5
7
24
36
39
12
2
28
12
40
4
105
39
48
18
19
19
47
127
258
98
171
117
396
239
191
84
273


47
19
11
318
56
160
34
88
39
15
11
5
5
13
5
14
3
2
1
27
4
5
5
0
0
0
70
14
1
38
17
3
0
6
36
39
0
10
6
6
3
7
30
43
72
138
3
15
52
0
5
104
91
5
7
24
36
39
12
2
28
12
40
4
105
39
48
18
19
19
47
127
258
98
171
117
396
239
191
84
273


1
1
0
12
2
183
0
1
1
1
0
0
0
0
2
1
1
2
0
1
1
0
0
0
0
0
0
0
0
2
1
1
0
0
0
18
0
0
0
0
3
1
0
3
2
7
0
0
0
0
0
19
37
0
0
0
9
21
0
0
1
0
87
0
23
2
5
0
0
0
9
15
39
17
90
18
92
61
30
13
31


1
1
0
12
2
183
0
1
1
1
0
0
0
0
2
1
1
2
0
1
1
0
0
0
0
0
0
0
0
2
1
1
0
0
0
18
0
0
0
0
3
1
0
3
2
7
0
0
0
0
0
19
37
0
0
0
9
21
0
0
1
0
87
0
23
2
5
0
0
0
9
15
39
17
90
18
92
61
30
13
31


1
1
0
12
2
183
0
1
1
1
0
0
0
0
2
1
1
2
0
1
1
0
0
0
0
0
0
0
0
2
1
1
0
0
0
18
0
0
0
0
3
1
0
3
2
7
0
0
0
0
0
19
37
0
0
0
9
21
0
0
1
0
87
0
23
2
5
0
0
0
9
15
39
17
90
18
92
61
30
13
31


23
11
9
1
8
3
15
13
20
17
14
10
19
12
14
10
20
17
15
12
18
11
13
0
0
4
0
2
0
3
3
0
0
0
16
1
0
0
1
1
1
11
26
10
4
2
0
3
9
0
15
0
10
19
20
10
6
7
15
13
25
10
0
12
7
16
2
19
8
0
3
0
0
2
0
0
0
0
1
0
0


13
10
9
1
3
1
9
10
15
5
11
6
12
7
7
5
9
9
6
7
1
5
7
0
0
0
0
0
0
0
0
0
0
0
6
0
0
0
0
0
0
3
1
3
0
1
0
0
3
0
10
0
8
12
8
7
5
2
6
6
3
5
0
9
0
4
1
7
3
0
2
0
0
2
0
0
0
0
1
0
0


13
10
9
1
3
1
9
10
15
5
11
6
12
7
7
5
9
9
6
7
1
5
7
0
0
0
0
0
0
0
0
0
0
0
6
0
0
0
0
0
0
3
1
3
0
1
0
0
3
0
10
0
8
12
8
7
5
2
6
6
3
5
0
9
0
4
1
7
3
0
2
0
0
2
0
0
0
0
1
0
0


13
10
9
1
3
1
9
10
15
5
11
6
12
7
7
5
9
9
6
7
1
5
7
0
0
0
0
0
0
0
0
0
0
0
6
0
0
0
0
0
0
3
1
3
0
1
0
0
3
0
10
0
8
12
8
7
5
2
6
6
3
5
0
9
0
4
1
7
3
0
2
0
0
2
0
0
0
0
1
0
0


13
10
9
1
3
1
9
10
15
5
11
6
12
7
7
5
9
9
6
7
1
5
7
0
0
0
0
0
0
0
0
0
0
0
6
0
0
0
0
0
0
3
1
3
0
1
0
0
3
0
10
0
8
12
8
7
5
2
6
6
3
5
0
9
0
4
1
7
3
0
2
0
0
2
0
0
0
0
1
0
0


10
1
0
0
5
2
6
3
5
12
3
4
7
5
7
5
11
8
8
5
17
6
6
0
0
4
0
2
0
3
3
0
0
0
10
1
0
0
1
1
1
8
25
7
4
1
0
3
6
0
5
0
2
7
12
3
1
5
9
7
22
5
0
3
7
12
1
12
5
0
1
0
0
0
0
0
0
0
0
0
0


10
1
0
0
5
2
6
3
5
12
3
4
7
5
7
5
11
8
8
5
17
6
6
0
0
4
0
2
0
3
3
0
0
0
10
1
0
0
1
1
1
8
25
7
4
1
0
3
6
0
5
0
2
7
12
3
1
5
9
7
22
5
0
3
7
12
1
12
5
0
1
0
0
0
0
0
0
0
0
0
0


10
1
0
0
5
2
6
3
5
12
3
4
7
5
7
5
11
8
8
5
17
6
6
0
0
4
0
2
0
3
3
0
0
0
10
1
0
0
1
1
1
8
25
7
4
1
0
3
6
0
5
0
2
7
12
3
1
5
9
7
22
5
0
3
7
12
1
12
5
0
1
0
0
0
0
0
0
0
0
0
0


10
1
0
0
5
2
6
3
5
12
3
4
7
5
7
5
11
8
8
5
17
6
6
0
0
4
0
2
0
3
3
0
0
0
10
1
0
0
1
1
1
8
25
7
4
1
0
3
6
0
5
0
2
7
12
3
1
5
9
7
22
5
0
3
7
12
1
12
5
0
1
0
0
0
0
0
0
0
0
0
0


0
0
0
0
0
0
0
0
0
0
0
0
0
0
0
0
0
0
1
0
0
0
0
0
0
0
0
0
0
0
0
0
0
0
0
0
0
0
0
0
0
0
0
0
0
0
0
0
0
0
0
0
0
0
0
0
0
0
0
0
0
0
0
0
0
0
0
0
0
0
0
0
0
0
0
0
0
0
0
0
0


0
0
0
0
0
0
0
0
0
0
0
0
0
0
0
0
0
0
1
0
0
0
0
0
0
0
0
0
0
0
0
0
0
0
0
0
0
0
0
0
0
0
0
0
0
0
0
0
0
0
0
0
0
0
0
0
0
0
0
0
0
0
0
0
0
0
0
0
0
0
0
0
0
0
0
0
0
0
0
0
0


0
0
0
0
0
0
0
0
0
0
0
0
0
0
0
0
0
0
1
0
0
0
0
0
0
0
0
0
0
0
0
0
0
0
0
0
0
0
0
0
0
0
0
0
0
0
0
0
0
0
0
0
0
0
0
0
0
0
0
0
0
0
0
0
0
0
0
0
0
0
0
0
0
0
0
0
0
0
0
0
0


0
0
0
0
0
0
0
0
0
0
0
0
0
0
0
0
0
0
1
0
0
0
0
0
0
0
0
0
0
0
0
0
0
0
0
0
0
0
0
0
0
0
0
0
0
0
0
0
0
0
0
0
0
0
0
0
0
0
0
0
0
0
0
0
0
0
0
0
0
0
0
0
0
0
0
0
0
0
0
0
0


1939
2868
2366
1041
840
987
1538
2945
2407
1291
1386
1307
1168
3496
1968
1332
1735
4176
1077
2335
2191
1188
1588
0
380
1042
0
1547
456
3403
1520
48
85
139
2884
365
84
3090
3704
1048
1048
1813
1604
1834
1482
1171
1390
2276
3554
685
2523
521
494
2482
2147
3576
2561
2721
3309
2965
3347
2570
194
2913
650
2063
536
3033
1157
423
1032
219
400
347
71
180
128
155
209
510
221


49
4
2
20
20
16
13
21
10
9
1
11
0
5
7
1
0
10
4
27
20
5
12
0
0
24
0
14
0
6
5
0
0
0
3
7
0
24
31
30
25
20
9
5
13
17
27
11
31
0
6
7
7
9
0
8
9
19
3
3
15
10
2
5
8
9
8
19
10
12
9
11
8
6
4
5
20
2
5
1
4


49
4
2
20
20
16
13
21
10
9
1
11
0
5
7
1
0
10
4
27
20
5
12
0
0
24
0
14
0
6
5
0
0
0
3
7
0
24
31
30
25
20
9
5
13
17
27
11
31
0
6
7
7
9
0
8
9
19
3
3
15
10
2
5
8
9
8
19
10
12
9
11
8
6
4
5
20
2
5
1
4


49
4
2
20
20
16
13
21
10
9
1
11
0
5
7
1
0
10
4
27
20
5
12
0
0
24
0
14
0
6
5
0
0
0
3
7
0
24
31
30
25
20
9
5
13
17
27
11
31
0
6
7
7
9
0
8
9
19
3
3
15
10
2
5
8
9
8
19
10
12
9
11
8
6
4
5
20
2
5
1
4


49
4
2
20
20
16
13
21
10
9
1
11
0
5
7
1
0
10
4
27
20
5
12
0
0
24
0
14
0
6
5
0
0
0
3
7
0
24
31
30
25
20
9
5
13
17
27
11
31
0
6
7
7
9
0
8
9
19
3
3
15
10
2
5
8
9
8
19
10
12
9
11
8
6
4
5
20
2
5
1
4


90
32
8
19
10
32
31
71
46
6
12
19
6
12
148
40
20
87
12
43
40
60
52
0
0
22
0
102
0
139
37
0
19
3
234
36
0
201
104
44
75
85
91
72
56
45
84
243
172
0
235
42
19
57
232
217
160
136
157
140
356
290
29
160
81
287
67
310
19
127
74
19
15
24
0
56
2
5
32
20
14


90
32
8
19
10
32
31
71
46
6
12
19
6
12
148
40
20
87
12
43
40
60
52
0
0
22
0
102
0
139
37
0
19
3
234
36
0
201
104
44
75
85
91
72
56
45
84
243
172
0
235
42
19
57
232
217
160
136
157
140
356
290
29
160
81
287
67
310
19
127
74
19
15
24
0
56
2
5
32
20
14


90
32
8
19
10
32
31
71
46
6
12
19
6
12
148
40
20
87
12
43
40
60
52
0
0
22
0
102
0
139
37
0
19
3
234
36
0
201
104
44
75
85
91
72
56
45
84
243
172
0
235
42
19
57
232
217
160
136
157
140
356
290
29
160
81
287
67
310
19
127
74
19
15
24
0
56
2
5
32
20
14


90
32
8
19
10
32
31
71
46
6
12
19
6
12
148
40
20
87
12
43
40
60
52
0
0
22
0
102
0
139
37
0
19
3
234
36
0
201
104
44
75
85
91
72
56
45
84
243
172
0
235
42
19
57
232
217
160
136
157
140
356
290
29
160
81
287
67
310
19
127
74
19
15
24
0
56
2
5
32
20
14


23
12
2
5
8
5
21
26
75
9
79
57
117
12
7
12
16
46
11
32
3
26
20
0
0
6
0
3
0
0
0
0
0
0
1
0
0
5
9
18
3
11
2
6
1
1
4
3
7
0
30
1
7
9
11
14
6
10
15
4
24
24
1
3
1
11
1
13
3
5
5
0
0
1
1
1
0
0
3
1
0


23
12
2
5
8
5
21
26
75
9
79
57
117
12
7
12
16
46
11
32
3
26
20
0
0
6
0
3
0
0
0
0
0
0
1
0
0
5
9
18
3
11
2
6
1
1
4
3
7
0
30
1
7
9
11
14
6
10
15
4
24
24
1
3
1
11
1
13
3
5
5
0
0
1
1
1
0
0
3
1
0


23
12
2
5
8
5
21
26
75
9
79
57
117
12
7
12
16
46
11
32
3
26
20
0
0
6
0
3
0
0
0
0
0
0
1
0
0
5
9
18
3
11
2
6
1
1
4
3
7
0
30
1
7
9
11
14
6
10
15
4
24
24
1
3
1
11
1
13
3
5
5
0
0
1
1
1
0
0
3
1
0


23
12
2
5
8
5
21
26
75
9
79
57
117
12
7
12
16
46
11
32
3
26
20
0
0
6
0
3
0
0
0
0
0
0
1
0
0
5
9
18
3
11
2
6
1
1
4
3
7
0
30
1
7
9
11
14
6
10
15
4
24
24
1
3
1
11
1
13
3
5
5
0
0
1
1
1
0
0
3
1
0


0
0
0
12
0
0
0
0
0
0
0
3
0
0
0
0
0
1
0
0
0
0
1
0
0
0
0
0
0
4
3
0
0
0
0
0
0
0
6
0
0
0
3
0
3
3
0
6
4
0
0
4
6
3
0
2
1
7
0
0
1
0
0
0
15
12
6
0
2
2
0
11
10
5
3
6
5
4
4
1
5


0
0
0
12
0
0
0
0
0
0
0
3
0
0
0
0
0
1
0
0
0
0
1
0
0
0
0
0
0
4
3
0
0
0
0
0
0
0
6
0
0
0
3
0
3
3
0
6
4
0
0
4
6
3
0
2
1
7
0
0
1
0
0
0
15
12
6
0
2
2
0
11
10
5
3
6
5
4
4
1
5


0
0
0
12
0
0
0
0
0
0
0
3
0
0
0
0
0
1
0
0
0
0
1
0
0
0
0
0
0
4
3
0
0
0
0
0
0
0
6
0
0
0
3
0
3
3
0
6
4
0
0
4
6
3
0
2
1
7
0
0
1
0
0
0
15
12
6
0
2
2
0
11
10
5
3
6
5
4
4
1
5


0
0
0
12
0
0
0
0
0
0
0
3
0
0
0
0
0
1
0
0
0
0
1
0
0
0
0
0
0
4
3
0
0
0
0
0
0
0
6
0
0
0
3
0
3
3
0
6
4
0
0
4
6
3
0
2
1
7
0
0
1
0
0
0
15
12
6
0
2
2
0
11
10
5
3
6
5
4
4
1
5


71
152
35
14
35
15
115
91
153
34
42
41
34
40
54
80
97
103
33
253
74
111
127
0
0
57
0
127
0
105
105
0
31
55
165
32
0
151
135
85
87
180
189
197
279
294
119
133
230
163
234
47
71
85
317
240
152
154
200
166
300
230
19
240
114
193
50
301
46
32
123
66
53
34
6
26
9
8
22
20
14


71
152
35
14
35
15
115
91
153
34
42
41
34
40
54
80
97
103
33
253
74
111
127
0
0
57
0
127
0
105
105
0
31
55
165
32
0
151
135
85
87
180
189
197
279
294
119
133
230
163
234
47
71
85
317
240
152
154
200
166
300
230
19
240
114
193
50
301
46
32
123
66
53
34
6
26
9
8
22
20
14


71
152
35
14
35
15
115
91
153
34
42
41
34
40
54
80
97
103
33
253
74
111
127
0
0
57
0
127
0
105
105
0
31
55
165
32
0
151
135
85
87
180
189
197
279
294
119
133
230
163
234
47
71
85
317
240
152
154
200
166
300
230
19
240
114
193
50
301
46
32
123
66
53
34
6
26
9
8
22
20
14


71
152
35
14
35
15
115
91
153
34
42
41
34
40
54
80
97
103
33
253
74
111
127
0
0
57
0
127
0
105
105
0
31
55
165
32
0
151
135
85
87
180
189
197
279
294
119
133
230
163
234
47
71
85
317
240
152
154
200
166
300
230
19
240
114
193
50
301
46
32
123
66
53
34
6
26
9
8
22
20
14


1706
2668
2319
970
765
919
1357
2735
2122
1231
1252
1176
1011
3427
1749
1196
1601
3929
1015
1977
2052
985
1375
0
380
933
0
1301
456
3147
1369
48
35
81
2481
290
84
2709
3414
870
858
1517
1293
1553
1129
811
1155
1880
3109
522
2018
420
383
2319
1585
3088
2228
2390
2928
2648
2649
2015
143
2502
430
1550
404
2389
1077
244
820
112
313
277
56
86
92
136
143
467
184


1454
2103
2071
861
523
710
1146
2621
1971
1105
954
1005
531
2011
1404
857
1435
3685
676
1646
1498
775
915
0
128
629
0
1141
264
2971
990
48
35
81
2428
131
84
2262
3052
687
725
1278
1209
1322
997
675
1030
1754
2803
522
1878
271
248
1809
1326
2701
1926
2148
2795
2117
2378
1864
62
2281
284
1236
372
1969
984
195
644
40
160
74
6
38
78
98
12
276
92


1454
2103
2071
861
523
710
1146
2621
1971
1105
954
1005
531
2011
1404
857
1435
3685
676
1646
1498
775
915
0
128
629
0
1141
264
2971
990
48
35
81
2428
131
84
2262
3052
687
725
1278
1209
1322
997
675
1030
1754
2803
522
1878
271
248
1809
1326
2701
1926
2148
2795
2117
2378
1864
62
2281
284
1236
372
1969
984
195
644
40
160
74
6
38
78
98
12
276
92


1454
2103
2071
861
523
710
1146
2621
1971
1105
954
1005
531
2011
1404
857
1435
3685
676
1646
1498
775
915
0
128
629
0
1141
264
2971
990
48
35
81
2428
131
84
2262
3052
687
725
1278
1209
1322
997
675
1030
1754
2803
522
1878
271
248
1809
1326
2701
1926
2148
2795
2117
2378
1864
62
2281
284
1236
372
1969
984
195
644
40
160
74
6
38
78
98
12
276
92


243
557
244
108
242
208
211
108
150
125
292
164
477
1403
343
317
163
239
335
323
544
206
454
0
252
295
0
152
192
175
373
0
0
0
53
158
0
445
354
180
130
234
79
225
132
135
121
126
300
0
140
148
134
510
253
382
295
234
125
517
264
150
81
214
145
308
32
418
92
49
173
72
153
203
50
48
14
38
131
191
92


243
557
244
108
242
208
211
108
150
125
292
164
477
1403
343
317
163
239
335
323
544
206
454
0
252
295
0
152
192
175
373
0
0
0
53
158
0
445
354
180
130
234
79
225
132
135
121
126
300
0
140
148
134
510
253
382
295
234
125
517
264
150
81
214
145
308
32
418
92
49
173
72
153
203
50
48
14
38
131
191
92


243
557
244
108
242
208
211
108
150
125
292
164
477
1403
343
317
163
239
335
323
544
206
454
0
252
295
0
152
192
175
373
0
0
0
53
158
0
445
354
180
130
234
79
225
132
135
121
126
300
0
140
148
134
510
253
382
295
234
125
517
264
150
81
214
145
308
32
418
92
49
173
72
153
203
50
48
14
38
131
191
92


9
8
4
1
0
1
0
6
1
1
6
7
3
13
2
22
3
5
4
8
10
4
6
0
0
9
0
8
0
1
6
0
0
0
0
1
0
2
8
3
3
5
5
6
0
1
4
0
6
0
0
1
1
0
6
5
7
8
8
14
7
1
0
7
1
6
0
2
1
0
3
0
0
0
0
0
0
0
0
0
0


9
8
4
1
0
1
0
6
1
1
6
7
3
13
2
22
3
5
4
8
10
4
6
0
0
9
0
8
0
1
6
0
0
0
0
1
0
2
8
3
3
5
5
6
0
1
4
0
6
0
0
1
1
0
6
5
7
8
8
14
7
1
0
7
1
6
0
2
1
0
3
0
0
0
0
0
0
0
0
0
0


9
8
4
1
0
1
0
6
1
1
6
7
3
13
2
22
3
5
4
8
10
4
6
0
0
9
0
8
0
1
6
0
0
0
0
1
0
2
8
3
3
5
5
6
0
1
4
0
6
0
0
1
1
0
6
5
7
8
8
14
7
1
0
7
1
6
0
2
1
0
3
0
0
0
0
0
0
0
0
0
0


0
0
0
1
2
0
1
1
1
2
0
0
0
0
3
3
1
0
2
3
2
1
1
0
0
0
0
0
0
2
1
0
0
0
0
0
0
0
5
1
0
0
17
1
1
0
1
0
1
0
0
0
1
0
2
7
5
5
6
4
2
1
0
3
1
1
0
1
0
1
1
0
1
0
1
0
0
0
0
0
0


0
0
0
1
2
0
1
1
1
2
0
0
0
0
3
3
1
0
2
3
2
1
1
0
0
0
0
0
0
2
1
0
0
0
0
0
0
0
5
1
0
0
17
1
1
0
1
0
1
0
0
0
1
0
2
7
5
5
6
4
2
1
0
3
1
1
0
1
0
1
1
0
1
0
1
0
0
0
0
0
0


0
0
0
1
2
0
1
1
1
2
0
0
0
0
3
3
1
0
2
3
2
1
1
0
0
0
0
0
0
2
1
0
0
0
0
0
0
0
5
1
0
0
17
1
1
0
1
0
1
0
0
0
1
0
2
7
5
5
6
4
2
1
0
3
1
1
0
1
0
1
1
0
1
0
1
0
0
0
0
0
0


0
0
0
1
2
0
1
1
1
2
0
0
0
0
3
3
1
0
2
3
2
1
1
0
0
0
0
0
0
2
1
0
0
0
0
0
0
0
5
1
0
0
17
1
1
0
1
0
1
0
0
0
1
0
2
7
5
5
6
4
2
1
0
3
1
1
0
1
0
1
1
0
1
0
1
0
0
0
0
0
0


72
7
5
727
184
1557
81
228
84
110
8
34
8
21
7
7
6
9
7
21
3
2
6
0
0
6
0
0
0
396
300
10
0
281
229
675
0
0
131
141
15
85
87
471
520
272
3
0
84
0
81
2333
3173
55
43
216
851
440
115
8
146
104
832
29
889
338
3907
87
3309
3580
1093
953
1332
663
278
1950
204
468
1492
292
587


72
7
5
727
184
1556
81
228
84
110
8
34
8
21
7
7
6
9
7
21
3
2
6
0
0
6
0
0
0
396
300
10
0
281
229
675
0
0
131
141
15
85
87
471
520
272
3
0
84
0
81
2333
3172
55
43
216
851
440
115
8
146
104
832
29
889
338
3906
87
3309
3580
1092
953
1332
663
278
1950
204
468
1492
292
587


72
7
5
727
184
1556
81
228
84
110
8
34
8
21
7
7
6
9
7
21
3
2
6
0
0
6
0
0
0
396
300
10
0
281
229
675
0
0
131
141
15
85
87
471
520
272
3
0
84
0
81
2333
3172
55
43
216
851
440
115
8
146
104
832
29
889
338
3906
87
3309
3580
1092
953
1332
663
278
1950
204
468
1492
292
587


72
7
5
727
184
1556
81
228
84
110
8
34
8
21
7
7
6
9
7
21
3
2
6
0
0
6
0
0
0
396
300
10
0
281
229
675
0
0
131
141
15
85
87
471
520
272
3
0
84
0
81
2333
3172
55
43
216
851
440
115
8
146
104
832
29
889
338
3906
87
3309
3580
1092
953
1332
663
278
1950
204
468
1492
292
587


72
7
5
727
184
1556
81
228
84
110
8
34
8
21
7
7
6
9
7
21
3
2
6
0
0
6
0
0
0
396
300
10
0
281
229
675
0
0
131
141
15
85
87
471
520
272
3
0
84
0
81
2333
3172
55
43
216
851
440
115
8
146
104
832
29
889
338
3906
87
3309
3580
1092
953
1332
663
278
1950
204
468
1492
292
587


0
0
0
0
0
1
0
0
0
0
0
0
0
0
0
0
0
0
0
0
0
0
0
0
0
0
0
0
0
0
0
0
0
0
0
0
0
0
0
0
0
0
0
0
0
0
0
0
0
0
0
0
1
0
0
0
0
0
0
0
0
0
0
0
0
0
1
0
0
0
1
0
0
0
0
0
0
0
0
0
0


0
0
0
0
0
1
0
0
0
0
0
0
0
0
0
0
0
0
0
0
0
0
0
0
0
0
0
0
0
0
0
0
0
0
0
0
0
0
0
0
0
0
0
0
0
0
0
0
0
0
0
0
1
0
0
0
0
0
0
0
0
0
0
0
0
0
1
0
0
0
1
0
0
0
0
0
0
0
0
0
0


0
0
0
0
0
1
0
0
0
0
0
0
0
0
0
0
0
0
0
0
0
0
0
0
0
0
0
0
0
0
0
0
0
0
0
0
0
0
0
0
0
0
0
0
0
0
0
0
0
0
0
0
1
0
0
0
0
0
0
0
0
0
0
0
0
0
1
0
0
0
1
0
0
0
0
0
0
0
0
0
0


0
0
0
0
0
1
0
0
0
0
0
0
0
0
0
0
0
0
0
0
0
0
0
0
0
0
0
0
0
0
0
0
0
0
0
0
0
0
0
0
0
0
0
0
0
0
0
0
0
0
0
0
1
0
0
0
0
0
0
0
0
0
0
0
0
0
1
0
0
0
1
0
0
0
0
0
0
0
0
0
0


9
2
8
5
5
7
2
7
4
3
16
9
11
34
5
2
9
8
11
4
4
4
12
0
0
0
0
2
0
0
0
0
0
0
0
5
0
11
12
1
2
1
5
4
2
1
8
0
3
0
4
3
14
10
2
6
27
19
9
14
14
8
3
22
31
21
13
7
20
12
24
6
10
6
4
9
1
2
3
5
8


9
2
8
5
5
7
2
7
4
3
16
9
11
34
5
2
9
8
11
4
4
4
12
0
0
0
0
2
0
0
0
0
0
0
0
5
0
11
12
1
2
1
5
4
2
1
8
0
3
0
4
3
14
10
2
6
27
19
9
14
14
8
3
22
31
21
13
7
20
12
24
6
10
6
4
9
1
2
3
5
8


9
2
8
5
5
7
2
7
4
3
16
9
11
34
5
2
9
8
11
4
4
4
12
0
0
0
0
2
0
0
0
0
0
0
0
5
0
11
12
1
2
1
5
4
2
1
8
0
3
0
4
3
14
10
2
6
27
19
9
14
14
8
3
22
31
21
13
7
20
12
24
6
10
6
4
9
1
2
3
5
8


9
2
8
5
5
7
2
7
4
3
16
9
11
34
5
2
9
8
11
4
4
4
12
0
0
0
0
2
0
0
0
0
0
0
0
5
0
11
12
1
2
1
5
4
2
1
8
0
3
0
4
3
14
10
2
6
27
19
9
14
14
8
3
22
31
21
13
7
20
12
24
6
10
6
4
9
1
2
3
5
8


9
2
8
5
5
7
2
7
4
3
16
9
11
34
5
2
9
8
11
4
4
4
12
0
0
0
0
2
0
0
0
0
0
0
0
5
0
11
12
1
2
1
5
4
2
1
8
0
3
0
4
3
14
10
2
6
27
19
9
14
14
8
3
22
31
21
13
7
20
12
24
6
10
6
4
9
1
2
3
5
8


0
0
0
2
0
1
1
1
2
4
0
6
0
4
0
0
0
0
0
0
0
0
0
0
0
0
0
0
0
0
0
0
1
0
0
0
0
0
0
0
0
0
0
0
0
0
0
0
0
0
0
0
0
0
0
1
1
0
0
0
0
0
78
0
2
0
0
0
0
0
0
1
1
15
0
0
0
0
2
2
1


0
0
0
2
0
1
1
1
2
4
0
6
0
4
0
0
0
0
0
0
0
0
0
0
0
0
0
0
0
0
0
0
1
0
0
0
0
0
0
0
0
0
0
0
0
0
0
0
0
0
0
0
0
0
0
1
1
0
0
0
0
0
78
0
2
0
0
0
0
0
0
1
1
15
0
0
0
0
2
2
1


0
0
0
0
0
0
0
0
0
0
0
0
0
0
0
0
0
0
0
0
0
0
0
0
0
0
0
0
0
0
0
0
0
0
0
0
0
0
0
0
0
0
0
0
0
0
0
0
0
0
0
0
0
0
0
0
0
0
0
0
0
0
1
0
0
0
0
0
0
0
0
0
0
0
0
0
0
0
0
0
0


0
0
0
0
0
0
0
0
0
0
0
0
0
0
0
0
0
0
0
0
0
0
0
0
0
0
0
0
0
0
0
0
0
0
0
0
0
0
0
0
0
0
0
0
0
0
0
0
0
0
0
0
0
0
0
0
0
0
0
0
0
0
1
0
0
0
0
0
0
0
0
0
0
0
0
0
0
0
0
0
0


0
0
0
0
0
0
0
0
0
0
0
0
0
0
0
0
0
0
0
0
0
0
0
0
0
0
0
0
0
0
0
0
0
0
0
0
0
0
0
0
0
0
0
0
0
0
0
0
0
0
0
0
0
0
0
0
0
0
0
0
0
0
1
0
0
0
0
0
0
0
0
0
0
0
0
0
0
0
0
0
0


0
0
0
2
0
1
1
1
2
4
0
6
0
4
0
0
0
0
0
0
0
0
0
0
0
0
0
0
0
0
0
0
1
0
0
0
0
0
0
0
0
0
0
0
0
0
0
0
0
0
0
0
0
0
0
1
1
0
0
0
0
0
77
0
2
0
0
0
0
0
0
1
1
15
0
0
0
0
2
2
1


0
0
0
2
0
1
1
0
1
1
0
3
0
4
0
0
0
0
0
0
0
0
0
0
0
0
0
0
0
0
0
0
1
0
0
0
0
0
0
0
0
0
0
0
0
0
0
0
0
0
0
0
0
0
0
1
1
0
0
0
0
0
77
0
0
0
0
0
0
0
0
1
1
12
0
0
0
0
1
2
0


0
0
0
2
0
1
1
0
1
1
0
3
0
4
0
0
0
0
0
0
0
0
0
0
0
0
0
0
0
0
0
0
1
0
0
0
0
0
0
0
0
0
0
0
0
0
0
0
0
0
0
0
0
0
0
1
1
0
0
0
0
0
77
0
0
0
0
0
0
0
0
1
1
12
0
0
0
0
1
2
0


0
0
0
0
0
0
0
0
0
0
0
0
0
0
0
0
0
0
0
0
0
0
0
0
0
0
0
0
0
0
0
0
0
0
0
0
0
0
0
0
0
0
0
0
0
0
0
0
0
0
0
0
0
0
0
0
0
0
0
0
0
0
0
0
0
0
0
0
0
0
0
0
0
3
0
0
0
0
0
0
0


0
0
0
0
0
0
0
0
0
0
0
0
0
0
0
0
0
0
0
0
0
0
0
0
0
0
0
0
0
0
0
0
0
0
0
0
0
0
0
0
0
0
0
0
0
0
0
0
0
0
0
0
0
0
0
0
0
0
0
0
0
0
0
0
0
0
0
0
0
0
0
0
0
3
0
0
0
0
0
0
0


0
0
0
0
0
0
0
1
1
3
0
3
0
0
0
0
0
0
0
0
0
0
0
0
0
0
0
0
0
0
0
0
0
0
0
0
0
0
0
0
0
0
0
0
0
0
0
0
0
0
0
0
0
0
0
0
0
0
0
0
0
0
0
0
2
0
0
0
0
0
0
0
0
0
0
0
0
0
1
0
1


0
0
0
0
0
0
0
1
1
3
0
3
0
0
0
0
0
0
0
0
0
0
0
0
0
0
0
0
0
0
0
0
0
0
0
0
0
0
0
0
0
0
0
0
0
0
0
0
0
0
0
0
0
0
0
0
0
0
0
0
0
0
0
0
2
0
0
0
0
0
0
0
0
0
0
0
0
0
1
0
1


34
6
4
5
20
5
124
66
102
51
39
58
44
136
19
17
15
6
36
26
4
7
9
0
0
10
0
1
0
10
2
0
0
0
47
3
0
0
9
2
4
24
49
24
21
3
1
0
7
0
93
1
13
47
135
84
22
19
86
40
155
124
1
120
14
24
20
200
48
0
35
0
0
0
0
0
0
0
0
0
1


34
6
4
5
20
5
124
66
102
51
39
58
44
136
19
17
15
6
36
26
4
7
9
0
0
10
0
1
0
10
2
0
0
0
47
3
0
0
9
2
4
24
49
24
21
3
1
0
7
0
93
1
13
47
135
84
22
19
86
40
155
124
1
120
14
24
20
200
48
0
35
0
0
0
0
0
0
0
0
0
1


34
6
4
5
20
5
124
66
102
51
39
58
44
136
19
17
15
6
36
26
4
7
9
0
0
10
0
1
0
10
2
0
0
0
47
3
0
0
9
2
4
24
49
24
21
3
1
0
7
0
93
1
13
47
135
84
22
19
86
40
155
124
1
120
14
24
20
200
48
0
35
0
0
0
0
0
0
0
0
0
1


34
6
4
5
20
5
124
66
102
51
39
58
44
136
19
17
15
6
36
26
4
7
9
0
0
10
0
1
0
10
2
0
0
0
47
3
0
0
9
2
4
24
49
24
21
3
1
0
7
0
93
1
13
47
135
84
22
19
86
40
155
124
1
120
14
24
20
200
48
0
35
0
0
0
0
0
0
0
0
0
1


34
6
4
5
20
5
124
66
102
51
39
58
44
136
19
17
15
6
36
26
4
7
9
0
0
10
0
1
0
10
2
0
0
0
47
3
0
0
9
2
4
24
49
24
21
3
1
0
7
0
93
1
13
47
135
84
22
19
86
40
155
124
1
120
14
24
20
200
48
0
35
0
0
0
0
0
0
0
0
0
1


78
2
0
0
3
0
143
68
13
45
16
48
43
68
6
29
23
1
46
18
0
3
4
0
0
0
0
0
0
0
0
0
0
0
0
0
0
0
0
0
1
10
0
3
0
2
0
0
1
0
5
0
2
20
76
18
5
6
44
83
13
7
1
87
14
24
1
23
73
0
24
0
0
0
0
0
0
0
0
0
0


78
2
0
0
3
0
143
68
13
45
16
48
43
68
6
29
23
1
46
18
0
3
4
0
0
0
0
0
0
0
0
0
0
0
0
0
0
0
0
0
1
10
0
3
0
2
0
0
1
0
5
0
2
20
76
18
5
6
44
83
13
7
1
87
14
24
1
23
73
0
24
0
0
0
0
0
0
0
0
0
0


78
2
0
0
3
0
143
68
13
45
16
48
43
68
6
29
23
1
46
18
0
3
4
0
0
0
0
0
0
0
0
0
0
0
0
0
0
0
0
0
1
10
0
3
0
2
0
0
1
0
5
0
2
20
76
18
5
6
44
83
13
7
1
87
14
24
1
23
73
0
24
0
0
0
0
0
0
0
0
0
0


78
2
0
0
3
0
143
68
13
45
16
48
43
68
6
29
23
1
46
18
0
3
4
0
0
0
0
0
0
0
0
0
0
0
0
0
0
0
0
0
1
10
0
3
0
2
0
0
1
0
5
0
2
20
76
18
5
6
44
83
13
7
1
87
14
24
1
23
73
0
24
0
0
0
0
0
0
0
0
0
0


78
2
0
0
3
0
143
68
13
45
16
48
43
68
6
29
23
1
46
18
0
3
4
0
0
0
0
0
0
0
0
0
0
0
0
0
0
0
0
0
1
10
0
3
0
2
0
0
1
0
5
0
2
20
76
18
5
6
44
83
13
7
1
87
14
24
1
23
73
0
24
0
0
0
0
0
0
0
0
0
0


3
0
1
1
0
1
9
47
7
1
0
0
0
0
0
0
0
1
0
0
3
6
6
0
0
0
0
0
0
6
9
0
0
0
32
0
0
0
0
0
2
19
58
12
12
8
2
2
5
0
2
0
0
1
2
43
2
6
12
0
19
7
0
12
3
6
0
14
3
13
8
0
0
0
0
0
0
0
0
0
0


3
0
1
1
0
1
9
47
7
1
0
0
0
0
0
0
0
1
0
0
3
6
6
0
0
0
0
0
0
6
9
0
0
0
32
0
0
0
0
0
2
19
58
12
12
8
2
2
5
0
2
0
0
1
2
43
2
6
12
0
19
7
0
12
3
6
0
14
3
13
8
0
0
0
0
0
0
0
0
0
0


3
0
1
1
0
1
9
47
7
1
0
0
0
0
0
0
0
1
0
0
3
6
6
0
0
0
0
0
0
6
9
0
0
0
32
0
0
0
0
0
2
19
58
12
12
8
2
2
5
0
2
0
0
1
2
43
2
6
12
0
19
7
0
12
3
6
0
14
3
13
8
0
0
0
0
0
0
0
0
0
0


3
0
1
1
0
1
9
47
7
1
0
0
0
0
0
0
0
1
0
0
3
6
6
0
0
0
0
0
0
6
9
0
0
0
32
0
0
0
0
0
2
19
58
12
12
8
2
2
5
0
2
0
0
1
2
43
2
6
12
0
19
7
0
12
3
6
0
14
3
13
8
0
0
0
0
0
0
0
0
0
0


3
0
1
1
0
1
9
47
7
1
0
0
0
0
0
0
0
1
0
0
3
6
6
0
0
0
0
0
0
6
9
0
0
0
32
0
0
0
0
0
2
19
58
12
12
8
2
2
5
0
2
0
0
1
2
43
2
6
12
0
19
7
0
12
3
6
0
14
3
13
8
0
0
0
0
0
0
0
0
0
0


522
551
430
1209
388
983
349
349
635
276
442
641
529
421
405
411
524
901
304
424
465
326
496
0
220
704
0
708
320
1037
1074
709
695
532
596
3757
562
473
717
590
520
658
792
1279
1679
1323
849
807
1094
360
336
1064
970
206
485
333
445
698
462
313
561
389
734
246
1125
826
715
358
529
790
493
1889
2184
1579
788
1664
1217
1077
1995
801
583


0
0
0
8
0
1
6
0
0
0
0
0
0
0
0
0
0
0
0
0
0
0
0
0
0
0
0
0
0
0
0
0
0
0
0
1
0
0
1
2
0
0
0
0
1
2
0
7
0
0
0
1
17
0
0
4
0
2
0
0
0
0
0
0
0
0
8
0
1
17
0
0
2
0
2
0
2
1
5
0
0


0
0
0
8
0
1
6
0
0
0
0
0
0
0
0
0
0
0
0
0
0
0
0
0
0
0
0
0
0
0
0
0
0
0
0
1
0
0
1
2
0
0
0
0
1
2
0
7
0
0
0
1
17
0
0
4
0
2
0
0
0
0
0
0
0
0
8
0
1
17
0
0
2
0
2
0
2
1
5
0
0


0
0
0
8
0
1
6
0
0
0
0
0
0
0
0
0
0
0
0
0
0
0
0
0
0
0
0
0
0
0
0
0
0
0
0
1
0
0
1
2
0
0
0
0
1
2
0
7
0
0
0
1
17
0
0
4
0
2
0
0
0
0
0
0
0
0
8
0
1
17
0
0
2
0
2
0
2
1
5
0
0


0
0
0
8
0
1
6
0
0
0
0
0
0
0
0
0
0
0
0
0
0
0
0
0
0
0
0
0
0
0
0
0
0
0
0
1
0
0
1
2
0
0
0
0
1
2
0
7
0
0
0
1
17
0
0
4
0
2
0
0
0
0
0
0
0
0
8
0
1
17
0
0
2
0
2
0
2
1
5
0
0


522
551
430
1201
388
982
343
349
635
275
441
640
529
421
405
411
524
901
304
424
465
326
496
0
220
704
0
708
320
1037
1073
709
695
532
596
3756
562
473
716
588
520
658
792
1279
1678
1321
848
800
1094
360
336
1063
953
206
485
329
445
696
462
313
561
389
734
246
1125
826
707
358
528
773
493
1888
2181
1579
784
1664
1215
1076
1990
801
583


238
342
260
485
207
365
170
158
362
129
298
307
348
202
271
235
323
736
199
188
385
210
362
0
218
558
0
596
318
545
462
18
257
84
261
674
308
312
450
285
314
348
427
642
701
444
664
557
517
310
169
429
419
109
250
181
213
339
226
198
275
238
148
138
489
269
259
216
276
314
185
507
772
516
244
510
313
375
806
217
210


238
342
260
485
207
365
170
158
362
129
298
307
348
202
271
235
323
736
199
188
385
210
362
0
218
558
0
596
318
545
462
18
257
84
261
674
308
312
450
285
314
348
427
642
701
444
664
557
517
310
169
429
419
109
250
181
213
339
226
198
275
238
148
138
489
269
259
216
276
314
185
507
772
516
244
510
313
375
806
217
210


238
342
260
485
207
365
170
158
362
129
298
307
348
202
271
235
323
736
199
188
385
210
362
0
218
558
0
596
318
545
462
18
257
84
261
674
308
312
450
285
314
348
427
642
701
444
664
557
517
310
169
429
419
109
250
181
213
339
226
198
275
238
148
138
489
269
259
216
276
314
185
507
772
516
244
510
313
375
806
217
210


0
0
0
0
0
0
0
0
0
0
0
0
0
0
0
0
0
0
0
0
1
0
2
0
0
2
0
0
0
0
0
0
0
0
0
0
0
0
0
0
0
0
0
0
0
0
0
0
1
0
0
0
0
0
0
0
0
0
0
0
0
0
0
0
0
0
0
0
0
0
0
0
0
0
0
0
0
0
0
0
0


0
0
0
0
0
0
0
0
0
0
0
0
0
0
0
0
0
0
0
0
1
0
2
0
0
2
0
0
0
0
0
0
0
0
0
0
0
0
0
0
0
0
0
0
0
0
0
0
1
0
0
0
0
0
0
0
0
0
0
0
0
0
0
0
0
0
0
0
0
0
0
0
0
0
0
0
0
0
0
0
0


0
0
0
0
0
0
0
0
0
0
0
0
0
0
0
0
0
0
0
0
1
0
2
0
0
2
0
0
0
0
0
0
0
0
0
0
0
0
0
0
0
0
0
0
0
0
0
0
1
0
0
0
0
0
0
0
0
0
0
0
0
0
0
0
0
0
0
0
0
0
0
0
0
0
0
0
0
0
0
0
0


33
29
20
20
32
3
24
14
26
30
17
13
20
11
19
25
44
12
10
23
1
3
10
0
0
8
0
0
0
0
1
0
0
0
20
7
0
0
3
0
4
40
26
25
0
3
2
0
34
0
13
2
7
5
12
11
10
2
10
3
1
8
0
0
3
8
0
6
2
0
10
0
0
3
1
0
0
0
2
0
0


22
29
20
9
31
2
23
13
26
24
17
12
20
11
19
25
44
12
10
21
1
2
10
0
0
8
0
0
0
0
1
0
0
0
20
6
0
0
3
0
4
27
26
25
0
3
2
0
34
0
13
2
7
5
12
11
10
2
10
3
1
8
0
0
3
8
0
6
2
0
10
0
0
2
1
0
0
0
0
0
0


22
29
20
9
31
2
23
13
26
24
17
12
20
11
19
25
44
12
10
21
1
2
10
0
0
8
0
0
0
0
1
0
0
0
20
6
0
0
3
0
4
27
26
25
0
3
2
0
34
0
13
2
7
5
12
11
10
2
10
3
1
8
0
0
3
8
0
6
2
0
10
0
0
2
1
0
0
0
0
0
0


11
0
0
11
1
1
1
1
0
6
0
1
0
0
0
0
0
0
0
2
0
1
0
0
0
0
0
0
0
0
0
0
0
0
0
1
0
0
0
0
0
13
0
0
0
0
0
0
0
0
0
0
0
0
0
0
0
0
0
0
0
0
0
0
0
0
0
0
0
0
0
0
0
1
0
0
0
0
2
0
0


11
0
0
11
1
1
1
1
0
6
0
1
0
0
0
0
0
0
0
2
0
1
0
0
0
0
0
0
0
0
0
0
0
0
0
1
0
0
0
0
0
13
0
0
0
0
0
0
0
0
0
0
0
0
0
0
0
0
0
0
0
0
0
0
0
0
0
0
0
0
0
0
0
1
0
0
0
0
2
0
0


0
0
0
0
0
2
0
0
0
0
0
0
0
0
0
0
0
0
0
1
0
0
0
0
0
0
0
0
0
0
0
0
0
0
0
0
0
0
0
0
0
0
1
0
2
1
0
0
0
0
0
2
0
0
0
0
1
2
0
0
0
1
0
0
0
0
12
0
9
0
0
0
0
0
0
0
4
4
4
0
1


0
0
0
0
0
2
0
0
0
0
0
0
0
0
0
0
0
0
0
1
0
0
0
0
0
0
0
0
0
0
0
0
0
0
0
0
0
0
0
0
0
0
1
0
2
1
0
0
0
0
0
2
0
0
0
0
1
2
0
0
0
1
0
0
0
0
12
0
9
0
0
0
0
0
0
0
4
4
4
0
1


0
0
0
0
0
2
0
0
0
0
0
0
0
0
0
0
0
0
0
1
0
0
0
0
0
0
0
0
0
0
0
0
0
0
0
0
0
0
0
0
0
0
1
0
2
1
0
0
0
0
0
2
0
0
0
0
1
2
0
0
0
1
0
0
0
0
12
0
9
0
0
0
0
0
0
0
4
4
4
0
1


0
0
1
0
0
0
1
0
0
0
5
0
2
0
0
0
0
1
6
0
1
0
0
0
0
0
0
0
0
0
0
0
0
0
0
0
0
0
0
0
0
0
0
0
0
0
0
0
0
0
0
0
0
3
0
0
0
0
0
1
0
0
0
1
0
0
0
0
0
0
0
0
0
0
0
0
0
0
0
0
0


0
0
1
0
0
0
1
0
0
0
5
0
2
0
0
0
0
1
6
0
1
0
0
0
0
0
0
0
0
0
0
0
0
0
0
0
0
0
0
0
0
0
0
0
0
0
0
0
0
0
0
0
0
3
0
0
0
0
0
1
0
0
0
1
0
0
0
0
0
0
0
0
0
0
0
0
0
0
0
0
0


0
0
1
0
0
0
1
0
0
0
5
0
2
0
0
0
0
1
6
0
1
0
0
0
0
0
0
0
0
0
0
0
0
0
0
0
0
0
0
0
0
0
0
0
0
0
0
0
0
0
0
0
0
3
0
0
0
0
0
1
0
0
0
1
0
0
0
0
0
0
0
0
0
0
0
0
0
0
0
0
0


0
0
0
0
0
0
0
0
0
0
0
0
0
0
0
0
0
0
0
0
0
0
0
0
0
0
0
0
0
0
0
0
0
0
0
0
0
0
0
0
0
0
0
0
0
0
0
0
0
0
0
0
1
0
0
0
0
0
0
0
0
0
0
0
0
0
0
0
0
0
0
0
0
0
0
0
0
0
0
0
0


0
0
0
0
0
0
0
0
0
0
0
0
0
0
0
0
0
0
0
0
0
0
0
0
0
0
0
0
0
0
0
0
0
0
0
0
0
0
0
0
0
0
0
0
0
0
0
0
0
0
0
0
1
0
0
0
0
0
0
0
0
0
0
0
0
0
0
0
0
0
0
0
0
0
0
0
0
0
0
0
0


0
0
0
0
0
0
0
0
0
0
0
0
0
0
0
0
0
0
0
0
0
0
0
0
0
0
0
0
0
0
0
0
0
0
0
0
0
0
0
0
0
0
0
0
0
0
0
0
0
0
0
0
1
0
0
0
0
0
0
0
0
0
0
0
0
0
0
0
0
0
0
0
0
0
0
0
0
0
0
0
0


201
83
54
613
114
521
106
147
206
98
86
277
123
179
86
109
118
94
67
176
43
80
75
0
0
83
0
76
0
433
547
656
388
424
250
2897
253
111
218
264
160
213
230
532
903
755
119
131
480
48
117
404
392
66
190
93
177
300
194
75
223
117
551
81
598
480
319
106
195
350
247
1229
1239
967
429
1001
774
565
1009
536
296


9
2
0
99
5
159
0
3
18
6
12
76
2
21
5
9
2
9
8
26
1
1
1
0
0
9
0
0
0
46
144
618
267
320
8
1204
130
0
11
80
14
6
5
150
281
375
7
8
146
0
2
12
23
1
3
8
9
48
7
0
14
6
362
0
95
26
37
1
16
18
20
378
476
168
296
192
570
200
261
148
100


9
2
0
99
5
159
0
3
18
6
12
76
2
21
5
9
2
9
8
26
1
1
1
0
0
9
0
0
0
46
144
618
267
320
8
1204
130
0
11
80
14
6
5
150
281
375
7
8
146
0
2
12
23
1
3
8
9
48
7
0
14
6
362
0
95
26
37
1
16
18
20
378
476
168
296
192
570
200
261
148
100


192
81
54
513
109
361
106
144
188
92
74
200
121
158
81
100
116
85
59
150
42
79
74
0
0
74
0
76
0
387
403
38
121
104
242
1690
123
111
207
184
146
207
224
382
619
378
112
123
333
48
115
392
369
65
187
85
168
252
187
75
208
111
189
81
503
453
282
105
179
331
227
850
762
798
133
809
203
365
748
388
195


192
81
54
513
109
361
106
144
188
92
74
200
121
158
81
100
116
85
59
150
42
79
74
0
0
74
0
76
0
387
403
38
121
104
242
1690
123
111
207
184
146
207
224
382
619
378
112
123
333
48
115
392
369
65
187
85
168
252
187
75
208
111
189
81
503
453
282
105
179
331
227
850
762
798
133
809
203
365
748
388
195


0
0
0
1
0
1
0
0
0
0
0
1
0
0
0
0
0
0
0
0
0
0
0
0
0
0
0
0
0
0
0
0
0
0
0
3
0
0
0
0
0
0
1
0
3
2
0
0
1
0
0
0
0
0
0
0
0
0
0
0
1
0
0
0
0
1
0
0
0
1
0
1
1
1
0
0
1
0
0
0
1


0
0
0
1
0
1
0
0
0
0
0
1
0
0
0
0
0
0
0
0
0
0
0
0
0
0
0
0
0
0
0
0
0
0
0
3
0
0
0
0
0
0
1
0
3
2
0
0
1
0
0
0
0
0
0
0
0
0
0
0
1
0
0
0
0
1
0
0
0
1
0
1
1
1
0
0
1
0
0
0
1


50
97
95
83
35
91
42
30
41
18
35
43
36
29
29
42
39
58
22
36
34
33
47
0
2
53
0
36
2
59
63
35
50
24
65
178
1
50
45
39
42
57
108
80
72
118
63
112
62
2
37
226
134
23
33
44
44
53
32
36
62
25
35
26
35
69
117
30
46
109
51
152
170
93
110
153
124
132
169
48
76


50
97
95
83
35
91
42
30
41
18
35
43
36
29
29
42
39
58
22
36
34
33
47
0
2
53
0
36
2
59
63
35
50
24
65
178
1
50
45
39
42
57
108
80
72
118
63
112
62
2
37
226
134
23
33
44
44
53
32
36
62
25
35
26
35
69
117
30
46
109
51
152
170
93
110
153
124
132
169
48
76


50
97
95
83
35
91
42
30
41
18
35
43
36
29
29
42
39
58
22
36
34
33
47
0
2
53
0
36
2
59
63
35
50
24
65
178
1
50
45
39
42
57
108
80
72
118
63
112
62
2
37
226
134
23
33
44
44
53
32
36
62
25
35
26
35
69
117
30
46
109
51
152
170
93
110
153
124
132
169
48
76


0
0
0
0
0
0
0
0
0
1
1
1
0
0
0
0
0
0
0
0
0
0
0
0
0
0
0
0
0
0
1
0
0
0
0
0
0
0
0
0
0
0
0
0
0
0
1
0
0
0
0
0
0
0
0
0
0
0
0
0
0
0
0
0
0
0
0
0
0
0
0
1
1
0
2
0
0
0
0
0
0


0
0
0
0
0
0
0
0
0
1
1
1
0
0
0
0
0
0
0
0
0
0
0
0
0
0
0
0
0
0
1
0
0
0
0
0
0
0
0
0
0
0
0
0
0
0
1
0
0
0
0
0
0
0
0
0
0
0
0
0
0
0
0
0
0
0
0
0
0
0
0
1
1
0
2
0
0
0
0
0
0


0
0
0
0
0
0
0
0
0
1
1
1
0
0
0
0
0
0
0
0
0
0
0
0
0
0
0
0
0
0
1
0
0
0
0
0
0
0
0
0
0
0
0
0
0
0
1
0
0
0
0
0
0
0
0
0
0
0
0
0
0
0
0
0
0
0
0
0
0
0
0
1
1
0
2
0
0
0
0
0
0


0
0
0
0
0
0
0
0
0
1
1
1
0
0
0
0
0
0
0
0
0
0
0
0
0
0
0
0
0
0
1
0
0
0
0
0
0
0
0
0
0
0
0
0
0
0
1
0
0
0
0
0
0
0
0
0
0
0
0
0
0
0
0
0
0
0
0
0
0
0
0
1
1
0
2
0
0
0
0
0
0


66
65
72
33
47
32
57
101
28
80
46
112
188
121
43
42
100
43
163
93
17
63
25
0
0
27
0
148
32
4
24
10
0
0
21
8
0
48
53
75
101
79
53
19
15
9
182
61
34
0
68
71
23
117
86
45
58
59
148
78
69
95
8
90
25
102
8
43
28
5
34
15
40
56
16
7
4
13
27
5
14


66
65
72
33
47
32
57
101
28
80
46
112
188
121
43
42
100
43
163
93
17
63
25
0
0
27
0
148
32
4
24
10
0
0
21
8
0
48
53
75
101
79
53
19
15
9
182
61
34
0
68
71
23
117
86
45
58
59
148
78
69
95
8
90
25
102
8
43
28
5
34
15
40
56
16
7
4
13
27
5
14


66
65
72
33
47
32
57
101
28
80
46
112
188
121
43
42
100
43
163
93
17
63
25
0
0
27
0
148
32
4
24
10
0
0
21
8
0
48
53
75
101
79
53
19
15
9
182
61
34
0
68
71
23
117
86
45
58
59
148
78
69
95
8
90
25
102
8
43
28
5
34
15
40
56
16
7
4
13
27
5
14


66
65
72
33
47
32
57
101
28
80
46
112
188
121
43
42
100
43
163
93
17
63
25
0
0
27
0
148
32
4
24
10
0
0
21
8
0
48
53
75
101
79
53
19
15
9
182
61
34
0
68
71
23
117
86
45
58
59
148
78
69
95
8
90
25
102
8
43
28
5
34
15
40
56
16
7
4
13
27
5
14


66
65
72
33
47
32
57
101
28
80
46
112
188
121
43
42
100
43
163
93
17
63
25
0
0
27
0
148
32
4
24
10
0
0
21
8
0
48
53
75
101
79
53
19
15
9
182
61
34
0
68
71
23
117
86
45
58
59
148
78
69
95
8
90
25
102
8
43
28
5
34
15
40
56
16
7
4
13
27
5
14


7
4
0
45
7
64
25
17
9
6
1
3
17
3
3
3
1
2
6
13
0
4
0
0
0
0
0
0
0
3
3
0
0
10
4
28
0
0
0
4
0
7
8
12
24
29
0
0
0
0
3
56
90
18
3
12
17
4
5
3
16
8
7
5
30
6
47
6
20
39
15
24
28
15
55
48
23
42
65
10
18


7
4
0
45
7
64
25
17
9
6
1
3
17
3
3
3
1
2
6
13
0
4
0
0
0
0
0
0
0
3
3
0
0
10
4
28
0
0
0
4
0
7
8
12
24
29
0
0
0
0
3
56
90
18
3
12
17
4
5
3
16
8
7
5
30
6
47
6
20
39
15
24
28
15
55
48
23
42
65
10
18


7
4
0
45
7
64
25
17
9
6
1
3
17
3
3
3
1
2
6
13
0
4
0
0
0
0
0
0
0
3
3
0
0
10
4
28
0
0
0
4
0
7
8
12
24
29
0
0
0
0
3
56
90
18
3
12
17
4
5
3
16
8
7
5
30
6
47
6
20
39
15
24
28
15
55
48
23
42
65
10
18


7
4
0
45
7
64
25
17
9
6
1
3
17
3
3
3
1
2
6
13
0
4
0
0
0
0
0
0
0
3
3
0
0
10
4
28
0
0
0
4
0
7
8
12
24
29
0
0
0
0
3
56
90
18
3
12
17
4
5
3
16
8
7
5
30
6
47
6
20
39
15
24
28
15
55
48
23
42
65
10
18


7
4
0
45
7
64
25
17
9
6
1
3
17
3
3
3
1
2
6
13
0
4
0
0
0
0
0
0
0
3
3
0
0
10
4
28
0
0
0
4
0
7
8
12
24
29
0
0
0
0
3
56
90
18
3
12
17
4
5
3
16
8
7
5
30
6
47
6
20
39
15
24
28
15
55
48
23
42
65
10
18


259
307
330
157
171
131
229
263
296
50
68
95
129
92
89
125
1191
288
89
106
312
180
199
161
52
153
7
145
751
228
151
0
47
16
155
60
0
162
307
116
203
129
126
149
116
75
150
240
101
94
115
119
361
297
101
481
247
123
84
149
171
153
48
38
162
223
347
58
201
548
88
71
134
173
88
35
61
98
170
38
61


0
0
2
0
3
0
0
5
2
2
0
1
0
0
0
1
0
0
2
0
3
2
1
0
0
0
0
0
0
0
2
0
0
0
0
0
0
3
1
1
0
2
1
0
1
3
1
10
2
0
3
0
0
2
2
2
2
0
1
1
2
1
0
1
0
0
0
2
0
0
0
0
0
0
0
0
0
0
0
0
0


0
0
2
0
3
0
0
5
2
2
0
1
0
0
0
1
0
0
2
0
3
2
1
0
0
0
0
0
0
0
2
0
0
0
0
0
0
3
1
1
0
2
1
0
1
3
1
10
2
0
3
0
0
2
2
2
2
0
1
1
2
1
0
1
0
0
0
2
0
0
0
0
0
0
0
0
0
0
0
0
0


0
0
2
0
3
0
0
5
2
2
0
1
0
0
0
1
0
0
2
0
3
2
1
0
0
0
0
0
0
0
2
0
0
0
0
0
0
3
1
1
0
2
1
0
1
3
1
10
2
0
3
0
0
2
2
2
2
0
1
1
2
1
0
1
0
0
0
2
0
0
0
0
0
0
0
0
0
0
0
0
0


0
0
2
0
3
0
0
5
2
2
0
1
0
0
0
1
0
0
2
0
3
2
1
0
0
0
0
0
0
0
2
0
0
0
0
0
0
3
1
1
0
2
1
0
1
3
1
10
2
0
3
0
0
2
2
2
2
0
1
1
2
1
0
1
0
0
0
2
0
0
0
0
0
0
0
0
0
0
0
0
0


48
2
2
31
28
2
63
36
13
26
5
20
4
17
10
8
14
6
6
11
37
27
16
0
0
0
0
5
0
144
18
0
0
15
103
6
0
2
5
6
9
37
51
36
33
28
1
3
14
11
14
12
31
3
25
12
24
18
29
5
22
12
12
2
31
50
78
8
45
61
10
7
20
23
6
7
8
17
52
21
8


48
2
2
31
28
2
63
36
13
26
5
20
4
17
10
8
14
6
6
11
37
27
16
0
0
0
0
5
0
144
18
0
0
15
103
6
0
2
5
6
9
37
51
36
33
28
1
3
14
11
14
12
31
3
25
12
24
18
29
5
22
12
12
2
31
50
78
8
45
61
10
7
20
23
6
7
8
17
52
21
8


48
2
2
31
28
2
63
36
13
26
5
20
4
17
10
8
14
6
6
11
37
27
16
0
0
0
0
5
0
144
18
0
0
15
103
6
0
2
5
6
9
37
51
36
33
28
1
3
14
11
14
12
31
3
25
12
24
18
29
5
22
12
12
2
31
50
78
8
45
61
10
7
20
23
6
7
8
17
52
21
8


48
2
2
31
28
2
63
36
13
26
5
20
4
17
10
8
14
6
6
11
37
27
16
0
0
0
0
5
0
144
18
0
0
15
103
6
0
2
5
6
9
37
51
36
33
28
1
3
14
11
14
12
31
3
25
12
24
18
29
5
22
12
12
2
31
50
78
8
45
61
10
7
20
23
6
7
8
17
52
21
8


202
304
326
121
133
127
149
216
280
20
63
68
124
75
78
110
1175
280
79
91
270
147
180
161
52
153
7
138
751
84
130
0
47
1
52
54
0
154
295
109
194
83
70
108
82
42
147
227
81
83
97
107
327
288
67
461
218
94
50
143
147
138
36
35
129
163
267
38
141
487
72
63
114
147
82
27
53
81
114
17
53


202
304
326
121
133
127
149
216
280
20
63
68
124
75
78
110
1175
280
79
91
270
147
180
161
52
153
7
138
751
84
130
0
47
1
52
54
0
154
295
109
194
83
70
108
82
42
147
227
81
83
97
107
327
288
67
461
218
94
50
143
147
138
36
35
129
163
267
38
141
487
72
63
114
147
82
27
53
81
114
17
53


202
304
326
121
133
127
149
216
280
20
63
68
124
75
78
110
1175
280
79
91
270
147
180
161
52
153
7
138
751
84
130
0
47
1
52
54
0
154
295
109
194
83
70
108
82
42
147
227
81
83
97
107
327
288
67
461
218
94
50
143
147
138
36
35
129
163
267
38
141
487
72
63
114
147
82
27
53
81
114
17
53


202
304
326
121
133
127
149
216
280
20
63
68
124
75
78
110
1175
280
79
91
270
147
180
161
52
153
7
138
751
84
130
0
47
1
52
54
0
154
295
109
194
83
70
108
82
42
147
227
81
83
97
107
327
288
67
461
218
94
50
143
147
138
36
35
129
163
267
38
141
487
72
63
114
147
82
27
53
81
114
17
53


9
0
0
5
7
2
17
6
1
2
0
6
0
0
1
4
1
1
0
3
0
4
1
0
0
0
0
2
0
0
0
0
0
0
0
0
0
3
6
0
0
7
4
5
0
2
0
0
4
0
1
0
2
3
7
5
3
11
3
0
0
2
0
0
2
10
2
10
14
0
6
1
0
2
0
1
0
0
4
0
0


9
0
0
5
7
2
17
6
1
2
0
6
0
0
1
4
1
1
0
3
0
4
1
0
0
0
0
2
0
0
0
0
0
0
0
0
0
3
6
0
0
7
4
5
0
2
0
0
4
0
1
0
2
3
7
5
3
11
3
0
0
2
0
0
2
10
2
10
14
0
6
1
0
2
0
1
0
0
4
0
0


9
0
0
5
7
2
17
6
1
2
0
6
0
0
1
4
1
1
0
3
0
4
1
0
0
0
0
2
0
0
0
0
0
0
0
0
0
3
6
0
0
7
4
5
0
2
0
0
4
0
1
0
2
3
7
5
3
11
3
0
0
2
0
0
2
10
2
10
14
0
6
1
0
2
0
1
0
0
4
0
0


9
0
0
5
7
2
17
6
1
2
0
6
0
0
1
4
1
1
0
3
0
4
1
0
0
0
0
2
0
0
0
0
0
0
0
0
0
3
6
0
0
7
4
5
0
2
0
0
4
0
1
0
2
3
7
5
3
11
3
0
0
2
0
0
2
10
2
10
14
0
6
1
0
2
0
1
0
0
4
0
0


0
1
0
0
0
0
0
0
0
0
0
0
1
0
0
2
1
1
2
1
2
0
1
0
0
0
0
0
0
0
1
0
0
0
0
0
0
0
0
0
0
0
0
0
0
0
1
0
0
0
0
0
1
1
0
1
0
0
1
0
0
0
0
0
0
0
0
0
1
0
0
0
0
1
0
0
0
0
0
0
0


0
1
0
0
0
0
0
0
0
0
0
0
1
0
0
2
1
1
2
1
2
0
1
0
0
0
0
0
0
0
1
0
0
0
0
0
0
0
0
0
0
0
0
0
0
0
1
0
0
0
0
0
1
1
0
1
0
0
1
0
0
0
0
0
0
0
0
0
1
0
0
0
0
1
0
0
0
0
0
0
0


0
1
0
0
0
0
0
0
0
0
0
0
1
0
0
2
1
1
2
1
2
0
1
0
0
0
0
0
0
0
1
0
0
0
0
0
0
0
0
0
0
0
0
0
0
0
1
0
0
0
0
0
1
1
0
1
0
0
1
0
0
0
0
0
0
0
0
0
1
0
0
0
0
1
0
0
0
0
0
0
0


0
1
0
0
0
0
0
0
0
0
0
0
1
0
0
2
1
1
2
1
2
0
1
0
0
0
0
0
0
0
1
0
0
0
0
0
0
0
0
0
0
0
0
0
0
0
1
0
0
0
0
0
1
1
0
1
0
0
1
0
0
0
0
0
0
0
0
0
1
0
0
0
0
1
0
0
0
0
0
0
0


38
12
10
21
9
30
33
28
15
24
18
20
9
15
11
12
19
12
15
14
11
16
5
0
0
4
0
4
1
25
8
0
0
0
7
6
0
2
13
5
19
16
20
10
16
11
13
2
19
0
26
24
37
13
26
28
32
42
33
20
25
21
21
16
11
38
34
25
26
3
37
27
30
11
43
20
43
16
29
10
50


38
12
10
21
9
30
33
28
15
24
18
20
9
15
11
12
19
12
15
14
11
16
5
0
0
4
0
4
1
25
8
0
0
0
7
6
0
2
13
5
19
16
20
10
16
11
13
2
19
0
26
24
37
13
26
28
32
42
33
20
25
21
21
16
11
38
34
25
26
3
37
27
30
11
43
20
43
16
29
10
50


38
12
10
21
9
30
33
28
15
24
18
20
9
15
11
12
19
12
15
14
11
16
5
0
0
4
0
4
1
25
8
0
0
0
7
6
0
2
13
5
19
16
20
10
16
11
13
2
19
0
26
24
37
13
26
28
32
42
33
20
25
21
21
16
11
38
34
25
26
3
37
27
30
11
43
20
43
16
29
10
50


38
12
10
21
9
30
33
28
15
24
18
20
9
15
11
12
19
12
15
14
11
16
5
0
0
4
0
4
1
25
8
0
0
0
7
6
0
2
13
5
19
16
20
10
16
11
13
2
19
0
26
24
37
13
26
28
32
42
33
20
25
21
21
16
11
38
34
25
26
3
37
27
30
11
43
20
43
16
29
10
50


38
12
10
21
9
30
33
28
15
24
18
20
9
15
11
12
19
12
15
14
11
16
5
0
0
4
0
4
1
25
8
0
0
0
7
6
0
2
13
5
19
16
20
10
16
11
13
2
19
0
26
24
37
13
26
28
32
42
33
20
25
21
21
16
11
38
34
25
26
3
37
27
30
11
43
20
43
16
29
10
50


1688
4733
7030
5096
4087
5936
3634
2805
2516
2493
3861
3314
3430
2171
5250
5335
7513
6055
5417
7529
6122
7998
4967
1962
3324
8215
3955
7116
5264
5074
6819
8577
5451
10276
3759
7492
2578
2903
4012
10168
7261
5143
4388
6346
7351
5080
7973
6923
7365
2449
2630
4464
2886
3783
3423
4744
4611
4772
6314
7603
6033
3051
6067
2216
3185
4114
1949
2798
2695
2658
3517
5226
4980
4690
2110
4296
3661
3595
2813
2053
1750


223
562
939
500
566
660
597
426
292
221
420
348
389
237
558
483
765
508
472
742
414
628
472
24
275
442
67
436
0
443
759
524
170
461
501
394
162
486
562
589
516
501
538
545
599
466
489
479
603
351
360
591
537
537
536
651
587
548
915
1207
826
428
340
409
340
523
248
344
646
254
440
425
465
460
145
469
262
410
298
171
148


223
562
939
500
566
660
597
426
292
221
420
348
389
237
558
483
765
508
472
742
414
628
472
24
275
442
67
436
0
443
759
524
170
461
501
394
162
486
562
589
516
501
538
545
599
466
489
479
603
351
360
591
537
537
536
651
587
548
915
1207
826
428
340
409
340
523
248
344
646
254
440
425
465
460
145
469
262
410
298
171
148


106
319
524
276
272
400
308
157
116
71
157
121
137
89
201
227
250
213
169
440
197
228
130
0
273
167
0
184
0
188
431
386
108
57
184
222
70
367
365
261
165
174
182
184
154
124
254
232
192
295
146
424
369
325
227
398
327
327
320
744
321
177
219
161
147
262
142
111
422
143
155
227
289
195
102
255
204
304
160
86
81


106
319
524
276
272
400
308
157
116
71
157
121
137
89
201
227
250
213
169
440
197
228
130
0
273
167
0
184
0
188
431
386
108
57
184
222
70
367
365
261
165
174
182
184
154
124
254
232
192
295
146
424
369
325
227
398
327
327
320
744
321
177
219
161
147
262
142
111
422
143
155
227
289
195
102
255
204
304
160
86
81


106
319
524
276
272
400
308
157
116
71
157
121
137
89
201
227
250
213
169
440
197
228
130
0
273
167
0
184
0
188
431
386
108
57
184
222
70
367
365
261
165
174
182
184
154
124
254
232
192
295
146
424
369
325
227
398
327
327
320
744
321
177
219
161
147
262
142
111
422
143
155
227
289
195
102
255
204
304
160
86
81


1
17
42
3
50
25
22
19
13
14
51
49
45
6
102
57
87
46
84
28
45
107
95
0
0
48
48
33
0
58
57
5
0
83
90
19
0
0
25
79
153
98
130
91
86
40
34
59
125
0
28
8
4
15
48
34
27
18
153
51
114
44
2
15
15
36
1
28
11
14
32
0
0
1
0
0
0
0
0
1
0


1
17
42
3
50
25
22
19
13
14
51
49
45
6
102
57
87
46
84
28
45
107
95
0
0
48
48
33
0
58
57
5
0
83
90
19
0
0
25
79
153
98
130
91
86
40
34
59
125
0
28
8
4
15
48
34
27
18
153
51
114
44
2
15
15
36
1
28
11
14
32
0
0
1
0
0
0
0
0
1
0


1
17
42
3
50
25
22
19
13
14
51
49
45
6
102
57
87
46
84
28
45
107
95
0
0
48
48
33
0
58
57
5
0
83
90
19
0
0
25
79
153
98
130
91
86
40
34
59
125
0
28
8
4
15
48
34
27
18
153
51
114
44
2
15
15
36
1
28
11
14
32
0
0
1
0
0
0
0
0
1
0


7
30
46
18
24
21
45
16
20
12
22
26
46
23
39
21
92
44
31
29
28
48
38
0
0
35
0
37
0
32
38
12
3
25
8
13
0
13
15
28
23
40
34
37
84
57
24
47
32
0
15
16
5
20
29
26
27
21
50
58
31
24
4
25
24
25
5
18
8
1
16
25
8
32
4
20
6
13
18
15
4


7
30
46
18
24
21
45
16
20
12
22
26
46
23
39
21
92
44
31
29
28
48
38
0
0
35
0
37
0
32
38
12
3
25
8
13
0
13
15
28
23
40
34
37
84
57
24
47
32
0
15
16
5
20
29
26
27
21
50
58
31
24
4
25
24
25
5
18
8
1
16
25
8
32
4
20
6
13
18
15
4


7
30
46
18
24
21
45
16
20
12
22
26
46
23
39
21
92
44
31
29
28
48
38
0
0
35
0
37
0
32
38
12
3
25
8
13
0
13
15
28
23
40
34
37
84
57
24
47
32
0
15
16
5
20
29
26
27
21
50
58
31
24
4
25
24
25
5
18
8
1
16
25
8
32
4
20
6
13
18
15
4


65
73
141
98
107
85
129
154
83
72
103
87
70
60
123
87
187
101
102
128
87
163
145
24
2
94
0
120
0
95
120
119
46
206
175
96
0
42
64
138
121
129
153
185
213
171
100
123
161
54
104
64
83
117
127
110
115
88
248
168
216
108
64
135
90
118
41
114
81
64
147
90
100
153
19
110
30
61
70
41
36


65
73
141
98
107
85
129
154
83
72
103
87
70
60
123
87
187
101
102
128
87
163
145
24
2
94
0
120
0
95
120
119
46
206
175
96
0
42
64
138
121
129
153
185
213
171
100
123
161
54
104
64
83
117
127
110
115
88
248
168
216
108
64
135
90
118
41
114
81
64
147
90
100
153
19
110
30
61
70
41
36


65
73
141
98
107
85
129
154
83
72
103
87
70
60
123
87
187
101
102
128
87
163
145
24
2
94
0
120
0
95
120
119
46
206
175
96
0
42
64
138
121
129
153
185
213
171
100
123
161
54
104
64
83
117
127
110
115
88
248
168
216
108
64
135
90
118
41
114
81
64
147
90
100
153
19
110
30
61
70
41
36


7
48
102
21
37
44
30
28
29
11
46
22
45
19
43
30
50
42
27
31
12
12
10
0
0
4
0
2
0
11
17
0
1
0
0
13
0
5
7
20
19
19
5
5
1
3
10
0
14
0
12
18
16
11
15
9
19
17
42
42
37
22
19
42
13
16
9
34
15
0
19
0
2
0
0
0
0
7
0
2
1


0
1
2
0
0
0
1
0
0
0
2
0
1
0
0
0
0
0
0
0
0
0
0
0
0
0
0
0
0
0
1
0
0
0
0
0
0
0
0
0
0
0
0
0
0
0
1
0
0
0
0
0
0
0
0
0
0
0
0
0
0
0
0
0
0
0
0
0
0
0
0
0
0
0
0
0
0
0
0
0
0


0
1
2
0
0
0
1
0
0
0
2
0
1
0
0
0
0
0
0
0
0
0
0
0
0
0
0
0
0
0
1
0
0
0
0
0
0
0
0
0
0
0
0
0
0
0
1
0
0
0
0
0
0
0
0
0
0
0
0
0
0
0
0
0
0
0
0
0
0
0
0
0
0
0
0
0
0
0
0
0
0


7
47
97
21
36
43
29
27
27
11
44
22
43
19
43
30
49
41
27
30
12
12
10
0
0
4
0
2
0
11
16
0
1
0
0
13
0
5
7
20
19
19
5
5
1
3
8
0
14
0
11
18
16
11
15
8
19
17
41
41
36
22
19
41
13
15
9
34
15
0
19
0
2
0
0
0
0
7
0
2
1


7
47
97
21
36
43
29
27
27
11
44
22
43
19
43
30
49
41
27
30
12
12
10
0
0
4
0
2
0
11
16
0
1
0
0
13
0
5
7
20
19
19
5
5
1
3
8
0
14
0
11
18
16
11
15
8
19
17
41
41
36
22
19
41
13
15
9
34
15
0
19
0
2
0
0
0
0
7
0
2
1


0
0
3
0
1
1
0
1
2
0
0
0
1
0
0
0
1
1
0
1
0
0
0
0
0
0
0
0
0
0
0
0
0
0
0
0
0
0
0
0
0
0
0
0
0
0
1
0
0
0
1
0
0
0
0
1
0
0
1
1
1
0
0
1
0
1
0
0
0
0
0
0
0
0
0
0
0
0
0
0
0


0
0
3
0
1
1
0
1
2
0
0
0
1
0
0
0
1
1
0
1
0
0
0
0
0
0
0
0
0
0
0
0
0
0
0
0
0
0
0
0
0
0
0
0
0
0
1
0
0
0
1
0
0
0
0
1
0
0
1
1
1
0
0
1
0
1
0
0
0
0
0
0
0
0
0
0
0
0
0
0
0


3
5
2
1
1
0
2
0
0
1
0
0
0
0
5
0
12
3
1
3
1
1
0
0
0
3
0
2
0
4
0
0
0
0
0
0
0
1
3
2
3
1
0
1
2
0
0
1
3
0
2
0
0
0
0
0
1
0
0
0
1
4
1
0
0
0
0
0
0
0
2
0
0
0
0
0
0
0
0
0
0


3
5
2
1
1
0
2
0
0
1
0
0
0
0
5
0
12
3
1
3
1
1
0
0
0
3
0
2
0
4
0
0
0
0
0
0
0
1
3
2
3
1
0
1
2
0
0
1
3
0
2
0
0
0
0
0
1
0
0
0
1
4
1
0
0
0
0
0
0
0
2
0
0
0
0
0
0
0
0
0
0


3
5
2
1
1
0
2
0
0
1
0
0
0
0
5
0
12
3
1
3
1
1
0
0
0
3
0
2
0
4
0
0
0
0
0
0
0
1
3
2
3
1
0
1
2
0
0
1
3
0
2
0
0
0
0
0
1
0
0
0
1
4
1
0
0
0
0
0
0
0
2
0
0
0
0
0
0
0
0
0
0


0
0
1
0
0
0
1
0
1
0
1
1
0
2
0
1
0
1
0
1
0
11
1
0
0
0
0
0
0
0
0
0
0
0
0
0
0
0
3
2
0
4
1
0
0
0
3
0
0
0
1
1
1
1
2
1
2
0
4
0
1
0
0
0
0
1
0
1
0
0
2
0
0
3
0
0
0
0
0
1
0


0
0
1
0
0
0
1
0
1
0
1
1
0
2
0
1
0
1
0
1
0
11
1
0
0
0
0
0
0
0
0
0
0
0
0
0
0
0
3
2
0
4
1
0
0
0
3
0
0
0
1
1
1
1
2
1
2
0
4
0
1
0
0
0
0
1
0
1
0
0
2
0
0
3
0
0
0
0
0
1
0


0
0
1
0
0
0
1
0
1
0
1
1
0
2
0
1
0
1
0
1
0
11
1
0
0
0
0
0
0
0
0
0
0
0
0
0
0
0
3
2
0
4
1
0
0
0
3
0
0
0
1
1
1
1
2
1
2
0
4
0
1
0
0
0
0
1
0
1
0
0
2
0
0
3
0
0
0
0
0
1
0


34
70
81
83
75
85
60
52
30
40
40
42
46
38
45
60
87
58
58
82
44
58
53
0
0
91
19
58
0
55
96
2
12
90
44
31
92
58
80
59
32
36
33
42
59
71
64
17
76
2
52
60
59
48
88
73
69
77
98
144
105
49
31
31
51
65
50
38
109
32
67
83
66
76
20
84
22
25
50
25
26


34
70
81
83
75
85
60
52
30
40
40
42
46
38
45
60
87
58
58
82
44
58
53
0
0
91
19
58
0
55
96
2
12
90
44
31
92
58
80
59
32
36
33
42
59
71
64
17
76
2
52
60
59
48
88
73
69
77
98
144
105
49
31
31
51
65
50
38
109
32
67
83
66
76
20
84
22
25
50
25
26


34
70
81
83
75
85
60
52
30
40
40
42
46
38
45
60
87
58
58
82
44
58
53
0
0
91
19
58
0
55
96
2
12
90
44
31
92
58
80
59
32
36
33
42
59
71
64
17
76
2
52
60
59
48
88
73
69
77
98
144
105
49
31
31
51
65
50
38
109
32
67
83
66
76
20
84
22
25
50
25
26


732
2071
2729
2550
2098
3087
1710
1028
1521
1139
2208
1716
2019
1050
2466
2691
3567
4084
3003
3504
1922
3355
2239
1592
2617
4411
3344
3675
4671
2649
3555
5827
4172
6345
1543
5289
1779
877
1829
6319
4196
2892
1745
3144
4204
2281
4176
3140
4185
327
1249
2545
1483
1647
1788
2274
2212
2321
2928
2544
2728
1523
4518
745
1690
1828
1114
1397
1078
1842
1878
2693
2404
1987
956
2206
1543
1473
1418
1150
942


681
2013
2628
2463
2028
3050
1668
972
1476
1081
2181
1684
2002
1034
2404
2629
3443
4018
2882
3428
1784
3241
2192
1592
2617
4376
3344
3584
4671
2512
3428
5771
4131
6199
1322
5248
1778
855
1785
6177
4046
2764
1621
3006
3972
1982
4068
3040
4099
209
1228
2516
1472
1638
1744
2264
2191
2287
2909
2516
2695
1494
4508
740
1686
1809
1109
1381
1077
1840
1864
2692
2397
1981
955
2202
1541
1471
1416
1147
938


12
2
18
190
20
134
11
14
24
23
12
49
21
9
11
16
16
1
11
81
5
15
14
0
0
1
0
4
0
69
142
481
263
541
138
1080
79
1
46
166
44
59
36
117
279
88
9
9
190
0
19
51
14
8
18
5
39
19
25
8
21
25
361
4
106
26
33
8
11
22
32
242
186
148
76
171
75
107
90
97
79


12
2
18
190
20
134
11
14
24
23
12
49
21
9
11
16
16
1
11
81
5
15
14
0
0
1
0
4
0
69
142
481
263
541
138
1080
79
1
46
166
44
59
36
117
279
88
9
9
190
0
19
51
14
8
18
5
39
19
25
8
21
25
361
4
106
26
33
8
11
22
32
242
186
148
76
171
75
107
90
97
79


12
2
18
190
20
134
11
14
24
23
12
49
21
9
11
16
16
1
11
81
5
15
14
0
0
1
0
4
0
69
142
481
263
541
138
1080
79
1
46
166
44
59
36
117
279
88
9
9
190
0
19
51
14
8
18
5
39
19
25
8
21
25
361
4
106
26
33
8
11
22
32
242
186
148
76
171
75
107
90
97
79


0
1
1
1
0
0
0
2
0
0
0
0
1
1
1
1
0
0
1
2
1
0
1
0
0
0
0
2
0
1
0
0
11
2
0
0
0
0
0
1
0
0
0
0
2
0
0
0
0
0
1
0
0
1
0
0
0
0
0
1
2
0
1
0
1
0
0
0
0
0
0
1
0
1
1
0
0
0
0
1
0


0
1
1
1
0
0
0
2
0
0
0
0
1
1
1
1
0
0
1
2
1
0
1
0
0
0
0
2
0
1
0
0
11
2
0
0
0
0
0
1
0
0
0
0
2
0
0
0
0
0
1
0
0
1
0
0
0
0
0
1
2
0
1
0
1
0
0
0
0
0
0
1
0
1
1
0
0
0
0
1
0


0
1
1
1
0
0
0
2
0
0
0
0
1
1
1
1
0
0
1
2
1
0
1
0
0
0
0
2
0
1
0
0
11
2
0
0
0
0
0
1
0
0
0
0
2
0
0
0
0
0
1
0
0
1
0
0
0
0
0
1
2
0
1
0
1
0
0
0
0
0
0
1
0
1
1
0
0
0
0
1
0


0
2
0
1
1
1
3
0
0
0
0
0
0
0
0
1
0
0
0
0
2
0
0
184
44
3
126
7
91
0
0
1
60
0
7
0
1
2
0
0
1
0
0
2
0
0
0
1
3
0
1
0
1
1
1
0
0
0
1
0
1
1
0
0
0
0
2
0
1
1
1
0
0
0
1
9
0
1
3
0
0


0
0
0
0
0
0
0
0
0
0
0
0
0
0
0
0
0
0
0
0
0
0
0
0
0
0
0
0
0
0
0
0
0
0
6
0
0
1
0
0
0
0
0
0
0
0
0
0
0
0
0
0
0
0
0
0
0
0
0
0
0
0
0
0
0
0
0
0
0
0
0
0
0
0
0
0
0
0
0
0
0


0
0
0
0
0
0
0
0
0
0
0
0
0
0
0
0
0
0
0
0
0
0
0
0
0
0
0
0
0
0
0
0
0
0
6
0
0
1
0
0
0
0
0
0
0
0
0
0
0
0
0
0
0
0
0
0
0
0
0
0
0
0
0
0
0
0
0
0
0
0
0
0
0
0
0
0
0
0
0
0
0


0
0
0
0
0
0
0
0
0
0
0
0
0
0
0
0
0
0
0
0
0
0
0
0
0
0
31
5
0
0
0
0
40
0
0
0
0
0
0
0
0
0
0
0
0
0
0
0
0
0
0
0
0
0
0
0
0
0
0
0
0
0
0
0
0
0
0
0
0
0
0
0
0
0
0
0
0
0
0
0
0


0
0
0
0
0
0
0
0
0
0
0
0
0
0
0
0
0
0
0
0
0
0
0
0
0
0
31
5
0
0
0
0
40
0
0
0
0
0
0
0
0
0
0
0
0
0
0
0
0
0
0
0
0
0
0
0
0
0
0
0
0
0
0
0
0
0
0
0
0
0
0
0
0
0
0
0
0
0
0
0
0


0
2
0
1
1
1
3
0
0
0
0
0
0
0
0
1
0
0
0
0
2
0
0
184
44
3
95
2
91
0
0
1
20
0
1
0
1
1
0
0
1
0
0
2
0
0
0
1
3
0
1
0
1
1
1
0
0
0
0
0
1
1
0
0
0
0
2
0
1
1
1
0
0
0
1
1
0
1
3
0
0


0
2
0
1
0
1
3
0
0
0
0
0
0
0
0
1
0
0
0
0
2
0
0
184
44
3
95
2
91
0
0
1
20
0
1
0
1
1
0
0
1
0
0
2
0
0
0
1
1
0
1
0
1
1
1
0
0
0
0
0
1
1
0
0
0
0
1
0
1
1
1
0
0
0
1
1
0
1
3
0
0


0
0
0
0
1
0
0
0
0
0
0
0
0
0
0
0
0
0
0
0
0
0
0
0
0
0
0
0
0
0
0
0
0
0
0
0
0
0
0
0
0
0
0
0
0
0
0
0
2
0
0
0
0
0
0
0
0
0
0
0
0
0
0
0
0
0
1
0
0
0
0
0
0
0
0
0
0
0
0
0
0


0
0
0
0
0
0
0
0
0
0
0
0
0
0
0
0
0
0
0
0
0
0
0
0
0
0
0
0
0
0
0
0
0
0
0
0
0
0
0
0
0
0
0
0
0
0
0
0
0
0
0
0
0
0
0
0
0
0
1
0
0
0
0
0
0
0
0
0
0
0
0
0
0
0
0
0
0
0
0
0
0


0
0
0
0
0
0
0
0
0
0
0
0
0
0
0
0
0
0
0
0
0
0
0
0
0
0
0
0
0
0
0
0
0
0
0
0
0
0
0
0
0
0
0
0
0
0
0
0
0
0
0
0
0
0
0
0
0
0
1
0
0
0
0
0
0
0
0
0
0
0
0
0
0
0
0
0
0
0
0
0
0


0
0
0
0
0
0
0
0
0
0
0
0
0
0
0
0
0
0
0
0
0
0
0
0
0
0
0
0
0
0
0
0
0
0
0
0
0
0
0
0
0
0
0
0
0
0
0
0
0
0
0
0
0
0
0
0
0
0
0
0
0
0
0
0
0
0
0
0
0
0
0
0
0
0
0
8
0
0
0
0
0


0
0
0
0
0
0
0
0
0
0
0
0
0
0
0
0
0
0
0
0
0
0
0
0
0
0
0
0
0
0
0
0
0
0
0
0
0
0
0
0
0
0
0
0
0
0
0
0
0
0
0
0
0
0
0
0
0
0
0
0
0
0
0
0
0
0
0
0
0
0
0
0
0
0
0
8
0
0
0
0
0


0
0
0
4
1
1
0
0
2
2
0
0
0
1
1
1
1
0
0
2
0
0
0
0
0
0
0
0
0
0
0
26
10
8
0
90
0
0
0
3
0
0
0
3
9
0
0
0
0
0
1
4
3
0
1
16
5
1
1
0
0
0
4
1
1
0
0
0
1
1
1
22
11
5
12
5
26
4
12
1
2


0
0
0
4
1
1
0
0
2
2
0
0
0
1
1
1
1
0
0
2
0
0
0
0
0
0
0
0
0
0
0
26
10
8
0
90
0
0
0
3
0
0
0
3
9
0
0
0
0
0
1
4
3
0
1
16
5
1
1
0
0
0
4
1
1
0
0
0
1
1
1
22
11
5
12
5
26
4
12
1
2


0
0
0
4
1
1
0
0
2
2
0
0
0
1
1
1
1
0
0
2
0
0
0
0
0
0
0
0
0
0
0
26
10
8
0
90
0
0
0
3
0
0
0
3
9
0
0
0
0
0
1
4
3
0
1
16
5
1
1
0
0
0
4
1
1
0
0
0
1
1
1
22
11
5
12
5
26
4
12
1
2


1
26
29
33
7
63
13
6
9
7
19
18
21
7
29
35
17
53
18
30
15
30
32
0
0
86
0
75
452
22
27
63
51
9
19
50
0
9
24
98
35
213
14
20
16
17
96
34
83
106
11
27
35
4
10
10
20
15
39
45
35
14
1488
18
11
13
11
16
9
17
12
7
24
8
15
9
23
3
8
6
4


0
0
0
0
0
3
0
0
0
0
0
0
9
2
0
0
0
2
0
0
0
5
14
0
0
4
0
1
0
1
1
0
0
0
3
0
0
0
0
8
2
7
1
4
2
0
10
0
29
0
7
0
0
0
2
0
0
0
1
0
0
0
0
1
0
0
0
3
0
0
0
0
2
0
4
0
0
0
0
0
0


0
0
0
0
0
3
0
0
0
0
0
0
9
2
0
0
0
2
0
0
0
5
14
0
0
4
0
1
0
1
1
0
0
0
3
0
0
0
0
8
2
7
1
4
2
0
10
0
29
0
7
0
0
0
2
0
0
0
1
0
0
0
0
1
0
0
0
3
0
0
0
0
2
0
4
0
0
0
0
0
0


0
0
0
0
0
0
0
0
0
0
0
0
0
0
0
0
0
0
0
0
0
0
0
0
0
0
0
0
0
0
0
0
0
0
0
0
0
0
0
0
0
0
0
0
0
1
0
0
0
0
0
1
0
0
0
0
0
0
0
0
0
0
0
0
0
0
0
0
0
0
0
0
0
0
0
0
0
0
0
0
0


0
0
0
0
0
0
0
0
0
0
0
0
0
0
0
0
0
0
0
0
0
0
0
0
0
0
0
0
0
0
0
0
0
0
0
0
0
0
0
0
0
0
0
0
0
1
0
0
0
0
0
1
0
0
0
0
0
0
0
0
0
0
0
0
0
0
0
0
0
0
0
0
0
0
0
0
0
0
0
0
0


0
11
5
12
0
9
2
4
3
0
1
0
1
1
4
6
4
14
0
11
4
6
1
0
0
30
0
25
0
0
12
2
6
0
11
1
0
7
6
52
7
6
3
15
7
1
18
11
24
105
2
4
15
1
0
1
4
6
8
6
10
5
1
5
3
5
1
1
2
0
2
0
0
1
0
0
4
1
0
3
0


0
11
5
12
0
9
2
4
3
0
1
0
1
1
4
6
4
14
0
11
4
6
1
0
0
30
0
25
0
0
12
2
6
0
11
1
0
7
6
52
7
6
3
15
7
1
18
11
24
105
2
4
15
1
0
1
4
6
8
6
10
5
1
5
3
5
1
1
2
0
2
0
0
1
0
0
4
1
0
3
0


0
0
0
8
2
23
0
0
0
0
0
0
0
0
0
0
0
0
0
1
0
0
0
0
0
0
0
0
0
0
1
8
16
8
0
33
0
0
8
4
0
0
0
0
2
0
0
0
0
0
0
2
9
0
0
0
1
3
0
0
0
0
16
0
5
5
9
0
1
7
2
4
19
7
6
7
7
2
2
3
4


0
0
0
8
2
23
0
0
0
0
0
0
0
0
0
0
0
0
0
1
0
0
0
0
0
0
0
0
0
0
1
8
16
8
0
33
0
0
8
4
0
0
0
0
2
0
0
0
0
0
0
2
9
0
0
0
1
3
0
0
0
0
16
0
5
5
9
0
1
7
2
4
19
7
6
7
7
2
2
3
4


1
4
0
0
0
0
0
0
1
1
2
1
0
0
0
3
0
3
1
2
2
0
3
0
0
0
0
2
0
3
2
2
0
0
0
0
0
0
3
3
4
160
0
0
0
2
0
1
2
0
2
1
4
1
0
0
0
1
2
0
0
0
0
1
1
1
0
2
0
0
0
0
0
0
0
0
0
0
0
0
0


0
1
0
0
0
0
0
0
0
0
0
0
0
0
0
0
0
1
0
0
0
0
0
0
0
0
0
0
0
0
1
0
0
0
0
0
0
0
0
0
0
0
0
0
0
0
0
0
1
0
0
0
0
0
0
0
0
0
0
0
0
0
0
0
0
0
0
0
0
0
0
0
0
0
0
0
0
0
0
0
0


0
0
0
0
0
0
0
0
0
1
2
1
0
0
0
2
0
1
0
1
0
0
1
0
0
0
0
0
0
1
1
0
0
0
0
0
0
0
0
1
1
9
0
0
0
2
0
0
0
0
0
1
1
0
0
0
0
0
0
0
0
0
0
1
0
1
0
1
0
0
0
0
0
0
0
0
0
0
0
0
0


1
3
0
0
0
0
0
0
1
0
0
0
0
0
0
1
0
1
1
1
2
0
2
0
0
0
0
2
0
2
0
2
0
0
0
0
0
0
3
2
3
151
0
0
0
0
0
1
1
0
2
0
3
1
0
0
0
1
2
0
0
0
0
0
1
0
0
1
0
0
0
0
0
0
0
0
0
0
0
0
0


0
0
0
0
0
0
1
0
0
0
1
2
0
0
0
0
0
0
0
0
0
0
0
0
0
0
0
1
0
1
1
0
0
0
0
0
0
0
1
0
0
8
1
0
0
0
2
0
0
0
0
0
0
0
3
0
0
0
0
0
0
0
1324
0
0
0
0
1
0
0
0
0
1
0
0
0
0
0
0
0
0


0
0
0
0
0
0
1
0
0
0
1
2
0
0
0
0
0
0
0
0
0
0
0
0
0
0
0
1
0
1
1
0
0
0
0
0
0
0
1
0
0
8
1
0
0
0
2
0
0
0
0
0
0
0
3
0
0
0
0
0
0
0
1324
0
0
0
0
1
0
0
0
0
1
0
0
0
0
0
0
0
0


0
11
24
13
5
28
10
2
5
6
15
15
11
4
25
26
13
34
17
16
9
19
14
0
0
52
0
46
452
17
10
51
29
1
5
16
0
2
6
31
22
32
9
1
5
13
66
22
28
1
0
19
7
2
5
9
15
5
28
39
25
9
147
11
2
2
1
9
6
10
8
3
2
0
5
2
12
0
6
0
0


0
11
24
13
5
28
10
2
5
6
15
15
11
4
25
26
13
34
17
16
9
19
14
0
0
52
0
46
452
17
10
51
29
1
5
16
0
2
6
31
22
32
9
1
5
13
66
22
28
1
0
19
7
2
5
9
15
5
28
39
25
9
147
11
2
2
1
9
6
10
8
3
2
0
5
2
12
0
6
0
0


0
0
1
0
5
0
0
0
0
0
0
0
0
0
0
2
0
0
0
0
0
4
0
0
0
0
0
0
0
0
0
0
0
0
0
0
0
0
0
0
0
0
0
0
0
0
0
0
0
0
0
0
0
0
0
0
0
0
0
0
0
0
0
0
0
0
0
0
1
0
0
0
0
0
0
0
0
0
0
0
0


0
0
1
0
5
0
0
0
0
0
0
0
0
0
0
2
0
0
0
0
0
4
0
0
0
0
0
0
0
0
0
0
0
0
0
0
0
0
0
0
0
0
0
0
0
0
0
0
0
0
0
0
0
0
0
0
0
0
0
0
0
0
0
0
0
0
0
0
1
0
0
0
0
0
0
0
0
0
0
0
0


0
0
1
0
5
0
0
0
0
0
0
0
0
0
0
2
0
0
0
0
0
4
0
0
0
0
0
0
0
0
0
0
0
0
0
0
0
0
0
0
0
0
0
0
0
0
0
0
0
0
0
0
0
0
0
0
0
0
0
0
0
0
0
0
0
0
0
0
1
0
0
0
0
0
0
0
0
0
0
0
0


0
0
0
0
0
3
0
0
0
0
0
0
0
0
0
0
0
0
0
0
0
0
0
0
0
0
0
0
0
0
2
0
0
0
0
0
0
0
0
0
0
0
0
0
0
0
0
1
0
0
0
0
0
0
0
0
0
0
0
0
0
0
0
0
0
0
0
0
0
0
0
0
0
0
0
0
0
0
0
2
0


0
0
0
0
0
3
0
0
0
0
0
0
0
0
0
0
0
0
0
0
0
0
0
0
0
0
0
0
0
0
2
0
0
0
0
0
0
0
0
0
0
0
0
0
0
0
0
1
0
0
0
0
0
0
0
0
0
0
0
0
0
0
0
0
0
0
0
0
0
0
0
0
0
0
0
0
0
0
0
2
0


0
0
0
0
0
3
0
0
0
0
0
0
0
0
0
0
0
0
0
0
0
0
0
0
0
0
0
0
0
0
2
0
0
0
0
0
0
0
0
0
0
0
0
0
0
0
0
1
0
0
0
0
0
0
0
0
0
0
0
0
0
0
0
0
0
0
0
0
0
0
0
0
0
0
0
0
0
0
0
2
0


0
0
0
0
0
0
0
0
0
0
0
0
0
0
0
3
0
3
0
1
0
1
0
0
0
0
213
0
0
0
77
0
0
0
0
0
0
0
0
0
0
0
0
0
0
0
0
5
0
0
0
0
0
0
0
0
0
0
0
0
0
0
0
0
0
0
0
0
0
0
0
0
0
0
0
0
0
0
0
0
0


0
0
0
0
0
0
0
0
0
0
0
0
0
0
0
0
0
0
0
0
0
1
0
0
0
0
97
0
0
0
0
0
0
0
0
0
0
0
0
0
0
0
0
0
0
0
0
0
0
0
0
0
0
0
0
0
0
0
0
0
0
0
0
0
0
0
0
0
0
0
0
0
0
0
0
0
0
0
0
0
0


0
0
0
0
0
0
0
0
0
0
0
0
0
0
0
0
0
0
0
0
0
1
0
0
0
0
97
0
0
0
0
0
0
0
0
0
0
0
0
0
0
0
0
0
0
0
0
0
0
0
0
0
0
0
0
0
0
0
0
0
0
0
0
0
0
0
0
0
0
0
0
0
0
0
0
0
0
0
0
0
0


0
0
0
0
0
0
0
0
0
0
0
0
0
0
0
3
0
3
0
1
0
0
0
0
0
0
116
0
0
0
77
0
0
0
0
0
0
0
0
0
0
0
0
0
0
0
0
5
0
0
0
0
0
0
0
0
0
0
0
0
0
0
0
0
0
0
0
0
0
0
0
0
0
0
0
0
0
0
0
0
0


0
0
0
0
0
0
0
0
0
0
0
0
0
0
0
1
0
0
0
0
0
0
0
0
0
0
1
0
0
0
0
0
0
0
0
0
0
0
0
0
0
0
0
0
0
0
0
0
0
0
0
0
0
0
0
0
0
0
0
0
0
0
0
0
0
0
0
0
0
0
0
0
0
0
0
0
0
0
0
0
0


0
0
0
0
0
0
0
0
0
0
0
0
0
0
0
2
0
3
0
1
0
0
0
0
0
0
0
0
0
0
70
0
0
0
0
0
0
0
0
0
0
0
0
0
0
0
0
5
0
0
0
0
0
0
0
0
0
0
0
0
0
0
0
0
0
0
0
0
0
0
0
0
0
0
0
0
0
0
0
0
0


0
0
0
0
0
0
0
0
0
0
0
0
0
0
0
0
0
0
0
0
0
0
0
0
0
0
0
0
0
0
3
0
0
0
0
0
0
0
0
0
0
0
0
0
0
0
0
0
0
0
0
0
0
0
0
0
0
0
0
0
0
0
0
0
0
0
0
0
0
0
0
0
0
0
0
0
0
0
0
0
0


0
0
0
0
0
0
0
0
0
0
0
0
0
0
0
0
0
0
0
0
0
0
0
0
0
0
115
0
0
0
4
0
0
0
0
0
0
0
0
0
0
0
0
0
0
0
0
0
0
0
0
0
0
0
0
0
0
0
0
0
0
0
0
0
0
0
0
0
0
0
0
0
0
0
0
0
0
0
0
0
0


5
2
0
2
2
8
2
0
5
7
6
2
1
3
3
1
3
6
5
8
10
61
4
0
0
8
0
5
0
0
0
0
0
0
0
0
0
0
0
1
8
1
4
2
0
0
3
0
1
0
3
2
3
0
18
4
1
1
7
2
3
1
0
0
2
3
0
3
0
0
5
2
4
8
9
2
0
0
0
3
4


2
0
0
2
2
2
2
0
4
2
4
1
0
2
3
0
3
1
3
4
2
41
3
0
0
2
0
5
0
0
0
0
0
0
0
0
0
0
0
1
2
0
4
2
0
0
3
0
0
0
2
2
1
0
15
4
0
0
5
1
1
0
0
0
0
3
0
3
0
0
0
2
3
8
3
2
0
0
0
3
4


1
0
0
0
0
0
0
0
0
0
0
0
0
0
0
0
2
0
3
0
2
41
2
0
0
1
0
0
0
0
0
0
0
0
0
0
0
0
0
0
0
0
4
2
0
0
2
0
0
0
2
2
1
0
14
2
0
0
1
1
1
0
0
0
0
2
0
2
0
0
0
2
2
4
3
2
0
0
0
3
4


0
0
0
0
0
2
0
0
0
0
1
0
0
1
0
0
0
0
0
2
0
0
0
0
0
0
0
0
0
0
0
0
0
0
0
0
0
0
0
0
0
0
0
0
0
0
0
0
0
0
0
0
0
0
0
0
0
0
0
0
0
0
0
0
0
0
0
0
0
0
0
0
0
0
0
0
0
0
0
0
0


1
0
0
2
2
0
2
0
4
2
3
1
0
1
3
0
1
1
0
2
0
0
1
0
0
1
0
5
0
0
0
0
0
0
0
0
0
0
0
1
2
0
0
0
0
0
1
0
0
0
0
0
0
0
1
2
0
0
4
0
0
0
0
0
0
1
0
1
0
0
0
0
1
4
0
0
0
0
0
0
0


0
1
0
0
0
6
0
0
1
0
1
1
0
0
0
0
0
0
2
0
0
0
0
0
0
0
0
0
0
0
0
0
0
0
0
0
0
0
0
0
0
0
0
0
0
0
0
0
0
0
1
0
0
0
0
0
0
0
0
0
0
0
0
0
0
0
0
0
0
0
4
0
0
0
0
0
0
0
0
0
0


0
1
0
0
0
6
0
0
1
0
1
1
0
0
0
0
0
0
2
0
0
0
0
0
0
0
0
0
0
0
0
0
0
0
0
0
0
0
0
0
0
0
0
0
0
0
0
0
0
0
1
0
0
0
0
0
0
0
0
0
0
0
0
0
0
0
0
0
0
0
4
0
0
0
0
0
0
0
0
0
0


3
1
0
0
0
0
0
0
0
5
1
0
1
1
0
1
0
5
0
4
8
20
1
0
0
6
0
0
0
0
0
0
0
0
0
0
0
0
0
0
6
1
0
0
0
0
0
0
1
0
0
0
2
0
3
0
1
1
2
1
2
1
0
0
2
0
0
0
0
0
1
0
1
0
6
0
0
0
0
0
0


3
1
0
0
0
0
0
0
0
5
1
0
1
1
0
1
0
5
0
4
8
20
1
0
0
6
0
0
0
0
0
0
0
0
0
0
0
0
0
0
6
1
0
0
0
0
0
0
1
0
0
0
2
0
3
0
1
1
2
1
2
1
0
0
2
0
0
0
0
0
1
0
1
0
6
0
0
0
0
0
0


2
2
1
1
1
0
2
2
3
2
0
0
0
3
0
0
1
1
2
3
5
2
1
860
2055
68
2239
63
1778
1
2
15
11
14
9
0
378
0
0
0
3
3
18
21
10
4
5
100
0
0
1
10
8
0
2
7
0
5
4
13
11
3
12
1
4
3
4
6
6
572
2
0
1
1
1
4
3
0
0
0
0


2
2
1
1
1
0
2
2
3
2
0
0
0
3
0
0
1
1
2
3
5
2
1
860
2055
68
2239
63
1778
1
2
15
11
14
9
0
378
0
0
0
3
3
18
21
10
4
5
100
0
0
1
10
8
0
2
7
0
5
4
13
11
3
12
1
4
3
4
6
6
572
2
0
1
1
1
4
3
0
0
0
0


0
1
1
0
0
0
0
0
1
0
0
0
0
0
0
0
0
0
1
0
1
1
0
65
389
11
297
1
204
0
0
0
5
0
9
0
245
0
0
0
1
2
2
10
2
0
1
3
0
0
0
1
3
0
1
0
0
0
0
1
1
1
4
0
0
0
0
0
0
88
0
0
0
1
1
0
0
0
0
0
0


0
0
0
0
0
0
0
0
0
0
0
0
0
0
0
0
0
0
0
0
0
0
0
0
0
8
0
0
1
0
0
0
0
0
0
0
0
0
0
0
0
0
0
0
1
0
2
0
0
0
0
1
0
0
0
0
0
0
0
0
0
0
0
0
0
0
0
0
0
0
0
0
0
0
0
0
0
0
0
0
0


1
0
0
1
1
0
0
1
2
1
0
0
0
2
0
0
1
1
1
2
4
1
1
791
1661
48
1940
61
1435
1
1
14
6
14
0
0
132
0
0
0
2
1
11
10
6
4
2
95
0
0
0
8
4
0
1
7
0
5
4
7
10
2
7
1
3
3
4
6
6
469
2
0
0
0
0
3
3
0
0
0
0


0
0
0
0
0
0
0
0
0
0
0
0
0
0
0
0
0
0
0
0
0
0
0
0
0
0
0
0
0
0
0
0
0
0
0
0
0
0
0
0
0
0
0
0
0
0
0
0
0
0
0
0
1
0
0
0
0
0
0
0
0
0
0
0
0
0
0
0
0
0
0
0
0
0
0
0
0
0
0
0
0


1
1
0
0
0
0
2
1
0
1
0
0
0
1
0
0
0
0
0
1
0
0
0
4
5
1
2
1
138
0
1
1
0
0
0
0
1
0
0
0
0
0
5
1
1
0
0
2
0
0
1
0
0
0
0
0
0
0
0
5
0
0
1
0
1
0
0
0
0
15
0
0
1
0
0
1
0
0
0
0
0


1
2
4
4
0
1
1
1
1
1
1
0
0
1
2
1
5
4
3
1
18
9
10
0
0
62
0
14
0
0
0
0
0
0
0
0
0
0
3
26
9
4
6
8
1
1
20
0
1
0
2
0
0
0
0
2
4
4
4
6
2
2
0
0
0
0
0
6
0
0
0
0
5
1
4
0
0
0
1
0
0


0
0
3
2
0
1
1
0
1
0
1
0
0
1
0
0
4
4
0
1
12
4
7
0
0
8
0
7
0
0
0
0
0
0
0
0
0
0
3
13
3
1
6
7
1
0
8
0
0
0
1
0
0
0
0
1
2
2
1
6
2
1
0
0
0
0
0
3
0
0
0
0
0
0
0
0
0
0
0
0
0


0
0
3
2
0
1
1
0
1
0
1
0
0
1
0
0
4
4
0
1
12
4
7
0
0
8
0
7
0
0
0
0
0
0
0
0
0
0
3
13
3
1
6
7
1
0
8
0
0
0
1
0
0
0
0
1
2
2
1
6
2
1
0
0
0
0
0
3
0
0
0
0
0
0
0
0
0
0
0
0
0


1
2
1
2
0
0
0
1
0
1
0
0
0
0
2
1
1
0
3
0
6
5
3
0
0
54
0
7
0
0
0
0
0
0
0
0
0
0
0
13
6
3
0
1
0
1
12
0
1
0
1
0
0
0
0
1
2
2
3
0
0
1
0
0
0
0
0
3
0
0
0
0
5
1
4
0
0
0
1
0
0


1
2
1
2
0
0
0
1
0
1
0
0
0
0
2
1
1
0
2
0
5
4
3
0
0
52
0
7
0
0
0
0
0
0
0
0
0
0
0
11
6
2
0
1
0
1
11
0
1
0
1
0
0
0
0
0
2
2
2
0
0
1
0
0
0
0
0
3
0
0
0
0
5
1
4
0
0
0
1
0
0


0
0
0
0
0
0
0
0
0
0
0
0
0
0
0
0
0
0
1
0
1
0
0
0
0
0
0
0
0
0
0
0
0
0
0
0
0
0
0
1
0
0
0
0
0
0
0
0
0
0
0
0
0
0
0
0
0
0
0
0
0
0
0
0
0
0
0
0
0
0
0
0
0
0
0
0
0
0
0
0
0


0
0
0
0
0
0
0
0
0
0
0
0
0
0
0
0
0
0
0
0
0
1
0
0
0
2
0
0
0
0
0
0
0
0
0
0
0
0
0
1
0
1
0
0
0
0
1
0
0
0
0
0
0
0
0
1
0
0
1
0
0
0
0
0
0
0
0
0
0
0
0
0
0
0
0
0
0
0
0
0
0


0
0
3
0
0
0
0
0
0
1
0
0
0
0
2
3
0
0
0
0
2
1
0
0
0
0
72
0
152
0
41
0
41
0
0
0
0
0
0
0
0
0
0
0
0
0
0
2
0
0
0
0
0
0
0
0
0
0
0
2
0
0
0
0
0
0
0
0
0
0
0
0
0
0
0
1
0
0
0
0
0


0
0
3
0
0
0
0
0
0
1
0
0
0
0
2
3
0
0
0
0
2
1
0
0
0
0
72
0
151
0
39
0
41
0
0
0
0
0
0
0
0
0
0
0
0
0
0
2
0
0
0
0
0
0
0
0
0
0
0
2
0
0
0
0
0
0
0
0
0
0
0
0
0
0
0
1
0
0
0
0
0


0
0
0
0
0
0
0
0
0
0
0
0
0
0
2
0
0
0
0
0
0
0
0
0
0
0
0
0
0
0
2
0
39
0
0
0
0
0
0
0
0
0
0
0
0
0
0
0
0
0
0
0
0
0
0
0
0
0
0
0
0
0
0
0
0
0
0
0
0
0
0
0
0
0
0
0
0
0
0
0
0


0
0
0
0
0
0
0
0
0
1
0
0
0
0
0
3
0
0
0
0
2
1
0
0
0
0
71
0
150
0
36
0
0
0
0
0
0
0
0
0
0
0
0
0
0
0
0
2
0
0
0
0
0
0
0
0
0
0
0
2
0
0
0
0
0
0
0
0
0
0
0
0
0
0
0
1
0
0
0
0
0


0
0
3
0
0
0
0
0
0
0
0
0
0
0
0
0
0
0
0
0
0
0
0
0
0
0
1
0
1
0
1
0
2
0
0
0
0
0
0
0
0
0
0
0
0
0
0
0
0
0
0
0
0
0
0
0
0
0
0
0
0
0
0
0
0
0
0
0
0
0
0
0
0
0
0
0
0
0
0
0
0


0
0
0
0
0
0
0
0
0
0
0
0
0
0
0
0
0
0
0
0
0
0
0
0
0
0
0
0
1
0
2
0
0
0
0
0
0
0
0
0
0
0
0
0
0
0
0
0
0
0
0
0
0
0
0
0
0
0
0
0
0
0
0
0
0
0
0
0
0
0
0
0
0
0
0
0
0
0
0
0
0


0
0
0
0
0
0
0
0
0
0
0
0
0
0
0
0
0
0
0
0
0
0
0
0
0
0
0
0
1
0
2
0
0
0
0
0
0
0
0
0
0
0
0
0
0
0
0
0
0
0
0
0
0
0
0
0
0
0
0
0
0
0
0
0
0
0
0
0
0
0
0
0
0
0
0
0
0
0
0
0
0


0
0
0
0
0
0
0
0
0
0
0
0
0
0
0
0
0
0
0
0
0
0
0
0
0
2
0
0
0
0
0
59
0
0
0
1
0
0
0
0
0
2
0
0
0
0
0
0
0
0
0
0
0
0
0
0
0
0
0
0
0
0
0
0
0
0
0
0
0
0
0
0
0
0
0
1
1
1
0
0
0


0
0
0
0
0
0
0
0
0
0
0
0
0
0
0
0
0
0
0
0
0
0
0
0
0
2
0
0
0
0
0
59
0
0
0
1
0
0
0
0
0
2
0
0
0
0
0
0
0
0
0
0
0
0
0
0
0
0
0
0
0
0
0
0
0
0
0
0
0
0
0
0
0
0
0
1
1
1
0
0
0


0
0
0
0
0
0
0
0
0
0
0
0
0
0
0
0
0
0
0
0
0
0
0
0
0
2
0
0
0
0
0
59
0
0
0
1
0
0
0
0
0
2
0
0
0
0
0
0
0
0
0
0
0
0
0
0
0
0
0
0
0
0
0
0
0
0
0
0
0
0
0
0
0
0
0
1
1
1
0
0
0


0
0
0
0
0
0
0
0
0
0
0
0
0
0
0
0
0
0
0
0
0
0
0
0
24
0
0
0
0
0
0
0
0
0
0
0
0
0
0
0
0
0
0
0
0
0
0
0
0
0
0
0
0
0
0
0
0
0
0
0
0
0
0
0
0
0
0
0
0
0
0
0
0
0
0
0
0
0
0
0
0


0
0
0
0
0
0
0
0
0
0
0
0
0
0
0
0
0
0
0
0
0
0
0
0
24
0
0
0
0
0
0
0
0
0
0
0
0
0
0
0
0
0
0
0
0
0
0
0
0
0
0
0
0
0
0
0
0
0
0
0
0
0
0
0
0
0
0
0
0
0
0
0
0
0
0
0
0
0
0
0
0


0
0
0
0
0
0
0
0
0
0
0
0
0
0
0
0
0
0
0
0
0
0
0
0
24
0
0
0
0
0
0
0
0
0
0
0
0
0
0
0
0
0
0
0
0
0
0
0
0
0
0
0
0
0
0
0
0
0
0
0
0
0
0
0
0
0
0
0
0
0
0
0
0
0
0
0
0
0
0
0
0


0
0
0
0
0
0
0
0
0
0
0
0
0
0
0
0
0
0
0
0
0
0
0
0
0
0
0
0
0
0
1
0
0
0
0
0
0
0
0
0
0
0
0
0
0
0
0
0
0
0
0
0
0
0
0
0
0
0
0
0
0
0
0
0
0
0
0
0
0
0
0
0
0
0
0
0
0
0
0
0
0


0
0
0
0
0
0
0
0
0
0
0
0
0
0
0
0
0
0
0
0
0
0
0
0
0
0
0
0
0
0
1
0
0
0
0
0
0
0
0
0
0
0
0
0
0
0
0
0
0
0
0
0
0
0
0
0
0
0
0
0
0
0
0
0
0
0
0
0
0
0
0
0
0
0
0
0
0
0
0
0
0


0
0
0
0
0
0
0
0
0
0
0
0
0
0
0
0
0
0
0
0
0
0
0
0
0
0
0
0
0
0
1
0
0
0
0
0
0
0
0
0
0
0
0
0
0
0
0
0
0
0
0
0
0
0
0
0
0
0
0
0
0
0
0
0
0
0
0
0
0
0
0
0
0
0
0
0
0
0
0
0
0


7
7
26
61
11
147
14
17
10
15
5
30
16
10
16
17
16
32
24
73
39
6
38
0
0
24
0
33
0
44
63
443
86
127
4
163
1
13
36
109
29
23
26
65
122
46
37
32
100
0
15
45
34
14
18
36
24
53
61
24
41
20
84
2
73
52
25
8
25
22
51
83
98
60
16
86
47
46
53
42
25


7
6
26
59
9
147
14
17
10
15
5
30
16
10
16
16
15
27
24
71
38
6
38
0
0
24
0
33
0
44
62
443
60
127
4
155
1
13
33
106
14
20
22
63
121
45
33
31
100
0
15
44
34
14
18
36
24
53
60
23
40
20
81
2
72
51
23
8
25
22
49
83
96
55
16
84
47
46
53
39
25


7
6
26
59
9
147
14
17
10
15
5
30
16
10
16
16
15
27
24
71
38
6
38
0
0
24
0
33
0
44
62
443
60
127
4
155
1
13
33
106
14
20
22
63
121
45
33
31
100
0
15
44
34
14
18
36
24
53
60
23
40
20
81
2
72
51
23
8
25
22
49
83
96
55
16
84
47
46
53
39
25


0
0
0
0
1
0
0
0
0
0
0
0
0
0
0
0
1
5
0
0
0
0
0
0
0
0
0
0
0
0
0
0
0
0
0
8
0
0
0
1
15
1
2
2
0
1
0
0
0
0
0
0
0
0
0
0
0
0
1
1
1
0
2
0
1
1
2
0
0
0
2
0
2
5
0
2
0
0
0
1
0


0
0
0
0
1
0
0
0
0
0
0
0
0
0
0
0
1
5
0
0
0
0
0
0
0
0
0
0
0
0
0
0
0
0
0
8
0
0
0
1
15
1
2
2
0
1
0
0
0
0
0
0
0
0
0
0
0
0
1
1
1
0
2
0
1
1
2
0
0
0
2
0
2
5
0
2
0
0
0
1
0


0
1
0
2
1
0
0
0
0
0
0
0
0
0
0
1
0
0
0
2
1
0
0
0
0
0
0
0
0
0
1
0
26
0
0
0
0
0
3
2
0
2
2
0
1
0
4
1
0
0
0
1
0
0
0
0
0
0
0
0
0
0
1
0
0
0
0
0
0
0
0
0
0
0
0
0
0
0
0
2
0


0
1
0
2
1
0
0
0
0
0
0
0
0
0
0
1
0
0
0
2
1
0
0
0
0
0
0
0
0
0
1
0
26
0
0
0
0
0
3
2
0
2
2
0
1
0
4
1
0
0
0
1
0
0
0
0
0
0
0
0
0
0
1
0
0
0
0
0
0
0
0
0
0
0
0
0
0
0
0
2
0


16
27
54
18
14
17
3
14
3
6
21
16
31
13
28
58
81
66
43
58
47
73
60
0
0
106
0
378
0
39
51
124
112
14
37
36
0
44
54
387
231
83
57
72
70
36
385
206
83
0
52
64
25
41
33
106
61
84
79
120
71
58
47
21
31
41
7
29
14
11
65
48
85
33
17
25
57
32
8
13
29


14
18
30
5
8
11
1
8
2
6
15
10
20
8
11
28
51
18
18
25
24
33
25
0
0
58
0
196
0
18
20
25
89
8
14
16
0
24
23
193
100
40
25
36
30
14
175
126
40
0
28
29
11
24
12
67
36
42
52
51
41
37
9
12
13
26
5
14
11
3
36
10
32
11
3
6
23
10
1
8
7


14
18
30
5
8
11
1
8
2
6
15
10
20
8
11
28
51
18
18
25
24
33
25
0
0
58
0
196
0
18
20
25
89
8
14
16
0
24
23
193
100
40
25
36
30
14
175
126
40
0
28
29
11
24
12
67
36
42
52
51
41
37
9
12
13
26
5
14
11
3
36
10
32
11
3
6
23
10
1
8
7


0
0
0
0
0
0
0
0
0
0
0
0
1
0
0
0
0
2
1
0
4
4
1
0
0
7
0
0
0
0
0
0
0
0
0
0
0
0
0
2
3
0
0
0
0
0
1
0
0
0
0
3
0
0
0
0
0
1
1
1
0
0
0
0
0
0
0
2
0
0
0
0
0
0
0
0
0
0
0
0
0


0
0
0
0
0
0
0
0
0
0
0
0
1
0
0
0
0
2
1
0
4
4
1
0
0
7
0
0
0
0
0
0
0
0
0
0
0
0
0
2
3
0
0
0
0
0
1
0
0
0
0
3
0
0
0
0
0
1
1
1
0
0
0
0
0
0
0
2
0
0
0
0
0
0
0
0
0
0
0
0
0


0
2
3
9
2
5
0
1
0
0
2
2
3
0
4
9
4
11
2
17
3
11
13
0
0
24
0
82
0
7
16
98
7
4
5
16
0
16
22
77
46
15
12
8
19
5
99
47
16
0
13
2
2
1
3
7
12
15
10
8
8
8
32
1
12
4
1
4
0
2
6
16
35
22
9
16
23
15
7
3
13


0
2
1
9
1
5
0
1
0
0
0
2
1
0
2
9
0
4
2
17
0
3
3
0
0
0
0
1
0
1
5
97
7
4
5
15
0
0
1
15
4
0
2
0
15
1
14
25
6
0
11
0
1
1
2
3
6
12
4
2
8
8
32
1
11
4
1
4
0
0
3
15
35
22
9
16
23
14
7
3
13


0
0
0
0
0
0
0
0
0
0
0
0
0
0
0
0
0
0
0
0
0
0
0
0
0
0
0
0
0
0
0
0
0
0
0
0
0
0
0
0
2
0
0
0
0
0
0
0
0
0
0
0
0
0
0
0
0
0
0
0
0
0
0
0
0
0
0
0
0
0
0
0
0
0
0
0
0
0
0
0
0


0
0
2
0
1
0
0
0
0
0
2
0
2
0
2
0
4
7
0
0
3
8
10
0
0
24
0
81
0
6
11
1
0
0
0
1
0
16
21
62
40
15
10
8
4
4
85
22
10
0
2
2
1
0
1
4
6
3
6
6
0
0
0
0
1
0
0
0
0
2
3
1
0
0
0
0
0
1
0
0
0


0
0
0
0
0
0
0
1
0
0
0
0
0
0
0
0
2
2
0
0
1
2
0
0
0
0
0
0
0
0
1
0
0
0
0
0
0
0
0
0
1
1
0
0
0
0
1
0
0
0
0
0
0
0
0
1
0
0
0
0
0
0
0
0
0
0
0
0
0
0
0
1
0
0
0
0
2
0
0
0
0


0
0
0
0
0
0
0
1
0
0
0
0
0
0
0
0
2
2
0
0
1
2
0
0
0
0
0
0
0
0
1
0
0
0
0
0
0
0
0
0
1
1
0
0
0
0
1
0
0
0
0
0
0
0
0
1
0
0
0
0
0
0
0
0
0
0
0
0
0
0
0
1
0
0
0
0
2
0
0
0
0


2
7
21
4
4
1
2
4
1
0
4
4
7
5
13
21
24
33
22
16
15
23
21
0
0
17
0
100
0
14
14
1
16
2
18
4
0
4
9
115
81
27
20
28
21
17
109
33
27
0
11
30
12
16
18
31
13
26
16
60
22
13
6
8
6
11
1
9
3
6
23
21
18
0
5
3
9
7
0
2
9


2
7
21
4
4
1
2
4
1
0
4
4
7
5
13
21
24
33
22
16
15
23
21
0
0
17
0
100
0
14
14
1
16
2
18
4
0
4
9
115
81
27
20
28
21
17
109
33
27
0
11
30
12
16
18
31
13
26
16
60
22
13
6
8
6
11
1
9
3
6
23
21
18
0
5
3
9
7
0
2
9


0
0
1
0
0
0
0
0
0
0
0
0
1
0
0
1
0
0
0
0
0
0
0
0
0
0
0
0
0
0
0
0
0
0
0
0
0
0
0
0
0
0
0
0
0
0
0
0
0
0
0
0
0
0
0
0
0
0
4
1
1
0
0
0
0
0
0
0
0
0
0
0
0
0
0
0
0
0
0
0
0


0
0
0
0
0
0
0
0
0
0
0
0
0
0
0
1
0
0
0
0
0
0
0
0
0
0
0
0
0
0
0
0
0
0
0
0
0
0
0
0
0
0
0
0
0
0
0
0
0
0
0
0
0
0
0
0
0
0
0
1
0
0
0
0
0
0
0
0
0
0
0
0
0
0
0
0
0
0
0
0
0


0
0
0
0
0
0
0
0
0
0
0
0
0
0
0
1
0
0
0
0
0
0
0
0
0
0
0
0
0
0
0
0
0
0
0
0
0
0
0
0
0
0
0
0
0
0
0
0
0
0
0
0
0
0
0
0
0
0
0
1
0
0
0
0
0
0
0
0
0
0
0
0
0
0
0
0
0
0
0
0
0


0
0
1
0
0
0
0
0
0
0
0
0
1
0
0
0
0
0
0
0
0
0
0
0
0
0
0
0
0
0
0
0
0
0
0
0
0
0
0
0
0
0
0
0
0
0
0
0
0
0
0
0
0
0
0
0
0
0
4
0
1
0
0
0
0
0
0
0
0
0
0
0
0
0
0
0
0
0
0
0
0


0
0
0
0
0
0
0
0
0
0
0
0
0
0
0
0
0
0
0
0
0
0
0
0
0
0
0
0
0
0
0
0
0
0
0
0
0
0
0
0
0
0
0
0
0
0
0
0
0
0
0
0
0
0
0
0
0
0
4
0
1
0
0
0
0
0
0
0
0
0
0
0
0
0
0
0
0
0
0
0
0


0
0
1
0
0
0
0
0
0
0
0
0
1
0
0
0
0
0
0
0
0
0
0
0
0
0
0
0
0
0
0
0
0
0
0
0
0
0
0
0
0
0
0
0
0
0
0
0
0
0
0
0
0
0
0
0
0
0
0
0
0
0
0
0
0
0
0
0
0
0
0
0
0
0
0
0
0
0
0
0
0


0
0
0
0
0
0
0
0
0
0
2
0
0
0
0
1
0
0
0
0
1
7
0
0
0
0
0
1
0
0
0
0
0
0
0
0
0
0
0
0
1
1
0
0
0
0
1
6
1
0
0
2
1
1
0
0
0
0
1
1
0
1
0
0
0
0
0
0
0
0
0
0
0
0
0
0
0
1
0
0
0


0
0
0
0
0
0
0
0
0
0
2
0
0
0
0
1
0
0
0
0
1
7
0
0
0
0
0
1
0
0
0
0
0
0
0
0
0
0
0
0
1
1
0
0
0
0
1
6
1
0
0
2
1
1
0
0
0
0
1
1
0
1
0
0
0
0
0
0
0
0
0
0
0
0
0
0
0
1
0
0
0


0
0
0
0
0
0
0
0
0
0
2
0
0
0
0
1
0
0
0
0
1
7
0
0
0
0
0
1
0
0
0
0
0
0
0
0
0
0
0
0
1
1
0
0
0
0
1
6
1
0
0
2
1
1
0
0
0
0
1
1
0
1
0
0
0
0
0
0
0
0
0
0
0
0
0
0
0
1
0
0
0


10
105
144
52
99
104
33
27
21
22
45
43
41
23
99
120
106
99
91
87
87
79
93
119
0
218
100
103
131
117
150
133
449
365
35
55
94
44
56
147
112
89
62
167
144
67
95
140
112
0
38
99
44
68
44
93
70
76
139
129
70
59
60
19
27
76
15
30
17
19
70
81
95
123
21
50
39
71
51
52
21


0
2
0
0
1
0
0
0
0
0
0
0
2
0
0
2
0
0
3
1
0
0
0
0
0
1
0
0
0
1
0
0
0
0
0
0
0
0
0
0
0
0
2
1
0
0
0
0
0
0
0
1
0
5
1
0
6
0
7
0
4
0
1
5
0
1
0
0
0
0
6
3
0
1
0
2
3
1
0
0
0


0
2
0
0
1
0
0
0
0
0
0
0
2
0
0
2
0
0
3
1
0
0
0
0
0
1
0
0
0
1
0
0
0
0
0
0
0
0
0
0
0
0
2
1
0
0
0
0
0
0
0
1
0
5
1
0
6
0
7
0
4
0
1
5
0
1
0
0
0
0
6
3
0
1
0
2
3
1
0
0
0


6
1
102
25
58
62
17
12
5
9
28
21
24
17
69
4
82
64
63
59
59
46
59
54
0
161
0
1
0
0
85
41
4
247
0
17
0
1
1
4
77
0
1
2
103
0
3
1
72
0
1
49
20
39
16
2
40
1
87
1
0
0
0
9
0
3
0
1
4
0
20
2
1
1
5
0
0
22
0
16
0


0
0
0
0
0
0
1
0
0
0
0
0
0
0
0
0
0
0
1
0
0
0
0
0
0
0
0
0
0
0
0
0
0
0
0
0
0
0
0
0
0
0
0
0
0
0
0
0
0
0
0
0
0
0
0
0
0
0
0
0
0
0
0
0
0
0
0
0
0
0
0
0
0
0
0
0
0
0
0
0
0


6
0
100
24
58
57
15
12
5
9
28
19
23
17
69
4
82
64
61
59
58
46
58
54
0
158
0
1
0
0
85
41
3
241
0
17
0
0
1
2
77
0
1
2
102
0
2
1
71
0
1
47
20
39
15
2
39
0
87
1
0
0
0
9
0
2
0
0
4
0
20
2
0
0
5
0
0
22
0
16
0


0
1
2
1
0
5
1
0
0
0
0
2
1
0
0
0
0
0
1
0
1
0
1
0
0
3
0
0
0
0
0
0
1
6
0
0
0
1
0
2
0
0
0
0
1
0
1
0
1
0
0
2
0
0
1
0
1
1
0
0
0
0
0
0
0
1
0
1
0
0
0
0
1
1
0
0
0
0
0
0
0


0
0
0
0
0
0
0
0
0
0
0
0
0
0
0
0
0
0
0
0
0
0
0
0
0
0
99
0
123
0
0
0
0
0
0
0
0
0
0
0
0
0
0
0
0
0
0
0
0
0
0
0
0
0
0
0
0
0
0
0
0
0
0
0
0
0
0
0
0
0
0
0
0
0
0
0
0
0
0
0
0


0
0
0
0
0
0
0
0
0
0
0
0
0
0
0
0
0
0
0
0
0
0
0
0
0
0
99
0
123
0
0
0
0
0
0
0
0
0
0
0
0
0
0
0
0
0
0
0
0
0
0
0
0
0
0
0
0
0
0
0
0
0
0
0
0
0
0
0
0
0
0
0
0
0
0
0
0
0
0
0
0


0
0
2
2
3
2
4
0
3
0
1
2
1
1
5
3
2
8
6
2
1
2
2
0
0
7
0
0
0
5
13
12
32
15
0
4
0
0
1
9
1
2
2
13
1
1
8
7
4
0
0
4
3
2
4
1
2
2
4
0
0
0
2
0
1
0
0
1
0
0
2
0
0
13
5
0
2
7
1
3
0


0
0
2
2
3
2
4
0
3
0
1
2
1
1
5
3
2
8
6
2
1
2
2
0
0
7
0
0
0
5
13
12
32
15
0
4
0
0
1
9
1
2
2
13
1
1
8
7
4
0
0
4
3
2
4
1
2
2
4
0
0
0
2
0
1
0
0
1
0
0
2
0
0
13
5
0
2
7
1
3
0


0
0
0
0
0
0
0
0
0
0
0
0
0
0
0
0
0
0
0
0
0
0
0
0
0
2
0
0
0
0
0
0
0
0
0
0
0
0
0
0
0
0
0
0
0
0
0
0
0
0
0
0
0
0
0
0
0
0
0
0
0
0
0
0
0
0
0
0
0
0
0
0
0
0
0
0
0
0
0
0
0


0
0
0
0
0
0
0
0
0
0
0
0
0
0
0
0
0
0
0
0
0
0
0
0
0
2
0
0
0
0
0
0
0
0
0
0
0
0
0
0
0
0
0
0
0
0
0
0
0
0
0
0
0
0
0
0
0
0
0
0
0
0
0
0
0
0
0
0
0
0
0
0
0
0
0
0
0
0
0
0
0


2
2
0
6
2
0
0
0
0
0
0
0
0
0
0
1
1
0
0
7
5
2
1
0
0
1
0
7
8
2
15
23
65
0
0
2
93
12
5
14
5
1
4
6
10
3
3
15
3
0
0
4
2
1
2
1
0
2
0
1
0
0
1
0
0
3
0
0
2
0
1
1
1
6
1
0
3
6
0
1
1


2
2
0
6
2
0
0
0
0
0
0
0
0
0
0
1
1
0
0
7
5
2
1
0
0
1
0
7
8
2
15
23
65
0
0
2
93
12
5
14
5
1
4
6
10
3
3
15
3
0
0
4
2
1
2
1
0
2
0
1
0
0
1
0
0
3
0
0
2
0
1
1
1
6
1
0
3
6
0
1
1


0
1
0
0
0
0
0
0
0
0
4
0
0
0
0
1
0
0
2
3
0
0
0
0
0
0
0
0
0
0
0
0
0
0
0
0
0
0
1
0
0
1
0
0
0
0
0
0
0
0
0
0
0
0
0
2
2
0
1
1
1
0
0
1
0
3
0
1
0
0
0
0
2
0
0
0
0
0
1
0
0


0
1
0
0
0
0
0
0
0
0
4
0
0
0
0
1
0
0
2
3
0
0
0
0
0
0
0
0
0
0
0
0
0
0
0
0
0
0
1
0
0
1
0
0
0
0
0
0
0
0
0
0
0
0
0
2
2
0
1
1
1
0
0
1
0
3
0
1
0
0
0
0
2
0
0
0
0
0
1
0
0


2
99
40
19
35
40
12
15
13
13
12
20
14
5
25
109
21
27
17
15
22
29
31
65
0
46
1
95
0
109
37
57
348
103
35
32
1
31
48
120
29
85
53
145
30
63
81
117
33
0
37
41
19
21
21
87
20
71
40
126
65
59
56
4
26
66
15
27
11
19
41
75
91
102
10
48
31
35
49
32
20


2
99
40
19
35
40
12
15
13
13
12
20
14
5
25
109
21
27
17
15
22
29
31
65
0
46
1
95
0
109
37
57
348
103
35
32
1
31
48
120
29
85
53
145
30
63
81
117
33
0
37
41
19
21
21
87
20
71
40
126
65
59
56
4
26
66
15
27
11
19
41
75
91
102
10
48
31
35
49
32
20


10
13
9
3
7
4
2
9
8
1
2
7
8
6
14
38
21
14
9
33
46
52
40
0
0
182
0
42
1
14
12
24
54
0
1
14
0
10
11
42
53
31
6
16
9
15
52
51
19
0
6
6
4
9
8
1
13
7
10
16
23
4
1
1
1
6
1
1
3
1
4
3
11
4
4
0
1
2
4
0
1


9
13
6
3
7
2
2
9
0
1
0
7
8
6
14
33
18
14
8
31
42
47
36
0
0
177
0
37
1
12
10
24
6
0
1
7
0
9
7
40
47
29
6
14
8
13
50
45
17
0
5
6
0
8
8
0
13
6
8
16
22
2
1
0
0
5
1
1
0
0
3
3
9
4
1
0
1
2
2
0
1


9
13
6
3
7
2
2
9
0
1
0
7
8
6
14
33
18
14
8
31
42
47
36
0
0
177
0
37
1
12
10
24
6
0
1
7
0
9
7
40
47
29
6
14
8
13
50
45
17
0
5
6
0
8
8
0
13
6
8
16
22
2
1
0
0
5
1
1
0
0
3
3
9
4
1
0
1
2
2
0
1


0
0
0
0
0
0
0
0
0
0
0
0
0
0
0
2
0
0
0
0
0
0
0
0
0
0
0
0
0
0
0
0
0
0
0
1
0
0
0
0
0
1
0
0
0
0
0
0
0
0
0
0
0
0
0
0
0
0
0
0
0
0
0
0
0
0
0
0
1
0
0
0
0
0
0
0
0
0
0
0
0


0
0
0
0
0
0
0
0
0
0
0
0
0
0
0
2
0
0
0
0
0
0
0
0
0
0
0
0
0
0
0
0
0
0
0
1
0
0
0
0
0
1
0
0
0
0
0
0
0
0
0
0
0
0
0
0
0
0
0
0
0
0
0
0
0
0
0
0
1
0
0
0
0
0
0
0
0
0
0
0
0


0
0
0
0
0
0
0
0
0
0
0
0
0
0
0
0
0
0
0
0
0
4
0
0
0
0
0
0
0
0
0
0
0
0
0
0
0
0
0
0
4
0
0
0
0
0
0
0
0
0
0
0
0
0
0
0
0
0
0
0
0
0
0
0
0
0
0
0
0
0
0
0
2
0
3
0
0
0
0
0
0


0
0
0
0
0
0
0
0
0
0
0
0
0
0
0
0
0
0
0
0
0
4
0
0
0
0
0
0
0
0
0
0
0
0
0
0
0
0
0
0
4
0
0
0
0
0
0
0
0
0
0
0
0
0
0
0
0
0
0
0
0
0
0
0
0
0
0
0
0
0
0
0
2
0
3
0
0
0
0
0
0


1
0
3
0
0
2
0
0
8
0
2
0
0
0
0
3
3
0
1
2
4
1
4
0
0
5
0
5
0
2
2
0
48
0
0
6
0
1
4
2
2
1
0
2
1
2
2
6
2
0
1
0
4
1
0
1
0
1
2
0
1
2
0
1
1
1
0
0
2
1
1
0
0
0
0
0
0
0
2
0
0


1
0
3
0
0
2
0
0
8
0
2
0
0
0
0
3
3
0
1
2
4
1
4
0
0
5
0
5
0
2
2
0
48
0
0
6
0
1
4
2
2
1
0
2
1
2
2
6
2
0
1
0
4
1
0
1
0
1
2
0
1
2
0
1
1
1
0
0
2
1
1
0
0
0
0
0
0
0
2
0
0


6
28
75
10
55
21
76
10
29
19
95
34
95
37
54
57
96
75
136
38
36
113
74
0
0
71
0
11
0
7
3
102
23
41
4
32
18
2
9
38
32
51
19
8
4
9
30
10
9
0
16
34
14
22
43
28
33
11
64
43
28
19
107
16
6
13
3
12
4
8
31
10
21
1
8
5
7
6
2
5
1


5
22
63
6
41
13
38
8
16
14
80
17
79
24
42
35
63
66
102
31
30
85
65
0
0
67
0
11
0
4
1
0
9
2
4
11
0
0
7
28
26
33
15
4
1
8
23
4
8
0
9
22
8
15
24
18
17
7
39
36
23
10
0
11
0
5
0
7
2
0
9
0
0
0
0
0
0
0
0
0
0


5
14
60
4
33
8
29
7
16
12
70
15
68
22
38
28
58
54
86
29
22
75
56
0
0
55
0
11
0
4
1
0
9
2
4
8
0
0
7
28
25
24
14
4
1
2
23
4
7
0
7
17
8
15
24
18
17
7
37
36
19
6
0
11
0
5
0
7
1
0
8
0
0
0
0
0
0
0
0
0
0


0
0
0
2
3
4
9
0
0
1
0
1
5
0
0
0
0
1
3
0
0
6
0
0
0
0
0
0
0
0
0
0
0
0
0
1
0
0
0
0
0
8
1
0
0
6
0
0
1
0
2
1
0
0
0
0
0
0
0
0
4
4
0
0
0
0
0
0
1
0
1
0
0
0
0
0
0
0
0
0
0


0
8
3
0
5
1
0
1
0
1
10
1
6
2
4
7
5
11
13
2
8
4
9
0
0
12
0
0
0
0
0
0
0
0
0
2
0
0
0
0
1
1
0
0
0
0
0
0
0
0
0
4
0
0
0
0
0
0
2
0
0
0
0
0
0
0
0
0
0
0
0
0
0
0
0
0
0
0
0
0
0


0
0
0
0
0
0
0
0
0
0
0
0
0
0
0
0
0
0
0
0
0
0
0
0
0
0
0
0
0
0
0
0
0
0
0
0
0
0
0
0
0
0
0
0
0
0
0
0
0
0
0
0
0
0
0
0
0
1
0
0
0
0
0
0
0
0
0
0
0
0
0
0
0
0
0
0
0
0
0
0
0


0
0
0
0
0
0
0
0
0
0
0
0
0
0
0
0
0
0
0
0
0
0
0
0
0
0
0
0
0
0
0
0
0
0
0
0
0
0
0
0
0
0
0
0
0
0
0
0
0
0
0
0
0
0
0
0
0
1
0
0
0
0
0
0
0
0
0
0
0
0
0
0
0
0
0
0
0
0
0
0
0


1
0
0
0
2
3
0
0
0
1
2
2
1
2
2
0
1
0
2
0
0
7
0
0
0
0
0
0
0
0
0
0
11
2
0
3
0
0
0
1
1
1
2
2
0
1
0
0
0
0
0
2
0
0
5
0
1
0
2
0
0
1
0
0
3
6
1
0
0
8
8
2
8
0
0
3
1
2
0
4
0


1
0
0
0
2
3
0
0
0
1
2
2
1
2
2
0
1
0
2
0
0
7
0
0
0
0
0
0
0
0
0
0
11
2
0
3
0
0
0
1
1
1
2
2
0
1
0
0
0
0
0
2
0
0
5
0
1
0
2
0
0
1
0
0
3
6
1
0
0
8
8
2
8
0
0
3
1
2
0
4
0


0
0
0
0
0
1
0
0
0
0
0
0
0
0
0
0
5
1
0
0
0
0
0
0
0
0
0
0
0
0
0
0
0
0
0
3
0
0
0
0
0
0
0
0
0
0
0
0
1
0
1
0
0
0
0
0
0
0
0
0
0
0
0
0
0
0
0
0
1
0
0
0
0
0
0
0
1
0
0
0
0


0
0
0
0
0
1
0
0
0
0
0
0
0
0
0
0
5
1
0
0
0
0
0
0
0
0
0
0
0
0
0
0
0
0
0
3
0
0
0
0
0
0
0
0
0
0
0
0
1
0
1
0
0
0
0
0
0
0
0
0
0
0
0
0
0
0
0
0
1
0
0
0
0
0
0
0
1
0
0
0
0


0
0
0
0
0
0
0
0
1
0
0
0
0
0
0
10
0
1
1
0
3
1
0
0
0
0
0
0
0
0
0
0
0
0
0
0
0
0
0
0
0
0
0
0
0
0
0
0
0
0
0
2
0
0
0
0
0
0
0
0
0
0
0
0
0
0
0
0
0
0
0
0
0
0
0
0
0
0
0
0
0


0
0
0
0
0
0
0
0
1
0
0
0
0
0
0
10
0
1
1
0
3
1
0
0
0
0
0
0
0
0
0
0
0
0
0
0
0
0
0
0
0
0
0
0
0
0
0
0
0
0
0
2
0
0
0
0
0
0
0
0
0
0
0
0
0
0
0
0
0
0
0
0
0
0
0
0
0
0
0
0
0


0
0
0
0
0
0
0
0
0
0
0
1
0
0
2
0
2
0
1
1
0
1
0
0
0
0
0
0
0
0
0
0
0
0
0
1
0
0
0
0
0
0
0
0
0
0
0
0
0
0
0
1
0
1
0
2
0
0
0
0
0
0
0
1
0
0
0
0
0
0
0
0
0
0
0
1
0
0
0
0
0


0
0
0
0
0
0
0
0
0
0
0
1
0
0
0
0
0
0
0
0
0
0
0
0
0
0
0
0
0
0
0
0
0
0
0
0
0
0
0
0
0
0
0
0
0
0
0
0
0
0
0
0
0
0
0
0
0
0
0
0
0
0
0
0
0
0
0
0
0
0
0
0
0
0
0
0
0
0
0
0
0


0
0
0
0
0
0
0
0
0
0
0
0
0
0
1
0
0
0
0
0
0
0
0
0
0
0
0
0
0
0
0
0
0
0
0
0
0
0
0
0
0
0
0
0
0
0
0
0
0
0
0
0
0
0
0
0
0
0
0
0
0
0
0
0
0
0
0
0
0
0
0
0
0
0
0
0
0
0
0
0
0


0
0
0
0
0
0
0
0
0
0
0
0
0
0
1
0
2
0
1
1
0
1
0
0
0
0
0
0
0
0
0
0
0
0
0
1
0
0
0
0
0
0
0
0
0
0
0
0
0
0
0
1
0
1
0
2
0
0
0
0
0
0
0
1
0
0
0
0
0
0
0
0
0
0
0
1
0
0
0
0
0


0
0
0
2
1
0
0
0
4
0
0
2
0
2
0
0
0
0
0
0
0
0
0
0
0
0
0
0
0
2
0
87
0
9
0
4
18
0
0
1
0
0
0
0
0
0
0
0
0
0
0
0
0
0
0
0
0
0
0
0
0
0
11
0
0
0
0
0
0
0
0
0
0
0
0
0
0
0
0
0
0


0
0
0
2
1
0
0
0
4
0
0
2
0
2
0
0
0
0
0
0
0
0
0
0
0
0
0
0
0
2
0
87
0
9
0
4
18
0
0
1
0
0
0
0
0
0
0
0
0
0
0
0
0
0
0
0
0
0
0
0
0
0
11
0
0
0
0
0
0
0
0
0
0
0
0
0
0
0
0
0
0


0
0
5
0
2
0
2
1
4
1
0
0
2
0
1
0
2
2
9
3
0
2
4
0
0
0
0
0
0
0
0
0
0
3
0
0
0
0
0
1
2
0
0
0
0
0
1
0
0
0
0
2
1
0
0
2
10
0
1
0
2
0
71
0
0
0
0
0
0
0
4
0
6
0
0
0
0
0
0
0
0


0
0
5
0
1
0
2
1
4
1
0
0
2
0
1
0
1
2
1
3
0
1
4
0
0
0
0
0
0
0
0
0
0
3
0
0
0
0
0
1
1
0
0
0
0
0
1
0
0
0
0
2
1
0
0
2
10
0
0
0
2
0
0
0
0
0
0
0
0
0
4
0
6
0
0
0
0
0
0
0
0


0
0
0
0
1
0
0
0
0
0
0
0
0
0
0
0
1
0
8
0
0
1
0
0
0
0
0
0
0
0
0
0
0
0
0
0
0
0
0
0
1
0
0
0
0
0
0
0
0
0
0
0
0
0
0
0
0
0
1
0
0
0
71
0
0
0
0
0
0
0
0
0
0
0
0
0
0
0
0
0
0


0
0
0
0
0
0
0
0
0
0
0
0
0
0
0
0
0
0
0
0
0
0
0
0
0
0
0
0
0
0
0
0
0
0
0
0
0
2
0
1
0
0
0
0
0
0
0
0
0
0
0
0
0
0
0
0
0
0
0
0
0
0
0
0
0
0
0
0
0
0
0
0
0
0
0
0
0
0
0
0
0


0
0
0
0
0
0
0
0
0
0
0
0
0
0
0
0
0
0
0
0
0
0
0
0
0
0
0
0
0
0
0
0
0
0
0
0
0
2
0
1
0
0
0
0
0
0
0
0
0
0
0
0
0
0
0
0
0
0
0
0
0
0
0
0
0
0
0
0
0
0
0
0
0
0
0
0
0
0
0
0
0


0
6
7
2
9
4
36
1
4
3
13
12
13
9
7
12
23
5
21
3
3
17
5
0
0
4
0
0
0
1
2
15
3
25
0
10
0
0
2
6
3
17
2
2
3
0
6
6
0
0
6
5
5
6
14
6
5
3
22
7
3
8
25
4
3
2
2
5
1
0
10
8
7
1
8
1
5
4
2
1
1


0
6
7
2
9
4
36
1
4
3
13
12
13
9
7
12
23
5
21
3
3
17
5
0
0
4
0
0
0
1
2
15
3
25
0
10
0
0
2
6
3
17
2
2
3
0
6
6
0
0
6
5
5
6
14
6
5
3
22
7
3
8
25
4
3
2
2
5
1
0
10
8
7
1
8
1
5
4
2
1
1


67
561
372
174
218
192
201
86
102
76
171
60
78
86
121
322
163
157
370
603
234
94
96
237
211
132
191
470
439
95
344
223
157
65
39
118
84
74
109
281
228
148
87
148
196
77
562
221
59
0
70
89
138
103
72
170
222
222
225
339
148
81
76
40
35
92
50
63
87
132
53
22
32
14
27
17
18
68
14
40
15


19
28
8
133
31
109
26
23
29
32
37
18
14
17
3
57
20
15
21
82
15
14
11
0
0
7
0
2
0
12
21
84
0
32
7
61
0
0
4
3
9
13
8
10
5
11
5
1
1
0
8
28
49
8
17
20
18
23
62
30
24
12
29
2
11
5
23
4
30
73
8
9
23
1
7
4
6
16
11
20
8


19
28
8
133
31
109
26
23
29
32
37
18
14
17
3
57
20
15
21
82
15
14
11
0
0
7
0
2
0
12
21
84
0
32
7
61
0
0
4
3
9
13
8
10
5
11
5
1
1
0
8
28
49
8
17
20
18
23
62
30
24
12
29
2
11
5
23
4
30
73
8
9
23
1
7
4
6
16
11
20
8


36
463
296
22
156
52
150
51
64
35
106
33
48
49
98
208
110
117
292
458
183
47
60
0
1
93
0
414
0
69
296
109
123
17
11
38
0
65
90
252
197
111
62
105
172
55
457
197
51
0
54
51
59
79
41
127
165
162
125
270
92
60
33
33
23
66
13
54
34
41
31
0
7
7
13
7
0
15
0
12
2


0
0
0
0
0
0
0
0
0
0
0
0
2
0
0
0
0
0
0
0
0
0
0
0
0
0
0
0
0
0
93
0
0
0
0
0
0
0
0
0
0
0
0
0
0
0
0
0
0
0
0
3
0
0
2
0
0
0
0
0
0
0
0
0
0
0
0
0
0
0
0
0
0
0
0
0
0
0
0
0
0


0
0
0
0
0
0
0
0
0
0
1
0
1
0
0
0
0
0
0
0
0
0
0
0
0
0
0
0
0
0
0
0
0
0
0
0
0
0
1
0
0
0
0
0
0
0
0
0
0
0
0
0
0
0
0
0
0
0
0
0
0
2
0
0
0
0
0
0
0
0
0
0
0
0
0
0
0
0
0
0
0


35
463
295
22
156
52
150
51
63
33
105
33
45
49
96
208
110
117
292
458
183
47
60
0
1
93
0
414
0
69
200
109
123
17
11
38
0
65
89
252
197
111
62
105
172
55
457
197
51
0
54
48
59
79
39
127
163
162
125
270
92
58
33
33
23
66
13
54
34
41
31
0
7
7
13
7
0
15
0
12
2


1
0
1
0
0
0
0
0
1
2
0
0
0
0
2
0
0
0
0
0
0
0
0
0
0
0
0
0
0
0
3
0
0
0
0
0
0
0
0
0
0
0
0
0
0
0
0
0
0
0
0
0
0
0
0
0
2
0
0
0
0
0
0
0
0
0
0
0
0
0
0
0
0
0
0
0
0
0
0
0
0


0
0
0
0
0
0
0
0
0
0
1
0
0
0
0
0
0
0
0
0
6
0
0
0
0
0
0
0
0
0
0
0
0
0
0
0
0
0
0
0
0
0
0
0
0
0
0
0
0
0
0
0
0
0
0
0
0
2
0
0
0
0
0
0
0
0
0
0
0
0
0
0
0
0
0
0
0
0
0
0
0


0
0
0
0
0
0
0
0
0
0
1
0
0
0
0
0
0
0
0
0
6
0
0
0
0
0
0
0
0
0
0
0
0
0
0
0
0
0
0
0
0
0
0
0
0
0
0
0
0
0
0
0
0
0
0
0
0
2
0
0
0
0
0
0
0
0
0
0
0
0
0
0
0
0
0
0
0
0
0
0
0


0
0
0
0
0
0
0
0
0
0
0
0
0
0
0
0
0
0
0
0
0
0
0
0
0
0
53
1
0
0
0
0
0
0
0
0
0
0
0
0
0
0
0
0
0
0
0
0
0
0
0
0
0
0
0
0
0
0
0
0
0
0
0
0
0
0
0
0
0
1
0
0
0
0
0
0
0
0
0
0
0


0
0
0
0
0
0
0
0
0
0
0
0
0
0
0
0
0
0
0
0
0
0
0
0
0
0
53
1
0
0
0
0
0
0
0
0
0
0
0
0
0
0
0
0
0
0
0
0
0
0
0
0
0
0
0
0
0
0
0
0
0
0
0
0
0
0
0
0
0
1
0
0
0
0
0
0
0
0
0
0
0


0
0
0
0
0
0
0
0
0
0
0
0
0
0
0
0
0
0
0
0
0
0
0
0
0
0
0
0
0
0
0
0
0
0
0
1
0
0
0
0
0
0
0
0
0
0
0
0
0
0
0
0
0
0
0
0
0
0
0
0
0
0
0
0
0
0
0
0
0
0
0
0
0
0
0
0
0
0
0
0
0


0
0
0
0
0
0
0
0
0
0
0
0
0
0
0
0
0
0
0
0
0
0
0
0
0
0
0
0
0
0
0
0
0
0
0
1
0
0
0
0
0
0
0
0
0
0
0
0
0
0
0
0
0
0
0
0
0
0
0
0
0
0
0
0
0
0
0
0
0
0
0
0
0
0
0
0
0
0
0
0
0


0
0
0
0
0
1
0
0
0
0
0
0
0
0
0
0
0
0
0
0
0
0
2
99
76
4
10
11
264
0
0
0
0
0
0
0
0
0
0
0
0
0
0
3
0
0
0
12
0
0
0
1
0
0
0
0
0
0
0
0
1
0
0
0
0
0
0
0
0
0
0
0
0
0
0
0
0
0
0
0
0


0
0
0
0
0
0
0
0
0
0
0
0
0
0
0
0
0
0
0
0
0
0
2
99
76
4
10
10
257
0
0
0
0
0
0
0
0
0
0
0
0
0
0
3
0
0
0
12
0
0
0
1
0
0
0
0
0
0
0
0
1
0
0
0
0
0
0
0
0
0
0
0
0
0
0
0
0
0
0
0
0


0
0
0
0
0
1
0
0
0
0
0
0
0
0
0
0
0
0
0
0
0
0
0
0
0
0
0
1
7
0
0
0
0
0
0
0
0
0
0
0
0
0
0
0
0
0
0
0
0
0
0
0
0
0
0
0
0
0
0
0
0
0
0
0
0
0
0
0
0
0
0
0
0
0
0
0
0
0
0
0
0


0
0
0
1
0
0
0
0
0
0
0
0
1
0
0
1
0
0
0
0
0
1
0
0
0
0
0
0
0
0
0
0
0
0
0
0
0
0
0
0
0
0
0
0
0
0
0
0
0
0
0
0
0
0
0
0
0
0
0
0
0
0
0
0
0
0
0
0
0
0
0
0
0
0
0
0
0
0
0
0
0


0
0
0
1
0
0
0
0
0
0
0
0
1
0
0
1
0
0
0
0
0
1
0
0
0
0
0
0
0
0
0
0
0
0
0
0
0
0
0
0
0
0
0
0
0
0
0
0
0
0
0
0
0
0
0
0
0
0
0
0
0
0
0
0
0
0
0
0
0
0
0
0
0
0
0
0
0
0
0
0
0


0
3
2
0
1
1
0
0
0
0
2
0
0
0
0
2
0
0
0
0
0
2
0
0
0
0
0
1
0
0
0
0
0
0
0
0
0
0
1
2
4
0
0
0
0
0
2
0
0
0
0
1
0
0
0
0
1
4
1
0
0
0
1
0
0
0
0
1
0
0
0
0
0
0
0
0
0
0
0
0
0


0
3
2
0
1
1
0
0
0
0
2
0
0
0
0
2
0
0
0
0
0
2
0
0
0
0
0
1
0
0
0
0
0
0
0
0
0
0
1
2
4
0
0
0
0
0
2
0
0
0
0
1
0
0
0
0
1
4
1
0
0
0
1
0
0
0
0
1
0
0
0
0
0
0
0
0
0
0
0
0
0


0
1
1
0
0
0
0
1
0
2
0
1
0
0
0
1
0
0
0
0
1
1
1
73
105
0
127
6
175
2
0
1
30
0
5
0
0
0
0
0
0
0
1
4
1
0
0
0
0
0
0
0
0
1
0
0
1
0
0
1
1
1
4
0
0
1
1
0
2
0
0
2
0
0
0
0
0
0
1
0
1


0
0
0
0
0
0
0
0
0
0
0
0
0
0
0
0
0
0
0
0
0
0
0
0
0
0
1
0
0
0
0
0
0
0
0
0
0
0
0
0
0
0
0
0
0
0
0
0
0
0
0
0
0
0
0
0
0
0
0
0
0
0
0
0
0
0
0
0
0
0
0
0
0
0
0
0
0
0
0
0
0


0
0
0
0
0
0
0
1
0
1
0
0
0
0
0
0
0
0
0
0
0
0
0
0
0
0
0
0
0
0
0
0
0
0
0
0
0
0
0
0
0
0
0
0
0
0
0
0
0
0
0
0
0
0
0
0
0
0
0
0
0
0
0
0
0
0
0
0
0
0
0
0
0
0
0
0
0
0
0
0
0


0
1
1
0
0
0
0
0
0
1
0
1
0
0
0
1
0
0
0
0
1
1
1
73
104
0
0
4
174
2
0
1
30
0
5
0
0
0
0
0
0
0
1
0
0
0
0
0
0
0
0
0
0
0
0
0
0
0
0
1
1
0
3
0
0
1
1
0
1
0
0
0
0
0
0
0
0
0
0
0
0


0
0
0
0
0
0
0
0
0
0
0
0
0
0
0
0
0
0
0
0
0
0
0
0
0
0
123
1
0
0
0
0
0
0
0
0
0
0
0
0
0
0
0
4
1
0
0
0
0
0
0
0
0
1
0
0
1
0
0
0
0
1
1
0
0
0
0
0
1
0
0
1
0
0
0
0
0
0
0
0
1


0
0
0
0
0
0
0
0
0
0
0
0
0
0
0
0
0
0
0
0
0
0
0
0
1
0
3
1
1
0
0
0
0
0
0
0
0
0
0
0
0
0
0
0
0
0
0
0
0
0
0
0
0
0
0
0
0
0
0
0
0
0
0
0
0
0
0
0
0
0
0
1
0
0
0
0
0
0
1
0
0


1
0
0
0
2
10
0
0
0
0
0
0
0
0
0
1
3
0
0
8
0
2
0
0
0
3
0
0
0
2
0
24
0
0
0
1
82
0
0
1
1
0
0
3
0
4
0
0
0
0
0
3
22
0
0
0
0
4
1
0
1
0
1
0
0
2
6
0
13
12
0
8
2
2
6
4
8
27
0
5
3


1
0
0
0
2
10
0
0
0
0
0
0
0
0
0
1
3
0
0
8
0
2
0
0
0
3
0
0
0
2
0
24
0
0
0
1
82
0
0
1
1
0
0
3
0
4
0
0
0
0
0
3
22
0
0
0
0
4
1
0
1
0
1
0
0
2
6
0
13
12
0
8
2
2
6
4
8
27
0
5
3


11
66
65
18
28
19
25
11
9
7
25
8
15
20
20
52
30
25
57
55
29
27
22
65
29
25
1
35
0
10
27
5
4
16
16
17
2
9
14
23
17
24
16
23
18
7
98
11
7
0
8
5
8
15
14
23
37
27
36
38
29
8
8
5
1
18
7
4
8
5
14
3
0
4
1
2
4
10
2
3
1


11
66
65
18
28
19
25
11
9
7
25
8
15
20
20
52
30
25
57
55
29
27
22
65
29
25
1
35
0
10
27
5
4
16
16
17
2
9
14
23
17
24
16
23
18
7
98
11
7
0
8
5
8
15
14
23
37
27
36
38
29
8
8
5
1
18
7
4
8
5
14
3
0
4
1
2
4
10
2
3
1


185
313
506
318
413
434
280
265
563
181
605
461
536
231
528
499
861
1493
620
455
493
808
953
28
63
1330
138
811
0
495
367
934
539
1006
330
707
134
165
338
1435
1027
602
431
632
861
421
928
899
1496
0
320
374
315
409
533
360
497
470
570
536
692
372
555
257
666
657
317
498
364
162
638
298
217
299
82
366
131
118
229
247
130


5
4
37
3
3
5
2
0
15
7
41
6
37
5
21
26
31
99
39
24
25
96
18
0
0
199
0
68
0
5
7
13
39
55
2
12
0
0
15
114
95
56
10
22
9
8
53
24
46
0
12
12
11
32
21
13
23
35
25
43
17
11
3
8
8
7
1
18
2
0
11
4
23
26
2
1
4
4
15
5
2


5
4
37
3
3
5
2
0
15
7
41
6
37
5
21
26
31
99
39
24
25
96
18
0
0
199
0
68
0
5
7
13
39
55
2
12
0
0
15
114
95
56
10
22
9
8
53
24
46
0
12
12
11
32
21
13
23
35
25
43
17
11
3
8
8
7
1
18
2
0
11
4
23
26
2
1
4
4
15
5
2


0
0
0
2
0
4
0
0
1
0
0
1
0
0
0
0
0
0
0
0
0
0
0
0
0
0
0
0
0
0
0
0
0
0
0
2
0
0
0
1
0
0
0
0
0
0
0
0
0
0
0
0
0
0
0
2
0
1
3
0
2
0
2
0
0
2
0
0
0
0
0
0
0
0
1
0
0
0
0
0
0


0
0
0
2
0
4
0
0
1
0
0
1
0
0
0
0
0
0
0
0
0
0
0
0
0
0
0
0
0
0
0
0
0
0
0
2
0
0
0
1
0
0
0
0
0
0
0
0
0
0
0
0
0
0
0
2
0
1
3
0
2
0
2
0
0
2
0
0
0
0
0
0
0
0
1
0
0
0
0
0
0


1
4
9
2
5
2
5
0
4
4
3
0
2
4
18
12
9
28
15
5
9
42
28
0
0
171
0
9
0
2
4
0
0
0
0
1
0
4
6
44
23
17
4
10
3
3
25
11
5
0
7
14
5
2
4
2
13
10
11
11
0
5
0
0
5
2
0
4
1
9
1
0
0
0
3
0
0
0
1
0
0


1
4
9
2
5
2
5
0
4
4
3
0
2
4
18
12
9
28
15
5
9
42
28
0
0
171
0
9
0
2
4
0
0
0
0
1
0
4
6
44
23
17
4
10
3
3
25
11
5
0
7
14
5
2
4
2
13
10
11
11
0
5
0
0
5
2
0
4
1
9
1
0
0
0
3
0
0
0
1
0
0


13
71
106
9
97
81
67
39
124
14
247
72
312
65
190
169
310
598
219
58
89
181
501
1
62
175
94
56
0
18
16
20
0
53
30
13
0
12
35
112
161
88
76
60
19
21
79
68
669
0
76
77
21
84
69
71
93
42
102
144
134
119
3
102
20
30
6
98
22
9
55
4
0
0
0
0
4
3
7
9
4


13
71
106
9
97
81
67
39
124
14
247
72
312
65
190
169
310
598
219
58
89
181
501
1
62
175
94
56
0
18
16
20
0
53
30
13
0
12
35
112
161
88
76
60
19
21
79
68
669
0
76
77
21
84
69
71
93
42
102
144
134
119
3
102
20
30
6
98
22
9
55
4
0
0
0
0
4
3
7
9
4


0
5
5
0
4
2
3
0
2
3
19
6
6
4
11
8
18
20
23
9
39
39
37
0
0
84
0
22
0
3
5
0
0
0
1
2
0
3
8
36
20
14
4
8
13
6
23
8
0
0
2
2
2
4
0
0
13
2
5
5
5
2
0
0
0
2
0
0
0
0
1
0
0
0
0
0
0
0
0
0
0


0
5
5
0
4
2
3
0
2
3
18
6
6
4
7
8
18
19
20
9
35
37
36
0
0
81
0
20
0
3
5
0
0
0
0
2
0
3
5
35
18
14
4
8
12
6
23
8
0
0
1
2
0
4
0
0
12
2
2
5
5
2
0
0
0
2
0
0
0
0
1
0
0
0
0
0
0
0
0
0
0


0
0
0
0
0
0
0
0
0
0
0
0
0
0
1
0
0
0
0
0
0
1
0
0
0
0
0
0
0
0
0
0
0
0
0
0
0
0
0
0
0
0
0
0
1
0
0
0
0
0
0
0
0
0
0
0
0
0
2
0
0
0
0
0
0
0
0
0
0
0
0
0
0
0
0
0
0
0
0
0
0


0
0
0
0
0
0
0
0
0
0
1
0
0
0
3
0
0
1
3
0
4
1
1
0
0
3
0
2
0
0
0
0
0
0
1
0
0
0
3
1
2
0
0
0
0
0
0
0
0
0
1
0
2
0
0
0
1
0
1
0
0
0
0
0
0
0
0
0
0
0
0
0
0
0
0
0
0
0
0
0
0


1
5
4
3
4
10
2
2
6
0
6
3
0
0
10
7
3
30
1
7
2
14
8
0
0
50
0
32
0
2
7
0
65
3
2
16
86
0
3
44
30
11
8
11
14
4
13
4
23
0
2
8
14
9
7
3
6
6
1
4
1
2
1
0
2
7
6
3
2
0
2
5
3
8
5
7
3
4
6
5
0


1
5
4
3
4
10
2
2
6
0
6
3
0
0
10
7
3
30
1
7
2
14
8
0
0
50
0
32
0
2
7
0
65
3
2
16
86
0
3
44
30
11
8
11
14
4
13
4
23
0
2
8
14
9
7
3
6
6
1
4
1
2
1
0
2
7
6
3
2
0
2
5
3
8
5
7
3
4
6
5
0


44
17
29
102
90
98
46
45
180
69
99
98
59
46
47
36
66
144
40
33
35
64
64
0
0
36
0
22
0
73
40
71
30
241
91
48
0
27
36
79
89
84
68
90
151
67
36
37
211
0
100
80
104
75
206
137
100
137
169
46
276
98
124
59
323
226
150
225
90
38
278
59
41
98
6
62
11
22
64
61
28


44
17
29
102
90
97
46
45
179
68
95
98
59
46
47
33
65
144
40
32
35
63
64
0
0
36
0
22
0
73
38
71
30
241
91
46
0
27
36
78
89
84
68
89
149
67
36
37
211
0
98
80
100
75
204
136
98
132
169
45
270
97
124
58
321
220
149
219
87
38
276
59
41
96
6
62
11
22
64
61
27


0
0
0
0
0
0
0
0
0
0
3
0
0
0
0
1
0
0
0
0
0
0
0
0
0
0
0
0
0
0
0
0
0
0
0
0
0
0
0
0
0
0
0
0
0
0
0
0
0
0
0
0
0
0
0
0
0
0
0
0
0
0
0
0
0
0
0
0
0
0
0
0
0
0
0
0
0
0
0
0
0


0
0
0
0
0
1
0
0
1
1
1
0
0
0
0
2
1
0
0
1
0
1
0
0
0
0
0
0
0
0
2
0
0
0
0
2
0
0
0
1
0
0
0
1
2
0
0
0
0
0
2
0
4
0
2
1
2
5
0
1
6
1
0
1
2
6
1
6
3
0
2
0
0
2
0
0
0
0
0
0
1


2
0
0
9
3
3
1
1
0
0
0
2
1
0
0
2
3
1
0
2
3
12
7
0
0
1
0
11
0
0
3
12
25
0
1
15
0
3
1
5
4
5
3
8
12
3
14
8
0
0
0
6
0
3
0
1
3
9
9
0
0
0
0
0
0
2
3
2
6
0
7
2
0
2
0
0
3
0
0
3
2


2
0
0
9
3
3
1
1
0
0
0
2
1
0
0
2
3
1
0
2
3
12
7
0
0
1
0
11
0
0
3
12
25
0
1
15
0
3
1
5
4
5
3
8
12
3
14
8
0
0
0
6
0
3
0
1
3
9
9
0
0
0
0
0
0
2
3
2
6
0
7
2
0
2
0
0
3
0
0
3
2


0
0
0
0
0
0
0
0
2
0
1
0
0
0
0
3
1
4
0
0
2
0
0
0
0
0
0
3
0
0
0
0
0
0
0
2
0
0
0
1
2
1
0
0
0
0
2
0
2
0
0
0
0
0
0
0
0
1
0
0
0
0
0
0
0
0
0
2
0
0
0
0
0
0
2
0
0
0
0
0
0


0
0
0
0
0
0
0
0
0
0
0
0
0
0
0
0
0
0
0
0
0
0
0
0
0
0
0
0
0
0
0
0
0
0
0
0
0
0
0
0
0
0
0
0
0
0
0
0
0
0
0
0
0
0
0
0
0
0
0
0
0
0
0
0
0
0
0
1
0
0
0
0
0
0
0
0
0
0
0
0
0


0
0
0
0
0
0
0
0
2
0
1
0
0
0
0
3
1
4
0
0
2
0
0
0
0
0
0
3
0
0
0
0
0
0
0
2
0
0
0
1
2
1
0
0
0
0
2
0
2
0
0
0
0
0
0
0
0
1
0
0
0
0
0
0
0
0
0
1
0
0
0
0
0
0
2
0
0
0
0
0
0


5
19
11
4
12
8
19
8
4
7
23
18
19
18
31
28
75
51
30
15
66
80
60
0
0
137
44
92
0
22
22
10
48
0
20
3
0
18
16
115
76
32
43
26
25
15
78
58
36
0
9
15
5
25
13
13
52
14
17
56
28
11
1
14
4
8
0
5
4
8
2
6
0
1
1
2
3
1
3
3
7


0
4
0
0
1
4
7
1
2
0
2
2
1
1
1
1
1
0
0
3
0
3
0
0
0
0
0
0
0
0
0
0
0
0
1
0
0
0
0
0
1
0
1
0
4
3
0
0
1
0
1
2
0
1
0
0
0
4
3
0
4
1
1
0
1
3
0
0
2
0
0
1
0
0
1
0
0
0
0
1
4


5
13
10
3
8
4
9
6
2
6
21
15
18
15
30
26
74
50
30
11
65
75
59
0
0
133
44
92
0
22
22
10
48
0
19
3
0
18
13
115
72
29
41
24
20
10
76
58
34
0
8
12
5
24
12
10
48
10
12
56
21
6
0
13
1
3
0
5
1
8
1
0
0
0
0
0
0
0
2
1
0


0
2
1
1
3
0
3
1
0
1
0
1
0
2
0
1
0
1
0
1
1
2
1
0
0
4
0
0
0
0
0
0
0
0
0
0
0
0
3
0
3
3
1
2
1
2
2
0
1
0
0
1
0
0
1
3
4
0
2
0
3
4
0
1
2
2
0
0
1
0
1
5
0
1
0
2
3
1
1
1
3


114
188
305
184
195
221
135
170
225
77
166
255
100
89
200
208
345
518
253
302
223
280
230
27
1
477
0
496
0
370
263
808
332
654
183
593
48
98
218
884
527
294
215
397
615
294
605
681
504
0
112
160
153
175
213
118
194
213
228
227
229
124
421
74
304
371
151
141
237
98
281
218
150
164
62
294
103
84
133
161
87


114
188
305
184
195
221
135
170
225
77
166
255
100
89
200
208
345
518
253
302
223
280
230
27
1
477
0
496
0
370
263
808
332
654
183
593
48
98
218
884
527
294
215
397
615
294
605
681
504
0
112
160
153
175
213
118
194
213
228
227
229
124
421
74
304
371
151
141
237
98
281
218
150
164
62
294
103
84
133
161
87


17
62
55
75
77
76
30
46
100
21
74
76
39
33
101
79
140
297
71
89
88
104
105
0
0
230
0
175
0
127
174
243
850
286
45
293
112
47
88
202
164
107
75
132
119
87
210
126
96
0
35
52
54
63
66
72
58
56
103
80
84
54
190
27
27
55
20
91
35
47
61
66
117
95
54
66
63
72
45
29
36


17
62
55
75
77
76
30
46
100
21
74
76
39
33
101
79
140
297
71
89
88
104
105
0
0
230
0
175
0
127
174
243
850
286
45
293
112
47
88
202
164
107
75
132
119
87
210
126
96
0
35
52
54
63
66
72
58
56
103
80
84
54
190
27
27
55
20
91
35
47
61
66
117
95
54
66
63
72
45
29
36


15
37
37
57
53
41
18
40
66
11
45
44
15
15
48
42
84
165
49
57
29
75
79
0
0
102
0
82
0
83
131
165
417
229
41
84
109
29
59
140
103
74
57
102
93
59
118
104
60
0
25
23
20
40
52
47
14
22
57
49
51
33
34
13
9
28
2
55
27
24
40
16
14
21
5
12
13
22
11
13
8


0
0
0
0
0
0
0
0
0
0
0
0
0
0
0
1
0
1
0
0
0
0
0
0
0
0
0
0
0
0
0
0
0
0
0
0
0
0
0
0
0
0
0
0
0
0
0
0
0
0
0
0
0
0
0
0
0
0
0
0
0
0
1
0
0
0
0
0
0
0
0
0
0
0
0
0
0
0
0
0
0


0
0
0
2
0
1
0
0
1
1
0
3
0
0
3
2
0
0
0
1
0
0
0
0
0
0
0
0
0
0
0
3
0
3
0
92
0
0
0
2
1
0
0
3
0
0
0
0
0
0
0
2
1
0
0
1
0
2
0
0
1
0
55
0
3
0
1
0
0
4
5
16
23
21
11
18
9
5
7
2
4


0
0
0
0
0
0
0
0
0
0
0
0
0
0
0
2
0
1
0
0
0
0
0
0
0
0
0
0
0
0
0
0
0
0
0
0
0
0
0
0
0
0
0
0
0
0
0
0
0
0
0
0
0
0
0
0
0
1
0
0
0
0
0
0
0
0
0
1
0
0
0
1
0
0
1
0
0
0
1
0
0


0
0
0
0
0
0
0
0
1
0
0
1
0
0
0
0
0
0
1
0
0
0
0
0
0
0
0
1
0
0
0
0
0
0
0
0
0
0
0
1
0
2
0
0
0
2
0
0
0
0
0
0
0
0
0
0
1
0
0
0
1
0
1
0
0
1
0
0
0
0
0
0
0
0
0
0
0
0
0
0
0


0
1
0
0
1
1
1
0
1
2
2
2
1
1
4
3
0
5
2
1
2
2
3
0
0
0
0
0
0
0
0
0
0
0
0
1
0
0
0
0
0
1
0
0
0
2
0
0
2
0
1
1
0
4
2
0
1
2
0
1
0
2
0
0
0
2
0
3
0
2
0
0
0
0
0
0
0
0
0
0
0


2
24
18
16
23
33
11
6
31
7
27
26
23
17
46
29
56
125
19
30
57
27
23
0
0
128
0
92
0
44
43
75
433
54
4
116
3
18
29
59
60
30
18
27
26
24
92
22
34
0
9
26
33
19
12
24
42
29
46
30
31
19
99
14
15
24
17
32
8
17
16
33
80
53
37
36
41
45
26
14
24


0
6
5
13
3
3
6
1
1
1
6
9
4
6
12
10
19
19
11
7
0
2
0
0
0
4
0
0
0
1
3
0
0
1
6
0
0
3
0
4
2
2
1
0
3
3
2
0
0
0
10
9
5
14
20
16
22
8
30
30
6
17
22
3
1
8
0
6
1
0
3
0
1
1
0
0
0
5
0
1
0


0
6
5
13
3
3
6
1
1
1
6
9
4
6
12
10
19
19
11
7
0
2
0
0
0
4
0
0
0
1
3
0
0
1
6
0
0
3
0
4
2
2
1
0
3
3
2
0
0
0
10
9
5
14
20
16
22
8
30
30
6
17
22
3
1
8
0
6
1
0
3
0
1
1
0
0
0
5
0
1
0


0
6
5
13
3
3
6
1
1
1
6
9
4
6
12
10
19
19
11
7
0
2
0
0
0
4
0
0
0
1
3
0
0
1
6
0
0
3
0
4
2
2
1
0
3
3
2
0
0
0
10
9
5
14
20
16
22
8
30
30
6
17
22
3
1
8
0
6
1
0
3
0
1
1
0
0
0
5
0
1
0


2
6
8
13
16
18
16
3
6
5
12
10
7
11
17
15
15
17
26
12
2
8
6
0
0
6
0
6
0
5
4
28
0
41
3
4
0
1
4
5
6
8
7
8
3
3
9
4
9
0
6
16
7
11
24
17
12
11
26
20
21
9
14
8
10
8
11
19
7
3
5
5
1
3
4
1
1
7
2
8
2


0
0
1
1
0
3
2
0
0
0
0
0
0
3
1
0
0
0
0
0
1
1
2
0
0
0
0
0
0
4
2
0
0
7
0
0
0
0
2
0
1
4
0
5
2
2
0
2
1
0
1
1
2
1
2
0
3
1
5
2
4
1
5
0
4
1
2
3
1
2
2
3
1
1
2
0
0
4
0
0
2


0
0
1
1
0
3
2
0
0
0
0
0
0
3
1
0
0
0
0
0
1
1
2
0
0
0
0
0
0
4
2
0
0
7
0
0
0
0
2
0
1
4
0
5
2
2
0
2
1
0
1
1
2
1
2
0
3
1
5
2
4
1
5
0
4
1
2
3
1
2
2
3
1
1
2
0
0
4
0
0
2


1
1
4
3
3
13
1
3
3
0
2
8
0
2
4
4
3
7
1
2
0
4
0
0
0
1
0
0
0
0
2
28
0
34
3
2
0
1
1
5
1
0
3
2
1
1
3
2
4
0
4
6
0
0
5
6
1
6
5
7
7
3
5
4
5
3
8
7
3
0
0
1
0
2
2
0
1
0
0
1
0


1
1
1
3
2
10
1
2
2
0
2
6
0
2
2
4
3
5
1
1
0
4
0
0
0
0
0
0
0
0
1
27
0
28
3
1
0
1
1
5
1
0
2
2
1
1
3
2
3
0
2
5
0
0
5
5
1
6
3
7
7
2
4
3
3
3
6
5
3
0
0
1
0
2
2
0
0
0
0
1
0


0
0
3
0
1
1
0
1
0
0
0
0
0
0
0
0
0
2
0
0
0
0
0
0
0
0
0
0
0
0
1
0
0
6
0
1
0
0
0
0
0
0
1
0
0
0
0
0
0
0
0
0
0
0
0
0
0
0
0
0
0
0
1
0
0
0
2
0
0
0
0
0
0
0
0
0
0
0
0
0
0


0
0
0
0
0
2
0
0
1
0
0
2
0
0
2
0
0
0
0
1
0
0
0
0
0
1
0
0
0
0
0
1
0
0
0
0
0
0
0
0
0
0
0
0
0
0
0
0
1
0
2
1
0
0
0
1
0
0
2
0
0
1
0
1
2
0
0
2
0
0
0
0
0
0
0
0
1
0
0
0
0


1
5
3
9
12
2
13
0
3
4
9
2
7
4
9
9
12
10
24
8
1
3
3
0
0
5
0
2
0
0
0
0
0
0
0
2
0
0
0
0
3
4
4
1
0
0
0
0
4
0
1
9
5
9
17
10
5
4
15
11
9
5
4
3
1
4
1
9
3
0
3
0
0
0
0
1
0
2
2
7
0


1
3
0
7
12
2
9
0
3
4
8
2
7
1
8
3
6
10
23
8
1
1
2
0
0
4
0
1
0
0
0
0
0
0
0
2
0
0
0
0
2
0
4
0
0
0
0
0
1
0
1
7
5
6
16
7
4
4
10
6
8
5
3
3
1
3
1
6
2
0
3
0
0
0
0
1
0
2
2
7
0


0
2
3
1
0
0
4
0
0
0
0
0
0
0
0
6
5
0
1
0
0
2
1
0
0
0
0
1
0
0
0
0
0
0
0
0
0
0
0
0
0
4
0
1
0
0
0
0
2
0
0
1
0
3
1
3
0
0
5
4
0
0
0
0
0
1
0
3
0
0
0
0
0
0
0
0
0
0
0
0
0


0
0
0
0
0
0
0
0
0
0
0
0
0
0
0
0
0
0
0
0
0
0
0
0
0
1
0
0
0
0
0
0
0
0
0
0
0
0
0
0
0
0
0
0
0
0
0
0
0
0
0
0
0
0
0
0
0
0
0
0
0
0
0
0
0
0
0
0
0
0
0
0
0
0
0
0
0
0
0
0
0


0
0
0
1
0
0
0
0
0
0
1
0
0
3
1
0
1
0
0
0
0
0
0
0
0
0
0
0
0
0
0
0
0
0
0
0
0
0
0
0
1
0
0
0
0
0
0
0
1
0
0
1
0
0
0
0
1
0
0
1
1
0
1
0
0
0
0
0
1
0
0
0
0
0
0
0
0
0
0
0
0


0
0
0
0
1
0
0
0
0
1
1
0
0
2
3
2
0
0
1
2
0
0
1
0
0
0
0
4
0
1
0
0
0
0
0
0
0
0
1
0
1
0
0
0
0
0
6
0
0
0
0
0
0
1
0
1
3
0
1
0
1
0
0
1
0
0
0
0
0
1
0
1
0
0
0
0
0
1
0
0
0


0
0
0
0
1
0
0
0
0
1
1
0
0
2
3
2
0
0
1
2
0
0
1
0
0
0
0
4
0
1
0
0
0
0
0
0
0
0
1
0
1
0
0
0
0
0
6
0
0
0
0
0
0
1
0
1
3
0
1
0
1
0
0
1
0
0
0
0
0
1
0
1
0
0
0
0
0
1
0
0
0


52
196
257
84
174
121
170
38
111
81
171
134
205
94
280
215
323
413
212
241
163
289
167
72
13
397
25
223
71
294
437
313
315
429
120
220
208
90
174
450
447
246
221
328
294
216
226
259
242
12
133
209
91
107
178
255
218
169
355
227
229
176
75
65
50
127
21
93
35
26
166
49
65
80
15
13
13
33
22
50
13


17
62
54
31
38
34
43
8
42
29
64
45
71
52
100
80
126
205
62
103
49
105
70
11
13
155
25
108
9
46
102
105
38
35
26
61
82
39
62
136
95
68
49
67
35
20
101
93
61
0
85
96
45
45
91
143
121
71
154
130
109
130
27
25
16
46
4
41
20
10
76
9
18
29
4
0
2
3
5
14
1


17
62
54
31
38
34
43
8
42
29
64
45
71
52
100
80
126
205
62
103
49
105
70
11
13
155
25
108
9
46
102
105
38
35
26
61
82
39
62
136
95
68
49
67
35
20
101
93
61
0
85
96
45
45
91
143
121
71
154
130
109
130
27
25
16
46
4
41
20
10
76
9
18
29
4
0
2
3
5
14
1


0
1
0
0
0
0
1
0
1
0
13
0
1
0
0
0
0
0
2
0
1
0
1
0
0
0
0
0
0
0
0
0
0
0
0
0
0
0
0
0
0
0
0
0
0
0
0
0
0
0
0
0
0
0
0
0
0
0
0
0
0
0
0
0
0
0
0
0
0
0
0
0
0
0
0
0
0
0
0
0
0


0
1
0
0
0
0
1
0
1
0
13
0
1
0
0
0
0
0
2
0
1
0
1
0
0
0
0
0
0
0
0
0
0
0
0
0
0
0
0
0
0
0
0
0
0
0
0
0
0
0
0
0
0
0
0
0
0
0
0
0
0
0
0
0
0
0
0
0
0
0
0
0
0
0
0
0
0
0
0
0
0


10
16
55
4
13
12
48
6
9
3
5
7
13
4
19
15
15
16
15
20
16
26
7
0
0
10
0
13
0
14
47
0
119
3
4
6
0
5
7
11
17
8
3
3
3
4
23
10
12
0
7
13
3
5
13
18
8
5
27
21
10
9
4
4
3
7
0
8
0
0
6
3
0
0
0
0
0
0
0
0
0


10
16
55
4
13
12
48
6
9
3
5
7
13
4
19
15
15
16
15
20
16
26
7
0
0
10
0
13
0
14
47
0
119
3
4
6
0
5
7
11
17
8
3
3
3
4
23
10
12
0
7
13
3
5
13
18
8
5
27
21
10
9
4
4
3
7
0
8
0
0
6
3
0
0
0
0
0
0
0
0
0


14
22
58
14
74
42
23
9
14
24
45
37
57
14
67
52
77
54
58
48
32
55
32
0
0
42
0
42
0
184
148
173
86
340
77
79
91
0
45
163
227
120
102
179
189
162
43
78
117
0
15
29
17
25
23
23
28
29
70
24
51
14
20
15
10
28
12
17
8
11
34
11
7
6
1
6
3
8
0
17
7


14
22
58
14
74
42
23
9
14
24
45
37
57
14
67
52
77
54
58
48
32
55
32
0
0
42
0
42
0
184
148
173
86
340
77
79
91
0
45
163
227
120
102
179
189
162
43
78
117
0
15
29
17
25
23
23
28
29
70
24
51
14
20
15
10
28
12
17
8
11
34
11
7
6
1
6
3
8
0
17
7


6
53
24
27
26
23
29
5
23
15
21
36
31
16
43
27
55
71
49
43
26
39
28
1
0
68
0
23
61
26
78
21
20
42
1
54
0
26
31
74
46
24
9
57
45
19
32
50
30
0
10
11
8
19
13
17
27
29
37
24
32
12
6
7
11
33
4
14
4
1
26
20
35
35
3
6
5
13
13
14
4


6
53
22
27
23
23
28
5
23
15
21
35
31
16
43
23
54
71
49
43
26
38
27
1
0
67
0
23
61
26
67
21
20
42
1
53
0
25
30
71
46
23
9
56
45
17
32
50
30
0
10
11
7
17
11
17
27
26
36
24
32
12
6
7
11
32
4
14
3
1
26
19
34
31
3
6
3
13
12
14
4


0
0
0
0
0
0
0
0
0
0
0
0
0
0
0
0
0
0
0
0
0
0
0
0
0
0
0
0
0
0
0
0
0
0
0
0
0
0
0
0
0
0
0
0
0
0
0
0
0
0
0
0
0
0
0
0
0
0
0
0
0
0
0
0
0
0
0
0
0
0
0
0
0
0
0
0
2
0
0
0
0


0
0
2
0
3
0
1
0
0
0
0
1
0
0
0
4
1
0
0
0
0
1
1
0
0
1
0
0
0
0
11
0
0
0
0
1
0
1
1
3
0
1
0
1
0
2
0
0
0
0
0
0
1
2
2
0
0
3
1
0
0
0
0
0
0
1
0
0
1
0
0
1
1
4
0
0
0
0
1
0
0


0
0
1
0
0
0
1
0
2
0
0
0
0
0
2
1
1
3
1
2
0
0
0
0
0
0
0
0
0
1
0
0
0
0
0
6
0
0
0
1
1
0
1
0
0
0
0
0
1
0
0
0
0
0
4
0
1
0
8
0
0
0
1
0
0
0
0
0
0
0
0
0
0
0
0
0
0
0
0
0
0


0
0
1
0
0
0
1
0
2
0
0
0
0
0
2
1
1
3
1
2
0
0
0
0
0
0
0
0
0
1
0
0
0
0
0
6
0
0
0
1
1
0
1
0
0
0
0
0
1
0
0
0
0
0
4
0
1
0
8
0
0
0
1
0
0
0
0
0
0
0
0
0
0
0
0
0
0
0
0
0
0


5
42
65
8
23
10
25
10
20
10
23
9
32
8
49
40
49
64
25
25
39
64
29
60
0
122
0
37
1
23
62
14
52
9
12
14
35
20
29
65
61
26
57
22
22
11
27
28
21
12
16
60
18
13
34
54
33
35
59
28
27
11
17
14
10
13
1
13
3
4
24
6
5
10
7
1
3
9
4
5
1


5
42
65
8
23
10
25
10
20
10
23
9
32
8
49
40
49
64
25
25
39
64
29
60
0
122
0
37
1
23
62
14
52
9
12
14
35
20
29
65
61
26
57
22
22
11
27
28
21
12
16
60
18
13
34
54
33
35
59
28
27
11
17
14
10
13
1
13
3
4
24
6
5
10
7
1
3
9
4
5
1


1
0
0
5
0
0
0
0
0
0
0
0
0
0
6
2
1
4
0
0
2
138
0
0
0
0
0
0
0
0
0
0
0
0
0
4
0
0
0
2
0
0
0
0
0
0
0
0
0
0
0
1
3
0
1
0
0
0
0
5
2
1
0
0
0
1
0
0
0
0
0
0
0
0
0
0
0
0
0
0
0


1
0
0
0
0
0
0
0
0
0
0
0
0
0
0
0
0
1
0
0
2
0
0
0
0
0
0
0
0
0
0
0
0
0
0
0
0
0
0
0
0
0
0
0
0
0
0
0
0
0
0
0
2
0
0
0
0
0
0
5
0
0
0
0
0
0
0
0
0
0
0
0
0
0
0
0
0
0
0
0
0


1
0
0
0
0
0
0
0
0
0
0
0
0
0
0
0
0
1
0
0
2
0
0
0
0
0
0
0
0
0
0
0
0
0
0
0
0
0
0
0
0
0
0
0
0
0
0
0
0
0
0
0
2
0
0
0
0
0
0
5
0
0
0
0
0
0
0
0
0
0
0
0
0
0
0
0
0
0
0
0
0


0
0
0
0
0
0
0
0
0
0
0
0
0
0
3
0
0
0
0
0
0
11
0
0
0
0
0
0
0
0
0
0
0
0
0
1
0
0
0
0
0
0
0
0
0
0
0
0
0
0
0
1
1
0
1
0
0
0
0
0
0
1
0
0
0
0
0
0
0
0
0
0
0
0
0
0
0
0
0
0
0


0
0
0
0
0
0
0
0
0
0
0
0
0
0
2
0
0
0
0
0
0
2
0
0
0
0
0
0
0
0
0
0
0
0
0
0
0
0
0
0
0
0
0
0
0
0
0
0
0
0
0
0
1
0
0
0
0
0
0
0
0
0
0
0
0
0
0
0
0
0
0
0
0
0
0
0
0
0
0
0
0


0
0
0
0
0
0
0
0
0
0
0
0
0
0
1
0
0
0
0
0
0
0
0
0
0
0
0
0
0
0
0
0
0
0
0
0
0
0
0
0
0
0
0
0
0
0
0
0
0
0
0
0
0
0
0
0
0
0
0
0
0
0
0
0
0
0
0
0
0
0
0
0
0
0
0
0
0
0
0
0
0


0
0
0
0
0
0
0
0
0
0
0
0
0
0
0
0
0
0
0
0
0
9
0
0
0
0
0
0
0
0
0
0
0
0
0
1
0
0
0
0
0
0
0
0
0
0
0
0
0
0
0
1
0
0
1
0
0
0
0
0
0
1
0
0
0
0
0
0
0
0
0
0
0
0
0
0
0
0
0
0
0


0
0
0
5
0
0
0
0
0
0
0
0
0
0
3
2
1
3
0
0
0
127
0
0
0
0
0
0
0
0
0
0
0
0
0
3
0
0
0
2
0
0
0
0
0
0
0
0
0
0
0
0
0
0
0
0
0
0
0
0
2
0
0
0
0
1
0
0
0
0
0
0
0
0
0
0
0
0
0
0
0


0
0
0
5
0
0
0
0
0
0
0
0
0
0
3
2
1
3
0
0
0
127
0
0
0
0
0
0
0
0
0
0
0
0
0
3
0
0
0
2
0
0
0
0
0
0
0
0
0
0
0
0
0
0
0
0
0
0
0
0
2
0
0
0
0
1
0
0
0
0
0
0
0
0
0
0
0
0
0
0
0


3
2
19
1
14
7
5
0
4
2
18
12
23
1
16
3
13
11
8
2
1
88
5
0
0
6
0
0
0
6
9
19
0
7
0
14
59
0
2
24
104
17
5
6
3
2
10
0
13
0
1
18
7
5
27
12
8
3
23
2
5
2
2
1
6
4
0
3
1
0
36
2
5
5
11
1
0
6
10
9
2


1
1
16
0
9
6
3
0
0
1
6
5
8
0
8
1
4
5
4
1
0
37
1
0
0
2
0
0
0
0
4
0
0
0
0
4
0
0
1
2
2
4
0
0
0
0
4
0
1
0
0
6
2
2
15
1
2
1
8
1
0
2
0
1
3
1
0
2
1
0
5
0
0
0
0
0
0
0
0
4
0


1
1
16
0
9
6
3
0
0
1
6
5
8
0
8
1
4
5
4
1
0
37
1
0
0
2
0
0
0
0
4
0
0
0
0
4
0
0
1
2
2
4
0
0
0
0
4
0
1
0
0
6
2
2
15
1
2
1
8
1
0
2
0
1
3
1
0
2
1
0
5
0
0
0
0
0
0
0
0
4
0


2
0
2
0
1
0
0
0
2
0
7
2
11
0
7
2
8
5
3
0
0
28
0
0
0
0
0
0
0
0
0
0
0
0
0
0
0
0
0
6
21
1
0
0
0
0
0
0
2
0
0
0
2
1
3
2
2
1
1
1
1
0
0
0
2
1
0
0
0
0
3
0
0
0
0
0
0
0
0
1
0


2
0
2
0
1
0
0
0
2
0
7
2
11
0
7
2
8
5
3
0
0
28
0
0
0
0
0
0
0
0
0
0
0
0
0
0
0
0
0
6
21
1
0
0
0
0
0
0
2
0
0
0
2
1
3
2
2
1
1
1
1
0
0
0
2
1
0
0
0
0
3
0
0
0
0
0
0
0
0
1
0


0
1
0
1
3
0
2
0
2
0
5
4
4
1
1
0
1
1
1
1
1
17
0
0
0
2
0
0
0
4
5
19
0
7
0
9
3
0
0
6
65
6
5
6
3
1
6
0
8
0
0
12
3
0
8
6
4
1
14
0
4
0
1
0
1
2
0
0
0
0
24
1
5
5
11
1
0
5
10
4
2


0
0
0
1
1
0
2
0
2
0
5
1
4
1
1
0
1
1
1
0
0
13
0
0
0
0
0
0
0
1
0
0
0
0
0
5
0
0
0
0
12
0
0
1
0
1
0
0
0
0
0
9
0
0
4
6
0
1
3
0
0
0
0
0
0
0
0
0
0
0
20
0
0
0
0
0
0
2
0
0
0


0
1
0
0
1
0
0
0
0
0
0
0
0
0
0
0
0
0
0
0
1
2
0
0
0
2
0
0
0
3
2
0
0
7
0
1
1
0
0
0
49
5
3
5
3
0
2
0
8
0
0
3
3
0
4
0
4
0
11
0
4
0
0
0
0
1
0
0
0
0
3
1
5
5
11
1
0
3
10
4
2


0
0
0
0
1
0
0
0
0
0
0
3
0
0
0
0
0
0
0
1
0
2
0
0
0
0
0
0
0
0
3
19
0
0
0
3
2
0
0
6
4
1
2
0
0
0
4
0
0
0
0
0
0
0
0
0
0
0
0
0
0
0
1
0
1
1
0
0
0
0
1
0
0
0
0
0
0
0
0
0
0


0
0
1
0
1
1
0
0
0
1
0
1
0
0
0
0
0
0
0
0
0
6
4
0
0
2
0
0
0
2
0
0
0
0
0
1
56
0
1
10
16
6
0
0
0
1
0
0
2
0
1
0
0
2
1
3
0
0
0
0
0
0
1
0
0
0
0
1
0
0
4
1
0
0
0
0
0
1
0
0
0


0
0
1
0
1
1
0
0
0
1
0
1
0
0
0
0
0
0
0
0
0
6
4
0
0
2
0
0
0
2
0
0
0
0
0
1
56
0
1
10
16
6
0
0
0
1
0
0
2
0
1
0
0
2
1
3
0
0
0
0
0
0
1
0
0
0
0
1
0
0
4
1
0
0
0
0
0
1
0
0
0


0
0
0
0
0
0
1
0
0
0
1
0
0
1
0
0
0
1
0
0
3
0
0
1
53
1
86
1
17
2
1
0
0
0
2
0
2
0
0
0
0
0
1
2
0
0
0
2
0
0
0
0
0
0
0
0
1
0
0
1
0
0
0
0
0
0
0
0
0
2
0
0
0
0
0
0
0
0
0
0
0


0
0
0
0
0
0
1
0
0
0
1
0
0
0
0
0
0
1
0
0
3
0
0
0
0
1
0
1
0
2
1
0
0
0
0
0
0
0
0
0
0
0
1
1
0
0
0
2
0
0
0
0
0
0
0
0
1
0
0
1
0
0
0
0
0
0
0
0
0
0
0
0
0
0
0
0
0
0
0
0
0


0
0
0
0
0
0
1
0
0
0
1
0
0
0
0
0
0
1
0
0
3
0
0
0
0
1
0
1
0
2
1
0
0
0
0
0
0
0
0
0
0
0
1
1
0
0
0
2
0
0
0
0
0
0
0
0
1
0
0
1
0
0
0
0
0
0
0
0
0
0
0
0
0
0
0
0
0
0
0
0
0


0
0
0
0
0
0
0
0
0
0
0
0
0
0
0
0
0
0
0
0
0
0
0
1
53
0
86
0
17
0
0
0
0
0
2
0
2
0
0
0
0
0
0
1
0
0
0
0
0
0
0
0
0
0
0
0
0
0
0
0
0
0
0
0
0
0
0
0
0
2
0
0
0
0
0
0
0
0
0
0
0


0
0
0
0
0
0
0
0
0
0
0
0
0
0
0
0
0
0
0
0
0
0
0
1
52
0
86
0
17
0
0
0
0
0
2
0
2
0
0
0
0
0
0
1
0
0
0
0
0
0
0
0
0
0
0
0
0
0
0
0
0
0
0
0
0
0
0
0
0
2
0
0
0
0
0
0
0
0
0
0
0


0
0
0
0
0
0
0
0
0
0
0
0
0
0
0
0
0
0
0
0
0
0
0
0
1
0
0
0
0
0
0
0
0
0
0
0
0
0
0
0
0
0
0
0
0
0
0
0
0
0
0
0
0
0
0
0
0
0
0
0
0
0
0
0
0
0
0
0
0
0
0
0
0
0
0
0
0
0
0
0
0


0
0
0
0
0
0
0
0
0
0
0
0
0
1
0
0
0
0
0
0
0
0
0
0
0
0
0
0
0
0
0
0
0
0
0
0
0
0
0
0
0
0
0
0
0
0
0
0
0
0
0
0
0
0
0
0
0
0
0
0
0
0
0
0
0
0
0
0
0
0
0
0
0
0
0
0
0
0
0
0
0


0
0
0
0
0
0
0
0
0
0
0
0
0
1
0
0
0
0
0
0
0
0
0
0
0
0
0
0
0
0
0
0
0
0
0
0
0
0
0
0
0
0
0
0
0
0
0
0
0
0
0
0
0
0
0
0
0
0
0
0
0
0
0
0
0
0
0
0
0
0
0
0
0
0
0
0
0
0
0
0
0


74
274
352
346
144
321
154
84
107
70
219
137
273
181
509
500
721
456
284
692
185
248
163
89
127
742
101
744
49
456
424
757
367
340
177
350
16
123
339
1206
754
523
190
466
610
238
784
528
716
91
167
346
164
174
147
424
276
364
309
369
238
213
155
71
122
179
58
186
96
146
155
101
216
71
43
39
171
74
48
78
47


15
80
86
190
31
89
40
38
47
14
79
39
85
73
245
196
395
194
86
448
18
22
5
0
0
6
0
157
0
206
181
297
62
79
18
158
0
33
131
248
257
195
49
129
203
78
174
105
367
0
38
133
18
13
31
23
60
97
32
86
27
50
50
8
35
55
18
55
25
47
44
6
85
6
1
14
18
9
3
13
13


15
80
86
190
31
89
40
38
47
14
79
39
85
73
245
196
395
194
86
448
18
22
5
0
0
6
0
157
0
206
181
297
62
79
18
158
0
33
131
248
257
195
49
129
203
78
174
105
367
0
38
133
18
13
31
23
60
97
32
86
27
50
50
8
35
55
18
55
25
47
44
6
85
6
1
14
18
9
3
13
13


5
33
24
18
7
10
10
1
9
4
6
7
30
6
21
32
32
35
13
29
66
37
26
0
65
182
0
85
0
13
28
87
18
27
19
30
0
16
26
106
80
24
13
64
42
27
102
83
38
91
4
17
2
9
6
28
11
9
6
18
5
10
2
0
6
7
1
3
0
0
4
25
33
5
2
2
1
2
3
3
0


5
33
24
18
7
10
10
1
9
4
6
7
30
6
21
32
32
35
13
29
66
37
26
0
65
182
0
85
0
13
28
87
18
27
19
30
0
16
26
106
80
24
13
64
42
27
102
83
38
91
4
17
2
9
6
28
11
9
6
18
5
10
2
0
6
7
1
3
0
0
4
25
33
5
2
2
1
2
3
3
0


1
12
24
20
5
71
15
7
7
3
4
8
8
2
17
15
6
17
11
32
6
13
5
0
34
80
101
20
0
44
14
117
82
33
39
54
0
6
16
44
28
18
6
21
17
11
32
9
9
0
11
27
5
4
3
15
4
25
14
11
21
4
15
1
12
4
20
2
12
3
11
33
43
14
17
13
113
41
13
21
6


1
12
18
20
5
66
15
7
7
3
4
8
8
2
15
14
6
15
10
31
3
12
5
0
34
80
101
20
0
43
10
107
81
31
39
54
0
6
16
44
27
17
6
20
15
9
32
6
9
0
10
24
5
3
3
14
3
20
14
11
20
4
13
1
11
3
19
2
12
3
9
32
39
10
16
13
106
37
13
21
6


0
0
0
0
0
0
0
0
0
0
0
0
0
0
0
0
0
0
0
0
0
0
0
0
0
0
0
0
0
0
0
0
0
0
0
0
0
0
0
0
0
0
0
0
0
0
0
0
0
0
0
0
0
0
0
0
1
1
0
0
0
0
0
0
0
0
0
0
0
0
0
0
0
0
0
0
0
0
0
0
0


0
0
0
0
0
0
0
0
0
0
0
0
0
0
0
0
0
0
0
0
0
0
0
0
0
0
0
0
0
0
0
0
0
0
0
0
0
0
0
0
0
0
0
0
2
0
0
0
0
0
0
0
0
0
0
0
0
0
0
0
0
0
0
0
0
0
0
0
0
0
0
0
0
0
0
0
0
0
0
0
0


0
0
6
0
0
5
0
0
0
0
0
0
0
0
2
1
0
2
1
1
3
1
0
0
0
0
0
0
0
1
4
10
1
2
0
0
0
0
0
0
1
1
0
1
0
2
0
3
0
0
1
3
0
1
0
1
0
4
0
0
1
0
2
0
1
1
1
0
0
0
2
1
4
4
1
0
7
4
0
0
0


0
0
0
0
0
1
0
0
0
0
0
1
1
0
0
0
0
0
0
0
4
3
0
0
0
0
0
0
0
0
0
0
0
0
0
0
0
0
0
2
0
0
1
0
0
0
0
2
2
0
0
0
0
0
0
0
0
0
0
0
0
0
0
0
0
0
0
1
0
0
0
0
0
0
0
0
0
0
0
0
0


0
0
0
0
0
1
0
0
0
0
0
1
1
0
0
0
0
0
0
0
4
3
0
0
0
0
0
0
0
0
0
0
0
0
0
0
0
0
0
2
0
0
1
0
0
0
0
2
2
0
0
0
0
0
0
0
0
0
0
0
0
0
0
0
0
0
0
1
0
0
0
0
0
0
0
0
0
0
0
0
0


49
120
178
90
87
64
81
31
40
30
66
60
114
88
171
192
244
176
134
146
76
134
103
0
28
376
0
457
49
167
167
239
201
171
92
94
15
62
136
699
328
197
101
209
293
102
405
298
259
0
87
148
116
133
89
294
172
198
215
236
146
119
72
59
61
103
18
80
32
95
79
33
45
39
19
6
22
19
26
34
16


46
101
157
79
67
59
71
25
36
19
53
51
100
67
146
161
205
151
95
126
65
110
87
0
28
304
0
366
0
135
148
238
192
162
79
69
15
49
115
585
260
161
70
168
239
89
315
245
226
0
71
122
90
119
77
264
141
173
194
205
123
109
67
50
52
92
15
60
27
81
73
31
37
38
15
5
22
18
26
33
13


0
1
1
2
8
1
3
2
3
1
9
7
10
10
11
19
20
13
25
10
1
11
3
0
0
25
0
10
0
1
0
0
0
0
1
10
0
0
3
8
7
21
1
4
1
0
4
1
1
0
0
3
0
6
2
8
4
1
5
3
2
0
0
2
1
1
0
6
0
0
0
0
2
1
3
1
0
0
0
0
0


3
18
20
9
12
4
7
4
1
10
4
2
4
11
14
12
19
12
14
10
10
13
13
0
0
47
0
81
49
31
19
1
9
9
12
15
0
13
18
106
61
15
30
37
53
13
86
52
32
0
16
23
26
8
10
22
27
24
16
28
21
10
5
7
8
10
3
14
5
14
6
2
6
0
1
0
0
1
0
1
3


0
0
0
0
0
0
0
0
0
0
1
0
0
0
1
0
0
0
0
0
3
0
0
0
0
0
0
0
0
0
0
0
0
0
0
0
0
0
0
0
0
0
0
0
0
0
0
0
0
0
0
0
0
0
0
0
0
0
0
0
0
0
0
0
0
0
0
0
0
0
0
0
0
0
0
0
0
0
0
0
0


0
0
0
0
0
0
0
0
0
0
1
0
0
0
1
0
0
0
0
0
3
0
0
0
0
0
0
0
0
0
0
0
0
0
0
0
0
0
0
0
0
0
0
0
0
0
0
0
0
0
0
0
0
0
0
0
0
0
0
0
0
0
0
0
0
0
0
0
0
0
0
0
0
0
0
0
0
0
0
0
0


0
0
0
0
0
1
0
1
0
0
0
0
0
0
3
0
0
0
0
0
0
1
1
89
0
0
0
0
0
2
4
0
0
0
0
0
0
1
0
0
1
0
0
3
0
1
0
0
0
0
1
0
1
0
0
0
1
0
0
0
0
0
0
0
0
0
0
0
0
0
0
0
3
2
0
1
0
0
0
1
0


0
0
0
0
0
0
0
0
0
0
0
0
0
0
0
0
0
0
0
0
0
1
1
0
0
0
0
0
0
2
4
0
0
0
0
0
0
0
0
0
0
0
0
2
0
1
0
0
0
0
1
0
1
0
0
0
0
0
0
0
0
0
0
0
0
0
0
0
0
0
0
0
3
0
0
1
0
0
0
0
0


0
0
0
0
0
1
0
1
0
0
0
0
0
0
1
0
0
0
0
0
0
0
0
89
0
0
0
0
0
0
0
0
0
0
0
0
0
0
0
0
1
0
0
1
0
0
0
0
0
0
0
0
0
0
0
0
1
0
0
0
0
0
0
0
0
0
0
0
0
0
0
0
0
2
0
0
0
0
0
0
0


0
0
0
0
0
0
0
0
0
0
0
0
0
0
2
0
0
0
0
0
0
0
0
0
0
0
0
0
0
0
0
0
0
0
0
0
0
1
0
0
0
0
0
0
0
0
0
0
0
0
0
0
0
0
0
0
0
0
0
0
0
0
0
0
0
0
0
0
0
0
0
0
0
0
0
0
0
0
0
1
0


0
0
0
0
0
0
0
0
0
0
1
0
0
0
0
0
0
0
0
0
0
0
0
0
0
0
0
0
0
0
0
0
0
0
0
0
0
0
0
0
0
0
0
0
0
0
0
0
0
0
0
0
0
0
0
0
0
0
0
0
0
0
0
0
0
0
0
0
0
0
0
0
0
0
0
0
0
0
0
0
0


0
0
0
0
0
0
0
0
0
0
1
0
0
0
0
0
0
0
0
0
0
0
0
0
0
0
0
0
0
0
0
0
0
0
0
0
0
0
0
0
0
0
0
0
0
0
0
0
0
0
0
0
0
0
0
0
0
0
0
0
0
0
0
0
0
0
0
0
0
0
0
0
0
0
0
0
0
0
0
0
0


4
29
40
28
14
85
8
6
4
19
62
22
35
12
51
65
44
34
40
37
12
38
23
0
0
98
0
25
0
24
30
17
4
30
9
14
1
5
30
107
60
89
20
40
55
19
71
31
41
0
26
21
22
15
18
64
28
35
42
18
39
30
16
3
8
10
1
45
27
1
17
4
7
5
4
3
17
3
3
6
12


4
29
40
28
14
85
8
6
4
19
62
22
35
12
51
65
44
34
40
37
12
38
23
0
0
98
0
25
0
24
30
17
4
30
9
14
1
5
30
107
60
89
20
40
55
19
71
31
41
0
26
21
22
15
18
64
28
35
42
18
39
30
16
3
8
10
1
45
27
1
17
4
7
5
4
3
17
3
3
6
12


0
0
0
0
0
0
0
0
0
0
0
0
0
0
0
0
0
0
0
1
0
4
0
0
0
0
0
0
0
0
0
0
0
0
0
0
0
0
0
0
0
0
0
0
0
0
0
0
0
0
0
0
0
0
0
1
0
0
0
0
0
0
0
0
0
0
0
0
0
0
0
0
0
0
0
0
2
0
0
0
0


0
0
0
0
0
0
0
0
0
0
0
0
0
0
0
0
0
0
0
1
0
4
0
0
0
0
0
0
0
0
0
0
0
0
0
0
0
0
0
0
0
0
0
0
0
0
0
0
0
0
0
0
0
0
0
1
0
0
0
0
0
0
0
0
0
0
0
0
0
0
0
0
0
0
0
0
2
0
0
0
0


0
0
0
0
0
0
0
0
0
0
0
0
0
0
0
0
0
0
0
1
0
4
0
0
0
0
0
0
0
0
0
0
0
0
0
0
0
0
0
0
0
0
0
0
0
0
0
0
0
0
0
0
0
0
0
1
0
0
0
0
0
0
0
0
0
0
0
0
0
0
0
0
0
0
0
0
2
0
0
0
0


6
22
26
61
19
50
34
6
8
7
3
9
7
3
5
14
12
14
16
30
8
11
5
0
0
4
0
8
0
17
42
72
125
58
3
45
42
9
35
19
9
6
0
13
18
6
15
4
12
0
4
13
15
0
12
15
19
17
14
8
10
6
51
5
8
17
7
7
21
7
4
12
17
12
7
13
16
19
2
11
7


4
12
6
28
11
35
5
3
0
4
2
5
1
1
1
5
3
4
4
3
6
9
4
0
0
4
0
4
0
13
33
71
125
56
2
32
42
6
20
6
4
0
0
9
17
5
4
4
7
0
1
0
4
0
3
6
6
6
8
1
2
0
38
0
1
2
4
2
6
6
0
11
14
10
4
13
12
15
0
11
4


4
12
6
28
11
35
5
3
0
4
2
5
1
1
1
5
3
4
4
3
6
9
4
0
0
4
0
4
0
13
33
71
125
56
2
32
42
6
20
6
4
0
0
9
17
5
4
4
7
0
1
0
4
0
3
6
6
6
8
1
2
0
38
0
1
2
4
2
6
6
0
11
14
10
4
13
12
15
0
11
4


2
10
20
33
8
15
29
3
8
3
1
4
6
2
4
9
9
10
12
27
2
2
1
0
0
0
0
4
0
3
9
1
0
2
1
12
0
3
15
13
5
6
0
4
1
1
11
0
5
0
3
13
11
0
9
9
13
11
6
7
8
6
13
5
7
15
3
5
15
1
4
1
3
2
3
0
4
4
2
0
3


2
10
20
33
8
15
29
3
8
3
1
4
6
2
4
9
9
10
12
27
2
2
1
0
0
0
0
4
0
3
9
1
0
2
1
12
0
3
15
13
5
6
0
4
1
1
11
0
5
0
3
13
11
0
9
9
13
11
6
7
8
6
13
5
7
15
3
5
15
1
4
1
3
2
3
0
4
4
2
0
3


0
0
0
0
0
0
0
0
0
0
0
0
0
0
0
0
0
0
0
0
0
0
0
0
0
0
0
0
0
1
0
0
0
0
0
1
0
0
0
0
0
0
0
0
0
0
0
0
0
0
0
0
0
0
0
0
0
0
0
0
0
0
0
0
0
0
0
0
0
0
0
0
0
0
0
0
0
0
0
0
0


0
0
0
0
0
0
0
0
0
0
0
0
0
0
0
0
0
0
0
0
0
0
0
0
0
0
0
0
0
1
0
0
0
0
0
1
0
0
0
0
0
0
0
0
0
0
0
0
0
0
0
0
0
0
0
0
0
0
0
0
0
0
0
0
0
0
0
0
0
0
0
0
0
0
0
0
0
0
0
0
0


64
130
322
267
434
578
408
126
196
281
454
247
421
112
165
182
302
269
626
227
66
531
76
0
0
316
0
102
313
148
128
82
150
81
70
295
59
123
88
331
131
138
97
113
93
89
125
97
137
0
110
129
70
430
268
297
64
213
351
142
378
126
149
79
61
109
77
161
31
227
69
108
78
69
26
66
36
118
51
36
19


2
11
16
4
3
13
14
0
0
8
53
1
84
12
2
5
15
2
44
1
0
23
8
0
0
1
0
1
0
0
0
0
0
0
0
1
0
0
1
0
12
4
0
2
0
1
1
0
9
0
1
0
3
0
1
0
1
0
1
12
0
1
1
0
2
2
0
0
0
0
0
0
1
0
0
0
0
2
2
0
0


2
11
16
4
3
13
14
0
0
8
53
1
84
12
2
5
15
2
44
1
0
23
8
0
0
1
0
1
0
0
0
0
0
0
0
1
0
0
1
0
12
4
0
2
0
1
1
0
9
0
1
0
3
0
1
0
1
0
1
12
0
1
1
0
2
2
0
0
0
0
0
0
1
0
0
0
0
2
2
0
0


15
0
27
49
35
74
5
10
10
55
15
16
3
3
8
4
1
0
7
37
0
9
3
0
0
0
0
0
0
7
5
0
0
5
2
35
0
0
0
0
0
1
1
3
6
1
0
0
2
0
3
18
23
0
2
2
2
4
13
4
6
5
29
0
8
4
40
0
6
173
0
11
2
20
4
4
2
8
16
0
1


15
0
27
49
35
74
5
10
10
55
15
16
3
3
8
4
1
0
7
37
0
9
3
0
0
0
0
0
0
7
5
0
0
5
2
35
0
0
0
0
0
1
1
3
6
1
0
0
2
0
3
18
23
0
2
2
2
4
13
4
6
5
29
0
8
4
40
0
6
173
0
11
2
20
4
4
2
8
16
0
1


0
0
0
0
0
1
0
0
0
0
0
0
0
0
0
0
0
0
1
0
0
1
0
0
0
0
0
0
0
0
0
0
0
0
0
0
0
0
0
4
0
0
0
0
1
0
0
0
0
0
0
0
0
0
0
0
0
0
0
0
0
0
1
0
0
1
0
0
0
0
0
0
1
0
3
0
0
0
0
0
0


0
0
0
0
0
1
0
0
0
0
0
0
0
0
0
0
0
0
1
0
0
1
0
0
0
0
0
0
0
0
0
0
0
0
0
0
0
0
0
4
0
0
0
0
1
0
0
0
0
0
0
0
0
0
0
0
0
0
0
0
0
0
1
0
0
1
0
0
0
0
0
0
1
0
3
0
0
0
0
0
0


11
53
236
38
57
90
43
12
75
52
143
69
176
35
77
98
208
202
291
120
57
288
50
0
0
167
0
90
312
69
87
72
149
61
60
183
59
112
54
174
107
60
45
59
58
46
94
85
111
0
37
49
26
26
30
54
13
58
91
43
86
36
30
23
20
46
19
48
13
14
26
29
17
30
10
7
10
55
23
18
7


0
0
0
3
1
0
0
0
0
0
1
4
1
0
0
3
0
2
6
0
6
1
7
0
0
0
0
10
0
1
1
0
5
0
0
0
0
3
8
103
1
6
1
1
7
0
19
5
7
0
0
0
0
0
0
3
0
2
1
5
1
0
2
3
0
0
0
1
0
0
3
0
0
0
0
0
0
0
0
0
0


0
1
2
0
1
0
0
0
0
0
1
0
1
0
0
1
0
0
0
0
1
2
0
0
0
1
0
1
0
0
1
0
0
0
0
0
0
1
0
0
0
0
0
0
2
0
0
0
0
0
1
0
0
0
0
1
1
0
0
0
1
0
0
0
0
3
0
0
0
0
0
0
0
0
0
0
0
0
0
0
0


0
0
0
0
0
0
1
0
0
0
0
0
0
0
0
0
0
0
0
0
0
0
0
0
0
0
0
0
0
0
0
0
0
0
0
0
0
0
0
0
0
0
0
0
0
0
0
0
0
0
1
0
0
0
0
0
1
0
0
0
0
0
0
0
0
0
0
0
0
0
0
0
0
0
0
0
0
0
0
0
0


3
5
18
16
9
32
14
5
7
8
6
8
57
0
13
8
35
11
51
2
1
36
2
0
0
10
0
9
196
15
11
1
0
37
13
27
0
0
11
10
19
13
12
6
8
16
10
0
15
0
2
4
3
1
1
1
3
7
5
3
4
3
2
4
5
11
0
3
0
0
3
1
2
0
0
2
0
0
1
1
0


0
0
0
0
2
0
0
0
2
0
0
0
0
0
0
0
1
0
0
1
0
0
0
0
0
29
0
1
0
0
0
0
2
0
0
25
0
0
0
1
2
0
2
7
0
0
0
1
1
0
0
0
0
0
0
1
2
2
0
0
0
0
0
0
0
0
0
0
0
0
0
0
0
2
0
0
1
0
0
0
2


1
3
91
0
8
3
3
1
8
12
38
7
52
7
7
30
22
40
77
40
24
47
15
0
0
10
0
22
114
4
0
0
92
0
16
33
0
46
2
4
20
4
3
1
4
3
11
8
33
0
7
1
1
0
6
3
0
3
2
3
2
3
0
0
0
2
10
1
0
0
1
0
3
0
0
1
2
9
4
0
0


0
0
0
0
0
0
0
0
0
0
0
0
0
0
0
0
0
0
0
0
0
0
0
0
0
0
0
0
0
0
0
0
0
0
0
0
0
0
0
0
0
0
0
1
0
0
0
0
0
0
0
0
0
0
0
0
0
0
0
0
0
0
0
2
0
0
0
0
2
0
0
0
0
0
0
0
0
0
0
0
0


0
21
3
10
6
18
6
1
21
9
50
10
39
2
28
20
48
73
13
34
10
13
0
0
0
7
0
26
0
1
16
0
0
0
0
1
0
18
5
0
2
0
5
12
0
3
23
21
16
0
12
0
0
10
7
18
1
6
27
13
19
14
4
6
3
3
2
12
1
10
7
0
0
0
0
0
0
1
0
0
0


0
0
0
0
0
4
0
0
0
0
0
0
0
0
0
0
0
0
0
0
0
0
0
0
0
0
0
0
0
0
0
0
0
0
0
0
0
0
0
0
0
0
0
0
0
0
0
0
0
0
2
0
0
0
0
0
0
0
0
0
0
0
0
0
0
0
0
0
0
0
0
0
0
0
0
0
0
0
0
0
0


7
23
122
9
30
33
19
5
37
23
47
40
26
26
29
36
102
76
144
43
15
189
26
0
0
110
0
21
2
48
58
71
50
24
31
97
59
44
28
56
63
37
22
31
37
24
31
50
39
0
12
44
22
15
16
27
5
38
56
19
59
16
22
8
12
27
7
31
10
4
12
28
12
28
10
4
7
45
18
17
5


36
66
43
176
339
400
346
104
111
166
243
161
158
62
78
75
78
65
283
69
9
210
15
0
0
148
0
11
1
72
36
10
1
15
8
76
0
11
33
153
12
73
51
49
28
41
30
12
15
0
69
62
18
404
235
241
48
151
246
83
286
84
88
56
31
56
18
113
12
40
43
68
57
19
9
55
24
53
10
18
11


36
66
43
176
339
400
346
104
111
166
243
161
158
62
78
75
78
65
283
69
9
210
15
0
0
148
0
11
1
72
36
10
1
15
8
76
0
11
33
153
12
73
51
49
28
41
30
12
15
0
69
62
18
404
235
241
48
151
246
83
286
84
88
56
31
56
18
113
12
40
43
68
57
19
9
55
24
53
10
18
11


0
1
1
0
4
5
11
4
6
3
10
7
8
7
3
4
10
8
41
3
11
13
16
0
0
3
0
26
0
2
8
2
4
21
16
12
0
1
7
16
30
9
11
15
11
6
24
5
11
0
7
7
7
5
16
21
13
15
20
8
22
5
8
7
2
9
1
5
4
0
6
7
3
2
4
0
3
2
1
2
0


0
0
0
0
0
0
1
0
1
0
0
0
1
0
0
0
0
4
0
1
0
2
3
0
0
0
0
2
0
1
6
0
0
17
8
3
0
1
1
3
18
1
5
11
4
1
4
1
6
0
1
0
1
0
5
1
1
6
5
0
6
1
4
1
0
6
1
0
0
0
2
5
0
0
0
0
0
0
1
0
0


0
0
0
0
0
0
1
0
1
0
0
0
1
0
0
0
0
4
0
1
0
2
3
0
0
0
0
2
0
1
6
0
0
17
8
3
0
1
1
3
18
1
5
11
4
1
4
1
6
0
1
0
1
0
5
1
1
6
5
0
6
1
4
1
0
6
1
0
0
0
2
5
0
0
0
0
0
0
1
0
0


0
0
0
0
3
3
7
4
5
3
8
6
5
6
3
4
7
3
27
2
1
6
8
0
0
0
0
6
0
0
2
2
0
0
4
5
0
0
0
3
4
2
1
0
0
0
5
0
0
0
1
4
2
3
7
12
6
3
9
5
10
3
0
2
2
1
0
5
2
0
3
0
1
0
0
0
0
0
0
1
0


0
0
0
0
3
3
6
3
4
3
4
6
5
6
3
3
7
3
26
2
0
4
8
0
0
0
0
6
0
0
2
2
0
0
4
4
0
0
0
1
3
0
1
0
0
0
4
0
0
0
1
4
2
3
6
6
5
2
7
4
9
3
0
0
2
1
0
3
2
0
3
0
1
0
0
0
0
0
0
1
0


0
0
0
0
0
0
0
0
0
0
0
0
0
0
0
0
0
0
0
0
1
0
0
0
0
0
0
0
0
0
0
0
0
0
0
0
0
0
0
1
0
2
0
0
0
0
0
0
0
0
0
0
0
0
0
0
0
0
0
0
0
0
0
0
0
0
0
0
0
0
0
0
0
0
0
0
0
0
0
0
0


0
0
0
0
0
0
1
1
1
0
4
0
0
0
0
1
0
0
1
0
0
2
0
0
0
0
0
0
0
0
0
0
0
0
0
1
0
0
0
1
1
0
0
0
0
0
1
0
0
0
0
0
0
0
1
6
1
1
2
1
1
0
0
2
0
0
0
2
0
0
0
0
0
0
0
0
0
0
0
0
0


0
1
0
0
0
0
1
0
0
0
0
0
0
0
0
0
0
1
0
0
1
1
2
0
0
0
0
13
0
0
0
0
0
0
4
0
0
0
0
0
4
1
1
0
0
0
3
0
1
0
0
1
1
1
2
6
0
0
3
0
3
1
1
0
0
0
0
0
0
0
0
0
2
0
1
0
2
0
0
0
0


0
1
0
0
0
0
1
0
0
0
0
0
0
0
0
0
0
1
0
0
1
1
2
0
0
0
0
13
0
0
0
0
0
0
4
0
0
0
0
0
4
1
1
0
0
0
3
0
1
0
0
1
1
1
2
6
0
0
3
0
3
1
1
0
0
0
0
0
0
0
0
0
2
0
1
0
2
0
0
0
0


0
0
1
0
1
0
0
0
0
0
1
0
0
0
0
0
1
0
1
0
0
2
3
0
0
3
0
3
0
0
0
0
4
4
0
4
0
0
6
2
2
2
3
2
2
1
8
4
2
0
1
2
2
1
2
1
2
4
2
3
1
0
3
1
0
1
0
0
0
0
0
2
0
0
2
0
1
2
0
1
0


0
0
0
0
0
0
0
0
0
0
1
0
0
0
0
0
0
0
1
0
0
0
3
0
0
3
0
3
0
0
0
0
4
4
0
4
0
0
6
2
0
2
2
1
2
1
7
4
2
0
1
2
2
1
2
1
2
2
0
3
1
0
2
1
0
1
0
0
0
0
0
2
0
0
2
0
1
2
0
1
0


0
0
1
0
0
0
0
0
0
0
0
0
0
0
0
0
1
0
0
0
0
0
0
0
0
0
0
0
0
0
0
0
0
0
0
0
0
0
0
0
0
0
0
0
0
0
0
0
0
0
0
0
0
0
0
0
0
1
0
0
0
0
0
0
0
0
0
0
0
0
0
0
0
0
0
0
0
0
0
0
0


0
0
0
0
1
0
0
0
0
0
0
0
0
0
0
0
0
0
0
0
0
2
0
0
0
0
0
0
0
0
0
0
0
0
0
0
0
0
0
0
2
0
1
1
0
0
1
0
0
0
0
0
0
0
0
0
0
1
2
0
0
0
1
0
0
0
0
0
0
0
0
0
0
0
0
0
0
0
0
0
0


0
0
0
0
0
2
2
0
0
0
1
1
2
1
0
0
2
0
13
0
9
2
0
0
0
0
0
2
0
1
0
0
0
0
0
0
0
0
0
8
2
3
1
2
5
4
4
0
2
0
4
0
1
0
0
1
4
2
1
0
2
0
0
3
0
1
0
0
2
0
1
0
0
2
1
0
0
0
0
0
0


0
0
0
0
0
2
2
0
0
0
1
1
2
1
0
0
2
0
13
0
9
2
0
0
0
0
0
2
0
1
0
0
0
0
0
0
0
0
0
8
2
3
1
2
5
4
4
0
2
0
4
0
1
0
0
1
4
2
1
0
2
0
0
3
0
1
0
0
2
0
1
0
0
2
1
0
0
0
0
0
0


5
13
33
24
15
59
6
7
6
9
8
22
14
7
17
33
18
108
6
20
14
43
14
0
0
48
0
21
1168
33
33
26
28
60
20
105
0
11
32
73
51
39
13
47
55
26
27
14
70
0
17
57
25
11
17
11
24
60
40
28
52
22
78
9
23
20
29
7
13
42
22
27
39
32
11
31
15
14
30
9
15


0
0
1
1
0
1
0
3
0
3
1
1
1
1
0
1
2
1
0
3
0
8
1
0
0
0
0
1
1
3
4
0
0
0
0
20
0
0
2
19
1
13
1
2
0
13
6
3
0
0
0
11
1
0
0
1
0
3
2
2
4
1
12
0
2
5
0
0
3
0
2
0
0
12
0
0
2
3
0
0
0


0
0
1
1
0
1
0
3
0
3
1
1
1
1
0
1
2
1
0
3
0
8
1
0
0
0
0
1
1
3
4
0
0
0
0
20
0
0
2
19
1
13
1
2
0
13
6
3
0
0
0
11
1
0
0
1
0
3
2
2
4
1
12
0
2
5
0
0
3
0
2
0
0
12
0
0
2
3
0
0
0


1
0
0
4
4
10
0
0
0
1
2
2
2
0
1
6
4
6
4
0
2
6
1
0
0
5
0
1
0
1
0
0
17
24
2
6
0
0
2
10
1
3
1
6
3
3
4
2
4
0
3
7
3
4
5
2
2
2
3
2
6
1
12
0
0
2
3
2
0
6
2
8
14
7
4
17
7
3
0
1
1


1
0
0
3
0
2
0
0
0
0
0
0
0
0
0
0
2
0
0
0
2
2
0
0
0
1
0
0
0
0
0
0
0
0
0
0
0
0
0
0
0
0
0
1
0
1
0
0
1
0
0
1
1
0
2
0
0
0
0
0
0
1
1
0
0
0
0
0
0
0
0
2
0
0
1
0
0
0
0
0
0


0
0
0
0
3
1
0
0
0
1
2
2
2
0
1
4
2
6
4
0
0
4
1
0
0
4
0
1
0
0
0
0
17
11
2
3
0
0
2
8
1
2
0
5
3
2
4
1
3
0
2
0
0
4
2
2
2
2
1
2
4
0
8
0
0
2
1
1
0
6
1
2
3
0
0
3
2
1
0
1
0


0
0
0
1
1
7
0
0
0
0
0
0
0
0
0
2
0
0
0
0
0
0
0
0
0
0
0
0
0
1
0
0
0
13
0
3
0
0
0
2
0
1
1
0
0
0
0
1
0
0
1
6
2
0
1
0
0
0
2
0
2
0
3
0
0
0
2
1
0
0
1
4
11
7
3
14
5
2
0
0
1


1
8
10
0
1
0
0
0
2
0
1
0
2
0
2
9
1
14
2
3
0
0
1
0
0
0
0
4
0
5
2
0
0
13
0
0
0
3
3
13
7
4
0
0
0
2
0
2
3
0
2
0
3
5
5
1
15
15
7
13
10
3
0
7
5
3
3
0
3
2
3
2
0
0
0
0
0
0
0
4
0


1
8
10
0
1
0
0
0
2
0
1
0
2
0
2
9
1
14
2
3
0
0
1
0
0
0
0
4
0
5
2
0
0
13
0
0
0
3
3
13
7
4
0
0
0
2
0
2
3
0
2
0
3
5
5
1
15
15
7
13
10
3
0
7
5
3
3
0
3
2
3
2
0
0
0
0
0
0
0
4
0


0
0
1
1
9
17
1
2
0
2
0
17
1
0
0
12
3
74
0
4
3
9
2
0
0
1
0
11
1
18
15
0
7
0
16
7
0
0
12
8
29
3
6
11
14
5
3
3
12
0
0
0
0
0
0
1
3
19
3
3
20
1
1
0
2
1
0
0
0
0
0
1
1
2
5
4
2
2
0
4
0


0
0
0
0
0
0
0
0
0
0
0
0
0
0
0
0
0
0
0
0
2
0
0
0
0
0
0
0
0
0
0
0
0
0
0
0
0
0
0
4
0
0
0
0
0
0
0
0
0
0
0
0
0
0
0
0
0
0
0
0
0
0
0
0
0
0
0
0
0
0
0
0
1
0
0
0
0
0
0
0
0


0
0
0
1
9
17
1
2
0
2
0
16
1
0
0
9
3
72
0
4
1
7
2
0
0
1
0
5
0
9
12
0
7
0
5
6
0
0
7
3
10
3
5
5
14
5
3
3
8
0
0
0
0
0
0
1
3
19
3
3
20
0
1
0
2
1
0
0
0
0
0
1
0
2
5
4
2
2
0
4
0


0
0
1
0
0
0
0
0
0
0
0
1
0
0
0
3
0
2
0
0
0
2
0
0
0
0
0
6
1
9
3
0
0
0
11
1
0
0
5
1
19
0
1
6
0
0
0
0
4
0
0
0
0
0
0
0
0
0
0
0
0
1
0
0
0
0
0
0
0
0
0
0
0
0
0
0
0
0
0
0
0


3
5
21
18
1
31
5
2
4
3
4
2
8
6
14
5
8
13
0
10
9
20
9
0
0
42
0
4
1166
6
12
26
4
23
2
72
0
8
13
23
13
16
5
28
38
3
14
4
51
0
12
39
18
2
7
6
4
21
25
8
12
16
53
2
14
9
23
5
7
34
15
16
24
11
2
10
4
6
30
0
14


3
5
21
18
1
31
5
2
4
3
4
2
8
6
14
5
8
13
0
10
9
20
9
0
0
42
0
4
1166
6
12
26
4
23
2
72
0
8
13
23
13
16
5
28
38
3
14
4
51
0
12
39
18
2
7
6
4
21
25
8
12
16
53
2
14
9
23
5
7
34
15
16
24
11
2
10
4
6
30
0
14


0
0
0
0
0
0
0
0
0
0
0
0
0
0
0
0
0
0
0
0
0
1
0
0
0
0
0
0
0
0
0
0
0
0
0
1
0
0
0
0
0
0
0
0
0
0
0
0
0
0
0
1
0
0
0
0
0
0
0
0
0
0
1
0
0
0
0
0
0
0
0
0
0
0
0
0
0
0
0
0
0


0
0
0
0
0
0
0
0
0
0
0
0
0
0
0
0
0
0
0
0
0
1
0
0
0
0
0
0
0
0
0
0
0
0
0
1
0
0
0
0
0
0
0
0
0
0
0
0
0
0
0
1
0
0
0
0
0
0
0
0
0
0
1
0
0
0
0
0
0
0
0
0
0
0
0
0
0
0
0
0
0


0
0
0
0
0
0
0
0
0
0
0
0
0
0
0
0
0
0
0
0
0
1
0
0
0
0
0
0
0
0
0
0
0
0
0
1
0
0
0
0
0
0
0
0
0
0
0
0
0
0
0
1
0
0
0
0
0
0
0
0
0
0
1
0
0
0
0
0
0
0
0
0
0
0
0
0
0
0
0
0
0


0
0
0
0
0
0
0
0
0
0
0
0
0
0
0
1
0
0
0
0
0
0
0
0
0
0
0
0
0
0
0
0
0
0
0
0
0
0
0
0
0
0
0
0
0
0
0
0
1
0
0
0
0
0
0
0
0
0
0
0
0
0
0
0
0
0
0
0
0
0
0
0
0
0
0
0
0
0
0
0
0


0
0
0
0
0
0
0
0
0
0
0
0
0
0
0
1
0
0
0
0
0
0
0
0
0
0
0
0
0
0
0
0
0
0
0
0
0
0
0
0
0
0
0
0
0
0
0
0
1
0
0
0
0
0
0
0
0
0
0
0
0
0
0
0
0
0
0
0
0
0
0
0
0
0
0
0
0
0
0
0
0


0
0
0
0
0
0
0
0
0
0
0
0
0
0
0
1
0
0
0
0
0
0
0
0
0
0
0
0
0
0
0
0
0
0
0
0
0
0
0
0
0
0
0
0
0
0
0
0
1
0
0
0
0
0
0
0
0
0
0
0
0
0
0
0
0
0
0
0
0
0
0
0
0
0
0
0
0
0
0
0
0


0
0
0
0
0
0
0
0
0
0
0
0
0
0
0
0
0
0
0
0
0
0
0
0
0
0
0
0
0
0
0
0
0
0
0
0
0
0
0
1
0
0
0
0
0
0
0
0
0
0
0
0
0
0
0
0
0
0
0
0
0
0
0
0
0
0
0
0
0
0
0
0
0
0
0
0
0
0
0
0
0


0
0
0
0
0
0
0
0
0
0
0
0
0
0
0
0
0
0
0
0
0
0
0
0
0
0
0
0
0
0
0
0
0
0
0
0
0
0
0
1
0
0
0
0
0
0
0
0
0
0
0
0
0
0
0
0
0
0
0
0
0
0
0
0
0
0
0
0
0
0
0
0
0
0
0
0
0
0
0
0
0


0
0
0
0
0
0
0
0
0
0
0
0
0
0
0
0
0
0
0
0
0
0
0
0
0
0
0
0
0
0
0
0
0
0
0
0
0
0
0
1
0
0
0
0
0
0
0
0
0
0
0
0
0
0
0
0
0
0
0
0
0
0
0
0
0
0
0
0
0
0
0
0
0
0
0
0
0
0
0
0
0


135
210
306
702
274
682
186
204
151
238
221
301
151
147
364
394
482
397
248
629
200
406
219
2
27
326
53
259
9
517
883
1601
425
2683
237
1559
491
83
330
1110
535
359
234
595
1040
525
413
284
635
0
182
851
385
137
167
285
487
403
404
310
499
203
967
85
412
290
417
123
290
372
372
1496
1065
905
486
1221
793
661
730
405
486


135
210
306
702
274
682
186
204
151
238
221
301
151
147
364
394
482
397
248
629
200
406
219
2
27
326
53
259
9
517
883
1601
425
2683
237
1559
491
83
330
1110
535
359
234
595
1040
525
413
284
635
0
182
851
385
137
167
285
487
403
404
310
499
203
967
85
412
290
417
123
290
372
372
1496
1065
905
486
1221
793
661
730
405
486


135
210
306
702
274
682
186
204
151
238
221
301
151
147
364
394
482
397
248
629
200
406
219
2
27
326
53
259
9
517
883
1601
425
2683
237
1559
491
83
330
1110
535
359
234
595
1040
525
413
284
635
0
182
851
385
137
167
285
487
403
404
310
499
203
967
85
412
290
417
123
290
372
372
1496
1065
905
486
1221
793
661
730
405
486


0
0
0
0
2
3
1
0
1
0
0
0
0
0
0
0
0
0
0
0
0
9
0
0
0
0
0
0
0
0
0
0
0
0
1
0
0
4
0
0
0
0
1
0
0
0
0
1
3
0
2
12
0
3
6
5
0
9
1
1
13
5
2
1
0
1
0
2
0
0
0
0
0
0
0
0
0
0
0
0
0


0
0
0
0
2
3
1
0
1
0
0
0
0
0
0
0
0
0
0
0
0
9
0
0
0
0
0
0
0
0
0
0
0
0
1
0
0
4
0
0
0
0
1
0
0
0
0
1
3
0
2
12
0
3
6
5
0
9
1
1
13
5
2
1
0
1
0
2
0
0
0
0
0
0
0
0
0
0
0
0
0


0
0
0
0
2
3
1
0
1
0
0
0
0
0
0
0
0
0
0
0
0
9
0
0
0
0
0
0
0
0
0
0
0
0
1
0
0
4
0
0
0
0
1
0
0
0
0
1
3
0
2
12
0
3
6
5
0
9
1
1
13
5
2
1
0
1
0
2
0
0
0
0
0
0
0
0
0
0
0
0
0


0
0
0
0
2
3
1
0
1
0
0
0
0
0
0
0
0
0
0
0
0
9
0
0
0
0
0
0
0
0
0
0
0
0
1
0
0
4
0
0
0
0
1
0
0
0
0
1
3
0
2
12
0
3
6
5
0
9
1
1
13
5
2
1
0
1
0
2
0
0
0
0
0
0
0
0
0
0
0
0
0


45
48
79
69
56
21
32
48
34
47
13
17
8
10
37
23
102
48
67
25
124
99
37
0
0
34
0
85
0
118
119
54
36
145
215
31
0
15
27
125
133
113
114
130
220
285
90
88
62
118
5
1
1
4
28
3
8
3
5
14
8
11
3
3
0
3
1
12
1
1
3
0
0
0
0
0
0
0
0
0
0


45
48
79
69
56
21
32
48
34
47
13
17
8
10
37
23
102
48
67
25
124
99
37
0
0
34
0
85
0
118
119
54
36
145
215
31
0
15
27
125
133
113
114
130
220
285
90
88
62
118
5
1
1
4
28
3
8
3
5
14
8
11
3
3
0
3
1
12
1
1
3
0
0
0
0
0
0
0
0
0
0


45
48
79
69
56
21
32
48
34
47
13
17
8
10
37
23
102
48
67
25
124
99
37
0
0
34
0
85
0
118
119
54
36
145
215
31
0
15
27
125
133
113
114
130
220
285
90
88
62
118
5
1
1
4
28
3
8
3
5
14
8
11
3
3
0
3
1
12
1
1
3
0
0
0
0
0
0
0
0
0
0


45
48
79
69
56
21
32
48
34
47
13
17
8
10
37
23
102
48
67
25
124
99
37
0
0
34
0
85
0
118
119
54
36
145
215
31
0
15
27
125
133
113
114
130
220
285
90
88
62
118
5
1
1
4
28
3
8
3
5
14
8
11
3
3
0
3
1
12
1
1
3
0
0
0
0
0
0
0
0
0
0


6
10
22
18
12
13
9
8
10
11
14
15
9
6
25
39
22
18
54
51
14
6
10
0
0
1
0
6
0
19
8
2
5
1
5
10
1
3
17
17
17
15
9
8
12
14
18
11
21
0
14
16
10
2
10
2
13
22
13
13
12
13
5
1
4
15
4
2
0
1
11
1
7
6
1
4
2
2
2
3
4


6
10
22
18
12
13
9
8
10
11
14
15
9
6
25
39
22
18
54
51
14
6
10
0
0
1
0
6
0
19
8
2
5
1
5
10
1
3
17
17
17
15
9
8
12
14
18
11
21
0
14
16
10
2
10
2
13
22
13
13
12
13
5
1
4
15
4
2
0
1
11
1
7
6
1
4
2
2
2
3
4


6
10
22
18
12
13
9
8
10
11
14
15
9
6
25
39
22
18
54
51
14
6
10
0
0
1
0
6
0
19
8
2
5
1
5
10
1
3
17
17
17
15
9
8
12
14
18
11
21
0
14
16
10
2
10
2
13
22
13
13
12
13
5
1
4
15
4
2
0
1
11
1
7
6
1
4
2
2
2
3
4


6
10
22
18
12
13
9
8
10
11
14
15
9
6
25
39
22
18
54
51
14
6
10
0
0
1
0
6
0
19
8
2
5
1
5
10
1
3
17
17
17
15
9
8
12
14
18
11
21
0
14
16
10
2
10
2
13
22
13
13
12
13
5
1
4
15
4
2
0
1
11
1
7
6
1
4
2
2
2
3
4


0
0
0
0
0
0
0
0
0
1
1
0
0
0
0
2
0
0
0
0
0
0
0
0
160
0
0
0
0
0
0
0
0
0
0
0
67
0
0
0
0
0
0
0
0
0
0
0
0
0
0
0
0
0
0
0
1
0
2
0
0
0
0
0
0
0
0
0
0
0
0
0
0
0
0
0
0
0
0
0
0


0
0
0
0
0
0
0
0
0
1
1
0
0
0
0
2
0
0
0
0
0
0
0
0
160
0
0
0
0
0
0
0
0
0
0
0
67
0
0
0
0
0
0
0
0
0
0
0
0
0
0
0
0
0
0
0
1
0
2
0
0
0
0
0
0
0
0
0
0
0
0
0
0
0
0
0
0
0
0
0
0


0
0
0
0
0
0
0
0
0
1
1
0
0
0
0
2
0
0
0
0
0
0
0
0
160
0
0
0
0
0
0
0
0
0
0
0
67
0
0
0
0
0
0
0
0
0
0
0
0
0
0
0
0
0
0
0
1
0
2
0
0
0
0
0
0
0
0
0
0
0
0
0
0
0
0
0
0
0
0
0
0


0
0
0
0
0
0
0
0
0
1
1
0
0
0
0
2
0
0
0
0
0
0
0
0
40
0
0
0
0
0
0
0
0
0
0
0
0
0
0
0
0
0
0
0
0
0
0
0
0
0
0
0
0
0
0
0
1
0
2
0
0
0
0
0
0
0
0
0
0
0
0
0
0
0
0
0
0
0
0
0
0


0
0
0
0
0
0
0
0
0
1
1
0
0
0
0
2
0
0
0
0
0
0
0
0
40
0
0
0
0
0
0
0
0
0
0
0
0
0
0
0
0
0
0
0
0
0
0
0
0
0
0
0
0
0
0
0
1
0
2
0
0
0
0
0
0
0
0
0
0
0
0
0
0
0
0
0
0
0
0
0
0


0
0
0
0
0
0
0
0
0
0
0
0
0
0
0
0
0
0
0
0
0
0
0
0
120
0
0
0
0
0
0
0
0
0
0
0
67
0
0
0
0
0
0
0
0
0
0
0
0
0
0
0
0
0
0
0
0
0
0
0
0
0
0
0
0
0
0
0
0
0
0
0
0
0
0
0
0
0
0
0
0


0
0
0
0
0
0
0
0
0
0
0
0
0
0
0
0
0
0
0
0
0
0
0
0
1
0
0
0
0
0
0
0
0
0
0
0
3
0
0
0
0
0
0
0
0
0
0
0
0
0
0
0
0
0
0
0
0
0
0
0
0
0
0
0
0
0
0
0
0
0
0
0
0
0
0
0
0
0
0
0
0


0
0
0
0
0
0
0
0
0
0
0
0
0
0
0
0
0
0
0
0
0
0
0
0
119
0
0
0
0
0
0
0
0
0
0
0
64
0
0
0
0
0
0
0
0
0
0
0
0
0
0
0
0
0
0
0
0
0
0
0
0
0
0
0
0
0
0
0
0
0
0
0
0
0
0
0
0
0
0
0
0


99
210
440
143
209
199
210
255
42
96
48
81
56
69
92
113
364
71
159
160
315
292
104
0
0
57
0
164
72
148
132
10
9
105
236
22
97
102
135
107
112
201
306
141
111
186
133
224
122
335
111
29
50
241
148
164
125
146
239
467
279
143
19
125
45
120
124
83
165
33
43
15
13
15
2
19
20
28
6
7
13


26
50
127
59
57
125
45
85
7
3
9
5
9
7
5
28
12
11
9
68
9
12
8
0
0
4
0
22
0
16
16
0
0
0
28
2
96
19
36
8
8
6
17
13
17
22
21
22
13
0
13
15
34
75
10
49
40
37
25
40
54
13
2
18
8
21
115
19
102
25
7
13
9
6
2
14
20
27
5
5
7


26
50
127
59
57
125
45
85
7
3
9
5
9
7
5
28
12
11
9
68
9
12
8
0
0
4
0
22
0
16
16
0
0
0
28
2
96
19
36
8
8
6
17
13
17
22
21
22
13
0
13
15
34
75
10
49
40
37
25
40
54
13
2
18
8
21
115
19
102
25
7
13
9
6
2
14
20
27
5
5
7


26
50
127
59
57
125
45
85
7
3
9
5
9
7
5
28
12
11
9
68
9
12
8
0
0
4
0
22
0
16
16
0
0
0
28
2
96
19
36
8
8
6
17
13
17
22
21
22
13
0
13
15
34
75
10
49
40
37
25
40
54
13
2
18
8
21
115
19
102
25
7
13
9
6
2
14
20
27
5
5
7


26
50
127
59
57
125
45
85
7
3
9
5
9
7
5
28
12
11
9
68
9
12
8
0
0
4
0
22
0
16
16
0
0
0
28
2
96
19
36
8
8
6
17
13
17
22
21
22
13
0
13
15
34
75
10
49
40
37
25
40
54
13
2
18
8
21
115
19
102
25
7
13
9
6
2
14
20
27
5
5
7


73
160
313
84
152
74
165
170
35
93
39
76
47
62
87
85
352
60
150
92
306
280
96
0
0
53
0
142
72
132
116
10
9
105
208
20
1
83
99
99
104
195
289
128
94
164
112
202
109
335
98
14
16
166
138
115
85
109
214
427
225
130
17
107
37
99
9
64
63
8
36
2
4
9
0
5
0
1
1
2
6


73
160
313
84
152
74
165
170
35
93
39
76
47
62
87
85
352
60
150
92
306
280
96
0
0
53
0
142
72
132
116
10
9
105
208
20
1
83
99
99
104
195
289
128
94
164
112
202
109
335
98
14
16
166
138
115
85
109
214
427
225
130
17
107
37
99
9
64
63
8
36
2
4
9
0
5
0
1
1
2
6


73
160
313
84
152
74
165
170
35
93
39
76
47
62
87
85
352
60
150
92
306
280
96
0
0
53
0
142
72
132
116
10
9
105
208
20
1
83
99
99
104
195
289
128
94
164
112
202
109
335
98
14
16
166
138
115
85
109
214
427
225
130
17
107
37
99
9
64
63
8
36
2
4
9
0
5
0
1
1
2
6


73
160
313
84
152
74
165
170
35
93
39
76
47
62
87
85
352
60
150
92
306
280
96
0
0
53
0
142
72
132
116
10
9
105
208
20
1
83
99
99
104
195
289
128
94
164
112
202
109
335
98
14
16
166
138
115
85
109
214
427
225
130
17
107
37
99
9
64
63
8
36
2
4
9
0
5
0
1
1
2
6


0
3
3
0
1
0
0
0
0
0
1
0
0
2
0
0
2
0
1
0
5
0
0
0
0
0
0
1
0
0
1
0
0
0
0
0
0
0
0
0
0
0
0
0
0
1
0
0
0
0
0
0
0
0
1
0
0
0
0
1
2
0
1
0
0
0
0
0
0
0
0
0
0
0
0
0
0
0
0
0
0


0
3
3
0
1
0
0
0
0
0
1
0
0
1
0
0
2
0
1
0
5
0
0
0
0
0
0
1
0
0
1
0
0
0
0
0
0
0
0
0
0
0
0
0
0
1
0
0
0
0
0
0
0
0
0
0
0
0
0
1
1
0
1
0
0
0
0
0
0
0
0
0
0
0
0
0
0
0
0
0
0


0
3
3
0
1
0
0
0
0
0
1
0
0
1
0
0
2
0
1
0
5
0
0
0
0
0
0
1
0
0
1
0
0
0
0
0
0
0
0
0
0
0
0
0
0
1
0
0
0
0
0
0
0
0
0
0
0
0
0
1
1
0
1
0
0
0
0
0
0
0
0
0
0
0
0
0
0
0
0
0
0


0
3
3
0
1
0
0
0
0
0
1
0
0
1
0
0
2
0
1
0
5
0
0
0
0
0
0
1
0
0
1
0
0
0
0
0
0
0
0
0
0
0
0
0
0
1
0
0
0
0
0
0
0
0
0
0
0
0
0
1
1
0
1
0
0
0
0
0
0
0
0
0
0
0
0
0
0
0
0
0
0


0
3
3
0
1
0
0
0
0
0
1
0
0
1
0
0
2
0
1
0
5
0
0
0
0
0
0
1
0
0
1
0
0
0
0
0
0
0
0
0
0
0
0
0
0
1
0
0
0
0
0
0
0
0
0
0
0
0
0
1
1
0
1
0
0
0
0
0
0
0
0
0
0
0
0
0
0
0
0
0
0


0
0
0
0
0
0
0
0
0
0
0
0
0
1
0
0
0
0
0
0
0
0
0
0
0
0
0
0
0
0
0
0
0
0
0
0
0
0
0
0
0
0
0
0
0
0
0
0
0
0
0
0
0
0
1
0
0
0
0
0
1
0
0
0
0
0
0
0
0
0
0
0
0
0
0
0
0
0
0
0
0


0
0
0
0
0
0
0
0
0
0
0
0
0
1
0
0
0
0
0
0
0
0
0
0
0
0
0
0
0
0
0
0
0
0
0
0
0
0
0
0
0
0
0
0
0
0
0
0
0
0
0
0
0
0
1
0
0
0
0
0
1
0
0
0
0
0
0
0
0
0
0
0
0
0
0
0
0
0
0
0
0


0
0
0
0
0
0
0
0
0
0
0
0
0
1
0
0
0
0
0
0
0
0
0
0
0
0
0
0
0
0
0
0
0
0
0
0
0
0
0
0
0
0
0
0
0
0
0
0
0
0
0
0
0
0
1
0
0
0
0
0
1
0
0
0
0
0
0
0
0
0
0
0
0
0
0
0
0
0
0
0
0


0
0
0
0
0
0
0
0
0
0
0
0
0
1
0
0
0
0
0
0
0
0
0
0
0
0
0
0
0
0
0
0
0
0
0
0
0
0
0
0
0
0
0
0
0
0
0
0
0
0
0
0
0
0
1
0
0
0
0
0
1
0
0
0
0
0
0
0
0
0
0
0
0
0
0
0
0
0
0
0
0


0
0
0
0
0
0
0
0
0
0
0
0
0
0
0
0
0
0
0
0
0
0
0
0
0
0
0
0
0
0
0
0
0
0
0
0
0
0
0
0
0
0
0
0
0
0
0
0
0
0
0
0
0
0
0
0
0
0
0
0
0
0
0
0
0
0
0
0
0
0
0
0
0
2
0
0
0
0
0
0
0


0
0
0
0
0
0
0
0
0
0
0
0
0
0
0
0
0
0
0
0
0
0
0
0
0
0
0
0
0
0
0
0
0
0
0
0
0
0
0
0
0
0
0
0
0
0
0
0
0
0
0
0
0
0
0
0
0
0
0
0
0
0
0
0
0
0
0
0
0
0
0
0
0
2
0
0
0
0
0
0
0


0
0
0
0
0
0
0
0
0
0
0
0
0
0
0
0
0
0
0
0
0
0
0
0
0
0
0
0
0
0
0
0
0
0
0
0
0
0
0
0
0
0
0
0
0
0
0
0
0
0
0
0
0
0
0
0
0
0
0
0
0
0
0
0
0
0
0
0
0
0
0
0
0
2
0
0
0
0
0
0
0


0
0
0
0
0
0
0
0
0
0
0
0
0
0
0
0
0
0
0
0
0
0
0
0
0
0
0
0
0
0
0
0
0
0
0
0
0
0
0
0
0
0
0
0
0
0
0
0
0
0
0
0
0
0
0
0
0
0
0
0
0
0
0
0
0
0
0
0
0
0
0
0
0
2
0
0
0
0
0
0
0


0
0
0
0
0
0
0
0
0
0
0
0
0
0
0
0
0
0
0
0
0
0
0
0
0
0
0
0
0
0
0
0
0
0
0
0
0
0
0
0
0
0
0
0
0
0
0
0
0
0
0
0
0
0
0
0
0
0
0
0
0
0
0
0
0
0
0
0
0
0
0
0
0
2
0
0
0
0
0
0
0


9
51
34
6
5
10
6
4
3
12
169
18
89
28
65
44
39
29
72
17
380
156
98
0
0
671
0
233
0
83
110
45
28
108
125
36
0
67
90
157
147
101
136
188
98
110
317
231
85
145
12
26
2
23
11
24
18
22
9
52
17
24
2
14
2
9
0
11
0
0
2
0
0
0
0
0
1
0
0
0
0


9
51
34
6
5
10
6
4
3
12
169
18
89
28
65
44
39
29
72
17
380
156
98
0
0
671
0
233
0
83
110
45
28
108
125
36
0
67
90
157
147
101
136
188
98
110
317
231
85
145
12
26
2
23
11
24
18
22
9
52
17
24
2
14
2
9
0
11
0
0
2
0
0
0
0
0
1
0
0
0
0


9
51
34
6
5
10
6
4
3
12
169
18
89
28
65
44
39
29
72
17
380
156
98
0
0
671
0
233
0
83
110
45
28
108
125
36
0
67
90
157
147
101
136
188
98
110
317
231
85
145
12
26
2
23
11
24
18
22
9
52
17
24
2
14
2
9
0
11
0
0
2
0
0
0
0
0
1
0
0
0
0


6
5
16
1
3
1
5
4
3
8
166
13
85
22
20
0
8
7
47
1
164
37
15
0
0
5
0
5
0
13
18
0
0
96
106
1
0
0
1
6
21
29
61
80
40
55
2
5
21
0
7
3
0
9
1
0
3
4
0
19
7
5
0
13
0
3
0
6
0
0
0
0
0
0
0
0
0
0
0
0
0


6
5
16
1
3
1
5
4
3
8
166
13
85
22
20
0
8
7
47
1
164
37
15
0
0
5
0
5
0
13
18
0
0
96
106
1
0
0
1
6
21
29
61
80
40
55
2
5
21
0
7
3
0
9
1
0
3
4
0
19
7
5
0
13
0
3
0
6
0
0
0
0
0
0
0
0
0
0
0
0
0


3
46
18
5
2
9
1
0
0
4
3
5
4
6
45
44
31
22
25
16
216
119
83
0
0
666
0
228
0
70
92
45
28
12
19
35
0
67
89
151
126
72
75
108
58
55
315
226
64
145
5
23
2
14
10
24
15
18
9
33
10
19
2
1
2
6
0
5
0
0
2
0
0
0
0
0
1
0
0
0
0


3
46
18
5
2
9
1
0
0
4
3
5
4
6
45
44
31
22
25
16
216
119
83
0
0
666
0
228
0
70
92
45
28
12
19
35
0
67
89
151
126
72
75
108
58
55
315
226
64
145
5
23
2
14
10
24
15
18
9
33
10
19
2
1
2
6
0
5
0
0
2
0
0
0
0
0
1
0
0
0
0


600
1796
2830
1841
1178
1919
1087
1058
648
1007
994
1134
860
765
2046
1959
2747
1331
1679
3077
3046
3516
2033
346
272
2615
544
2582
518
1708
2236
2163
1072
3244
1332
1723
473
1321
1356
2946
2242
1427
1636
2297
2305
2017
2828
2796
2329
1291
873
1252
782
1311
909
1597
1629
1683
2174
3272
2157
914
1154
892
1094
1618
433
937
761
514
1137
2086
2083
2213
996
1593
1813
1666
1079
724
630


368
753
1183
1214
589
1040
607
643
331
662
396
468
322
360
900
830
1328
484
880
1396
1148
1483
751
346
188
543
199
546
185
824
1156
679
413
2272
895
706
92
324
447
650
524
529
720
1097
1314
1319
573
889
1043
634
250
194
264
446
354
411
403
719
903
964
855
284
514
341
644
751
263
346
500
277
470
716
596
1141
156
560
264
434
403
380
237


27
23
41
47
39
49
20
33
8
20
14
37
6
10
26
48
50
13
17
49
10
56
24
0
0
35
0
47
0
33
38
105
33
49
9
69
0
18
39
72
35
34
24
55
57
35
53
54
59
0
13
9
13
29
18
44
41
72
83
37
62
14
34
15
37
67
24
17
22
32
40
60
57
56
7
42
12
16
29
32
15


27
23
41
47
39
49
20
33
8
20
14
37
6
10
26
48
50
13
17
49
10
56
24
0
0
35
0
47
0
33
38
105
33
49
9
69
0
18
39
72
35
34
24
55
57
35
53
54
59
0
13
9
13
29
18
44
41
72
83
37
62
14
34
15
37
67
24
17
22
32
40
60
57
56
7
42
12
16
29
32
15


27
23
41
47
39
49
20
33
8
20
14
37
6
10
26
48
50
13
17
49
10
56
24
0
0
35
0
47
0
33
38
105
33
49
9
69
0
18
39
72
35
34
24
55
57
35
53
54
59
0
13
9
13
29
18
44
41
72
83
37
62
14
34
15
37
67
24
17
22
32
40
60
57
56
7
42
12
16
29
32
15


0
0
2
0
0
0
2
0
1
3
0
3
3
1
1
0
2
0
2
2
0
2
0
0
0
2
0
5
0
2
3
0
0
0
0
0
0
2
4
6
0
0
2
2
1
2
1
0
0
0
0
1
0
1
1
4
0
3
1
2
9
1
2
1
0
3
1
0
0
0
1
5
1
1
0
0
0
0
0
0
0


0
0
2
0
0
0
2
0
1
3
0
3
3
1
1
0
2
0
2
2
0
2
0
0
0
2
0
5
0
2
3
0
0
0
0
0
0
2
4
6
0
0
2
2
1
2
1
0
0
0
0
1
0
1
1
4
0
3
1
2
9
1
2
1
0
3
1
0
0
0
1
5
1
1
0
0
0
0
0
0
0


0
0
2
0
0
0
2
0
1
3
0
3
3
1
1
0
2
0
2
2
0
2
0
0
0
2
0
5
0
2
3
0
0
0
0
0
0
2
4
6
0
0
2
2
1
2
1
0
0
0
0
1
0
1
1
4
0
3
1
2
9
1
2
1
0
3
1
0
0
0
1
5
1
1
0
0
0
0
0
0
0


341
730
1138
1166
550
990
585
610
322
639
382
426
313
348
873
782
1276
471
861
1342
1138
1425
727
346
188
505
199
493
185
786
1115
574
380
2223
886
634
92
303
404
568
489
495
694
1040
1254
1282
517
835
981
634
237
184
251
415
334
363
361
642
817
925
781
269
478
325
606
679
237
329
476
245
429
651
538
1081
149
518
252
418
374
348
220


341
730
1138
1166
550
990
585
610
322
639
382
426
313
348
873
782
1276
471
861
1342
1138
1425
727
346
188
505
199
493
185
786
1115
574
380
2223
886
634
92
303
404
568
489
495
694
1040
1254
1282
517
835
981
634
237
184
251
415
334
363
361
642
817
925
781
269
478
325
606
679
237
329
476
245
429
651
538
1081
149
518
252
418
374
348
220


341
730
1138
1166
550
990
585
610
322
639
382
426
313
348
873
782
1276
471
861
1342
1138
1425
727
346
188
505
199
493
185
786
1115
574
380
2223
886
634
92
303
404
568
489
495
694
1040
1254
1282
517
835
981
634
237
184
251
415
334
363
361
642
817
925
781
269
478
325
606
679
237
329
476
245
429
651
538
1081
149
518
252
418
374
348
220


0
0
2
1
0
1
0
0
0
0
0
2
0
1
0
0
0
0
0
3
0
0
0
0
0
1
0
1
0
3
0
0
0
0
0
3
0
1
0
4
0
0
0
0
2
0
2
0
3
0
0
0
0
1
1
0
1
2
2
0
3
0
0
0
1
2
1
0
2
0
0
0
0
3
0
0
0
0
0
0
2


0
0
2
1
0
1
0
0
0
0
0
2
0
1
0
0
0
0
0
3
0
0
0
0
0
1
0
1
0
3
0
0
0
0
0
3
0
1
0
4
0
0
0
0
2
0
2
0
3
0
0
0
0
1
1
0
1
2
2
0
3
0
0
0
1
2
1
0
2
0
0
0
0
3
0
0
0
0
0
0
2


0
0
2
1
0
1
0
0
0
0
0
2
0
1
0
0
0
0
0
3
0
0
0
0
0
1
0
1
0
3
0
0
0
0
0
3
0
1
0
4
0
0
0
0
2
0
2
0
3
0
0
0
0
1
1
0
1
2
2
0
3
0
0
0
1
2
1
0
2
0
0
0
0
3
0
0
0
0
0
0
2


232
1043
1645
624
589
876
476
414
317
342
598
666
538
404
1145
1128
1416
847
798
1678
1892
2030
1281
0
84
2072
345
2036
333
884
1079
1484
659
972
437
1017
381
997
907
2295
1718
898
915
1200
990
695
2255
1904
1286
657
623
1058
518
865
554
1184
1224
963
1270
2303
1302
630
637
551
450
864
170
591
261
237
667
1369
1487
1072
837
1032
1549
1232
676
344
391


91
546
1001
200
244
240
229
180
137
146
293
312
226
246
566
458
765
439
388
758
1026
782
557
0
48
867
215
857
138
400
461
370
154
252
183
254
198
439
411
817
635
369
407
504
433
343
1008
857
593
439
259
246
138
407
204
437
413
357
551
1213
700
274
98
338
178
388
27
269
86
53
205
100
102
95
41
103
93
98
29
75
20


91
546
1001
200
244
240
229
180
137
146
293
312
226
246
566
458
765
439
388
758
1026
782
557
0
48
867
215
857
138
400
461
370
154
252
183
254
198
439
411
817
635
369
407
504
433
343
1008
857
593
439
259
246
138
407
204
437
413
357
551
1213
700
274
98
338
178
388
27
269
86
53
205
100
102
95
41
103
93
98
29
75
20


91
546
1001
200
244
240
229
180
137
146
293
312
226
246
566
458
765
439
388
758
1026
782
557
0
48
867
215
857
138
400
461
370
154
252
183
254
198
439
411
817
635
369
407
504
433
343
1008
857
593
439
259
246
138
407
204
437
413
357
551
1213
700
274
98
338
178
388
27
269
86
53
205
100
102
95
41
103
93
98
29
75
20


13
49
76
131
56
261
36
48
27
27
21
33
12
13
40
70
45
50
18
347
91
78
61
0
0
133
0
59
0
69
165
701
23
398
58
354
91
68
37
134
82
63
42
93
90
88
84
95
86
0
67
318
143
37
76
160
217
99
122
98
95
52
336
12
110
113
78
33
72
47
145
1121
1215
788
661
805
1290
992
526
198
290


11
43
60
104
51
226
34
42
17
26
16
26
11
6
34
63
43
46
14
324
83
65
53
0
0
127
0
55
0
64
138
540
22
363
48
314
0
50
30
104
67
53
42
88
76
86
72
92
80
0
59
295
119
33
54
149
178
82
94
89
82
43
316
9
97
105
70
24
57
46
118
1037
1127
736
597
750
1160
912
475
180
245


11
43
60
104
51
226
34
42
17
26
16
26
11
6
34
63
43
46
14
324
83
65
53
0
0
127
0
55
0
64
138
540
22
363
48
314
0
50
30
104
67
53
42
88
76
86
72
92
80
0
59
295
119
33
54
149
178
82
94
89
82
43
316
9
97
105
70
24
57
46
118
1037
1127
736
597
750
1160
912
475
180
245


0
0
0
2
1
4
0
4
7
0
3
4
1
6
6
4
1
4
2
3
1
11
6
0
0
2
0
0
0
2
12
12
0
5
1
4
3
0
0
19
8
5
0
1
5
0
5
0
1
0
3
4
3
3
16
3
13
9
12
7
3
3
15
2
3
2
0
7
3
0
11
6
9
9
5
3
6
6
5
1
4


0
0
0
2
1
4
0
4
7
0
3
4
1
6
6
4
1
4
2
3
1
11
6
0
0
2
0
0
0
2
12
12
0
5
1
4
3
0
0
19
8
5
0
1
5
0
5
0
1
0
3
4
3
3
16
3
13
9
12
7
3
3
15
2
3
2
0
7
3
0
11
6
9
9
5
3
6
6
5
1
4


2
6
16
25
4
31
2
2
3
1
2
3
0
1
0
3
1
0
2
20
7
2
2
0
0
4
0
4
0
3
15
149
1
30
9
36
88
18
7
11
7
5
0
4
9
2
7
3
5
0
5
19
21
1
6
8
26
8
16
2
10
6
5
1
10
6
8
2
12
1
16
78
79
43
59
52
124
74
46
17
41


2
6
16
25
4
31
2
2
3
1
2
3
0
1
0
3
1
0
2
20
7
2
2
0
0
4
0
4
0
3
15
149
1
30
9
36
88
18
7
11
7
5
0
4
9
2
7
3
5
0
5
19
21
1
6
8
26
8
16
2
10
6
5
1
10
6
8
2
12
1
16
78
79
43
59
52
124
74
46
17
41


19
16
27
31
21
31
27
17
12
10
24
14
10
8
26
59
45
10
26
28
73
74
40
0
0
27
0
127
0
33
46
71
106
97
29
61
1
16
44
158
97
59
13
89
87
48
105
105
38
119
26
10
13
19
20
45
24
41
28
34
26
16
10
19
14
38
1
26
6
8
23
6
9
13
4
0
1
2
8
9
6


18
16
27
31
21
31
27
17
12
10
24
14
10
8
25
59
44
10
26
28
72
74
40
0
0
26
0
127
0
33
46
71
106
97
29
61
1
16
44
158
97
59
13
89
87
48
105
105
38
119
26
10
13
19
20
44
24
41
27
34
26
16
10
19
14
38
1
26
6
8
23
5
9
13
4
0
0
2
8
9
6


18
16
27
31
21
31
27
17
12
10
24
14
10
8
25
59
44
10
26
28
72
74
40
0
0
26
0
127
0
33
46
71
106
97
29
61
1
16
44
158
97
59
13
89
87
48
105
105
38
119
26
10
13
19
20
44
24
41
27
34
26
16
10
19
14
38
1
26
6
8
23
5
9
13
4
0
0
2
8
9
6


1
0
0
0
0
0
0
0
0
0
0
0
0
0
0
0
1
0
0
0
1
0
0
0
0
0
0
0
0
0
0
0
0
0
0
0
0
0
0
0
0
0
0
0
0
0
0
0
0
0
0
0
0
0
0
1
0
0
1
0
0
0
0
0
0
0
0
0
0
0
0
1
0
0
0
0
1
0
0
0
0


1
0
0
0
0
0
0
0
0
0
0
0
0
0
0
0
1
0
0
0
1
0
0
0
0
0
0
0
0
0
0
0
0
0
0
0
0
0
0
0
0
0
0
0
0
0
0
0
0
0
0
0
0
0
0
1
0
0
1
0
0
0
0
0
0
0
0
0
0
0
0
1
0
0
0
0
1
0
0
0
0


0
0
0
0
0
0
0
0
0
0
0
0
0
0
1
0
0
0
0
0
0
0
0
0
0
1
0
0
0
0
0
0
0
0
0
0
0
0
0
0
0
0
0
0
0
0
0
0
0
0
0
0
0
0
0
0
0
0
0
0
0
0
0
0
0
0
0
0
0
0
0
0
0
0
0
0
0
0
0
0
0


0
0
0
0
0
0
0
0
0
0
0
0
0
0
1
0
0
0
0
0
0
0
0
0
0
1
0
0
0
0
0
0
0
0
0
0
0
0
0
0
0
0
0
0
0
0
0
0
0
0
0
0
0
0
0
0
0
0
0
0
0
0
0
0
0
0
0
0
0
0
0
0
0
0
0
0
0
0
0
0
0


86
270
370
185
174
228
101
98
112
116
207
248
240
100
434
423
464
248
314
304
533
939
552
0
36
897
127
874
157
274
259
306
309
131
155
133
91
371
286
893
729
330
364
414
304
144
861
734
420
98
211
286
153
315
203
442
437
337
481
765
375
230
55
132
89
246
5
233
49
95
219
18
42
36
6
9
6
8
26
35
14


51
215
305
142
109
156
56
53
45
76
83
108
135
53
265
289
327
139
207
184
417
697
404
0
36
644
127
661
157
175
187
222
177
39
117
80
88
288
219
645
501
229
220
265
200
90
656
557
232
95
120
216
95
212
120
317
272
215
266
577
232
150
30
83
44
127
1
153
22
75
109
11
37
26
4
7
3
8
19
25
11


51
215
305
142
109
156
56
53
45
76
83
108
135
53
265
289
327
139
207
184
417
697
404
0
36
644
127
661
157
175
187
222
177
39
117
80
88
288
219
645
501
229
220
265
200
90
656
557
232
95
120
216
95
212
120
317
272
215
266
577
232
150
30
83
44
127
1
153
22
75
109
11
37
26
4
7
3
8
19
25
11


29
46
49
8
55
58
39
45
60
31
117
127
94
43
158
115
122
89
92
96
88
202
135
0
0
223
0
181
0
91
59
0
132
1
35
44
0
53
4
196
184
85
132
127
90
42
174
149
163
0
81
60
57
92
72
106
144
89
193
161
119
71
20
41
7
96
4
68
25
0
86
5
3
5
1
0
2
0
6
8
3


29
46
49
8
55
58
39
45
60
31
117
127
94
43
158
115
122
89
92
96
88
202
135
0
0
223
0
181
0
91
59
0
132
1
35
44
0
53
4
196
184
85
132
127
90
42
174
149
163
0
81
60
57
92
72
106
144
89
193
161
119
71
20
41
7
96
4
68
25
0
86
5
3
5
1
0
2
0
6
8
3


6
9
16
35
10
14
6
0
7
9
7
13
11
4
11
19
15
20
15
24
28
40
13
0
0
30
0
32
0
8
13
84
0
91
3
9
3
30
63
52
44
16
12
22
14
12
31
28
25
3
10
10
1
11
11
19
21
33
22
27
24
9
5
8
38
23
0
12
2
20
24
2
2
5
1
2
1
0
1
2
0


6
9
16
35
10
14
6
0
7
9
7
13
11
4
11
19
15
20
15
24
28
40
13
0
0
30
0
32
0
8
13
84
0
91
3
9
3
30
63
52
44
16
12
22
14
12
31
28
25
3
10
10
1
11
11
19
21
33
22
27
24
9
5
8
38
23
0
12
2
20
24
2
2
5
1
2
1
0
1
2
0


23
162
171
77
94
116
83
71
29
43
53
59
50
37
79
118
97
100
52
241
169
157
71
0
0
148
3
119
38
108
148
36
67
94
12
215
0
103
129
293
175
77
89
100
76
72
197
113
149
1
60
198
71
87
51
100
133
129
88
193
106
58
138
50
59
79
59
30
48
34
75
124
119
140
125
115
159
132
87
27
61


23
162
171
77
94
116
83
71
29
43
53
59
50
37
79
118
97
100
52
241
169
157
71
0
0
148
3
119
38
108
148
36
67
94
12
215
0
103
129
293
175
77
89
100
76
72
197
113
149
1
60
198
71
87
51
100
133
129
88
193
106
58
138
50
59
79
59
30
48
34
75
124
119
140
125
115
159
132
87
27
61


23
162
171
77
94
116
83
71
29
43
53
59
50
37
79
118
97
100
52
241
169
157
71
0
0
148
3
119
38
108
148
36
67
94
12
215
0
103
129
293
175
77
89
100
76
72
197
113
149
1
60
198
71
87
51
100
133
129
88
193
106
58
138
50
59
79
59
30
48
34
75
124
119
140
125
115
159
132
87
27
61


0
0
2
3
0
3
4
1
0
3
0
0
0
1
1
1
3
0
1
3
6
3
1
0
0
0
0
0
0
0
1
0
0
0
0
0
0
0
2
1
0
0
1
0
1
3
0
3
0
0
0
0
0
0
1
2
2
1
1
5
0
0
3
0
0
3
0
0
0
0
0
1
0
0
3
1
0
0
0
0
2


0
0
2
3
0
3
4
1
0
3
0
0
0
1
1
1
3
0
1
3
6
3
1
0
0
0
0
0
0
0
1
0
0
0
0
0
0
0
2
1
0
0
1
0
1
3
0
3
0
0
0
0
0
0
1
2
2
1
1
5
0
0
3
0
0
3
0
0
0
0
0
1
0
0
3
1
0
0
0
0
2


0
0
2
3
0
3
4
1
0
3
0
0
0
1
1
1
3
0
1
3
6
3
1
0
0
0
0
0
0
0
1
0
0
0
0
0
0
0
2
1
0
0
1
0
1
3
0
3
0
0
0
0
0
0
1
2
2
1
1
5
0
0
3
0
0
3
0
0
0
0
0
1
0
0
3
1
0
0
0
0
2


0
0
2
3
0
3
4
1
0
3
0
0
0
1
1
1
3
0
1
3
6
3
1
0
0
0
0
0
0
0
1
0
0
0
0
0
0
0
2
1
0
0
1
0
1
3
0
3
0
0
0
0
0
0
1
2
2
1
1
5
0
0
3
0
0
3
0
0
0
0
0
1
0
0
3
1
0
0
0
0
2


25
40
55
56
30
61
24
34
10
17
20
17
17
20
23
43
29
32
31
29
40
51
21
0
0
19
0
25
3
43
26
8
0
13
22
28
0
50
40
50
48
21
27
31
34
19
30
53
41
0
25
21
32
24
30
34
39
52
47
60
24
19
33
31
14
16
30
26
45
15
17
7
15
13
11
9
22
18
12
1
17


25
40
55
56
30
61
24
34
10
17
20
17
17
20
23
43
29
32
31
29
40
51
21
0
0
19
0
25
3
43
26
8
0
13
22
28
0
50
40
50
48
21
27
31
34
19
30
53
41
0
25
21
32
24
30
34
39
52
47
60
24
19
33
31
14
16
30
26
45
15
17
7
15
13
11
9
22
18
12
1
17


25
40
55
56
30
61
24
34
10
17
20
17
17
20
23
43
29
32
31
29
40
51
21
0
0
19
0
25
3
43
26
8
0
13
22
28
0
50
40
50
48
21
27
31
34
19
30
53
41
0
25
21
32
24
30
34
39
52
47
60
24
19
33
31
14
16
30
26
45
15
17
7
15
13
11
9
22
18
12
1
17


25
40
55
56
30
61
24
34
10
17
20
17
17
20
23
43
29
32
31
29
40
51
21
0
0
19
0
25
3
43
26
8
0
13
22
28
0
50
40
50
48
21
27
31
34
19
30
53
41
0
25
21
32
24
30
34
39
52
47
60
24
19
33
31
14
16
30
26
45
15
17
7
15
13
11
9
22
18
12
1
17


25
40
55
56
30
61
24
34
10
17
20
17
17
20
23
43
29
32
31
29
40
51
21
0
0
19
0
25
3
43
26
8
0
13
22
28
0
50
40
50
48
21
27
31
34
19
30
53
41
0
25
21
32
24
30
34
39
52
47
60
24
19
33
31
14
16
30
26
45
15
17
7
15
13
11
9
22
18
12
1
17


126
77
92
204
77
92
81
125
53
62
38
115
43
100
67
68
91
58
75
131
140
108
99
18
0
103
0
157
0
179
194
0
251
45
72
233
119
259
272
107
113
108
59
147
190
184
153
247
106
30
60
94
110
65
58
86
77
124
55
87
104
44
90
34
123
106
122
43
100
102
90
168
377
163
189
183
180
217
221
67
113


17
13
33
22
9
14
20
13
7
7
10
31
13
13
11
21
30
31
19
26
60
40
31
18
0
25
0
84
0
41
28
0
1
0
19
14
0
36
67
52
46
32
24
35
50
60
71
84
28
30
14
27
19
19
8
16
16
19
18
24
22
15
27
15
14
26
20
16
18
24
16
11
27
7
13
26
15
30
13
9
2


17
13
33
22
9
14
20
13
7
7
10
31
13
13
11
21
30
31
19
26
60
40
31
18
0
25
0
84
0
41
28
0
1
0
19
14
0
36
67
52
46
32
24
35
50
60
71
84
28
30
14
27
19
19
8
16
16
19
18
24
22
15
27
15
14
26
20
16
18
24
16
11
27
7
13
26
15
30
13
9
2


17
13
33
22
9
14
20
13
7
7
10
31
13
13
11
21
30
31
19
26
60
40
31
18
0
25
0
84
0
41
28
0
1
0
19
14
0
36
67
52
46
32
24
35
50
60
71
84
28
30
14
27
19
19
8
16
16
19
18
24
22
15
27
15
14
26
20
16
18
24
16
11
27
7
13
26
15
30
13
9
2


17
13
33
22
9
14
20
13
7
7
10
31
13
13
11
21
30
31
19
26
60
40
31
18
0
25
0
84
0
41
28
0
1
0
19
14
0
36
67
52
46
32
24
35
50
60
71
84
28
30
14
27
19
19
8
16
16
19
18
24
22
15
27
15
14
26
20
16
18
24
16
11
27
7
13
26
15
30
13
9
2


17
13
33
22
9
14
20
13
7
7
10
31
13
13
11
21
30
31
19
26
60
40
31
18
0
25
0
84
0
41
28
0
1
0
19
14
0
36
67
52
46
32
24
35
50
60
71
84
28
30
14
27
19
19
8
16
16
19
18
24
22
15
27
15
14
26
20
16
18
24
16
11
27
7
13
26
15
30
13
9
2


3
1
1
16
0
2
0
2
0
4
0
4
0
5
0
2
1
4
0
4
4
3
0
0
0
6
0
6
0
6
4
0
250
37
0
48
0
3
8
6
4
1
1
14
25
7
4
4
4
0
0
6
3
0
1
5
0
4
2
2
2
0
5
0
7
2
2
1
0
0
4
33
39
11
34
15
22
20
22
4
7


0
1
1
6
0
0
0
0
0
0
0
2
0
1
0
2
1
0
0
1
3
3
0
0
0
5
0
6
0
3
2
0
0
0
0
9
0
0
5
4
2
1
1
6
3
4
2
4
4
0
0
2
1
0
1
2
0
4
2
2
2
0
0
0
3
2
0
0
0
0
3
10
19
3
5
4
0
8
4
1
1


0
1
1
6
0
0
0
0
0
0
0
2
0
1
0
2
1
0
0
1
3
3
0
0
0
5
0
6
0
3
2
0
0
0
0
9
0
0
5
4
2
1
1
6
3
4
2
4
4
0
0
2
1
0
1
2
0
4
2
2
2
0
0
0
3
2
0
0
0
0
3
10
19
3
5
4
0
8
4
1
1


0
0
1
6
0
0
0
0
0
0
0
1
0
1
0
2
1
0
0
1
1
2
0
0
0
5
0
6
0
2
2
0
0
0
0
9
0
0
4
4
1
1
1
5
3
3
2
3
3
0
0
1
1
0
1
2
0
4
1
1
2
0
0
0
3
1
0
0
0
0
3
9
19
3
4
3
0
7
3
1
1


0
0
1
6
0
0
0
0
0
0
0
1
0
1
0
2
1
0
0
1
1
2
0
0
0
5
0
6
0
2
2
0
0
0
0
9
0
0
4
4
1
1
1
5
3
3
2
3
3
0
0
1
1
0
1
2
0
4
1
1
2
0
0
0
3
1
0
0
0
0
3
9
19
3
4
3
0
7
3
1
1


0
0
0
0
0
0
0
0
0
0
0
0
0
0
0
0
0
0
0
0
0
0
0
0
0
0
0
0
0
0
0
0
0
0
0
0
0
0
0
0
0
0
0
0
0
0
0
0
0
0
0
0
0
0
0
0
0
0
0
1
0
0
0
0
0
0
0
0
0
0
0
0
0
0
0
1
0
0
0
0
0


0
0
0
0
0
0
0
0
0
0
0
0
0
0
0
0
0
0
0
0
0
0
0
0
0
0
0
0
0
0
0
0
0
0
0
0
0
0
0
0
0
0
0
0
0
0
0
0
0
0
0
0
0
0
0
0
0
0
0
1
0
0
0
0
0
0
0
0
0
0
0
0
0
0
0
1
0
0
0
0
0


0
1
0
0
0
0
0
0
0
0
0
1
0
0
0
0
0
0
0
0
2
1
0
0
0
0
0
0
0
1
0
0
0
0
0
0
0
0
1
0
1
0
0
1
0
1
0
1
1
0
0
1
0
0
0
0
0
0
1
0
0
0
0
0
0
1
0
0
0
0
0
1
0
0
1
0
0
1
1
0
0


0
1
0
0
0
0
0
0
0
0
0
1
0
0
0
0
0
0
0
0
2
1
0
0
0
0
0
0
0
1
0
0
0
0
0
0
0
0
1
0
1
0
0
1
0
1
0
1
1
0
0
1
0
0
0
0
0
0
1
0
0
0
0
0
0
1
0
0
0
0
0
1
0
0
1
0
0
1
1
0
0


3
0
0
10
0
2
0
2
0
4
0
2
0
4
0
0
0
4
0
3
1
0
0
0
0
1
0
0
0
3
2
0
250
37
0
39
0
3
3
2
2
0
0
8
22
3
2
0
0
0
0
4
2
0
0
3
0
0
0
0
0
0
5
0
4
0
2
1
0
0
1
23
20
8
29
11
22
12
18
3
6


3
0
0
10
0
2
0
2
0
4
0
2
0
4
0
0
0
4
0
3
1
0
0
0
0
1
0
0
0
3
2
0
250
37
0
39
0
3
3
2
2
0
0
8
22
3
2
0
0
0
0
4
2
0
0
3
0
0
0
0
0
0
5
0
4
0
2
1
0
0
1
23
20
8
29
11
22
12
18
3
6


3
0
0
10
0
2
0
2
0
4
0
2
0
4
0
0
0
4
0
3
1
0
0
0
0
1
0
0
0
3
2
0
250
37
0
39
0
3
3
2
2
0
0
8
22
3
2
0
0
0
0
4
2
0
0
3
0
0
0
0
0
0
5
0
4
0
2
1
0
0
1
23
20
8
29
11
22
12
18
3
6


3
0
0
10
0
2
0
2
0
4
0
2
0
4
0
0
0
4
0
3
1
0
0
0
0
1
0
0
0
3
2
0
250
37
0
39
0
3
3
2
2
0
0
8
22
3
2
0
0
0
0
4
2
0
0
3
0
0
0
0
0
0
5
0
4
0
2
1
0
0
1
23
20
8
29
11
22
12
18
3
6


53
17
20
74
28
35
30
67
12
12
2
9
16
17
16
17
17
9
5
44
20
33
19
0
0
19
0
17
0
111
57
0
0
4
31
42
0
63
65
23
19
20
22
63
66
78
16
44
18
0
16
34
40
20
12
32
17
51
22
20
29
8
12
3
37
27
55
18
47
65
19
39
135
29
58
67
49
76
44
25
44


25
4
11
36
6
17
8
18
10
9
0
6
0
9
8
11
7
4
4
23
6
11
4
0
0
7
0
9
0
41
41
0
0
4
12
31
0
19
34
12
10
11
8
37
40
26
10
19
5
0
10
13
20
12
9
19
9
27
10
15
17
4
6
1
20
17
20
10
15
28
6
30
104
17
41
38
32
41
30
18
26


0
0
1
0
0
0
0
0
0
0
0
0
0
0
0
0
0
0
0
0
0
0
0
0
0
0
0
0
0
0
1
0
0
0
0
0
0
1
1
0
0
1
0
3
0
1
0
0
0
0
0
0
0
0
0
0
0
0
1
0
0
0
0
0
0
0
0
0
0
0
0
0
0
0
0
0
0
0
0
0
0


0
0
1
0
0
0
0
0
0
0
0
0
0
0
0
0
0
0
0
0
0
0
0
0
0
0
0
0
0
0
1
0
0
0
0
0
0
1
1
0
0
1
0
3
0
1
0
0
0
0
0
0
0
0
0
0
0
0
1
0
0
0
0
0
0
0
0
0
0
0
0
0
0
0
0
0
0
0
0
0
0


0
0
1
0
0
0
0
0
0
0
0
0
0
0
0
0
0
0
0
0
0
0
0
0
0
0
0
0
0
0
1
0
0
0
0
0
0
1
1
0
0
1
0
3
0
1
0
0
0
0
0
0
0
0
0
0
0
0
1
0
0
0
0
0
0
0
0
0
0
0
0
0
0
0
0
0
0
0
0
0
0


23
4
9
35
6
16
8
18
9
9
0
6
0
8
8
11
7
2
4
23
4
10
4
0
0
6
0
9
0
37
38
0
0
4
8
27
0
18
31
11
8
8
8
30
30
25
7
11
4
0
9
13
17
12
9
17
9
27
8
12
12
4
6
1
14
15
20
7
12
25
3
28
100
16
35
35
24
34
30
17
26


21
4
9
35
5
15
7
17
9
8
0
6
0
8
8
10
7
2
4
23
4
9
4
0
0
6
0
9
0
37
38
0
0
4
8
26
0
17
31
11
8
8
8
30
29
20
7
11
4
0
9
9
15
12
9
16
9
26
8
12
12
4
3
1
14
14
18
7
12
25
3
27
93
16
35
31
23
26
27
16
25


21
4
9
35
5
15
7
17
9
8
0
6
0
8
8
10
7
2
4
23
4
9
4
0
0
6
0
9
0
37
38
0
0
4
8
26
0
17
31
11
8
8
8
30
29
20
7
11
4
0
9
9
15
12
9
16
9
26
8
12
12
4
3
1
14
14
18
7
12
25
3
27
93
16
35
31
23
26
27
16
25


0
0
0
0
0
0
0
0
0
0
0
0
0
0
0
0
0
0
0
0
0
0
0
0
0
0
0
0
0
0
0
0
0
0
0
0
0
0
0
0
0
0
0
0
0
0
0
0
0
0
0
1
0
0
0
0
0
0
0
0
0
0
1
0
0
0
0
0
0
0
0
0
0
0
0
0
0
0
3
0
0


0
0
0
0
0
0
0
0
0
0
0
0
0
0
0
0
0
0
0
0
0
0
0
0
0
0
0
0
0
0
0
0
0
0
0
0
0
0
0
0
0
0
0
0
0
0
0
0
0
0
0
1
0
0
0
0
0
0
0
0
0
0
1
0
0
0
0
0
0
0
0
0
0
0
0
0
0
0
3
0
0


2
0
0
0
1
1
1
1
0
1
0
0
0
0
0
1
0
0
0
0
0
1
0
0
0
0
0
0
0
0
0
0
0
0
0
1
0
1
0
0
0
0
0
0
1
5
0
0
0
0
0
3
2
0
0
1
0
1
0
0
0
0
2
0
0
1
2
0
0
0
0
1
7
0
0
4
1
8
0
1
1


2
0
0
0
1
1
1
1
0
1
0
0
0
0
0
1
0
0
0
0
0
1
0
0
0
0
0
0
0
0
0
0
0
0
0
1
0
1
0
0
0
0
0
0
1
5
0
0
0
0
0
3
2
0
0
1
0
1
0
0
0
0
2
0
0
1
2
0
0
0
0
1
7
0
0
4
1
8
0
1
1


2
0
1
1
0
1
0
0
1
0
0
0
0
1
0
0
0
2
0
0
2
1
0
0
0
1
0
0
0
4
2
0
0
0
4
4
0
0
2
1
2
2
0
4
10
0
3
8
1
0
1
0
3
0
0
2
0
0
1
3
5
0
0
0
6
2
0
3
3
3
3
2
4
1
6
3
8
7
0
1
0


2
0
1
1
0
1
0
0
1
0
0
0
0
1
0
0
0
2
0
0
2
1
0
0
0
1
0
0
0
4
2
0
0
0
4
4
0
0
2
1
2
2
0
4
10
0
3
8
1
0
1
0
3
0
0
2
0
0
1
3
5
0
0
0
6
2
0
3
3
3
3
2
4
1
6
3
8
7
0
1
0


2
0
1
1
0
1
0
0
1
0
0
0
0
1
0
0
0
2
0
0
2
1
0
0
0
1
0
0
0
4
2
0
0
0
4
4
0
0
2
1
2
2
0
4
10
0
3
8
1
0
1
0
3
0
0
2
0
0
1
3
5
0
0
0
6
2
0
3
3
3
3
2
4
1
6
3
8
7
0
1
0


28
13
9
38
20
18
22
48
2
3
2
3
16
8
8
6
10
5
1
21
14
22
15
0
0
12
0
8
0
70
16
0
0
0
19
11
0
44
31
11
9
9
14
26
26
52
5
25
13
0
6
21
20
8
3
13
8
24
12
5
12
4
6
2
17
10
35
8
31
37
13
9
31
12
17
29
17
35
14
7
18


28
13
9
38
20
18
22
48
2
3
2
3
16
8
8
6
10
5
1
21
14
22
15
0
0
12
0
8
0
70
16
0
0
0
19
11
0
44
31
11
9
9
14
26
26
52
5
25
13
0
6
21
20
8
3
13
8
24
12
5
12
4
6
2
17
10
35
8
31
37
13
9
31
12
17
29
17
35
14
7
18


28
13
9
38
20
18
22
48
2
3
2
3
16
8
8
6
10
5
1
21
14
22
15
0
0
12
0
8
0
70
16
0
0
0
19
11
0
44
31
11
9
9
14
26
26
52
5
25
13
0
6
21
20
8
3
13
8
24
12
5
12
4
6
2
17
10
35
8
31
37
13
9
31
12
17
29
17
35
14
7
18


28
13
9
38
20
18
22
48
2
3
2
3
16
8
8
6
10
5
1
21
14
22
15
0
0
12
0
8
0
70
16
0
0
0
19
11
0
44
31
11
9
9
14
26
26
52
5
25
13
0
6
21
20
8
3
13
8
24
12
5
12
4
6
2
17
10
35
8
31
37
13
9
31
12
17
29
17
35
14
7
18


0
0
0
0
2
0
0
1
0
0
0
0
0
0
0
0
0
0
0
0
0
0
0
0
0
0
0
0
0
0
0
0
0
0
0
0
0
0
0
0
0
0
0
0
0
0
1
0
0
0
0
0
0
0
0
0
0
0
0
0
0
0
0
0
0
0
0
0
1
0
0
0
0
0
0
0
0
0
0
0
0


0
0
0
0
2
0
0
1
0
0
0
0
0
0
0
0
0
0
0
0
0
0
0
0
0
0
0
0
0
0
0
0
0
0
0
0
0
0
0
0
0
0
0
0
0
0
1
0
0
0
0
0
0
0
0
0
0
0
0
0
0
0
0
0
0
0
0
0
1
0
0
0
0
0
0
0
0
0
0
0
0


0
0
0
0
2
0
0
1
0
0
0
0
0
0
0
0
0
0
0
0
0
0
0
0
0
0
0
0
0
0
0
0
0
0
0
0
0
0
0
0
0
0
0
0
0
0
1
0
0
0
0
0
0
0
0
0
0
0
0
0
0
0
0
0
0
0
0
0
1
0
0
0
0
0
0
0
0
0
0
0
0


0
0
0
0
2
0
0
1
0
0
0
0
0
0
0
0
0
0
0
0
0
0
0
0
0
0
0
0
0
0
0
0
0
0
0
0
0
0
0
0
0
0
0
0
0
0
1
0
0
0
0
0
0
0
0
0
0
0
0
0
0
0
0
0
0
0
0
0
1
0
0
0
0
0
0
0
0
0
0
0
0


52
46
36
85
40
41
31
43
34
39
26
69
14
63
40
26
40
12
50
52
51
29
47
0
0
45
0
49
0
15
93
0
0
0
22
128
119
151
122
24
43
53
10
33
45
35
53
109
54
0
30
24
45
24
37
31
43
45
13
36
50
21
45
16
64
50
43
8
33
12
48
80
173
114
81
72
94
87
142
29
58


0
1
0
0
0
0
0
0
0
0
0
0
0
0
1
0
0
0
1
0
0
0
0
0
0
1
0
0
0
0
0
0
0
0
0
0
0
0
0
0
0
0
0
3
0
0
0
0
0
0
0
0
0
0
0
0
0
0
0
0
0
0
0
0
0
0
0
0
0
0
1
0
0
0
0
0
0
0
2
0
0


0
1
0
0
0
0
0
0
0
0
0
0
0
0
1
0
0
0
1
0
0
0
0
0
0
1
0
0
0
0
0
0
0
0
0
0
0
0
0
0
0
0
0
3
0
0
0
0
0
0
0
0
0
0
0
0
0
0
0
0
0
0
0
0
0
0
0
0
0
0
1
0
0
0
0
0
0
0
2
0
0


0
1
0
0
0
0
0
0
0
0
0
0
0
0
1
0
0
0
1
0
0
0
0
0
0
1
0
0
0
0
0
0
0
0
0
0
0
0
0
0
0
0
0
3
0
0
0
0
0
0
0
0
0
0
0
0
0
0
0
0
0
0
0
0
0
0
0
0
0
0
1
0
0
0
0
0
0
0
2
0
0


0
1
0
0
0
0
0
0
0
0
0
0
0
0
1
0
0
0
1
0
0
0
0
0
0
1
0
0
0
0
0
0
0
0
0
0
0
0
0
0
0
0
0
3
0
0
0
0
0
0
0
0
0
0
0
0
0
0
0
0
0
0
0
0
0
0
0
0
0
0
1
0
0
0
0
0
0
0
2
0
0


52
45
36
85
40
41
31
43
34
39
26
69
14
63
39
26
40
12
49
52
51
29
47
0
0
44
0
49
0
15
93
0
0
0
22
128
119
151
122
24
43
53
10
30
45
35
53
109
54
0
30
24
45
24
37
31
43
45
13
36
50
21
45
16
64
50
43
8
33
12
47
80
173
114
81
72
94
87
140
29
58


0
0
12
0
2
0
0
3
3
1
0
0
6
5
2
0
0
0
1
2
1
0
5
0
0
0
0
2
0
0
2
0
0
0
0
0
0
0
1
0
1
2
0
0
0
0
1
0
0
0
0
0
0
0
1
0
1
0
1
0
1
0
0
3
1
1
0
0
0
0
0
0
0
0
0
0
0
0
0
0
0


0
0
12
0
2
0
0
3
3
1
0
0
6
5
2
0
0
0
1
2
1
0
5
0
0
0
0
2
0
0
2
0
0
0
0
0
0
0
1
0
1
2
0
0
0
0
1
0
0
0
0
0
0
0
1
0
1
0
1
0
1
0
0
3
1
1
0
0
0
0
0
0
0
0
0
0
0
0
0
0
0


0
0
12
0
2
0
0
3
3
1
0
0
6
5
2
0
0
0
1
2
1
0
5
0
0
0
0
2
0
0
2
0
0
0
0
0
0
0
1
0
1
2
0
0
0
0
1
0
0
0
0
0
0
0
1
0
1
0
1
0
1
0
0
3
1
1
0
0
0
0
0
0
0
0
0
0
0
0
0
0
0


52
38
24
85
36
39
28
31
30
38
25
68
7
58
37
26
40
11
48
48
49
29
42
0
0
44
0
47
0
15
91
0
0
0
22
128
119
149
121
24
42
51
10
30
45
35
52
109
49
0
28
24
45
24
32
31
40
45
12
29
43
21
45
12
63
47
43
7
33
12
47
80
173
114
81
72
94
87
140
29
58


2
5
0
0
0
0
2
12
13
0
0
0
0
6
10
1
3
0
3
1
0
1
0
0
0
0
0
0
0
0
4
0
0
0
0
0
0
1
0
0
1
4
3
0
0
0
1
0
0
0
0
1
0
0
2
8
0
4
1
1
1
3
0
0
4
4
2
1
6
0
0
0
1
0
1
1
2
0
1
1
0


2
5
0
0
0
0
2
12
13
0
0
0
0
6
10
1
3
0
3
1
0
1
0
0
0
0
0
0
0
0
4
0
0
0
0
0
0
1
0
0
1
4
3
0
0
0
1
0
0
0
0
1
0
0
2
8
0
4
1
1
1
3
0
0
4
4
2
1
6
0
0
0
1
0
1
1
2
0
1
1
0


33
32
24
74
35
33
20
13
9
37
23
43
6
26
18
17
24
8
35
34
43
13
13
0
0
26
0
41
0
11
73
0
0
0
16
107
0
61
98
21
23
30
7
25
40
30
39
53
42
0
24
20
33
21
27
19
28
35
6
27
40
18
38
11
54
34
36
4
15
7
39
43
88
99
44
58
53
59
96
27
47


33
32
24
74
35
33
20
13
9
37
23
43
6
26
18
17
24
8
35
34
43
13
13
0
0
26
0
41
0
11
73
0
0
0
16
107
0
61
98
21
23
30
7
25
40
30
39
53
42
0
24
20
33
21
27
19
28
35
6
27
40
18
38
11
54
34
36
4
15
7
39
43
88
99
44
58
53
59
96
27
47


17
1
0
11
1
6
6
6
8
1
2
25
1
26
9
8
13
3
10
13
6
15
29
0
0
18
0
6
0
4
14
0
0
0
6
21
119
87
23
3
18
17
0
5
5
5
12
56
7
0
4
3
12
3
3
4
12
6
5
1
2
0
7
1
5
9
5
2
12
5
8
37
84
15
36
13
39
28
43
1
11


17
1
0
11
1
6
6
6
8
1
2
25
1
26
9
8
13
3
10
13
6
15
29
0
0
18
0
6
0
4
14
0
0
0
6
21
119
87
23
3
18
17
0
5
5
5
12
56
7
0
4
3
12
3
3
4
12
6
5
1
2
0
7
1
5
9
5
2
12
5
8
37
84
15
36
13
39
28
43
1
11


0
7
0
0
2
2
3
9
1
0
1
1
1
0
0
0
0
1
0
2
1
0
0
0
0
0
0
0
0
0
0
0
0
0
0
0
0
2
0
0
0
0
0
0
0
0
0
0
5
0
2
0
0
0
4
0
2
0
0
7
6
0
0
1
0
2
0
1
0
0
0
0
0
0
0
0
0
0
0
0
0


0
7
0
0
2
2
3
9
1
0
1
1
1
0
0
0
0
1
0
2
1
0
0
0
0
0
0
0
0
0
0
0
0
0
0
0
0
2
0
0
0
0
0
0
0
0
0
0
5
0
2
0
0
0
4
0
2
0
0
7
6
0
0
1
0
2
0
1
0
0
0
0
0
0
0
0
0
0
0
0
0


0
7
0
0
2
2
3
9
1
0
1
1
1
0
0
0
0
1
0
2
1
0
0
0
0
0
0
0
0
0
0
0
0
0
0
0
0
2
0
0
0
0
0
0
0
0
0
0
5
0
2
0
0
0
4
0
2
0
0
7
6
0
0
1
0
2
0
1
0
0
0
0
0
0
0
0
0
0
0
0
0


0
0
0
2
0
0
0
0
0
0
0
1
0
2
0
2
1
0
0
2
2
1
1
0
0
4
0
0
0
2
12
0
0
3
0
1
0
6
7
2
1
0
1
2
3
2
6
2
1
0
0
2
3
0
0
1
1
4
0
0
1
0
1
0
1
0
0
0
0
1
2
2
0
1
0
2
0
3
0
0
2


0
0
0
2
0
0
0
0
0
0
0
1
0
2
0
2
1
0
0
2
2
1
1
0
0
4
0
0
0
2
12
0
0
3
0
1
0
6
7
2
1
0
1
2
3
2
6
2
1
0
0
2
3
0
0
1
1
4
0
0
1
0
1
0
1
0
0
0
0
1
2
2
0
1
0
2
0
3
0
0
2


0
0
0
2
0
0
0
0
0
0
0
1
0
2
0
2
1
0
0
2
2
1
1
0
0
4
0
0
0
2
12
0
0
3
0
1
0
6
7
2
1
0
1
2
3
2
6
2
1
0
0
2
3
0
0
1
1
4
0
0
1
0
1
0
1
0
0
0
0
1
2
2
0
1
0
2
0
3
0
0
2


0
0
0
2
0
0
0
0
0
0
0
1
0
2
0
2
1
0
0
2
2
1
1
0
0
4
0
0
0
2
12
0
0
3
0
1
0
6
7
2
1
0
1
2
3
2
6
2
1
0
0
2
3
0
0
1
1
4
0
0
1
0
1
0
1
0
0
0
0
1
2
2
0
1
0
2
0
3
0
0
2


0
0
0
2
0
0
0
0
0
0
0
1
0
2
0
2
1
0
0
2
2
1
1
0
0
4
0
0
0
2
12
0
0
3
0
1
0
6
7
2
1
0
1
2
3
2
6
2
1
0
0
2
3
0
0
1
1
4
0
0
1
0
1
0
1
0
0
0
0
1
2
2
0
1
0
2
0
3
0
0
2


0
0
0
0
0
0
0
0
0
0
0
0
0
0
0
0
0
0
0
0
0
0
0
0
0
0
0
0
0
0
0
0
0
0
0
0
0
0
0
0
0
0
0
0
0
1
2
0
0
0
0
0
0
0
0
0
0
1
0
0
0
0
0
0
0
0
0
0
0
0
0
0
2
0
0
0
0
0
0
0
0


0
0
0
0
0
0
0
0
0
0
0
0
0
0
0
0
0
0
0
0
0
0
0
0
0
0
0
0
0
0
0
0
0
0
0
0
0
0
0
0
0
0
0
0
0
1
2
0
0
0
0
0
0
0
0
0
0
1
0
0
0
0
0
0
0
0
0
0
0
0
0
0
2
0
0
0
0
0
0
0
0


0
0
0
0
0
0
0
0
0
0
0
0
0
0
0
0
0
0
0
0
0
0
0
0
0
0
0
0
0
0
0
0
0
0
0
0
0
0
0
0
0
0
0
0
0
1
2
0
0
0
0
0
0
0
0
0
0
1
0
0
0
0
0
0
0
0
0
0
0
0
0
0
2
0
0
0
0
0
0
0
0


0
0
0
0
0
0
0
0
0
0
0
0
0
0
0
0
0
0
0
0
0
0
0
0
0
0
0
0
0
0
0
0
0
0
0
0
0
0
0
0
0
0
0
0
0
1
2
0
0
0
0
0
0
0
0
0
0
1
0
0
0
0
0
0
0
0
0
0
0
0
0
0
2
0
0
0
0
0
0
0
0


0
0
0
0
0
0
0
0
0
0
0
0
0
0
0
0
0
0
0
0
0
0
0
0
0
0
0
0
0
0
0
0
0
0
0
0
0
0
0
0
0
0
0
0
0
1
2
0
0
0
0
0
0
0
0
0
0
1
0
0
0
0
0
0
0
0
0
0
0
0
0
0
2
0
0
0
0
0
0
0
0


0
0
0
0
0
0
0
0
0
0
0
0
0
0
0
0
0
0
0
1
2
0
0
0
0
0
0
0
0
0
0
0
0
0
0
0
0
0
0
0
0
0
0
0
0
0
0
0
0
0
0
0
0
0
0
0
0
0
0
3
0
0
0
0
0
0
0
0
1
0
0
0
0
0
0
0
0
0
0
0
0


0
0
0
0
0
0
0
0
0
0
0
0
0
0
0
0
0
0
0
1
0
0
0
0
0
0
0
0
0
0
0
0
0
0
0
0
0
0
0
0
0
0
0
0
0
0
0
0
0
0
0
0
0
0
0
0
0
0
0
3
0
0
0
0
0
0
0
0
1
0
0
0
0
0
0
0
0
0
0
0
0


0
0
0
0
0
0
0
0
0
0
0
0
0
0
0
0
0
0
0
1
0
0
0
0
0
0
0
0
0
0
0
0
0
0
0
0
0
0
0
0
0
0
0
0
0
0
0
0
0
0
0
0
0
0
0
0
0
0
0
3
0
0
0
0
0
0
0
0
1
0
0
0
0
0
0
0
0
0
0
0
0


0
0
0
0
0
0
0
0
0
0
0
0
0
0
0
0
0
0
0
1
0
0
0
0
0
0
0
0
0
0
0
0
0
0
0
0
0
0
0
0
0
0
0
0
0
0
0
0
0
0
0
0
0
0
0
0
0
0
0
3
0
0
0
0
0
0
0
0
1
0
0
0
0
0
0
0
0
0
0
0
0


0
0
0
0
0
0
0
0
0
0
0
0
0
0
0
0
0
0
0
1
0
0
0
0
0
0
0
0
0
0
0
0
0
0
0
0
0
0
0
0
0
0
0
0
0
0
0
0
0
0
0
0
0
0
0
0
0
0
0
3
0
0
0
0
0
0
0
0
1
0
0
0
0
0
0
0
0
0
0
0
0


0
0
0
0
0
0
0
0
0
0
0
0
0
0
0
0
0
0
0
0
2
0
0
0
0
0
0
0
0
0
0
0
0
0
0
0
0
0
0
0
0
0
0
0
0
0
0
0
0
0
0
0
0
0
0
0
0
0
0
0
0
0
0
0
0
0
0
0
0
0
0
0
0
0
0
0
0
0
0
0
0


0
0
0
0
0
0
0
0
0
0
0
0
0
0
0
0
0
0
0
0
2
0
0
0
0
0
0
0
0
0
0
0
0
0
0
0
0
0
0
0
0
0
0
0
0
0
0
0
0
0
0
0
0
0
0
0
0
0
0
0
0
0
0
0
0
0
0
0
0
0
0
0
0
0
0
0
0
0
0
0
0


0
0
0
0
0
0
0
0
0
0
0
0
0
0
0
0
0
0
0
0
2
0
0
0
0
0
0
0
0
0
0
0
0
0
0
0
0
0
0
0
0
0
0
0
0
0
0
0
0
0
0
0
0
0
0
0
0
0
0
0
0
0
0
0
0
0
0
0
0
0
0
0
0
0
0
0
0
0
0
0
0


0
0
0
0
0
0
0
0
0
0
0
0
0
0
0
0
0
0
0
0
2
0
0
0
0
0
0
0
0
0
0
0
0
0
0
0
0
0
0
0
0
0
0
0
0
0
0
0
0
0
0
0
0
0
0
0
0
0
0
0
0
0
0
0
0
0
0
0
0
0
0
0
0
0
0
0
0
0
0
0
0


1
0
2
5
0
0
0
0
0
0
0
1
0
0
0
0
2
2
1
2
1
2
1
0
0
4
0
1
0
4
0
0
0
1
0
0
0
0
3
0
0
2
1
0
1
1
1
4
1
0
0
1
0
2
0
1
0
0
0
2
0
0
0
0
0
1
2
0
1
0
1
3
1
1
3
1
0
1
0
0
0


1
0
2
5
0
0
0
0
0
0
0
1
0
0
0
0
2
2
1
2
1
2
1
0
0
4
0
1
0
4
0
0
0
1
0
0
0
0
3
0
0
2
1
0
1
1
1
4
1
0
0
1
0
2
0
1
0
0
0
2
0
0
0
0
0
1
2
0
1
0
1
3
1
1
3
1
0
1
0
0
0


1
0
2
5
0
0
0
0
0
0
0
1
0
0
0
0
2
2
1
2
1
2
1
0
0
4
0
1
0
4
0
0
0
1
0
0
0
0
3
0
0
2
1
0
1
1
1
4
1
0
0
1
0
2
0
1
0
0
0
2
0
0
0
0
0
1
2
0
1
0
1
3
1
1
3
1
0
1
0
0
0


1
0
2
5
0
0
0
0
0
0
0
1
0
0
0
0
2
2
1
2
1
2
1
0
0
4
0
1
0
4
0
0
0
1
0
0
0
0
3
0
0
2
1
0
1
1
1
4
1
0
0
1
0
2
0
1
0
0
0
2
0
0
0
0
0
1
2
0
1
0
1
3
1
1
3
1
0
1
0
0
0


1
0
2
5
0
0
0
0
0
0
0
1
0
0
0
0
2
2
1
2
1
2
1
0
0
4
0
1
0
4
0
0
0
1
0
0
0
0
3
0
0
2
1
0
1
1
1
4
1
0
0
1
0
2
0
1
0
0
0
2
0
0
0
0
0
1
2
0
1
0
1
3
1
1
3
1
0
1
0
0
0


0
0
0
2
0
0
3
0
1
0
1
2
0
0
0
0
0
0
0
1
0
1
0
0
0
0
0
0
0
0
1
0
0
0
0
1
0
0
3
2
0
1
0
2
0
0
0
0
0
0
0
0
3
3
1
0
3
5
0
2
2
3
1
0
0
0
0
0
3
0
1
0
4
1
1
0
0
4
3
0
1


0
0
0
2
0
0
3
0
1
0
1
2
0
0
0
0
0
0
0
1
0
1
0
0
0
0
0
0
0
0
1
0
0
0
0
1
0
0
3
2
0
1
0
2
0
0
0
0
0
0
0
0
3
3
1
0
3
5
0
2
2
3
1
0
0
0
0
0
3
0
1
0
4
1
1
0
0
4
3
0
1


0
0
0
2
0
0
3
0
1
0
1
2
0
0
0
0
0
0
0
1
0
1
0
0
0
0
0
0
0
0
1
0
0
0
0
1
0
0
3
2
0
1
0
2
0
0
0
0
0
0
0
0
3
3
1
0
3
5
0
2
2
3
1
0
0
0
0
0
3
0
1
0
4
1
1
0
0
4
3
0
1


0
0
0
2
0
0
3
0
1
0
1
2
0
0
0
0
0
0
0
1
0
1
0
0
0
0
0
0
0
0
1
0
0
0
0
1
0
0
3
2
0
1
0
2
0
0
0
0
0
0
0
0
3
3
1
0
3
5
0
2
2
3
1
0
0
0
0
0
3
0
1
0
4
1
1
0
0
4
3
0
1


0
0
0
2
0
0
3
0
1
0
1
2
0
0
0
0
0
0
0
1
0
1
0
0
0
0
0
0
0
0
1
0
0
0
0
1
0
0
3
2
0
1
0
2
0
0
0
0
0
0
0
0
3
3
1
0
3
5
0
2
2
3
1
0
0
0
0
0
3
0
1
0
4
1
1
0
0
4
3
0
1


0
0
0
2
0
0
3
0
1
0
1
2
0
0
0
0
0
0
0
1
0
1
0
0
0
0
0
0
0
0
1
0
0
0
0
1
0
0
3
2
0
1
0
2
0
0
0
0
0
0
0
0
3
3
1
0
3
5
0
2
2
3
1
0
0
0
0
0
3
0
1
0
4
1
1
0
0
4
3
0
1


10
12
4
7
6
3
15
3
5
5
12
15
10
14
7
13
15
11
9
11
4
7
9
0
0
6
0
14
0
7
2
0
0
0
10
1
0
9
9
1
6
10
3
3
3
3
7
0
14
0
4
7
2
6
7
9
10
6
5
9
15
6
1
5
3
17
3
9
4
3
10
1
0
0
0
0
0
1
0
1
0


0
0
0
0
0
0
0
0
0
0
0
0
0
1
0
0
0
0
0
0
0
0
0
0
0
0
0
0
0
0
0
0
0
0
0
0
0
0
0
0
0
0
0
0
0
0
0
0
0
0
0
0
0
0
0
0
0
0
0
0
0
0
0
0
0
0
0
0
0
0
0
0
0
0
0
0
0
0
0
0
0


0
0
0
0
0
0
0
0
0
0
0
0
0
1
0
0
0
0
0
0
0
0
0
0
0
0
0
0
0
0
0
0
0
0
0
0
0
0
0
0
0
0
0
0
0
0
0
0
0
0
0
0
0
0
0
0
0
0
0
0
0
0
0
0
0
0
0
0
0
0
0
0
0
0
0
0
0
0
0
0
0


0
0
0
0
0
0
0
0
0
0
0
0
0
1
0
0
0
0
0
0
0
0
0
0
0
0
0
0
0
0
0
0
0
0
0
0
0
0
0
0
0
0
0
0
0
0
0
0
0
0
0
0
0
0
0
0
0
0
0
0
0
0
0
0
0
0
0
0
0
0
0
0
0
0
0
0
0
0
0
0
0


0
0
0
0
0
0
0
0
0
0
0
0
0
1
0
0
0
0
0
0
0
0
0
0
0
0
0
0
0
0
0
0
0
0
0
0
0
0
0
0
0
0
0
0
0
0
0
0
0
0
0
0
0
0
0
0
0
0
0
0
0
0
0
0
0
0
0
0
0
0
0
0
0
0
0
0
0
0
0
0
0


0
0
0
0
0
0
0
0
0
0
0
0
0
1
0
0
0
0
0
0
0
0
0
0
0
0
0
0
0
0
0
0
0
0
0
0
0
0
0
0
0
0
0
0
0
0
0
0
0
0
0
0
0
0
0
0
0
0
0
0
0
0
0
0
0
0
0
0
0
0
0
0
0
0
0
0
0
0
0
0
0


10
12
4
7
6
3
15
3
5
5
12
15
10
13
7
13
15
11
9
11
4
7
9
0
0
6
0
14
0
7
2
0
0
0
10
1
0
9
9
1
6
10
3
3
3
3
7
0
14
0
4
7
2
6
7
9
10
6
5
9
15
6
1
5
3
17
3
9
4
3
10
1
0
0
0
0
0
1
0
1
0


10
12
4
7
6
3
15
3
5
5
12
15
10
13
7
13
15
11
9
11
4
7
9
0
0
6
0
14
0
7
2
0
0
0
10
1
0
9
9
1
6
10
3
3
3
3
7
0
14
0
4
7
2
6
7
9
10
6
5
9
15
6
1
5
3
17
3
9
4
3
10
1
0
0
0
0
0
1
0
1
0


10
12
4
7
6
3
15
3
5
5
12
15
10
13
7
13
15
11
9
11
4
7
9
0
0
6
0
14
0
7
2
0
0
0
10
1
0
9
9
1
6
10
3
3
3
3
7
0
14
0
4
7
2
6
7
9
10
6
5
9
15
6
1
5
3
17
3
9
4
3
10
1
0
0
0
0
0
1
0
1
0


10
12
4
7
6
3
15
3
5
5
12
15
10
13
7
13
15
11
9
11
4
7
9
0
0
6
0
14
0
7
2
0
0
0
10
1
0
9
9
1
6
10
3
3
3
3
7
0
14
0
4
7
2
6
7
9
10
6
5
9
15
6
1
5
3
17
3
9
4
3
10
1
0
0
0
0
0
1
0
1
0


10
12
4
7
6
3
15
3
5
5
12
15
10
13
7
13
15
11
9
11
4
7
9
0
0
6
0
14
0
7
2
0
0
0
10
1
0
9
9
1
6
10
3
3
3
3
7
0
14
0
4
7
2
6
7
9
10
6
5
9
15
6
1
5
3
17
3
9
4
3
10
1
0
0
0
0
0
1
0
1
0


2914
3884
2866
1907
3946
2384
3596
1798
3045
2493
3055
2721
3704
3664
3191
2650
2762
3270
3399
2477
3275
2883
3205
1112
567
2038
617
1034
1516
1239
2669
1198
1836
80
784
1497
1227
1632
2657
2208
1400
2093
991
1138
1054
1324
1363
574
2420
905
3350
1967
2375
2129
4240
3334
2495
2534
2762
1803
3965
4423
3761
2076
2073
2521
631
2195
1205
803
2671
1588
1479
3219
802
683
462
698
2434
1389
624


2
4
3
11
1
4
2
0
0
2
4
0
0
8
0
5
0
0
4
2
22
0
0
0
0
0
0
1
0
19
6
46
32
0
0
3
0
0
2
0
0
1
0
1
3
1
3
0
2
0
3
7
3
1
0
7
0
0
0
2
2
2
2
0
0
1
1
0
0
0
4
10
8
1
18
17
1
31
5
2
1


2
4
3
11
1
4
2
0
0
2
4
0
0
8
0
5
0
0
4
2
22
0
0
0
0
0
0
1
0
19
6
46
32
0
0
3
0
0
2
0
0
1
0
1
3
1
3
0
2
0
3
7
3
1
0
7
0
0
0
2
2
2
2
0
0
1
1
0
0
0
4
10
8
1
18
17
1
31
5
2
1


2
4
3
11
1
4
2
0
0
2
4
0
0
8
0
5
0
0
4
2
22
0
0
0
0
0
0
1
0
19
6
46
32
0
0
3
0
0
2
0
0
1
0
1
3
1
3
0
2
0
3
7
3
1
0
7
0
0
0
2
2
2
2
0
0
1
1
0
0
0
4
10
8
1
18
17
1
31
5
2
1


2
4
3
11
1
4
2
0
0
2
4
0
0
8
0
5
0
0
4
2
22
0
0
0
0
0
0
1
0
19
6
46
32
0
0
3
0
0
2
0
0
1
0
1
3
1
3
0
2
0
3
7
3
1
0
7
0
0
0
2
2
2
2
0
0
1
1
0
0
0
4
10
8
1
18
17
1
31
5
2
1


2
4
3
11
1
4
2
0
0
2
4
0
0
8
0
5
0
0
4
2
22
0
0
0
0
0
0
1
0
19
6
46
32
0
0
3
0
0
2
0
0
1
0
1
3
1
3
0
2
0
3
7
3
1
0
7
0
0
0
2
2
2
2
0
0
1
1
0
0
0
4
10
8
1
18
17
1
31
5
2
1


0
0
0
0
0
0
0
2
0
1
0
0
0
0
1
0
0
0
1
1
0
0
0
0
0
0
0
0
0
0
0
0
0
0
0
0
0
0
0
0
2
1
0
0
0
0
0
0
0
0
0
0
0
0
0
2
0
0
0
9
2
0
1
0
0
0
0
0
0
0
0
0
0
0
0
0
0
0
0
0
0


0
0
0
0
0
0
0
2
0
1
0
0
0
0
1
0
0
0
1
1
0
0
0
0
0
0
0
0
0
0
0
0
0
0
0
0
0
0
0
0
2
1
0
0
0
0
0
0
0
0
0
0
0
0
0
2
0
0
0
9
2
0
1
0
0
0
0
0
0
0
0
0
0
0
0
0
0
0
0
0
0


0
0
0
0
0
0
0
2
0
1
0
0
0
0
1
0
0
0
1
1
0
0
0
0
0
0
0
0
0
0
0
0
0
0
0
0
0
0
0
0
2
1
0
0
0
0
0
0
0
0
0
0
0
0
0
2
0
0
0
9
2
0
1
0
0
0
0
0
0
0
0
0
0
0
0
0
0
0
0
0
0


0
0
0
0
0
0
0
2
0
1
0
0
0
0
1
0
0
0
1
1
0
0
0
0
0
0
0
0
0
0
0
0
0
0
0
0
0
0
0
0
2
1
0
0
0
0
0
0
0
0
0
0
0
0
0
2
0
0
0
9
2
0
1
0
0
0
0
0
0
0
0
0
0
0
0
0
0
0
0
0
0


0
0
0
0
0
0
0
2
0
1
0
0
0
0
1
0
0
0
1
1
0
0
0
0
0
0
0
0
0
0
0
0
0
0
0
0
0
0
0
0
2
1
0
0
0
0
0
0
0
0
0
0
0
0
0
2
0
0
0
9
2
0
1
0
0
0
0
0
0
0
0
0
0
0
0
0
0
0
0
0
0


1
60
13
3
1
2
2
12
7
10
3
1
4
4
3
99
4
6
3
0
2
4
6
91
0
2
211
2
663
3
2
0
0
1
13
2
66
6
0
3
1
6
1
6
2
7
4
9
3
221
2
5
4
0
5
2
4
59
196
3
3
1
0
4
2
8
2
1
2
15
8
2
5
98
6
4
0
38
8
1
2


1
60
13
3
1
2
2
12
7
10
3
1
4
4
3
99
4
6
3
0
2
4
6
91
0
2
211
2
663
3
2
0
0
1
13
2
66
6
0
3
1
6
1
6
2
7
4
9
3
221
2
5
4
0
5
2
4
59
196
3
3
1
0
4
2
8
2
1
2
15
8
2
5
98
6
4
0
38
8
1
2


1
0
0
0
0
0
0
0
2
4
0
0
0
0
0
0
0
0
0
0
0
0
5
1
0
0
0
0
0
0
0
0
0
0
0
0
0
0
0
0
1
0
0
0
0
4
0
1
0
0
0
4
4
0
1
1
2
22
37
0
0
0
0
1
1
0
0
0
0
0
2
0
2
16
1
0
0
6
2
1
0


1
0
0
0
0
0
0
0
2
4
0
0
0
0
0
0
0
0
0
0
0
0
5
1
0
0
0
0
0
0
0
0
0
0
0
0
0
0
0
0
1
0
0
0
0
4
0
1
0
0
0
4
4
0
1
1
2
22
37
0
0
0
0
1
1
0
0
0
0
0
2
0
2
16
1
0
0
6
2
1
0


1
0
0
0
0
0
0
0
2
4
0
0
0
0
0
0
0
0
0
0
0
0
5
1
0
0
0
0
0
0
0
0
0
0
0
0
0
0
0
0
1
0
0
0
0
4
0
1
0
0
0
4
4
0
1
1
2
22
37
0
0
0
0
1
1
0
0
0
0
0
2
0
2
16
1
0
0
6
2
1
0


0
0
4
0
0
0
0
0
0
0
0
0
0
0
0
35
0
3
0
0
0
0
0
0
0
0
59
0
0
0
0
0
0
0
0
0
0
0
0
0
0
0
0
0
0
0
0
0
0
0
0
0
0
0
0
0
0
0
0
0
0
0
0
0
0
0
0
0
0
0
0
0
0
0
0
0
0
0
0
0
0


0
0
3
0
0
0
0
0
0
0
0
0
0
0
0
6
0
3
0
0
0
0
0
0
0
0
59
0
0
0
0
0
0
0
0
0
0
0
0
0
0
0
0
0
0
0
0
0
0
0
0
0
0
0
0
0
0
0
0
0
0
0
0
0
0
0
0
0
0
0
0
0
0
0
0
0
0
0
0
0
0


0
0
3
0
0
0
0
0
0
0
0
0
0
0
0
2
0
3
0
0
0
0
0
0
0
0
0
0
0
0
0
0
0
0
0
0
0
0
0
0
0
0
0
0
0
0
0
0
0
0
0
0
0
0
0
0
0
0
0
0
0
0
0
0
0
0
0
0
0
0
0
0
0
0
0
0
0
0
0
0
0


0
0
0
0
0
0
0
0
0
0
0
0
0
0
0
0
0
0
0
0
0
0
0
0
0
0
58
0
0
0
0
0
0
0
0
0
0
0
0
0
0
0
0
0
0
0
0
0
0
0
0
0
0
0
0
0
0
0
0
0
0
0
0
0
0
0
0
0
0
0
0
0
0
0
0
0
0
0
0
0
0


0
0
0
0
0
0
0
0
0
0
0
0
0
0
0
4
0
0
0
0
0
0
0
0
0
0
1
0
0
0
0
0
0
0
0
0
0
0
0
0
0
0
0
0
0
0
0
0
0
0
0
0
0
0
0
0
0
0
0
0
0
0
0
0
0
0
0
0
0
0
0
0
0
0
0
0
0
0
0
0
0


0
0
1
0
0
0
0
0
0
0
0
0
0
0
0
29
0
0
0
0
0
0
0
0
0
0
0
0
0
0
0
0
0
0
0
0
0
0
0
0
0
0
0
0
0
0
0
0
0
0
0
0
0
0
0
0
0
0
0
0
0
0
0
0
0
0
0
0
0
0
0
0
0
0
0
0
0
0
0
0
0


0
0
1
0
0
0
0
0
0
0
0
0
0
0
0
29
0
0
0
0
0
0
0
0
0
0
0
0
0
0
0
0
0
0
0
0
0
0
0
0
0
0
0
0
0
0
0
0
0
0
0
0
0
0
0
0
0
0
0
0
0
0
0
0
0
0
0
0
0
0
0
0
0
0
0
0
0
0
0
0
0


0
0
0
0
0
0
0
0
0
0
0
0
1
0
0
0
1
0
0
0
0
1
0
90
0
0
0
0
58
0
1
0
0
0
0
0
1
0
0
0
0
2
0
4
0
0
0
2
0
0
0
0
0
0
1
0
0
0
0
0
0
0
0
0
0
0
0
0
0
9
0
0
1
0
0
0
0
0
0
0
0


0
0
0
0
0
0
0
0
0
0
0
0
1
0
0
0
1
0
0
0
0
1
0
90
0
0
0
0
58
0
1
0
0
0
0
0
1
0
0
0
0
2
0
4
0
0
0
2
0
0
0
0
0
0
1
0
0
0
0
0
0
0
0
0
0
0
0
0
0
9
0
0
1
0
0
0
0
0
0
0
0


0
0
0
0
0
0
0
0
0
0
0
0
0
0
0
0
1
0
0
0
0
1
0
90
0
0
0
0
58
0
0
0
0
0
0
0
1
0
0
0
0
1
0
4
0
0
0
2
0
0
0
0
0
0
1
0
0
0
0
0
0
0
0
0
0
0
0
0
0
0
0
0
1
0
0
0
0
0
0
0
0


0
0
0
0
0
0
0
0
0
0
0
0
1
0
0
0
0
0
0
0
0
0
0
0
0
0
0
0
0
0
1
0
0
0
0
0
0
0
0
0
0
1
0
0
0
0
0
0
0
0
0
0
0
0
0
0
0
0
0
0
0
0
0
0
0
0
0
0
0
9
0
0
0
0
0
0
0
0
0
0
0


0
1
4
0
0
0
0
1
0
5
1
1
0
1
1
33
0
1
0
0
0
2
0
0
0
1
0
2
0
3
0
0
0
0
11
1
64
1
0
2
0
2
0
0
2
0
1
0
2
220
1
0
0
0
1
0
0
19
107
1
1
0
0
1
0
4
2
0
0
0
2
1
1
49
1
2
0
17
6
0
2


0
0
1
0
0
0
0
0
0
4
0
0
0
0
0
5
0
0
0
0
0
1
0
0
0
0
0
0
0
0
0
0
0
0
0
0
0
0
0
0
0
0
0
0
0
0
0
0
0
0
0
0
0
0
1
0
0
13
88
0
0
0
0
0
0
2
0
0
0
0
2
1
1
0
0
0
0
6
3
0
0


0
0
1
0
0
0
0
0
0
4
0
0
0
0
0
5
0
0
0
0
0
1
0
0
0
0
0
0
0
0
0
0
0
0
0
0
0
0
0
0
0
0
0
0
0
0
0
0
0
0
0
0
0
0
1
0
0
13
88
0
0
0
0
0
0
2
0
0
0
0
2
1
1
0
0
0
0
6
3
0
0


0
0
0
0
0
0
0
0
0
0
0
0
0
0
0
26
0
0
0
0
0
0
0
0
0
0
0
0
0
0
0
0
0
0
0
0
0
0
0
0
0
0
0
0
0
0
0
0
0
0
0
0
0
0
0
0
0
0
12
0
0
0
0
0
0
0
0
0
0
0
0
0
0
0
0
0
0
1
1
0
0


0
0
0
0
0
0
0
0
0
0
0
0
0
0
0
6
0
0
0
0
0
0
0
0
0
0
0
0
0
0
0
0
0
0
0
0
0
0
0
0
0
0
0
0
0
0
0
0
0
0
0
0
0
0
0
0
0
0
0
0
0
0
0
0
0
0
0
0
0
0
0
0
0
0
0
0
0
0
0
0
0


0
0
0
0
0
0
0
0
0
0
0
0
0
0
0
0
0
0
0
0
0
0
0
0
0
0
0
0
0
0
0
0
0
0
0
0
0
0
0
0
0
0
0
0
0
0
0
0
0
0
0
0
0
0
0
0
0
0
0
0
0
0
0
0
0
0
0
0
0
0
0
0
0
0
0
0
0
1
0
0
0


0
0
0
0
0
0
0
0
0
0
0
0
0
0
0
20
0
0
0
0
0
0
0
0
0
0
0
0
0
0
0
0
0
0
0
0
0
0
0
0
0
0
0
0
0
0
0
0
0
0
0
0
0
0
0
0
0
0
12
0
0
0
0
0
0
0
0
0
0
0
0
0
0
0
0
0
0
0
1
0
0


0
0
2
0
0
0
0
0
0
1
0
0
0
0
0
1
0
0
0
0
0
0
0
0
0
0
0
0
0
0
0
0
0
0
0
0
0
0
0
0
0
0
0
0
0
0
0
0
1
0
0
0
0
0
0
0
0
5
5
0
0
0
0
0
0
0
0
0
0
0
0
0
0
49
0
0
0
0
0
0
1


0
0
2
0
0
0
0
0
0
1
0
0
0
0
0
1
0
0
0
0
0
0
0
0
0
0
0
0
0
0
0
0
0
0
0
0
0
0
0
0
0
0
0
0
0
0
0
0
1
0
0
0
0
0
0
0
0
5
5
0
0
0
0
0
0
0
0
0
0
0
0
0
0
49
0
0
0
0
0
0
1


0
0
0
0
0
0
0
0
0
0
0
0
0
0
0
0
0
0
0
0
0
0
0
0
0
0
0
0
0
0
0
0
0
0
0
0
0
0
0
0
0
0
0
0
1
0
0
0
0
0
0
0
0
0
0
0
0
0
0
0
0
0
0
0
0
0
0
0
0
0
0
0
0
0
0
0
0
0
0
0
0


0
0
0
0
0
0
0
0
0
0
0
0
0
0
0
0
0
0
0
0
0
0
0
0
0
0
0
0
0
0
0
0
0
0
0
0
0
0
0
0
0
0
0
0
1
0
0
0
0
0
0
0
0
0
0
0
0
0
0
0
0
0
0
0
0
0
0
0
0
0
0
0
0
0
0
0
0
0
0
0
0


0
1
0
0
0
0
0
1
0
0
1
1
0
1
1
0
0
1
0
0
0
1
0
0
0
1
0
2
0
3
0
0
0
0
11
1
64
1
0
2
0
1
0
0
1
0
1
0
1
220
1
0
0
0
0
0
0
0
0
1
1
0
0
1
0
1
2
0
0
0
0
0
0
0
1
1
0
0
2
0
1


0
0
0
0
0
0
0
1
0
0
1
0
0
1
1
0
0
1
0
0
0
1
0
0
0
1
0
2
0
3
0
0
0
0
11
1
64
1
0
2
0
0
0
0
1
0
1
0
1
220
0
0
0
0
0
0
0
0
0
0
1
0
0
1
0
1
1
0
0
0
0
0
0
0
1
1
0
0
1
0
1


0
1
0
0
0
0
0
0
0
0
0
1
0
0
0
0
0
0
0
0
0
0
0
0
0
0
0
0
0
0
0
0
0
0
0
0
0
0
0
0
0
1
0
0
0
0
0
0
0
0
1
0
0
0
0
0
0
0
0
1
0
0
0
0
0
0
1
0
0
0
0
0
0
0
0
0
0
0
0
0
0


0
0
0
0
0
0
0
0
0
0
0
0
0
0
0
0
0
0
0
0
0
0
0
0
0
0
0
0
0
0
0
0
0
0
0
0
0
0
0
0
0
0
0
0
0
0
0
0
0
0
0
0
0
0
0
0
0
0
0
0
0
0
0
0
0
0
0
0
0
0
0
0
0
0
0
0
0
0
1
0
0


0
0
1
0
0
0
0
0
0
0
0
0
0
0
0
0
0
0
0
0
0
0
0
0
0
0
0
0
0
0
0
0
0
0
0
0
0
0
0
0
0
0
0
0
0
0
0
0
0
0
0
0
0
0
0
0
0
1
0
0
0
0
0
0
0
0
0
0
0
0
0
0
0
0
0
0
0
0
0
0
0


0
0
1
0
0
0
0
0
0
0
0
0
0
0
0
0
0
0
0
0
0
0
0
0
0
0
0
0
0
0
0
0
0
0
0
0
0
0
0
0
0
0
0
0
0
0
0
0
0
0
0
0
0
0
0
0
0
1
0
0
0
0
0
0
0
0
0
0
0
0
0
0
0
0
0
0
0
0
0
0
0


0
0
0
0
0
0
0
0
0
0
0
0
0
0
0
1
0
0
0
0
0
0
0
0
0
0
0
0
0
0
0
0
0
0
0
0
0
0
0
0
0
1
0
0
0
0
0
0
0
0
0
0
0
0
0
0
0
0
2
0
0
0
0
0
0
1
0
0
0
0
0
0
0
0
0
1
0
10
0
0
0


0
0
0
0
0
0
0
0
0
0
0
0
0
0
0
1
0
0
0
0
0
0
0
0
0
0
0
0
0
0
0
0
0
0
0
0
0
0
0
0
0
1
0
0
0
0
0
0
0
0
0
0
0
0
0
0
0
0
2
0
0
0
0
0
0
1
0
0
0
0
0
0
0
0
0
1
0
10
0
0
0


0
0
0
2
0
1
0
2
1
1
1
0
0
3
0
1
0
2
1
0
0
1
1
0
0
1
151
0
110
0
1
0
0
1
0
0
1
1
0
0
0
2
1
2
0
2
2
4
1
1
0
0
0
0
1
1
0
1
0
2
2
0
0
1
1
1
0
1
1
6
0
1
1
1
2
2
0
1
0
0
0


0
0
0
2
0
1
0
2
1
1
1
0
0
3
0
1
0
2
1
0
0
1
1
0
0
1
151
0
110
0
1
0
0
1
0
0
1
1
0
0
0
2
1
2
0
2
2
4
1
1
0
0
0
0
1
1
0
1
0
2
2
0
0
1
1
1
0
1
1
6
0
1
1
1
2
2
0
1
0
0
0


0
0
0
0
0
0
0
2
0
0
0
0
0
1
0
0
0
0
0
0
0
0
1
0
0
1
0
0
0
0
1
0
0
1
0
0
0
1
0
0
0
1
0
1
0
1
2
0
1
1
0
0
0
0
0
0
0
0
0
0
0
0
0
0
0
0
0
0
1
6
0
0
0
0
1
1
0
0
0
0
0


0
0
0
0
0
0
0
0
1
0
0
0
0
0
0
0
0
0
0
0
0
0
0
0
0
0
0
0
0
0
0
0
0
0
0
0
0
0
0
0
0
0
0
0
0
0
0
0
0
0
0
0
0
0
0
0
0
0
0
0
1
0
0
0
1
0
0
0
0
0
0
0
0
0
0
0
0
0
0
0
0


0
0
0
2
0
0
0
0
0
1
1
0
0
0
0
0
0
1
0
0
0
0
0
0
0
0
150
0
0
0
0
0
0
0
0
0
1
0
0
0
0
0
1
1
0
1
0
4
0
0
0
0
0
0
0
0
0
0
0
0
0
0
0
1
0
1
0
1
0
0
0
1
0
0
1
0
0
1
0
0
0


0
0
0
0
0
0
0
0
0
0
0
0
0
0
0
0
0
0
0
0
0
1
0
0
0
0
0
0
0
0
0
0
0
0
0
0
0
0
0
0
0
0
0
0
0
0
0
0
0
0
0
0
0
0
0
0
0
0
0
0
0
0
0
0
0
0
0
0
0
0
0
0
0
0
0
0
0
0
0
0
0


0
0
0
0
0
0
0
0
0
0
0
0
0
2
0
1
0
0
1
0
0
0
0
0
0
0
0
0
0
0
0
0
0
0
0
0
0
0
0
0
0
1
0
0
0
0
0
0
0
0
0
0
0
0
1
1
0
0
0
2
1
0
0
0
0
0
0
0
0
0
0
0
1
1
0
0
0
0
0
0
0


0
0
0
0
0
1
0
0
0
0
0
0
0
0
0
0
0
1
0
0
0
0
0
0
0
0
1
0
110
0
0
0
0
0
0
0
0
0
0
0
0
0
0
0
0
0
0
0
0
0
0
0
0
0
0
0
0
0
0
0
0
0
0
0
0
0
0
0
0
0
0
0
0
0
0
0
0
0
0
0
0


0
0
0
0
0
0
0
0
0
0
0
0
0
0
0
0
0
0
0
0
0
0
0
0
0
0
0
0
0
0
0
0
0
0
0
0
0
0
0
0
0
0
0
0
0
0
0
0
0
0
0
0
0
0
0
0
0
1
0
0
0
0
0
0
0
0
0
0
0
0
0
0
0
0
0
1
0
0
0
0
0


0
0
3
0
0
0
0
0
0
0
0
0
0
0
0
17
0
0
0
0
0
0
0
0
0
0
0
0
0
0
0
0
0
0
0
0
0
0
0
0
0
0
0
0
0
0
0
0
0
0
0
0
0
0
0
0
0
2
16
0
0
0
0
0
0
0
0
0
0
0
2
0
0
0
0
0
0
3
0
0
0


0
0
3
0
0
0
0
0
0
0
0
0
0
0
0
10
0
0
0
0
0
0
0
0
0
0
0
0
0
0
0
0
0
0
0
0
0
0
0
0
0
0
0
0
0
0
0
0
0
0
0
0
0
0
0
0
0
2
16
0
0
0
0
0
0
0
0
0
0
0
2
0
0
0
0
0
0
0
0
0
0


0
0
3
0
0
0
0
0
0
0
0
0
0
0
0
10
0
0
0
0
0
0
0
0
0
0
0
0
0
0
0
0
0
0
0
0
0
0
0
0
0
0
0
0
0
0
0
0
0
0
0
0
0
0
0
0
0
2
16
0
0
0
0
0
0
0
0
0
0
0
2
0
0
0
0
0
0
0
0
0
0


0
0
0
0
0
0
0
0
0
0
0
0
0
0
0
7
0
0
0
0
0
0
0
0
0
0
0
0
0
0
0
0
0
0
0
0
0
0
0
0
0
0
0
0
0
0
0
0
0
0
0
0
0
0
0
0
0
0
0
0
0
0
0
0
0
0
0
0
0
0
0
0
0
0
0
0
0
3
0
0
0


0
0
0
0
0
0
0
0
0
0
0
0
0
0
0
7
0
0
0
0
0
0
0
0
0
0
0
0
0
0
0
0
0
0
0
0
0
0
0
0
0
0
0
0
0
0
0
0
0
0
0
0
0
0
0
0
0
0
0
0
0
0
0
0
0
0
0
0
0
0
0
0
0
0
0
0
0
3
0
0
0


0
0
0
0
0
0
0
0
0
0
0
0
0
0
0
0
0
0
0
0
0
0
0
0
0
0
0
0
494
0
0
0
0
0
0
0
0
0
0
0
0
0
0
0
0
0
0
0
0
0
0
0
0
0
0
0
0
0
1
0
0
0
0
0
0
0
0
0
0
0
0
0
0
0
0
0
0
0
0
0
0


0
0
0
0
0
0
0
0
0
0
0
0
0
0
0
0
0
0
0
0
0
0
0
0
0
0
0
0
494
0
0
0
0
0
0
0
0
0
0
0
0
0
0
0
0
0
0
0
0
0
0
0
0
0
0
0
0
0
1
0
0
0
0
0
0
0
0
0
0
0
0
0
0
0
0
0
0
0
0
0
0


0
0
0
0
0
0
0
0
0
0
0
0
0
0
0
0
0
0
0
0
0
0
0
0
0
0
0
0
494
0
0
0
0
0
0
0
0
0
0
0
0
0
0
0
0
0
0
0
0
0
0
0
0
0
0
0
0
0
1
0
0
0
0
0
0
0
0
0
0
0
0
0
0
0
0
0
0
0
0
0
0


0
1
0
0
0
0
0
0
0
0
0
0
1
0
0
0
0
0
0
0
0
0
0
0
0
0
1
0
0
0
0
0
0
0
0
0
0
0
0
1
0
0
0
0
0
0
0
0
0
0
0
0
0
0
0
0
0
0
0
0
0
0
0
0
0
0
0
0
0
0
0
0
0
0
0
0
0
0
0
0
0


0
1
0
0
0
0
0
0
0
0
0
0
1
0
0
0
0
0
0
0
0
0
0
0
0
0
1
0
0
0
0
0
0
0
0
0
0
0
0
1
0
0
0
0
0
0
0
0
0
0
0
0
0
0
0
0
0
0
0
0
0
0
0
0
0
0
0
0
0
0
0
0
0
0
0
0
0
0
0
0
0


0
1
0
0
0
0
0
0
0
0
0
0
1
0
0
0
0
0
0
0
0
0
0
0
0
0
1
0
0
0
0
0
0
0
0
0
0
0
0
1
0
0
0
0
0
0
0
0
0
0
0
0
0
0
0
0
0
0
0
0
0
0
0
0
0
0
0
0
0
0
0
0
0
0
0
0
0
0
0
0
0


0
0
0
1
0
0
0
0
0
0
0
0
0
0
0
0
0
0
0
0
0
0
0
0
0
0
0
0
0
0
0
0
0
0
0
0
0
0
0
0
0
0
0
0
0
0
0
0
0
0
0
0
0
0
0
0
0
0
0
0
0
0
0
0
0
0
0
0
0
0
0
0
0
0
0
0
0
0
0
0
0


0
0
0
1
0
0
0
0
0
0
0
0
0
0
0
0
0
0
0
0
0
0
0
0
0
0
0
0
0
0
0
0
0
0
0
0
0
0
0
0
0
0
0
0
0
0
0
0
0
0
0
0
0
0
0
0
0
0
0
0
0
0
0
0
0
0
0
0
0
0
0
0
0
0
0
0
0
0
0
0
0


0
0
0
1
0
0
0
0
0
0
0
0
0
0
0
0
0
0
0
0
0
0
0
0
0
0
0
0
0
0
0
0
0
0
0
0
0
0
0
0
0
0
0
0
0
0
0
0
0
0
0
0
0
0
0
0
0
0
0
0
0
0
0
0
0
0
0
0
0
0
0
0
0
0
0
0
0
0
0
0
0


0
58
2
0
1
1
2
9
4
0
1
0
2
0
2
13
3
0
2
0
2
0
0
0
0
0
0
0
1
0
0
0
0
0
2
1
0
4
0
0
0
0
0
0
0
1
1
2
0
0
1
1
0
0
1
0
2
15
35
0
0
1
0
1
0
3
0
0
1
0
2
0
0
32
2
0
0
11
0
0
0


0
58
2
0
1
1
2
9
4
0
1
0
2
0
2
13
3
0
2
0
2
0
0
0
0
0
0
0
1
0
0
0
0
0
2
1
0
4
0
0
0
0
0
0
0
1
1
2
0
0
1
1
0
0
1
0
2
15
35
0
0
1
0
1
0
3
0
0
1
0
2
0
0
32
2
0
0
11
0
0
0


0
58
2
0
1
1
2
9
4
0
1
0
2
0
2
13
3
0
2
0
2
0
0
0
0
0
0
0
1
0
0
0
0
0
2
1
0
4
0
0
0
0
0
0
0
1
1
2
0
0
1
1
0
0
1
0
2
15
35
0
0
1
0
1
0
3
0
0
1
0
2
0
0
32
2
0
0
11
0
0
0


602
693
545
379
647
703
744
340
856
566
912
439
1009
564
577
329
315
447
853
450
390
342
533
0
0
237
231
173
0
87
177
0
185
5
55
262
1
88
187
134
232
363
67
135
82
163
242
24
473
0
472
528
626
512
1172
620
364
345
486
270
649
632
1411
400
362
307
117
341
127
152
561
77
96
721
163
22
12
29
658
237
110


602
693
545
379
647
703
744
340
856
566
912
439
1009
564
577
329
315
447
853
450
390
342
533
0
0
237
231
173
0
87
177
0
185
5
55
262
1
88
187
134
232
363
67
135
82
163
242
24
473
0
472
528
626
512
1172
620
364
345
486
270
649
632
1411
400
362
307
117
341
127
152
561
77
96
721
163
22
12
29
658
237
110


0
0
0
0
0
0
0
1
0
0
0
0
0
0
1
0
0
0
0
0
2
0
1
0
0
5
0
0
0
0
1
0
0
0
0
1
0
0
0
0
1
0
1
0
0
1
0
0
2
0
0
3
0
0
6
1
0
0
1
2
0
1
0
0
0
0
0
0
0
0
2
0
1
0
0
0
0
0
0
0
0


0
0
0
0
0
0
0
1
0
0
0
0
0
0
1
0
0
0
0
0
2
0
1
0
0
5
0
0
0
0
1
0
0
0
0
1
0
0
0
0
1
0
1
0
0
1
0
0
2
0
0
3
0
0
6
1
0
0
1
2
0
1
0
0
0
0
0
0
0
0
2
0
1
0
0
0
0
0
0
0
0


0
0
0
0
0
0
0
1
0
0
0
0
0
0
1
0
0
0
0
0
2
0
1
0
0
5
0
0
0
0
1
0
0
0
0
1
0
0
0
0
1
0
1
0
0
1
0
0
2
0
0
3
0
0
6
1
0
0
1
2
0
1
0
0
0
0
0
0
0
0
2
0
1
0
0
0
0
0
0
0
0


12
0
0
1
1
2
1
1
0
2
2
12
13
0
3
8
4
7
1
1
6
2
1
0
0
5
0
5
0
15
1
0
0
0
9
7
0
0
7
0
5
1
1
16
21
5
18
0
6
0
0
2
0
2
5
1
0
8
1
4
4
1
2
1
13
0
0
0
1
10
0
1
3
0
0
0
3
0
1
1
0


0
0
0
0
0
2
0
0
0
0
0
7
11
0
0
3
0
0
0
0
0
0
1
0
0
0
0
0
0
13
0
0
0
0
9
1
0
0
0
0
1
0
1
0
3
2
15
0
6
0
0
1
0
1
2
0
0
2
0
1
1
0
0
0
3
0
0
0
1
0
0
1
0
0
0
0
3
0
0
0
0


0
0
0
0
0
2
0
0
0
0
0
7
11
0
0
3
0
0
0
0
0
0
1
0
0
0
0
0
0
13
0
0
0
0
9
1
0
0
0
0
1
0
1
0
3
2
15
0
6
0
0
1
0
1
2
0
0
2
0
1
1
0
0
0
3
0
0
0
1
0
0
1
0
0
0
0
3
0
0
0
0


1
0
0
1
0
0
0
1
0
2
2
1
2
0
3
5
4
4
1
1
5
2
0
0
0
5
0
5
0
0
1
0
0
0
0
6
0
0
7
0
3
1
0
0
2
1
2
0
0
0
0
1
0
1
3
1
0
1
1
2
2
1
2
1
9
0
0
0
0
10
0
0
3
0
0
0
0
0
1
1
0


1
0
0
1
0
0
0
1
0
2
2
1
2
0
3
5
4
4
1
1
5
2
0
0
0
5
0
5
0
0
1
0
0
0
0
6
0
0
7
0
3
1
0
0
2
1
2
0
0
0
0
1
0
1
3
1
0
1
1
2
2
1
2
1
9
0
0
0
0
10
0
0
3
0
0
0
0
0
1
1
0


0
0
0
0
0
0
0
0
0
0
0
0
0
0
0
0
0
0
0
0
0
0
0
0
0
0
0
0
0
0
0
0
0
0
0
0
0
0
0
0
0
0
0
16
16
0
0
0
0
0
0
0
0
0
0
0
0
5
0
1
0
0
0
0
1
0
0
0
0
0
0
0
0
0
0
0
0
0
0
0
0


0
0
0
0
0
0
0
0
0
0
0
0
0
0
0
0
0
0
0
0
0
0
0
0
0
0
0
0
0
0
0
0
0
0
0
0
0
0
0
0
0
0
0
16
16
0
0
0
0
0
0
0
0
0
0
0
0
5
0
1
0
0
0
0
1
0
0
0
0
0
0
0
0
0
0
0
0
0
0
0
0


0
0
0
0
0
0
0
0
0
0
0
0
0
0
0
0
0
0
0
0
0
0
0
0
0
0
0
0
0
0
0
0
0
0
0
0
0
0
0
0
0
0
0
0
0
2
1
0
0
0
0
0
0
0
0
0
0
0
0
0
0
0
0
0
0
0
0
0
0
0
0
0
0
0
0
0
0
0
0
0
0


0
0
0
0
0
0
0
0
0
0
0
0
0
0
0
0
0
0
0
0
0
0
0
0
0
0
0
0
0
0
0
0
0
0
0
0
0
0
0
0
0
0
0
0
0
2
1
0
0
0
0
0
0
0
0
0
0
0
0
0
0
0
0
0
0
0
0
0
0
0
0
0
0
0
0
0
0
0
0
0
0


0
0
0
0
0
0
1
0
0
0
0
0
0
0
0
0
0
0
0
0
0
0
0
0
0
0
0
0
0
0
0
0
0
0
0
0
0
0
0
0
0
0
0
0
0
0
0
0
0
0
0
0
0
0
0
0
0
0
0
0
1
0
0
0
0
0
0
0
0
0
0
0
0
0
0
0
0
0
0
0
0


0
0
0
0
0
0
1
0
0
0
0
0
0
0
0
0
0
0
0
0
0
0
0
0
0
0
0
0
0
0
0
0
0
0
0
0
0
0
0
0
0
0
0
0
0
0
0
0
0
0
0
0
0
0
0
0
0
0
0
0
1
0
0
0
0
0
0
0
0
0
0
0
0
0
0
0
0
0
0
0
0


11
0
0
0
1
0
0
0
0
0
0
4
0
0
0
0
0
3
0
0
1
0
0
0
0
0
0
0
0
2
0
0
0
0
0
0
0
0
0
0
1
0
0
0
0
0
0
0
0
0
0
0
0
0
0
0
0
0
0
0
0
0
0
0
0
0
0
0
0
0
0
0
0
0
0
0
0
0
0
0
0


11
0
0
0
1
0
0
0
0
0
0
4
0
0
0
0
0
3
0
0
1
0
0
0
0
0
0
0
0
2
0
0
0
0
0
0
0
0
0
0
1
0
0
0
0
0
0
0
0
0
0
0
0
0
0
0
0
0
0
0
0
0
0
0
0
0
0
0
0
0
0
0
0
0
0
0
0
0
0
0
0


587
692
544
378
645
700
743
338
852
560
909
423
996
564
569
318
311
440
849
449
373
336
529
0
0
226
156
167
0
69
168
0
185
5
41
254
1
88
178
132
225
362
64
116
58
150
215
20
464
0
472
502
623
510
1157
610
362
336
483
264
642
628
1407
397
349
307
116
335
126
142
556
76
92
720
161
22
7
28
656
236
110


533
534
383
310
410
665
624
304
786
523
846
388
820
494
484
272
282
366
698
408
153
234
414
0
0
183
86
131
0
46
60
0
165
5
41
224
1
75
173
97
182
314
49
90
34
102
158
10
435
0
409
290
519
379
971
450
302
258
342
198
438
504
1379
348
317
254
112
219
111
124
489
13
48
660
132
9
2
13
623
171
63


533
534
383
310
410
665
624
304
786
523
846
388
820
494
484
272
282
366
698
408
153
234
414
0
0
183
86
131
0
46
60
0
165
5
41
224
1
75
173
97
182
314
49
90
34
102
158
10
435
0
409
290
519
379
971
450
302
258
342
198
438
504
1379
348
317
254
112
219
111
124
489
13
48
660
132
9
2
13
623
171
63


34
141
106
5
91
18
70
2
33
8
28
10
127
25
64
31
19
37
105
13
178
19
85
0
0
9
0
7
0
0
71
0
0
0
0
3
0
1
1
9
6
4
6
9
6
8
12
0
16
0
52
192
78
124
145
135
37
56
118
59
156
97
2
36
31
37
2
100
9
18
48
2
16
29
0
0
0
0
15
62
42


34
141
106
5
91
18
70
2
33
8
28
10
127
25
64
31
19
37
105
13
178
19
85
0
0
9
0
7
0
0
71
0
0
0
0
3
0
1
1
9
6
4
6
9
6
8
12
0
16
0
52
192
78
124
145
135
37
56
118
59
156
97
2
36
31
37
2
100
9
18
48
2
16
29
0
0
0
0
15
62
42


0
0
0
0
0
0
0
0
0
0
0
1
0
1
0
0
3
0
4
0
1
2
1
0
0
0
0
0
0
0
0
0
15
0
0
0
0
0
0
0
0
0
0
0
0
8
0
0
2
0
0
0
2
1
9
0
2
0
2
0
0
0
0
0
0
0
0
0
0
0
0
0
2
9
0
0
0
0
1
0
0


0
0
0
0
0
0
0
0
0
0
0
1
0
1
0
0
3
0
4
0
1
2
1
0
0
0
0
0
0
0
0
0
15
0
0
0
0
0
0
0
0
0
0
0
0
8
0
0
2
0
0
0
2
1
9
0
2
0
2
0
0
0
0
0
0
0
0
0
0
0
0
0
2
9
0
0
0
0
1
0
0


12
3
43
40
95
1
10
8
8
18
9
9
8
26
1
3
3
1
11
5
5
65
8
0
0
20
0
6
0
9
7
0
0
0
0
14
0
4
0
6
7
14
0
0
1
9
0
0
1
0
0
8
17
0
13
0
5
4
6
1
8
6
7
3
0
3
0
0
2
0
1
18
6
1
25
0
0
4
2
0
0


12
3
43
40
95
1
10
8
8
18
9
9
8
26
1
3
3
1
11
5
5
65
8
0
0
20
0
6
0
9
7
0
0
0
0
14
0
4
0
6
7
14
0
0
1
9
0
0
1
0
0
8
17
0
13
0
5
4
6
1
8
6
7
3
0
3
0
0
2
0
1
18
6
1
25
0
0
4
2
0
0


0
0
1
0
1
1
0
0
0
1
1
3
0
0
0
0
0
0
5
0
1
1
3
0
0
0
0
0
0
0
0
0
0
0
0
0
0
6
0
0
5
10
0
0
0
0
0
0
1
0
1
0
0
0
2
0
1
1
0
0
0
0
4
0
0
1
0
0
0
0
2
0
2
0
0
0
0
0
0
0
0


0
0
1
0
1
1
0
0
0
1
1
3
0
0
0
0
0
0
5
0
1
1
3
0
0
0
0
0
0
0
0
0
0
0
0
0
0
6
0
0
5
10
0
0
0
0
0
0
1
0
1
0
0
0
2
0
1
1
0
0
0
0
4
0
0
1
0
0
0
0
2
0
2
0
0
0
0
0
0
0
0


0
0
0
1
7
0
0
3
0
0
0
0
1
0
0
0
0
0
0
0
2
2
0
0
0
1
0
0
0
0
6
0
0
0
0
0
0
0
0
4
0
0
0
0
2
0
0
0
2
0
0
0
0
0
0
0
0
0
0
0
0
0
0
0
0
0
0
0
0
0
0
5
3
0
0
6
1
4
1
0
1


0
0
0
1
7
0
0
3
0
0
0
0
1
0
0
0
0
0
0
0
2
2
0
0
0
1
0
0
0
0
6
0
0
0
0
0
0
0
0
4
0
0
0
0
2
0
0
0
2
0
0
0
0
0
0
0
0
0
0
0
0
0
0
0
0
0
0
0
0
0
0
5
3
0
0
6
1
4
1
0
1


0
0
0
0
2
0
0
0
0
0
0
0
0
0
0
0
0
0
0
0
0
0
0
0
0
0
0
0
0
0
0
0
0
0
0
0
0
0
0
0
1
0
0
0
0
0
0
0
0
0
0
0
0
0
0
0
1
0
0
0
0
0
0
0
0
0
0
0
0
0
0
0
1
0
0
0
0
1
1
0
0


0
0
0
0
2
0
0
0
0
0
0
0
0
0
0
0
0
0
0
0
0
0
0
0
0
0
0
0
0
0
0
0
0
0
0
0
0
0
0
0
1
0
0
0
0
0
0
0
0
0
0
0
0
0
0
0
1
0
0
0
0
0
0
0
0
0
0
0
0
0
0
0
1
0
0
0
0
1
1
0
0


0
0
0
0
0
0
0
0
0
0
0
0
0
0
0
0
0
0
0
0
0
0
0
0
0
0
0
0
0
0
0
0
0
0
0
0
0
0
0
0
0
0
0
0
0
0
0
0
0
0
0
0
0
0
0
0
1
0
0
0
0
0
0
0
0
0
0
0
0
0
0
0
0
0
0
0
0
0
0
0
0


0
0
0
0
0
0
0
0
0
0
0
0
0
0
0
0
0
0
0
0
0
0
0
0
0
0
0
0
0
0
0
0
0
0
0
0
0
0
0
0
0
0
0
0
0
0
0
0
0
0
0
0
0
0
0
0
1
0
0
0
0
0
0
0
0
0
0
0
0
0
0
0
0
0
0
0
0
0
0
0
0


0
0
0
0
0
0
0
0
0
0
0
0
0
0
0
0
0
0
0
0
0
0
0
0
0
0
0
0
0
0
5
0
0
0
0
0
0
0
1
0
0
0
0
0
0
0
1
0
0
0
0
0
0
0
0
1
0
0
0
0
0
0
0
0
0
0
0
0
0
0
0
0
0
0
0
0
0
0
0
0
0


0
0
0
0
0
0
0
0
0
0
0
0
0
0
0
0
0
0
0
0
0
0
0
0
0
0
0
0
0
0
5
0
0
0
0
0
0
0
1
0
0
0
0
0
0
0
1
0
0
0
0
0
0
0
0
1
0
0
0
0
0
0
0
0
0
0
0
0
0
0
0
0
0
0
0
0
0
0
0
0
0


0
0
1
0
0
0
0
0
0
0
1
0
0
0
0
1
0
1
0
0
16
1
0
0
0
0
70
14
0
9
7
0
0
0
0
0
0
0
1
12
21
6
6
15
8
4
32
9
4
0
2
7
4
0
0
0
5
3
2
2
0
2
2
1
0
2
0
1
0
0
0
0
0
0
0
0
0
0
0
0
0


0
0
1
0
0
0
0
0
0
0
1
0
0
0
0
1
0
1
0
0
16
1
0
0
0
0
70
14
0
9
7
0
0
0
0
0
0
0
1
12
21
6
6
15
8
4
32
9
4
0
2
7
4
0
0
0
5
3
2
2
0
2
2
1
0
2
0
1
0
0
0
0
0
0
0
0
0
0
0
0
0


0
0
0
0
0
0
0
0
0
0
0
0
0
0
0
0
0
0
0
0
0
0
0
0
0
0
0
0
0
1
0
0
0
0
0
0
0
0
0
0
0
0
0
0
0
0
0
0
0
0
0
0
0
0
0
0
0
0
0
0
0
0
0
0
0
0
0
0
0
0
0
0
0
0
0
0
0
0
0
0
0


0
0
0
0
0
0
0
0
0
0
0
0
0
0
0
0
0
0
0
0
0
0
0
0
0
0
0
0
0
1
0
0
0
0
0
0
0
0
0
0
0
0
0
0
0
0
0
0
0
0
0
0
0
0
0
0
0
0
0
0
0
0
0
0
0
0
0
0
0
0
0
0
0
0
0
0
0
0
0
0
0


0
0
0
0
0
0
0
0
0
0
0
0
0
0
0
0
0
0
0
0
0
0
0
0
0
0
0
0
0
0
0
0
0
0
0
0
0
0
0
0
0
0
0
0
0
0
0
0
0
0
0
0
0
0
0
0
1
0
0
0
0
0
0
0
0
0
0
0
0
0
0
0
0
0
0
0
0
0
0
0
0


0
0
0
0
0
0
0
0
0
0
0
0
0
0
0
0
0
0
0
0
0
0
0
0
0
0
0
0
0
0
0
0
0
0
0
0
0
0
0
0
0
0
0
0
0
0
0
0
0
0
0
0
0
0
0
0
1
0
0
0
0
0
0
0
0
0
0
0
0
0
0
0
0
0
0
0
0
0
0
0
0


0
0
0
1
1
0
0
0
0
0
0
0
0
1
0
3
0
2
0
0
0
3
0
0
0
0
0
0
0
0
0
0
0
0
0
0
0
0
0
0
0
0
0
0
0
5
0
0
0
0
1
0
0
0
0
2
1
0
1
0
0
0
2
0
0
0
0
0
0
0
0
29
3
1
3
0
0
1
3
0
0


0
0
0
1
1
0
0
0
0
0
0
0
0
1
0
3
0
2
0
0
0
3
0
0
0
0
0
0
0
0
0
0
0
0
0
0
0
0
0
0
0
0
0
0
0
5
0
0
0
0
1
0
0
0
0
2
1
0
1
0
0
0
2
0
0
0
0
0
0
0
0
29
3
1
3
0
0
1
3
0
0


6
9
4
20
26
9
27
17
14
8
21
7
36
9
16
7
3
27
20
17
13
5
12
0
0
0
0
0
0
2
8
0
0
0
0
9
0
0
1
3
2
5
1
2
3
5
3
0
3
0
6
1
2
2
11
8
3
10
9
3
31
15
7
6
1
7
1
7
2
0
8
5
7
13
1
0
3
3
9
1
2


6
9
4
20
26
9
27
17
14
8
21
7
36
9
16
7
3
27
20
17
13
5
12
0
0
0
0
0
0
2
8
0
0
0
0
9
0
0
1
3
2
5
1
2
3
5
3
0
3
0
6
1
2
2
11
8
3
10
9
3
31
15
7
6
1
7
1
7
2
0
8
5
7
13
1
0
3
3
9
1
2


2
5
6
1
12
6
12
4
11
2
3
5
4
8
4
1
1
6
6
6
4
4
6
0
0
13
0
9
0
2
4
0
5
0
0
4
0
2
1
1
1
9
2
0
4
9
9
1
0
0
1
4
1
4
6
14
3
4
3
1
9
4
4
3
0
3
1
8
2
0
8
4
4
7
0
7
1
2
1
2
2


2
5
6
1
12
6
12
4
11
2
3
5
4
8
4
1
1
6
6
6
4
4
6
0
0
13
0
9
0
2
4
0
5
0
0
4
0
2
1
1
1
9
2
0
4
9
9
1
0
0
1
4
1
4
6
14
3
4
3
1
9
4
4
3
0
3
1
8
2
0
8
4
4
7
0
7
1
2
1
2
2


0
0
0
0
0
0
0
0
0
0
0
0
0
0
0
0
0
0
0
0
0
0
1
0
0
0
0
0
0
2
0
0
0
0
0
0
0
0
0
0
0
0
0
0
0
0
0
0
0
0
0
0
0
0
0
0
0
0
0
0
0
0
0
0
0
0
0
0
0
0
0
0
0
0
0
0
0
0
1
0
0


0
0
0
0
0
0
0
0
0
0
0
0
0
0
0
0
0
0
0
0
0
0
0
0
0
0
0
0
0
2
0
0
0
0
0
0
0
0
0
0
0
0
0
0
0
0
0
0
0
0
0
0
0
0
0
0
0
0
0
0
0
0
0
0
0
0
0
0
0
0
0
0
0
0
0
0
0
0
0
0
0


0
0
0
0
0
0
0
0
0
0
0
0
0
0
0
0
0
0
0
0
0
0
0
0
0
0
0
0
0
2
0
0
0
0
0
0
0
0
0
0
0
0
0
0
0
0
0
0
0
0
0
0
0
0
0
0
0
0
0
0
0
0
0
0
0
0
0
0
0
0
0
0
0
0
0
0
0
0
0
0
0


0
0
0
0
0
0
0
0
0
0
0
0
0
0
0
0
0
0
0
0
0
0
1
0
0
0
0
0
0
0
0
0
0
0
0
0
0
0
0
0
0
0
0
0
0
0
0
0
0
0
0
0
0
0
0
0
0
0
0
0
0
0
0
0
0
0
0
0
0
0
0
0
0
0
0
0
0
0
1
0
0


0
0
0
0
0
0
0
0
0
0
0
0
0
0
0
0
0
0
0
0
0
0
1
0
0
0
0
0
0
0
0
0
0
0
0
0
0
0
0
0
0
0
0
0
0
0
0
0
0
0
0
0
0
0
0
0
0
0
0
0
0
0
0
0
0
0
0
0
0
0
0
0
0
0
0
0
0
0
1
0
0


3
1
1
0
1
1
0
0
4
4
1
4
0
0
4
3
0
0
3
0
9
4
1
0
0
1
75
1
0
1
7
0
0
0
5
0
0
0
2
2
1
0
1
3
3
7
9
4
1
0
0
21
3
0
4
8
2
1
1
0
3
2
2
2
0
0
1
6
0
0
3
0
0
1
2
0
2
1
0
0
0


3
1
1
0
1
1
0
0
4
4
1
4
0
0
4
3
0
0
3
0
9
4
1
0
0
1
75
1
0
1
7
0
0
0
5
0
0
0
2
2
1
0
1
3
3
7
9
4
1
0
0
21
3
0
4
8
2
1
1
0
3
2
2
2
0
0
1
6
0
0
3
0
0
1
2
0
2
1
0
0
0


3
1
1
0
1
1
0
0
4
4
1
4
0
0
4
3
0
0
3
0
9
4
1
0
0
1
75
1
0
1
7
0
0
0
5
0
0
0
2
2
1
0
1
3
3
7
9
4
1
0
0
21
3
0
4
8
2
1
1
0
3
2
2
2
0
0
1
6
0
0
3
0
0
1
2
0
2
1
0
0
0


131
133
61
45
317
44
348
93
293
89
140
95
129
105
89
90
267
45
156
58
65
402
100
188
45
12
0
9
66
10
221
2
95
0
4
61
4
65
41
49
36
55
10
9
10
133
8
14
27
1
160
49
110
130
225
129
123
75
149
84
336
232
52
114
66
65
8
76
29
7
126
34
48
173
12
5
4
29
91
35
21


131
133
61
45
317
44
348
93
293
89
140
95
129
105
89
90
267
45
156
58
65
402
100
188
45
12
0
9
66
10
221
2
95
0
4
61
4
65
41
49
36
55
10
9
10
133
8
14
27
1
160
49
110
130
225
129
123
75
149
84
336
232
52
114
66
65
8
76
29
7
126
34
48
173
12
5
4
29
91
35
21


0
0
0
0
0
0
0
0
0
0
0
0
0
0
0
0
0
0
0
0
0
0
0
0
0
0
0
0
0
0
0
0
0
0
0
0
0
0
0
0
0
0
0
0
0
0
0
0
0
0
0
1
0
0
0
0
0
0
0
0
0
0
1
0
0
0
0
0
0
0
0
0
0
0
0
0
0
0
0
0
0


0
0
0
0
0
0
0
0
0
0
0
0
0
0
0
0
0
0
0
0
0
0
0
0
0
0
0
0
0
0
0
0
0
0
0
0
0
0
0
0
0
0
0
0
0
0
0
0
0
0
0
1
0
0
0
0
0
0
0
0
0
0
1
0
0
0
0
0
0
0
0
0
0
0
0
0
0
0
0
0
0


0
0
0
0
0
0
0
0
0
0
0
0
0
0
0
0
0
0
0
0
0
0
0
0
0
0
0
0
0
0
0
0
0
0
0
0
0
0
0
0
0
0
0
0
0
0
0
0
0
0
0
1
0
0
0
0
0
0
0
0
0
0
1
0
0
0
0
0
0
0
0
0
0
0
0
0
0
0
0
0
0


0
0
0
3
0
0
166
0
0
0
0
0
0
0
0
0
0
0
0
0
0
0
0
0
0
0
0
0
0
0
0
0
0
0
0
1
0
0
0
0
0
0
0
0
0
0
0
0
0
0
0
0
0
0
0
0
0
30
0
5
0
0
0
0
0
0
0
0
0
0
0
0
4
0
0
0
0
0
0
0
0


0
0
0
3
0
0
166
0
0
0
0
0
0
0
0
0
0
0
0
0
0
0
0
0
0
0
0
0
0
0
0
0
0
0
0
1
0
0
0
0
0
0
0
0
0
0
0
0
0
0
0
0
0
0
0
0
0
30
0
5
0
0
0
0
0
0
0
0
0
0
0
0
4
0
0
0
0
0
0
0
0


0
0
0
3
0
0
166
0
0
0
0
0
0
0
0
0
0
0
0
0
0
0
0
0
0
0
0
0
0
0
0
0
0
0
0
1
0
0
0
0
0
0
0
0
0
0
0
0
0
0
0
0
0
0
0
0
0
30
0
5
0
0
0
0
0
0
0
0
0
0
0
0
4
0
0
0
0
0
0
0
0


19
102
47
2
43
3
72
23
86
12
16
30
42
27
28
17
201
17
48
27
37
22
67
0
0
10
0
2
64
6
13
0
25
0
3
2
0
63
29
23
31
30
3
5
5
9
7
14
24
0
106
27
21
56
60
44
46
20
55
55
26
150
10
36
17
22
2
22
10
0
29
2
0
1
0
0
0
2
1
1
3


19
98
44
2
38
2
64
17
72
7
14
30
35
20
26
15
198
14
35
25
35
15
67
0
0
9
0
1
64
0
11
0
0
0
3
2
0
63
24
17
21
20
3
3
3
2
6
9
22
0
106
27
21
43
54
26
44
20
28
53
22
146
7
35
16
20
2
15
8
0
27
0
0
1
0
0
0
2
1
1
0


19
98
44
2
38
2
64
17
72
7
14
30
35
20
26
15
198
14
35
25
35
15
67
0
0
9
0
1
64
0
11
0
0
0
3
2
0
63
24
17
21
20
3
3
3
2
6
9
22
0
106
27
21
43
54
26
44
20
28
53
22
146
7
35
16
20
2
15
8
0
27
0
0
1
0
0
0
2
1
1
0


0
0
0
0
0
1
0
0
0
0
0
0
6
0
0
0
0
0
10
0
0
0
0
0
0
0
0
0
0
0
0
0
0
0
0
0
0
0
0
0
0
0
0
0
0
0
0
0
0
0
0
0
0
0
0
0
0
0
0
0
1
0
0
0
0
0
0
0
0
0
0
0
0
0
0
0
0
0
0
0
0


0
0
0
0
0
1
0
0
0
0
0
0
6
0
0
0
0
0
10
0
0
0
0
0
0
0
0
0
0
0
0
0
0
0
0
0
0
0
0
0
0
0
0
0
0
0
0
0
0
0
0
0
0
0
0
0
0
0
0
0
1
0
0
0
0
0
0
0
0
0
0
0
0
0
0
0
0
0
0
0
0


0
4
3
0
5
0
6
6
14
5
2
0
1
7
2
2
3
3
3
2
2
7
0
0
0
1
0
1
0
6
2
0
25
0
0
0
0
0
5
6
10
10
0
2
2
7
1
5
2
0
0
0
0
13
6
18
2
0
27
2
3
3
3
1
1
2
0
7
2
0
2
2
0
0
0
0
0
0
0
0
3


0
4
3
0
5
0
6
6
14
5
2
0
1
7
2
2
3
3
3
2
2
7
0
0
0
1
0
1
0
6
2
0
25
0
0
0
0
0
5
6
10
10
0
2
2
7
1
5
2
0
0
0
0
13
6
18
2
0
27
2
3
3
3
1
1
2
0
7
2
0
2
2
0
0
0
0
0
0
0
0
3


0
0
0
0
0
0
2
0
0
0
0
0
0
0
0
0
0
0
0
0
0
0
0
0
0
0
0
0
0
0
0
0
0
0
0
0
0
0
0
0
0
0
0
0
0
0
0
0
0
0
0
0
0
0
0
0
0
0
0
0
0
1
0
0
0
0
0
0
0
0
0
0
0
0
0
0
0
0
0
0
0


0
0
0
0
0
0
2
0
0
0
0
0
0
0
0
0
0
0
0
0
0
0
0
0
0
0
0
0
0
0
0
0
0
0
0
0
0
0
0
0
0
0
0
0
0
0
0
0
0
0
0
0
0
0
0
0
0
0
0
0
0
1
0
0
0
0
0
0
0
0
0
0
0
0
0
0
0
0
0
0
0


95
29
13
35
228
39
108
70
201
68
70
65
80
72
56
69
59
21
81
22
19
375
33
188
0
0
0
1
0
4
11
2
70
0
0
54
4
0
3
21
4
20
5
2
3
113
1
0
3
1
48
19
81
72
155
79
65
24
93
21
284
80
12
76
47
37
5
46
13
7
94
22
44
171
8
2
4
14
90
33
18


0
1
1
0
0
0
1
0
0
1
0
0
0
0
0
0
0
0
0
0
1
0
0
0
0
0
0
0
0
2
0
1
0
0
0
0
0
0
0
1
0
0
2
0
0
0
0
0
0
1
0
0
0
0
0
0
0
1
0
0
0
0
0
0
1
0
0
0
0
0
1
0
0
1
0
0
0
0
0
0
0


0
1
0
0
0
0
0
0
0
1
0
0
0
0
0
0
0
0
0
0
0
0
0
0
0
0
0
0
0
0
0
0
0
0
0
0
0
0
0
1
0
0
0
0
0
0
0
0
0
0
0
0
0
0
0
0
0
0
0
0
0
0
0
0
0
0
0
0
0
0
0
0
0
0
0
0
0
0
0
0
0


0
0
1
0
0
0
0
0
0
0
0
0
0
0
0
0
0
0
0
0
1
0
0
0
0
0
0
0
0
2
0
1
0
0
0
0
0
0
0
0
0
0
2
0
0
0
0
0
0
1
0
0
0
0
0
0
0
1
0
0
0
0
0
0
1
0
0
0
0
0
1
0
0
0
0
0
0
0
0
0
0


0
0
0
0
0
0
1
0
0
0
0
0
0
0
0
0
0
0
0
0
0
0
0
0
0
0
0
0
0
0
0
0
0
0
0
0
0
0
0
0
0
0
0
0
0
0
0
0
0
0
0
0
0
0
0
0
0
0
0
0
0
0
0
0
0
0
0
0
0
0
0
0
0
1
0
0
0
0
0
0
0


95
28
12
34
205
39
107
70
200
67
70
63
80
71
56
68
58
21
81
22
18
373
33
188
0
0
0
1
0
2
11
1
70
0
0
54
4
0
3
14
4
20
3
2
3
113
1
0
3
0
48
19
81
71
155
78
63
23
93
19
284
79
12
76
46
37
5
46
13
7
93
22
43
169
8
2
4
14
90
33
18


66
5
8
29
80
28
74
32
156
43
51
40
59
34
32
23
28
16
55
12
11
111
16
0
0
0
0
1
0
1
4
1
46
0
0
16
0
0
3
12
2
7
1
2
2
30
1
0
3
0
37
16
66
57
122
50
31
16
85
18
225
66
6
60
37
27
5
19
11
6
64
15
39
147
3
1
4
10
68
32
16


0
13
0
0
0
0
5
4
0
0
1
0
5
2
4
6
0
0
8
2
0
1
5
0
0
0
0
0
0
0
0
0
0
0
0
0
0
0
0
0
0
1
0
0
0
1
0
0
0
0
0
0
0
0
5
5
6
0
0
0
0
1
0
0
0
0
0
8
0
0
0
0
0
1
0
0
0
0
20
0
0


17
1
3
4
82
1
11
26
6
16
6
16
3
19
5
28
13
3
2
3
3
122
9
167
0
0
0
0
0
0
4
0
23
0
0
37
0
0
0
1
2
10
0
0
0
79
0
0
0
0
1
2
10
0
10
0
20
1
1
0
10
3
2
1
8
1
0
5
0
0
15
2
0
1
2
1
0
1
2
0
0


12
9
1
1
43
10
17
8
38
8
12
7
13
16
15
11
17
2
16
5
4
139
3
21
0
0
0
0
0
1
3
0
1
0
0
1
4
0
0
1
0
2
2
0
1
3
0
0
0
0
10
1
5
14
18
23
6
6
7
1
49
9
4
15
1
9
0
14
2
1
14
5
4
20
3
0
0
3
0
1
2


0
0
0
1
23
0
0
0
1
0
0
2
0
1
0
1
1
0
0
0
0
2
0
0
0
0
0
0
0
0
0
0
0
0
0
0
0
0
0
6
0
0
0
0
0
0
0
0
0
0
0
0
0
1
0
1
2
0
0
2
0
1
0
0
0
0
0
0
0
0
0
0
1
1
0
0
0
0
0
0
0


0
0
0
1
23
0
0
0
1
0
0
2
0
1
0
1
1
0
0
0
0
2
0
0
0
0
0
0
0
0
0
0
0
0
0
0
0
0
0
6
0
0
0
0
0
0
0
0
0
0
0
0
0
1
0
1
2
0
0
2
0
1
0
0
0
0
0
0
0
0
0
0
1
1
0
0
0
0
0
0
0


17
1
0
5
46
2
1
0
6
9
53
0
6
6
4
3
4
1
13
0
8
5
0
0
45
2
0
0
2
0
193
0
0
0
0
3
0
0
0
3
1
5
0
2
2
10
0
0
0
0
3
2
3
1
5
5
12
0
1
1
25
2
26
1
2
6
1
8
4
0
2
10
0
1
4
3
0
13
0
1
0


0
1
0
0
1
0
0
0
0
0
0
0
0
0
0
0
0
0
0
0
0
0
0
0
1
0
0
0
0
0
1
0
0
0
0
0
0
0
0
0
1
0
0
0
0
0
0
0
0
0
0
0
0
0
0
2
0
0
0
0
0
0
0
0
0
0
0
0
1
0
0
0
0
0
0
0
0
0
0
0
0


0
1
0
0
1
0
0
0
0
0
0
0
0
0
0
0
0
0
0
0
0
0
0
0
1
0
0
0
0
0
1
0
0
0
0
0
0
0
0
0
1
0
0
0
0
0
0
0
0
0
0
0
0
0
0
2
0
0
0
0
0
0
0
0
0
0
0
0
1
0
0
0
0
0
0
0
0
0
0
0
0


16
0
0
5
45
0
1
0
6
9
53
0
6
6
2
3
4
1
13
0
8
5
0
0
44
2
0
0
0
0
182
0
0
0
0
3
0
0
0
3
0
5
0
2
1
8
0
0
0
0
3
2
3
0
5
3
12
0
1
1
24
2
26
1
2
6
1
8
3
0
2
10
0
1
1
1
0
13
0
1
0


16
0
0
5
45
0
1
0
6
9
53
0
6
6
2
3
4
1
13
0
8
5
0
0
44
2
0
0
0
0
182
0
0
0
0
3
0
0
0
3
0
5
0
2
1
8
0
0
0
0
3
2
3
0
5
3
12
0
1
1
24
2
26
1
2
6
1
8
3
0
2
10
0
1
1
1
0
13
0
1
0


0
0
0
0
0
0
0
0
0
0
0
0
0
0
0
0
0
0
0
0
0
0
0
0
0
0
0
0
0
0
0
0
0
0
0
0
0
0
0
0
0
0
0
0
0
0
0
0
0
0
0
0
0
0
0
0
0
0
0
0
0
0
0
0
0
0
0
0
0
0
0
0
0
0
1
2
0
0
0
0
0


0
0
0
0
0
0
0
0
0
0
0
0
0
0
0
0
0
0
0
0
0
0
0
0
0
0
0
0
0
0
0
0
0
0
0
0
0
0
0
0
0
0
0
0
0
0
0
0
0
0
0
0
0
0
0
0
0
0
0
0
0
0
0
0
0
0
0
0
0
0
0
0
0
0
1
2
0
0
0
0
0


0
0
0
0
0
0
0
0
0
0
0
0
0
0
0
0
0
0
0
0
0
0
0
0
0
0
0
0
2
0
0
0
0
0
0
0
0
0
0
0
0
0
0
0
0
0
0
0
0
0
0
0
0
0
0
0
0
0
0
0
0
0
0
0
0
0
0
0
0
0
0
0
0
0
0
0
0
0
0
0
0


0
0
0
0
0
0
0
0
0
0
0
0
0
0
0
0
0
0
0
0
0
0
0
0
0
0
0
0
2
0
0
0
0
0
0
0
0
0
0
0
0
0
0
0
0
0
0
0
0
0
0
0
0
0
0
0
0
0
0
0
0
0
0
0
0
0
0
0
0
0
0
0
0
0
0
0
0
0
0
0
0


0
0
0
0
0
1
0
0
0
0
0
0
0
0
0
0
0
0
0
0
0
0
0
0
0
0
0
0
0
0
0
0
0
0
0
0
0
0
0
0
0
0
0
0
1
0
0
0
0
0
0
0
0
0
0
0
0
0
0
0
0
0
0
0
0
0
0
0
0
0
0
0
0
0
0
0
0
0
0
0
0


0
0
0
0
0
1
0
0
0
0
0
0
0
0
0
0
0
0
0
0
0
0
0
0
0
0
0
0
0
0
0
0
0
0
0
0
0
0
0
0
0
0
0
0
1
0
0
0
0
0
0
0
0
0
0
0
0
0
0
0
0
0
0
0
0
0
0
0
0
0
0
0
0
0
0
0
0
0
0
0
0


0
0
0
0
0
0
0
0
0
0
0
0
0
0
0
0
0
0
0
0
0
0
0
0
0
0
0
0
0
0
0
0
0
0
0
0
0
0
0
0
0
0
0
0
0
0
0
0
0
0
0
0
0
1
0
0
0
0
0
0
0
0
0
0
0
0
0
0
0
0
0
0
0
0
0
0
0
0
0
0
0


0
0
0
0
0
0
0
0
0
0
0
0
0
0
0
0
0
0
0
0
0
0
0
0
0
0
0
0
0
0
0
0
0
0
0
0
0
0
0
0
0
0
0
0
0
0
0
0
0
0
0
0
0
1
0
0
0
0
0
0
0
0
0
0
0
0
0
0
0
0
0
0
0
0
0
0
0
0
0
0
0


1
0
0
0
0
0
0
0
0
0
0
0
0
0
2
0
0
0
0
0
0
0
0
0
0
0
0
0
0
0
0
0
0
0
0
0
0
0
0
0
0
0
0
0
0
0
0
0
0
0
0
0
0
0
0
0
0
0
0
0
0
0
0
0
0
0
0
0
0
0
0
0
0
0
0
0
0
0
0
0
0


1
0
0
0
0
0
0
0
0
0
0
0
0
0
2
0
0
0
0
0
0
0
0
0
0
0
0
0
0
0
0
0
0
0
0
0
0
0
0
0
0
0
0
0
0
0
0
0
0
0
0
0
0
0
0
0
0
0
0
0
0
0
0
0
0
0
0
0
0
0
0
0
0
0
0
0
0
0
0
0
0


0
0
0
0
0
1
0
0
0
0
0
0
0
0
0
0
0
0
0
0
0
0
0
0
0
0
0
0
0
0
10
0
0
0
0
0
0
0
0
0
0
0
0
0
0
2
0
0
0
0
0
0
0
0
0
0
0
0
0
0
1
0
0
0
0
0
0
0
0
0
0
0
0
0
2
0
0
0
0
0
0


0
0
0
0
0
1
0
0
0
0
0
0
0
0
0
0
0
0
0
0
0
0
0
0
0
0
0
0
0
0
10
0
0
0
0
0
0
0
0
0
0
0
0
0
0
2
0
0
0
0
0
0
0
0
0
0
0
0
0
0
1
0
0
0
0
0
0
0
0
0
0
0
0
0
2
0
0
0
0
0
0


0
1
1
0
0
0
1
0
0
0
1
0
1
0
1
1
3
6
14
9
1
0
0
0
0
0
0
6
0
0
4
0
0
0
1
1
0
2
9
2
0
0
2
0
0
1
0
0
0
0
3
0
5
1
5
1
0
1
0
2
1
0
3
1
0
0
0
0
2
0
1
0
0
0
0
0
0
0
0
0
0


0
1
1
0
0
0
1
0
0
0
1
0
1
0
1
1
3
6
14
9
1
0
0
0
0
0
0
6
0
0
4
0
0
0
1
1
0
2
9
2
0
0
2
0
0
1
0
0
0
0
3
0
5
1
5
1
0
1
0
2
1
0
3
1
0
0
0
0
2
0
1
0
0
0
0
0
0
0
0
0
0


0
1
1
0
0
0
1
0
0
0
1
0
1
0
1
1
3
6
14
9
1
0
0
0
0
0
0
6
0
0
4
0
0
0
1
1
0
2
9
2
0
0
2
0
0
1
0
0
0
0
3
0
5
1
5
1
0
1
0
2
1
0
3
1
0
0
0
0
2
0
1
0
0
0
0
0
0
0
0
0
0


0
0
0
0
1
0
0
0
0
0
1
0
0
0
0
0
0
1
1
0
9
0
0
0
0
0
1
0
0
1
1
0
0
1
0
0
0
1
0
1
0
0
1
1
1
1
0
0
0
0
0
0
0
1
0
0
1
0
0
0
0
0
0
0
0
0
0
0
0
0
0
0
0
0
0
0
0
0
0
0
0


0
0
0
0
1
0
0
0
0
0
1
0
0
0
0
0
0
1
1
0
9
0
0
0
0
0
1
0
0
1
1
0
0
1
0
0
0
1
0
1
0
0
1
1
1
1
0
0
0
0
0
0
0
1
0
0
1
0
0
0
0
0
0
0
0
0
0
0
0
0
0
0
0
0
0
0
0
0
0
0
0


0
0
0
0
1
0
0
0
0
0
1
0
0
0
0
0
0
0
1
0
0
0
0
0
0
0
1
0
0
0
0
0
0
1
0
0
0
1
0
1
0
0
1
0
1
1
0
0
0
0
0
0
0
1
0
0
1
0
0
0
0
0
0
0
0
0
0
0
0
0
0
0
0
0
0
0
0
0
0
0
0


0
0
0
0
1
0
0
0
0
0
0
0
0
0
0
0
0
0
1
0
0
0
0
0
0
0
0
0
0
0
0
0
0
0
0
0
0
1
0
1
0
0
1
0
0
0
0
0
0
0
0
0
0
0
0
0
0
0
0
0
0
0
0
0
0
0
0
0
0
0
0
0
0
0
0
0
0
0
0
0
0


0
0
0
0
1
0
0
0
0
0
0
0
0
0
0
0
0
0
1
0
0
0
0
0
0
0
0
0
0
0
0
0
0
0
0
0
0
1
0
1
0
0
1
0
0
0
0
0
0
0
0
0
0
0
0
0
0
0
0
0
0
0
0
0
0
0
0
0
0
0
0
0
0
0
0
0
0
0
0
0
0


0
0
0
0
0
0
0
0
0
0
1
0
0
0
0
0
0
0
0
0
0
0
0
0
0
0
0
0
0
0
0
0
0
0
0
0
0
0
0
0
0
0
0
0
1
0
0
0
0
0
0
0
0
1
0
0
0
0
0
0
0
0
0
0
0
0
0
0
0
0
0
0
0
0
0
0
0
0
0
0
0


0
0
0
0
0
0
0
0
0
0
1
0
0
0
0
0
0
0
0
0
0
0
0
0
0
0
0
0
0
0
0
0
0
0
0
0
0
0
0
0
0
0
0
0
1
0
0
0
0
0
0
0
0
1
0
0
0
0
0
0
0
0
0
0
0
0
0
0
0
0
0
0
0
0
0
0
0
0
0
0
0


0
0
0
0
0
0
0
0
0
0
0
0
0
0
0
0
0
0
0
0
0
0
0
0
0
0
1
0
0
0
0
0
0
1
0
0
0
0
0
0
0
0
0
0
0
1
0
0
0
0
0
0
0
0
0
0
1
0
0
0
0
0
0
0
0
0
0
0
0
0
0
0
0
0
0
0
0
0
0
0
0


0
0
0
0
0
0
0
0
0
0
0
0
0
0
0
0
0
0
0
0
0
0
0
0
0
0
1
0
0
0
0
0
0
1
0
0
0
0
0
0
0
0
0
0
0
1
0
0
0
0
0
0
0
0
0
0
1
0
0
0
0
0
0
0
0
0
0
0
0
0
0
0
0
0
0
0
0
0
0
0
0


0
0
0
0
0
0
0
0
0
0
0
0
0
0
0
0
0
1
0
0
9
0
0
0
0
0
0
0
0
1
1
0
0
0
0
0
0
0
0
0
0
0
0
0
0
0
0
0
0
0
0
0
0
0
0
0
0
0
0
0
0
0
0
0
0
0
0
0
0
0
0
0
0
0
0
0
0
0
0
0
0


0
0
0
0
0
0
0
0
0
0
0
0
0
0
0
0
0
0
0
0
0
0
0
0
0
0
0
0
0
1
0
0
0
0
0
0
0
0
0
0
0
0
0
0
0
0
0
0
0
0
0
0
0
0
0
0
0
0
0
0
0
0
0
0
0
0
0
0
0
0
0
0
0
0
0
0
0
0
0
0
0


0
0
0
0
0
0
0
0
0
0
0
0
0
0
0
0
0
0
0
0
0
0
0
0
0
0
0
0
0
1
0
0
0
0
0
0
0
0
0
0
0
0
0
0
0
0
0
0
0
0
0
0
0
0
0
0
0
0
0
0
0
0
0
0
0
0
0
0
0
0
0
0
0
0
0
0
0
0
0
0
0


0
0
0
0
0
0
0
0
0
0
0
0
0
0
0
0
0
0
0
0
9
0
0
0
0
0
0
0
0
0
1
0
0
0
0
0
0
0
0
0
0
0
0
0
0
0
0
0
0
0
0
0
0
0
0
0
0
0
0
0
0
0
0
0
0
0
0
0
0
0
0
0
0
0
0
0
0
0
0
0
0


0
0
0
0
0
0
0
0
0
0
0
0
0
0
0
0
0
0
0
0
9
0
0
0
0
0
0
0
0
0
1
0
0
0
0
0
0
0
0
0
0
0
0
0
0
0
0
0
0
0
0
0
0
0
0
0
0
0
0
0
0
0
0
0
0
0
0
0
0
0
0
0
0
0
0
0
0
0
0
0
0


0
0
0
0
0
0
0
0
0
0
0
0
0
0
0
0
0
1
0
0
0
0
0
0
0
0
0
0
0
0
0
0
0
0
0
0
0
0
0
0
0
0
0
0
0
0
0
0
0
0
0
0
0
0
0
0
0
0
0
0
0
0
0
0
0
0
0
0
0
0
0
0
0
0
0
0
0
0
0
0
0


0
0
0
0
0
0
0
0
0
0
0
0
0
0
0
0
0
1
0
0
0
0
0
0
0
0
0
0
0
0
0
0
0
0
0
0
0
0
0
0
0
0
0
0
0
0
0
0
0
0
0
0
0
0
0
0
0
0
0
0
0
0
0
0
0
0
0
0
0
0
0
0
0
0
0
0
0
0
0
0
0


0
0
0
0
0
0
0
0
0
0
0
0
0
0
0
0
0
0
0
0
0
0
0
0
0
0
0
0
0
0
0
0
0
0
0
0
0
0
0
0
0
0
0
1
0
0
0
0
0
0
0
0
0
0
0
0
0
0
0
0
0
0
0
0
0
0
0
0
0
0
0
0
0
0
0
0
0
0
0
0
0


0
0
0
0
0
0
0
0
0
0
0
0
0
0
0
0
0
0
0
0
0
0
0
0
0
0
0
0
0
0
0
0
0
0
0
0
0
0
0
0
0
0
0
1
0
0
0
0
0
0
0
0
0
0
0
0
0
0
0
0
0
0
0
0
0
0
0
0
0
0
0
0
0
0
0
0
0
0
0
0
0


0
0
0
0
0
0
0
0
0
0
0
0
0
0
0
0
0
0
0
0
0
0
0
0
0
0
0
0
0
0
0
0
0
0
0
0
0
0
0
0
0
0
0
1
0
0
0
0
0
0
0
0
0
0
0
0
0
0
0
0
0
0
0
0
0
0
0
0
0
0
0
0
0
0
0
0
0
0
0
0
0


0
0
0
0
0
0
0
0
0
0
0
0
0
4
1
0
1
1
2
0
0
0
0
0
0
0
0
0
0
0
0
0
0
0
0
0
0
0
0
0
0
0
0
0
0
0
0
0
0
0
0
0
2
9
2
3
1
0
0
8
0
1
0
1
0
0
0
0
0
0
0
0
0
0
0
0
0
0
0
0
0


0
0
0
0
0
0
0
0
0
0
0
0
0
4
1
0
1
1
2
0
0
0
0
0
0
0
0
0
0
0
0
0
0
0
0
0
0
0
0
0
0
0
0
0
0
0
0
0
0
0
0
0
2
9
2
3
1
0
0
8
0
1
0
1
0
0
0
0
0
0
0
0
0
0
0
0
0
0
0
0
0


0
0
0
0
0
0
0
0
0
0
0
0
0
4
1
0
1
1
2
0
0
0
0
0
0
0
0
0
0
0
0
0
0
0
0
0
0
0
0
0
0
0
0
0
0
0
0
0
0
0
0
0
2
9
2
3
1
0
0
8
0
1
0
1
0
0
0
0
0
0
0
0
0
0
0
0
0
0
0
0
0


0
0
0
0
0
0
0
0
0
0
0
0
0
4
1
0
1
1
2
0
0
0
0
0
0
0
0
0
0
0
0
0
0
0
0
0
0
0
0
0
0
0
0
0
0
0
0
0
0
0
0
0
2
9
2
3
1
0
0
8
0
1
0
1
0
0
0
0
0
0
0
0
0
0
0
0
0
0
0
0
0


0
0
0
0
0
0
0
0
0
0
0
0
0
4
1
0
1
1
2
0
0
0
0
0
0
0
0
0
0
0
0
0
0
0
0
0
0
0
0
0
0
0
0
0
0
0
0
0
0
0
0
0
2
9
2
3
1
0
0
8
0
1
0
1
0
0
0
0
0
0
0
0
0
0
0
0
0
0
0
0
0


1425
2057
1733
1000
2135
1275
1683
794
1217
1219
1560
1469
1914
2158
1764
1580
1574
1942
1863
1313
2125
1677
1859
547
367
1514
173
720
702
891
1568
995
1271
73
506
947
397
983
1657
1770
895
1223
718
836
785
742
894
387
1334
152
1348
1062
1032
983
1943
1776
1555
1573
1455
937
2097
1780
1054
982
1235
1369
350
1205
631
422
1184
989
947
1680
383
446
228
363
1317
800
326


1424
2056
1733
999
2135
1275
1683
793
1217
1219
1560
1469
1914
2158
1763
1580
1574
1942
1863
1313
2125
1677
1859
547
367
1514
173
720
702
891
1568
995
1271
73
506
947
397
983
1657
1770
895
1223
718
836
784
742
894
387
1334
152
1347
1062
1032
983
1943
1776
1553
1573
1455
937
2097
1780
1054
982
1235
1369
350
1205
631
422
1184
989
947
1680
383
446
228
363
1317
800
326


7
25
16
1
2
3
5
8
7
7
13
14
8
15
13
12
7
15
13
7
4
3
9
0
0
0
0
4
0
1
1
0
0
0
8
1
0
3
13
0
1
6
0
4
0
1
0
0
9
0
30
3
3
8
7
11
12
8
9
12
11
26
11
14
8
17
0
17
3
5
24
8
10
21
2
0
2
1
17
6
2


7
25
16
1
2
3
5
8
7
7
13
14
8
15
13
12
7
15
13
7
4
3
9
0
0
0
0
4
0
1
1
0
0
0
8
1
0
3
13
0
1
6
0
4
0
1
0
0
9
0
30
3
3
8
7
11
12
8
9
12
11
26
11
14
8
17
0
17
3
5
24
8
10
21
2
0
2
1
17
6
2


7
25
16
1
2
3
5
8
7
7
13
14
8
15
13
12
7
15
13
7
4
3
9
0
0
0
0
4
0
1
1
0
0
0
8
1
0
3
13
0
1
6
0
4
0
1
0
0
9
0
30
3
3
8
7
11
12
8
9
12
11
26
11
14
8
17
0
17
3
5
24
8
10
21
2
0
2
1
17
6
2


1320
1892
1579
995
1994
1268
1449
705
1091
1145
1400
1322
1693
1985
1595
1471
1409
1692
1690
1197
2019
1566
1617
547
367
1470
172
627
701
882
1513
995
1270
73
486
936
394
812
1380
1717
744
1110
688
791
739
721
771
372
1225
151
1214
1049
990
939
1772
1680
1497
1535
1401
854
2006
1621
1039
948
1204
1258
350
931
615
417
1093
981
937
1641
375
445
226
359
1299
794
324


1095
1440
976
784
1552
723
1152
579
926
979
1183
1173
1418
1664
1353
1182
1209
1138
1270
948
1077
878
1189
358
315
737
0
249
189
604
813
938
782
61
362
495
167
396
876
642
495
768
432
387
356
454
320
194
829
0
1023
572
732
710
1338
1171
1149
992
1073
593
1598
1393
818
849
977
1014
237
792
432
277
827
732
658
1276
199
332
94
156
1043
639
226


1095
1440
976
784
1552
723
1152
579
926
979
1183
1173
1418
1664
1353
1182
1209
1138
1270
948
1077
878
1189
358
315
737
0
249
189
604
813
938
782
61
362
495
167
396
876
642
495
768
432
387
356
454
320
194
829
0
1023
572
732
710
1338
1171
1149
992
1073
593
1598
1393
818
849
977
1014
237
792
432
277
827
732
658
1276
199
332
94
156
1043
639
226


11
45
144
54
79
271
53
7
19
12
44
12
50
39
19
38
22
313
153
27
10
306
13
115
0
134
0
6
0
24
19
0
124
0
63
169
0
52
22
787
18
46
2
32
36
31
4
7
43
0
36
53
5
13
79
66
46
21
65
60
90
33
68
41
14
32
27
26
36
14
30
10
0
3
51
2
0
2
0
4
1


11
45
141
40
44
110
31
5
17
9
43
9
48
33
19
38
22
305
152
27
9
35
13
114
0
134
0
6
0
24
19
0
124
0
63
127
0
52
17
772
18
45
2
19
2
30
4
0
43
0
36
32
5
13
75
66
41
19
65
60
66
32
49
41
14
30
23
26
27
7
28
3
0
0
48
1
0
2
0
4
1


0
0
2
14
35
132
22
2
2
3
1
3
2
5
0
0
0
7
0
0
0
269
0
0
0
0
0
0
0
0
0
0
0
0
0
0
0
0
0
0
0
0
0
0
0
0
0
7
0
0
0
20
0
0
3
0
4
2
0
0
24
0
15
0
0
2
4
0
8
6
2
7
0
3
0
0
0
0
0
0
0


0
0
1
0
0
29
0
0
0
0
0
0
0
1
0
0
0
1
1
0
1
2
0
1
0
0
0
0
0
0
0
0
0
0
0
42
0
0
5
15
0
1
0
13
34
1
0
0
0
0
0
1
0
0
1
0
1
0
0
0
0
1
4
0
0
0
0
0
1
1
0
0
0
0
3
1
0
0
0
0
0


20
3
8
6
24
7
8
1
9
13
5
9
11
1
8
3
1
2
2
2
0
14
2
0
0
0
0
0
0
0
0
0
0
0
0
4
0
0
0
3
2
6
11
0
0
10
0
0
3
0
2
6
11
4
18
8
18
14
9
0
14
4
7
1
1
8
0
9
0
0
15
11
1
43
0
11
0
2
1
0
3


20
3
8
6
24
7
8
1
9
13
5
9
11
1
8
3
1
2
2
2
0
14
2
0
0
0
0
0
0
0
0
0
0
0
0
4
0
0
0
3
2
6
11
0
0
10
0
0
3
0
2
6
11
4
18
8
18
14
9
0
14
4
7
1
1
8
0
9
0
0
15
11
1
43
0
11
0
2
1
0
3


24
159
162
20
132
40
43
18
30
33
43
22
84
49
84
79
47
84
31
78
513
172
217
0
38
263
0
100
225
67
278
19
131
0
3
28
0
60
166
112
43
54
84
156
160
58
146
67
95
0
53
178
83
68
75
186
80
142
98
72
93
65
31
6
49
54
10
17
20
38
56
100
178
125
67
47
103
147
73
69
38


24
159
162
20
132
40
43
18
30
33
43
22
84
49
84
79
47
84
31
78
513
172
217
0
38
263
0
100
225
67
278
19
131
0
3
28
0
60
166
112
43
54
84
156
160
58
146
67
95
0
53
178
83
68
75
186
80
142
98
72
93
65
31
6
49
54
10
17
20
38
56
100
178
125
67
47
103
147
73
69
38


0
1
3
0
1
0
3
0
2
1
9
0
7
23
3
8
8
2
8
9
3
0
16
0
0
5
0
2
0
0
0
0
0
0
0
0
0
0
2
2
2
4
0
0
1
0
0
0
0
0
0
5
2
8
4
9
13
1
1
3
1
3
5
0
0
1
0
14
1
11
0
0
0
0
0
0
0
0
2
0
0


0
1
3
0
1
0
3
0
2
1
9
0
7
23
3
8
8
2
8
9
3
0
16
0
0
5
0
2
0
0
0
0
0
0
0
0
0
0
2
2
2
4
0
0
1
0
0
0
0
0
0
5
2
8
4
9
13
1
1
3
1
3
5
0
0
1
0
14
1
11
0
0
0
0
0
0
0
0
2
0
0


20
41
129
44
50
74
17
34
19
19
17
11
14
59
20
23
15
23
97
15
44
54
33
0
14
88
0
22
277
37
30
0
0
0
29
50
0
154
20
13
30
20
27
29
7
28
27
14
60
0
11
28
29
35
31
47
30
66
37
13
38
19
1
11
49
34
29
6
30
47
32
66
15
32
3
0
4
6
34
14
21


20
41
129
44
50
74
17
34
19
19
17
11
14
59
20
23
15
23
97
15
44
54
33
0
14
88
0
22
277
37
30
0
0
0
29
50
0
154
20
13
30
20
27
29
7
28
27
14
60
0
11
28
29
35
31
47
30
66
37
13
38
19
1
11
49
34
29
6
30
47
32
66
15
32
3
0
4
6
34
14
21


4
8
0
12
8
4
7
1
2
2
1
4
0
2
3
4
2
11
0
5
7
8
8
0
0
49
0
17
0
6
13
0
0
0
3
3
0
0
11
8
27
5
6
10
7
9
22
13
3
0
2
6
4
2
6
9
14
7
4
0
4
7
1
1
0
7
1
0
1
0
10
7
15
36
4
0
3
0
8
5
4


4
8
0
12
8
4
7
1
2
2
1
4
0
2
3
4
2
11
0
5
7
8
8
0
0
49
0
17
0
6
13
0
0
0
3
3
0
0
11
8
27
5
6
10
7
9
22
13
3
0
2
6
4
2
6
9
14
7
4
0
4
7
1
1
0
7
1
0
1
0
10
7
15
36
4
0
3
0
8
5
4


3
13
14
2
2
2
2
0
0
1
0
0
0
2
3
13
1
2
1
10
38
10
5
48
0
40
172
39
0
8
171
28
12
11
1
5
0
4
26
15
9
4
22
39
57
13
26
14
6
150
6
15
9
11
9
17
9
22
6
14
7
10
10
0
0
7
1
0
11
3
0
0
0
2
0
3
1
3
5
1
0


3
13
14
2
2
2
2
0
0
1
0
0
0
2
3
13
1
2
1
10
38
10
5
48
0
40
172
39
0
8
171
28
12
11
1
5
0
4
26
15
9
4
22
39
57
13
26
14
6
150
6
15
9
11
9
17
9
22
6
14
7
10
10
0
0
7
1
0
11
3
0
0
0
2
0
3
1
3
5
1
0


0
0
0
0
1
0
0
0
0
0
0
0
0
0
0
0
0
0
1
0
0
0
0
0
0
0
0
0
0
0
0
0
0
0
0
0
0
0
0
0
0
0
0
0
0
0
0
0
0
0
0
0
0
0
0
0
2
0
0
0
0
0
0
0
0
0
0
0
0
0
0
0
0
0
0
0
0
0
0
0
0


0
0
0
0
1
0
0
0
0
0
0
0
0
0
0
0
0
0
1
0
0
0
0
0
0
0
0
0
0
0
0
0
0
0
0
0
0
0
0
0
0
0
0
0
0
0
0
0
0
0
0
0
0
0
0
0
2
0
0
0
0
0
0
0
0
0
0
0
0
0
0
0
0
0
0
0
0
0
0
0
0


143
182
143
73
145
147
164
65
84
85
98
91
109
146
102
121
104
117
127
103
327
124
134
26
0
154
0
192
10
136
189
10
221
1
25
182
227
146
257
135
118
203
104
138
115
118
226
63
186
1
81
186
115
88
212
167
136
270
108
99
161
87
98
39
114
101
45
67
84
27
123
55
70
124
51
50
21
43
133
62
31


143
182
143
73
145
147
164
65
84
85
98
91
109
146
102
121
104
117
127
103
327
124
134
26
0
154
0
192
10
136
189
10
221
1
25
182
227
146
257
135
118
203
104
138
115
118
226
63
186
1
81
186
115
88
212
167
136
270
108
99
161
87
98
39
114
101
45
67
84
27
123
55
70
124
51
50
21
43
133
62
31


90
134
138
2
137
4
217
68
118
67
147
132
213
156
153
95
155
234
160
107
98
108
233
0
0
40
1
87
0
8
50
0
1
0
12
10
3
168
258
52
148
107
28
41
45
20
123
15
98
1
103
10
37
36
163
82
43
30
44
70
79
132
3
19
23
93
0
256
13
0
66
0
0
17
6
1
0
3
1
0
0


90
132
131
2
137
4
216
68
118
67
147
130
212
156
152
95
150
234
159
104
97
104
185
0
0
40
1
86
0
7
49
0
1
0
12
9
0
66
253
45
115
106
26
32
42
20
120
15
98
1
103
10
34
35
162
82
43
30
44
69
79
132
3
19
23
93
0
254
13
0
66
0
0
17
6
1
0
3
1
0
0


90
132
131
2
137
4
216
68
118
67
147
130
212
156
152
95
150
234
159
104
97
104
185
0
0
40
1
86
0
7
49
0
1
0
12
9
0
66
253
45
115
106
26
32
42
20
120
15
98
1
103
10
34
35
162
82
43
30
44
69
79
132
3
19
23
93
0
254
13
0
66
0
0
17
6
1
0
3
1
0
0


0
0
0
0
0
0
0
0
0
0
0
0
0
0
0
0
0
0
0
0
0
0
0
0
0
0
0
0
0
0
0
0
0
0
0
0
3
0
0
0
0
0
0
0
0
0
0
0
0
0
0
0
0
0
0
0
0
0
0
0
0
0
0
0
0
0
0
0
0
0
0
0
0
0
0
0
0
0
0
0
0


0
0
0
0
0
0
0
0
0
0
0
0
0
0
0
0
0
0
0
0
0
0
0
0
0
0
0
0
0
0
0
0
0
0
0
0
3
0
0
0
0
0
0
0
0
0
0
0
0
0
0
0
0
0
0
0
0
0
0
0
0
0
0
0
0
0
0
0
0
0
0
0
0
0
0
0
0
0
0
0
0


0
0
0
0
0
0
0
0
0
0
0
0
0
0
0
0
0
0
0
0
0
0
0
0
0
0
0
0
0
0
0
0
0
0
0
0
0
0
0
0
0
0
0
0
0
0
0
0
0
0
0
0
0
0
1
0
0
0
0
0
0
0
0
0
0
0
0
1
0
0
0
0
0
0
0
0
0
0
0
0
0


0
0
0
0
0
0
0
0
0
0
0
0
0
0
0
0
0
0
0
0
0
0
0
0
0
0
0
0
0
0
0
0
0
0
0
0
0
0
0
0
0
0
0
0
0
0
0
0
0
0
0
0
0
0
1
0
0
0
0
0
0
0
0
0
0
0
0
1
0
0
0
0
0
0
0
0
0
0
0
0
0


0
2
7
0
0
0
1
0
0
0
0
2
1
0
1
0
5
0
1
3
1
4
48
0
0
0
0
1
0
1
1
0
0
0
0
1
0
102
5
7
33
1
2
9
3
0
3
0
0
0
0
0
3
1
0
0
0
0
0
1
0
0
0
0
0
0
0
1
0
0
0
0
0
0
0
0
0
0
0
0
0


0
2
7
0
0
0
1
0
0
0
0
2
1
0
1
0
5
0
1
3
1
4
48
0
0
0
0
1
0
1
1
0
0
0
0
1
0
102
5
7
33
1
2
9
3
0
3
0
0
0
0
0
3
1
0
0
0
0
0
1
0
0
0
0
0
0
0
1
0
0
0
0
0
0
0
0
0
0
0
0
0


7
5
0
1
2
0
12
12
1
0
0
1
0
2
2
2
3
1
0
2
4
0
0
0
0
4
0
2
1
0
4
0
0
0
0
0
0
0
6
1
2
0
2
0
0
0
0
0
2
0
0
0
2
0
1
3
1
0
1
1
1
1
1
1
0
1
0
1
0
0
1
0
0
1
0
0
0
0
0
0
0


7
5
0
1
2
0
12
12
1
0
0
1
0
2
2
2
3
1
0
2
4
0
0
0
0
4
0
2
1
0
4
0
0
0
0
0
0
0
6
1
2
0
2
0
0
0
0
0
2
0
0
0
2
0
1
3
1
0
1
1
1
1
1
1
0
1
0
1
0
0
1
0
0
1
0
0
0
0
0
0
0


7
5
0
1
2
0
12
12
1
0
0
1
0
2
2
2
3
1
0
2
4
0
0
0
0
4
0
2
1
0
4
0
0
0
0
0
0
0
6
1
2
0
2
0
0
0
0
0
2
0
0
0
2
0
1
3
1
0
1
1
1
1
1
1
0
1
0
1
0
0
1
0
0
1
0
0
0
0
0
0
0


1
1
0
1
0
0
0
1
0
0
0
0
0
0
1
0
0
0
0
0
0
0
0
0
0
0
0
0
0
0
0
0
0
0
0
0
0
0
0
0
0
0
0
0
1
0
0
0
0
0
1
0
0
0
0
0
2
0
0
0
0
0
0
0
0
0
0
0
0
0
0
0
0
0
0
0
0
0
0
0
0


1
1
0
1
0
0
0
1
0
0
0
0
0
0
1
0
0
0
0
0
0
0
0
0
0
0
0
0
0
0
0
0
0
0
0
0
0
0
0
0
0
0
0
0
1
0
0
0
0
0
1
0
0
0
0
0
2
0
0
0
0
0
0
0
0
0
0
0
0
0
0
0
0
0
0
0
0
0
0
0
0


1
1
0
1
0
0
0
1
0
0
0
0
0
0
1
0
0
0
0
0
0
0
0
0
0
0
0
0
0
0
0
0
0
0
0
0
0
0
0
0
0
0
0
0
1
0
0
0
0
0
1
0
0
0
0
0
2
0
0
0
0
0
0
0
0
0
0
0
0
0
0
0
0
0
0
0
0
0
0
0
0


1
1
0
1
0
0
0
1
0
0
0
0
0
0
1
0
0
0
0
0
0
0
0
0
0
0
0
0
0
0
0
0
0
0
0
0
0
0
0
0
0
0
0
0
1
0
0
0
0
0
1
0
0
0
0
0
2
0
0
0
0
0
0
0
0
0
0
0
0
0
0
0
0
0
0
0
0
0
0
0
0


614
747
446
411
769
327
690
485
569
498
343
573
580
671
598
417
498
639
406
556
490
391
589
286
155
220
0
59
84
184
581
155
234
0
186
190
759
307
566
192
169
329
144
104
114
222
97
116
490
531
1049
266
545
388
778
693
328
370
401
361
696
1368
1094
438
267
639
107
424
333
180
659
428
302
465
124
149
126
148
311
266
131


614
747
446
411
769
327
690
485
569
498
343
573
580
671
598
417
498
639
406
556
490
391
589
286
155
220
0
59
84
184
581
155
234
0
186
190
759
307
566
192
169
329
144
104
114
222
97
116
490
531
1049
266
545
388
778
693
328
370
401
361
696
1368
1094
438
267
639
107
424
333
180
659
428
302
465
124
149
126
148
311
266
131


416
716
435
229
419
206
639
451
531
447
332
547
567
598
586
330
458
618
369
532
470
289
573
0
1
184
0
53
1
167
353
0
85
0
154
52
85
306
557
114
103
240
143
98
97
177
92
111
480
0
1028
213
448
383
755
620
301
355
375
324
653
1337
254
428
240
623
87
352
313
167
633
144
126
296
47
19
66
36
144
96
78


416
716
435
229
419
206
639
451
531
447
332
547
567
598
586
330
458
618
369
532
470
289
573
0
1
184
0
53
1
167
353
0
85
0
154
52
85
306
557
114
103
240
143
98
97
177
92
111
480
0
1028
213
448
383
755
620
301
355
375
324
653
1337
254
428
240
623
87
352
313
167
633
144
126
296
47
19
66
36
144
96
78


416
716
435
229
419
206
639
451
531
447
332
547
567
598
586
330
458
618
369
532
470
289
573
0
1
184
0
53
1
167
353
0
85
0
154
52
85
306
557
114
103
240
143
98
97
177
92
111
480
0
1028
213
448
383
755
620
301
355
375
324
653
1337
254
428
240
623
87
352
313
167
633
144
126
296
47
19
66
36
144
96
78


198
31
11
179
348
101
51
34
37
51
7
26
13
67
12
87
40
21
37
24
20
102
16
286
154
36
0
6
83
17
228
155
149
0
32
137
674
0
9
77
66
89
1
6
17
45
4
5
10
531
7
53
96
5
21
72
23
15
26
35
39
12
840
10
27
14
20
70
16
13
19
284
176
168
77
130
60
112
167
168
53


34
0
1
117
101
79
6
29
17
37
3
16
1
56
4
13
26
15
16
12
5
18
3
156
63
13
0
0
0
7
37
155
14
0
0
119
585
0
9
16
28
23
1
5
11
20
3
5
9
0
5
27
30
4
5
6
5
12
20
4
28
6
644
9
24
7
20
3
8
11
9
146
161
148
64
127
52
81
160
163
44


34
0
1
117
101
79
6
29
17
37
3
16
1
56
4
13
26
15
16
12
5
18
3
156
63
13
0
0
0
7
37
155
14
0
0
119
585
0
9
16
28
23
1
5
11
20
3
5
9
0
5
27
30
4
5
6
5
12
20
4
28
6
644
9
24
7
20
3
8
11
9
146
161
148
64
127
52
81
160
163
44


0
6
1
3
0
0
0
0
0
0
0
0
0
1
0
0
0
0
0
0
0
0
0
0
0
0
0
0
0
0
0
0
0
0
0
0
0
0
0
0
0
0
0
0
0
0
0
0
0
0
0
0
0
0
0
1
0
0
0
0
0
0
0
0
0
0
0
0
0
0
0
0
0
0
0
0
0
0
0
0
0


0
6
1
3
0
0
0
0
0
0
0
0
0
1
0
0
0
0
0
0
0
0
0
0
0
0
0
0
0
0
0
0
0
0
0
0
0
0
0
0
0
0
0
0
0
0
0
0
0
0
0
0
0
0
0
1
0
0
0
0
0
0
0
0
0
0
0
0
0
0
0
0
0
0
0
0
0
0
0
0
0


0
0
0
0
0
0
0
0
0
0
0
0
0
0
0
0
0
0
0
0
0
0
0
0
0
0
0
0
0
0
0
0
0
0
0
0
0
0
0
0
0
0
0
0
0
0
0
0
0
0
0
0
0
0
0
0
0
0
0
1
0
0
0
0
0
0
0
0
0
0
0
0
0
0
0
0
0
0
0
0
0


0
0
0
0
0
0
0
0
0
0
0
0
0
0
0
0
0
0
0
0
0
0
0
0
0
0
0
0
0
0
0
0
0
0
0
0
0
0
0
0
0
0
0
0
0
0
0
0
0
0
0
0
0
0
0
0
0
0
0
1
0
0
0
0
0
0
0
0
0
0
0
0
0
0
0
0
0
0
0
0
0


139
2
2
26
143
4
42
5
15
6
1
9
2
7
5
67
7
3
9
4
4
6
6
128
69
5
0
6
83
5
177
0
65
0
0
14
85
0
0
43
1
54
0
0
6
10
1
0
1
524
2
16
46
1
15
4
10
0
1
1
10
5
48
0
0
1
0
65
2
2
10
124
10
1
7
1
7
23
5
2
3


132
2
2
23
141
3
42
4
15
6
1
9
2
6
5
67
7
3
9
4
3
6
6
128
69
1
0
6
83
5
177
0
65
0
0
14
83
0
0
42
1
52
0
0
5
10
1
0
1
522
2
16
46
1
14
3
10
0
0
1
9
5
45
0
0
1
0
65
2
2
9
122
7
1
7
1
5
22
5
2
3


5
0
0
1
0
0
0
1
0
0
0
0
0
0
0
0
0
0
0
0
0
0
0
0
0
0
0
0
0
0
0
0
0
0
0
0
0
0
0
0
0
0
0
0
1
0
0
0
0
0
0
0
0
0
0
0
0
0
1
0
0
0
0
0
0
0
0
0
0
0
0
0
3
0
0
0
2
0
0
0
0


2
0
0
2
2
1
0
0
0
0
0
0
0
1
0
0
0
0
0
0
1
0
0
0
0
4
0
0
0
0
0
0
0
0
0
0
2
0
0
1
0
2
0
0
0
0
0
0
0
2
0
0
0
0
1
1
0
0
0
0
1
0
3
0
0
0
0
0
0
0
1
2
0
0
0
0
0
1
0
0
0


7
14
0
0
42
0
3
0
0
1
0
1
8
2
0
2
2
1
0
0
8
73
0
0
18
4
0
0
0
1
0
0
69
0
0
0
0
0
0
15
0
7
0
1
0
0
0
0
0
0
0
2
1
0
1
34
4
1
0
9
0
0
61
1
0
0
0
0
2
0
0
2
2
0
0
0
0
3
0
0
0


7
14
0
0
38
0
1
0
0
1
0
0
0
1
0
1
1
1
0
0
0
1
0
0
0
0
0
0
0
1
0
0
0
0
0
0
0
0
0
13
0
7
0
0
0
0
0
0
0
0
0
2
1
0
0
30
0
1
0
9
0
0
2
1
0
0
0
0
2
0
0
2
0
0
0
0
0
1
0
0
0


0
0
0
0
4
0
2
0
0
0
0
1
8
1
0
1
1
0
0
0
8
71
0
0
18
4
0
0
0
0
0
0
69
0
0
0
0
0
0
2
0
0
0
1
0
0
0
0
0
0
0
0
0
0
1
4
4
0
0
0
0
0
59
0
0
0
0
0
0
0
0
0
2
0
0
0
0
2
0
0
0


0
0
0
0
0
0
0
0
0
0
0
0
0
0
0
0
0
0
0
0
0
1
0
0
0
0
0
0
0
0
0
0
0
0
0
0
0
0
0
0
0
0
0
0
0
0
0
0
0
0
0
0
0
0
0
0
0
0
0
0
0
0
0
0
0
0
0
0
0
0
0
0
0
0
0
0
0
0
0
0
0


18
9
7
33
62
18
0
0
5
7
3
0
2
1
3
5
5
2
12
8
3
5
7
2
4
14
0
0
0
4
14
0
1
0
32
4
4
0
0
3
37
5
0
0
0
15
0
0
0
7
0
8
19
0
0
27
4
2
5
20
1
1
87
0
3
6
0
2
4
0
0
12
3
19
6
2
1
5
2
3
6


18
9
7
33
62
18
0
0
5
7
3
0
2
1
3
5
5
2
12
8
3
5
7
2
4
14
0
0
0
4
14
0
1
0
32
4
4
0
0
3
37
5
0
0
0
15
0
0
0
7
0
8
19
0
0
27
4
2
5
20
1
1
87
0
3
6
0
2
4
0
0
12
3
19
6
2
1
5
2
3
6


0
0
0
3
2
20
0
0
1
0
4
0
0
6
0
0
0
0
0
0
0
0
0
0
0
0
0
0
0
0
0
0
0
0
0
1
0
1
0
1
0
0
0
0
0
0
1
0
0
0
14
0
1
0
2
1
4
0
0
2
4
19
0
0
0
2
0
2
4
0
7
0
0
1
0
0
0
0
0
2
0


0
0
0
3
2
20
0
0
1
0
4
0
0
6
0
0
0
0
0
0
0
0
0
0
0
0
0
0
0
0
0
0
0
0
0
1
0
1
0
1
0
0
0
0
0
0
1
0
0
0
14
0
1
0
2
1
4
0
0
2
4
19
0
0
0
2
0
2
4
0
7
0
0
1
0
0
0
0
0
2
0


0
0
0
3
2
20
0
0
1
0
4
0
0
6
0
0
0
0
0
0
0
0
0
0
0
0
0
0
0
0
0
0
0
0
0
1
0
1
0
1
0
0
0
0
0
0
1
0
0
0
14
0
1
0
2
1
4
0
0
2
4
19
0
0
0
2
0
2
4
0
7
0
0
1
0
0
0
0
0
2
0


3
23
8
0
5
0
23
3
18
0
11
11
4
2
28
7
5
24
16
5
3
1
7
0
0
0
0
0
0
0
0
0
0
0
0
1
0
0
7
2
0
11
0
0
0
0
2
0
1
0
9
0
0
6
15
5
0
4
6
2
9
9
0
1
1
8
1
3
1
0
6
0
0
0
7
0
0
0
0
2
0


3
23
8
0
5
0
23
3
18
0
11
11
4
2
28
7
5
24
16
5
3
1
7
0
0
0
0
0
0
0
0
0
0
0
0
1
0
0
7
2
0
11
0
0
0
0
2
0
1
0
9
0
0
6
15
5
0
4
6
2
9
9
0
1
1
8
1
3
1
0
6
0
0
0
7
0
0
0
0
2
0


3
23
8
0
5
0
23
3
18
0
11
11
4
2
28
7
5
24
16
5
3
1
7
0
0
0
0
0
0
0
0
0
0
0
0
1
0
0
7
2
0
11
0
0
0
0
2
0
1
0
9
0
0
6
15
5
0
4
6
2
9
9
0
1
1
8
1
3
1
0
6
0
0
0
7
0
0
0
0
2
0


3
23
8
0
5
0
23
3
18
0
11
11
4
2
28
7
5
24
16
5
3
1
7
0
0
0
0
0
0
0
0
0
0
0
0
1
0
0
7
2
0
11
0
0
0
0
2
0
1
0
9
0
0
6
15
5
0
4
6
2
9
9
0
1
1
8
1
3
1
0
6
0
0
0
7
0
0
0
0
2
0


3
23
8
0
5
0
23
3
18
0
11
11
4
2
28
7
5
24
16
5
3
1
7
0
0
0
0
0
0
0
0
0
0
0
0
1
0
0
7
2
0
11
0
0
0
0
2
0
1
0
9
0
0
6
15
5
0
4
6
2
9
9
0
1
1
8
1
3
1
0
6
0
0
0
7
0
0
0
0
2
0


136
167
57
58
70
29
104
69
85
108
81
133
64
148
130
123
98
165
94
92
169
66
111
0
0
53
1
70
1
44
113
0
19
0
20
31
0
182
197
57
65
104
50
46
57
55
113
24
90
0
307
50
53
99
100
97
119
108
69
127
171
398
147
136
140
124
45
145
82
27
123
48
73
81
89
40
91
60
44
46
33


136
167
57
58
70
29
104
69
85
108
81
133
64
148
130
123
98
165
94
92
169
66
111
0
0
53
1
70
1
44
113
0
19
0
20
31
0
182
197
57
65
104
50
46
57
55
113
24
90
0
307
50
53
99
100
97
119
108
69
127
171
398
147
136
140
124
45
145
82
27
123
48
73
81
89
40
91
60
44
46
33


136
167
57
58
70
29
104
69
85
108
81
133
64
148
130
123
98
165
94
92
169
66
111
0
0
53
1
70
1
44
113
0
19
0
20
31
0
182
197
57
65
104
50
46
57
55
113
24
90
0
307
50
53
99
100
97
119
108
69
127
171
398
147
136
140
124
45
145
82
27
123
48
73
81
89
40
91
60
44
46
33


136
167
57
58
70
29
104
69
85
108
81
133
64
148
130
123
98
165
94
92
169
66
111
0
0
53
1
70
1
44
113
0
19
0
20
31
0
182
197
57
65
104
50
46
57
55
113
24
90
0
307
50
53
99
100
97
119
108
69
127
171
398
147
136
140
124
45
145
82
27
123
48
73
81
89
40
91
60
44
46
33


136
167
57
58
70
29
104
69
85
108
81
133
64
148
130
123
98
165
94
92
169
66
111
0
0
53
1
70
1
44
113
0
19
0
20
31
0
182
197
57
65
104
50
46
57
55
113
24
90
0
307
50
53
99
100
97
119
108
69
127
171
398
147
136
140
124
45
145
82
27
123
48
73
81
89
40
91
60
44
46
33


74
39
16
54
34
46
44
33
66
30
42
45
32
76
26
26
39
46
62
53
15
25
30
0
31
10
0
9
149
11
36
0
0
9
21
20
0
25
20
16
12
41
15
21
54
42
20
7
28
0
54
27
39
29
55
55
36
45
31
22
87
94
89
63
79
41
57
84
46
76
49
75
61
35
10
37
41
42
25
30
11


74
39
16
54
34
46
44
33
66
30
42
45
32
76
26
26
39
46
62
53
15
25
30
0
31
10
0
9
149
11
36
0
0
9
21
20
0
25
20
16
12
41
15
21
54
42
20
7
28
0
54
27
39
29
55
55
36
45
31
22
87
94
89
63
79
41
57
84
46
76
49
75
61
35
10
37
41
42
25
30
11


4
0
0
0
0
0
0
2
1
0
0
1
0
0
1
0
1
0
0
0
0
1
2
0
0
0
0
1
0
0
0
0
0
0
0
1
0
0
0
0
0
0
0
0
0
1
0
0
3
0
1
0
4
0
10
1
0
2
5
0
6
4
0
4
2
2
4
2
2
2
4
1
0
2
0
6
2
2
0
1
0


4
0
0
0
0
0
0
2
1
0
0
1
0
0
1
0
1
0
0
0
0
1
2
0
0
0
0
1
0
0
0
0
0
0
0
1
0
0
0
0
0
0
0
0
0
1
0
0
3
0
1
0
4
0
10
1
0
2
5
0
6
4
0
4
2
2
4
2
2
2
4
1
0
2
0
6
2
2
0
1
0


4
0
0
0
0
0
0
2
1
0
0
1
0
0
1
0
1
0
0
0
0
1
2
0
0
0
0
1
0
0
0
0
0
0
0
1
0
0
0
0
0
0
0
0
0
1
0
0
3
0
1
0
4
0
10
1
0
2
5
0
6
4
0
4
2
2
4
2
2
2
4
1
0
2
0
6
2
2
0
1
0


4
0
0
0
0
0
0
2
1
0
0
1
0
0
1
0
1
0
0
0
0
1
2
0
0
0
0
1
0
0
0
0
0
0
0
1
0
0
0
0
0
0
0
0
0
1
0
0
3
0
1
0
4
0
10
1
0
2
5
0
6
4
0
4
2
2
4
2
2
2
4
1
0
2
0
6
2
2
0
1
0


60
39
16
51
31
37
40
23
58
30
42
42
32
75
24
26
37
46
62
52
15
22
28
0
31
10
0
8
149
11
18
0
0
9
21
15
0
25
20
14
12
40
15
21
40
32
20
7
24
0
51
19
29
29
45
53
35
42
22
21
78
84
80
55
71
37
45
82
42
58
43
66
56
31
8
31
36
39
20
23
10


18
4
4
13
15
5
20
4
11
17
2
5
1
19
3
3
1
4
13
9
0
2
7
0
0
1
0
2
0
0
0
0
0
0
0
2
0
3
5
0
0
3
0
0
1
3
4
0
0
0
4
3
5
2
4
5
2
2
3
5
11
8
11
4
5
4
6
4
8
4
4
3
0
1
5
4
11
8
6
0
1


18
4
4
13
15
5
20
4
11
17
2
5
1
19
3
3
1
4
13
9
0
2
7
0
0
1
0
2
0
0
0
0
0
0
0
2
0
3
5
0
0
3
0
0
1
3
4
0
0
0
4
3
5
2
4
5
2
2
3
5
11
8
11
4
5
4
6
4
8
4
4
3
0
1
5
4
11
8
6
0
1


18
4
4
13
15
5
20
4
11
17
2
5
1
19
3
3
1
4
13
9
0
2
7
0
0
1
0
2
0
0
0
0
0
0
0
2
0
3
5
0
0
3
0
0
1
3
4
0
0
0
4
3
5
2
4
5
2
2
3
5
11
8
11
4
5
4
6
4
8
4
4
3
0
1
5
4
11
8
6
0
1


0
0
0
1
0
0
0
0
0
0
0
0
0
0
0
0
0
0
0
0
0
0
0
0
0
0
0
0
0
0
0
0
0
0
0
0
0
0
0
0
0
0
0
0
0
0
0
0
0
0
0
0
0
0
0
0
0
0
0
0
0
0
0
0
1
0
0
0
0
0
0
0
0
0
0
0
0
0
0
0
0


0
0
0
1
0
0
0
0
0
0
0
0
0
0
0
0
0
0
0
0
0
0
0
0
0
0
0
0
0
0
0
0
0
0
0
0
0
0
0
0
0
0
0
0
0
0
0
0
0
0
0
0
0
0
0
0
0
0
0
0
0
0
0
0
1
0
0
0
0
0
0
0
0
0
0
0
0
0
0
0
0


0
0
0
1
0
0
0
0
0
0
0
0
0
0
0
0
0
0
0
0
0
0
0
0
0
0
0
0
0
0
0
0
0
0
0
0
0
0
0
0
0
0
0
0
0
0
0
0
0
0
0
0
0
0
0
0
0
0
0
0
0
0
0
0
1
0
0
0
0
0
0
0
0
0
0
0
0
0
0
0
0


0
1
0
0
0
0
2
0
1
0
2
0
2
0
0
0
1
0
0
1
0
2
1
0
0
0
0
0
0
0
0
0
0
0
0
0
0
0
0
1
0
0
0
0
0
0
0
0
0
0
1
0
1
2
0
2
1
1
0
5
3
1
5
1
0
2
0
5
2
0
0
2
3
0
0
0
0
0
0
0
0


0
1
0
0
0
0
2
0
1
0
2
0
2
0
0
0
1
0
0
1
0
2
1
0
0
0
0
0
0
0
0
0
0
0
0
0
0
0
0
1
0
0
0
0
0
0
0
0
0
0
1
0
1
2
0
2
1
1
0
5
3
1
5
1
0
2
0
5
2
0
0
2
3
0
0
0
0
0
0
0
0


0
1
0
0
0
0
2
0
1
0
2
0
2
0
0
0
1
0
0
1
0
2
1
0
0
0
0
0
0
0
0
0
0
0
0
0
0
0
0
1
0
0
0
0
0
0
0
0
0
0
1
0
1
2
0
2
1
1
0
5
3
1
5
1
0
2
0
5
2
0
0
2
3
0
0
0
0
0
0
0
0


15
21
9
7
8
5
7
3
15
4
21
13
17
22
11
10
6
17
17
10
2
11
12
0
31
2
0
5
149
6
5
0
0
0
5
9
0
8
7
10
6
14
3
3
12
15
12
3
11
0
6
10
7
11
24
15
3
18
8
6
24
20
12
18
25
17
10
22
8
19
12
12
18
14
0
9
13
17
11
5
3


2
2
0
2
0
0
0
0
0
1
0
0
0
0
1
0
0
4
1
0
1
1
0
0
0
0
0
1
0
0
1
0
0
0
0
2
0
0
1
0
3
3
0
0
4
2
0
0
4
0
0
1
3
1
5
8
1
3
2
1
3
3
0
4
6
1
1
3
2
3
2
0
1
1
0
1
5
4
0
1
0


2
2
0
2
0
0
0
0
0
1
0
0
0
0
1
0
0
4
1
0
1
1
0
0
0
0
0
1
0
0
1
0
0
0
0
2
0
0
1
0
3
3
0
0
4
2
0
0
4
0
0
1
3
1
5
8
1
3
2
1
3
3
0
4
6
1
1
3
2
3
2
0
1
1
0
1
5
4
0
1
0


0
7
3
3
3
2
2
2
2
0
4
0
1
0
2
0
2
1
0
0
0
1
1
0
0
2
0
2
0
3
1
0
0
0
0
1
0
0
4
8
0
0
0
0
1
0
1
2
3
0
2
0
1
3
3
2
0
5
1
2
7
4
2
3
2
4
4
4
0
8
3
7
3
2
0
2
6
6
1
2
1


0
7
3
3
3
2
2
2
2
0
4
0
1
0
2
0
2
1
0
0
0
1
1
0
0
2
0
2
0
3
1
0
0
0
0
1
0
0
4
8
0
0
0
0
1
0
1
2
3
0
2
0
1
3
3
2
0
5
1
2
7
4
2
3
2
4
4
4
0
8
3
7
3
2
0
2
6
6
1
2
1


1
0
0
0
0
0
0
0
0
1
0
2
0
1
0
0
2
1
0
0
0
1
0
0
0
0
0
0
0
0
0
0
0
0
0
1
0
0
1
0
0
0
0
0
0
1
0
0
0
0
1
0
0
0
0
0
0
0
0
0
0
0
0
0
0
0
0
0
0
0
0
0
0
0
0
0
0
0
0
0
0


1
0
0
0
0
0
0
0
0
0
0
0
0
0
0
0
2
0
0
0
0
1
0
0
0
0
0
0
0
0
0
0
0
0
0
0
0
0
0
0
0
0
0
0
0
0
0
0
0
0
0
0
0
0
0
0
0
0
0
0
0
0
0
0
0
0
0
0
0
0
0
0
0
0
0
0
0
0
0
0
0


0
0
0
0
0
0
0
0
0
1
0
2
0
1
0
0
0
1
0
0
0
0
0
0
0
0
0
0
0
0
0
0
0
0
0
1
0
0
1
0
0
0
0
0
0
1
0
0
0
0
1
0
0
0
0
0
0
0
0
0
0
0
0
0
0
0
0
0
0
0
0
0
0
0
0
0
0
0
0
0
0


12
12
6
2
5
3
5
1
13
2
17
11
16
21
8
10
2
11
16
10
1
8
11
0
31
0
0
2
149
3
3
0
0
0
5
5
0
8
1
2
3
11
3
3
7
12
11
1
4
0
3
9
3
7
16
5
2
10
5
3
14
13
10
11
17
12
5
15
6
8
7
5
14
11
0
6
2
7
10
2
2


12
12
6
2
5
3
5
1
13
2
17
11
16
21
8
10
2
11
16
10
1
8
11
0
31
0
0
2
149
3
3
0
0
0
5
5
0
8
1
2
3
11
3
3
7
12
11
1
4
0
3
9
3
7
16
5
2
10
5
3
14
13
10
11
17
12
5
15
6
8
7
5
14
11
0
6
2
7
10
2
2


5
2
1
11
1
1
0
2
6
1
6
4
2
2
0
2
2
0
2
2
2
0
4
0
0
0
0
0
0
0
3
0
0
0
2
0
0
4
0
0
1
2
0
1
1
1
0
0
0
0
1
2
7
9
3
12
15
2
2
0
12
9
0
2
1
2
17
9
4
6
3
33
6
5
0
16
0
0
0
15
1


5
2
1
11
1
1
0
2
6
1
6
4
2
2
0
2
2
0
2
2
2
0
4
0
0
0
0
0
0
0
3
0
0
0
2
0
0
4
0
0
1
2
0
1
1
1
0
0
0
0
1
2
7
9
3
12
15
2
2
0
12
9
0
2
1
2
17
9
4
6
3
33
6
5
0
16
0
0
0
15
1


5
2
1
11
1
1
0
2
6
1
6
4
2
2
0
2
2
0
2
2
2
0
4
0
0
0
0
0
0
0
3
0
0
0
2
0
0
4
0
0
1
2
0
1
1
1
0
0
0
0
1
2
7
9
3
12
15
2
2
0
12
9
0
2
1
2
17
9
4
6
3
33
6
5
0
16
0
0
0
15
1


0
0
0
0
0
0
0
0
0
0
0
0
0
0
0
0
0
0
0
0
0
0
0
0
0
0
0
0
0
0
0
0
0
0
0
1
0
0
0
0
0
0
0
0
0
0
0
0
0
0
0
0
0
0
0
0
0
0
0
0
0
0
0
0
0
0
0
0
0
0
0
0
0
0
0
0
0
0
0
0
0


0
0
0
0
0
0
0
0
0
0
0
0
0
0
0
0
0
0
0
0
0
0
0
0
0
0
0
0
0
0
0
0
0
0
0
1
0
0
0
0
0
0
0
0
0
0
0
0
0
0
0
0
0
0
0
0
0
0
0
0
0
0
0
0
0
0
0
0
0
0
0
0
0
0
0
0
0
0
0
0
0


0
0
0
0
0
0
0
0
0
0
0
0
0
0
0
0
0
0
0
0
0
0
0
0
0
0
0
0
0
0
0
0
0
0
0
1
0
0
0
0
0
0
0
0
0
0
0
0
0
0
0
0
0
0
0
0
0
0
0
0
0
0
0
0
0
0
0
0
0
0
0
0
0
0
0
0
0
0
0
0
0


0
0
0
0
0
0
0
0
0
0
0
0
0
0
0
0
0
0
0
1
0
0
0
0
0
0
0
0
0
0
0
0
0
0
0
0
0
0
0
0
0
0
0
0
0
0
0
0
1
0
0
0
0
0
0
0
0
2
0
0
0
0
0
0
2
0
0
0
0
0
0
0
2
1
0
0
0
0
0
0
0


0
0
0
0
0
0
0
0
0
0
0
0
0
0
0
0
0
0
0
1
0
0
0
0
0
0
0
0
0
0
0
0
0
0
0
0
0
0
0
0
0
0
0
0
0
0
0
0
1
0
0
0
0
0
0
0
0
1
0
0
0
0
0
0
0
0
0
0
0
0
0
0
2
1
0
0
0
0
0
0
0


0
0
0
0
0
0
0
0
0
0
0
0
0
0
0
0
0
0
0
1
0
0
0
0
0
0
0
0
0
0
0
0
0
0
0
0
0
0
0
0
0
0
0
0
0
0
0
0
1
0
0
0
0
0
0
0
0
1
0
0
0
0
0
0
0
0
0
0
0
0
0
0
2
1
0
0
0
0
0
0
0


0
0
0
0
0
0
0
0
0
0
0
0
0
0
0
0
0
0
0
0
0
0
0
0
0
0
0
0
0
0
0
0
0
0
0
0
0
0
0
0
0
0
0
0
0
0
0
0
0
0
0
0
0
0
0
0
0
1
0
0
0
0
0
0
2
0
0
0
0
0
0
0
0
0
0
0
0
0
0
0
0


0
0
0
0
0
0
0
0
0
0
0
0
0
0
0
0
0
0
0
0
0
0
0
0
0
0
0
0
0
0
0
0
0
0
0
0
0
0
0
0
0
0
0
0
0
0
0
0
0
0
0
0
0
0
0
0
0
1
0
0
0
0
0
0
2
0
0
0
0
0
0
0
0
0
0
0
0
0
0
0
0


22
11
2
19
7
26
11
14
25
8
11
20
10
32
10
11
27
25
30
29
11
7
4
0
0
7
0
1
0
5
10
0
0
9
14
3
0
10
8
3
5
21
12
17
26
13
4
4
12
0
39
4
9
5
14
19
14
17
9
5
28
46
52
30
37
12
12
42
20
29
24
16
27
10
3
2
12
14
3
3
5


22
11
2
19
7
26
11
14
25
8
11
20
10
32
10
11
27
25
30
29
11
7
4
0
0
7
0
1
0
5
10
0
0
9
14
3
0
10
8
3
5
21
12
17
26
13
4
4
12
0
39
4
9
5
14
19
14
17
9
5
28
46
52
30
37
12
12
42
20
29
24
16
27
10
3
2
12
14
3
3
5


22
11
2
19
7
26
11
14
25
8
11
20
10
32
10
11
27
25
30
29
11
7
4
0
0
7
0
1
0
5
10
0
0
9
14
3
0
10
8
3
5
21
12
17
26
13
4
4
12
0
39
4
9
5
14
19
14
17
9
5
28
46
52
30
37
12
12
42
20
29
24
16
27
10
3
2
12
14
3
3
5


10
0
0
3
3
9
4
8
7
0
0
2
0
1
1
0
1
0
0
1
0
2
0
0
0
0
0
0
0
0
18
0
0
0
0
4
0
0
0
2
0
1
0
0
14
9
0
0
1
0
2
8
6
0
0
1
1
1
4
1
3
6
9
4
6
2
8
0
2
16
2
8
5
2
2
0
3
1
5
6
1


10
0
0
3
3
9
4
8
7
0
0
2
0
1
1
0
1
0
0
1
0
2
0
0
0
0
0
0
0
0
18
0
0
0
0
4
0
0
0
2
0
1
0
0
14
9
0
0
1
0
2
8
6
0
0
1
1
1
4
1
3
6
9
4
6
2
8
0
2
16
2
8
5
2
2
0
3
1
5
6
1


10
0
0
3
3
9
4
8
7
0
0
2
0
1
1
0
1
0
0
1
0
2
0
0
0
0
0
0
0
0
18
0
0
0
0
4
0
0
0
2
0
1
0
0
14
9
0
0
1
0
2
8
6
0
0
1
1
1
4
1
3
6
9
4
6
2
8
0
2
16
2
8
5
2
2
0
3
1
5
6
1


10
0
0
3
3
9
4
8
7
0
0
2
0
1
1
0
1
0
0
1
0
2
0
0
0
0
0
0
0
0
18
0
0
0
0
4
0
0
0
2
0
1
0
0
14
9
0
0
1
0
2
8
6
0
0
1
1
1
4
1
3
6
9
4
6
2
8
0
2
16
2
8
5
2
2
0
3
1
5
6
1


43
125
68
80
22
83
151
170
49
35
17
36
15
94
63
37
35
52
49
66
124
48
41
0
0
16
0
20
0
46
72
0
0
0
17
15
0
107
82
23
33
46
46
16
21
42
63
34
60
0
85
30
46
43
87
81
83
49
56
52
96
118
55
66
88
62
78
63
55
49
29
63
61
79
47
27
45
67
123
29
67


19
47
61
64
18
80
23
33
27
27
12
31
9
57
41
10
11
23
26
44
28
26
23
0
0
7
0
7
0
31
35
0
0
0
11
15
0
93
45
13
9
25
17
9
8
30
41
28
42
0
25
23
33
23
20
29
19
29
18
14
32
42
50
19
22
26
47
18
19
30
8
63
61
77
43
25
45
65
122
25
67


19
47
61
64
18
80
23
33
27
27
12
31
9
57
41
10
11
23
26
44
28
26
23
0
0
7
0
7
0
31
35
0
0
0
11
15
0
93
45
13
9
25
17
9
8
30
41
28
42
0
25
23
33
23
20
29
19
29
18
14
32
42
50
19
22
26
47
18
19
30
8
63
61
77
43
25
45
65
122
25
67


19
47
61
64
18
80
23
33
27
27
12
31
9
57
41
10
11
23
26
44
28
26
23
0
0
7
0
7
0
31
35
0
0
0
11
15
0
93
45
13
9
25
17
9
8
30
41
28
42
0
25
23
33
23
20
29
19
29
18
14
32
42
50
19
22
26
47
18
19
30
8
63
61
77
43
25
45
65
122
25
67


19
47
61
64
18
80
23
33
27
27
12
31
9
57
41
10
11
23
26
44
28
26
23
0
0
7
0
7
0
31
35
0
0
0
11
15
0
93
45
13
9
25
17
9
8
30
41
28
42
0
25
23
33
23
20
29
19
29
18
14
32
42
50
19
22
26
47
18
19
30
8
63
61
77
43
25
45
65
122
25
67


19
47
61
64
18
80
23
33
27
27
12
31
9
57
41
10
11
23
26
44
28
26
23
0
0
7
0
7
0
31
35
0
0
0
11
15
0
93
45
13
9
25
17
9
8
30
41
28
42
0
25
23
33
23
20
29
19
29
18
14
32
42
50
19
22
26
47
18
19
30
8
63
61
77
43
25
45
65
122
25
67


8
0
0
13
1
0
105
38
4
4
0
2
1
3
1
0
1
0
3
0
0
0
0
0
0
0
0
0
0
0
0
0
0
0
0
0
0
0
3
0
0
3
0
3
2
0
0
0
3
0
7
0
3
9
11
16
0
14
7
3
18
16
0
22
5
5
25
3
26
0
13
0
0
0
0
0
0
0
1
3
0


8
0
0
13
1
0
60
38
4
4
0
2
1
3
1
0
1
0
3
0
0
0
0
0
0
0
0
0
0
0
0
0
0
0
0
0
0
0
3
0
0
3
0
3
2
0
0
0
3
0
7
0
3
3
11
16
0
14
7
3
18
16
0
22
5
5
25
3
26
0
13
0
0
0
0
0
0
0
1
3
0


8
0
0
13
1
0
60
38
4
4
0
2
1
3
1
0
1
0
3
0
0
0
0
0
0
0
0
0
0
0
0
0
0
0
0
0
0
0
3
0
0
3
0
3
2
0
0
0
3
0
7
0
3
3
11
16
0
14
7
3
18
16
0
22
5
5
25
3
26
0
13
0
0
0
0
0
0
0
1
3
0


8
0
0
13
1
0
60
38
4
4
0
2
1
3
1
0
1
0
3
0
0
0
0
0
0
0
0
0
0
0
0
0
0
0
0
0
0
0
3
0
0
3
0
3
2
0
0
0
3
0
7
0
3
3
11
16
0
14
7
3
18
16
0
22
5
5
25
3
26
0
13
0
0
0
0
0
0
0
1
3
0


8
0
0
13
1
0
60
38
4
4
0
2
1
3
1
0
1
0
3
0
0
0
0
0
0
0
0
0
0
0
0
0
0
0
0
0
0
0
3
0
0
3
0
3
2
0
0
0
3
0
7
0
3
3
11
16
0
14
7
3
18
16
0
22
5
5
25
3
26
0
13
0
0
0
0
0
0
0
1
3
0


0
0
0
0
0
0
45
0
0
0
0
0
0
0
0
0
0
0
0
0
0
0
0
0
0
0
0
0
0
0
0
0
0
0
0
0
0
0
0
0
0
0
0
0
0
0
0
0
0
0
0
0
0
6
0
0
0
0
0
0
0
0
0
0
0
0
0
0
0
0
0
0
0
0
0
0
0
0
0
0
0


0
0
0
0
0
0
45
0
0
0
0
0
0
0
0
0
0
0
0
0
0
0
0
0
0
0
0
0
0
0
0
0
0
0
0
0
0
0
0
0
0
0
0
0
0
0
0
0
0
0
0
0
0
6
0
0
0
0
0
0
0
0
0
0
0
0
0
0
0
0
0
0
0
0
0
0
0
0
0
0
0


0
0
0
0
0
0
45
0
0
0
0
0
0
0
0
0
0
0
0
0
0
0
0
0
0
0
0
0
0
0
0
0
0
0
0
0
0
0
0
0
0
0
0
0
0
0
0
0
0
0
0
0
0
6
0
0
0
0
0
0
0
0
0
0
0
0
0
0
0
0
0
0
0
0
0
0
0
0
0
0
0


0
0
0
0
0
0
45
0
0
0
0
0
0
0
0
0
0
0
0
0
0
0
0
0
0
0
0
0
0
0
0
0
0
0
0
0
0
0
0
0
0
0
0
0
0
0
0
0
0
0
0
0
0
6
0
0
0
0
0
0
0
0
0
0
0
0
0
0
0
0
0
0
0
0
0
0
0
0
0
0
0


0
0
0
0
0
0
0
0
2
0
0
0
0
0
0
0
0
0
0
0
0
0
2
0
0
0
0
0
0
0
0
0
0
0
0
0
0
0
0
0
0
0
0
0
0
0
0
0
0
0
0
0
0
0
0
0
0
0
0
0
0
0
0
0
0
0
0
0
0
0
0
0
0
0
0
0
0
0
0
0
0


0
0
0
0
0
0
0
0
2
0
0
0
0
0
0
0
0
0
0
0
0
0
2
0
0
0
0
0
0
0
0
0
0
0
0
0
0
0
0
0
0
0
0
0
0
0
0
0
0
0
0
0
0
0
0
0
0
0
0
0
0
0
0
0
0
0
0
0
0
0
0
0
0
0
0
0
0
0
0
0
0


0
0
0
0
0
0
0
0
2
0
0
0
0
0
0
0
0
0
0
0
0
0
2
0
0
0
0
0
0
0
0
0
0
0
0
0
0
0
0
0
0
0
0
0
0
0
0
0
0
0
0
0
0
0
0
0
0
0
0
0
0
0
0
0
0
0
0
0
0
0
0
0
0
0
0
0
0
0
0
0
0


0
0
0
0
0
0
0
0
2
0
0
0
0
0
0
0
0
0
0
0
0
0
2
0
0
0
0
0
0
0
0
0
0
0
0
0
0
0
0
0
0
0
0
0
0
0
0
0
0
0
0
0
0
0
0
0
0
0
0
0
0
0
0
0
0
0
0
0
0
0
0
0
0
0
0
0
0
0
0
0
0


0
0
0
0
0
0
0
0
2
0
0
0
0
0
0
0
0
0
0
0
0
0
2
0
0
0
0
0
0
0
0
0
0
0
0
0
0
0
0
0
0
0
0
0
0
0
0
0
0
0
0
0
0
0
0
0
0
0
0
0
0
0
0
0
0
0
0
0
0
0
0
0
0
0
0
0
0
0
0
0
0


12
22
2
0
0
3
11
86
1
0
0
1
2
15
17
13
15
17
11
18
69
10
11
0
0
5
0
12
0
12
22
0
0
0
3
0
0
8
16
3
15
9
16
3
7
4
16
0
9
0
25
5
4
7
22
4
25
5
13
1
7
21
1
4
59
18
6
2
1
0
2
0
0
0
2
2
0
0
0
1
0


12
22
2
0
0
3
11
86
1
0
0
1
2
15
17
13
15
17
11
18
69
10
11
0
0
5
0
12
0
12
22
0
0
0
3
0
0
8
16
3
15
9
16
3
7
4
16
0
9
0
25
5
4
7
22
4
25
5
13
1
7
21
1
4
59
18
6
2
1
0
2
0
0
0
2
2
0
0
0
1
0


12
22
2
0
0
3
11
86
1
0
0
1
2
15
17
13
15
17
11
18
69
10
11
0
0
5
0
12
0
12
22
0
0
0
3
0
0
8
16
3
15
9
16
3
7
4
16
0
9
0
25
5
4
7
22
4
25
5
13
1
7
21
1
4
59
18
6
2
1
0
2
0
0
0
2
2
0
0
0
1
0


12
22
2
0
0
3
11
86
1
0
0
1
2
15
17
13
15
17
11
18
69
10
11
0
0
5
0
12
0
12
22
0
0
0
3
0
0
8
16
3
15
9
16
3
7
4
16
0
9
0
25
5
4
7
22
4
25
5
13
1
7
21
1
4
59
18
6
2
1
0
2
0
0
0
2
2
0
0
0
1
0


12
22
2
0
0
3
11
86
1
0
0
1
2
15
17
13
15
17
11
18
69
10
11
0
0
5
0
12
0
12
22
0
0
0
3
0
0
8
16
3
15
9
16
3
7
4
16
0
9
0
25
5
4
7
22
4
25
5
13
1
7
21
1
4
59
18
6
2
1
0
2
0
0
0
2
2
0
0
0
1
0


2
1
0
0
0
0
10
4
5
3
4
2
3
15
2
6
4
0
3
1
1
6
0
0
0
0
0
0
0
0
3
0
0
0
0
0
0
0
1
0
1
3
0
0
0
1
0
0
2
0
24
1
6
2
28
18
33
1
16
16
12
36
1
20
0
6
0
39
4
19
4
0
0
0
2
0
0
2
0
0
0


2
1
0
0
0
0
10
4
5
3
4
2
3
15
2
6
4
0
3
1
1
6
0
0
0
0
0
0
0
0
3
0
0
0
0
0
0
0
1
0
1
3
0
0
0
1
0
0
2
0
24
1
6
2
28
18
33
1
16
16
12
36
1
20
0
6
0
39
4
19
4
0
0
0
2
0
0
2
0
0
0


2
1
0
0
0
0
10
4
5
3
4
2
3
15
2
6
4
0
3
1
1
6
0
0
0
0
0
0
0
0
3
0
0
0
0
0
0
0
1
0
1
3
0
0
0
1
0
0
2
0
24
1
6
2
28
18
33
1
16
16
12
36
1
20
0
6
0
39
4
19
4
0
0
0
2
0
0
2
0
0
0


2
1
0
0
0
0
10
4
5
3
4
2
3
15
2
6
4
0
3
1
1
6
0
0
0
0
0
0
0
0
3
0
0
0
0
0
0
0
1
0
1
3
0
0
0
1
0
0
2
0
24
1
6
2
28
18
33
1
16
16
12
36
1
20
0
6
0
39
4
19
4
0
0
0
2
0
0
2
0
0
0


2
1
0
0
0
0
10
4
5
3
4
2
3
15
2
6
4
0
3
1
1
6
0
0
0
0
0
0
0
0
3
0
0
0
0
0
0
0
1
0
1
3
0
0
0
1
0
0
2
0
24
1
6
2
28
18
33
1
16
16
12
36
1
20
0
6
0
39
4
19
4
0
0
0
2
0
0
2
0
0
0


2
55
5
3
3
0
2
9
10
1
1
0
0
4
2
8
4
12
6
3
26
6
5
0
0
4
0
1
0
3
12
0
0
0
3
0
0
6
17
7
8
6
13
1
4
7
6
6
4
0
4
1
0
2
6
14
6
0
2
18
27
3
3
1
2
7
0
1
5
0
2
0
0
2
0
0
0
0
0
0
0


2
55
5
3
3
0
2
9
10
1
1
0
0
4
2
8
4
12
6
3
26
6
5
0
0
4
0
1
0
3
12
0
0
0
3
0
0
6
17
7
8
6
13
1
4
7
6
6
4
0
4
1
0
2
6
14
6
0
2
18
27
3
3
1
2
7
0
1
5
0
2
0
0
2
0
0
0
0
0
0
0


2
55
5
3
3
0
2
9
10
1
1
0
0
4
2
8
4
12
6
3
26
6
5
0
0
4
0
1
0
3
12
0
0
0
3
0
0
6
17
7
8
6
13
1
4
7
6
6
4
0
4
1
0
2
6
14
6
0
2
18
27
3
3
1
2
7
0
1
5
0
2
0
0
2
0
0
0
0
0
0
0


2
55
5
3
3
0
2
9
10
1
1
0
0
4
2
8
4
12
6
3
26
6
5
0
0
4
0
1
0
3
12
0
0
0
3
0
0
6
17
7
8
6
13
1
4
7
6
6
4
0
4
1
0
2
6
14
6
0
2
18
27
3
3
1
2
7
0
1
5
0
2
0
0
2
0
0
0
0
0
0
0


2
55
5
3
3
0
2
9
10
1
1
0
0
4
2
8
4
12
6
3
26
6
5
0
0
4
0
1
0
3
12
0
0
0
3
0
0
6
17
7
8
6
13
1
4
7
6
6
4
0
4
1
0
2
6
14
6
0
2
18
27
3
3
1
2
7
0
1
5
0
2
0
0
2
0
0
0
0
0
0
0


669
853
1100
830
793
1111
1365
856
1345
497
990
1112
751
1022
985
945
1875
1243
1385
1152
862
925
1186
5
181
1209
170
1022
126
713
786
1107
806
1161
791
3214
183
684
791
911
845
1182
1245
951
902
858
1074
946
1372
570
910
1955
1893
1666
1041
1448
1330
1068
1299
1275
1662
1198
1133
1059
1256
997
1586
692
1232
1801
1105
3585
3922
1955
3059
3530
4339
2446
2900
714
1035


0
0
2
0
0
0
0
0
0
1
0
0
2
2
3
2
6
0
5
2
1
5
0
0
0
6
0
3
0
0
0
0
0
0
0
0
0
5
0
2
0
1
0
0
0
1
0
0
3
0
0
0
0
1
0
0
0
0
0
1
0
0
0
0
0
0
0
0
0
0
0
0
0
0
0
0
0
0
0
0
0


0
0
2
0
0
0
0
0
0
1
0
0
2
2
3
2
6
0
5
2
1
5
0
0
0
6
0
3
0
0
0
0
0
0
0
0
0
5
0
2
0
1
0
0
0
1
0
0
3
0
0
0
0
1
0
0
0
0
0
1
0
0
0
0
0
0
0
0
0
0
0
0
0
0
0
0
0
0
0
0
0


0
0
2
0
0
0
0
0
0
1
0
0
2
2
3
2
6
0
5
2
1
5
0
0
0
6
0
3
0
0
0
0
0
0
0
0
0
5
0
2
0
1
0
0
0
1
0
0
3
0
0
0
0
1
0
0
0
0
0
1
0
0
0
0
0
0
0
0
0
0
0
0
0
0
0
0
0
0
0
0
0


0
0
2
0
0
0
0
0
0
1
0
0
2
2
3
2
6
0
5
2
1
5
0
0
0
6
0
3
0
0
0
0
0
0
0
0
0
5
0
2
0
1
0
0
0
1
0
0
3
0
0
0
0
1
0
0
0
0
0
1
0
0
0
0
0
0
0
0
0
0
0
0
0
0
0
0
0
0
0
0
0


0
0
2
0
0
0
0
0
0
1
0
0
2
2
3
2
6
0
5
2
1
5
0
0
0
6
0
3
0
0
0
0
0
0
0
0
0
5
0
2
0
1
0
0
0
1
0
0
3
0
0
0
0
1
0
0
0
0
0
1
0
0
0
0
0
0
0
0
0
0
0
0
0
0
0
0
0
0
0
0
0


280
322
231
114
266
136
771
304
988
121
566
456
397
629
591
352
878
666
812
397
300
237
549
0
75
467
1
346
126
121
101
1
50
40
276
62
0
230
239
193
291
650
544
270
184
207
405
241
639
217
432
119
214
971
460
755
434
367
411
480
704
691
48
601
251
313
132
327
256
185
432
71
222
215
106
53
62
62
220
58
42


0
0
0
0
0
0
3
2
1
2
4
2
4
3
2
1
6
2
2
0
3
2
1
0
0
2
0
8
0
0
0
0
0
0
0
0
0
2
4
2
2
1
3
0
2
0
3
2
1
0
4
0
0
1
2
2
3
0
0
5
2
6
0
0
0
1
0
1
0
0
0
0
0
0
0
0
0
0
0
0
0


0
0
0
0
0
0
3
2
1
2
4
2
4
3
2
1
6
2
2
0
3
2
1
0
0
2
0
8
0
0
0
0
0
0
0
0
0
2
4
2
2
1
3
0
2
0
3
2
1
0
4
0
0
1
2
2
3
0
0
5
2
6
0
0
0
1
0
1
0
0
0
0
0
0
0
0
0
0
0
0
0


0
0
0
0
0
0
3
2
1
2
4
2
4
3
2
1
6
2
2
0
3
2
1
0
0
2
0
8
0
0
0
0
0
0
0
0
0
2
4
2
2
1
3
0
2
0
3
2
1
0
4
0
0
1
2
2
3
0
0
5
2
6
0
0
0
1
0
1
0
0
0
0
0
0
0
0
0
0
0
0
0


0
0
0
0
0
0
3
2
1
2
4
2
4
3
2
1
6
2
2
0
3
2
1
0
0
2
0
8
0
0
0
0
0
0
0
0
0
2
4
2
2
1
3
0
2
0
3
2
1
0
4
0
0
1
2
2
3
0
0
5
2
6
0
0
0
1
0
1
0
0
0
0
0
0
0
0
0
0
0
0
0


4
0
1
11
1
1
7
5
4
4
11
11
6
7
21
8
11
14
15
5
22
4
16
0
57
11
0
8
0
2
3
0
0
0
8
0
0
19
4
3
10
7
8
2
1
0
4
10
9
0
11
3
6
20
5
16
4
34
7
15
11
6
8
7
10
11
7
9
10
7
6
0
3
3
0
5
1
2
1
7
2


0
0
0
1
0
0
0
2
0
0
0
0
0
0
0
0
0
0
0
0
0
0
0
0
0
0
0
0
0
0
0
0
0
0
0
0
0
0
0
0
0
0
0
0
0
0
0
0
0
0
0
0
0
0
0
0
0
0
0
0
0
0
0
0
0
0
0
0
0
0
0
0
0
0
0
0
0
0
0
0
0


0
0
0
1
0
0
0
2
0
0
0
0
0
0
0
0
0
0
0
0
0
0
0
0
0
0
0
0
0
0
0
0
0
0
0
0
0
0
0
0
0
0
0
0
0
0
0
0
0
0
0
0
0
0
0
0
0
0
0
0
0
0
0
0
0
0
0
0
0
0
0
0
0
0
0
0
0
0
0
0
0


0
0
0
1
0
0
0
2
0
0
0
0
0
0
0
0
0
0
0
0
0
0
0
0
0
0
0
0
0
0
0
0
0
0
0
0
0
0
0
0
0
0
0
0
0
0
0
0
0
0
0
0
0
0
0
0
0
0
0
0
0
0
0
0
0
0
0
0
0
0
0
0
0
0
0
0
0
0
0
0
0


4
0
1
10
1
1
7
3
4
4
11
11
6
7
21
8
11
14
15
5
22
4
16
0
57
11
0
8
0
2
3
0
0
0
8
0
0
19
4
3
10
7
8
2
1
0
4
10
9
0
11
3
6
20
5
16
4
34
6
15
11
6
8
7
10
11
7
9
9
7
6
0
3
3
0
5
1
2
1
7
2


4
0
1
10
1
1
7
3
4
4
11
11
6
7
21
8
11
14
15
5
22
4
16
0
57
11
0
8
0
2
3
0
0
0
8
0
0
19
4
3
10
7
8
2
1
0
4
10
9
0
11
3
6
20
5
16
4
34
6
15
11
6
8
7
10
11
7
9
9
7
6
0
3
3
0
5
1
2
1
7
2


4
0
1
10
1
1
7
3
4
4
11
11
6
7
21
8
11
14
15
5
22
4
16
0
57
11
0
8
0
2
3
0
0
0
8
0
0
19
4
3
10
7
8
2
1
0
4
10
9
0
11
3
6
20
5
16
4
34
6
15
11
6
8
7
10
11
7
9
9
7
6
0
3
3
0
5
1
2
1
7
2


0
0
0
0
0
0
0
0
0
0
0
0
0
0
0
0
0
0
0
0
0
0
0
0
0
0
0
0
0
0
0
0
0
0
0
0
0
0
0
0
0
0
0
0
0
0
0
0
0
0
0
0
0
0
0
0
0
0
1
0
0
0
0
0
0
0
0
0
1
0
0
0
0
0
0
0
0
0
0
0
0


0
0
0
0
0
0
0
0
0
0
0
0
0
0
0
0
0
0
0
0
0
0
0
0
0
0
0
0
0
0
0
0
0
0
0
0
0
0
0
0
0
0
0
0
0
0
0
0
0
0
0
0
0
0
0
0
0
0
1
0
0
0
0
0
0
0
0
0
1
0
0
0
0
0
0
0
0
0
0
0
0


0
0
0
0
0
0
0
0
0
0
0
0
0
0
0
0
0
0
0
0
0
0
0
0
0
0
0
0
0
0
0
0
0
0
0
0
0
0
0
0
0
0
0
0
0
0
0
0
0
0
0
0
0
0
0
0
0
0
1
0
0
0
0
0
0
0
0
0
1
0
0
0
0
0
0
0
0
0
0
0
0


0
0
0
0
0
0
0
0
0
0
0
0
0
0
0
0
0
0
0
0
2
0
2
0
0
0
0
0
0
0
0
0
0
0
0
0
0
0
0
0
0
0
0
0
0
0
0
0
0
0
0
0
0
0
0
0
0
0
0
0
0
0
0
0
0
0
0
0
0
0
0
0
0
2
0
0
0
0
0
0
0


0
0
0
0
0
0
0
0
0
0
0
0
0
0
0
0
0
0
0
0
2
0
2
0
0
0
0
0
0
0
0
0
0
0
0
0
0
0
0
0
0
0
0
0
0
0
0
0
0
0
0
0
0
0
0
0
0
0
0
0
0
0
0
0
0
0
0
0
0
0
0
0
0
2
0
0
0
0
0
0
0


0
0
0
0
0
0
0
0
0
0
0
0
0
0
0
0
0
0
0
0
0
0
1
0
0
0
0
0
0
0
0
0
0
0
0
0
0
0
0
0
0
0
0
0
0
0
0
0
0
0
0
0
0
0
0
0
0
0
0
0
0
0
0
0
0
0
0
0
0
0
0
0
0
0
0
0
0
0
0
0
0


0
0
0
0
0
0
0
0
0
0
0
0
0
0
0
0
0
0
0
0
0
0
1
0
0
0
0
0
0
0
0
0
0
0
0
0
0
0
0
0
0
0
0
0
0
0
0
0
0
0
0
0
0
0
0
0
0
0
0
0
0
0
0
0
0
0
0
0
0
0
0
0
0
0
0
0
0
0
0
0
0


0
0
0
0
0
0
0
0
0
0
0
0
0
0
0
0
0
0
0
0
0
0
1
0
0
0
0
0
0
0
0
0
0
0
0
0
0
0
0
0
0
0
0
0
0
0
0
0
0
0
0
0
0
0
0
0
0
0
0
0
0
0
0
0
0
0
0
0
0
0
0
0
0
1
0
0
0
0
0
0
0


0
0
0
0
0
0
0
0
0
0
0
0
0
0
0
0
0
0
0
0
0
0
1
0
0
0
0
0
0
0
0
0
0
0
0
0
0
0
0
0
0
0
0
0
0
0
0
0
0
0
0
0
0
0
0
0
0
0
0
0
0
0
0
0
0
0
0
0
0
0
0
0
0
1
0
0
0
0
0
0
0


0
0
0
0
0
0
0
0
0
0
0
0
0
0
0
0
0
0
0
0
1
0
0
0
0
0
0
0
0
0
0
0
0
0
0
0
0
0
0
0
0
0
0
0
0
0
0
0
0
0
0
0
0
0
0
0
0
0
0
0
0
0
0
0
0
0
0
0
0
0
0
0
0
0
0
0
0
0
0
0
0


0
0
0
0
0
0
0
0
0
0
0
0
0
0
0
0
0
0
0
0
1
0
0
0
0
0
0
0
0
0
0
0
0
0
0
0
0
0
0
0
0
0
0
0
0
0
0
0
0
0
0
0
0
0
0
0
0
0
0
0
0
0
0
0
0
0
0
0
0
0
0
0
0
0
0
0
0
0
0
0
0


0
0
0
0
0
0
0
0
0
0
0
0
0
0
0
0
0
0
0
0
1
0
0
0
0
0
0
0
0
0
0
0
0
0
0
0
0
0
0
0
0
0
0
0
0
0
0
0
0
0
0
0
0
0
0
0
0
0
0
0
0
0
0
0
0
0
0
0
0
0
0
0
0
1
0
0
0
0
0
0
0


0
0
0
0
0
0
0
0
0
0
0
0
0
0
0
0
0
0
0
0
1
0
0
0
0
0
0
0
0
0
0
0
0
0
0
0
0
0
0
0
0
0
0
0
0
0
0
0
0
0
0
0
0
0
0
0
0
0
0
0
0
0
0
0
0
0
0
0
0
0
0
0
0
1
0
0
0
0
0
0
0


0
2
2
0
0
0
3
0
2
0
5
0
2
1
2
0
9
2
3
0
1
0
4
0
0
1
0
3
0
4
0
0
0
0
0
0
0
0
1
1
1
3
0
0
0
0
2
0
0
0
0
0
0
0
0
0
3
1
0
0
0
0
0
0
0
0
0
0
0
0
0
0
0
0
0
0
0
0
0
0
0


0
2
2
0
0
0
3
0
2
0
5
0
2
1
2
0
9
2
3
0
1
0
4
0
0
1
0
3
0
4
0
0
0
0
0
0
0
0
1
1
1
3
0
0
0
0
2
0
0
0
0
0
0
0
0
0
3
1
0
0
0
0
0
0
0
0
0
0
0
0
0
0
0
0
0
0
0
0
0
0
0


0
2
2
0
0
0
3
0
2
0
5
0
2
1
2
0
9
2
3
0
1
0
4
0
0
1
0
3
0
4
0
0
0
0
0
0
0
0
1
1
1
3
0
0
0
0
2
0
0
0
0
0
0
0
0
0
3
1
0
0
0
0
0
0
0
0
0
0
0
0
0
0
0
0
0
0
0
0
0
0
0


0
2
2
0
0
0
3
0
2
0
5
0
2
1
2
0
9
2
3
0
1
0
4
0
0
1
0
3
0
4
0
0
0
0
0
0
0
0
1
1
1
3
0
0
0
0
2
0
0
0
0
0
0
0
0
0
3
1
0
0
0
0
0
0
0
0
0
0
0
0
0
0
0
0
0
0
0
0
0
0
0


38
17
24
0
45
9
342
55
606
7
58
46
19
23
46
17
197
31
114
13
13
18
46
0
0
7
0
13
104
1
5
0
0
0
32
0
0
7
16
12
24
139
180
0
2
3
28
1
24
0
132
4
38
226
128
375
92
82
100
48
231
266
3
297
27
50
1
131
34
0
73
0
0
0
0
0
0
0
0
1
0


38
17
24
0
45
9
342
55
606
7
58
46
19
23
46
17
197
31
114
13
13
18
46
0
0
7
0
13
104
1
5
0
0
0
32
0
0
7
16
12
24
139
180
0
2
3
28
1
24
0
132
4
38
226
128
375
92
82
100
48
231
266
3
297
27
50
1
131
34
0
73
0
0
0
0
0
0
0
0
1
0


38
17
24
0
45
9
342
55
606
7
58
46
19
23
46
17
197
31
114
13
13
18
46
0
0
7
0
13
104
1
5
0
0
0
32
0
0
7
16
12
24
139
180
0
2
3
28
1
24
0
132
4
38
226
128
375
92
82
100
48
231
266
3
297
27
50
1
131
34
0
73
0
0
0
0
0
0
0
0
1
0


38
17
24
0
45
9
342
55
606
7
58
46
19
23
46
17
197
31
114
13
13
18
46
0
0
7
0
13
104
1
5
0
0
0
32
0
0
7
16
12
24
139
180
0
2
3
28
1
24
0
132
4
38
226
128
375
92
82
100
48
231
266
3
297
27
50
1
131
34
0
73
0
0
0
0
0
0
0
0
1
0


6
11
16
2
4
1
2
0
5
3
7
15
15
11
6
11
16
12
17
9
26
7
13
0
0
11
0
4
0
3
3
0
0
0
0
3
0
1
8
5
4
10
7
2
6
11
7
5
9
0
1
3
4
16
6
5
3
13
7
7
9
7
2
3
4
5
0
4
1
0
5
0
1
2
0
0
0
2
0
1
2


6
11
16
2
4
1
2
0
5
3
7
15
14
11
6
11
16
11
17
9
25
5
13
0
0
11
0
4
0
3
3
0
0
0
0
3
0
1
8
5
3
10
6
2
5
11
6
5
8
0
1
2
3
16
5
5
3
12
6
7
8
7
2
3
3
5
0
4
1
0
5
0
1
1
0
0
0
2
0
1
2


4
9
16
2
4
1
2
0
4
2
6
11
14
8
3
9
12
8
14
4
21
2
4
0
0
3
0
1
0
0
3
0
0
0
0
0
0
1
5
0
3
6
4
1
3
6
3
2
2
0
0
2
0
13
5
3
3
10
6
7
6
6
2
2
3
5
0
4
0
0
2
0
0
0
0
0
0
0
0
1
1


4
9
16
2
4
1
2
0
4
2
6
11
14
8
3
9
12
8
14
4
21
2
4
0
0
3
0
1
0
0
3
0
0
0
0
0
0
1
5
0
3
6
4
1
3
6
3
2
2
0
0
2
0
13
5
3
3
10
6
7
6
6
2
2
3
5
0
4
0
0
2
0
0
0
0
0
0
0
0
1
1


0
1
0
0
0
0
0
0
0
1
0
1
0
2
2
0
3
1
1
0
1
2
7
0
0
7
0
0
0
0
0
0
0
0
0
0
0
0
1
0
0
0
0
0
0
0
0
0
2
0
0
0
0
3
0
0
0
0
0
0
0
0
0
0
0
0
0
0
0
0
0
0
0
0
0
0
0
0
0
0
0


0
1
0
0
0
0
0
0
0
1
0
1
0
2
2
0
3
1
1
0
1
2
7
0
0
7
0
0
0
0
0
0
0
0
0
0
0
0
1
0
0
0
0
0
0
0
0
0
2
0
0
0
0
3
0
0
0
0
0
0
0
0
0
0
0
0
0
0
0
0
0
0
0
0
0
0
0
0
0
0
0


2
1
0
0
0
0
0
0
1
0
1
3
0
1
1
2
1
2
2
5
3
1
2
0
0
1
0
3
0
3
0
0
0
0
0
3
0
0
2
5
0
4
2
1
2
5
3
3
4
0
1
0
3
0
0
2
0
2
0
0
2
1
0
1
0
0
0
0
1
0
3
0
1
1
0
0
0
2
0
0
1


2
1
0
0
0
0
0
0
1
0
1
3
0
1
1
2
1
2
2
5
3
1
2
0
0
1
0
3
0
3
0
0
0
0
0
3
0
0
2
5
0
4
2
1
2
5
3
3
4
0
1
0
3
0
0
2
0
2
0
0
2
1
0
1
0
0
0
0
1
0
3
0
1
1
0
0
0
2
0
0
1


0
0
0
0
0
0
0
0
0
0
0
0
1
0
0
0
0
1
0
0
1
2
0
0
0
0
0
0
0
0
0
0
0
0
0
0
0
0
0
0
1
0
1
0
1
0
1
0
1
0
0
1
1
0
1
0
0
1
1
0
1
0
0
0
1
0
0
0
0
0
0
0
0
1
0
0
0
0
0
0
0


0
0
0
0
0
0
0
0
0
0
0
0
1
0
0
0
0
1
0
0
1
2
0
0
0
0
0
0
0
0
0
0
0
0
0
0
0
0
0
0
1
0
1
0
1
0
1
0
1
0
0
1
1
0
1
0
0
1
1
0
1
0
0
0
1
0
0
0
0
0
0
0
0
1
0
0
0
0
0
0
0


0
0
0
0
0
0
0
0
0
0
0
0
1
0
0
0
0
1
0
0
1
2
0
0
0
0
0
0
0
0
0
0
0
0
0
0
0
0
0
0
1
0
1
0
1
0
1
0
1
0
0
1
1
0
1
0
0
1
1
0
1
0
0
0
1
0
0
0
0
0
0
0
0
1
0
0
0
0
0
0
0


0
0
0
0
0
0
3
0
0
0
12
0
1
0
0
0
0
0
4
0
1
0
0
0
0
0
0
2
0
0
0
0
0
0
0
0
0
0
0
1
0
0
0
0
0
0
2
0
0
0
1
0
0
0
0
0
0
0
0
4
0
1
0
0
0
0
0
0
0
0
0
0
0
0
0
0
0
0
0
0
0


0
0
0
0
0
0
3
0
0
0
12
0
1
0
0
0
0
0
4
0
1
0
0
0
0
0
0
2
0
0
0
0
0
0
0
0
0
0
0
1
0
0
0
0
0
0
2
0
0
0
1
0
0
0
0
0
0
0
0
4
0
1
0
0
0
0
0
0
0
0
0
0
0
0
0
0
0
0
0
0
0


0
0
0
0
0
0
3
0
0
0
12
0
1
0
0
0
0
0
4
0
1
0
0
0
0
0
0
2
0
0
0
0
0
0
0
0
0
0
0
1
0
0
0
0
0
0
2
0
0
0
1
0
0
0
0
0
0
0
0
4
0
1
0
0
0
0
0
0
0
0
0
0
0
0
0
0
0
0
0
0
0


0
0
0
0
0
0
3
0
0
0
12
0
1
0
0
0
0
0
4
0
1
0
0
0
0
0
0
2
0
0
0
0
0
0
0
0
0
0
0
1
0
0
0
0
0
0
2
0
0
0
1
0
0
0
0
0
0
0
0
4
0
1
0
0
0
0
0
0
0
0
0
0
0
0
0
0
0
0
0
0
0


0
0
0
0
0
0
16
0
0
0
0
0
1
72
1
0
0
0
1
0
1
1
0
0
0
7
0
0
0
0
0
0
0
0
0
0
0
0
0
0
0
2
0
0
0
0
0
1
0
0
0
0
2
0
0
0
0
0
0
0
0
0
0
3
0
0
0
7
5
0
0
0
0
0
0
0
0
0
0
0
0


0
0
0
0
0
0
16
0
0
0
0
0
1
72
1
0
0
0
1
0
1
1
0
0
0
7
0
0
0
0
0
0
0
0
0
0
0
0
0
0
0
2
0
0
0
0
0
1
0
0
0
0
2
0
0
0
0
0
0
0
0
0
0
3
0
0
0
7
5
0
0
0
0
0
0
0
0
0
0
0
0


0
0
0
0
0
0
16
0
0
0
0
0
1
72
1
0
0
0
1
0
1
1
0
0
0
7
0
0
0
0
0
0
0
0
0
0
0
0
0
0
0
2
0
0
0
0
0
1
0
0
0
0
2
0
0
0
0
0
0
0
0
0
0
3
0
0
0
7
5
0
0
0
0
0
0
0
0
0
0
0
0


0
0
0
0
0
0
16
0
0
0
0
0
1
72
1
0
0
0
1
0
1
1
0
0
0
7
0
0
0
0
0
0
0
0
0
0
0
0
0
0
0
2
0
0
0
0
0
1
0
0
0
0
2
0
0
0
0
0
0
0
0
0
0
3
0
0
0
7
5
0
0
0
0
0
0
0
0
0
0
0
0


21
21
31
11
13
8
31
10
31
7
38
49
22
38
27
17
67
55
31
60
9
20
23
0
0
15
0
13
0
0
1
0
0
0
7
0
0
12
2
2
8
16
20
7
5
4
5
12
28
0
22
11
23
73
27
50
22
23
40
64
73
26
1
49
29
35
9
13
55
24
27
0
4
7
2
1
2
2
0
2
1


21
21
31
11
13
8
31
10
31
7
38
49
22
38
27
17
67
55
31
60
9
20
23
0
0
15
0
13
0
0
1
0
0
0
7
0
0
12
2
2
8
16
20
7
5
4
5
12
28
0
22
11
23
73
27
50
22
23
40
64
73
26
1
49
29
35
9
13
55
24
27
0
4
7
2
1
2
2
0
2
1


21
21
31
11
13
8
31
10
31
7
38
49
22
38
27
17
67
55
31
60
9
20
23
0
0
15
0
13
0
0
1
0
0
0
7
0
0
12
2
2
8
16
20
7
5
4
5
12
28
0
22
11
23
73
27
50
22
23
40
64
73
26
1
49
29
35
9
13
55
24
27
0
4
7
2
1
2
2
0
2
1


21
21
31
11
13
8
31
10
31
7
38
49
22
38
27
17
67
55
31
60
9
20
23
0
0
15
0
13
0
0
1
0
0
0
7
0
0
12
2
2
8
16
20
7
5
4
5
12
28
0
22
11
23
73
27
50
22
23
40
64
73
26
1
49
29
35
9
13
55
24
27
0
4
7
2
1
2
2
0
2
1


6
1
0
0
0
0
12
6
2
0
25
10
10
43
15
2
16
16
22
0
8
5
10
0
0
17
0
9
0
0
0
0
0
0
0
0
0
3
0
2
4
14
15
0
0
1
11
5
3
0
15
2
9
17
10
5
8
2
35
54
28
22
0
13
1
5
0
12
0
2
1
0
0
0
0
0
0
0
0
0
0


6
1
0
0
0
0
12
6
2
0
25
10
10
43
15
2
16
16
22
0
8
5
10
0
0
17
0
9
0
0
0
0
0
0
0
0
0
3
0
2
4
14
15
0
0
1
11
5
3
0
15
2
9
17
10
5
8
2
35
54
28
22
0
13
1
5
0
12
0
2
1
0
0
0
0
0
0
0
0
0
0


6
1
0
0
0
0
12
6
2
0
25
10
10
43
15
2
16
16
22
0
8
5
10
0
0
17
0
9
0
0
0
0
0
0
0
0
0
3
0
2
4
14
15
0
0
1
11
5
3
0
15
2
9
17
10
5
8
2
35
54
28
22
0
13
1
5
0
12
0
2
1
0
0
0
0
0
0
0
0
0
0


6
1
0
0
0
0
12
6
2
0
25
10
10
43
15
2
16
16
22
0
8
5
10
0
0
17
0
9
0
0
0
0
0
0
0
0
0
3
0
2
4
14
15
0
0
1
11
5
3
0
15
2
9
17
10
5
8
2
35
54
28
22
0
13
1
5
0
12
0
2
1
0
0
0
0
0
0
0
0
0
0


171
249
147
67
185
108
253
171
288
90
385
297
301
401
451
277
436
508
570
286
142
142
390
0
18
335
0
247
1
74
56
0
15
20
109
53
0
141
173
144
213
389
183
172
81
103
297
163
530
0
205
89
121
577
249
266
266
185
204
255
292
318
32
210
172
193
100
136
134
136
302
69
208
191
101
44
49
47
200
47
31


113
210
134
43
165
78
201
117
272
79
338
284
272
353
416
234
385
444
514
253
84
106
313
0
0
273
0
174
1
37
36
0
1
0
54
44
0
113
127
117
173
333
119
109
33
81
200
109
459
0
177
62
73
507
192
199
205
114
171
240
214
267
24
197
101
140
72
121
76
29
249
2
4
46
33
0
2
3
16
19
2


113
210
134
43
165
78
201
117
272
79
338
284
272
353
416
234
385
444
514
253
84
106
313
0
0
273
0
174
1
37
36
0
1
0
54
44
0
113
127
117
173
333
119
109
33
81
200
109
459
0
177
62
73
507
192
199
205
114
171
240
214
267
24
197
101
140
72
121
76
29
249
2
4
46
33
0
2
3
16
19
2


113
210
134
43
165
78
201
117
272
79
338
284
272
353
416
234
385
444
514
253
84
106
313
0
0
273
0
174
1
37
36
0
1
0
54
44
0
113
127
117
173
333
119
109
33
81
200
109
459
0
177
62
73
507
192
199
205
114
171
240
214
267
24
197
101
140
72
121
76
29
249
2
4
46
33
0
2
3
16
19
2


0
2
0
0
4
0
17
1
4
0
34
3
27
35
12
17
10
2
42
0
8
0
6
0
0
0
0
0
0
0
0
0
4
0
0
0
0
0
0
0
5
6
0
0
0
0
1
0
0
0
4
1
1
11
5
0
4
0
0
1
0
4
0
0
0
1
0
0
0
0
1
0
0
0
0
0
0
0
0
0
0


0
2
0
0
4
0
17
1
4
0
34
3
27
35
12
17
10
2
42
0
8
0
6
0
0
0
0
0
0
0
0
0
4
0
0
0
0
0
0
0
5
6
0
0
0
0
1
0
0
0
4
1
1
11
5
0
4
0
0
1
0
4
0
0
0
1
0
0
0
0
1
0
0
0
0
0
0
0
0
0
0


0
2
0
0
4
0
17
1
4
0
34
3
27
35
12
17
10
2
42
0
8
0
6
0
0
0
0
0
0
0
0
0
4
0
0
0
0
0
0
0
5
6
0
0
0
0
1
0
0
0
4
1
1
11
5
0
4
0
0
1
0
4
0
0
0
1
0
0
0
0
1
0
0
0
0
0
0
0
0
0
0


58
32
13
24
15
30
32
53
12
11
12
7
0
13
22
25
40
56
11
32
50
36
67
0
18
62
0
72
0
37
20
0
10
20
55
9
0
28
46
27
32
49
63
63
48
22
95
54
67
0
24
14
39
58
51
67
57
71
33
14
77
45
8
13
70
50
28
14
57
99
50
67
192
144
53
44
40
43
172
28
29


58
32
13
24
15
30
32
53
12
11
12
7
0
13
22
25
40
56
11
32
50
36
67
0
18
62
0
72
0
37
20
0
10
20
55
9
0
28
46
27
32
49
63
63
48
22
95
54
67
0
24
14
39
58
51
67
57
71
33
14
77
45
8
13
70
50
28
14
57
99
50
67
192
144
53
44
40
43
172
28
29


58
32
13
24
15
30
32
53
12
11
12
7
0
13
22
25
40
56
11
32
50
36
67
0
18
62
0
72
0
37
20
0
10
20
55
9
0
28
46
27
32
49
63
63
48
22
95
54
67
0
24
14
39
58
51
67
57
71
33
14
77
45
8
13
70
50
28
14
57
99
50
67
192
144
53
44
40
43
172
28
29


0
5
0
0
1
0
3
0
0
0
1
3
2
0
1
1
1
6
3
1
0
0
4
0
0
0
0
1
0
0
0
0
0
0
0
0
0
0
0
0
3
1
1
0
0
0
1
0
4
0
0
12
8
1
1
0
0
0
0
0
1
2
0
0
1
2
0
1
1
8
2
0
12
1
15
0
7
1
12
0
0


0
5
0
0
1
0
3
0
0
0
1
3
2
0
1
1
1
6
3
1
0
0
4
0
0
0
0
1
0
0
0
0
0
0
0
0
0
0
0
0
3
1
1
0
0
0
1
0
4
0
0
12
8
1
1
0
0
0
0
0
1
2
0
0
1
2
0
1
1
8
2
0
12
1
15
0
7
1
12
0
0


0
5
0
0
1
0
3
0
0
0
1
3
2
0
1
1
1
6
3
1
0
0
4
0
0
0
0
1
0
0
0
0
0
0
0
0
0
0
0
0
3
1
1
0
0
0
1
0
4
0
0
12
8
1
1
0
0
0
0
0
1
2
0
0
1
2
0
1
1
8
2
0
12
1
15
0
7
1
12
0
0


10
4
0
13
2
3
35
33
0
1
0
0
0
0
0
0
0
0
0
0
27
14
4
0
0
0
0
5
0
25
11
0
0
9
102
1
0
0
1
2
5
10
63
57
55
45
1
5
1
217
0
0
5
0
0
0
0
1
0
0
0
0
0
0
0
1
2
0
0
0
0
0
2
0
0
0
1
3
3
0
1


10
4
0
13
2
3
35
33
0
1
0
0
0
0
0
0
0
0
0
0
27
14
4
0
0
0
0
5
0
25
11
0
0
9
102
1
0
0
1
2
5
10
63
57
55
45
1
5
1
217
0
0
5
0
0
0
0
1
0
0
0
0
0
0
0
1
2
0
0
0
0
0
2
0
0
0
1
3
3
0
1


10
4
0
13
2
3
35
33
0
1
0
0
0
0
0
0
0
0
0
0
27
14
4
0
0
0
0
5
0
25
11
0
0
9
102
1
0
0
1
2
5
10
63
57
55
45
1
5
1
217
0
0
5
0
0
0
0
1
0
0
0
0
0
0
0
1
2
0
0
0
0
0
2
0
0
0
1
3
3
0
1


10
4
0
13
2
3
35
33
0
1
0
0
0
0
0
0
0
0
0
0
27
14
4
0
0
0
0
5
0
25
11
0
0
9
102
1
0
0
1
2
5
10
63
57
55
45
1
5
1
217
0
0
5
0
0
0
0
1
0
0
0
0
0
0
0
1
2
0
0
0
0
0
2
0
0
0
1
3
3
0
1


0
0
0
0
0
0
0
0
0
0
0
0
0
0
0
0
0
0
0
0
0
0
0
0
0
0
0
0
0
0
0
0
0
0
0
0
0
0
0
0
0
0
0
0
0
0
0
0
0
0
0
0
0
0
0
0
0
0
0
0
0
0
0
0
0
0
0
0
0
0
0
0
0
1
0
0
0
0
0
0
0


0
0
0
0
0
0
0
0
0
0
0
0
0
0
0
0
0
0
0
0
0
0
0
0
0
0
0
0
0
0
0
0
0
0
0
0
0
0
0
0
0
0
0
0
0
0
0
0
0
0
0
0
0
0
0
0
0
0
0
0
0
0
0
0
0
0
0
0
0
0
0
0
0
1
0
0
0
0
0
0
0


0
0
0
0
0
0
0
0
0
0
0
0
0
0
0
0
0
0
0
0
0
0
0
0
0
0
0
0
0
0
0
0
0
0
0
0
0
0
0
0
0
0
0
0
0
0
0
0
0
0
0
0
0
0
0
0
0
0
0
0
0
0
0
0
0
0
0
0
0
0
0
0
0
1
0
0
0
0
0
0
0


0
0
0
0
0
0
0
0
0
0
0
0
0
0
0
0
0
0
0
0
0
0
0
0
0
0
0
0
0
0
0
0
0
0
0
0
0
0
0
0
0
0
0
0
0
0
0
0
0
0
0
0
0
0
0
0
0
0
0
0
0
0
0
0
0
0
0
0
0
0
0
0
0
1
0
0
0
0
0
0
0


5
6
4
4
1
2
3
3
0
3
0
0
0
0
0
3
1
1
1
9
7
7
3
0
0
0
0
4
0
5
6
0
35
11
11
1
0
3
3
2
6
8
29
12
22
32
11
16
8
0
2
3
3
13
0
3
6
10
0
2
2
2
1
0
1
3
7
1
6
7
2
0
2
1
0
1
1
4
5
0
3


5
6
4
4
1
2
3
3
0
3
0
0
0
0
0
3
1
1
1
9
7
7
3
0
0
0
0
4
0
5
6
0
35
11
11
1
0
3
3
2
6
8
29
12
22
32
11
16
8
0
2
3
3
13
0
3
6
10
0
2
2
2
1
0
1
3
7
1
6
7
2
0
2
1
0
1
1
4
5
0
3


5
6
4
4
1
2
3
3
0
3
0
0
0
0
0
3
1
1
1
9
7
7
3
0
0
0
0
4
0
5
6
0
35
11
11
1
0
3
3
2
6
8
29
12
22
32
11
16
8
0
2
3
3
13
0
3
6
10
0
2
2
2
1
0
1
3
7
1
6
7
2
0
2
1
0
1
1
4
5
0
3


5
6
4
4
1
2
3
3
0
3
0
0
0
0
0
3
1
1
1
9
7
7
3
0
0
0
0
4
0
5
6
0
35
11
11
1
0
3
3
2
6
8
29
12
22
32
11
16
8
0
2
3
3
13
0
3
6
10
0
2
2
2
1
0
1
3
7
1
6
7
2
0
2
1
0
1
1
4
5
0
3


9
9
5
4
5
0
38
5
19
2
5
16
5
16
9
11
23
12
6
11
24
10
24
0
0
52
1
15
1
4
3
1
0
0
2
0
0
41
15
10
8
26
23
11
7
4
17
21
11
0
27
3
3
25
7
19
8
7
16
21
23
14
0
3
7
4
6
10
7
0
11
0
0
0
0
0
0
0
0
0
0


9
9
5
4
5
0
38
5
19
2
5
16
5
16
9
11
23
12
6
11
24
10
24
0
0
52
1
15
1
4
3
1
0
0
2
0
0
41
15
10
8
26
23
11
7
4
17
21
11
0
27
3
3
25
7
19
8
7
16
21
23
14
0
3
7
4
6
10
7
0
11
0
0
0
0
0
0
0
0
0
0


9
9
5
4
5
0
38
5
19
2
5
16
5
16
9
11
23
12
6
11
24
10
24
0
0
52
1
15
1
4
3
1
0
0
2
0
0
41
15
10
8
26
23
11
7
4
17
21
11
0
27
3
3
25
7
19
8
7
16
21
23
14
0
3
7
4
6
10
7
0
11
0
0
0
0
0
0
0
0
0
0


9
9
5
4
5
0
38
5
19
2
5
16
5
16
9
11
23
12
6
11
24
10
24
0
0
52
1
15
1
4
3
1
0
0
2
0
0
41
15
10
8
26
23
11
7
4
17
21
11
0
27
3
3
25
7
19
8
7
16
21
23
14
0
3
7
4
6
10
7
0
11
0
0
0
0
0
0
0
0
0
0


0
0
0
0
0
0
5
0
0
0
0
0
0
0
0
0
5
0
1
0
0
0
0
0
0
0
0
0
0
0
0
0
0
0
0
0
0
0
0
0
0
0
0
0
0
0
0
0
0
0
0
0
0
0
15
1
0
0
0
0
0
0
0
0
0
1
0
1
0
0
0
0
0
0
0
0
0
0
0
0
0


0
0
0
0
0
0
5
0
0
0
0
0
0
0
0
0
5
0
1
0
0
0
0
0
0
0
0
0
0
0
0
0
0
0
0
0
0
0
0
0
0
0
0
0
0
0
0
0
0
0
0
0
0
0
15
1
0
0
0
0
0
0
0
0
0
1
0
1
0
0
0
0
0
0
0
0
0
0
0
0
0


0
0
0
0
0
0
5
0
0
0
0
0
0
0
0
0
5
0
1
0
0
0
0
0
0
0
0
0
0
0
0
0
0
0
0
0
0
0
0
0
0
0
0
0
0
0
0
0
0
0
0
0
0
0
15
1
0
0
0
0
0
0
0
0
0
1
0
1
0
0
0
0
0
0
0
0
0
0
0
0
0


0
0
0
0
0
0
5
0
0
0
0
0
0
0
0
0
5
0
1
0
0
0
0
0
0
0
0
0
0
0
0
0
0
0
0
0
0
0
0
0
0
0
0
0
0
0
0
0
0
0
0
0
0
0
15
1
0
0
0
0
0
0
0
0
0
1
0
1
0
0
0
0
0
0
0
0
0
0
0
0
0


10
2
1
2
10
4
18
14
30
2
16
10
11
14
11
5
91
13
25
4
14
7
13
0
0
9
0
15
20
3
13
0
0
0
5
4
0
1
12
7
6
25
13
7
3
4
17
0
15
0
12
1
0
3
11
13
19
9
2
5
33
23
1
16
0
4
0
2
4
9
5
2
2
8
3
2
8
2
11
0
2


10
2
1
2
10
4
18
14
30
2
16
10
11
14
11
5
91
13
25
4
14
7
13
0
0
9
0
15
20
3
13
0
0
0
5
4
0
1
12
7
6
25
13
7
3
4
17
0
15
0
12
1
0
3
11
13
19
9
2
5
33
23
1
16
0
4
0
2
4
9
5
2
2
8
3
2
8
2
11
0
2


10
2
1
2
10
4
18
14
30
2
16
10
11
14
11
5
91
13
25
4
14
7
13
0
0
9
0
15
20
3
13
0
0
0
5
4
0
1
12
7
6
25
13
7
3
4
17
0
15
0
12
1
0
3
11
13
19
9
2
5
33
23
1
16
0
4
0
2
4
9
5
2
2
8
3
2
8
2
11
0
2


10
2
1
2
10
4
18
14
30
2
16
10
11
14
11
5
91
13
25
4
14
7
13
0
0
9
0
15
20
3
13
0
0
0
5
4
0
1
12
7
6
25
13
7
3
4
17
0
15
0
12
1
0
3
11
13
19
9
2
5
33
23
1
16
0
4
0
2
4
9
5
2
2
8
3
2
8
2
11
0
2


10
10
3
9
5
9
11
24
1
10
3
12
0
1
12
25
25
9
0
25
16
10
9
0
0
18
0
20
0
26
51
3
65
1
18
12
0
31
32
20
15
13
29
30
18
32
22
43
16
0
10
14
12
18
19
29
9
38
15
13
27
11
38
2
21
27
20
3
13
18
9
24
32
39
9
21
16
36
21
23
9


10
10
3
9
5
9
11
24
1
10
3
12
0
1
12
25
25
9
0
25
16
10
9
0
0
18
0
20
0
26
51
3
65
1
18
12
0
31
32
20
15
13
29
30
18
32
22
43
16
0
10
14
12
18
19
29
9
38
15
13
27
11
38
2
21
27
20
3
13
18
9
24
32
39
9
21
16
36
21
23
9


10
10
3
9
5
9
11
24
1
10
3
12
0
1
12
25
25
9
0
25
16
10
9
0
0
18
0
20
0
26
51
3
65
1
18
12
0
31
32
20
15
13
29
30
18
32
22
43
16
0
10
14
12
18
19
29
9
38
15
13
27
11
38
2
21
27
20
3
13
18
9
24
32
39
9
21
16
36
21
23
9


10
10
3
9
5
9
11
24
1
10
3
12
0
1
12
25
25
9
0
25
16
10
9
0
0
18
0
20
0
26
51
3
65
1
18
12
0
31
32
20
15
13
29
30
18
32
22
43
16
0
10
14
12
18
19
29
9
38
15
13
27
11
38
2
21
27
20
3
13
18
9
24
32
39
9
21
16
36
21
23
9


10
10
3
9
5
9
11
24
1
10
3
12
0
1
12
25
25
9
0
25
16
10
9
0
0
18
0
20
0
26
51
3
65
1
18
12
0
31
32
20
15
13
29
30
18
32
22
43
16
0
10
14
12
18
19
29
9
38
15
13
27
11
38
2
21
27
20
3
13
18
9
24
32
39
9
21
16
36
21
23
9


56
78
41
37
43
19
74
90
9
21
41
38
25
22
40
68
151
87
54
57
114
173
205
0
33
329
15
265
0
126
156
7
54
27
147
33
0
151
199
224
175
219
241
199
205
211
252
191
185
0
64
30
30
99
66
77
56
59
136
115
89
72
12
42
32
36
6
39
40
7
31
2
3
13
14
2
5
6
16
6
7


2
7
3
0
0
0
1
1
0
2
2
0
0
1
2
5
2
6
1
6
8
2
1
0
0
27
0
33
0
9
8
2
36
1
10
0
0
11
12
19
13
5
2
1
9
11
34
10
4
0
4
3
7
3
2
1
3
8
8
8
4
1
1
0
4
4
1
2
3
0
4
0
3
2
4
1
1
2
1
1
4


2
7
3
0
0
0
1
1
0
2
2
0
0
1
2
5
2
6
1
6
8
2
1
0
0
27
0
33
0
9
8
2
36
1
10
0
0
11
12
19
13
5
2
1
9
11
34
10
4
0
4
3
7
3
2
1
3
8
8
8
4
1
1
0
4
4
1
2
3
0
4
0
3
2
4
1
1
2
1
1
4


2
7
3
0
0
0
1
1
0
2
2
0
0
1
2
5
2
6
1
6
8
2
1
0
0
27
0
33
0
9
8
2
36
1
10
0
0
11
12
19
13
5
2
1
9
11
34
10
4
0
4
3
7
3
2
1
3
8
8
8
4
1
1
0
4
4
1
2
3
0
4
0
3
2
4
1
1
2
1
1
4


2
7
3
0
0
0
1
1
0
2
2
0
0
1
2
5
2
6
1
6
8
2
1
0
0
27
0
33
0
9
8
2
36
1
10
0
0
11
12
19
13
5
2
1
9
11
34
10
4
0
4
3
7
3
2
1
3
8
8
8
4
1
1
0
4
4
1
2
3
0
4
0
3
2
4
1
1
2
1
1
4


0
0
0
0
0
0
0
0
0
0
0
0
0
0
0
2
0
0
0
0
4
2
2
0
0
38
0
15
0
1
0
0
0
0
11
1
0
8
7
12
7
5
5
2
3
4
14
2
4
0
1
4
0
1
1
1
3
3
2
1
3
4
0
4
0
0
0
0
0
0
1
0
0
0
2
0
0
0
1
0
0


0
0
0
0
0
0
0
0
0
0
0
0
0
0
0
0
0
0
0
0
0
0
0
0
0
1
0
0
0
0
0
0
0
0
0
0
0
1
0
0
0
0
0
0
1
0
1
0
0
0
0
0
0
0
0
0
0
0
0
0
0
0
0
0
0
0
0
0
0
0
0
0
0
0
0
0
0
0
0
0
0


0
0
0
0
0
0
0
0
0
0
0
0
0
0
0
0
0
0
0
0
0
0
0
0
0
1
0
0
0
0
0
0
0
0
0
0
0
1
0
0
0
0
0
0
1
0
1
0
0
0
0
0
0
0
0
0
0
0
0
0
0
0
0
0
0
0
0
0
0
0
0
0
0
0
0
0
0
0
0
0
0


0
0
0
0
0
0
0
0
0
0
0
0
0
0
0
0
0
0
0
0
0
0
0
0
0
1
0
0
0
0
0
0
0
0
0
0
0
1
0
0
0
0
0
0
1
0
1
0
0
0
0
0
0
0
0
0
0
0
0
0
0
0
0
0
0
0
0
0
0
0
0
0
0
0
0
0
0
0
0
0
0


0
0
0
0
0
0
0
0
0
0
0
0
0
0
0
0
0
0
0
0
0
0
0
0
0
2
0
1
0
0
0
0
0
0
0
0
0
0
2
1
0
1
0
0
1
0
0
0
0
0
0
0
0
0
0
0
2
0
0
0
0
0
0
0
0
0
0
0
0
0
0
0
0
0
0
0
0
0
0
0
0


0
0
0
0
0
0
0
0
0
0
0
0
0
0
0
0
0
0
0
0
0
0
0
0
0
2
0
1
0
0
0
0
0
0
0
0
0
0
2
1
0
1
0
0
1
0
0
0
0
0
0
0
0
0
0
0
2
0
0
0
0
0
0
0
0
0
0
0
0
0
0
0
0
0
0
0
0
0
0
0
0


0
0
0
0
0
0
0
0
0
0
0
0
0
0
0
0
0
0
0
0
0
0
0
0
0
2
0
1
0
0
0
0
0
0
0
0
0
0
2
1
0
1
0
0
1
0
0
0
0
0
0
0
0
0
0
0
2
0
0
0
0
0
0
0
0
0
0
0
0
0
0
0
0
0
0
0
0
0
0
0
0


0
0
0
0
0
0
0
0
0
0
0
0
0
0
0
1
0
0
0
0
4
1
1
0
0
10
0
11
0
0
0
0
0
0
4
0
0
0
3
4
4
2
3
1
1
2
7
0
1
0
1
4
0
1
0
1
0
3
2
1
3
0
0
3
0
0
0
0
0
0
1
0
0
0
2
0
0
0
0
0
0


0
0
0
0
0
0
0
0
0
0
0
0
0
0
0
1
0
0
0
0
4
1
1
0
0
10
0
11
0
0
0
0
0
0
4
0
0
0
3
4
4
2
3
1
1
2
7
0
1
0
1
4
0
1
0
1
0
3
2
1
3
0
0
3
0
0
0
0
0
0
1
0
0
0
2
0
0
0
0
0
0


0
0
0
0
0
0
0
0
0
0
0
0
0
0
0
1
0
0
0
0
4
1
1
0
0
10
0
11
0
0
0
0
0
0
4
0
0
0
3
4
4
2
3
1
1
2
7
0
1
0
1
4
0
1
0
1
0
3
2
1
3
0
0
3
0
0
0
0
0
0
1
0
0
0
2
0
0
0
0
0
0


0
0
0
0
0
0
0
0
0
0
0
0
0
0
0
1
0
0
0
0
0
1
1
0
0
25
0
3
0
1
0
0
0
0
7
1
0
7
2
7
3
2
2
1
0
2
6
2
3
0
0
0
0
0
1
0
1
0
0
0
0
4
0
1
0
0
0
0
0
0
0
0
0
0
0
0
0
0
1
0
0


0
0
0
0
0
0
0
0
0
0
0
0
0
0
0
1
0
0
0
0
0
1
1
0
0
25
0
3
0
1
0
0
0
0
7
1
0
7
2
7
3
2
2
1
0
2
6
2
3
0
0
0
0
0
1
0
1
0
0
0
0
4
0
1
0
0
0
0
0
0
0
0
0
0
0
0
0
0
1
0
0


0
0
0
0
0
0
0
0
0
0
0
0
0
0
0
1
0
0
0
0
0
1
1
0
0
25
0
3
0
1
0
0
0
0
7
1
0
7
2
7
3
2
2
1
0
2
6
2
3
0
0
0
0
0
1
0
1
0
0
0
0
4
0
1
0
0
0
0
0
0
0
0
0
0
0
0
0
0
1
0
0


0
12
7
0
2
0
3
0
0
0
4
0
3
0
0
1
1
5
3
0
1
1
1
0
0
1
0
1
0
0
5
0
0
0
0
0
0
0
11
5
1
1
1
1
0
2
4
0
1
0
2
3
1
0
0
3
1
4
0
6
5
2
3
1
1
4
0
0
0
0
0
0
0
0
0
0
0
0
0
1
0


0
12
7
0
2
0
3
0
0
0
4
0
3
0
0
1
1
5
3
0
1
1
1
0
0
1
0
1
0
0
5
0
0
0
0
0
0
0
11
5
1
1
1
1
0
2
4
0
1
0
2
3
1
0
0
3
1
4
0
6
5
2
3
1
1
4
0
0
0
0
0
0
0
0
0
0
0
0
0
1
0


0
12
7
0
2
0
3
0
0
0
4
0
3
0
0
1
1
5
3
0
1
1
1
0
0
1
0
1
0
0
5
0
0
0
0
0
0
0
11
5
1
1
1
1
0
2
4
0
1
0
2
3
1
0
0
3
1
4
0
6
5
2
3
1
1
4
0
0
0
0
0
0
0
0
0
0
0
0
0
1
0


0
12
7
0
2
0
3
0
0
0
4
0
3
0
0
1
1
5
3
0
1
1
1
0
0
1
0
1
0
0
5
0
0
0
0
0
0
0
11
5
1
1
1
1
0
2
4
0
1
0
2
3
1
0
0
3
1
4
0
6
5
2
3
1
1
4
0
0
0
0
0
0
0
0
0
0
0
0
0
1
0


53
58
30
37
41
19
69
87
9
19
35
38
22
21
38
58
148
74
50
50
101
168
201
0
33
260
15
213
0
115
141
5
18
26
126
32
0
130
165
183
153
207
233
195
192
190
200
175
175
0
57
20
22
95
61
71
49
44
126
99
77
65
8
37
27
27
5
37
35
6
26
2
0
11
7
1
4
4
13
4
3


9
13
10
7
11
3
15
20
3
2
24
12
16
7
20
18
71
33
18
12
46
35
60
0
0
71
0
71
0
14
9
3
10
0
28
2
0
26
33
55
41
60
54
26
36
31
55
31
37
0
17
7
7
18
29
14
12
10
20
24
13
23
1
10
1
2
0
14
3
0
4
0
0
0
0
0
0
0
1
0
0


9
13
10
7
11
3
15
20
3
2
24
12
16
7
20
18
71
33
18
12
46
35
60
0
0
71
0
71
0
14
9
3
10
0
28
2
0
26
33
55
41
60
54
26
36
31
55
31
37
0
17
7
7
18
29
14
12
10
20
24
13
23
1
10
1
2
0
14
3
0
4
0
0
0
0
0
0
0
1
0
0


9
13
10
7
11
3
15
20
3
2
24
12
16
7
20
18
71
33
18
12
46
35
60
0
0
71
0
71
0
14
9
3
10
0
28
2
0
26
33
55
41
60
54
26
36
31
55
31
37
0
17
7
7
18
29
14
12
10
20
24
13
23
1
10
1
2
0
14
3
0
4
0
0
0
0
0
0
0
1
0
0


39
40
15
19
21
14
34
44
5
12
9
24
6
12
15
32
53
31
23
35
51
114
117
0
33
152
15
104
0
86
108
2
8
26
78
24
0
98
117
93
83
112
126
130
111
111
103
111
121
0
33
11
11
61
19
50
32
28
99
67
51
38
6
21
24
21
3
20
31
3
22
2
0
8
1
0
4
4
7
3
3


36
36
13
19
16
14
28
35
5
11
8
23
6
12
15
30
47
31
23
35
50
106
107
0
33
140
0
86
0
80
96
2
8
14
74
21
0
88
111
77
78
94
117
120
95
98
95
106
110
0
31
10
10
54
12
43
30
27
99
65
48
32
5
17
22
16
2
16
19
3
20
2
0
8
1
0
4
4
7
3
2


36
36
13
19
16
14
28
35
5
11
8
23
6
12
15
30
47
31
23
35
50
106
107
0
33
140
0
86
0
80
96
2
8
14
74
21
0
88
111
77
78
94
117
120
95
98
95
106
110
0
31
10
10
54
12
43
30
27
99
65
48
32
5
17
22
16
2
16
19
3
20
2
0
8
1
0
4
4
7
3
2


0
0
0
0
0
0
0
0
0
0
0
0
0
0
0
0
0
0
0
0
0
0
0
0
0
8
0
5
0
0
0
0
0
0
0
1
0
0
2
5
1
0
0
1
4
0
4
0
0
0
0
0
0
0
0
0
0
0
0
0
0
0
0
0
0
0
0
0
0
0
0
0
0
0
0
0
0
0
0
0
0


0
0
0
0
0
0
0
0
0
0
0
0
0
0
0
0
0
0
0
0
0
0
0
0
0
8
0
5
0
0
0
0
0
0
0
1
0
0
2
5
1
0
0
1
4
0
4
0
0
0
0
0
0
0
0
0
0
0
0
0
0
0
0
0
0
0
0
0
0
0
0
0
0
0
0
0
0
0
0
0
0


3
4
2
0
5
0
6
9
0
1
1
1
0
0
0
2
6
0
0
0
1
8
10
0
0
4
15
13
0
6
12
0
0
12
4
2
0
10
4
11
4
18
9
9
12
13
4
5
11
0
2
1
1
7
7
7
2
1
0
2
3
6
1
4
2
5
1
4
12
0
2
0
0
0
0
0
0
0
0
0
1


3
4
2
0
5
0
6
9
0
1
1
1
0
0
0
2
6
0
0
0
1
8
10
0
0
4
15
13
0
6
12
0
0
12
4
2
0
10
4
11
4
18
9
9
12
13
4
5
11
0
2
1
1
7
7
7
2
1
0
2
3
6
1
4
2
5
1
4
12
0
2
0
0
0
0
0
0
0
0
0
1


1
0
0
5
0
0
4
3
0
1
0
0
0
0
0
0
1
0
0
0
0
0
0
0
0
0
0
3
0
0
1
0
0
0
2
0
0
0
0
0
0
5
6
2
2
9
0
0
1
0
0
0
0
0
0
0
0
0
0
0
1
0
0
0
0
0
0
0
0
0
0
0
0
0
0
0
0
0
0
0
0


1
0
0
5
0
0
4
3
0
1
0
0
0
0
0
0
1
0
0
0
0
0
0
0
0
0
0
3
0
0
1
0
0
0
2
0
0
0
0
0
0
5
6
2
2
9
0
0
1
0
0
0
0
0
0
0
0
0
0
0
1
0
0
0
0
0
0
0
0
0
0
0
0
0
0
0
0
0
0
0
0


1
0
0
5
0
0
4
3
0
1
0
0
0
0
0
0
1
0
0
0
0
0
0
0
0
0
0
3
0
0
1
0
0
0
2
0
0
0
0
0
0
5
6
2
2
9
0
0
1
0
0
0
0
0
0
0
0
0
0
0
1
0
0
0
0
0
0
0
0
0
0
0
0
0
0
0
0
0
0
0
0


4
5
5
6
9
2
16
20
1
4
2
2
0
2
3
8
23
10
9
3
4
19
24
0
0
37
0
35
0
15
23
0
0
0
18
6
0
6
15
35
29
30
47
37
43
39
42
33
16
0
7
2
4
16
13
7
5
6
7
8
12
4
1
6
2
4
2
3
1
3
0
0
0
3
6
1
0
0
5
1
0


4
5
5
6
9
2
16
20
1
4
2
2
0
2
3
8
23
10
9
3
4
19
24
0
0
37
0
35
0
15
23
0
0
0
18
6
0
6
15
35
29
30
47
37
43
39
42
33
16
0
7
2
4
16
13
7
5
6
7
8
12
4
1
6
2
4
2
3
1
3
0
0
0
3
6
1
0
0
5
1
0


4
5
5
6
9
2
16
20
1
4
2
2
0
2
3
8
23
10
9
3
4
19
24
0
0
37
0
35
0
15
23
0
0
0
18
6
0
6
15
35
29
30
47
37
43
39
42
33
16
0
7
2
4
16
13
7
5
6
7
8
12
4
1
6
2
4
2
3
1
3
0
0
0
3
6
1
0
0
5
1
0


1
1
1
0
0
0
1
2
0
0
0
0
0
0
0
2
0
2
0
1
0
0
0
0
0
3
0
3
0
1
2
0
0
0
0
0
0
2
4
5
1
1
0
0
1
4
0
4
1
0
0
0
0
0
2
1
0
0
0
1
0
0
0
0
0
1
0
0
2
1
0
0
0
0
1
0
0
0
1
0
0


1
1
1
0
0
0
1
2
0
0
0
0
0
0
0
2
0
2
0
1
0
0
0
0
0
3
0
3
0
1
2
0
0
0
0
0
0
2
4
5
1
1
0
0
1
4
0
4
1
0
0
0
0
0
2
1
0
0
0
1
0
0
0
0
0
1
0
0
2
1
0
0
0
0
1
0
0
0
1
0
0


1
1
1
0
0
0
1
2
0
0
0
0
0
0
0
2
0
2
0
1
0
0
0
0
0
3
0
3
0
1
2
0
0
0
0
0
0
2
4
5
1
1
0
0
1
4
0
4
1
0
0
0
0
0
2
1
0
0
0
1
0
0
0
0
0
1
0
0
2
1
0
0
0
0
1
0
0
0
1
0
0


1
1
1
0
0
0
1
2
0
0
0
0
0
0
0
2
0
2
0
1
0
0
0
0
0
3
0
3
0
1
2
0
0
0
0
0
0
2
4
5
1
1
0
0
1
4
0
4
1
0
0
0
0
0
2
1
0
0
0
1
0
0
0
0
0
1
0
0
2
1
0
0
0
0
1
0
0
0
1
0
0


76
166
296
40
110
88
138
71
84
104
114
156
94
147
105
206
283
143
182
166
102
176
111
1
0
122
0
123
0
75
66
17
15
46
72
17
1
101
93
116
96
69
77
55
53
54
85
122
98
131
102
24
52
150
233
174
110
128
339
260
267
100
45
154
72
235
50
109
142
19
118
32
27
42
4
21
4
11
20
33
10


76
166
296
40
110
88
138
71
84
104
114
156
94
147
105
206
283
143
182
166
102
176
111
1
0
122
0
123
0
75
66
17
15
46
72
17
1
101
93
116
96
69
77
55
53
54
85
122
98
131
102
24
52
150
233
174
110
128
339
260
267
100
45
154
72
235
50
109
142
19
118
32
27
42
4
21
4
11
20
33
10


76
166
296
40
110
88
138
71
84
104
114
156
94
147
105
206
283
143
182
166
102
176
111
1
0
122
0
123
0
75
66
17
15
46
72
17
1
101
93
116
96
69
77
55
53
54
85
122
98
131
102
24
52
150
233
174
110
128
339
260
267
100
45
154
72
235
50
109
142
19
118
32
27
42
4
21
4
11
20
33
10


76
166
296
40
110
88
138
71
84
104
114
156
94
147
105
206
283
143
182
166
102
176
111
1
0
122
0
123
0
75
66
17
15
46
72
17
1
101
93
116
96
69
77
55
53
54
85
122
98
131
102
24
52
150
233
174
110
128
339
260
267
100
45
154
72
235
50
109
142
19
118
32
27
42
4
21
4
11
20
33
10


76
166
296
40
110
88
138
71
84
104
114
156
94
147
105
206
283
143
182
166
102
176
111
1
0
122
0
123
0
75
66
17
15
46
72
17
1
101
93
116
96
69
77
55
53
54
85
122
98
131
102
24
52
150
233
174
110
128
339
260
267
100
45
154
72
235
50
109
142
19
118
32
27
42
4
21
4
11
20
33
10


10
26
178
18
20
25
21
19
7
12
42
15
50
28
50
55
104
38
91
38
32
25
19
0
1
16
0
5
0
26
17
10
3
2
16
0
0
3
6
11
5
5
17
14
5
23
4
8
7
164
34
15
26
39
15
13
26
17
37
64
77
27
7
20
6
9
14
37
9
1
4
3
22
13
10
8
3
11
3
2
2


10
26
178
18
20
25
21
19
7
12
42
15
50
28
50
55
104
38
91
38
32
25
19
0
1
16
0
5
0
26
17
10
3
2
16
0
0
3
6
11
5
5
17
14
5
23
4
8
7
164
34
15
26
39
15
13
26
17
37
64
77
27
7
20
6
9
14
37
9
1
4
3
22
13
10
8
3
11
3
2
2


10
26
178
18
20
25
21
19
7
12
42
15
50
28
50
55
104
38
91
38
32
25
19
0
1
16
0
5
0
26
17
10
3
2
16
0
0
3
6
11
5
5
17
14
5
23
4
8
7
164
34
15
26
39
15
13
26
17
37
64
77
27
7
20
6
9
14
37
9
1
4
3
22
13
10
8
3
11
3
2
2


10
26
178
18
20
25
21
19
7
12
42
15
50
28
50
55
104
38
91
38
32
25
19
0
1
16
0
5
0
26
17
10
3
2
16
0
0
3
6
11
5
5
17
14
5
23
4
8
7
164
34
15
26
39
15
13
26
17
37
64
77
27
7
20
6
9
14
37
9
1
4
3
22
13
10
8
3
11
3
2
2


10
26
178
18
20
25
21
19
7
12
42
15
50
28
50
55
104
38
91
38
32
25
19
0
1
16
0
5
0
26
17
10
3
2
16
0
0
3
6
11
5
5
17
14
5
23
4
8
7
164
34
15
26
39
15
13
26
17
37
64
77
27
7
20
6
9
14
37
9
1
4
3
22
13
10
8
3
11
3
2
2


16
8
3
258
56
503
33
69
45
18
18
152
12
17
7
16
16
8
9
124
32
37
21
0
1
20
0
18
0
85
95
804
414
638
85
2857
182
4
14
57
20
35
49
92
96
64
10
30
101
0
47
1503
1246
52
41
140
444
207
68
16
104
66
862
10
504
99
1028
15
433
1253
326
3125
3278
1316
2769
3098
4002
2077
2437
487
817


1
0
1
27
3
38
1
4
5
1
1
6
0
1
0
0
1
0
2
15
0
0
0
0
0
7
0
0
0
6
5
15
0
21
4
252
33
0
2
14
2
3
1
2
6
5
3
0
5
0
1
48
22
3
0
7
11
0
1
0
3
1
34
0
61
16
39
0
12
12
20
113
26
23
13
75
14
15
35
3
23


1
0
1
27
3
38
1
4
5
1
1
6
0
1
0
0
1
0
2
15
0
0
0
0
0
7
0
0
0
6
5
15
0
21
4
252
33
0
2
14
2
3
1
2
6
5
3
0
5
0
1
48
22
3
0
7
11
0
1
0
3
1
34
0
61
16
39
0
12
12
20
113
26
23
13
75
14
15
35
3
23


1
0
1
27
3
38
1
4
5
1
1
6
0
1
0
0
1
0
2
15
0
0
0
0
0
7
0
0
0
6
5
15
0
21
4
252
33
0
2
14
2
3
1
2
6
5
3
0
5
0
1
48
22
3
0
7
11
0
1
0
3
1
34
0
61
16
39
0
12
12
20
113
26
23
13
75
14
15
35
3
23


1
0
1
27
3
38
1
4
5
1
1
6
0
1
0
0
1
0
2
15
0
0
0
0
0
7
0
0
0
6
5
15
0
21
4
252
33
0
2
14
2
3
1
2
6
5
3
0
5
0
1
48
22
3
0
7
11
0
1
0
3
1
34
0
61
16
39
0
12
12
20
113
26
23
13
75
14
15
35
3
23


0
0
0
0
0
0
0
0
0
0
0
0
0
0
0
0
0
0
0
0
0
0
0
0
0
0
0
0
0
0
0
0
0
0
0
0
0
0
0
0
0
0
0
0
0
1
0
0
0
0
0
1
0
0
0
0
0
0
0
0
0
0
2
0
0
0
0
0
0
0
0
0
0
0
1
0
0
0
0
0
0


0
0
0
0
0
0
0
0
0
0
0
0
0
0
0
0
0
0
0
0
0
0
0
0
0
0
0
0
0
0
0
0
0
0
0
0
0
0
0
0
0
0
0
0
0
1
0
0
0
0
0
1
0
0
0
0
0
0
0
0
0
0
2
0
0
0
0
0
0
0
0
0
0
0
1
0
0
0
0
0
0


0
0
0
0
0
0
0
0
0
0
0
0
0
0
0
0
0
0
0
0
0
0
0
0
0
0
0
0
0
0
0
0
0
0
0
0
0
0
0
0
0
0
0
0
0
1
0
0
0
0
0
1
0
0
0
0
0
0
0
0
0
0
2
0
0
0
0
0
0
0
0
0
0
0
1
0
0
0
0
0
0


0
0
0
0
0
0
0
0
0
0
0
0
0
0
0
0
0
0
0
0
0
0
0
0
0
0
0
0
0
0
0
0
0
0
0
0
0
0
0
0
0
0
0
0
0
1
0
0
0
0
0
1
0
0
0
0
0
0
0
0
0
0
2
0
0
0
0
0
0
0
0
0
0
0
1
0
0
0
0
0
0


0
0
0
4
0
6
0
0
0
0
0
0
0
0
0
0
0
0
0
2
0
0
0
0
0
0
0
0
0
1
1
0
0
0
0
0
0
0
0
0
0
0
0
0
0
1
0
0
0
0
0
17
19
0
0
0
0
2
0
0
1
1
1
0
4
0
6
0
5
2
0
42
80
36
77
39
157
118
32
6
22


0
0
0
4
0
6
0
0
0
0
0
0
0
0
0
0
0
0
0
2
0
0
0
0
0
0
0
0
0
1
1
0
0
0
0
0
0
0
0
0
0
0
0
0
0
1
0
0
0
0
0
17
19
0
0
0
0
2
0
0
1
1
1
0
4
0
6
0
5
2
0
42
80
36
77
39
157
118
32
6
22


0
0
0
4
0
6
0
0
0
0
0
0
0
0
0
0
0
0
0
2
0
0
0
0
0
0
0
0
0
1
1
0
0
0
0
0
0
0
0
0
0
0
0
0
0
1
0
0
0
0
0
17
19
0
0
0
0
2
0
0
1
1
1
0
4
0
6
0
5
2
0
42
80
36
77
39
157
118
32
6
22


0
0
0
4
0
6
0
0
0
0
0
0
0
0
0
0
0
0
0
2
0
0
0
0
0
0
0
0
0
1
1
0
0
0
0
0
0
0
0
0
0
0
0
0
0
1
0
0
0
0
0
17
19
0
0
0
0
2
0
0
1
1
1
0
4
0
6
0
5
2
0
42
80
36
77
39
157
118
32
6
22


0
0
0
1
0
1
0
0
0
0
0
0
0
0
0
0
0
0
0
0
0
0
0
0
0
0
0
0
0
4
0
3
0
6
0
1
0
0
0
0
0
0
0
0
0
0
0
0
0
0
0
0
0
0
0
0
0
0
0
0
0
0
1
0
0
0
0
0
0
2
0
0
1
0
1
0
0
3
0
0
0


0
0
0
1
0
1
0
0
0
0
0
0
0
0
0
0
0
0
0
0
0
0
0
0
0
0
0
0
0
4
0
3
0
6
0
1
0
0
0
0
0
0
0
0
0
0
0
0
0
0
0
0
0
0
0
0
0
0
0
0
0
0
1
0
0
0
0
0
0
2
0
0
1
0
1
0
0
3
0
0
0


0
0
0
1
0
1
0
0
0
0
0
0
0
0
0
0
0
0
0
0
0
0
0
0
0
0
0
0
0
4
0
3
0
6
0
1
0
0
0
0
0
0
0
0
0
0
0
0
0
0
0
0
0
0
0
0
0
0
0
0
0
0
1
0
0
0
0
0
0
2
0
0
1
0
1
0
0
3
0
0
0


0
0
0
1
0
1
0
0
0
0
0
0
0
0
0
0
0
0
0
0
0
0
0
0
0
0
0
0
0
4
0
3
0
6
0
1
0
0
0
0
0
0
0
0
0
0
0
0
0
0
0
0
0
0
0
0
0
0
0
0
0
0
1
0
0
0
0
0
0
2
0
0
1
0
1
0
0
3
0
0
0


0
1
0
50
17
48
5
4
23
5
11
139
2
11
2
3
1
2
4
49
3
5
2
0
0
1
0
2
0
8
22
47
31
30
33
231
0
3
4
11
4
9
13
16
18
24
1
8
24
0
13
155
88
17
20
29
65
31
13
6
17
10
227
1
103
25
163
4
44
89
91
141
45
78
33
180
25
41
67
39
24


0
1
0
50
17
48
5
4
23
5
11
139
2
11
2
3
1
2
4
49
3
5
2
0
0
1
0
2
0
8
22
47
31
30
33
231
0
3
4
11
4
9
13
16
18
24
1
8
24
0
13
155
88
17
20
29
65
31
13
6
17
10
227
1
103
25
163
4
44
89
91
141
45
78
33
180
25
41
67
39
24


0
1
0
50
17
48
5
4
23
5
11
139
2
11
2
3
1
2
4
49
3
5
2
0
0
1
0
2
0
8
22
47
31
30
33
231
0
3
4
11
4
9
13
16
18
24
1
8
24
0
13
155
88
17
20
29
65
31
13
6
17
10
227
1
103
25
163
4
44
89
91
141
45
78
33
180
25
41
67
39
24


0
1
0
50
17
48
5
4
23
5
11
139
2
11
2
3
1
2
4
49
3
5
2
0
0
1
0
2
0
8
22
47
31
30
33
231
0
3
4
11
4
9
13
16
18
24
1
8
24
0
13
155
88
17
20
29
65
31
13
6
17
10
227
1
103
25
163
4
44
89
91
141
45
78
33
180
25
41
67
39
24


5
2
0
4
3
21
8
5
8
5
5
2
2
2
1
3
1
5
0
3
7
7
5
0
0
3
0
16
0
14
6
74
0
88
9
21
0
0
0
2
5
9
10
24
30
8
4
9
8
0
14
152
32
3
14
21
32
24
11
6
19
18
14
0
28
13
37
6
8
43
21
340
254
100
164
199
406
171
307
56
40


5
2
0
4
3
21
8
5
8
5
5
2
2
2
1
3
1
5
0
3
7
7
5
0
0
3
0
16
0
14
6
74
0
88
9
21
0
0
0
2
5
9
10
24
30
8
4
9
8
0
14
152
32
3
14
21
32
24
11
6
19
18
14
0
28
13
37
6
8
43
21
340
254
100
164
199
406
171
307
56
40


5
2
0
4
3
21
8
4
8
5
5
2
2
2
1
3
1
5
0
3
4
7
5
0
0
3
0
16
0
12
5
74
0
72
9
21
0
0
0
2
5
9
10
19
18
5
4
9
7
0
14
115
26
3
13
19
14
23
10
6
19
17
13
0
28
13
36
5
7
38
15
333
237
80
163
190
401
162
208
44
39


5
2
0
4
3
21
8
4
8
5
5
2
2
2
1
3
1
5
0
3
4
7
5
0
0
3
0
16
0
12
5
74
0
72
9
21
0
0
0
2
5
9
10
19
18
5
4
9
7
0
14
115
26
3
13
19
14
23
10
6
19
17
13
0
28
13
36
5
7
38
15
333
237
80
163
190
401
162
208
44
39


0
0
0
0
0
0
0
1
0
0
0
0
0
0
0
0
0
0
0
0
3
0
0
0
0
0
0
0
0
2
0
0
0
16
0
0
0
0
0
0
0
0
0
3
12
3
0
0
1
0
0
37
6
0
1
2
16
1
1
0
0
1
1
0
0
0
1
1
1
5
4
5
13
20
1
9
5
9
99
12
1


0
0
0
0
0
0
0
1
0
0
0
0
0
0
0
0
0
0
0
0
3
0
0
0
0
0
0
0
0
2
0
0
0
16
0
0
0
0
0
0
0
0
0
3
12
3
0
0
1
0
0
37
6
0
1
2
16
1
1
0
0
1
1
0
0
0
1
1
1
5
4
5
13
20
1
9
5
9
99
12
1


0
0
0
0
0
0
0
0
0
0
0
0
0
0
0
0
0
0
0
0
0
0
0
0
0
0
0
0
0
0
1
0
0
0
0
0
0
0
0
0
0
0
0
2
0
0
0
0
0
0
0
0
0
0
0
0
2
0
0
0
0
0
0
0
0
0
0
0
0
0
2
2
4
0
0
0
0
0
0
0
0


0
0
0
0
0
0
0
0
0
0
0
0
0
0
0
0
0
0
0
0
0
0
0
0
0
0
0
0
0
0
1
0
0
0
0
0
0
0
0
0
0
0
0
2
0
0
0
0
0
0
0
0
0
0
0
0
2
0
0
0
0
0
0
0
0
0
0
0
0
0
2
2
4
0
0
0
0
0
0
0
0


2
0
0
16
4
20
1
2
0
1
0
1
1
1
0
1
1
0
0
4
0
2
2
0
0
1
0
0
0
1
2
3
6
0
0
7
0
1
2
1
0
1
1
2
1
4
1
3
2
0
0
4
11
1
2
0
4
5
0
1
2
0
14
0
6
2
6
1
1
0
1
4
2
4
6
5
0
3
6
1
2


2
0
0
16
4
20
1
2
0
1
0
1
1
1
0
1
1
0
0
4
0
2
2
0
0
1
0
0
0
1
2
3
6
0
0
7
0
1
2
1
0
1
1
2
1
4
1
3
2
0
0
4
11
1
2
0
4
5
0
1
2
0
14
0
6
2
6
1
1
0
1
4
2
4
6
5
0
3
6
1
2


2
0
0
16
4
20
1
2
0
1
0
1
1
1
0
1
1
0
0
4
0
2
2
0
0
1
0
0
0
1
2
3
6
0
0
7
0
1
2
1
0
1
1
2
1
4
1
3
2
0
0
4
11
1
2
0
4
5
0
1
2
0
14
0
6
2
6
1
1
0
1
4
2
4
6
5
0
3
6
1
2


2
0
0
16
4
20
1
2
0
1
0
1
1
1
0
1
1
0
0
4
0
2
2
0
0
1
0
0
0
1
2
3
6
0
0
7
0
1
2
1
0
1
1
2
1
4
1
3
2
0
0
4
11
1
2
0
4
5
0
1
2
0
14
0
6
2
6
1
1
0
1
4
2
4
6
5
0
3
6
1
2


6
1
2
137
16
333
14
41
8
3
1
2
7
2
2
7
11
0
0
43
18
16
3
0
1
4
0
0
0
38
33
645
376
383
18
2158
148
0
0
20
3
11
10
25
16
11
1
10
51
0
17
957
963
23
4
62
280
124
40
3
45
25
552
7
183
27
744
2
323
1046
162
2041
2150
781
1313
2019
1736
1423
1606
327
570


6
1
2
136
16
318
14
41
8
3
1
2
7
2
2
5
10
0
0
42
17
16
2
0
1
4
0
0
0
36
32
633
376
348
13
2150
148
0
0
20
1
7
10
25
15
11
1
10
50
0
17
916
947
22
3
62
278
122
37
3
43
25
548
6
183
26
737
1
319
1037
159
1987
2072
756
1241
2014
1620
1403
1533
324
568


6
1
2
134
16
316
14
41
8
3
1
2
7
2
2
5
10
0
0
42
17
16
2
0
1
4
0
0
0
36
31
633
376
348
13
2149
148
0
0
20
1
7
10
25
15
11
1
10
50
0
17
909
941
22
3
62
278
122
37
3
43
25
545
6
181
26
733
1
319
1037
159
1980
2060
754
1232
2011
1567
1396
1527
323
568


6
1
2
134
16
316
14
41
8
3
1
2
7
2
2
5
10
0
0
42
17
16
2
0
1
4
0
0
0
36
31
633
376
348
13
2149
148
0
0
20
1
7
10
25
15
11
1
10
50
0
17
909
941
22
3
62
278
122
37
3
43
25
545
6
181
26
733
1
319
1037
159
1980
2060
754
1232
2011
1567
1396
1527
323
568


0
0
0
2
0
2
0
0
0
0
0
0
0
0
0
0
0
0
0
0
0
0
0
0
0
0
0
0
0
0
1
0
0
0
0
1
0
0
0
0
0
0
0
0
0
0
0
0
0
0
0
7
6
0
0
0
0
0
0
0
0
0
3
0
2
0
4
0
0
0
0
7
12
2
9
3
53
7
6
1
0


0
0
0
2
0
2
0
0
0
0
0
0
0
0
0
0
0
0
0
0
0
0
0
0
0
0
0
0
0
0
1
0
0
0
0
1
0
0
0
0
0
0
0
0
0
0
0
0
0
0
0
7
6
0
0
0
0
0
0
0
0
0
3
0
2
0
4
0
0
0
0
7
12
2
9
3
53
7
6
1
0


0
0
0
1
0
15
0
0
0
0
0
0
0
0
0
2
1
0
0
1
1
0
1
0
0
0
0
0
0
2
1
12
0
35
5
8
0
0
0
0
2
4
0
0
1
0
0
0
1
0
0
41
16
1
1
0
2
2
3
0
2
0
4
1
0
1
7
1
4
9
3
54
78
25
72
5
116
20
73
3
2


0
0
0
1
0
15
0
0
0
0
0
0
0
0
0
2
1
0
0
1
1
0
1
0
0
0
0
0
0
2
1
12
0
35
5
8
0
0
0
0
2
4
0
0
1
0
0
0
1
0
0
41
16
1
1
0
2
2
3
0
2
0
4
1
0
1
7
1
4
9
3
54
78
25
72
5
116
20
73
3
2


0
0
0
1
0
15
0
0
0
0
0
0
0
0
0
2
1
0
0
1
1
0
1
0
0
0
0
0
0
2
1
12
0
35
5
8
0
0
0
0
2
4
0
0
1
0
0
0
1
0
0
41
16
1
1
0
2
2
3
0
2
0
4
1
0
1
7
1
4
9
3
54
78
25
72
5
116
20
73
3
2


2
4
0
19
13
36
4
13
1
3
0
2
0
0
2
2
1
1
3
8
4
7
9
0
0
4
0
0
0
13
26
17
1
110
21
187
1
0
6
9
6
2
14
23
25
10
0
0
11
0
2
169
111
5
1
21
52
21
3
0
17
11
17
2
119
16
33
2
40
59
31
444
720
294
1161
581
1664
303
384
55
136


2
4
0
19
13
36
4
13
1
3
0
2
0
0
2
2
1
1
3
8
4
7
9
0
0
4
0
0
0
13
26
17
1
110
21
187
1
0
6
9
6
2
14
23
25
10
0
0
11
0
2
169
111
5
1
21
52
21
3
0
17
11
17
2
119
16
33
2
40
59
31
444
720
294
1161
581
1664
303
384
55
136


2
4
0
19
13
36
4
13
1
3
0
2
0
0
2
2
1
1
3
8
4
7
9
0
0
4
0
0
0
13
26
17
1
110
21
187
1
0
6
9
6
2
14
23
25
10
0
0
11
0
2
169
111
5
1
21
52
21
3
0
17
11
17
2
119
16
33
2
40
59
31
444
720
294
1161
581
1664
303
384
55
136


2
4
0
19
13
36
4
13
1
3
0
2
0
0
2
2
1
1
3
8
4
7
9
0
0
4
0
0
0
13
26
17
1
110
21
187
1
0
6
9
6
2
14
23
25
10
0
0
11
0
2
169
111
5
1
21
52
21
3
0
17
11
17
2
119
16
33
2
40
59
31
444
720
294
1161
581
1664
303
384
55
136


28
7
52
23
28
38
13
12
1
1
1
0
0
2
1
4
5
0
2
11
3
8
7
0
5
0
0
2
0
5
2
0
0
7
2
1
0
0
5
0
2
4
9
8
15
7
3
18
1
0
4
10
27
18
1
19
24
26
6
13
8
0
1
4
12
8
47
2
80
94
7
2
2
1
0
6
2
3
1
2
2


28
7
52
23
28
38
13
12
1
1
1
0
0
2
1
4
5
0
2
11
3
8
7
0
5
0
0
2
0
5
2
0
0
7
2
1
0
0
5
0
2
4
9
8
15
7
3
18
1
0
4
10
27
18
1
19
24
26
6
13
8
0
1
4
12
8
47
2
80
94
7
2
2
1
0
6
2
3
1
2
2


28
7
52
23
28
38
13
12
1
1
1
0
0
2
1
4
5
0
2
11
3
8
7
0
5
0
0
2
0
5
2
0
0
7
2
1
0
0
5
0
2
4
9
8
15
7
3
18
1
0
4
10
27
18
1
19
24
26
6
13
8
0
1
4
12
8
47
2
80
94
7
2
2
1
0
6
2
3
1
2
2


28
7
52
23
28
38
13
12
1
1
1
0
0
2
1
4
5
0
2
11
3
8
7
0
5
0
0
2
0
5
2
0
0
7
2
1
0
0
5
0
2
4
9
8
15
7
3
18
1
0
4
10
27
18
1
19
24
26
6
13
8
0
1
4
12
8
47
2
80
94
7
2
2
1
0
6
2
3
1
2
2


28
7
52
23
28
38
13
12
1
1
1
0
0
2
1
4
5
0
2
11
3
8
7
0
5
0
0
2
0
5
2
0
0
7
2
1
0
0
5
0
2
4
9
8
15
7
3
18
1
0
4
10
27
18
1
19
24
26
6
13
8
0
1
4
12
8
47
2
80
94
7
2
2
1
0
6
2
3
1
2
2


35
32
66
10
24
18
63
44
31
18
53
35
47
40
14
24
49
33
54
29
57
27
41
0
0
15
0
16
0
19
19
8
23
0
27
16
0
28
18
13
21
14
29
19
16
14
22
10
23
0
64
10
23
91
43
64
30
40
64
102
91
63
10
68
35
52
27
40
32
10
27
6
10
12
0
2
1
5
3
6
2


35
32
66
10
24
18
63
44
31
18
53
35
47
40
14
24
49
33
54
29
57
27
41
0
0
15
0
16
0
19
19
8
23
0
27
16
0
28
18
13
21
14
29
19
16
14
22
10
23
0
64
10
23
91
43
64
30
40
64
102
91
63
10
68
35
52
27
40
32
10
27
6
10
12
0
2
1
5
3
6
2


35
32
66
10
24
18
63
44
31
18
53
35
47
40
14
24
49
33
54
29
57
27
41
0
0
15
0
16
0
19
19
8
23
0
27
16
0
28
18
13
21
14
29
19
16
14
22
10
23
0
64
10
23
91
43
64
30
40
64
102
91
63
10
68
35
52
27
40
32
10
27
6
10
12
0
2
1
5
3
6
2


35
32
66
10
24
18
63
44
31
18
53
35
47
40
14
24
49
33
54
29
57
27
41
0
0
15
0
16
0
19
19
8
23
0
27
16
0
28
18
13
21
14
29
19
16
14
22
10
23
0
64
10
23
91
43
64
30
40
64
102
91
63
10
68
35
52
27
40
32
10
27
6
10
12
0
2
1
5
3
6
2


35
32
66
10
24
18
63
44
31
18
53
35
47
40
14
24
49
33
54
29
57
27
41
0
0
15
0
16
0
19
19
8
23
0
27
16
0
28
18
13
21
14
29
19
16
14
22
10
23
0
64
10
23
91
43
64
30
40
64
102
91
63
10
68
35
52
27
40
32
10
27
6
10
12
0
2
1
5
3
6
2


0
0
0
0
0
0
0
0
0
0
1
0
0
0
0
0
0
0
0
0
1
0
0
0
0
0
0
0
0
0
0
0
0
0
0
0
0
0
0
0
0
0
0
0
0
0
0
0
0
0
0
0
0
0
0
0
0
0
1
0
0
0
0
2
1
0
0
1
0
0
0
0
0
0
0
0
0
0
0
0
0


0
0
0
0
0
0
0
0
0
0
1
0
0
0
0
0
0
0
0
0
1
0
0
0
0
0
0
0
0
0
0
0
0
0
0
0
0
0
0
0
0
0
0
0
0
0
0
0
0
0
0
0
0
0
0
0
0
0
1
0
0
0
0
2
1
0
0
1
0
0
0
0
0
0
0
0
0
0
0
0
0


0
0
0
0
0
0
0
0
0
0
1
0
0
0
0
0
0
0
0
0
1
0
0
0
0
0
0
0
0
0
0
0
0
0
0
0
0
0
0
0
0
0
0
0
0
0
0
0
0
0
0
0
0
0
0
0
0
0
1
0
0
0
0
2
1
0
0
1
0
0
0
0
0
0
0
0
0
0
0
0
0


0
0
0
0
0
0
0
0
0
0
1
0
0
0
0
0
0
0
0
0
1
0
0
0
0
0
0
0
0
0
0
0
0
0
0
0
0
0
0
0
0
0
0
0
0
0
0
0
0
0
0
0
0
0
0
0
0
0
1
0
0
0
0
2
1
0
0
1
0
0
0
0
0
0
0
0
0
0
0
0
0


0
0
0
0
0
0
0
0
0
0
1
0
0
0
0
0
0
0
0
0
1
0
0
0
0
0
0
0
0
0
0
0
0
0
0
0
0
0
0
0
0
0
0
0
0
0
0
0
0
0
0
0
0
0
0
0
0
0
1
0
0
0
0
2
1
0
0
1
0
0
0
0
0
0
0
0
0
0
0
0
0


0
1
0
0
0
0
0
0
0
0
0
0
0
0
0
0
0
0
0
0
1
0
0
0
0
0
0
0
0
0
0
0
0
0
0
0
0
0
1
0
0
0
0
0
0
0
0
0
0
0
0
1
0
0
0
0
2
0
0
0
0
0
0
0
0
0
1
0
0
0
1
1
0
0
0
0
1
0
1
0
0


0
1
0
0
0
0
0
0
0
0
0
0
0
0
0
0
0
0
0
0
1
0
0
0
0
0
0
0
0
0
0
0
0
0
0
0
0
0
1
0
0
0
0
0
0
0
0
0
0
0
0
1
0
0
0
0
2
0
0
0
0
0
0
0
0
0
1
0
0
0
1
1
0
0
0
0
1
0
1
0
0


0
1
0
0
0
0
0
0
0
0
0
0
0
0
0
0
0
0
0
0
1
0
0
0
0
0
0
0
0
0
0
0
0
0
0
0
0
0
1
0
0
0
0
0
0
0
0
0
0
0
0
1
0
0
0
0
2
0
0
0
0
0
0
0
0
0
1
0
0
0
1
1
0
0
0
0
1
0
1
0
0


0
1
0
0
0
0
0
0
0
0
0
0
0
0
0
0
0
0
0
0
1
0
0
0
0
0
0
0
0
0
0
0
0
0
0
0
0
0
1
0
0
0
0
0
0
0
0
0
0
0
0
1
0
0
0
0
2
0
0
0
0
0
0
0
0
0
1
0
0
0
1
1
0
0
0
0
1
0
1
0
0


0
1
0
0
0
0
0
0
0
0
0
0
0
0
0
0
0
0
0
0
1
0
0
0
0
0
0
0
0
0
0
0
0
0
0
0
0
0
1
0
0
0
0
0
0
0
0
0
0
0
0
1
0
0
0
0
2
0
0
0
0
0
0
0
0
0
1
0
0
0
1
1
0
0
0
0
1
0
1
0
0


1
0
0
0
0
0
0
0
0
0
0
0
0
0
0
0
0
0
0
0
0
0
0
0
0
0
0
0
0
0
0
0
0
0
0
0
0
0
0
0
0
0
0
0
0
0
0
0
0
0
0
0
0
0
0
0
0
0
0
0
0
0
0
0
0
0
0
0
0
0
0
0
0
0
0
0
0
0
0
0
0


1
0
0
0
0
0
0
0
0
0
0
0
0
0
0
0
0
0
0
0
0
0
0
0
0
0
0
0
0
0
0
0
0
0
0
0
0
0
0
0
0
0
0
0
0
0
0
0
0
0
0
0
0
0
0
0
0
0
0
0
0
0
0
0
0
0
0
0
0
0
0
0
0
0
0
0
0
0
0
0
0


1
0
0
0
0
0
0
0
0
0
0
0
0
0
0
0
0
0
0
0
0
0
0
0
0
0
0
0
0
0
0
0
0
0
0
0
0
0
0
0
0
0
0
0
0
0
0
0
0
0
0
0
0
0
0
0
0
0
0
0
0
0
0
0
0
0
0
0
0
0
0
0
0
0
0
0
0
0
0
0
0


1
0
0
0
0
0
0
0
0
0
0
0
0
0
0
0
0
0
0
0
0
0
0
0
0
0
0
0
0
0
0
0
0
0
0
0
0
0
0
0
0
0
0
0
0
0
0
0
0
0
0
0
0
0
0
0
0
0
0
0
0
0
0
0
0
0
0
0
0
0
0
0
0
0
0
0
0
0
0
0
0


1
0
0
0
0
0
0
0
0
0
0
0
0
0
0
0
0
0
0
0
0
0
0
0
0
0
0
0
0
0
0
0
0
0
0
0
0
0
0
0
0
0
0
0
0
0
0
0
0
0
0
0
0
0
0
0
0
0
0
0
0
0
0
0
0
0
0
0
0
0
0
0
0
0
0
0
0
0
0
0
0


121
152
167
264
204
223
181
173
129
159
105
195
90
97
126
143
267
203
117
251
158
152
166
4
66
133
100
176
0
189
229
179
156
373
117
154
0
82
132
193
180
128
190
205
266
219
210
199
249
55
119
187
193
170
98
124
151
131
154
152
237
122
83
113
270
175
230
84
185
193
111
261
237
253
114
288
171
174
139
72
124


18
28
22
8
29
13
50
28
28
6
15
25
5
13
25
20
20
34
7
35
32
15
30
0
0
6
0
32
0
24
19
0
0
0
9
5
0
27
35
14
18
18
35
33
18
19
37
42
54
24
19
8
8
33
14
14
4
11
18
35
47
10
1
24
4
16
4
6
9
6
6
0
0
0
0
0
0
0
0
0
0


18
28
22
8
29
13
50
28
28
6
15
25
5
13
25
20
20
34
7
35
32
15
30
0
0
6
0
32
0
24
19
0
0
0
9
5
0
27
35
14
18
18
35
33
18
19
37
42
54
24
19
8
8
33
14
14
4
11
18
35
47
10
1
24
4
16
4
6
9
6
6
0
0
0
0
0
0
0
0
0
0


18
28
22
8
29
13
50
28
28
6
15
25
5
13
25
20
20
34
7
35
32
15
30
0
0
6
0
32
0
24
19
0
0
0
9
5
0
27
35
14
18
18
35
33
18
19
37
42
54
24
19
8
8
33
14
14
4
11
18
35
47
10
1
24
4
16
4
6
9
6
6
0
0
0
0
0
0
0
0
0
0


18
28
22
8
29
13
50
28
28
6
15
25
5
13
25
20
20
34
7
35
32
15
30
0
0
6
0
32
0
24
19
0
0
0
9
5
0
27
35
14
18
18
35
33
18
19
37
42
54
24
19
8
8
33
14
14
4
11
18
35
47
10
1
24
4
16
4
6
9
6
6
0
0
0
0
0
0
0
0
0
0


36
71
75
88
41
49
61
61
62
78
58
65
36
41
38
70
110
72
60
115
64
61
70
0
4
70
100
92
0
28
49
48
29
47
24
17
0
39
54
72
74
30
40
51
31
20
101
59
97
0
58
25
36
84
41
52
63
45
66
71
77
65
9
58
166
61
14
45
39
28
28
5
13
27
1
9
3
17
6
17
15


0
2
0
0
0
0
0
0
0
0
0
0
0
0
0
0
0
0
0
0
2
1
0
0
0
0
0
8
0
0
0
0
0
0
0
0
0
0
2
7
4
1
1
1
1
0
6
2
0
0
0
0
0
0
0
0
0
0
0
1
2
0
0
0
0
0
0
0
0
0
0
0
0
0
0
0
0
0
0
0
0


0
2
0
0
0
0
0
0
0
0
0
0
0
0
0
0
0
0
0
0
2
1
0
0
0
0
0
8
0
0
0
0
0
0
0
0
0
0
2
7
4
1
1
1
1
0
6
2
0
0
0
0
0
0
0
0
0
0
0
1
2
0
0
0
0
0
0
0
0
0
0
0
0
0
0
0
0
0
0
0
0


0
2
0
0
0
0
0
0
0
0
0
0
0
0
0
0
0
0
0
0
2
1
0
0
0
0
0
8
0
0
0
0
0
0
0
0
0
0
2
7
4
1
1
1
1
0
6
2
0
0
0
0
0
0
0
0
0
0
0
1
2
0
0
0
0
0
0
0
0
0
0
0
0
0
0
0
0
0
0
0
0


18
44
34
12
18
21
26
31
11
10
8
23
11
14
11
14
18
27
15
20
15
7
32
0
0
10
0
5
0
8
13
0
14
0
13
3
0
7
19
11
9
4
16
8
9
8
9
7
13
0
17
6
16
44
19
31
29
20
29
22
24
13
4
19
16
23
5
15
21
9
16
4
11
7
1
2
2
12
1
6
4


18
44
34
12
18
21
26
31
11
10
8
23
11
14
11
14
18
27
15
20
15
7
32
0
0
10
0
5
0
8
13
0
14
0
13
3
0
7
19
11
9
4
16
8
9
8
9
7
13
0
17
6
16
44
19
31
29
20
29
22
24
13
4
19
16
23
5
15
21
9
16
4
11
7
1
2
2
12
1
6
4


18
44
34
12
18
21
26
31
11
10
8
23
11
14
11
14
18
27
15
20
15
7
32
0
0
10
0
5
0
8
13
0
14
0
13
3
0
7
19
11
9
4
16
8
9
8
9
7
13
0
17
6
16
44
19
31
29
20
29
22
24
13
4
19
16
23
5
15
21
9
16
4
11
7
1
2
2
12
1
6
4


16
23
35
62
18
27
28
28
46
66
25
33
16
16
26
54
86
43
25
95
44
51
35
0
4
59
100
78
0
20
36
48
15
47
11
14
0
26
33
54
59
25
23
40
21
12
84
42
82
0
23
19
17
38
20
20
30
24
32
43
36
40
4
30
144
37
5
21
15
19
12
1
2
19
0
6
0
5
4
11
10


16
23
35
62
18
27
28
28
46
66
25
33
16
16
26
54
86
43
25
95
44
51
35
0
4
59
100
78
0
20
36
48
15
47
11
14
0
26
33
54
59
25
23
40
21
12
84
42
82
0
23
19
17
38
20
20
30
24
32
43
36
40
4
30
144
37
5
21
15
19
12
1
2
19
0
6
0
5
4
11
10


16
23
35
62
18
27
28
28
46
66
25
33
16
16
26
54
86
43
25
95
44
51
35
0
4
59
100
78
0
20
36
48
15
47
11
14
0
26
33
54
59
25
23
40
21
12
84
42
82
0
23
19
17
38
20
20
30
24
32
43
36
40
4
30
144
37
5
21
15
19
12
1
2
19
0
6
0
5
4
11
10


2
2
6
14
5
1
7
2
5
2
25
9
9
11
1
2
6
2
20
0
3
2
3
0
0
1
0
1
0
0
0
0
0
0
0
0
0
6
0
0
2
0
0
2
0
0
2
8
2
0
18
0
3
2
2
1
4
1
5
5
15
12
1
9
6
1
4
9
3
0
0
0
0
1
0
1
1
0
1
0
1


2
2
6
14
5
1
7
2
5
2
25
9
9
11
1
2
6
2
20
0
3
2
3
0
0
1
0
1
0
0
0
0
0
0
0
0
0
6
0
0
2
0
0
2
0
0
2
8
2
0
18
0
3
2
2
1
4
1
5
5
15
12
1
9
6
1
4
9
3
0
0
0
0
1
0
1
1
0
1
0
1


2
2
6
14
5
1
7
2
5
2
25
9
9
11
1
2
6
2
20
0
3
2
3
0
0
1
0
1
0
0
0
0
0
0
0
0
0
6
0
0
2
0
0
2
0
0
2
8
2
0
18
0
3
2
2
1
4
1
5
5
15
12
1
9
6
1
4
9
3
0
0
0
0
1
0
1
1
0
1
0
1


66
52
70
168
133
161
69
84
38
74
31
104
47
43
63
53
135
94
47
99
58
76
63
4
62
53
0
52
0
137
161
131
127
325
82
131
0
16
42
107
88
75
114
120
216
178
71
98
94
30
42
152
149
53
43
56
83
75
70
43
108
47
72
28
100
95
212
33
137
159
76
252
222
226
113
279
167
157
133
55
106


47
30
61
96
97
71
54
57
26
67
25
97
46
37
51
41
117
87
39
63
32
58
51
3
0
46
0
33
0
107
132
115
71
218
68
36
0
10
29
80
76
59
59
87
176
126
47
68
78
13
34
84
70
37
37
39
51
42
32
31
84
33
23
22
42
74
100
31
74
80
60
71
60
176
74
71
94
92
90
23
58


47
30
61
96
97
71
54
57
26
67
25
97
46
37
51
41
117
87
39
63
32
58
51
3
0
46
0
33
0
107
132
115
71
218
68
36
0
10
29
80
76
59
59
87
176
126
47
68
78
13
34
84
70
37
37
39
51
42
32
31
84
33
23
22
42
74
100
31
74
80
60
71
60
176
74
71
94
92
90
23
58


47
30
61
96
97
71
54
57
26
67
25
97
46
37
51
41
117
87
39
63
32
58
51
3
0
46
0
33
0
107
132
115
71
218
68
36
0
10
29
80
76
59
59
87
176
126
47
68
78
13
34
84
70
37
37
39
51
42
32
31
84
33
23
22
42
74
100
31
74
80
60
71
60
176
74
71
94
92
90
23
58


1
0
0
3
0
0
2
2
2
0
0
1
0
0
1
1
4
0
0
0
0
1
1
0
10
0
0
10
0
2
1
0
0
21
0
0
0
1
1
0
0
0
7
3
4
3
2
4
3
0
1
3
37
0
0
1
2
1
0
2
2
0
2
0
3
1
66
1
45
35
2
6
2
9
4
4
5
4
3
1
4


1
0
0
3
0
0
2
2
2
0
0
1
0
0
1
1
4
0
0
0
0
1
1
0
10
0
0
10
0
2
1
0
0
21
0
0
0
1
1
0
0
0
7
3
4
3
2
4
3
0
1
3
37
0
0
1
2
1
0
2
2
0
2
0
3
1
66
1
45
35
2
6
2
9
4
4
5
4
3
1
4


1
0
0
3
0
0
2
2
2
0
0
1
0
0
1
1
4
0
0
0
0
1
1
0
10
0
0
10
0
2
1
0
0
21
0
0
0
1
1
0
0
0
7
3
4
3
2
4
3
0
1
3
37
0
0
1
2
1
0
2
2
0
2
0
3
1
66
1
45
35
2
6
2
9
4
4
5
4
3
1
4


18
22
9
69
36
90
13
25
10
7
6
6
1
6
11
11
14
7
8
36
26
17
11
1
52
7
0
9
0
28
28
16
56
86
14
95
0
5
12
27
12
16
48
30
36
49
22
26
13
17
7
65
42
16
6
16
30
32
38
10
22
14
47
6
55
20
46
1
18
44
14
175
160
41
35
204
68
61
40
31
44


18
22
9
69
36
90
13
25
10
7
6
6
1
6
11
11
14
7
8
36
26
17
11
1
52
7
0
9
0
28
28
16
56
86
14
95
0
5
12
27
12
16
48
30
36
49
22
26
13
17
7
65
42
16
6
16
30
32
38
10
22
14
47
6
55
20
46
1
18
44
14
175
160
41
35
204
68
61
40
31
44


18
22
9
69
36
90
13
25
10
7
6
6
1
6
11
11
14
7
8
36
26
17
11
1
52
7
0
9
0
28
28
16
56
86
14
95
0
5
12
27
12
16
48
30
36
49
22
26
13
17
7
65
42
16
6
16
30
32
38
10
22
14
47
6
55
20
46
1
18
44
14
175
160
41
35
204
68
61
40
31
44


1
1
0
0
1
0
1
0
1
1
1
1
2
0
0
0
2
3
3
2
4
0
3
0
0
4
0
0
0
0
0
0
0
1
2
1
0
0
1
0
0
5
1
1
1
2
1
0
4
1
0
2
0
0
0
2
1
0
0
3
5
0
1
3
0
3
0
0
0
0
1
4
2
0
0
0
1
0
0
0
3


1
1
0
0
1
0
1
0
1
1
1
1
2
0
0
0
2
3
3
2
4
0
3
0
0
4
0
0
0
0
0
0
0
1
2
1
0
0
1
0
0
5
1
1
1
2
1
0
4
1
0
2
0
0
0
2
1
0
0
3
5
0
1
3
0
3
0
0
0
0
1
4
2
0
0
0
1
0
0
0
3


1
1
0
0
1
0
1
0
1
1
1
1
2
0
0
0
2
3
3
2
4
0
3
0
0
4
0
0
0
0
0
0
0
1
2
1
0
0
1
0
0
5
1
1
1
2
1
0
4
1
0
2
0
0
0
2
1
0
0
3
5
0
1
3
0
3
0
0
0
0
1
4
2
0
0
0
1
0
0
0
3


1
1
0
0
1
0
1
0
1
1
1
1
2
0
0
0
2
3
3
2
4
0
3
0
0
4
0
0
0
0
0
0
0
1
2
1
0
0
1
0
0
5
1
1
1
2
1
0
4
1
0
2
0
0
0
2
1
0
0
3
5
0
1
3
0
3
0
0
0
0
1
4
2
0
0
0
1
0
0
0
3


8
14
24
7
9
10
13
12
5
4
7
12
3
5
8
10
17
8
18
15
5
3
6
0
0
0
0
5
0
7
5
0
0
1
5
4
0
6
5
3
4
6
11
1
4
2
5
0
8
0
5
4
13
14
16
10
10
4
9
13
13
9
1
9
10
5
5
6
11
8
7
9
5
5
0
2
6
5
4
1
2


8
14
22
7
9
10
13
12
5
4
7
12
3
5
6
10
17
8
16
15
5
3
6
0
0
0
0
5
0
7
5
0
0
1
5
4
0
6
4
3
2
6
11
1
4
2
5
0
8
0
5
4
13
14
16
10
10
4
9
13
13
9
1
9
10
5
5
6
11
8
7
9
5
5
0
2
6
5
4
1
2


8
14
22
7
9
10
13
12
5
4
7
12
3
5
6
10
17
8
16
15
5
3
6
0
0
0
0
5
0
7
5
0
0
1
5
4
0
6
4
3
2
6
11
1
4
2
5
0
8
0
5
4
13
14
16
10
10
4
9
13
13
9
1
9
10
5
5
6
11
8
7
9
5
5
0
2
6
5
4
1
2


8
14
22
7
9
10
13
12
5
4
7
12
3
5
6
10
17
8
16
15
5
3
6
0
0
0
0
5
0
7
5
0
0
1
5
4
0
6
4
3
2
6
11
1
4
2
5
0
8
0
5
4
13
14
16
10
10
4
9
13
13
9
1
9
10
5
5
6
11
8
7
9
5
5
0
2
6
5
4
1
2


8
14
22
7
9
10
13
12
5
4
7
12
3
5
6
10
17
8
16
15
5
3
6
0
0
0
0
5
0
7
5
0
0
1
5
4
0
6
4
3
2
6
11
1
4
2
5
0
8
0
5
4
13
14
16
10
10
4
9
13
13
9
1
9
10
5
5
6
11
8
7
9
5
5
0
2
6
5
4
1
2


0
0
2
0
0
0
0
0
0
0
0
0
0
0
2
0
0
0
2
0
0
0
0
0
0
0
0
0
0
0
0
0
0
0
0
0
0
0
1
0
2
0
0
0
0
0
0
0
0
0
0
0
0
0
0
0
0
0
0
0
0
0
0
0
0
0
0
0
0
0
0
0
0
0
0
0
0
0
0
0
0


0
0
2
0
0
0
0
0
0
0
0
0
0
0
2
0
0
0
2
0
0
0
0
0
0
0
0
0
0
0
0
0
0
0
0
0
0
0
1
0
2
0
0
0
0
0
0
0
0
0
0
0
0
0
0
0
0
0
0
0
0
0
0
0
0
0
0
0
0
0
0
0
0
0
0
0
0
0
0
0
0


0
0
2
0
0
0
0
0
0
0
0
0
0
0
2
0
0
0
2
0
0
0
0
0
0
0
0
0
0
0
0
0
0
0
0
0
0
0
1
0
2
0
0
0
0
0
0
0
0
0
0
0
0
0
0
0
0
0
0
0
0
0
0
0
0
0
0
0
0
0
0
0
0
0
0
0
0
0
0
0
0


0
0
2
0
0
0
0
0
0
0
0
0
0
0
2
0
0
0
2
0
0
0
0
0
0
0
0
0
0
0
0
0
0
0
0
0
0
0
1
0
2
0
0
0
0
0
0
0
0
0
0
0
0
0
0
0
0
0
0
0
0
0
0
0
0
0
0
0
0
0
0
0
0
0
0
0
0
0
0
0
0


1
10
17
6
12
8
11
3
14
8
22
25
21
11
15
16
24
28
14
12
19
35
29
0
0
55
54
23
0
8
18
57
26
15
6
19
0
21
22
31
22
12
12
29
13
7
25
25
18
0
15
12
11
24
17
19
10
12
21
21
11
9
9
11
9
5
2
16
7
2
9
6
9
8
2
5
6
7
6
10
2


0
0
0
1
0
0
0
0
3
0
0
5
0
0
0
0
0
0
0
0
0
0
0
0
0
0
0
2
0
0
0
37
5
0
0
4
0
0
0
0
0
0
0
0
0
2
0
0
0
0
0
0
0
0
0
0
0
0
0
0
0
0
3
0
0
0
0
0
0
0
0
0
0
0
0
0
0
0
0
0
0


0
0
0
1
0
0
0
0
3
0
0
5
0
0
0
0
0
0
0
0
0
0
0
0
0
0
0
2
0
0
0
37
5
0
0
4
0
0
0
0
0
0
0
0
0
2
0
0
0
0
0
0
0
0
0
0
0
0
0
0
0
0
3
0
0
0
0
0
0
0
0
0
0
0
0
0
0
0
0
0
0


0
0
0
1
0
0
0
0
3
0
0
5
0
0
0
0
0
0
0
0
0
0
0
0
0
0
0
2
0
0
0
37
5
0
0
4
0
0
0
0
0
0
0
0
0
2
0
0
0
0
0
0
0
0
0
0
0
0
0
0
0
0
3
0
0
0
0
0
0
0
0
0
0
0
0
0
0
0
0
0
0


0
0
0
1
0
0
0
0
3
0
0
5
0
0
0
0
0
0
0
0
0
0
0
0
0
0
0
2
0
0
0
37
5
0
0
4
0
0
0
0
0
0
0
0
0
2
0
0
0
0
0
0
0
0
0
0
0
0
0
0
0
0
3
0
0
0
0
0
0
0
0
0
0
0
0
0
0
0
0
0
0


0
0
1
0
0
0
1
0
1
0
0
0
1
1
0
1
1
4
0
1
0
0
1
0
0
0
0
2
0
1
0
0
3
0
1
0
0
0
0
1
3
0
0
1
0
0
2
0
0
0
1
2
1
0
0
0
0
1
1
1
2
2
0
0
2
0
1
1
4
0
1
0
0
0
0
0
0
0
0
0
0


0
0
1
0
0
0
1
0
1
0
0
0
1
1
0
1
1
4
0
1
0
0
1
0
0
0
0
2
0
1
0
0
3
0
1
0
0
0
0
1
3
0
0
1
0
0
2
0
0
0
1
2
1
0
0
0
0
1
1
1
2
2
0
0
2
0
1
1
4
0
1
0
0
0
0
0
0
0
0
0
0


0
0
1
0
0
0
1
0
1
0
0
0
1
1
0
1
1
4
0
1
0
0
1
0
0
0
0
2
0
1
0
0
3
0
1
0
0
0
0
1
3
0
0
1
0
0
2
0
0
0
1
2
1
0
0
0
0
1
1
1
2
2
0
0
2
0
1
1
4
0
1
0
0
0
0
0
0
0
0
0
0


0
0
1
0
0
0
1
0
1
0
0
0
1
1
0
1
1
4
0
1
0
0
1
0
0
0
0
2
0
1
0
0
3
0
1
0
0
0
0
1
3
0
0
1
0
0
2
0
0
0
1
2
1
0
0
0
0
1
1
1
2
2
0
0
2
0
1
1
4
0
1
0
0
0
0
0
0
0
0
0
0


0
0
0
3
1
2
2
0
0
0
0
1
0
0
0
1
2
0
1
1
0
0
0
0
0
0
0
0
0
0
6
20
0
15
2
11
0
2
2
1
1
1
2
7
8
1
0
0
9
0
0
3
4
0
0
1
1
1
2
0
0
0
0
0
6
1
1
0
2
2
0
5
9
8
2
5
6
6
4
6
2


0
0
0
3
1
2
2
0
0
0
0
1
0
0
0
1
2
0
1
1
0
0
0
0
0
0
0
0
0
0
6
20
0
15
2
11
0
2
2
1
1
1
2
7
8
1
0
0
9
0
0
3
4
0
0
1
1
1
2
0
0
0
0
0
6
1
1
0
2
2
0
5
9
8
2
5
6
6
4
6
2


0
0
0
3
1
2
2
0
0
0
0
1
0
0
0
1
2
0
1
1
0
0
0
0
0
0
0
0
0
0
6
20
0
15
2
11
0
2
2
1
1
1
2
7
8
1
0
0
9
0
0
3
4
0
0
1
1
1
2
0
0
0
0
0
6
1
1
0
2
2
0
5
9
8
2
5
6
6
4
6
2


0
0
0
3
1
2
2
0
0
0
0
1
0
0
0
1
2
0
1
1
0
0
0
0
0
0
0
0
0
0
6
20
0
15
2
11
0
2
2
1
1
1
2
7
8
1
0
0
9
0
0
3
4
0
0
1
1
1
2
0
0
0
0
0
6
1
1
0
2
2
0
5
9
8
2
5
6
6
4
6
2


1
9
16
2
11
6
4
2
10
8
17
18
11
10
11
12
21
23
8
10
14
31
24
0
0
52
54
18
0
7
12
0
18
0
3
3
0
19
19
29
18
11
10
21
5
4
22
24
8
0
13
7
5
24
17
18
9
10
17
20
9
7
6
11
1
4
0
14
0
0
8
1
0
0
0
0
0
1
2
4
0


1
9
16
2
11
6
4
2
10
8
17
18
11
10
11
12
21
23
8
10
14
31
24
0
0
52
54
18
0
7
12
0
18
0
3
3
0
19
19
29
18
11
10
21
5
4
22
24
8
0
13
7
5
24
17
18
9
10
17
20
9
7
6
11
1
4
0
14
0
0
8
1
0
0
0
0
0
1
2
4
0


1
9
16
2
11
6
4
2
10
8
17
18
11
10
11
12
21
23
8
10
14
31
24
0
0
52
54
18
0
7
12
0
18
0
3
3
0
19
19
29
18
11
10
21
5
4
22
24
8
0
13
7
5
24
17
18
9
10
17
20
9
7
6
11
1
4
0
14
0
0
8
1
0
0
0
0
0
1
2
4
0


1
9
16
2
11
6
4
2
10
8
17
18
11
10
11
12
21
23
8
10
14
31
24
0
0
52
54
18
0
7
12
0
18
0
3
3
0
19
19
29
18
11
10
21
5
4
22
24
8
0
13
7
5
24
17
18
9
10
17
20
9
7
6
11
1
4
0
14
0
0
8
1
0
0
0
0
0
1
2
4
0


0
0
0
0
0
0
0
0
0
0
0
0
0
0
0
0
0
0
0
0
0
0
0
0
0
0
0
0
0
0
0
0
0
0
0
0
0
0
0
0
0
0
0
0
0
0
0
1
0
0
0
0
0
0
0
0
0
0
0
0
0
0
0
0
0
0
0
0
0
0
0
0
0
0
0
0
0
0
0
0
0


0
0
0
0
0
0
0
0
0
0
0
0
0
0
0
0
0
0
0
0
0
0
0
0
0
0
0
0
0
0
0
0
0
0
0
0
0
0
0
0
0
0
0
0
0
0
0
1
0
0
0
0
0
0
0
0
0
0
0
0
0
0
0
0
0
0
0
0
0
0
0
0
0
0
0
0
0
0
0
0
0


0
0
0
0
0
0
0
0
0
0
0
0
0
0
0
0
0
0
0
0
0
0
0
0
0
0
0
0
0
0
0
0
0
0
0
0
0
0
0
0
0
0
0
0
0
0
0
1
0
0
0
0
0
0
0
0
0
0
0
0
0
0
0
0
0
0
0
0
0
0
0
0
0
0
0
0
0
0
0
0
0


0
0
0
0
0
0
0
0
0
0
0
0
0
0
0
0
0
0
0
0
0
0
0
0
0
0
0
0
0
0
0
0
0
0
0
0
0
0
0
0
0
0
0
0
0
0
0
1
0
0
0
0
0
0
0
0
0
0
0
0
0
0
0
0
0
0
0
0
0
0
0
0
0
0
0
0
0
0
0
0
0


0
1
0
0
0
0
4
1
0
0
5
1
9
0
4
2
0
1
5
0
5
4
4
0
0
3
0
1
0
0
0
0
0
0
0
1
0
0
1
0
0
0
0
0
0
0
1
0
1
0
1
0
1
0
0
0
0
0
1
0
0
0
0
0
0
0
0
1
1
0
0
0
0
0
0
0
0
0
0
0
0


0
1
0
0
0
0
4
1
0
0
5
1
9
0
4
2
0
1
5
0
5
4
4
0
0
3
0
1
0
0
0
0
0
0
0
1
0
0
1
0
0
0
0
0
0
0
1
0
1
0
1
0
1
0
0
0
0
0
1
0
0
0
0
0
0
0
0
1
1
0
0
0
0
0
0
0
0
0
0
0
0


0
1
0
0
0
0
4
1
0
0
5
1
9
0
4
2
0
1
5
0
5
4
4
0
0
3
0
1
0
0
0
0
0
0
0
1
0
0
1
0
0
0
0
0
0
0
1
0
1
0
1
0
1
0
0
0
0
0
1
0
0
0
0
0
0
0
0
1
1
0
0
0
0
0
0
0
0
0
0
0
0


0
1
0
0
0
0
4
1
0
0
5
1
9
0
4
2
0
1
5
0
5
4
4
0
0
3
0
1
0
0
0
0
0
0
0
1
0
0
1
0
0
0
0
0
0
0
1
0
1
0
1
0
1
0
0
0
0
0
1
0
0
0
0
0
0
0
0
1
1
0
0
0
0
0
0
0
0
0
0
0
0


27
27
20
44
16
34
36
35
31
20
17
16
10
21
13
24
50
20
27
25
21
37
23
0
0
28
0
20
0
26
27
21
0
11
20
39
0
22
25
48
14
26
37
29
27
17
31
59
24
3
14
26
46
19
32
24
24
39
38
25
34
28
17
23
33
33
24
13
24
11
23
43
75
38
31
24
60
49
29
14
16


27
27
20
44
16
34
36
35
31
20
17
16
10
21
13
24
50
20
27
25
21
37
23
0
0
28
0
20
0
26
27
21
0
11
20
39
0
22
25
48
14
26
37
29
27
17
31
59
24
3
14
26
46
19
32
24
24
39
38
25
34
28
17
23
33
33
24
13
24
11
23
43
75
38
31
24
60
49
29
14
16


27
27
20
44
16
34
36
35
31
20
17
16
10
21
13
24
50
20
27
25
21
37
23
0
0
28
0
20
0
26
27
21
0
11
20
39
0
22
25
48
14
26
37
29
27
17
31
59
24
3
14
26
46
19
32
24
24
39
38
25
34
28
17
23
33
33
24
13
24
11
23
43
75
38
31
24
60
49
29
14
16


27
27
20
44
16
34
36
35
31
20
17
16
10
21
13
24
50
20
27
25
21
37
23
0
0
28
0
20
0
26
27
21
0
11
20
39
0
22
25
48
14
26
37
29
27
17
31
59
24
3
14
26
46
19
32
24
24
39
38
25
34
28
17
23
33
33
24
13
24
11
23
43
75
38
31
24
60
49
29
14
16


27
27
20
44
16
34
36
35
31
20
17
16
10
21
13
24
50
20
27
25
21
37
23
0
0
28
0
20
0
26
27
21
0
11
20
39
0
22
25
48
14
26
37
29
27
17
31
59
24
3
14
26
46
19
32
24
24
39
38
25
34
28
17
23
33
33
24
13
24
11
23
43
75
38
31
24
60
49
29
14
16


72
63
45
96
34
58
14
65
105
76
116
38
45
46
149
124
85
47
124
66
116
113
58
17
58
200
209
73
206
97
71
159
414
104
337
1150
799
30
62
129
37
50
46
288
66
60
107
27
26
82
64
208
43
94
72
69
39
71
58
43
100
94
176
57
74
86
32
111
48
46
61
195
249
62
74
94
75
141
240
41
46


0
0
0
0
0
0
0
0
0
0
0
0
0
0
0
0
0
0
0
0
0
1
0
0
0
3
0
8
0
0
1
0
0
0
4
1
0
0
1
6
2
4
1
24
11
6
13
0
0
0
0
0
0
0
0
0
0
0
0
1
0
0
0
0
0
0
0
0
0
0
0
0
0
0
0
0
0
0
0
0
0


0
0
0
0
0
0
0
0
0
0
0
0
0
0
0
0
0
0
0
0
0
1
0
0
0
3
0
8
0
0
1
0
0
0
4
1
0
0
1
6
2
4
1
24
11
6
13
0
0
0
0
0
0
0
0
0
0
0
0
1
0
0
0
0
0
0
0
0
0
0
0
0
0
0
0
0
0
0
0
0
0


0
0
0
0
0
0
0
0
0
0
0
0
0
0
0
0
0
0
0
0
0
1
0
0
0
3
0
8
0
0
1
0
0
0
4
1
0
0
1
6
2
4
1
24
11
6
13
0
0
0
0
0
0
0
0
0
0
0
0
1
0
0
0
0
0
0
0
0
0
0
0
0
0
0
0
0
0
0
0
0
0


0
0
0
0
0
0
0
0
0
0
0
0
0
0
0
0
0
0
0
0
0
1
0
0
0
3
0
8
0
0
1
0
0
0
4
1
0
0
1
6
2
4
1
24
11
6
13
0
0
0
0
0
0
0
0
0
0
0
0
1
0
0
0
0
0
0
0
0
0
0
0
0
0
0
0
0
0
0
0
0
0


0
0
0
0
0
0
0
0
0
0
0
0
0
0
0
0
0
0
0
0
0
1
0
0
0
3
0
8
0
0
1
0
0
0
4
1
0
0
1
6
2
4
1
24
11
6
13
0
0
0
0
0
0
0
0
0
0
0
0
1
0
0
0
0
0
0
0
0
0
0
0
0
0
0
0
0
0
0
0
0
0


37
7
17
53
11
19
11
17
26
39
35
22
14
33
35
31
10
15
19
24
24
19
16
0
0
7
0
10
0
6
2
0
0
0
9
13
0
13
23
10
19
12
3
10
14
4
21
5
9
0
25
5
8
5
16
33
12
21
19
13
71
37
57
16
40
61
15
80
30
16
40
53
90
35
51
74
37
49
51
19
25


31
4
0
46
9
16
4
11
14
16
6
13
3
6
10
10
1
4
12
9
2
4
5
0
0
0
0
9
0
0
1
0
0
0
9
7
0
0
16
6
5
7
3
3
8
1
16
5
3
0
11
4
6
2
9
18
3
11
7
0
58
17
52
6
38
45
14
71
20
9
23
28
49
22
38
59
24
32
31
4
24


31
4
0
46
9
16
4
11
14
16
6
13
3
6
10
10
1
4
12
9
2
4
5
0
0
0
0
9
0
0
1
0
0
0
9
7
0
0
16
6
5
7
3
3
8
1
16
5
3
0
11
4
6
2
9
18
3
11
7
0
58
17
52
6
38
45
14
71
20
9
23
28
49
22
38
59
24
32
31
4
24


31
4
0
46
9
16
4
11
14
16
6
13
3
6
10
10
1
4
12
9
2
4
5
0
0
0
0
9
0
0
1
0
0
0
9
7
0
0
16
6
5
7
3
3
8
1
16
5
3
0
11
4
6
2
9
18
3
11
7
0
58
17
52
6
38
45
14
71
20
9
23
28
49
22
38
59
24
32
31
4
24


31
4
0
46
9
16
4
11
14
16
6
13
3
6
10
10
1
4
12
9
2
4
5
0
0
0
0
9
0
0
1
0
0
0
9
7
0
0
16
6
5
7
3
3
8
1
16
5
3
0
11
4
6
2
9
18
3
11
7
0
58
17
52
6
38
45
14
71
20
9
23
28
49
22
38
59
24
32
31
4
24


6
3
17
4
2
3
7
6
10
22
29
9
11
26
25
18
8
10
7
13
22
15
10
0
0
7
0
1
0
6
0
0
0
0
0
2
0
13
5
3
13
4
0
6
4
3
5
0
6
0
11
1
2
3
6
13
8
9
8
12
12
18
5
6
2
14
1
8
10
7
15
24
39
12
11
14
13
15
20
15
1


6
3
17
4
2
3
7
6
10
22
29
9
11
26
25
18
8
10
7
13
22
15
10
0
0
7
0
1
0
6
0
0
0
0
0
2
0
13
5
3
13
4
0
6
4
3
5
0
6
0
11
1
2
3
6
13
8
9
8
12
12
18
5
6
2
14
1
8
10
7
15
24
39
12
11
14
13
15
20
15
1


6
3
17
4
2
3
7
6
10
22
29
9
11
26
25
18
8
10
7
13
22
15
10
0
0
7
0
1
0
6
0
0
0
0
0
2
0
13
5
3
13
4
0
6
4
3
5
0
6
0
11
1
2
3
6
13
8
9
8
12
12
18
5
6
2
14
1
8
10
7
15
24
39
12
11
14
13
15
20
15
1


6
3
17
4
2
3
7
6
10
22
29
9
11
26
25
18
8
10
7
13
22
15
10
0
0
7
0
1
0
6
0
0
0
0
0
2
0
13
5
3
13
4
0
6
4
3
5
0
6
0
11
1
2
3
6
13
8
9
8
12
12
18
5
6
2
14
1
8
10
7
15
24
39
12
11
14
13
15
20
15
1


0
0
0
0
0
0
0
0
0
0
0
0
0
0
0
0
0
0
0
0
0
0
0
0
0
0
0
0
0
0
0
0
0
0
0
0
0
0
0
0
0
1
0
0
0
0
0
0
0
0
0
0
0
0
0
1
0
0
0
0
0
0
0
0
0
0
0
0
0
0
0
0
0
0
0
0
0
0
0
0
0


0
0
0
0
0
0
0
0
0
0
0
0
0
0
0
0
0
0
0
0
0
0
0
0
0
0
0
0
0
0
0
0
0
0
0
0
0
0
0
0
0
1
0
0
0
0
0
0
0
0
0
0
0
0
0
1
0
0
0
0
0
0
0
0
0
0
0
0
0
0
0
0
0
0
0
0
0
0
0
0
0


0
0
0
0
0
0
0
0
0
0
0
0
0
0
0
0
0
0
0
0
0
0
0
0
0
0
0
0
0
0
0
0
0
0
0
0
0
0
0
0
0
1
0
0
0
0
0
0
0
0
0
0
0
0
0
1
0
0
0
0
0
0
0
0
0
0
0
0
0
0
0
0
0
0
0
0
0
0
0
0
0


0
0
0
0
0
0
0
0
0
0
0
0
0
0
0
0
0
0
0
0
0
0
0
0
0
0
0
0
0
0
0
0
0
0
0
0
0
0
0
0
0
1
0
0
0
0
0
0
0
0
0
0
0
0
0
1
0
0
0
0
0
0
0
0
0
0
0
0
0
0
0
0
0
0
0
0
0
0
0
0
0


0
0
0
0
0
0
0
0
0
0
0
0
0
0
0
1
0
0
0
0
0
0
0
0
0
0
0
0
0
0
1
0
0
0
0
0
0
0
0
0
0
0
0
0
0
0
0
0
0
0
0
0
0
0
0
0
0
1
3
1
1
0
0
0
0
1
0
0
0
0
2
1
1
0
0
1
0
1
0
0
0


0
0
0
0
0
0
0
0
0
0
0
0
0
0
0
1
0
0
0
0
0
0
0
0
0
0
0
0
0
0
1
0
0
0
0
0
0
0
0
0
0
0
0
0
0
0
0
0
0
0
0
0
0
0
0
0
0
1
3
1
1
0
0
0
0
1
0
0
0
0
2
1
1
0
0
1
0
1
0
0
0


0
0
0
0
0
0
0
0
0
0
0
0
0
0
0
1
0
0
0
0
0
0
0
0
0
0
0
0
0
0
1
0
0
0
0
0
0
0
0
0
0
0
0
0
0
0
0
0
0
0
0
0
0
0
0
0
0
1
3
1
1
0
0
0
0
1
0
0
0
0
2
1
1
0
0
1
0
1
0
0
0


0
0
0
0
0
0
0
0
0
0
0
0
0
0
0
1
0
0
0
0
0
0
0
0
0
0
0
0
0
0
1
0
0
0
0
0
0
0
0
0
0
0
0
0
0
0
0
0
0
0
0
0
0
0
0
0
0
1
3
1
1
0
0
0
0
1
0
0
0
0
2
1
1
0
0
1
0
1
0
0
0


0
0
0
3
0
0
0
0
2
1
0
0
0
1
0
2
1
1
0
2
0
0
1
0
0
0
0
0
0
0
0
0
0
0
0
4
0
0
2
1
1
0
0
1
2
0
0
0
0
0
3
0
0
0
1
1
1
0
1
0
0
2
0
4
0
1
0
1
0
0
0
0
1
1
2
0
0
1
0
0
0


0
0
0
3
0
0
0
0
2
1
0
0
0
1
0
2
1
1
0
2
0
0
1
0
0
0
0
0
0
0
0
0
0
0
0
4
0
0
2
1
1
0
0
1
2
0
0
0
0
0
3
0
0
0
1
1
1
0
1
0
0
2
0
4
0
1
0
1
0
0
0
0
1
1
2
0
0
1
0
0
0


0
0
0
3
0
0
0
0
2
1
0
0
0
1
0
2
1
1
0
2
0
0
1
0
0
0
0
0
0
0
0
0
0
0
0
4
0
0
2
1
1
0
0
1
2
0
0
0
0
0
3
0
0
0
1
1
1
0
1
0
0
2
0
4
0
1
0
1
0
0
0
0
1
1
2
0
0
1
0
0
0


0
0
0
3
0
0
0
0
2
1
0
0
0
1
0
2
1
1
0
2
0
0
1
0
0
0
0
0
0
0
0
0
0
0
0
4
0
0
2
1
1
0
0
1
2
0
0
0
0
0
3
0
0
0
1
1
1
0
1
0
0
2
0
4
0
1
0
1
0
0
0
0
1
1
2
0
0
1
0
0
0


34
53
26
42
14
35
3
46
69
34
78
6
27
5
109
85
72
16
101
32
44
60
21
17
57
146
209
19
206
79
23
126
263
45
308
986
799
2
18
31
4
22
31
78
15
32
11
7
9
0
32
192
27
84
45
25
18
36
32
18
8
37
14
25
14
14
11
19
8
6
8
119
112
19
15
14
24
55
86
21
8


0
0
0
0
0
0
0
0
0
0
0
0
0
0
0
0
0
0
0
0
0
0
0
0
0
0
0
0
0
0
0
0
0
0
0
0
0
0
1
1
0
0
0
4
1
0
0
0
0
0
0
1
0
0
0
0
0
0
0
0
0
0
0
0
0
0
0
0
0
0
0
0
0
0
0
0
0
2
0
0
0


0
0
0
0
0
0
0
0
0
0
0
0
0
0
0
0
0
0
0
0
0
0
0
0
0
0
0
0
0
0
0
0
0
0
0
0
0
0
1
1
0
0
0
4
1
0
0
0
0
0
0
1
0
0
0
0
0
0
0
0
0
0
0
0
0
0
0
0
0
0
0
0
0
0
0
0
0
2
0
0
0


0
0
0
0
0
0
0
0
0
0
0
0
0
0
0
0
0
0
0
0
0
0
0
0
0
0
0
0
0
0
0
0
0
0
0
0
0
0
1
1
0
0
0
4
1
0
0
0
0
0
0
1
0
0
0
0
0
0
0
0
0
0
0
0
0
0
0
0
0
0
0
0
0
0
0
0
0
2
0
0
0


0
0
0
0
0
0
0
0
0
0
0
0
0
0
0
0
0
0
0
0
0
0
0
0
0
0
0
0
0
0
0
0
0
0
0
0
0
0
1
1
0
0
0
4
1
0
0
0
0
0
0
1
0
0
0
0
0
0
0
0
0
0
0
0
0
0
0
0
0
0
0
0
0
0
0
0
0
2
0
0
0


0
0
1
0
2
2
0
0
1
2
1
1
2
1
1
1
0
4
0
0
7
14
8
0
0
28
0
4
0
3
5
0
7
0
0
1
0
0
0
2
0
0
1
2
3
6
4
5
2
0
1
31
8
1
4
2
0
2
0
5
1
0
0
0
0
0
0
0
0
0
1
12
18
1
0
8
6
4
11
3
2


0
0
1
0
2
2
0
0
1
2
1
1
2
0
1
1
0
4
0
0
2
8
3
0
0
5
0
0
0
0
2
0
0
0
0
0
0
0
0
0
0
0
1
2
3
2
2
5
1
0
0
24
3
1
0
2
0
1
0
3
1
0
0
0
0
0
0
0
0
0
1
1
12
0
0
3
2
3
8
2
1


0
0
1
0
2
2
0
0
1
2
1
1
2
0
1
1
0
4
0
0
2
8
3
0
0
5
0
0
0
0
2
0
0
0
0
0
0
0
0
0
0
0
1
2
3
2
2
5
1
0
0
24
3
1
0
2
0
1
0
3
1
0
0
0
0
0
0
0
0
0
1
1
12
0
0
3
2
3
8
2
1


0
0
1
0
2
2
0
0
1
2
1
1
2
0
1
1
0
4
0
0
2
8
3
0
0
5
0
0
0
0
2
0
0
0
0
0
0
0
0
0
0
0
1
2
3
2
2
5
1
0
0
24
3
1
0
2
0
1
0
3
1
0
0
0
0
0
0
0
0
0
1
1
12
0
0
3
2
3
8
2
1


0
0
0
0
0
0
0
0
0
0
0
0
0
1
0
0
0
0
0
0
3
6
5
0
0
23
0
4
0
3
3
0
7
0
0
1
0
0
0
2
0
0
0
0
0
3
2
0
1
0
1
6
5
0
4
0
0
1
0
2
0
0
0
0
0
0
0
0
0
0
0
6
1
1
0
3
4
1
0
0
1


0
0
0
0
0
0
0
0
0
0
0
0
0
1
0
0
0
0
0
0
3
6
5
0
0
23
0
4
0
3
3
0
7
0
0
1
0
0
0
2
0
0
0
0
0
3
2
0
1
0
1
6
5
0
4
0
0
1
0
2
0
0
0
0
0
0
0
0
0
0
0
6
1
1
0
3
4
1
0
0
1


0
0
0
0
0
0
0
0
0
0
0
0
0
1
0
0
0
0
0
0
3
6
5
0
0
23
0
4
0
3
3
0
7
0
0
1
0
0
0
2
0
0
0
0
0
3
2
0
1
0
1
6
5
0
4
0
0
1
0
2
0
0
0
0
0
0
0
0
0
0
0
6
1
1
0
3
4
1
0
0
1


0
0
0
0
0
0
0
0
0
0
0
0
0
0
0
0
0
0
0
0
0
0
0
0
0
0
0
0
0
0
0
0
0
0
0
0
0
0
0
0
0
0
0
0
0
0
0
0
0
0
0
1
0
0
0
0
0
0
0
0
0
0
0
0
0
0
0
0
0
0
0
5
5
0
0
2
0
0
3
1
0


0
0
0
0
0
0
0
0
0
0
0
0
0
0
0
0
0
0
0
0
0
0
0
0
0
0
0
0
0
0
0
0
0
0
0
0
0
0
0
0
0
0
0
0
0
0
0
0
0
0
0
1
0
0
0
0
0
0
0
0
0
0
0
0
0
0
0
0
0
0
0
5
5
0
0
2
0
0
3
1
0


0
0
0
0
0
0
0
0
0
0
0
0
0
0
0
0
0
0
0
0
0
0
0
0
0
0
0
0
0
0
0
0
0
0
0
0
0
0
0
0
0
0
0
0
0
0
0
0
0
0
0
1
0
0
0
0
0
0
0
0
0
0
0
0
0
0
0
0
0
0
0
5
5
0
0
2
0
0
3
1
0


0
0
0
0
0
0
0
0
0
0
0
0
0
0
0
0
0
0
0
0
2
0
0
0
0
0
0
0
0
0
0
0
0
0
0
0
0
0
0
0
0
0
0
0
0
1
0
0
0
0
0
0
0
0
0
0
0
0
0
0
0
0
0
0
0
0
0
0
0
0
0
0
0
0
0
0
0
0
0
0
0


0
0
0
0
0
0
0
0
0
0
0
0
0
0
0
0
0
0
0
0
2
0
0
0
0
0
0
0
0
0
0
0
0
0
0
0
0
0
0
0
0
0
0
0
0
1
0
0
0
0
0
0
0
0
0
0
0
0
0
0
0
0
0
0
0
0
0
0
0
0
0
0
0
0
0
0
0
0
0
0
0


0
0
0
0
0
0
0
0
0
0
0
0
0
0
0
0
0
0
0
0
2
0
0
0
0
0
0
0
0
0
0
0
0
0
0
0
0
0
0
0
0
0
0
0
0
1
0
0
0
0
0
0
0
0
0
0
0
0
0
0
0
0
0
0
0
0
0
0
0
0
0
0
0
0
0
0
0
0
0
0
0


0
0
0
1
0
0
0
0
0
0
0
0
0
0
0
0
0
0
0
0
0
0
0
0
0
0
0
0
0
0
0
0
0
0
0
3
0
0
0
0
0
1
0
0
0
0
0
0
0
0
0
0
0
0
1
0
0
0
0
0
0
0
0
0
0
0
0
0
0
0
1
0
1
0
0
0
0
0
0
0
0


0
0
0
1
0
0
0
0
0
0
0
0
0
0
0
0
0
0
0
0
0
0
0
0
0
0
0
0
0
0
0
0
0
0
0
3
0
0
0
0
0
1
0
0
0
0
0
0
0
0
0
0
0
0
1
0
0
0
0
0
0
0
0
0
0
0
0
0
0
0
1
0
1
0
0
0
0
0
0
0
0


0
0
0
1
0
0
0
0
0
0
0
0
0
0
0
0
0
0
0
0
0
0
0
0
0
0
0
0
0
0
0
0
0
0
0
3
0
0
0
0
0
1
0
0
0
0
0
0
0
0
0
0
0
0
1
0
0
0
0
0
0
0
0
0
0
0
0
0
0
0
1
0
1
0
0
0
0
0
0
0
0


0
0
0
1
0
0
0
0
0
0
0
0
0
0
0
0
0
0
0
0
0
0
0
0
0
0
0
0
0
0
0
0
0
0
0
3
0
0
0
0
0
1
0
0
0
0
0
0
0
0
0
0
0
0
1
0
0
0
0
0
0
0
0
0
0
0
0
0
0
0
1
0
1
0
0
0
0
0
0
0
0


9
2
1
2
0
1
0
0
0
1
1
3
2
1
0
0
0
0
3
1
33
34
9
0
0
59
0
4
0
3
14
0
19
0
2
21
1
2
11
21
2
0
1
29
9
26
6
2
5
0
1
63
5
6
2
3
5
3
3
7
3
3
2
0
1
0
1
2
1
2
0
17
14
9
0
2
5
17
40
3
5


9
2
1
2
0
1
0
0
0
1
1
3
2
1
0
0
0
0
3
1
33
34
9
0
0
59
0
4
0
3
14
0
19
0
2
21
1
2
11
21
2
0
1
29
9
26
6
2
5
0
1
63
5
6
2
3
5
3
3
7
3
3
2
0
1
0
1
2
1
2
0
17
14
9
0
2
5
17
40
3
5


9
2
1
2
0
1
0
0
0
1
1
3
2
1
0
0
0
0
3
1
33
34
9
0
0
59
0
4
0
3
14
0
19
0
2
21
1
2
11
21
2
0
1
29
9
26
6
2
5
0
1
63
5
6
2
3
5
3
3
7
3
3
2
0
1
0
1
2
1
2
0
17
14
9
0
2
5
17
40
3
5


9
2
1
2
0
1
0
0
0
1
1
3
2
1
0
0
0
0
3
1
33
34
9
0
0
59
0
4
0
3
14
0
19
0
2
21
1
2
11
21
2
0
1
29
9
26
6
2
5
0
1
63
5
6
2
3
5
3
3
7
3
3
2
0
1
0
1
2
1
2
0
17
14
9
0
2
5
17
40
3
5


25
51
24
39
11
30
2
46
68
30
76
2
23
3
108
84
61
12
96
31
2
10
4
17
57
56
209
11
206
73
4
126
206
38
306
958
798
0
6
3
2
21
29
43
2
0
0
0
2
0
30
92
13
77
38
19
12
31
29
6
3
34
12
25
13
13
10
17
5
4
5
90
74
8
13
4
12
25
29
15
1


25
51
24
39
11
30
2
46
68
30
76
2
23
3
108
84
61
12
96
31
2
10
4
17
57
56
209
11
206
73
4
126
206
38
306
958
798
0
6
3
2
21
29
43
2
0
0
0
2
0
30
92
13
77
38
19
12
31
29
6
3
34
12
25
13
13
10
17
5
4
5
90
74
8
13
4
12
25
29
15
1


25
51
24
39
11
30
2
46
68
30
76
2
23
3
108
84
61
12
96
31
2
10
4
17
57
56
209
11
206
73
4
126
206
38
306
958
798
0
6
3
2
21
29
43
2
0
0
0
2
0
30
92
13
77
38
19
12
31
29
6
3
34
12
25
13
13
10
17
5
4
5
90
74
8
13
4
12
25
29
15
1


25
51
24
39
11
30
2
46
68
30
76
2
23
3
108
84
61
12
96
31
2
10
4
17
57
56
209
11
206
73
4
126
206
38
306
958
798
0
6
3
2
21
29
43
2
0
0
0
2
0
30
92
13
77
38
19
12
31
29
6
3
34
12
25
13
13
10
17
5
4
5
90
74
8
13
4
12
25
29
15
1


0
0
0
0
1
2
1
0
0
1
0
0
0
0
0
0
11
0
2
0
2
2
0
0
0
3
0
0
0
0
0
0
31
7
0
3
0
0
0
4
0
0
0
0
0
0
1
0
0
0
0
5
1
0
0
1
1
0
0
0
1
0
0
0
0
1
0
0
2
0
1
0
5
1
2
0
1
7
6
0
0


0
0
0
0
1
2
1
0
0
1
0
0
0
0
0
0
11
0
2
0
2
2
0
0
0
3
0
0
0
0
0
0
31
7
0
3
0
0
0
4
0
0
0
0
0
0
1
0
0
0
0
5
1
0
0
1
1
0
0
0
1
0
0
0
0
1
0
0
2
0
1
0
5
1
2
0
1
7
6
0
0


0
0
0
0
1
2
1
0
0
1
0
0
0
0
0
0
11
0
2
0
2
2
0
0
0
3
0
0
0
0
0
0
31
7
0
3
0
0
0
4
0
0
0
0
0
0
1
0
0
0
0
5
1
0
0
1
1
0
0
0
1
0
0
0
0
1
0
0
2
0
1
0
5
1
2
0
1
7
6
0
0


0
0
0
0
1
2
1
0
0
1
0
0
0
0
0
0
11
0
2
0
2
2
0
0
0
3
0
0
0
0
0
0
31
7
0
3
0
0
0
4
0
0
0
0
0
0
1
0
0
0
0
5
1
0
0
1
1
0
0
0
1
0
0
0
0
1
0
0
2
0
1
0
5
1
2
0
1
7
6
0
0


1
2
0
1
8
3
0
2
9
3
3
10
3
8
3
7
3
15
4
9
17
6
4
0
0
8
0
8
0
7
23
33
144
47
3
5
0
11
11
19
2
8
4
6
3
3
9
0
5
0
7
3
8
4
10
9
8
11
7
9
18
18
29
14
19
11
6
11
8
24
12
1
24
7
5
6
9
12
7
1
3


1
2
0
1
8
3
0
2
9
3
3
10
3
8
3
7
3
15
4
9
17
6
4
0
0
8
0
8
0
7
23
33
144
47
3
5
0
11
11
19
2
8
4
6
3
3
9
0
5
0
7
3
8
4
10
9
8
11
7
9
18
18
29
14
19
11
6
11
8
24
12
1
24
7
5
6
9
12
7
1
3


1
2
0
1
8
3
0
2
9
3
3
10
3
8
3
7
3
15
4
9
17
6
4
0
0
8
0
8
0
7
23
33
144
47
3
5
0
11
11
19
2
8
4
6
3
3
9
0
5
0
7
3
8
4
10
9
8
11
7
9
18
18
29
14
19
11
6
11
8
24
12
1
24
7
5
6
9
12
7
1
3


1
2
0
1
8
3
0
2
9
3
3
10
3
8
3
7
3
15
4
9
17
6
4
0
0
8
0
8
0
7
23
33
144
47
3
5
0
11
11
19
2
8
4
6
3
3
9
0
5
0
7
3
8
4
10
9
8
11
7
9
18
18
29
14
19
11
6
11
8
24
12
1
24
7
5
6
9
12
7
1
3


1
2
0
1
8
3
0
2
9
3
3
10
3
8
3
7
3
15
4
9
17
6
4
0
0
8
0
8
0
7
23
33
144
47
3
5
0
11
11
19
2
8
4
6
3
3
9
0
5
0
7
3
8
4
10
9
8
11
7
9
18
18
29
14
19
11
6
11
8
24
12
1
24
7
5
6
9
12
7
1
3


0
0
0
0
0
0
0
0
0
0
0
0
0
0
0
0
0
0
0
0
5
8
16
0
0
31
0
9
0
1
1
0
0
12
0
2
0
4
2
2
3
1
0
3
0
4
16
0
1
0
0
1
0
0
0
0
0
0
0
0
0
0
0
0
0
0
0
0
0
0
0
0
0
0
0
0
0
0
2
0
0


0
0
0
0
0
0
0
0
0
0
0
0
0
0
0
0
0
0
0
0
5
8
16
0
0
31
0
9
0
0
1
0
0
12
0
2
0
4
2
2
3
1
0
3
0
4
16
0
1
0
0
1
0
0
0
0
0
0
0
0
0
0
0
0
0
0
0
0
0
0
0
0
0
0
0
0
0
0
2
0
0


0
0
0
0
0
0
0
0
0
0
0
0
0
0
0
0
0
0
0
0
5
8
16
0
0
30
0
9
0
0
1
0
0
12
0
2
0
4
2
2
3
1
0
3
0
4
16
0
1
0
0
1
0
0
0
0
0
0
0
0
0
0
0
0
0
0
0
0
0
0
0
0
0
0
0
0
0
0
2
0
0


0
0
0
0
0
0
0
0
0
0
0
0
0
0
0
0
0
0
0
0
2
5
9
0
0
19
0
0
0
0
0
0
0
0
0
2
0
0
0
0
0
0
0
3
0
0
7
0
0
0
0
1
0
0
0
0
0
0
0
0
0
0
0
0
0
0
0
0
0
0
0
0
0
0
0
0
0
0
0
0
0


0
0
0
0
0
0
0
0
0
0
0
0
0
0
0
0
0
0
0
0
2
5
9
0
0
19
0
0
0
0
0
0
0
0
0
2
0
0
0
0
0
0
0
3
0
0
7
0
0
0
0
1
0
0
0
0
0
0
0
0
0
0
0
0
0
0
0
0
0
0
0
0
0
0
0
0
0
0
0
0
0


0
0
0
0
0
0
0
0
0
0
0
0
0
0
0
0
0
0
0
0
0
0
0
0
0
0
0
0
0
0
0
0
0
0
0
0
0
0
0
0
1
0
0
0
0
0
0
0
0
0
0
0
0
0
0
0
0
0
0
0
0
0
0
0
0
0
0
0
0
0
0
0
0
0
0
0
0
0
2
0
0


0
0
0
0
0
0
0
0
0
0
0
0
0
0
0
0
0
0
0
0
0
0
0
0
0
0
0
0
0
0
0
0
0
0
0
0
0
0
0
0
1
0
0
0
0
0
0
0
0
0
0
0
0
0
0
0
0
0
0
0
0
0
0
0
0
0
0
0
0
0
0
0
0
0
0
0
0
0
2
0
0


0
0
0
0
0
0
0
0
0
0
0
0
0
0
0
0
0
0
0
0
3
3
7
0
0
11
0
9
0
0
1
0
0
12
0
0
0
4
2
2
2
1
0
0
0
4
9
0
1
0
0
0
0
0
0
0
0
0
0
0
0
0
0
0
0
0
0
0
0
0
0
0
0
0
0
0
0
0
0
0
0


0
0
0
0
0
0
0
0
0
0
0
0
0
0
0
0
0
0
0
0
3
3
7
0
0
11
0
9
0
0
1
0
0
12
0
0
0
4
2
2
2
1
0
0
0
4
9
0
1
0
0
0
0
0
0
0
0
0
0
0
0
0
0
0
0
0
0
0
0
0
0
0
0
0
0
0
0
0
0
0
0


0
0
0
0
0
0
0
0
0
0
0
0
0
0
0
0
0
0
0
0
0
0
0
0
0
1
0
0
0
0
0
0
0
0
0
0
0
0
0
0
0
0
0
0
0
0
0
0
0
0
0
0
0
0
0
0
0
0
0
0
0
0
0
0
0
0
0
0
0
0
0
0
0
0
0
0
0
0
0
0
0


0
0
0
0
0
0
0
0
0
0
0
0
0
0
0
0
0
0
0
0
0
0
0
0
0
1
0
0
0
0
0
0
0
0
0
0
0
0
0
0
0
0
0
0
0
0
0
0
0
0
0
0
0
0
0
0
0
0
0
0
0
0
0
0
0
0
0
0
0
0
0
0
0
0
0
0
0
0
0
0
0


0
0
0
0
0
0
0
0
0
0
0
0
0
0
0
0
0
0
0
0
0
0
0
0
0
1
0
0
0
0
0
0
0
0
0
0
0
0
0
0
0
0
0
0
0
0
0
0
0
0
0
0
0
0
0
0
0
0
0
0
0
0
0
0
0
0
0
0
0
0
0
0
0
0
0
0
0
0
0
0
0


0
0
0
0
0
0
0
0
0
0
0
0
0
0
0
0
0
0
0
0
0
0
0
0
0
0
0
0
0
1
0
0
0
0
0
0
0
0
0
0
0
0
0
0
0
0
0
0
0
0
0
0
0
0
0
0
0
0
0
0
0
0
0
0
0
0
0
0
0
0
0
0
0
0
0
0
0
0
0
0
0


0
0
0
0
0
0
0
0
0
0
0
0
0
0
0
0
0
0
0
0
0
0
0
0
0
0
0
0
0
1
0
0
0
0
0
0
0
0
0
0
0
0
0
0
0
0
0
0
0
0
0
0
0
0
0
0
0
0
0
0
0
0
0
0
0
0
0
0
0
0
0
0
0
0
0
0
0
0
0
0
0


0
0
0
0
0
0
0
0
0
0
0
0
0
0
0
0
0
0
0
0
0
0
0
0
0
0
0
0
0
1
0
0
0
0
0
0
0
0
0
0
0
0
0
0
0
0
0
0
0
0
0
0
0
0
0
0
0
0
0
0
0
0
0
0
0
0
0
0
0
0
0
0
0
0
0
0
0
0
0
0
0


0
0
0
0
0
0
0
0
0
0
0
0
0
0
0
0
0
0
0
0
0
0
0
0
0
0
0
0
0
1
0
0
0
0
0
0
0
0
0
0
0
0
0
0
0
0
0
0
0
0
0
0
0
0
0
0
0
0
0
0
0
0
0
0
0
0
0
0
0
0
0
0
0
0
0
0
0
0
0
0
0


0
0
0
0
0
0
0
0
0
0
0
0
0
0
0
0
0
0
0
0
19
7
0
0
1
0
0
0
0
0
12
0
0
0
12
133
0
0
0
49
2
0
1
113
12
8
8
0
0
82
0
2
0
0
0
0
0
0
0
0
0
1
76
0
0
0
0
0
0
0
0
15
20
1
1
0
2
6
53
0
8


0
0
0
0
0
0
0
0
0
0
0
0
0
0
0
0
0
0
0
0
0
0
0
0
0
0
0
0
0
0
0
0
0
0
0
0
0
0
0
0
1
0
0
0
9
2
0
0
0
0
0
0
0
0
0
0
0
0
0
0
0
0
0
0
0
0
0
0
0
0
0
0
0
0
0
0
0
0
0
0
0


0
0
0
0
0
0
0
0
0
0
0
0
0
0
0
0
0
0
0
0
0
0
0
0
0
0
0
0
0
0
0
0
0
0
0
0
0
0
0
0
0
0
0
0
0
2
0
0
0
0
0
0
0
0
0
0
0
0
0
0
0
0
0
0
0
0
0
0
0
0
0
0
0
0
0
0
0
0
0
0
0


0
0
0
0
0
0
0
0
0
0
0
0
0
0
0
0
0
0
0
0
0
0
0
0
0
0
0
0
0
0
0
0
0
0
0
0
0
0
0
0
0
0
0
0
0
2
0
0
0
0
0
0
0
0
0
0
0
0
0
0
0
0
0
0
0
0
0
0
0
0
0
0
0
0
0
0
0
0
0
0
0


0
0
0
0
0
0
0
0
0
0
0
0
0
0
0
0
0
0
0
0
0
0
0
0
0
0
0
0
0
0
0
0
0
0
0
0
0
0
0
0
0
0
0
0
0
2
0
0
0
0
0
0
0
0
0
0
0
0
0
0
0
0
0
0
0
0
0
0
0
0
0
0
0
0
0
0
0
0
0
0
0


0
0
0
0
0
0
0
0
0
0
0
0
0
0
0
0
0
0
0
0
0
0
0
0
0
0
0
0
0
0
0
0
0
0
0
0
0
0
0
0
1
0
0
0
1
0
0
0
0
0
0
0
0
0
0
0
0
0
0
0
0
0
0
0
0
0
0
0
0
0
0
0
0
0
0
0
0
0
0
0
0


0
0
0
0
0
0
0
0
0
0
0
0
0
0
0
0
0
0
0
0
0
0
0
0
0
0
0
0
0
0
0
0
0
0
0
0
0
0
0
0
1
0
0
0
1
0
0
0
0
0
0
0
0
0
0
0
0
0
0
0
0
0
0
0
0
0
0
0
0
0
0
0
0
0
0
0
0
0
0
0
0


0
0
0
0
0
0
0
0
0
0
0
0
0
0
0
0
0
0
0
0
0
0
0
0
0
0
0
0
0
0
0
0
0
0
0
0
0
0
0
0
1
0
0
0
1
0
0
0
0
0
0
0
0
0
0
0
0
0
0
0
0
0
0
0
0
0
0
0
0
0
0
0
0
0
0
0
0
0
0
0
0


0
0
0
0
0
0
0
0
0
0
0
0
0
0
0
0
0
0
0
0
0
0
0
0
0
0
0
0
0
0
0
0
0
0
0
0
0
0
0
0
0
0
0
0
8
0
0
0
0
0
0
0
0
0
0
0
0
0
0
0
0
0
0
0
0
0
0
0
0
0
0
0
0
0
0
0
0
0
0
0
0


0
0
0
0
0
0
0
0
0
0
0
0
0
0
0
0
0
0
0
0
0
0
0
0
0
0
0
0
0
0
0
0
0
0
0
0
0
0
0
0
0
0
0
0
8
0
0
0
0
0
0
0
0
0
0
0
0
0
0
0
0
0
0
0
0
0
0
0
0
0
0
0
0
0
0
0
0
0
0
0
0


0
0
0
0
0
0
0
0
0
0
0
0
0
0
0
0
0
0
0
0
0
0
0
0
0
0
0
0
0
0
0
0
0
0
0
0
0
0
0
0
0
0
0
0
8
0
0
0
0
0
0
0
0
0
0
0
0
0
0
0
0
0
0
0
0
0
0
0
0
0
0
0
0
0
0
0
0
0
0
0
0


0
0
0
0
0
0
0
0
0
0
0
0
0
0
0
0
0
0
0
0
17
7
0
0
1
0
0
0
0
0
11
0
0
0
12
131
0
0
0
48
1
0
0
110
3
6
5
0
0
81
0
1
0
0
0
0
0
0
0
0
0
1
76
0
0
0
0
0
0
0
0
15
19
1
1
0
2
6
53
0
8


0
0
0
0
0
0
0
0
0
0
0
0
0
0
0
0
0
0
0
0
17
7
0
0
1
0
0
0
0
0
11
0
0
0
12
131
0
0
0
48
1
0
0
110
3
6
5
0
0
81
0
1
0
0
0
0
0
0
0
0
0
1
76
0
0
0
0
0
0
0
0
15
19
1
1
0
2
6
53
0
8


0
0
0
0
0
0
0
0
0
0
0
0
0
0
0
0
0
0
0
0
2
6
0
0
0
0
0
0
0
0
5
0
0
0
2
7
0
0
0
21
1
0
0
52
3
1
3
0
0
0
0
0
0
0
0
0
0
0
0
0
0
0
0
0
0
0
0
0
0
0
0
3
2
0
0
0
0
1
32
0
0


0
0
0
0
0
0
0
0
0
0
0
0
0
0
0
0
0
0
0
0
2
5
0
0
0
0
0
0
0
0
5
0
0
0
2
7
0
0
0
21
1
0
0
50
3
1
3
0
0
0
0
0
0
0
0
0
0
0
0
0
0
0
0
0
0
0
0
0
0
0
0
3
2
0
0
0
0
0
31
0
0


0
0
0
0
0
0
0
0
0
0
0
0
0
0
0
0
0
0
0
0
0
0
0
0
0
0
0
0
0
0
0
0
0
0
0
0
0
0
0
0
0
0
0
2
0
0
0
0
0
0
0
0
0
0
0
0
0
0
0
0
0
0
0
0
0
0
0
0
0
0
0
0
0
0
0
0
0
0
1
0
0


0
0
0
0
0
0
0
0
0
0
0
0
0
0
0
0
0
0
0
0
0
1
0
0
0
0
0
0
0
0
0
0
0
0
0
0
0
0
0
0
0
0
0
0
0
0
0
0
0
0
0
0
0
0
0
0
0
0
0
0
0
0
0
0
0
0
0
0
0
0
0
0
0
0
0
0
0
1
0
0
0


0
0
0
0
0
0
0
0
0
0
0
0
0
0
0
0
0
0
0
0
0
0
0
0
0
0
0
0
0
0
1
0
0
0
0
32
0
0
0
0
0
0
0
0
0
0
0
0
0
0
0
0
0
0
0
0
0
0
0
0
0
0
0
0
0
0
0
0
0
0
0
0
0
0
0
0
0
0
0
0
0


0
0
0
0
0
0
0
0
0
0
0
0
0
0
0
0
0
0
0
0
0
0
0
0
0
0
0
0
0
0
1
0
0
0
0
32
0
0
0
0
0
0
0
0
0
0
0
0
0
0
0
0
0
0
0
0
0
0
0
0
0
0
0
0
0
0
0
0
0
0
0
0
0
0
0
0
0
0
0
0
0


0
0
0
0
0
0
0
0
0
0
0
0
0
0
0
0
0
0
0
0
15
1
0
0
1
0
0
0
0
0
5
0
0
0
10
92
0
0
0
27
0
0
0
58
0
5
2
0
0
81
0
1
0
0
0
0
0
0
0
0
0
1
76
0
0
0
0
0
0
0
0
12
17
1
1
0
2
5
21
0
8


0
0
0
0
0
0
0
0
0
0
0
0
0
0
0
0
0
0
0
0
15
1
0
0
1
0
0
0
0
0
5
0
0
0
10
92
0
0
0
27
0
0
0
58
0
5
2
0
0
81
0
1
0
0
0
0
0
0
0
0
0
1
76
0
0
0
0
0
0
0
0
12
17
1
1
0
2
5
21
0
8


0
0
0
0
0
0
0
0
0
0
0
0
0
0
0
0
0
0
0
0
2
0
0
0
0
0
0
0
0
0
1
0
0
0
0
2
0
0
0
1
0
0
1
3
0
0
3
0
0
1
0
1
0
0
0
0
0
0
0
0
0
0
0
0
0
0
0
0
0
0
0
0
1
0
0
0
0
0
0
0
0


0
0
0
0
0
0
0
0
0
0
0
0
0
0
0
0
0
0
0
0
2
0
0
0
0
0
0
0
0
0
1
0
0
0
0
2
0
0
0
1
0
0
1
3
0
0
3
0
0
1
0
1
0
0
0
0
0
0
0
0
0
0
0
0
0
0
0
0
0
0
0
0
1
0
0
0
0
0
0
0
0


0
0
0
0
0
0
0
0
0
0
0
0
0
0
0
0
0
0
0
0
2
0
0
0
0
0
0
0
0
0
1
0
0
0
0
2
0
0
0
1
0
0
1
3
0
0
3
0
0
1
0
1
0
0
0
0
0
0
0
0
0
0
0
0
0
0
0
0
0
0
0
0
1
0
0
0
0
0
0
0
0


0
0
0
0
0
0
0
0
0
0
0
0
0
0
0
0
0
0
0
0
2
0
0
0
0
0
0
0
0
0
1
0
0
0
0
2
0
0
0
1
0
0
1
3
0
0
3
0
0
1
0
1
0
0
0
0
0
0
0
0
0
0
0
0
0
0
0
0
0
0
0
0
1
0
0
0
0
0
0
0
0


0
0
1
0
0
1
0
0
1
0
0
0
0
0
0
1
0
0
0
1
3
1
1
0
0
5
0
3
0
0
5
0
7
0
1
9
0
0
3
3
1
1
0
14
1
1
2
1
0
0
0
2
0
0
0
0
0
1
0
0
1
0
0
0
0
0
0
0
1
0
1
2
0
0
1
0
1
12
7
0
0


0
0
0
0
0
0
0
0
1
0
0
0
0
0
0
1
0
0
0
1
3
1
0
0
0
4
0
2
0
0
5
0
7
0
0
9
0
0
3
2
1
1
0
14
1
1
2
1
0
0
0
2
0
0
0
0
0
1
0
0
1
0
0
0
0
0
0
0
0
0
1
2
0
0
1
0
1
12
7
0
0


0
0
0
0
0
0
0
0
0
0
0
0
0
0
0
1
0
0
0
0
2
0
0
0
0
0
0
0
0
0
1
0
0
0
0
0
0
0
0
1
0
0
0
2
0
0
0
0
0
0
0
1
0
0
0
0
0
0
0
0
0
0
0
0
0
0
0
0
0
0
0
0
0
0
0
0
0
0
0
0
0


0
0
0
0
0
0
0
0
0
0
0
0
0
0
0
1
0
0
0
0
2
0
0
0
0
0
0
0
0
0
1
0
0
0
0
0
0
0
0
1
0
0
0
2
0
0
0
0
0
0
0
1
0
0
0
0
0
0
0
0
0
0
0
0
0
0
0
0
0
0
0
0
0
0
0
0
0
0
0
0
0


0
0
0
0
0
0
0
0
0
0
0
0
0
0
0
1
0
0
0
0
2
0
0
0
0
0
0
0
0
0
1
0
0
0
0
0
0
0
0
1
0
0
0
2
0
0
0
0
0
0
0
1
0
0
0
0
0
0
0
0
0
0
0
0
0
0
0
0
0
0
0
0
0
0
0
0
0
0
0
0
0


0
0
0
0
0
0
0
0
1
0
0
0
0
0
0
0
0
0
0
1
1
1
0
0
0
4
0
2
0
0
4
0
7
0
0
9
0
0
3
1
1
1
0
12
1
1
2
1
0
0
0
1
0
0
0
0
0
1
0
0
1
0
0
0
0
0
0
0
0
0
1
2
0
0
1
0
1
12
7
0
0


0
0
0
0
0
0
0
0
1
0
0
0
0
0
0
0
0
0
0
1
0
0
0
0
0
0
0
0
0
0
0
0
0
0
0
0
0
0
0
1
0
0
0
0
0
0
0
0
0
0
0
1
0
0
0
0
0
1
0
0
1
0
0
0
0
0
0
0
0
0
0
0
0
0
1
0
0
0
0
0
0


0
0
0
0
0
0
0
0
1
0
0
0
0
0
0
0
0
0
0
1
0
0
0
0
0
0
0
0
0
0
0
0
0
0
0
0
0
0
0
1
0
0
0
0
0
0
0
0
0
0
0
1
0
0
0
0
0
1
0
0
1
0
0
0
0
0
0
0
0
0
0
0
0
0
1
0
0
0
0
0
0


0
0
0
0
0
0
0
0
0
0
0
0
0
0
0
0
0
0
0
0
1
1
0
0
0
4
0
2
0
0
4
0
7
0
0
7
0
0
3
0
1
0
0
12
1
1
2
1
0
0
0
0
0
0
0
0
0
0
0
0
0
0
0
0
0
0
0
0
0
0
1
0
0
0
0
0
0
7
6
0
0


0
0
0
0
0
0
0
0
0
0
0
0
0
0
0
0
0
0
0
0
0
1
0
0
0
3
0
2
0
0
1
0
7
0
0
7
0
0
3
0
1
0
0
4
1
0
2
1
0
0
0
0
0
0
0
0
0
0
0
0
0
0
0
0
0
0
0
0
0
0
1
0
0
0
0
0
0
7
5
0
0


0
0
0
0
0
0
0
0
0
0
0
0
0
0
0
0
0
0
0
0
1
0
0
0
0
1
0
0
0
0
1
0
0
0
0
0
0
0
0
0
0
0
0
8
0
1
0
0
0
0
0
0
0
0
0
0
0
0
0
0
0
0
0
0
0
0
0
0
0
0
0
0
0
0
0
0
0
0
1
0
0


0
0
0
0
0
0
0
0
0
0
0
0
0
0
0
0
0
0
0
0
0
0
0
0
0
0
0
0
0
0
2
0
0
0
0
0
0
0
0
0
0
0
0
0
0
0
0
0
0
0
0
0
0
0
0
0
0
0
0
0
0
0
0
0
0
0
0
0
0
0
0
0
0
0
0
0
0
0
0
0
0


0
0
0
0
0
0
0
0
0
0
0
0
0
0
0
0
0
0
0
0
0
0
0
0
0
0
0
0
0
0
0
0
0
0
0
2
0
0
0
0
0
1
0
0
0
0
0
0
0
0
0
0
0
0
0
0
0
0
0
0
0
0
0
0
0
0
0
0
0
0
0
2
0
0
0
0
1
5
1
0
0


0
0
0
0
0
0
0
0
0
0
0
0
0
0
0
0
0
0
0
0
0
0
0
0
0
0
0
0
0
0
0
0
0
0
0
2
0
0
0
0
0
1
0
0
0
0
0
0
0
0
0
0
0
0
0
0
0
0
0
0
0
0
0
0
0
0
0
0
0
0
0
2
0
0
0
0
1
5
1
0
0


0
0
1
0
0
1
0
0
0
0
0
0
0
0
0
0
0
0
0
0
0
0
1
0
0
1
0
1
0
0
0
0
0
0
0
0
0
0
0
0
0
0
0
0
0
0
0
0
0
0
0
0
0
0
0
0
0
0
0
0
0
0
0
0
0
0
0
0
1
0
0
0
0
0
0
0
0
0
0
0
0


0
0
1
0
0
1
0
0
0
0
0
0
0
0
0
0
0
0
0
0
0
0
1
0
0
1
0
1
0
0
0
0
0
0
0
0
0
0
0
0
0
0
0
0
0
0
0
0
0
0
0
0
0
0
0
0
0
0
0
0
0
0
0
0
0
0
0
0
1
0
0
0
0
0
0
0
0
0
0
0
0


0
0
1
0
0
1
0
0
0
0
0
0
0
0
0
0
0
0
0
0
0
0
1
0
0
1
0
1
0
0
0
0
0
0
0
0
0
0
0
0
0
0
0
0
0
0
0
0
0
0
0
0
0
0
0
0
0
0
0
0
0
0
0
0
0
0
0
0
1
0
0
0
0
0
0
0
0
0
0
0
0


0
0
1
0
0
1
0
0
0
0
0
0
0
0
0
0
0
0
0
0
0
0
1
0
0
1
0
1
0
0
0
0
0
0
0
0
0
0
0
0
0
0
0
0
0
0
0
0
0
0
0
0
0
0
0
0
0
0
0
0
0
0
0
0
0
0
0
0
1
0
0
0
0
0
0
0
0
0
0
0
0


0
0
0
0
0
0
0
0
0
0
0
0
0
0
0
0
0
0
0
0
0
0
0
0
0
0
0
0
0
0
0
0
0
0
1
0
0
0
0
1
0
0
0
0
0
0
0
0
0
0
0
0
0
0
0
0
0
0
0
0
0
0
0
0
0
0
0
0
0
0
0
0
0
0
0
0
0
0
0
0
0


0
0
0
0
0
0
0
0
0
0
0
0
0
0
0
0
0
0
0
0
0
0
0
0
0
0
0
0
0
0
0
0
0
0
1
0
0
0
0
1
0
0
0
0
0
0
0
0
0
0
0
0
0
0
0
0
0
0
0
0
0
0
0
0
0
0
0
0
0
0
0
0
0
0
0
0
0
0
0
0
0


0
0
0
0
0
0
0
0
0
0
0
0
0
0
0
0
0
0
0
0
0
0
0
0
0
0
0
0
0
0
0
0
0
0
1
0
0
0
0
1
0
0
0
0
0
0
0
0
0
0
0
0
0
0
0
0
0
0
0
0
0
0
0
0
0
0
0
0
0
0
0
0
0
0
0
0
0
0
0
0
0


0
0
0
0
0
0
0
0
0
0
0
0
0
0
0
0
0
0
0
0
0
0
0
0
0
0
0
0
0
0
0
0
0
0
1
0
0
0
0
1
0
0
0
0
0
0
0
0
0
0
0
0
0
0
0
0
0
0
0
0
0
0
0
0
0
0
0
0
0
0
0
0
0
0
0
0
0
0
0
0
0


0
1
1
0
1
0
0
0
0
0
0
0
1
0
2
0
0
1
0
0
4
11
0
0
0
0
0
16
0
4
4
0
0
0
0
1
0
0
4
9
4
2
6
40
10
2
27
14
2
0
0
3
0
1
1
2
1
2
0
2
2
1
0
2
1
0
0
1
1
0
0
5
3
0
1
0
2
7
34
0
2


0
1
1
0
1
0
0
0
0
0
0
0
1
0
2
0
0
1
0
0
4
11
0
0
0
0
0
16
0
4
4
0
0
0
0
1
0
0
4
9
4
2
6
40
10
2
27
14
2
0
0
3
0
1
1
2
1
2
0
2
2
1
0
2
1
0
0
1
1
0
0
5
3
0
1
0
2
7
34
0
2


0
1
1
0
1
0
0
0
0
0
0
0
1
0
2
0
0
1
0
0
4
11
0
0
0
0
0
16
0
4
4
0
0
0
0
1
0
0
4
9
4
2
6
40
10
2
27
14
2
0
0
3
0
1
1
2
1
2
0
2
2
1
0
2
1
0
0
1
1
0
0
5
3
0
1
0
2
7
34
0
2


0
1
1
0
1
0
0
0
0
0
0
0
1
0
2
0
0
1
0
0
4
11
0
0
0
0
0
16
0
4
4
0
0
0
0
1
0
0
4
9
4
2
6
40
10
2
27
14
2
0
0
3
0
1
1
2
1
2
0
2
2
1
0
2
1
0
0
1
1
0
0
5
3
0
1
0
2
7
34
0
2


0
1
1
0
1
0
0
0
0
0
0
0
1
0
2
0
0
1
0
0
4
11
0
0
0
0
0
16
0
4
4
0
0
0
0
1
0
0
4
9
4
2
6
40
10
2
27
14
2
0
0
3
0
1
1
2
1
2
0
2
2
1
0
2
1
0
0
1
1
0
0
5
3
0
1
0
2
7
34
0
2


0
0
0
0
0
0
0
0
0
0
0
0
0
0
0
2
0
0
0
0
0
0
0
0
0
0
0
0
0
0
0
0
0
0
0
0
0
0
0
0
0
0
0
0
0
0
0
0
0
0
0
0
0
0
0
0
0
1
1
0
0
0
0
0
0
0
0
0
0
0
0
0
0
0
0
0
0
0
0
0
0


0
0
0
0
0
0
0
0
0
0
0
0
0
0
0
2
0
0
0
0
0
0
0
0
0
0
0
0
0
0
0
0
0
0
0
0
0
0
0
0
0
0
0
0
0
0
0
0
0
0
0
0
0
0
0
0
0
1
1
0
0
0
0
0
0
0
0
0
0
0
0
0
0
0
0
0
0
0
0
0
0


0
0
0
0
0
0
0
0
0
0
0
0
0
0
0
2
0
0
0
0
0
0
0
0
0
0
0
0
0
0
0
0
0
0
0
0
0
0
0
0
0
0
0
0
0
0
0
0
0
0
0
0
0
0
0
0
0
1
1
0
0
0
0
0
0
0
0
0
0
0
0
0
0
0
0
0
0
0
0
0
0


0
0
0
0
0
0
0
0
0
0
0
0
0
0
0
2
0
0
0
0
0
0
0
0
0
0
0
0
0
0
0
0
0
0
0
0
0
0
0
0
0
0
0
0
0
0
0
0
0
0
0
0
0
0
0
0
0
1
1
0
0
0
0
0
0
0
0
0
0
0
0
0
0
0
0
0
0
0
0
0
0


0
0
0
0
0
0
0
0
0
0
0
0
0
0
0
2
0
0
0
0
0
0
0
0
0
0
0
0
0
0
0
0
0
0
0
0
0
0
0
0
0
0
0
0
0
0
0
0
0
0
0
0
0
0
0
0
0
1
1
0
0
0
0
0
0
0
0
0
0
0
0
0
0
0
0
0
0
0
0
0
0


0
0
0
0
0
0
0
0
0
0
0
0
0
0
0
2
0
0
0
0
0
0
0
0
0
0
0
0
0
0
0
0
0
0
0
0
0
0
0
0
0
0
0
0
0
0
0
0
0
0
0
0
0
0
0
0
0
1
1
0
0
0
0
0
0
0
0
0
0
0
0
0
0
0
0
0
0
0
0
0
0


248
151
102
283
114
235
176
313
101
171
109
141
106
179
242
121
211
135
170
223
127
93
214
0
0
63
0
90
0
101
205
0
3
0
31
140
0
155
221
64
52
86
70
59
75
107
93
66
152
0
230
88
200
130
128
96
83
215
152
85
270
329
182
103
342
208
178
81
287
312
142
170
337
245
44
79
65
76
221
27
129


0
0
0
0
0
0
0
2
0
0
0
0
0
0
0
0
0
0
0
0
0
0
0
0
0
0
0
0
0
0
1
0
0
0
0
0
0
0
0
0
0
0
0
0
1
2
0
0
0
0
0
0
1
0
0
0
0
1
0
0
0
0
0
0
0
3
0
0
0
0
0
0
0
0
0
0
0
0
0
0
1


0
0
0
0
0
0
0
2
0
0
0
0
0
0
0
0
0
0
0
0
0
0
0
0
0
0
0
0
0
0
1
0
0
0
0
0
0
0
0
0
0
0
0
0
1
2
0
0
0
0
0
0
1
0
0
0
0
1
0
0
0
0
0
0
0
3
0
0
0
0
0
0
0
0
0
0
0
0
0
0
1


0
0
0
0
0
0
0
2
0
0
0
0
0
0
0
0
0
0
0
0
0
0
0
0
0
0
0
0
0
0
1
0
0
0
0
0
0
0
0
0
0
0
0
0
1
2
0
0
0
0
0
0
1
0
0
0
0
1
0
0
0
0
0
0
0
3
0
0
0
0
0
0
0
0
0
0
0
0
0
0
1


0
0
0
0
0
0
0
2
0
0
0
0
0
0
0
0
0
0
0
0
0
0
0
0
0
0
0
0
0
0
1
0
0
0
0
0
0
0
0
0
0
0
0
0
1
2
0
0
0
0
0
0
1
0
0
0
0
1
0
0
0
0
0
0
0
3
0
0
0
0
0
0
0
0
0
0
0
0
0
0
1


0
0
0
0
0
0
0
2
0
0
0
0
0
0
0
0
0
0
0
0
0
0
0
0
0
0
0
0
0
0
1
0
0
0
0
0
0
0
0
0
0
0
0
0
1
2
0
0
0
0
0
0
1
0
0
0
0
1
0
0
0
0
0
0
0
3
0
0
0
0
0
0
0
0
0
0
0
0
0
0
1


229
143
100
273
110
228
167
294
99
169
109
139
105
176
241
120
211
135
170
217
126
92
208
0
0
62
0
90
0
95
200
0
3
0
29
138
0
146
214
62
50
84
69
59
72
101
84
63
149
0
225
88
188
120
127
95
78
208
149
80
258
324
181
100
328
201
175
77
268
312
138
165
337
240
44
78
61
73
219
27
125


0
0
0
0
0
0
3
0
0
0
0
0
0
0
0
0
0
0
0
0
0
0
0
0
0
0
0
0
0
0
0
0
0
0
0
0
0
0
0
0
0
0
0
0
0
0
0
0
0
0
0
0
0
0
0
0
0
0
0
0
0
0
1
0
0
0
0
0
0
0
0
0
0
0
0
0
0
0
0
0
0


0
0
0
0
0
0
3
0
0
0
0
0
0
0
0
0
0
0
0
0
0
0
0
0
0
0
0
0
0
0
0
0
0
0
0
0
0
0
0
0
0
0
0
0
0
0
0
0
0
0
0
0
0
0
0
0
0
0
0
0
0
0
1
0
0
0
0
0
0
0
0
0
0
0
0
0
0
0
0
0
0


0
0
0
0
0
0
3
0
0
0
0
0
0
0
0
0
0
0
0
0
0
0
0
0
0
0
0
0
0
0
0
0
0
0
0
0
0
0
0
0
0
0
0
0
0
0
0
0
0
0
0
0
0
0
0
0
0
0
0
0
0
0
1
0
0
0
0
0
0
0
0
0
0
0
0
0
0
0
0
0
0


0
0
0
0
0
0
3
0
0
0
0
0
0
0
0
0
0
0
0
0
0
0
0
0
0
0
0
0
0
0
0
0
0
0
0
0
0
0
0
0
0
0
0
0
0
0
0
0
0
0
0
0
0
0
0
0
0
0
0
0
0
0
1
0
0
0
0
0
0
0
0
0
0
0
0
0
0
0
0
0
0


14
0
0
13
11
11
2
8
12
16
1
0
0
3
3
7
0
1
3
7
0
1
3
0
0
0
0
1
0
1
1
0
3
0
0
0
0
0
9
1
1
1
0
0
1
4
1
0
1
0
11
15
17
15
9
7
5
10
15
3
20
16
10
25
52
26
9
19
34
27
16
4
4
6
5
3
4
7
12
1
9


14
0
0
13
11
11
2
8
12
15
1
0
0
3
3
1
0
1
3
7
0
1
3
0
0
0
0
1
0
1
1
0
3
0
0
0
0
0
9
1
1
1
0
0
1
4
1
0
1
0
10
13
17
15
9
7
5
8
11
3
20
16
9
25
51
24
9
19
34
27
15
4
4
6
5
3
4
7
12
1
9


14
0
0
13
11
11
2
8
12
15
1
0
0
3
3
1
0
1
3
7
0
1
3
0
0
0
0
1
0
1
1
0
3
0
0
0
0
0
9
1
1
1
0
0
1
4
1
0
1
0
10
13
17
15
9
7
5
8
11
3
20
16
9
25
51
24
9
19
34
27
15
4
4
6
5
3
4
7
12
1
9


14
0
0
13
11
11
2
8
12
15
1
0
0
3
3
1
0
1
3
7
0
1
3
0
0
0
0
1
0
1
1
0
3
0
0
0
0
0
9
1
1
1
0
0
1
4
1
0
1
0
10
13
17
15
9
7
5
8
11
3
20
16
9
25
51
24
9
19
34
27
15
4
4
6
5
3
4
7
12
1
9


0
0
0
0
0
0
0
0
0
1
0
0
0
0
0
6
0
0
0
0
0
0
0
0
0
0
0
0
0
0
0
0
0
0
0
0
0
0
0
0
0
0
0
0
0
0
0
0
0
0
1
2
0
0
0
0
0
2
4
0
0
0
1
0
1
2
0
0
0
0
1
0
0
0
0
0
0
0
0
0
0


0
0
0
0
0
0
0
0
0
1
0
0
0
0
0
0
0
0
0
0
0
0
0
0
0
0
0
0
0
0
0
0
0
0
0
0
0
0
0
0
0
0
0
0
0
0
0
0
0
0
0
1
0
0
0
0
0
0
1
0
0
0
0
0
0
1
0
0
0
0
1
0
0
0
0
0
0
0
0
0
0


0
0
0
0
0
0
0
0
0
1
0
0
0
0
0
0
0
0
0
0
0
0
0
0
0
0
0
0
0
0
0
0
0
0
0
0
0
0
0
0
0
0
0
0
0
0
0
0
0
0
0
1
0
0
0
0
0
0
1
0
0
0
0
0
0
1
0
0
0
0
1
0
0
0
0
0
0
0
0
0
0


0
0
0
0
0
0
0
0
0
0
0
0
0
0
0
6
0
0
0
0
0
0
0
0
0
0
0
0
0
0
0
0
0
0
0
0
0
0
0
0
0
0
0
0
0
0
0
0
0
0
1
1
0
0
0
0
0
0
2
0
0
0
0
0
1
1
0
0
0
0
0
0
0
0
0
0
0
0
0
0
0


0
0
0
0
0
0
0
0
0
0
0
0
0
0
0
6
0
0
0
0
0
0
0
0
0
0
0
0
0
0
0
0
0
0
0
0
0
0
0
0
0
0
0
0
0
0
0
0
0
0
1
1
0
0
0
0
0
0
2
0
0
0
0
0
1
1
0
0
0
0
0
0
0
0
0
0
0
0
0
0
0


0
0
0
0
0
0
0
0
0
0
0
0
0
0
0
0
0
0
0
0
0
0
0
0
0
0
0
0
0
0
0
0
0
0
0
0
0
0
0
0
0
0
0
0
0
0
0
0
0
0
0
0
0
0
0
0
0
2
1
0
0
0
1
0
0
0
0
0
0
0
0
0
0
0
0
0
0
0
0
0
0


0
0
0
0
0
0
0
0
0
0
0
0
0
0
0
0
0
0
0
0
0
0
0
0
0
0
0
0
0
0
0
0
0
0
0
0
0
0
0
0
0
0
0
0
0
0
0
0
0
0
0
0
0
0
0
0
0
2
1
0
0
0
1
0
0
0
0
0
0
0
0
0
0
0
0
0
0
0
0
0
0


157
87
73
188
61
183
72
189
44
106
53
72
48
98
161
69
147
86
83
144
62
56
158
0
0
50
0
48
0
82
127
0
0
0
16
113
0
111
147
39
32
41
48
45
44
79
49
37
95
0
153
60
125
83
76
71
57
163
103
45
144
239
19
55
207
139
115
44
190
244
97
133
286
200
24
60
30
37
185
22
93


157
87
73
188
61
183
72
189
44
106
53
72
48
98
161
69
147
86
83
144
62
56
158
0
0
50
0
48
0
82
127
0
0
0
16
113
0
111
147
39
32
41
48
45
44
79
49
37
95
0
153
60
125
83
76
71
57
163
103
45
144
239
19
55
207
139
115
44
190
244
97
133
286
200
24
60
30
37
185
22
93


157
87
73
188
61
183
72
189
44
106
53
72
48
98
161
69
147
86
83
144
62
56
158
0
0
50
0
48
0
82
127
0
0
0
16
113
0
111
147
39
32
41
48
45
44
79
49
37
95
0
153
60
125
83
76
71
57
163
103
45
144
239
19
55
207
139
115
44
190
244
97
133
286
200
24
60
30
37
185
22
93


157
87
73
188
61
183
72
189
44
106
53
72
48
98
161
69
147
86
83
144
62
56
158
0
0
50
0
48
0
82
127
0
0
0
16
113
0
111
147
39
32
41
48
45
44
79
49
37
95
0
153
60
125
83
76
71
57
163
103
45
144
239
19
55
207
139
115
44
190
244
97
133
286
200
24
60
30
37
185
22
93


49
52
25
64
31
27
70
89
32
37
31
55
43
57
64
38
36
42
70
51
57
28
42
0
0
1
0
26
0
7
68
0
0
0
7
23
0
19
48
18
10
32
14
14
26
16
22
18
41
0
56
9
28
19
41
13
11
25
30
14
68
64
151
12
55
26
45
13
40
39
20
26
45
29
13
14
25
29
16
4
22


49
52
25
64
31
27
70
89
32
37
31
55
43
57
64
38
36
42
70
51
57
28
42
0
0
1
0
26
0
7
68
0
0
0
7
23
0
19
48
18
10
32
14
14
26
16
22
18
41
0
56
9
28
19
41
13
11
25
30
14
68
64
151
12
55
26
45
13
40
39
20
26
45
29
13
14
25
29
16
4
22


49
52
25
64
31
27
70
89
32
37
31
55
43
57
64
38
36
42
70
51
57
28
42
0
0
1
0
26
0
7
68
0
0
0
7
23
0
19
48
18
10
32
14
14
26
16
22
18
41
0
56
9
28
19
41
13
11
25
30
14
68
64
151
12
55
26
45
13
40
39
20
26
45
29
13
14
25
29
16
4
22


49
52
25
64
31
27
70
89
32
37
31
55
43
57
64
38
36
42
70
51
57
28
42
0
0
1
0
26
0
7
68
0
0
0
7
23
0
19
48
18
10
32
14
14
26
16
22
18
41
0
56
9
28
19
41
13
11
25
30
14
68
64
151
12
55
26
45
13
40
39
20
26
45
29
13
14
25
29
16
4
22


3
4
2
7
6
1
19
5
2
7
13
10
13
15
5
4
23
6
10
15
4
5
1
0
0
11
0
13
0
5
4
0
0
0
6
0
0
16
9
4
7
9
7
0
0
1
9
8
12
0
5
3
11
3
0
2
3
8
1
13
25
5
0
7
13
7
6
1
4
2
5
1
2
4
2
0
1
0
4
0
1


3
4
2
7
6
1
19
5
2
7
13
10
13
15
5
4
23
6
10
15
4
5
1
0
0
11
0
13
0
5
4
0
0
0
6
0
0
16
9
4
7
9
7
0
0
1
9
8
12
0
5
3
11
3
0
2
3
8
1
13
25
5
0
7
13
7
6
1
4
2
5
1
2
4
2
0
1
0
4
0
1


3
4
2
7
6
1
19
5
2
7
13
10
13
15
5
4
23
6
10
15
4
5
1
0
0
11
0
13
0
5
4
0
0
0
6
0
0
16
9
4
7
9
7
0
0
1
9
8
12
0
5
3
11
3
0
2
3
8
1
13
25
5
0
7
13
7
6
1
4
2
5
1
2
4
2
0
1
0
4
0
1


3
4
2
7
6
1
19
5
2
7
13
10
13
15
5
4
23
6
10
15
4
5
1
0
0
11
0
13
0
5
4
0
0
0
6
0
0
16
9
4
7
9
7
0
0
1
9
8
12
0
5
3
11
3
0
2
3
8
1
13
25
5
0
7
13
7
6
1
4
2
5
1
2
4
2
0
1
0
4
0
1


6
0
0
1
1
6
1
3
9
3
11
2
1
3
8
2
5
0
4
0
3
2
4
0
0
0
0
2
0
0
0
0
0
0
0
2
0
0
1
0
0
1
0
0
1
1
3
0
0
0
0
1
7
0
1
2
2
2
0
5
1
0
0
1
1
3
0
0
0
0
0
1
0
1
0
1
1
0
2
0
0


6
0
0
1
1
6
1
3
9
3
11
2
1
3
8
2
5
0
4
0
3
2
4
0
0
0
0
2
0
0
0
0
0
0
0
2
0
0
1
0
0
1
0
0
1
1
3
0
0
0
0
1
7
0
1
2
2
2
0
5
1
0
0
1
1
3
0
0
0
0
0
1
0
1
0
1
1
0
2
0
0


6
0
0
1
1
6
1
3
9
3
11
2
1
3
8
2
5
0
4
0
3
2
4
0
0
0
0
2
0
0
0
0
0
0
0
2
0
0
1
0
0
1
0
0
1
1
3
0
0
0
0
1
7
0
1
2
2
2
0
5
1
0
0
1
1
3
0
0
0
0
0
1
0
1
0
1
1
0
2
0
0


6
0
0
1
1
6
1
3
9
3
11
2
1
3
8
2
5
0
4
0
3
2
4
0
0
0
0
2
0
0
0
0
0
0
0
2
0
0
1
0
0
1
0
0
1
1
3
0
0
0
0
1
7
0
1
2
2
2
0
5
1
0
0
1
1
3
0
0
0
0
0
1
0
1
0
1
1
0
2
0
0


3
1
2
0
0
1
2
0
0
2
0
0
0
0
0
0
0
0
0
1
0
1
0
0
0
0
0
0
0
1
0
0
0
0
0
0
0
0
1
1
0
1
0
0
2
0
1
2
1
0
0
0
3
0
0
0
0
0
1
4
0
0
1
0
2
0
2
2
0
0
0
1
0
1
0
1
0
2
0
0
0


3
1
2
0
0
1
2
0
0
2
0
0
0
0
0
0
0
0
0
1
0
1
0
0
0
0
0
0
0
1
0
0
0
0
0
0
0
0
1
1
0
1
0
0
2
0
1
2
1
0
0
0
3
0
0
0
0
0
1
4
0
0
1
0
2
0
2
2
0
0
0
1
0
1
0
1
0
2
0
0
0


3
1
2
0
0
1
2
0
0
2
0
0
0
0
0
0
0
0
0
1
0
1
0
0
0
0
0
0
0
1
0
0
0
0
0
0
0
0
1
1
0
1
0
0
2
0
1
2
1
0
0
0
3
0
0
0
0
0
1
4
0
0
1
0
2
0
2
2
0
0
0
1
0
1
0
1
0
2
0
0
0


3
1
2
0
0
1
2
0
0
2
0
0
0
0
0
0
0
0
0
1
0
1
0
0
0
0
0
0
0
1
0
0
0
0
0
0
0
0
1
1
0
1
0
0
2
0
1
2
1
0
0
0
3
0
0
0
0
0
1
4
0
0
1
0
2
0
2
2
0
0
0
1
0
1
0
1
0
2
0
0
0


3
1
2
0
0
1
2
0
0
2
0
0
0
0
0
0
0
0
0
1
0
1
0
0
0
0
0
0
0
1
0
0
0
0
0
0
0
0
1
1
0
1
0
0
2
0
1
2
1
0
0
0
3
0
0
0
0
0
1
4
0
0
1
0
2
0
2
2
0
0
0
1
0
1
0
1
0
2
0
0
0


16
7
0
10
4
6
7
17
2
0
0
2
1
3
1
1
0
0
0
5
1
0
6
0
0
1
0
0
0
5
4
0
0
0
2
2
0
9
6
1
2
1
1
0
0
4
8
1
2
0
5
0
8
10
1
1
5
6
2
1
12
5
0
3
12
4
1
2
19
0
4
4
0
4
0
0
4
1
2
0
3


16
7
0
10
4
6
7
17
2
0
0
2
1
3
1
1
0
0
0
5
1
0
6
0
0
1
0
0
0
5
4
0
0
0
2
2
0
9
6
1
2
1
1
0
0
4
8
1
2
0
5
0
8
10
1
1
5
6
2
1
12
5
0
3
12
4
1
2
19
0
4
4
0
4
0
0
4
1
2
0
3


16
7
0
10
4
6
7
17
2
0
0
2
1
3
1
1
0
0
0
5
1
0
6
0
0
1
0
0
0
5
4
0
0
0
2
2
0
9
6
1
2
1
1
0
0
4
8
1
2
0
5
0
8
10
1
1
5
6
2
1
12
5
0
3
12
4
1
2
19
0
4
4
0
4
0
0
4
1
2
0
3


16
7
0
10
4
6
7
17
2
0
0
2
1
3
1
1
0
0
0
5
1
0
6
0
0
1
0
0
0
5
4
0
0
0
2
2
0
9
6
1
2
1
1
0
0
4
8
1
2
0
5
0
8
10
1
1
5
6
2
1
12
5
0
3
12
4
1
2
19
0
4
4
0
4
0
0
4
1
2
0
3


16
7
0
10
4
6
7
17
2
0
0
2
1
3
1
1
0
0
0
5
1
0
6
0
0
1
0
0
0
5
4
0
0
0
2
2
0
9
6
1
2
1
1
0
0
4
8
1
2
0
5
0
8
10
1
1
5
6
2
1
12
5
0
3
12
4
1
2
19
0
4
4
0
4
0
0
4
1
2
0
3


8
101
3
5
2
7
3
11
13
2
2
16
6
20
6
17
7
50
7
11
194
14
30
0
31
20
0
6
0
64
49
0
0
0
4
2
0
26
27
7
9
8
15
13
20
18
18
8
11
0
17
4
6
9
41
15
12
5
7
16
2
15
18
63
3
2
0
7
8
0
2
11
12
3
8
3
4
6
1
2
3


8
101
3
5
2
7
3
11
13
2
2
16
6
20
6
17
7
50
7
11
194
14
30
0
31
20
0
6
0
64
49
0
0
0
4
2
0
26
27
7
9
8
15
13
20
18
18
8
11
0
17
4
6
9
41
15
12
5
7
16
2
15
18
63
3
2
0
7
8
0
2
11
12
3
8
3
4
6
1
2
3


8
101
3
5
2
7
3
11
13
2
2
16
6
20
6
17
7
50
7
11
194
14
30
0
31
20
0
6
0
64
49
0
0
0
4
2
0
26
27
7
9
8
15
13
20
18
18
8
11
0
17
4
6
9
41
15
12
5
7
16
2
15
18
63
3
2
0
7
8
0
2
11
12
3
8
3
4
6
1
2
3


8
101
3
5
2
7
3
11
13
2
2
16
6
20
6
17
7
50
7
11
194
14
30
0
31
20
0
6
0
64
49
0
0
0
4
2
0
26
27
7
9
8
15
13
20
18
18
8
11
0
17
4
6
9
41
15
12
5
7
16
2
15
18
63
3
2
0
7
8
0
2
11
12
3
8
3
4
6
1
2
3


8
101
3
5
2
7
3
11
13
2
2
16
6
20
6
17
7
50
7
11
194
14
30
0
31
20
0
6
0
64
49
0
0
0
4
2
0
26
27
7
9
8
15
13
20
18
18
8
11
0
17
4
6
9
41
15
12
5
7
16
2
15
18
63
3
2
0
7
8
0
2
11
12
3
8
3
4
6
1
2
3


8
101
3
5
2
7
3
11
13
2
2
16
6
20
6
17
7
50
7
11
194
14
30
0
31
20
0
6
0
64
49
0
0
0
4
2
0
26
27
7
9
8
15
13
20
18
18
8
11
0
17
4
6
9
41
15
12
5
7
16
2
15
18
63
3
2
0
7
8
0
2
11
12
3
8
3
4
6
1
2
3


59
7
9
41
15
15
7
17
2
53
1
6
0
2
2
3
0
0
1
7
0
0
0
0
0
0
0
0
0
0
1
0
0
0
0
0
0
0
7
1
0
1
0
0
0
0
0
0
2
0
29
12
6
12
13
61
5
13
8
12
60
32
2
22
102
32
40
17
28
36
29
1
5
12
46
5
0
9
54
0
51


59
7
9
41
15
15
7
17
2
53
1
6
0
2
2
3
0
0
1
7
0
0
0
0
0
0
0
0
0
0
1
0
0
0
0
0
0
0
7
1
0
1
0
0
0
0
0
0
2
0
29
12
6
12
13
61
5
13
8
12
60
32
2
22
102
32
40
17
28
36
29
1
5
12
46
5
0
9
54
0
51


59
7
9
41
15
15
7
17
2
53
1
6
0
2
2
3
0
0
1
7
0
0
0
0
0
0
0
0
0
0
1
0
0
0
0
0
0
0
7
1
0
1
0
0
0
0
0
0
2
0
29
12
6
12
13
61
5
13
8
12
60
32
2
22
102
32
40
17
28
36
29
1
5
12
46
5
0
9
54
0
51


59
7
9
41
15
15
7
17
2
53
1
6
0
2
2
3
0
0
1
7
0
0
0
0
0
0
0
0
0
0
1
0
0
0
0
0
0
0
7
1
0
1
0
0
0
0
0
0
2
0
29
12
6
12
13
61
5
13
8
12
60
32
2
22
102
32
40
17
28
36
29
1
5
12
46
5
0
9
54
0
51


59
7
9
41
15
15
7
17
2
53
1
6
0
2
2
3
0
0
1
7
0
0
0
0
0
0
0
0
0
0
1
0
0
0
0
0
0
0
7
1
0
1
0
0
0
0
0
0
2
0
29
12
6
12
13
61
5
13
8
12
60
32
2
22
102
32
40
17
28
36
29
1
5
12
46
5
0
9
54
0
51


59
7
9
41
15
15
7
17
2
53
1
6
0
2
2
3
0
0
1
7
0
0
0
0
0
0
0
0
0
0
1
0
0
0
0
0
0
0
7
1
0
1
0
0
0
0
0
0
2
0
29
12
6
12
13
61
5
13
8
12
60
32
2
22
102
32
40
17
28
36
29
1
5
12
46
5
0
9
54
0
51


4
4
3
2
1
3
2
0
2
1
3
3
1
1
0
12
10
3
15
4
23
20
24
0
0
10
34
1
0
6
3
0
16
0
5
0
0
1
14
8
6
358
4
2
4
3
5
12
5
0
12
14
10
7
32
19
9
8
2
3
26
22
0
4
5
11
0
18
8
15
20
4
9
8
0
0
3
6
4
8
1


4
4
3
2
1
3
1
0
2
1
3
3
1
1
0
12
10
3
15
4
6
20
24
0
0
10
34
1
0
6
3
0
16
0
5
0
0
1
14
8
6
358
4
2
4
3
5
12
5
0
11
12
10
7
32
19
9
7
2
3
26
21
0
4
5
11
0
18
8
15
20
4
9
8
0
0
3
5
4
5
1


4
4
3
2
1
3
1
0
2
1
3
3
1
1
0
12
9
3
15
4
6
20
24
0
0
10
34
1
0
6
3
0
16
0
5
0
0
1
14
8
6
358
4
2
4
3
5
12
5
0
11
12
10
7
32
19
9
7
2
3
26
21
0
4
5
11
0
18
8
15
20
4
9
8
0
0
3
5
4
5
1


4
4
3
2
1
3
1
0
2
1
3
3
1
1
0
12
9
3
15
4
6
20
24
0
0
10
34
1
0
6
3
0
16
0
5
0
0
1
14
8
6
358
4
2
4
3
5
12
5
0
11
12
10
7
32
19
9
7
2
3
26
21
0
4
5
11
0
18
8
15
20
4
9
8
0
0
3
5
4
5
1


4
4
3
2
1
3
1
0
2
1
3
3
1
1
0
12
9
3
15
4
6
20
24
0
0
10
34
1
0
6
3
0
16
0
5
0
0
1
14
8
6
358
4
2
4
3
5
12
5
0
11
12
10
7
32
19
9
7
2
3
26
21
0
4
5
11
0
18
8
15
20
4
9
8
0
0
3
5
4
5
1


4
4
3
2
1
3
1
0
2
1
3
3
1
1
0
12
9
3
15
4
6
20
24
0
0
10
34
1
0
6
3
0
16
0
5
0
0
1
14
8
6
358
4
2
4
3
5
12
5
0
11
12
10
7
32
19
9
7
2
3
26
21
0
4
5
11
0
18
8
15
20
4
9
8
0
0
3
5
4
5
1


0
0
0
0
0
0
0
0
0
0
0
0
0
0
0
0
1
0
0
0
0
0
0
0
0
0
0
0
0
0
0
0
0
0
0
0
0
0
0
0
0
0
0
0
0
0
0
0
0
0
0
0
0
0
0
0
0
0
0
0
0
0
0
0
0
0
0
0
0
0
0
0
0
0
0
0
0
0
0
0
0


0
0
0
0
0
0
0
0
0
0
0
0
0
0
0
0
1
0
0
0
0
0
0
0
0
0
0
0
0
0
0
0
0
0
0
0
0
0
0
0
0
0
0
0
0
0
0
0
0
0
0
0
0
0
0
0
0
0
0
0
0
0
0
0
0
0
0
0
0
0
0
0
0
0
0
0
0
0
0
0
0


0
0
0
0
0
0
0
0
0
0
0
0
0
0
0
0
1
0
0
0
0
0
0
0
0
0
0
0
0
0
0
0
0
0
0
0
0
0
0
0
0
0
0
0
0
0
0
0
0
0
0
0
0
0
0
0
0
0
0
0
0
0
0
0
0
0
0
0
0
0
0
0
0
0
0
0
0
0
0
0
0


0
0
0
0
0
0
0
0
0
0
0
0
0
0
0
0
1
0
0
0
0
0
0
0
0
0
0
0
0
0
0
0
0
0
0
0
0
0
0
0
0
0
0
0
0
0
0
0
0
0
0
0
0
0
0
0
0
0
0
0
0
0
0
0
0
0
0
0
0
0
0
0
0
0
0
0
0
0
0
0
0


0
0
0
0
0
0
1
0
0
0
0
0
0
0
0
0
0
0
0
0
0
0
0
0
0
0
0
0
0
0
0
0
0
0
0
0
0
0
0
0
0
0
0
0
0
0
0
0
0
0
0
0
0
0
0
0
0
0
0
0
0
0
0
0
0
0
0
0
0
0
0
0
0
0
0
0
0
0
0
0
0


0
0
0
0
0
0
1
0
0
0
0
0
0
0
0
0
0
0
0
0
0
0
0
0
0
0
0
0
0
0
0
0
0
0
0
0
0
0
0
0
0
0
0
0
0
0
0
0
0
0
0
0
0
0
0
0
0
0
0
0
0
0
0
0
0
0
0
0
0
0
0
0
0
0
0
0
0
0
0
0
0


0
0
0
0
0
0
1
0
0
0
0
0
0
0
0
0
0
0
0
0
0
0
0
0
0
0
0
0
0
0
0
0
0
0
0
0
0
0
0
0
0
0
0
0
0
0
0
0
0
0
0
0
0
0
0
0
0
0
0
0
0
0
0
0
0
0
0
0
0
0
0
0
0
0
0
0
0
0
0
0
0


0
0
0
0
0
0
1
0
0
0
0
0
0
0
0
0
0
0
0
0
0
0
0
0
0
0
0
0
0
0
0
0
0
0
0
0
0
0
0
0
0
0
0
0
0
0
0
0
0
0
0
0
0
0
0
0
0
0
0
0
0
0
0
0
0
0
0
0
0
0
0
0
0
0
0
0
0
0
0
0
0


0
0
0
0
0
0
1
0
0
0
0
0
0
0
0
0
0
0
0
0
0
0
0
0
0
0
0
0
0
0
0
0
0
0
0
0
0
0
0
0
0
0
0
0
0
0
0
0
0
0
0
0
0
0
0
0
0
0
0
0
0
0
0
0
0
0
0
0
0
0
0
0
0
0
0
0
0
0
0
0
0


0
0
0
0
0
0
0
0
0
0
0
0
0
0
0
0
0
0
0
0
17
0
0
0
0
0
0
0
0
0
0
0
0
0
0
0
0
0
0
0
0
0
0
0
0
0
0
0
0
0
0
0
0
0
0
0
0
1
0
0
0
1
0
0
0
0
0
0
0
0
0
0
0
0
0
0
0
1
0
3
0


0
0
0
0
0
0
0
0
0
0
0
0
0
0
0
0
0
0
0
0
17
0
0
0
0
0
0
0
0
0
0
0
0
0
0
0
0
0
0
0
0
0
0
0
0
0
0
0
0
0
0
0
0
0
0
0
0
0
0
0
0
1
0
0
0
0
0
0
0
0
0
0
0
0
0
0
0
0
0
1
0


0
0
0
0
0
0
0
0
0
0
0
0
0
0
0
0
0
0
0
0
17
0
0
0
0
0
0
0
0
0
0
0
0
0
0
0
0
0
0
0
0
0
0
0
0
0
0
0
0
0
0
0
0
0
0
0
0
0
0
0
0
0
0
0
0
0
0
0
0
0
0
0
0
0
0
0
0
0
0
0
0


0
0
0
0
0
0
0
0
0
0
0
0
0
0
0
0
0
0
0
0
17
0
0
0
0
0
0
0
0
0
0
0
0
0
0
0
0
0
0
0
0
0
0
0
0
0
0
0
0
0
0
0
0
0
0
0
0
0
0
0
0
0
0
0
0
0
0
0
0
0
0
0
0
0
0
0
0
0
0
0
0


0
0
0
0
0
0
0
0
0
0
0
0
0
0
0
0
0
0
0
0
17
0
0
0
0
0
0
0
0
0
0
0
0
0
0
0
0
0
0
0
0
0
0
0
0
0
0
0
0
0
0
0
0
0
0
0
0
0
0
0
0
0
0
0
0
0
0
0
0
0
0
0
0
0
0
0
0
0
0
0
0


0
0
0
0
0
0
0
0
0
0
0
0
0
0
0
0
0
0
0
0
0
0
0
0
0
0
0
0
0
0
0
0
0
0
0
0
0
0
0
0
0
0
0
0
0
0
0
0
0
0
0
0
0
0
0
0
0
0
0
0
0
1
0
0
0
0
0
0
0
0
0
0
0
0
0
0
0
0
0
1
0


0
0
0
0
0
0
0
0
0
0
0
0
0
0
0
0
0
0
0
0
0
0
0
0
0
0
0
0
0
0
0
0
0
0
0
0
0
0
0
0
0
0
0
0
0
0
0
0
0
0
0
0
0
0
0
0
0
0
0
0
0
1
0
0
0
0
0
0
0
0
0
0
0
0
0
0
0
0
0
1
0


0
0
0
0
0
0
0
0
0
0
0
0
0
0
0
0
0
0
0
0
0
0
0
0
0
0
0
0
0
0
0
0
0
0
0
0
0
0
0
0
0
0
0
0
0
0
0
0
0
0
0
0
0
0
0
0
0
0
0
0
0
1
0
0
0
0
0
0
0
0
0
0
0
0
0
0
0
0
0
1
0


0
0
0
0
0
0
0
0
0
0
0
0
0
0
0
0
0
0
0
0
0
0
0
0
0
0
0
0
0
0
0
0
0
0
0
0
0
0
0
0
0
0
0
0
0
0
0
0
0
0
0
0
0
0
0
0
0
1
0
0
0
0
0
0
0
0
0
0
0
0
0
0
0
0
0
0
0
1
0
2
0


0
0
0
0
0
0
0
0
0
0
0
0
0
0
0
0
0
0
0
0
0
0
0
0
0
0
0
0
0
0
0
0
0
0
0
0
0
0
0
0
0
0
0
0
0
0
0
0
0
0
0
0
0
0
0
0
0
1
0
0
0
0
0
0
0
0
0
0
0
0
0
0
0
0
0
0
0
1
0
2
0


0
0
0
0
0
0
0
0
0
0
0
0
0
0
0
0
0
0
0
0
0
0
0
0
0
0
0
0
0
0
0
0
0
0
0
0
0
0
0
0
0
0
0
0
0
0
0
0
0
0
0
0
0
0
0
0
0
1
0
0
0
0
0
0
0
0
0
0
0
0
0
0
0
0
0
0
0
1
0
2
0


0
0
0
0
0
0
0
0
0
0
0
0
0
0
0
0
0
0
0
0
0
0
0
0
0
0
0
0
0
0
0
0
0
0
0
0
0
0
0
0
0
0
0
0
0
0
0
0
0
0
0
0
0
0
0
0
0
1
0
0
0
0
0
0
0
0
0
0
0
0
0
0
0
0
0
0
0
1
0
2
0


0
0
0
0
0
0
0
0
0
0
0
0
0
0
0
0
0
0
0
0
0
0
0
0
0
0
0
0
0
0
0
0
0
0
0
0
0
0
0
0
0
0
0
0
0
0
0
0
0
0
1
2
0
0
0
0
0
0
0
0
0
0
0
0
0
0
0
0
0
0
0
0
0
0
0
0
0
0
0
0
0


0
0
0
0
0
0
0
0
0
0
0
0
0
0
0
0
0
0
0
0
0
0
0
0
0
0
0
0
0
0
0
0
0
0
0
0
0
0
0
0
0
0
0
0
0
0
0
0
0
0
1
2
0
0
0
0
0
0
0
0
0
0
0
0
0
0
0
0
0
0
0
0
0
0
0
0
0
0
0
0
0


0
0
0
0
0
0
0
0
0
0
0
0
0
0
0
0
0
0
0
0
0
0
0
0
0
0
0
0
0
0
0
0
0
0
0
0
0
0
0
0
0
0
0
0
0
0
0
0
0
0
1
2
0
0
0
0
0
0
0
0
0
0
0
0
0
0
0
0
0
0
0
0
0
0
0
0
0
0
0
0
0


0
0
0
0
0
0
0
0
0
0
0
0
0
0
0
0
0
0
0
0
0
0
0
0
0
0
0
0
0
0
0
0
0
0
0
0
0
0
0
0
0
0
0
0
0
0
0
0
0
0
1
2
0
0
0
0
0
0
0
0
0
0
0
0
0
0
0
0
0
0
0
0
0
0
0
0
0
0
0
0
0


0
0
0
0
0
0
0
0
0
0
0
0
0
0
0
0
0
0
0
0
0
0
0
0
0
0
0
0
0
0
0
0
0
0
0
0
0
0
0
0
0
0
0
0
0
0
0
0
0
0
1
2
0
0
0
0
0
0
0
0
0
0
0
0
0
0
0
0
0
0
0
0
0
0
0
0
0
0
0
0
0


180
361
151
254
256
344
214
189
220
426
861
339
427
178
667
2251
168
1055
2194
358
1265
262
353
6022
3439
621
5739
454
6649
195
244
968
626
781
240
646
4557
55
97
354
190
176
146
227
415
356
249
590
353
874
128
324
141
238
165
268
126
391
445
157
467
164
505
107
291
182
162
155
53
625
192
541
516
373
177
369
453
385
215
1457
107


167
349
147
228
238
312
202
186
206
408
836
293
417
154
649
2182
158
980
2180
348
802
217
276
5732
3375
563
5481
445
6397
183
239
942
584
763
230
617
4457
50
95
339
188
166
137
219
401
345
244
575
336
818
118
297
130
228
155
253
119
340
223
147
454
151
493
94
265
163
154
147
51
598
167
506
474
259
143
339
342
280
183
1441
96


166
345
141
227
229
306
199
181
202
404
833
291
416
151
645
2176
155
976
2178
342
787
206
266
2437
1600
427
2531
272
2571
158
228
819
435
729
115
600
433
38
89
333
181
153
119
188
385
330
236
155
333
98
111
289
129
223
143
250
114
336
216
144
446
146
488
90
264
161
153
142
47
517
165
497
463
254
143
331
336
272
173
1438
94


0
0
0
0
0
2
1
0
0
0
1
0
0
0
1
0
0
0
0
0
3
1
0
0
0
0
0
0
0
0
0
0
0
0
0
8
0
0
0
0
0
0
1
0
1
2
0
0
0
0
0
0
0
0
0
0
0
0
0
0
2
0
2
0
0
0
1
0
0
0
0
0
0
0
0
0
0
0
0
0
0


0
0
0
0
0
2
1
0
0
0
1
0
0
0
1
0
0
0
0
0
3
1
0
0
0
0
0
0
0
0
0
0
0
0
0
8
0
0
0
0
0
0
1
0
1
2
0
0
0
0
0
0
0
0
0
0
0
0
0
0
2
0
2
0
0
0
1
0
0
0
0
0
0
0
0
0
0
0
0
0
0


0
0
0
0
0
2
1
0
0
0
1
0
0
0
1
0
0
0
0
0
3
1
0
0
0
0
0
0
0
0
0
0
0
0
0
8
0
0
0
0
0
0
1
0
1
2
0
0
0
0
0
0
0
0
0
0
0
0
0
0
2
0
2
0
0
0
1
0
0
0
0
0
0
0
0
0
0
0
0
0
0


14
6
3
9
2
5
3
14
5
10
11
3
2
6
3
9
6
14
6
9
65
20
12
9
12
41
38
10
0
20
2
40
0
0
6
5
0
0
0
33
11
8
4
20
61
30
27
3
81
0
7
5
2
22
5
17
5
24
1
9
18
3
22
2
25
7
5
2
5
31
3
14
17
7
12
7
13
19
5
7
3


0
0
0
0
0
0
0
0
0
0
0
0
0
0
0
0
0
0
0
1
0
1
0
0
0
1
0
0
0
0
0
0
0
0
6
1
0
0
0
0
0
0
0
0
0
0
0
0
0
0
2
4
2
6
1
3
1
0
0
2
8
1
11
1
4
2
0
1
0
0
0
2
3
1
2
2
1
6
0
0
0


0
0
0
0
0
0
0
0
0
0
0
0
0
0
0
0
0
0
0
1
0
1
0
0
0
1
0
0
0
0
0
0
0
0
6
1
0
0
0
0
0
0
0
0
0
0
0
0
0
0
2
4
2
6
1
3
1
0
0
2
8
1
11
1
4
2
0
1
0
0
0
2
3
1
2
2
1
6
0
0
0


14
6
3
9
2
5
3
14
5
10
9
3
2
6
3
9
5
14
6
8
65
19
12
9
12
40
38
10
0
20
2
40
0
0
0
4
0
0
0
33
11
8
4
20
61
30
27
3
81
0
5
1
0
16
4
14
4
24
1
7
10
2
11
1
21
5
5
1
5
31
3
12
14
6
10
5
12
13
5
7
3


14
6
3
9
2
5
3
14
5
10
9
3
2
6
3
9
5
14
6
8
65
19
12
9
12
40
38
10
0
20
2
40
0
0
0
4
0
0
0
33
11
8
4
20
61
30
27
3
81
0
5
1
0
16
4
14
4
24
1
7
10
2
11
1
21
5
5
1
5
31
3
12
14
6
10
5
12
13
5
7
3


0
0
0
0
0
0
0
0
0
0
2
0
0
0
0
0
1
0
0
0
0
0
0
0
0
0
0
0
0
0
0
0
0
0
0
0
0
0
0
0
0
0
0
0
0
0
0
0
0
0
0
0
0
0
0
0
0
0
0
0
0
0
0
0
0
0
0
0
0
0
0
0
0
0
0
0
0
0
0
0
0


0
0
0
0
0
0
0
0
0
0
2
0
0
0
0
0
1
0
0
0
0
0
0
0
0
0
0
0
0
0
0
0
0
0
0
0
0
0
0
0
0
0
0
0
0
0
0
0
0
0
0
0
0
0
0
0
0
0
0
0
0
0
0
0
0
0
0
0
0
0
0
0
0
0
0
0
0
0
0
0
0


53
180
78
75
64
110
53
37
43
94
394
99
163
37
370
1207
45
409
763
82
166
90
105
110
8
128
165
94
413
37
62
433
121
335
35
144
308
20
35
95
55
39
37
32
94
78
106
15
75
98
23
78
29
84
46
76
31
87
77
34
149
31
138
21
73
55
22
20
11
82
30
105
123
37
25
123
70
72
47
427
18


0
0
0
1
1
1
0
0
0
0
0
1
0
0
0
0
0
0
0
0
0
6
0
1
0
0
0
0
4
0
0
0
0
0
0
0
0
0
0
0
0
1
0
0
0
3
0
0
0
0
0
1
1
0
0
0
0
0
0
0
0
0
0
0
0
0
0
0
0
0
1
0
2
0
0
2
0
2
0
0
0


0
0
0
1
1
1
0
0
0
0
0
1
0
0
0
0
0
0
0
0
0
6
0
1
0
0
0
0
4
0
0
0
0
0
0
0
0
0
0
0
0
1
0
0
0
3
0
0
0
0
0
1
1
0
0
0
0
0
0
0
0
0
0
0
0
0
0
0
0
0
1
0
2
0
0
2
0
2
0
0
0


34
161
61
59
44
82
38
20
23
81
368
82
156
26
339
1171
30
376
721
69
111
70
88
0
0
101
165
85
70
15
39
347
67
260
15
89
177
12
28
82
26
26
19
17
64
46
93
8
46
97
15
33
17
47
32
53
18
57
56
23
103
20
109
13
53
42
9
10
2
36
14
49
65
19
9
49
29
28
27
388
12


1
1
0
0
1
0
0
0
0
0
1
0
0
0
0
0
0
0
5
0
7
7
4
0
0
2
89
3
70
0
0
12
0
0
0
1
0
0
2
1
1
0
0
0
4
0
1
0
0
0
0
0
0
0
0
0
1
0
0
0
1
0
1
1
1
0
0
0
0
0
0
1
2
0
0
0
0
0
0
0
0


0
0
0
0
1
0
1
0
0
0
8
4
7
0
1
5
0
16
24
2
2
2
0
0
0
0
0
1
0
0
1
0
0
0
0
7
0
0
1
4
3
0
1
0
2
3
1
0
2
0
1
0
1
3
1
0
1
4
1
2
2
0
3
1
0
0
0
0
0
0
1
4
2
0
0
0
1
0
1
0
0


3
0
0
0
1
5
0
2
0
0
0
0
0
1
2
0
0
1
0
2
0
2
3
0
0
0
0
0
0
0
0
0
0
0
0
4
0
0
0
0
0
0
0
0
0
1
0
0
0
0
1
0
0
1
0
2
0
1
0
0
0
1
0
0
0
5
0
0
0
0
0
0
0
0
0
1
0
0
0
0
0


0
0
0
0
0
0
0
0
0
0
0
0
0
0
0
0
0
0
0
0
0
0
4
0
0
18
0
0
0
0
0
0
0
0
0
0
0
0
0
0
0
0
0
0
0
0
0
0
0
0
0
0
0
0
0
0
0
0
0
0
0
0
0
0
0
0
0
0
0
0
0
0
0
0
0
0
0
0
0
0
0


0
0
0
0
0
1
0
0
0
0
0
0
0
0
0
0
0
0
0
0
0
0
0
0
0
0
0
0
0
0
0
0
0
0
0
0
0
0
0
0
0
0
0
0
0
0
0
0
0
0
0
0
0
0
0
0
0
0
0
0
0
0
0
0
0
0
0
0
0
0
0
0
0
0
0
0
0
0
0
0
0


0
0
0
0
0
0
0
0
0
0
0
0
1
0
0
0
0
0
0
0
0
0
0
0
0
0
0
0
0
0
0
0
0
0
0
0
0
0
0
0
0
0
0
0
0
0
0
0
0
0
0
0
0
0
0
0
0
0
0
0
0
0
0
0
0
0
0
0
0
0
0
0
0
0
0
0
0
0
0
0
0


0
0
0
0
0
0
1
0
0
0
0
0
0
1
1
0
0
0
0
1
6
1
1
0
0
0
0
0
0
2
1
0
0
0
4
0
0
0
0
0
0
0
0
0
0
2
2
0
0
0
1
1
0
0
0
0
0
0
0
0
0
0
0
0
0
0
0
0
0
0
0
0
1
1
1
0
0
0
0
0
0


19
148
56
41
28
56
29
11
10
56
329
68
124
19
314
1037
28
320
598
52
51
47
58
0
0
67
0
81
0
9
19
256
34
83
0
47
0
12
21
60
16
21
8
13
27
19
82
2
31
97
9
12
9
39
13
20
13
35
35
14
66
14
70
3
44
29
6
5
1
13
10
10
6
4
0
15
6
3
13
2
1


2
5
0
0
0
0
0
0
0
0
1
1
0
0
0
0
0
0
0
0
0
0
0
0
0
0
0
0
0
0
10
0
5
1
0
0
0
0
0
0
0
0
0
0
0
0
0
0
0
0
0
0
0
0
0
0
0
1
0
0
0
0
0
1
1
0
1
0
0
8
0
0
0
0
0
0
0
0
0
0
0


4
1
0
2
1
2
0
1
2
8
4
3
0
2
2
39
0
5
18
5
0
0
2
0
0
2
0
0
0
0
0
0
0
0
0
0
0
0
0
0
0
0
1
0
0
0
1
0
0
0
0
0
0
0
0
0
1
1
0
0
1
1
2
0
0
2
0
0
0
0
0
1
0
0
0
1
0
1
0
3
0


0
0
0
0
0
1
1
0
0
0
2
0
0
0
3
0
0
10
26
0
6
0
0
0
0
0
0
0
0
0
0
20
0
0
0
0
0
0
0
0
0
0
0
0
0
0
0
0
0
0
1
0
0
0
0
5
0
0
0
0
0
0
0
0
0
0
0
0
0
0
0
2
1
0
0
10
0
1
3
35
0


0
0
0
0
1
0
0
0
0
0
0
0
0
0
0
0
0
0
0
0
0
2
1
0
0
0
0
0
0
0
0
1
28
29
0
0
0
0
0
0
0
0
0
0
0
0
0
0
0
0
0
0
1
0
0
0
0
1
0
0
0
0
0
0
0
0
0
0
0
0
0
0
0
0
0
0
0
0
0
0
0


0
0
0
1
0
1
0
0
0
3
1
0
0
1
0
0
0
0
0
0
3
0
1
0
0
0
0
0
0
0
0
0
0
11
0
0
0
0
0
1
0
1
2
1
2
4
0
2
0
0
0
4
0
2
2
9
1
7
4
0
7
2
7
4
4
1
0
1
0
10
0
14
9
5
2
4
4
6
8
46
3


0
0
0
0
0
0
0
0
0
0
2
0
0
0
0
0
0
0
1
0
0
0
0
0
0
0
0
0
0
0
0
0
0
0
0
0
0
0
0
0
0
0
0
0
0
0
0
0
0
0
0
0
0
0
0
0
0
0
0
0
0
0
0
0
0
0
0
0
0
0
0
0
0
0
0
0
0
0
0
1
0


3
0
3
0
0
2
2
0
0
1
0
0
0
0
0
0
0
1
14
0
6
0
4
0
0
4
0
0
0
1
3
34
0
32
0
2
0
0
0
0
1
0
0
0
0
2
0
1
0
0
0
0
0
0
1
0
0
0
0
4
0
0
2
0
0
0
0
1
0
0
0
0
0
0
0
3
0
0
0
17
2


0
0
0
0
0
0
0
0
0
0
0
0
0
0
2
0
0
0
0
0
0
0
0
0
0
4
0
0
0
0
0
0
0
0
0
0
0
0
0
0
0
0
0
0
0
0
0
0
0
0
0
0
0
0
0
0
0
0
0
0
0
0
0
0
0
0
0
0
0
0
0
0
0
0
0
0
0
0
0
0
0


0
0
0
0
0
0
0
0
0
0
0
0
0
0
0
0
0
0
1
1
5
0
0
0
0
0
0
0
0
0
0
0
0
0
0
0
0
0
0
0
0
0
0
0
0
0
0
0
0
0
0
0
0
0
0
0
0
0
0
0
0
0
0
0
0
0
0
0
0
0
0
0
0
0
0
0
0
0
0
0
0


0
0
0
0
0
0
0
0
0
0
0
0
0
1
0
0
0
0
0
0
1
0
1
0
0
0
75
0
0
0
0
0
0
0
0
0
0
0
0
0
0
0
0
0
0
0
0
0
0
0
0
0
0
0
0
1
0
0
0
0
0
0
0
0
0
0
0
0
0
2
0
0
0
0
0
1
0
0
0
2
0


0
0
0
0
0
0
0
0
0
1
0
0
2
0
0
1
0
0
0
0
1
1
0
0
0
0
0
0
0
0
0
0
0
0
0
0
0
0
1
0
0
0
0
0
1
0
0
0
1
0
0
0
0
0
0
0
0
1
0
0
0
1
0
0
0
0
0
0
0
0
1
0
0
0
0
0
0
0
0
0
0


2
6
2
15
11
14
4
6
11
12
20
6
22
1
14
89
2
23
34
6
23
8
9
0
0
4
1
0
0
3
5
24
0
104
11
28
177
0
3
16
5
4
7
3
28
15
6
3
12
0
2
16
6
2
15
16
1
6
16
3
26
1
24
3
3
5
2
3
1
3
2
17
44
9
6
14
18
17
2
282
6


0
0
0
0
0
0
0
0
0
0
0
0
0
0
0
0
0
0
0
0
0
0
0
0
0
0
0
0
0
0
0
0
0
0
0
0
0
0
0
0
0
0
0
0
0
0
0
0
0
0
0
0
0
0
1
0
0
0
0
0
0
0
0
0
0
0
0
0
0
0
0
0
0
0
0
0
0
0
0
0
0


0
0
0
0
0
0
0
0
0
0
0
0
0
0
0
0
0
0
0
0
0
0
0
0
0
0
0
0
0
0
0
0
0
0
0
0
0
0
0
0
0
0
0
0
0
0
0
0
0
0
0
0
0
0
1
0
0
0
0
0
0
0
0
0
0
0
0
0
0
0
0
0
0
0
0
0
0
0
0
0
0


0
1
0
0
0
0
0
0
0
0
0
0
0
0
0
0
0
0
0
0
5
0
1
0
0
0
0
0
0
0
0
0
0
0
0
1
0
0
0
0
0
0
0
0
0
0
0
0
0
0
0
0
0
0
0
0
0
0
0
0
0
0
0
0
0
0
0
0
0
0
0
0
0
0
0
0
0
0
0
0
0


0
1
0
0
0
0
0
0
0
0
0
0
0
0
0
0
0
0
0
0
5
0
1
0
0
0
0
0
0
0
0
0
0
0
0
1
0
0
0
0
0
0
0
0
0
0
0
0
0
0
0
0
0
0
0
0
0
0
0
0
0
0
0
0
0
0
0
0
0
0
0
0
0
0
0
0
0
0
0
0
0


0
0
0
0
0
0
0
0
0
0
0
0
0
0
1
0
0
0
0
0
0
1
0
0
0
0
0
0
0
0
0
0
0
0
0
0
0
0
0
0
0
0
0
0
0
0
0
0
0
0
0
0
0
0
0
0
0
0
0
0
0
0
0
0
0
0
0
0
0
0
0
0
0
0
0
0
0
0
0
0
0


0
0
0
0
0
0
0
0
0
0
0
0
0
0
1
0
0
0
0
0
0
1
0
0
0
0
0
0
0
0
0
0
0
0
0
0
0
0
0
0
0
0
0
0
0
0
0
0
0
0
0
0
0
0
0
0
0
0
0
0
0
0
0
0
0
0
0
0
0
0
0
0
0
0
0
0
0
0
0
0
0


0
0
0
0
0
0
0
0
0
0
0
0
0
0
0
0
0
1
0
0
0
0
0
0
0
0
0
0
0
0
0
0
0
0
0
1
0
0
0
0
0
0
0
0
0
0
0
0
0
0
0
0
0
0
0
0
0
0
0
0
0
0
0
0
0
0
0
0
0
0
0
0
0
0
0
0
0
0
0
0
0


0
0
0
0
0
0
0
0
0
0
0
0
0
0
0
0
0
1
0
0
0
0
0
0
0
0
0
0
0
0
0
0
0
0
0
1
0
0
0
0
0
0
0
0
0
0
0
0
0
0
0
0
0
0
0
0
0
0
0
0
0
0
0
0
0
0
0
0
0
0
0
0
0
0
0
0
0
0
0
0
0


1
2
0
0
0
0
0
0
0
1
0
2
0
0
3
0
0
2
9
0
2
0
1
0
0
0
0
0
0
0
3
0
0
0
0
0
0
0
0
0
1
0
0
0
0
0
0
0
1
0
1
4
0
1
3
2
0
2
2
1
6
2
0
1
2
1
0
0
0
0
0
0
0
0
0
2
0
0
0
0
0


1
2
0
0
0
0
0
0
0
1
0
2
0
0
3
0
0
2
9
0
2
0
1
0
0
0
0
0
0
0
3
0
0
0
0
0
0
0
0
0
1
0
0
0
0
0
0
0
1
0
1
4
0
1
3
2
0
2
2
1
6
2
0
1
2
1
0
0
0
0
0
0
0
0
0
2
0
0
0
0
0


0
0
0
0
0
1
0
0
6
0
0
6
0
2
1
0
1
2
0
0
5
2
2
0
0
0
0
0
0
0
3
0
0
9
7
6
0
2
0
7
1
0
0
0
1
0
0
0
2
0
0
0
2
0
0
0
0
0
0
0
1
1
8
0
0
0
0
1
0
0
0
0
0
0
0
1
0
1
0
0
0


0
0
0
0
0
0
0
0
4
0
0
4
0
2
1
0
1
0
0
0
3
0
1
0
0
0
0
0
0
0
0
0
0
9
0
5
0
0
0
3
1
0
0
0
1
0
0
0
2
0
0
0
0
0
0
0
0
0
0
0
1
1
5
0
0
0
0
0
0
0
0
0
0
0
0
1
0
1
0
0
0


0
0
0
0
0
0
0
0
0
0
0
0
0
0
0
0
0
0
0
0
0
0
1
0
0
0
0
0
0
0
0
0
0
0
0
0
0
0
0
0
0
0
0
0
0
0
0
0
0
0
0
0
0
0
0
0
0
0
0
0
0
0
0
0
0
0
0
0
0
0
0
0
0
0
0
0
0
0
0
0
0


0
0
0
0
0
1
0
0
2
0
0
2
0
0
0
0
0
2
0
0
2
2
0
0
0
0
0
0
0
0
3
0
0
0
7
1
0
2
0
4
0
0
0
0
0
0
0
0
0
0
0
0
2
0
0
0
0
0
0
0
0
0
3
0
0
0
0
1
0
0
0
0
0
0
0
0
0
0
0
0
0


18
16
17
15
19
26
15
17
14
12
26
8
7
9
26
36
14
28
33
13
43
11
13
109
8
27
0
9
339
22
17
86
54
66
13
47
131
6
7
6
27
12
18
15
29
29
13
7
26
1
7
40
9
36
10
21
13
28
19
10
39
8
21
7
18
12
13
9
9
46
15
56
56
18
16
69
41
41
20
39
6


18
16
17
15
19
26
15
17
14
12
26
8
7
9
26
36
14
28
33
13
43
11
13
109
8
27
0
9
339
22
17
86
54
66
13
47
131
6
7
6
27
12
18
15
29
29
13
7
26
1
7
40
9
36
10
21
13
28
19
10
39
8
21
7
18
12
13
9
9
46
15
56
56
18
16
69
41
41
20
39
6


0
0
0
0
0
0
0
1
0
0
0
0
0
0
0
0
0
0
0
1
0
0
0
0
0
0
137
0
0
0
0
0
9
0
0
0
0
0
0
1
0
0
0
0
0
0
0
0
0
0
0
0
0
0
1
0
0
0
0
0
0
0
0
0
0
1
0
0
0
3
0
0
0
0
1
0
0
0
1
0
0


0
0
0
0
0
0
0
0
0
0
0
0
0
0
0
0
0
0
0
0
0
0
0
0
0
0
0
0
0
0
0
0
0
0
0
0
0
0
0
0
0
0
0
0
0
0
0
0
0
0
0
0
0
0
0
0
0
0
0
0
0
0
0
0
0
0
0
0
0
3
0
0
0
0
0
0
0
0
0
0
0


0
0
0
0
0
0
0
0
0
0
0
0
0
0
0
0
0
0
0
0
0
0
0
0
0
0
0
0
0
0
0
0
0
0
0
0
0
0
0
0
0
0
0
0
0
0
0
0
0
0
0
0
0
0
0
0
0
0
0
0
0
0
0
0
0
0
0
0
0
3
0
0
0
0
0
0
0
0
0
0
0


0
0
0
0
0
0
0
1
0
0
0
0
0
0
0
0
0
0
0
1
0
0
0
0
0
0
137
0
0
0
0
0
9
0
0
0
0
0
0
1
0
0
0
0
0
0
0
0
0
0
0
0
0
0
1
0
0
0
0
0
0
0
0
0
0
1
0
0
0
0
0
0
0
0
1
0
0
0
1
0
0


0
0
0
0
0
0
0
1
0
0
0
0
0
0
0
0
0
0
0
1
0
0
0
0
0
0
137
0
0
0
0
0
9
0
0
0
0
0
0
1
0
0
0
0
0
0
0
0
0
0
0
0
0
0
1
0
0
0
0
0
0
0
0
0
0
1
0
0
0
0
0
0
0
0
1
0
0
0
1
0
0


0
0
0
0
0
0
0
0
0
0
0
0
0
0
0
0
0
0
0
0
0
0
0
0
0
0
0
0
0
0
0
0
0
0
0
0
0
0
0
0
0
0
0
0
0
0
0
0
0
0
0
1
0
0
0
0
0
0
0
2
0
0
0
0
0
0
0
0
2
0
0
0
0
0
0
0
0
0
0
0
0


0
0
0
0
0
0
0
0
0
0
0
0
0
0
0
0
0
0
0
0
0
0
0
0
0
0
0
0
0
0
0
0
0
0
0
0
0
0
0
0
0
0
0
0
0
0
0
0
0
0
0
0
0
0
0
0
0
0
0
2
0
0
0
0
0
0
0
0
2
0
0
0
0
0
0
0
0
0
0
0
0


0
0
0
0
0
0
0
0
0
0
0
0
0
0
0
0
0
0
0
0
0
0
0
0
0
0
0
0
0
0
0
0
0
0
0
0
0
0
0
0
0
0
0
0
0
0
0
0
0
0
0
0
0
0
0
0
0
0
0
2
0
0
0
0
0
0
0
0
2
0
0
0
0
0
0
0
0
0
0
0
0


0
0
0
0
0
0
0
0
0
0
0
0
0
0
0
0
0
0
0
0
0
0
0
0
0
0
0
0
0
0
0
0
0
0
0
0
0
0
0
0
0
0
0
0
0
0
0
0
0
0
0
1
0
0
0
0
0
0
0
0
0
0
0
0
0
0
0
0
0
0
0
0
0
0
0
0
0
0
0
0
0


0
0
0
0
0
0
0
0
0
0
0
0
0
0
0
0
0
0
0
0
0
0
0
0
0
0
0
0
0
0
0
0
0
0
0
0
0
0
0
0
0
0
0
0
0
0
0
0
0
0
0
1
0
0
0
0
0
0
0
0
0
0
0
0
0
0
0
0
0
0
0
0
0
0
0
0
0
0
0
0
0


28
102
39
59
44
51
33
37
43
35
97
32
102
51
46
56
32
66
191
84
96
43
52
0
0
88
1
42
0
46
68
269
142
198
22
217
0
9
36
121
82
48
31
63
77
57
39
12
95
0
31
99
61
48
35
65
29
69
75
41
112
28
91
30
58
32
69
45
14
222
68
181
124
38
55
86
109
72
42
35
41


0
0
2
0
1
0
0
0
4
0
2
0
0
6
3
4
0
4
3
2
17
0
0
0
0
4
0
0
0
1
3
0
0
0
0
4
0
1
0
0
0
3
0
0
2
2
2
0
1
0
0
1
0
0
0
0
0
0
0
0
0
0
1
0
0
0
0
0
0
0
1
0
0
0
0
0
0
0
0
2
0


0
0
2
0
1
0
0
0
4
0
2
0
0
6
3
4
0
4
3
2
17
0
0
0
0
4
0
0
0
1
3
0
0
0
0
4
0
1
0
0
0
3
0
0
2
2
2
0
1
0
0
1
0
0
0
0
0
0
0
0
0
0
1
0
0
0
0
0
0
0
1
0
0
0
0
0
0
0
0
2
0


1
0
0
1
0
0
0
2
0
0
0
4
0
2
1
12
1
1
2
4
2
0
2
0
0
0
0
0
0
0
0
0
0
0
0
1
0
0
0
1
2
0
0
2
0
3
1
0
1
0
0
1
0
4
0
2
0
1
0
0
3
1
0
0
6
0
7
6
0
24
4
3
10
1
1
0
1
0
0
4
1


0
0
0
1
0
0
0
0
0
0
0
0
0
1
1
11
1
1
0
2
1
0
2
0
0
0
0
0
0
0
0
0
0
0
0
0
0
0
0
0
2
0
0
0
0
3
0
0
0
0
0
1
0
4
0
0
0
0
0
0
3
1
0
0
4
0
7
6
0
24
4
1
9
1
1
0
1
0
0
0
1


0
0
0
0
0
0
0
0
0
0
0
0
0
0
0
1
0
0
0
0
1
0
0
0
0
0
0
0
0
0
0
0
0
0
0
1
0
0
0
1
0
0
0
0
0
0
1
0
0
0
0
0
0
0
0
0
0
0
0
0
0
0
0
0
0
0
0
0
0
0
0
0
0
0
0
0
0
0
0
1
0


1
0
0
0
0
0
0
2
0
0
0
4
0
1
0
0
0
0
2
2
0
0
0
0
0
0
0
0
0
0
0
0
0
0
0
0
0
0
0
0
0
0
0
2
0
0
0
0
1
0
0
0
0
0
0
2
0
1
0
0
0
0
0
0
2
0
0
0
0
0
0
2
1
0
0
0
0
0
0
3
0


0
3
0
0
0
1
0
0
0
0
1
1
1
1
0
0
0
0
0
0
1
0
0
0
0
3
0
0
0
0
0
0
0
2
0
0
0
0
0
0
0
0
0
0
0
0
0
0
0
0
1
0
0
0
0
0
0
0
0
1
1
0
0
1
0
1
0
2
0
0
0
0
1
0
1
0
0
1
0
0
0


0
1
0
0
0
0
0
0
0
0
0
0
0
0
0
0
0
0
0
0
1
0
0
0
0
3
0
0
0
0
0
0
0
2
0
0
0
0
0
0
0
0
0
0
0
0
0
0
0
0
1
0
0
0
0
0
0
0
0
1
1
0
0
0
0
0
0
2
0
0
0
0
0
0
1
0
0
1
0
0
0


0
0
0
0
0
0
0
0
0
0
0
0
0
1
0
0
0
0
0
0
0
0
0
0
0
0
0
0
0
0
0
0
0
0
0
0
0
0
0
0
0
0
0
0
0
0
0
0
0
0
0
0
0
0
0
0
0
0
0
0
0
0
0
0
0
0
0
0
0
0
0
0
0
0
0
0
0
0
0
0
0


0
2
0
0
0
1
0
0
0
0
1
1
1
0
0
0
0
0
0
0
0
0
0
0
0
0
0
0
0
0
0
0
0
0
0
0
0
0
0
0
0
0
0
0
0
0
0
0
0
0
0
0
0
0
0
0
0
0
0
0
0
0
0
1
0
1
0
0
0
0
0
0
1
0
0
0
0
0
0
0
0


2
4
5
4
1
0
1
1
0
1
0
0
2
1
1
2
2
2
5
2
3
1
10
0
0
0
1
9
0
1
0
0
0
0
0
5
0
0
0
2
1
4
0
0
1
6
4
0
1
0
4
2
0
4
1
0
2
5
1
1
0
0
3
3
1
3
2
1
0
0
3
6
2
1
2
3
0
3
1
0
2


1
3
5
2
0
0
0
1
0
0
0
0
1
0
1
2
0
2
1
1
2
0
4
0
0
0
1
9
0
0
0
0
0
0
0
5
0
0
0
2
1
4
0
0
0
3
2
0
1
0
2
2
0
3
1
0
2
1
0
0
0
0
2
2
1
2
1
0
0
0
2
3
2
1
1
1
0
1
1
0
1


0
0
0
0
0
0
0
0
0
0
0
0
0
0
0
0
0
0
0
0
0
0
0
0
0
0
0
0
0
0
0
0
0
0
0
0
0
0
0
0
0
0
0
0
0
0
2
0
0
0
0
0
0
0
0
0
0
0
0
0
0
0
0
0
0
1
0
0
0
0
0
0
0
0
0
0
0
0
0
0
0


0
0
0
0
0
0
0
0
0
0
0
0
0
0
0
0
0
0
0
0
0
0
1
0
0
0
0
0
0
0
0
0
0
0
0
0
0
0
0
0
0
0
0
0
0
1
0
0
0
0
0
0
0
0
0
0
0
1
0
0
0
0
0
0
0
0
0
0
0
0
1
0
0
0
0
0
0
0
0
0
0


0
0
0
2
0
0
0
0
0
0
0
0
1
0
0
0
0
0
3
0
0
0
4
0
0
0
0
0
0
1
0
0
0
0
0
0
0
0
0
0
0
0
0
0
1
1
0
0
0
0
0
0
0
1
0
0
0
1
0
0
0
0
0
0
0
0
1
0
0
0
0
3
0
0
0
0
0
0
0
0
0


1
1
0
0
1
0
1
0
0
1
0
0
0
1
0
0
2
0
1
1
1
1
1
0
0
0
0
0
0
0
0
0
0
0
0
0
0
0
0
0
0
0
0
0
0
1
0
0
0
0
2
0
0
0
0
0
0
2
1
1
0
0
1
1
0
0
0
1
0
0
0
0
0
0
1
2
0
2
0
0
1


6
5
5
8
5
14
3
5
3
3
6
2
2
5
4
7
2
5
7
6
10
5
1
0
0
7
0
4
0
10
9
59
7
27
5
32
0
0
6
11
10
5
9
9
9
9
1
8
16
0
3
14
16
4
2
5
5
4
11
5
13
1
6
6
5
4
10
3
0
36
8
20
21
5
5
17
21
8
6
4
5


6
5
5
8
5
14
3
5
3
3
6
2
2
5
4
7
2
5
7
6
10
5
1
0
0
7
0
4
0
10
9
59
7
27
5
32
0
0
6
11
10
5
9
9
9
9
1
8
16
0
3
14
16
4
2
5
5
4
11
5
13
1
6
6
5
4
10
3
0
36
8
20
21
5
5
17
21
8
6
4
5


18
87
25
42
31
35
27
27
31
31
82
24
93
28
36
27
24
49
170
62
52
34
34
0
0
71
0
25
0
31
51
173
110
122
17
161
0
8
30
103
66
36
21
48
61
34
30
4
74
0
21
77
42
34
32
50
19
52
62
33
88
24
76
20
40
22
44
32
14
141
51
147
80
30
39
66
82
58
31
22
31


7
64
9
19
14
15
11
6
14
14
35
13
56
11
19
15
10
19
67
37
25
12
19
0
0
11
0
6
0
18
24
57
80
20
0
49
0
2
7
8
21
17
5
24
27
7
7
0
26
0
5
23
17
17
14
18
5
16
20
12
42
10
35
9
17
6
26
15
4
48
21
57
37
7
10
28
34
19
9
11
4


0
0
0
0
0
0
0
0
0
0
0
0
0
0
0
0
0
0
0
0
0
0
0
0
0
0
0
0
0
0
0
0
0
0
0
0
0
0
0
0
0
0
0
0
0
0
0
0
0
0
0
0
0
0
0
1
0
0
0
0
0
0
0
0
0
0
0
0
0
0
1
2
0
0
0
0
0
0
1
0
1


3
4
3
3
7
1
0
1
1
8
15
0
18
2
4
3
0
4
32
1
12
1
1
0
0
8
0
0
0
0
2
0
0
0
4
6
0
0
3
2
2
2
1
2
3
2
2
0
4
0
1
2
5
2
0
5
0
4
1
0
6
0
4
1
8
0
0
2
2
0
5
2
0
3
2
4
1
2
1
2
0


0
0
0
0
0
0
0
0
0
0
0
0
0
0
0
0
0
0
0
0
0
0
0
0
0
2
0
0
0
0
0
0
0
0
0
0
0
0
0
0
0
0
0
0
0
0
0
0
0
0
0
0
0
0
0
0
0
0
0
0
0
0
0
0
0
0
0
0
0
0
0
0
0
0
0
0
0
0
0
0
0


0
0
0
0
0
0
0
0
0
0
0
0
0
0
0
0
0
0
0
0
0
0
0
0
0
0
0
0
0
0
0
0
0
0
0
0
0
0
0
0
0
0
0
0
0
0
0
0
0
0
0
0
0
0
0
0
0
3
0
0
0
0
0
0
0
0
0
0
0
0
0
0
4
0
0
0
0
0
0
0
0


5
5
3
2
4
7
0
8
3
5
9
0
1
4
2
1
3
6
31
6
0
2
3
0
0
0
0
10
0
0
7
0
0
20
0
27
0
0
0
1
1
0
0
1
2
3
3
2
8
0
3
13
4
0
0
1
0
4
7
0
1
2
4
1
1
2
3
2
0
18
3
5
1
1
0
0
0
6
3
0
2


0
3
2
6
3
6
6
2
4
0
8
5
6
4
4
1
5
4
10
9
2
1
2
0
0
24
0
0
0
5
9
1
0
75
4
52
0
3
8
7
25
6
8
15
17
11
4
0
18
0
6
28
9
4
11
15
5
10
22
9
16
5
13
1
2
4
7
2
2
66
7
43
20
15
11
16
27
24
10
3
14


0
0
0
0
0
0
1
0
0
0
0
0
0
0
0
0
0
0
3
0
2
0
2
0
0
0
0
0
0
1
0
0
0
0
0
0
0
0
0
0
1
0
0
0
0
0
1
0
0
0
1
0
0
0
1
0
0
0
1
1
0
0
0
0
0
0
0
3
0
0
1
0
0
0
2
1
1
0
0
0
0


2
0
0
0
1
0
0
0
0
0
0
0
0
0
0
0
0
0
0
0
0
0
0
0
0
0
0
0
0
0
0
0
0
0
0
0
0
0
0
0
0
0
1
0
1
1
0
0
0
0
0
0
0
0
1
0
0
0
0
0
1
0
3
0
0
0
0
0
0
0
0
0
0
0
0
0
0
0
0
0
0


0
0
0
0
0
2
0
0
0
0
1
2
0
0
1
1
1
0
0
1
0
0
2
0
0
3
0
0
0
0
0
0
0
0
3
0
0
0
0
1
0
0
0
2
0
0
1
2
0
0
0
0
0
1
0
0
0
0
1
0
0
0
0
0
0
0
0
0
0
0
0
0
0
0
0
0
0
0
0
0
0


0
0
0
0
0
0
0
0
0
0
0
0
0
0
0
0
0
0
2
0
0
0
0
0
0
0
0
0
0
0
0
0
0
0
0
0
0
0
0
0
0
0
0
0
0
0
0
0
0
0
0
0
0
0
0
0
0
0
0
0
0
0
0
0
0
0
0
0
0
0
0
0
0
0
0
0
0
0
0
0
0


0
0
0
0
0
0
0
0
0
0
0
0
0
0
0
0
0
0
0
0
0
0
0
0
0
10
0
0
0
0
0
0
0
0
0
0
0
0
0
0
0
0
0
0
0
0
0
0
0
0
1
0
0
0
0
0
0
0
0
1
0
0
0
0
0
0
0
0
0
0
0
0
0
0
0
0
0
0
0
0
0


0
0
1
0
0
0
0
0
0
0
0
0
0
0
0
0
0
0
0
0
0
0
0
0
0
0
0
0
0
0
0
0
0
0
0
0
0
0
0
0
0
0
0
0
0
0
0
0
0
0
0
1
0
0
0
1
0
0
0
0
0
0
0
0
0
0
0
0
0
0
0
0
0
0
0
0
0
0
0
0
0


0
0
0
0
0
0
0
0
0
0
0
0
0
0
0
0
0
0
0
0
0
0
0
0
0
1
0
0
0
0
0
0
0
0
0
0
0
0
0
0
0
0
0
0
0
0
0
0
0
0
0
0
0
0
0
0
0
0
0
0
0
0
0
0
0
0
0
0
0
0
0
0
0
0
0
0
0
0
0
0
0


1
11
7
12
2
4
9
10
9
4
14
4
12
7
6
6
5
16
25
8
11
18
5
0
0
12
0
9
0
7
9
115
30
7
6
27
0
3
12
84
16
11
6
4
11
10
12
0
18
0
4
10
7
10
5
9
9
15
10
10
22
7
17
8
12
10
8
8
6
9
13
38
18
4
14
17
19
7
7
6
10


1
3
2
4
6
1
2
2
5
0
6
1
4
8
1
4
3
5
4
8
11
3
5
0
0
3
0
4
0
3
5
37
25
47
0
14
0
0
0
4
3
0
1
4
4
3
1
0
2
0
2
4
3
2
0
8
3
7
1
1
7
2
5
0
6
2
6
1
0
21
1
5
10
1
7
0
5
2
4
3
2


1
3
2
4
6
1
2
2
5
0
6
1
4
8
1
4
3
5
4
8
11
3
5
0
0
3
0
4
0
3
5
37
25
47
0
14
0
0
0
4
3
0
1
4
4
3
1
0
2
0
2
4
3
2
0
8
3
7
1
1
7
2
5
0
6
2
6
1
0
21
1
5
10
1
7
0
5
2
4
3
2


0
0
0
0
1
0
0
0
0
0
0
0
0
0
0
0
1
0
0
0
0
0
0
0
0
0
0
0
0
0
0
0
0
0
0
1
0
0
0
0
0
0
0
0
0
0
0
1
0
0
1
0
0
0
2
0
0
1
0
2
0
0
0
0
0
0
0
1
0
10
0
0
0
0
0
0
0
0
0
0
0


0
0
0
0
0
0
0
0
0
0
0
0
0
0
0
0
0
0
0
0
0
0
0
0
0
0
0
0
0
0
0
0
0
0
0
0
0
0
0
0
0
0
0
0
0
0
0
0
0
0
0
0
0
0
0
0
0
0
0
0
0
0
0
0
0
0
0
0
0
1
0
0
0
0
0
0
0
0
0
0
0


0
0
0
0
0
0
0
0
0
0
0
0
0
0
0
0
0
0
0
0
0
0
0
0
0
0
0
0
0
0
0
0
0
0
0
0
0
0
0
0
0
0
0
0
0
0
0
0
0
0
0
0
0
0
0
0
0
0
0
0
0
0
0
0
0
0
0
0
0
1
0
0
0
0
0
0
0
0
0
0
0


0
0
0
0
1
0
0
0
0
0
0
0
0
0
0
0
0
0
0
0
0
0
0
0
0
0
0
0
0
0
0
0
0
0
0
1
0
0
0
0
0
0
0
0
0
0
0
1
0
0
1
0
0
0
0
0
0
1
0
2
0
0
0
0
0
0
0
1
0
0
0
0
0
0
0
0
0
0
0
0
0


0
0
0
0
1
0
0
0
0
0
0
0
0
0
0
0
0
0
0
0
0
0
0
0
0
0
0
0
0
0
0
0
0
0
0
1
0
0
0
0
0
0
0
0
0
0
0
1
0
0
1
0
0
0
0
0
0
1
0
2
0
0
0
0
0
0
0
1
0
0
0
0
0
0
0
0
0
0
0
0
0


0
0
0
0
0
0
0
0
0
0
0
0
0
0
0
0
1
0
0
0
0
0
0
0
0
0
0
0
0
0
0
0
0
0
0
0
0
0
0
0
0
0
0
0
0
0
0
0
0
0
0
0
0
0
2
0
0
0
0
0
0
0
0
0
0
0
0
0
0
9
0
0
0
0
0
0
0
0
0
0
0


0
0
0
0
0
0
0
0
0
0
0
0
0
0
0
0
1
0
0
0
0
0
0
0
0
0
0
0
0
0
0
0
0
0
0
0
0
0
0
0
0
0
0
0
0
0
0
0
0
0
0
0
0
0
2
0
0
0
0
0
0
0
0
0
0
0
0
0
0
9
0
0
0
0
0
0
0
0
0
0
0


35
8
1
20
27
46
29
41
33
53
40
44
28
17
14
37
9
29
112
14
145
11
50
1
3
39
2
23
0
10
27
0
1
51
0
64
0
0
8
6
17
6
9
14
15
14
8
4
16
0
8
19
10
17
13
17
9
37
17
11
39
24
95
9
29
18
10
13
1
38
17
47
61
45
20
22
54
26
28
152
4


0
0
0
0
0
1
0
0
0
0
0
0
0
0
1
3
0
6
0
0
11
1
0
0
1
0
0
0
0
0
0
0
0
0
0
0
0
0
0
0
0
2
0
0
0
0
0
0
0
0
0
1
0
0
1
1
0
4
0
0
2
3
0
2
4
0
1
0
0
2
0
4
2
2
2
1
0
0
3
62
0


0
0
0
0
0
1
0
0
0
0
0
0
0
0
1
3
0
6
0
0
11
1
0
0
1
0
0
0
0
0
0
0
0
0
0
0
0
0
0
0
0
2
0
0
0
0
0
0
0
0
0
1
0
0
1
1
0
4
0
0
2
3
0
2
4
0
1
0
0
2
0
4
2
2
2
1
0
0
3
62
0


0
0
0
0
0
0
0
0
0
0
0
1
0
0
0
0
0
0
0
0
0
0
0
0
0
0
0
0
0
0
0
0
0
0
0
0
0
0
0
0
0
0
0
0
0
0
0
0
0
0
0
0
0
0
0
0
0
0
0
0
0
0
0
0
0
0
0
0
0
0
0
0
0
0
1
0
0
0
0
0
0


0
0
0
0
0
0
0
0
0
0
0
0
0
0
0
0
0
0
0
0
0
0
0
0
0
0
0
0
0
0
0
0
0
0
0
0
0
0
0
0
0
0
0
0
0
0
0
0
0
0
0
0
0
0
0
0
0
0
0
0
0
0
0
0
0
0
0
0
0
0
0
0
0
0
1
0
0
0
0
0
0


0
0
0
0
0
0
0
0
0
0
0
1
0
0
0
0
0
0
0
0
0
0
0
0
0
0
0
0
0
0
0
0
0
0
0
0
0
0
0
0
0
0
0
0
0
0
0
0
0
0
0
0
0
0
0
0
0
0
0
0
0
0
0
0
0
0
0
0
0
0
0
0
0
0
0
0
0
0
0
0
0


0
0
0
0
0
0
0
0
0
0
0
0
2
0
1
4
0
0
0
0
0
0
0
0
0
0
0
0
0
0
0
0
0
0
0
0
0
0
0
0
0
0
0
0
0
0
0
0
0
0
0
0
0
0
0
0
0
0
1
0
0
0
0
0
0
0
1
0
0
0
0
1
0
0
0
0
0
0
0
0
0


0
0
0
0
0
0
0
0
0
0
0
0
2
0
0
0
0
0
0
0
0
0
0
0
0
0
0
0
0
0
0
0
0
0
0
0
0
0
0
0
0
0
0
0
0
0
0
0
0
0
0
0
0
0
0
0
0
0
1
0
0
0
0
0
0
0
1
0
0
0
0
0
0
0
0
0
0
0
0
0
0


0
0
0
0
0
0
0
0
0
0
0
0
0
0
1
4
0
0
0
0
0
0
0
0
0
0
0
0
0
0
0
0
0
0
0
0
0
0
0
0
0
0
0
0
0
0
0
0
0
0
0
0
0
0
0
0
0
0
0
0
0
0
0
0
0
0
0
0
0
0
0
1
0
0
0
0
0
0
0
0
0


0
0
0
0
0
0
0
0
0
0
0
0
0
0
0
0
0
0
1
0
7
0
0
0
0
0
0
0
0
0
0
0
0
0
0
0
0
0
0
0
1
0
0
0
0
0
0
0
0
0
0
0
0
0
0
0
0
0
0
0
0
0
0
0
0
0
0
0
0
0
0
0
1
0
0
0
0
0
0
0
0


0
0
0
0
0
0
0
0
0
0
0
0
0
0
0
0
0
0
1
0
7
0
0
0
0
0
0
0
0
0
0
0
0
0
0
0
0
0
0
0
1
0
0
0
0
0
0
0
0
0
0
0
0
0
0
0
0
0
0
0
0
0
0
0
0
0
0
0
0
0
0
0
1
0
0
0
0
0
0
0
0


1
0
0
0
0
0
0
0
0
0
0
0
0
0
0
1
0
0
0
0
0
0
0
0
0
0
0
0
0
0
14
0
1
0
0
0
0
0
0
0
0
0
0
0
0
0
0
0
0
0
0
0
0
0
4
0
2
0
2
0
0
0
6
0
0
0
1
0
0
11
0
0
0
0
0
0
0
0
0
0
0


1
0
0
0
0
0
0
0
0
0
0
0
0
0
0
1
0
0
0
0
0
0
0
0
0
0
0
0
0
0
14
0
1
0
0
0
0
0
0
0
0
0
0
0
0
0
0
0
0
0
0
0
0
0
4
0
2
0
2
0
0
0
6
0
0
0
1
0
0
11
0
0
0
0
0
0
0
0
0
0
0


0
0
0
1
0
0
0
0
0
0
0
0
0
0
0
0
0
0
0
0
1
0
0
0
0
0
0
0
0
0
0
0
0
0
0
0
0
0
0
0
0
0
0
0
0
0
0
0
0
0
0
0
0
1
0
1
0
1
0
0
0
0
0
0
0
0
0
2
0
0
0
5
0
0
1
0
0
1
0
32
0


0
0
0
1
0
0
0
0
0
0
0
0
0
0
0
0
0
0
0
0
1
0
0
0
0
0
0
0
0
0
0
0
0
0
0
0
0
0
0
0
0
0
0
0
0
0
0
0
0
0
0
0
0
1
0
1
0
1
0
0
0
0
0
0
0
0
0
2
0
0
0
5
0
0
1
0
0
1
0
32
0


0
0
0
0
0
0
0
0
0
0
1
1
0
0
0
0
0
1
3
0
10
2
8
0
0
12
0
0
0
0
0
0
0
22
0
1
0
0
0
1
1
0
0
3
0
1
0
0
0
0
0
0
1
1
1
0
0
1
2
2
0
0
0
1
0
0
1
0
0
0
1
6
4
1
1
3
5
2
3
0
0


0
0
0
0
0
0
0
0
0
0
1
1
0
0
0
0
0
1
3
0
10
2
8
0
0
12
0
0
0
0
0
0
0
22
0
1
0
0
0
1
1
0
0
3
0
1
0
0
0
0
0
0
1
1
1
0
0
1
2
2
0
0
0
1
0
0
1
0
0
0
1
6
4
1
1
3
5
2
3
0
0


0
0
0
3
4
1
0
0
21
2
1
3
1
6
2
0
0
4
4
6
17
1
1
0
0
0
0
0
0
1
0
0
0
0
0
6
0
0
1
2
6
0
0
0
0
2
1
0
3
0
2
2
1
1
0
0
0
8
1
1
0
0
4
0
0
3
0
0
0
0
1
0
0
0
0
0
0
0
0
0
0


0
0
0
3
2
1
0
0
18
2
1
3
0
5
2
0
0
2
3
6
16
1
1
0
0
0
0
0
0
1
0
0
0
0
0
6
0
0
1
2
6
0
0
0
0
2
1
0
2
0
2
2
0
1
0
0
0
7
1
1
0
0
1
0
0
3
0
0
0
0
1
0
0
0
0
0
0
0
0
0
0


0
0
0
0
0
0
0
0
1
0
0
0
1
0
0
0
0
2
0
0
0
0
0
0
0
0
0
0
0
0
0
0
0
0
0
0
0
0
0
0
0
0
0
0
0
0
0
0
0
0
0
0
0
0
0
0
0
0
0
0
0
0
0
0
0
0
0
0
0
0
0
0
0
0
0
0
0
0
0
0
0


0
0
0
0
0
0
0
0
0
0
0
0
0
0
0
0
0
0
1
0
0
0
0
0
0
0
0
0
0
0
0
0
0
0
0
0
0
0
0
0
0
0
0
0
0
0
0
0
0
0
0
0
0
0
0
0
0
0
0
0
0
0
0
0
0
0
0
0
0
0
0
0
0
0
0
0
0
0
0
0
0


0
0
0
0
2
0
0
0
2
0
0
0
0
1
0
0
0
0
0
0
1
0
0
0
0
0
0
0
0
0
0
0
0
0
0
0
0
0
0
0
0
0
0
0
0
0
0
0
1
0
0
0
1
0
0
0
0
1
0
0
0
0
3
0
0
0
0
0
0
0
0
0
0
0
0
0
0
0
0
0
0


0
0
0
4
1
2
0
0
0
0
5
0
0
0
0
3
0
10
0
0
28
0
0
0
0
0
0
0
0
0
0
0
0
0
0
0
0
0
0
0
0
0
0
0
0
0
0
0
0
0
0
0
0
0
0
0
0
0
0
0
0
0
0
0
0
0
0
1
0
0
0
0
0
0
0
0
0
0
0
0
0


0
0
0
4
1
2
0
0
0
0
5
0
0
0
0
3
0
10
0
0
28
0
0
0
0
0
0
0
0
0
0
0
0
0
0
0
0
0
0
0
0
0
0
0
0
0
0
0
0
0
0
0
0
0
0
0
0
0
0
0
0
0
0
0
0
0
0
1
0
0
0
0
0
0
0
0
0
0
0
0
0


34
8
1
12
22
42
29
41
12
51
33
39
25
11
10
26
9
8
104
8
71
7
41
1
2
27
2
23
0
9
13
0
0
29
0
57
0
0
7
3
9
4
9
11
15
11
7
4
13
0
6
16
8
14
7
15
7
23
11
8
37
21
85
6
25
15
6
10
1
25
15
31
54
42
15
18
49
23
22
58
4


34
8
1
12
22
42
29
41
12
51
33
39
25
11
10
26
9
8
104
8
71
7
41
1
2
27
2
23
0
9
13
0
0
29
0
57
0
0
7
3
9
4
9
11
15
11
7
4
13
0
6
16
8
14
7
15
7
23
11
8
37
21
85
6
25
15
6
10
1
25
15
31
54
42
15
18
49
23
22
58
4


0
0
0
0
0
0
0
0
0
0
0
0
1
1
0
0
0
0
0
0
1
0
0
0
0
0
0
0
0
0
0
0
0
0
0
0
0
0
0
0
0
0
0
0
0
0
0
0
0
0
0
0
0
0
0
0
0
0
0
0
0
1
0
0
2
0
0
0
0
0
0
0
4
0
0
0
0
0
0
0
0


0
0
0
0
0
0
0
0
0
0
0
0
0
0
0
0
0
0
0
0
1
0
0
0
0
0
0
0
0
0
0
0
0
0
0
0
0
0
0
0
0
0
0
0
0
0
0
0
0
0
0
0
0
0
0
0
0
0
0
0
0
0
0
0
0
0
0
0
0
0
0
0
0
0
0
0
0
0
0
0
0


0
0
0
0
0
0
0
0
0
0
0
0
0
0
0
0
0
0
0
0
1
0
0
0
0
0
0
0
0
0
0
0
0
0
0
0
0
0
0
0
0
0
0
0
0
0
0
0
0
0
0
0
0
0
0
0
0
0
0
0
0
0
0
0
0
0
0
0
0
0
0
0
0
0
0
0
0
0
0
0
0


0
0
0
0
0
0
0
0
0
0
0
0
1
1
0
0
0
0
0
0
0
0
0
0
0
0
0
0
0
0
0
0
0
0
0
0
0
0
0
0
0
0
0
0
0
0
0
0
0
0
0
0
0
0
0
0
0
0
0
0
0
1
0
0
2
0
0
0
0
0
0
0
4
0
0
0
0
0
0
0
0


0
0
0
0
0
0
0
0
0
0
0
0
1
1
0
0
0
0
0
0
0
0
0
0
0
0
0
0
0
0
0
0
0
0
0
0
0
0
0
0
0
0
0
0
0
0
0
0
0
0
0
0
0
0
0
0
0
0
0
0
0
1
0
0
2
0
0
0
0
0
0
0
4
0
0
0
0
0
0
0
0


0
4
2
2
1
3
3
5
0
0
0
0
0
0
0
2
0
0
0
4
9
0
0
1719
1405
58
1663
57
1501
3
2
0
93
4
13
1
119
0
1
4
1
3
2
9
6
8
3
89
0
0
2
1
0
2
0
0
0
0
0
2
1
0
1
1
5
0
1
1
2
54
0
0
0
0
0
0
0
0
0
0
0


0
0
0
0
0
0
0
0
0
0
0
0
0
0
0
0
0
0
0
0
0
0
0
0
0
0
0
0
0
0
0
0
0
0
0
0
0
0
0
0
0
0
0
0
0
0
0
0
0
0
0
0
0
0
0
0
0
0
0
2
0
0
0
0
0
0
0
0
0
0
0
0
0
0
0
0
0
0
0
0
0


0
0
0
0
0
0
0
0
0
0
0
0
0
0
0
0
0
0
0
0
0
0
0
0
0
0
0
0
0
0
0
0
0
0
0
0
0
0
0
0
0
0
0
0
0
0
0
0
0
0
0
0
0
0
0
0
0
0
0
2
0
0
0
0
0
0
0
0
0
0
0
0
0
0
0
0
0
0
0
0
0


0
4
2
2
1
3
3
5
0
0
0
0
0
0
0
1
0
0
0
4
9
0
0
1717
1401
58
1661
57
1499
3
2
0
93
4
13
1
119
0
1
4
1
3
2
9
6
8
3
88
0
0
2
1
0
2
0
0
0
0
0
0
1
0
1
1
5
0
1
1
0
54
0
0
0
0
0
0
0
0
0
0
0


0
0
0
0
0
0
0
0
0
0
0
0
0
0
0
0
0
0
0
0
0
0
0
7
1
1
0
0
1
0
0
0
0
0
0
0
0
0
0
0
0
0
0
0
0
0
0
0
0
0
0
0
0
0
0
0
0
0
0
0
0
0
0
0
0
0
0
0
0
0
0
0
0
0
0
0
0
0
0
0
0


0
0
0
0
0
0
1
0
0
0
0
0
0
0
0
0
0
0
0
0
0
0
0
0
0
0
0
0
165
0
0
0
0
0
0
0
0
0
0
0
0
0
0
0
0
0
0
10
0
0
0
0
0
0
0
0
0
0
0
0
0
0
0
0
0
0
0
0
0
0
0
0
0
0
0
0
0
0
0
0
0


0
0
0
0
0
0
0
0
0
0
0
0
0
0
0
0
0
0
0
0
0
0
0
0
0
0
1
0
0
0
0
0
0
0
0
0
0
0
0
0
0
0
0
0
0
0
0
0
0
0
0
0
0
0
0
0
0
0
0
0
0
0
0
0
0
0
0
0
0
0
0
0
0
0
0
0
0
0
0
0
0


0
0
1
0
0
1
0
3
0
0
0
0
0
0
0
0
0
0
0
0
9
0
0
218
356
9
610
10
145
0
0
0
0
0
0
0
0
0
1
0
1
0
0
0
2
0
0
12
0
0
0
0
0
0
0
0
0
0
0
0
0
0
0
1
0
0
0
0
0
2
0
0
0
0
0
0
0
0
0
0
0


0
4
1
2
1
2
2
2
0
0
0
0
0
0
0
1
0
0
0
4
0
0
0
1492
1044
48
1050
47
1188
3
2
0
93
4
13
1
119
0
0
4
0
3
2
9
4
8
3
66
0
0
2
1
0
2
0
0
0
0
0
0
1
0
1
0
5
0
1
1
0
52
0
0
0
0
0
0
0
0
0
0
0


0
0
0
0
0
0
0
0
0
0
0
0
0
0
0
1
0
0
0
0
0
0
0
2
4
0
2
0
2
0
0
0
0
0
0
0
0
0
0
0
0
0
0
0
0
0
0
1
0
0
0
0
0
0
0
0
0
0
0
0
0
0
0
0
0
0
0
0
2
0
0
0
0
0
0
0
0
0
0
0
0


0
0
0
0
0
0
0
0
0
0
0
0
0
0
0
1
0
0
0
0
0
0
0
2
4
0
2
0
2
0
0
0
0
0
0
0
0
0
0
0
0
0
0
0
0
0
0
1
0
0
0
0
0
0
0
0
0
0
0
0
0
0
0
0
0
0
0
0
2
0
0
0
0
0
0
0
0
0
0
0
0


11
7
2
7
8
16
4
10
9
7
16
5
2
6
3
7
4
20
12
9
73
4
8
0
0
18
0
5
0
3
6
0
17
10
0
9
0
0
0
1
2
6
3
5
10
14
4
2
4
0
1
0
5
1
2
0
1
0
0
0
2
0
1
2
0
1
1
2
1
6
0
2
2
1
0
2
0
0
0
0
0


6
3
2
3
3
13
2
5
9
5
10
3
0
3
2
5
3
6
3
7
52
2
1
0
0
0
0
0
0
3
2
0
17
0
0
6
0
0
0
0
0
5
1
0
10
12
2
0
2
0
1
0
2
1
1
0
0
0
0
0
1
0
1
0
0
0
1
2
1
6
0
0
0
1
0
0
0
0
0
0
0


6
3
2
3
3
13
2
5
9
5
10
3
0
3
2
5
3
6
3
7
52
2
1
0
0
0
0
0
0
3
2
0
17
0
0
6
0
0
0
0
0
5
1
0
10
12
2
0
2
0
1
0
2
1
1
0
0
0
0
0
1
0
1
0
0
0
1
2
1
6
0
0
0
1
0
0
0
0
0
0
0


1
0
0
1
0
0
0
1
0
0
1
0
0
0
0
0
0
0
0
0
0
0
2
0
0
0
0
0
0
0
0
0
0
0
0
0
0
0
0
0
0
0
0
0
0
1
0
0
0
0
0
0
0
0
0
0
0
0
0
0
0
0
0
0
0
1
0
0
0
0
0
0
0
0
0
0
0
0
0
0
0


1
0
0
1
0
0
0
1
0
0
1
0
0
0
0
0
0
0
0
0
0
0
2
0
0
0
0
0
0
0
0
0
0
0
0
0
0
0
0
0
0
0
0
0
0
1
0
0
0
0
0
0
0
0
0
0
0
0
0
0
0
0
0
0
0
1
0
0
0
0
0
0
0
0
0
0
0
0
0
0
0


0
0
0
0
2
1
0
0
0
1
0
1
0
1
0
0
0
3
2
1
11
0
1
0
0
3
0
5
0
0
0
0
0
0
0
0
0
0
0
0
0
0
0
0
0
0
0
0
0
0
0
0
0
0
0
0
0
0
0
0
0
0
0
0
0
0
0
0
0
0
0
0
0
0
0
0
0
0
0
0
0


0
0
0
0
2
1
0
0
0
1
0
1
0
1
0
0
0
3
2
1
11
0
1
0
0
3
0
5
0
0
0
0
0
0
0
0
0
0
0
0
0
0
0
0
0
0
0
0
0
0
0
0
0
0
0
0
0
0
0
0
0
0
0
0
0
0
0
0
0
0
0
0
0
0
0
0
0
0
0
0
0


0
1
0
2
1
0
1
2
0
0
0
1
1
0
1
0
0
2
6
1
3
2
1
0
0
0
0
0
0
0
3
0
0
10
0
1
0
0
0
0
2
1
2
5
0
0
2
2
2
0
0
0
1
0
0
0
1
0
0
0
1
0
0
2
0
0
0
0
0
0
0
1
2
0
0
2
0
0
0
0
0


0
1
0
2
1
0
1
2
0
0
0
1
1
0
1
0
0
2
6
1
3
2
1
0
0
0
0
0
0
0
3
0
0
10
0
1
0
0
0
0
2
1
2
5
0
0
2
2
2
0
0
0
1
0
0
0
1
0
0
0
1
0
0
2
0
0
0
0
0
0
0
1
2
0
0
2
0
0
0
0
0


0
0
0
0
0
0
0
0
0
0
0
0
0
0
0
1
0
6
0
0
0
0
3
0
0
15
0
0
0
0
0
0
0
0
0
0
0
0
0
1
0
0
0
0
0
0
0
0
0
0
0
0
0
0
1
0
0
0
0
0
0
0
0
0
0
0
0
0
0
0
0
0
0
0
0
0
0
0
0
0
0


0
0
0
0
0
0
0
0
0
0
0
0
0
0
0
1
0
6
0
0
0
0
3
0
0
15
0
0
0
0
0
0
0
0
0
0
0
0
0
1
0
0
0
0
0
0
0
0
0
0
0
0
0
0
1
0
0
0
0
0
0
0
0
0
0
0
0
0
0
0
0
0
0
0
0
0
0
0
0
0
0


4
3
0
1
2
2
1
2
0
1
5
0
1
2
0
1
1
3
1
0
7
0
0
0
0
0
0
0
0
0
1
0
0
0
0
2
0
0
0
0
0
0
0
0
0
1
0
0
0
0
0
0
2
0
0
0
0
0
0
0
0
0
0
0
0
0
0
0
0
0
0
1
0
0
0
0
0
0
0
0
0


4
3
0
1
2
2
1
2
0
1
5
0
1
2
0
1
1
3
1
0
7
0
0
0
0
0
0
0
0
0
1
0
0
0
0
2
0
0
0
0
0
0
0
0
0
1
0
0
0
0
0
0
2
0
0
0
0
0
0
0
0
0
0
0
0
0
0
0
0
0
0
1
0
0
0
0
0
0
0
0
0


25
38
16
55
82
73
73
36
69
205
274
108
118
33
208
858
58
438
1094
139
229
37
39
598
172
55
525
41
657
39
61
77
52
131
39
151
6
9
9
72
13
43
32
45
121
127
49
29
62
0
38
86
22
49
39
75
39
118
46
43
123
59
138
25
72
47
44
58
11
71
47
148
132
126
30
91
90
83
50
817
28


25
38
16
55
82
73
73
36
69
205
274
108
118
33
208
858
58
438
1094
139
229
37
39
598
172
55
525
41
657
39
61
77
52
131
39
151
6
9
9
72
13
43
32
45
121
127
49
29
62
0
38
86
22
49
39
75
39
118
46
43
123
59
138
25
72
47
44
58
11
71
47
148
132
126
30
91
90
83
50
817
28


25
38
16
55
82
73
73
36
69
205
274
108
118
33
208
858
58
438
1094
139
229
37
39
598
172
55
525
41
657
39
61
77
52
131
39
151
6
9
9
72
13
43
32
45
121
127
49
29
62
0
38
86
22
49
39
75
39
118
46
43
123
59
138
25
72
47
44
58
11
71
47
148
132
126
30
91
90
83
50
817
28


0
0
0
0
3
0
1
1
1
0
0
0
0
0
1
0
0
0
0
0
0
1
0
0
89
4
63
1
1
6
2
0
0
0
4
0
162
1
0
0
0
1
0
0
0
0
0
1
0
0
1
0
0
0
0
0
0
0
1
0
0
0
1
0
0
0
0
1
0
8
1
0
1
0
0
0
1
0
0
0
0


0
0
0
0
0
0
0
0
0
0
0
0
0
0
0
0
0
0
0
0
0
0
0
0
0
0
0
0
1
0
0
0
0
0
0
0
0
0
0
0
0
0
0
0
0
0
0
1
0
0
0
0
0
0
0
0
0
0
0
0
0
0
0
0
0
0
0
0
0
1
0
0
0
0
0
0
0
0
0
0
0


0
0
0
0
0
0
0
0
0
0
0
0
0
0
0
0
0
0
0
0
0
0
0
0
0
0
0
0
1
0
0
0
0
0
0
0
0
0
0
0
0
0
0
0
0
0
0
1
0
0
0
0
0
0
0
0
0
0
0
0
0
0
0
0
0
0
0
0
0
1
0
0
0
0
0
0
0
0
0
0
0


0
0
0
0
0
0
0
0
0
0
0
0
0
0
0
0
0
0
0
0
0
0
0
0
0
0
0
0
1
0
0
0
0
0
0
0
0
0
0
0
0
0
0
0
0
0
0
1
0
0
0
0
0
0
0
0
0
0
0
0
0
0
0
0
0
0
0
0
0
1
0
0
0
0
0
0
0
0
0
0
0


0
0
0
0
3
0
1
1
1
0
0
0
0
0
1
0
0
0
0
0
0
1
0
0
89
4
63
1
0
6
2
0
0
0
4
0
162
1
0
0
0
1
0
0
0
0
0
0
0
0
1
0
0
0
0
0
0
0
1
0
0
0
1
0
0
0
0
1
0
7
1
0
1
0
0
0
1
0
0
0
0


0
0
0
0
3
0
1
1
1
0
0
0
0
0
1
0
0
0
0
0
0
1
0
0
89
3
63
1
0
5
2
0
0
0
3
0
162
1
0
0
0
1
0
0
0
0
0
0
0
0
1
0
0
0
0
0
0
0
1
0
0
0
1
0
0
0
0
1
0
7
1
0
1
0
0
0
1
0
0
0
0


0
0
0
0
3
0
1
1
1
0
0
0
0
0
1
0
0
0
0
0
0
1
0
0
89
3
63
1
0
5
2
0
0
0
3
0
162
1
0
0
0
1
0
0
0
0
0
0
0
0
1
0
0
0
0
0
0
0
1
0
0
0
1
0
0
0
0
1
0
7
1
0
1
0
0
0
1
0
0
0
0


0
0
0
0
0
0
0
0
0
0
0
0
0
0
0
0
0
0
0
0
0
0
0
0
0
1
0
0
0
1
0
0
0
0
1
0
0
0
0
0
0
0
0
0
0
0
0
0
0
0
0
0
0
0
0
0
0
0
0
0
0
0
0
0
0
0
0
0
0
0
0
0
0
0
0
0
0
0
0
0
0


0
0
0
0
0
0
0
0
0
0
0
0
0
0
0
0
0
0
0
0
0
0
0
0
0
1
0
0
0
1
0
0
0
0
1
0
0
0
0
0
0
0
0
0
0
0
0
0
0
0
0
0
0
0
0
0
0
0
0
0
0
0
0
0
0
0
0
0
0
0
0
0
0
0
0
0
0
0
0
0
0


0
0
0
0
1
0
0
0
0
0
0
0
1
1
0
0
2
1
0
0
5
1
1
0
0
0
0
0
0
0
0
0
0
0
0
0
0
0
0
0
0
0
0
0
0
0
0
0
0
0
0
0
0
0
0
0
0
0
0
0
0
0
0
0
0
0
0
0
0
0
0
0
0
0
0
0
0
0
0
0
0


0
0
0
0
1
0
0
0
0
0
0
0
1
1
0
0
2
1
0
0
5
1
1
0
0
0
0
0
0
0
0
0
0
0
0
0
0
0
0
0
0
0
0
0
0
0
0
0
0
0
0
0
0
0
0
0
0
0
0
0
0
0
0
0
0
0
0
0
0
0
0
0
0
0
0
0
0
0
0
0
0


0
0
0
0
1
0
0
0
0
0
0
0
1
1
0
0
2
1
0
0
5
1
1
0
0
0
0
0
0
0
0
0
0
0
0
0
0
0
0
0
0
0
0
0
0
0
0
0
0
0
0
0
0
0
0
0
0
0
0
0
0
0
0
0
0
0
0
0
0
0
0
0
0
0
0
0
0
0
0
0
0


0
0
0
0
1
0
0
0
0
0
0
0
1
1
0
0
2
1
0
0
5
1
1
0
0
0
0
0
0
0
0
0
0
0
0
0
0
0
0
0
0
0
0
0
0
0
0
0
0
0
0
0
0
0
0
0
0
0
0
0
0
0
0
0
0
0
0
0
0
0
0
0
0
0
0
0
0
0
0
0
0


0
4
2
1
4
3
2
3
2
1
0
2
0
1
2
3
1
0
2
3
2
3
2
653
499
31
1278
39
1450
13
6
64
137
28
105
15
2974
10
5
4
4
5
10
17
7
5
2
88
2
401
4
7
1
2
4
1
2
2
3
3
2
5
0
2
1
1
1
2
3
43
0
3
7
2
0
1
0
7
6
3
2


0
0
0
0
0
0
0
0
0
0
0
0
0
0
0
0
0
0
0
0
0
0
0
0
12
0
0
0
0
0
0
11
14
0
0
0
0
0
0
0
0
0
0
0
0
0
0
0
0
19
0
0
0
0
0
0
0
0
0
0
0
0
0
0
0
0
0
0
0
0
0
0
0
0
0
0
0
0
0
0
0


0
0
0
0
0
0
0
0
0
0
0
0
0
0
0
0
0
0
0
0
0
0
0
0
12
0
0
0
0
0
0
0
14
0
0
0
0
0
0
0
0
0
0
0
0
0
0
0
0
0
0
0
0
0
0
0
0
0
0
0
0
0
0
0
0
0
0
0
0
0
0
0
0
0
0
0
0
0
0
0
0


0
0
0
0
0
0
0
0
0
0
0
0
0
0
0
0
0
0
0
0
0
0
0
0
12
0
0
0
0
0
0
0
14
0
0
0
0
0
0
0
0
0
0
0
0
0
0
0
0
0
0
0
0
0
0
0
0
0
0
0
0
0
0
0
0
0
0
0
0
0
0
0
0
0
0
0
0
0
0
0
0


0
0
0
0
0
0
0
0
0
0
0
0
0
0
0
0
0
0
0
0
0
0
0
0
0
0
0
0
0
0
0
11
0
0
0
0
0
0
0
0
0
0
0
0
0
0
0
0
0
19
0
0
0
0
0
0
0
0
0
0
0
0
0
0
0
0
0
0
0
0
0
0
0
0
0
0
0
0
0
0
0


0
0
0
0
0
0
0
0
0
0
0
0
0
0
0
0
0
0
0
0
0
0
0
0
0
0
0
0
0
0
0
11
0
0
0
0
0
0
0
0
0
0
0
0
0
0
0
0
0
0
0
0
0
0
0
0
0
0
0
0
0
0
0
0
0
0
0
0
0
0
0
0
0
0
0
0
0
0
0
0
0


0
0
0
0
0
0
0
0
0
0
0
0
0
0
0
0
0
0
0
0
0
0
0
0
0
0
0
0
0
0
0
0
0
0
0
0
0
0
0
0
0
0
0
0
0
0
0
0
0
19
0
0
0
0
0
0
0
0
0
0
0
0
0
0
0
0
0
0
0
0
0
0
0
0
0
0
0
0
0
0
0


0
0
0
0
0
0
0
0
0
0
0
0
0
0
0
0
0
0
0
0
0
0
1
73
0
0
40
0
0
0
0
0
0
0
2
1
42
0
0
0
0
0
0
0
0
0
1
1
0
0
0
0
0
0
0
0
0
0
0
0
0
0
0
1
1
0
0
0
0
0
0
0
0
0
0
0
0
0
0
0
0


0
0
0
0
0
0
0
0
0
0
0
0
0
0
0
0
0
0
0
0
0
0
0
0
0
0
40
0
0
0
0
0
0
0
0
0
0
0
0
0
0
0
0
0
0
0
0
1
0
0
0
0
0
0
0
0
0
0
0
0
0
0
0
0
0
0
0
0
0
0
0
0
0
0
0
0
0
0
0
0
0


0
0
0
0
0
0
0
0
0
0
0
0
0
0
0
0
0
0
0
0
0
0
0
0
0
0
40
0
0
0
0
0
0
0
0
0
0
0
0
0
0
0
0
0
0
0
0
1
0
0
0
0
0
0
0
0
0
0
0
0
0
0
0
0
0
0
0
0
0
0
0
0
0
0
0
0
0
0
0
0
0


0
0
0
0
0
0
0
0
0
0
0
0
0
0
0
0
0
0
0
0
0
0
1
73
0
0
0
0
0
0
0
0
0
0
2
1
42
0
0
0
0
0
0
0
0
0
0
0
0
0
0
0
0
0
0
0
0
0
0
0
0
0
0
1
1
0
0
0
0
0
0
0
0
0
0
0
0
0
0
0
0


0
0
0
0
0
0
0
0
0
0
0
0
0
0
0
0
0
0
0
0
0
0
1
73
0
0
0
0
0
0
0
0
0
0
2
1
42
0
0
0
0
0
0
0
0
0
0
0
0
0
0
0
0
0
0
0
0
0
0
0
0
0
0
1
1
0
0
0
0
0
0
0
0
0
0
0
0
0
0
0
0


0
0
0
0
0
0
0
0
0
0
0
0
0
0
0
0
0
0
0
0
0
0
0
0
0
0
0
0
0
0
0
0
0
0
0
0
0
0
0
0
0
0
0
0
0
0
1
0
0
0
0
0
0
0
0
0
0
0
0
0
0
0
0
0
0
0
0
0
0
0
0
0
0
0
0
0
0
0
0
0
0


0
0
0
0
0
0
0
0
0
0
0
0
0
0
0
0
0
0
0
0
0
0
0
0
0
0
0
0
0
0
0
0
0
0
0
0
0
0
0
0
0
0
0
0
0
0
1
0
0
0
0
0
0
0
0
0
0
0
0
0
0
0
0
0
0
0
0
0
0
0
0
0
0
0
0
0
0
0
0
0
0


0
0
0
0
1
0
0
0
0
0
0
0
0
0
0
3
0
0
0
0
1
0
0
11
1
0
0
0
0
0
1
0
0
0
0
0
0
0
0
0
1
1
0
0
0
0
0
0
0
0
0
0
0
0
0
0
0
0
0
3
0
1
0
0
0
0
0
0
0
13
0
0
0
0
0
0
0
1
1
2
0


0
0
0
0
0
0
0
0
0
0
0
0
0
0
0
3
0
0
0
0
1
0
0
11
0
0
0
0
0
0
1
0
0
0
0
0
0
0
0
0
1
1
0
0
0
0
0
0
0
0
0
0
0
0
0
0
0
0
0
3
0
1
0
0
0
0
0
0
0
13
0
0
0
0
0
0
0
1
0
2
0


0
0
0
0
0
0
0
0
0
0
0
0
0
0
0
0
0
0
0
0
0
0
0
0
0
0
0
0
0
0
1
0
0
0
0
0
0
0
0
0
0
0
0
0
0
0
0
0
0
0
0
0
0
0
0
0
0
0
0
0
0
0
0
0
0
0
0
0
0
3
0
0
0
0
0
0
0
0
0
0
0


0
0
0
0
0
0
0
0
0
0
0
0
0
0
0
0
0
0
0
0
0
0
0
0
0
0
0
0
0
0
0
0
0
0
0
0
0
0
0
0
0
0
0
0
0
0
0
0
0
0
0
0
0
0
0
0
0
0
0
0
0
0
0
0
0
0
0
0
0
10
0
0
0
0
0
0
0
0
0
0
0


0
0
0
0
0
0
0
0
0
0
0
0
0
0
0
3
0
0
0
0
1
0
0
11
0
0
0
0
0
0
0
0
0
0
0
0
0
0
0
0
1
1
0
0
0
0
0
0
0
0
0
0
0
0
0
0
0
0
0
3
0
1
0
0
0
0
0
0
0
0
0
0
0
0
0
0
0
1
0
2
0


0
0
0
0
1
0
0
0
0
0
0
0
0
0
0
0
0
0
0
0
0
0
0
0
0
0
0
0
0
0
0
0
0
0
0
0
0
0
0
0
0
0
0
0
0
0
0
0
0
0
0
0
0
0
0
0
0
0
0
0
0
0
0
0
0
0
0
0
0
0
0
0
0
0
0
0
0
0
1
0
0


0
0
0
0
1
0
0
0
0
0
0
0
0
0
0
0
0
0
0
0
0
0
0
0
0
0
0
0
0
0
0
0
0
0
0
0
0
0
0
0
0
0
0
0
0
0
0
0
0
0
0
0
0
0
0
0
0
0
0
0
0
0
0
0
0
0
0
0
0
0
0
0
0
0
0
0
0
0
1
0
0


0
0
0
0
0
0
0
0
0
0
0
0
0
0
0
0
0
0
0
0
0
0
0
0
1
0
0
0
0
0
0
0
0
0
0
0
0
0
0
0
0
0
0
0
0
0
0
0
0
0
0
0
0
0
0
0
0
0
0
0
0
0
0
0
0
0
0
0
0
0
0
0
0
0
0
0
0
0
0
0
0


0
0
0
0
0
0
0
0
0
0
0
0
0
0
0
0
0
0
0
0
0
0
0
0
1
0
0
0
0
0
0
0
0
0
0
0
0
0
0
0
0
0
0
0
0
0
0
0
0
0
0
0
0
0
0
0
0
0
0
0
0
0
0
0
0
0
0
0
0
0
0
0
0
0
0
0
0
0
0
0
0


0
1
1
0
0
1
0
0
1
0
0
0
0
0
0
0
0
0
0
0
0
0
0
29
26
1
58
0
0
0
0
1
1
1
0
0
0
0
0
0
2
0
0
2
0
0
0
1
0
0
1
0
0
0
1
0
0
0
0
0
0
1
0
0
0
0
1
1
1
8
0
1
1
0
0
0
0
0
0
0
0


0
1
1
0
0
1
0
0
1
0
0
0
0
0
0
0
0
0
0
0
0
0
0
29
26
1
58
0
0
0
0
1
1
1
0
0
0
0
0
0
0
0
0
2
0
0
0
0
0
0
1
0
0
0
1
0
0
0
0
0
0
1
0
0
0
0
1
1
1
8
0
1
1
0
0
0
0
0
0
0
0


0
0
1
0
0
1
0
0
1
0
0
0
0
0
0
0
0
0
0
0
0
0
0
0
0
1
0
0
0
0
0
1
1
0
0
0
0
0
0
0
0
0
0
0
0
0
0
0
0
0
1
0
0
0
0
0
0
0
0
0
0
1
0
0
0
0
1
1
0
0
0
1
1
0
0
0
0
0
0
0
0


0
0
0
0
0
0
0
0
0
0
0
0
0
0
0
0
0
0
0
0
0
0
0
0
0
0
0
0
0
0
0
0
0
1
0
0
0
0
0
0
0
0
0
0
0
0
0
0
0
0
0
0
0
0
0
0
0
0
0
0
0
0
0
0
0
0
0
0
0
0
0
0
0
0
0
0
0
0
0
0
0


0
0
0
0
0
0
0
0
0
0
0
0
0
0
0
0
0
0
0
0
0
0
0
0
0
0
0
0
0
0
0
0
0
0
0
0
0
0
0
0
0
0
0
0
0
0
0
0
0
0
0
0
0
0
1
0
0
0
0
0
0
0
0
0
0
0
0
0
0
0
0
0
0
0
0
0
0
0
0
0
0


0
1
0
0
0
0
0
0
0
0
0
0
0
0
0
0
0
0
0
0
0
0
0
29
26
0
58
0
0
0
0
0
0
0
0
0
0
0
0
0
0
0
0
2
0
0
0
0
0
0
0
0
0
0
0
0
0
0
0
0
0
0
0
0
0
0
0
0
1
8
0
0
0
0
0
0
0
0
0
0
0


0
0
0
0
0
0
0
0
0
0
0
0
0
0
0
0
0
0
0
0
0
0
0
0
0
0
0
0
0
0
0
0
0
0
0
0
0
0
0
0
2
0
0
0
0
0
0
1
0
0
0
0
0
0
0
0
0
0
0
0
0
0
0
0
0
0
0
0
0
0
0
0
0
0
0
0
0
0
0
0
0


0
0
0
0
0
0
0
0
0
0
0
0
0
0
0
0
0
0
0
0
0
0
0
0
0
0
0
0
0
0
0
0
0
0
0
0
0
0
0
0
2
0
0
0
0
0
0
1
0
0
0
0
0
0
0
0
0
0
0
0
0
0
0
0
0
0
0
0
0
0
0
0
0
0
0
0
0
0
0
0
0


0
0
0
0
1
0
0
0
0
0
0
1
0
1
0
0
0
0
0
0
1
0
0
0
0
0
1
0
0
0
0
0
0
0
0
0
0
0
2
0
0
0
0
1
0
0
0
0
0
0
0
0
0
0
1
0
0
0
0
0
1
1
0
0
0
0
0
0
0
0
0
0
1
0
0
0
0
1
0
0
0


0
0
0
0
1
0
0
0
0
0
0
1
0
0
0
0
0
0
0
0
0
0
0
0
0
0
0
0
0
0
0
0
0
0
0
0
0
0
1
0
0
0
0
0
0
0
0
0
0
0
0
0
0
0
0
0
0
0
0
0
1
0
0
0
0
0
0
0
0
0
0
0
0
0
0
0
0
1
0
0
0


0
0
0
0
0
0
0
0
0
0
0
1
0
0
0
0
0
0
0
0
0
0
0
0
0
0
0
0
0
0
0
0
0
0
0
0
0
0
1
0
0
0
0
0
0
0
0
0
0
0
0
0
0
0
0
0
0
0
0
0
1
0
0
0
0
0
0
0
0
0
0
0
0
0
0
0
0
0
0
0
0


0
0
0
0
1
0
0
0
0
0
0
0
0
0
0
0
0
0
0
0
0
0
0
0
0
0
0
0
0
0
0
0
0
0
0
0
0
0
0
0
0
0
0
0
0
0
0
0
0
0
0
0
0
0
0
0
0
0
0
0
0
0
0
0
0
0
0
0
0
0
0
0
0
0
0
0
0
1
0
0
0


0
0
0
0
0
0
0
0
0
0
0
0
0
0
0
0
0
0
0
0
0
0
0
0
0
0
0
0
0
0
0
0
0
0
0
0
[truncated: 441,397 more chars]
