# Supplementary figures and images for: Prokaryotic Diversity in the Rhizosphere of Organic, Intensive, and Transitional Coffee Farms in Brazil
Source: PLoS One. 2015 Jun 17;10(6):e0106355. doi: 10.1371/journal.pone.0106355 (PMC4471275; doi:10.1371/journal.pone.0106355)

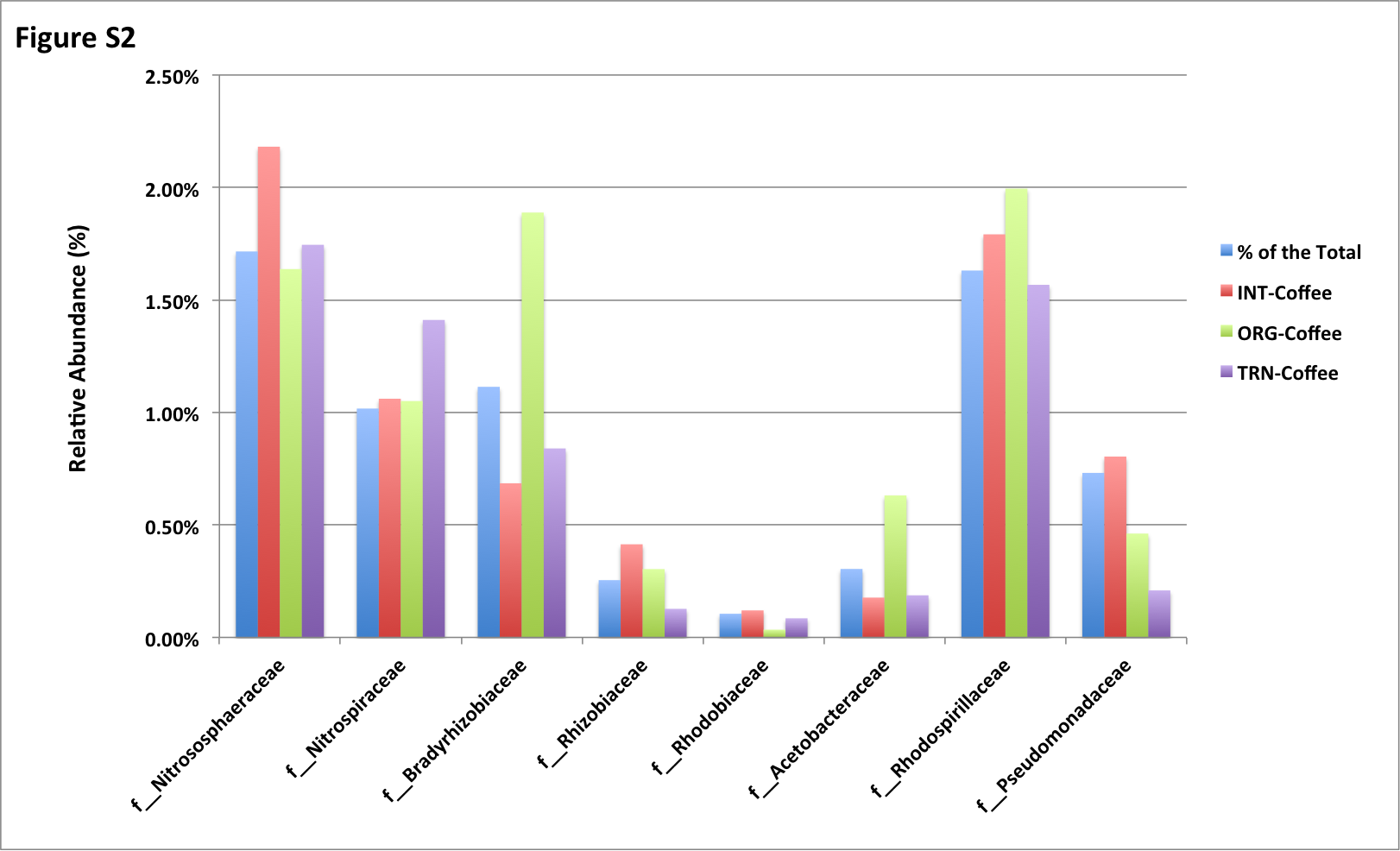

Supplement: S2 Fig — Relative abundance of nitrogen-fixing archaeal and bacterial family-level groups detected in the rhizosphere of each of the three farms (INT, ORG, and TRN) or all sites together (% of the Total). The family-level relative abundance was calculated as the percentage of sequences of each family found in the total number of sequences retrieved from each rhizosphere. The % of the Total was the number of family-level sequences divided by the total number of sequences from all sites combined. Even though a low number of sequences were retrieved from the genus level of the nitrogen-fixing bacteria, the relative abundance of the respective families were some of the highest among all families in the study. Raw 16S rRNA sequence data have been deposited at Zenodo: http://dx.doi.org/10.5281/zenodo.11120 The full mothur protocol and additional data analysis scripts can be downloaded from Zenodo: http://dx.doi.org/10.5281/zenodo.11126 (PNG) [file pone.0106355.s004.png]
